# Supplementary material for: Microautophagy regulated by STK38 and GABARAPs is essential to repair lysosomes and prevent aging
Source: EMBO Rep. 2023 Nov 21;24(12):e57300. doi: 10.15252/embr.202357300 (PMC10702834; doi:10.15252/embr.202357300)
Supplement: Supplementary file 1 — Appendix [file EMBR-24-e57300-s006.pdf]

Table of contents

Page	Content
2	**Appendix Figure S1.** Knockdown efficiency of siRNAs of Hippo pathway components.
3	**Appendix Figure S2.** STK38 is recruited to lysosomes in macroautophagy independent manner in response to lysosomal damage.
5	**Appendix Figure S3.** Recruitment of ESCRTs in STK38 KO cells.
7	**Appendix Figure S4.** Depletion of STK38 does not affect basal lysosomal integrity.
8	**Appendix Figure S5.** Knockdown efficiency of siRNAs of ESCRT components.
9	**Appendix Figure S6.** Localization of 10 candidates of STK38 substrate in LLOMe treated cells.
10	**Appendix Figure S7.** Quantification of expression levels of senescent markers in non-induced cells**.**
11	**Appendix Table S1.** siRNAs used in this study.
13	**Appendix Table S2.** qPCR primers used in this study.

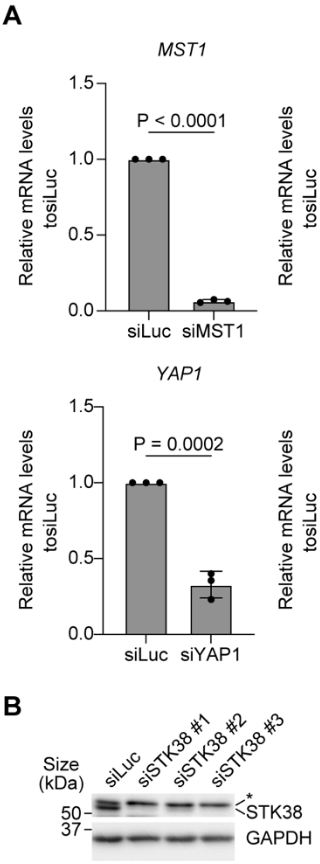

Appendix Figure S1. Knockdown efficiency of siRNAs of Hippo pathway components.

A. Relative expression levels of indicated mRNAs in siRNA-transfected HeLa cells.

B. Representative immunoblots of STK38 in siSTK38-transfected HeLa cells. The asterisk in STK38 blot represents STK38L.

Data information: All data presented as means ± SD, from $n \geq 3$ independent experiments. P-values were determined using the unpaired t-test.

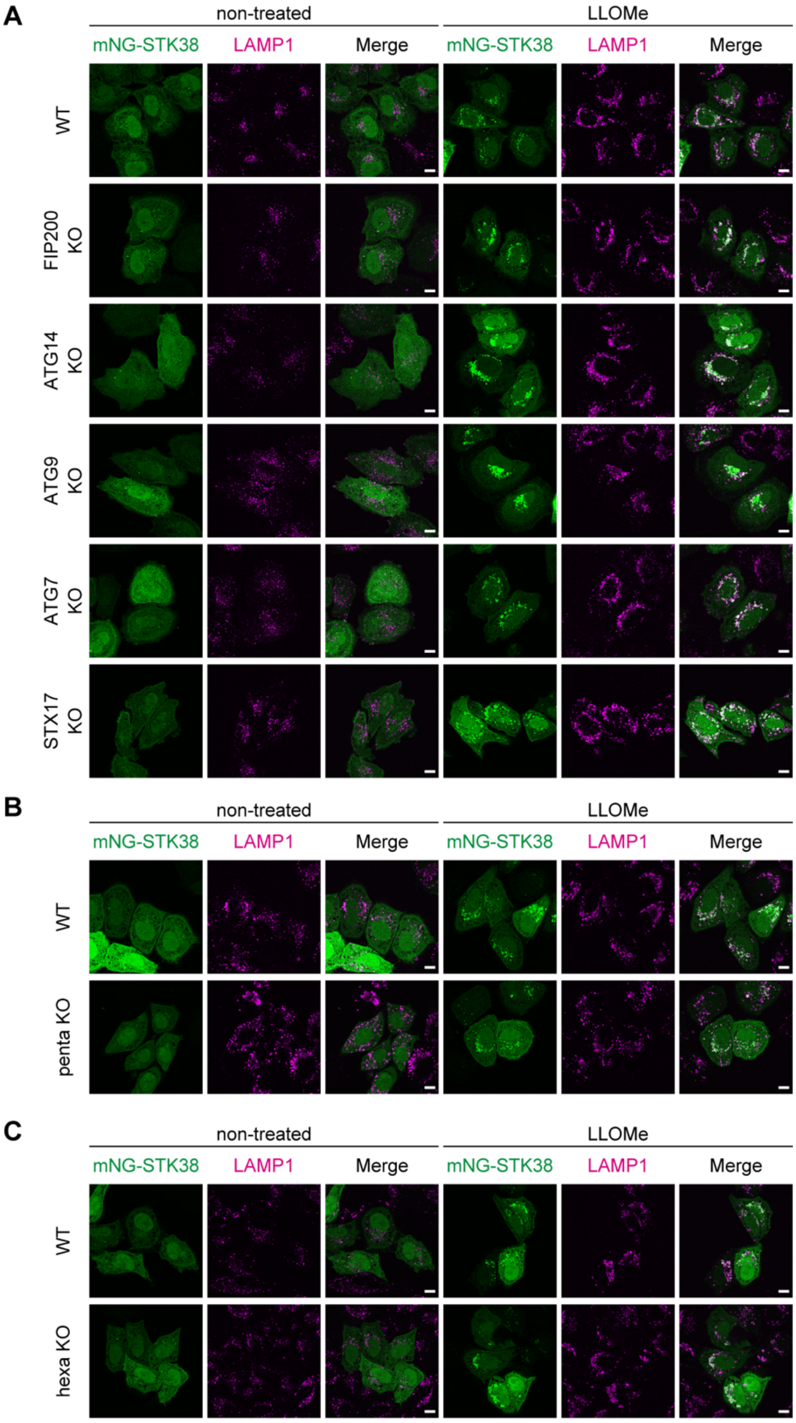

Appendix Figure S2. STK38 is recruited to lysosomes in macroautophagy independent manner in response to lysosomal damage.

A. Representative images of transiently expressed mNG-STK38 (green) and immunostained LAMP1 (magenta) in WT or macroautophagy-deficient FIP200, ATG14, ATG9, ATG7, and STX17 KO HeLa cells. Cells were treated with LLOMe (1 mM for 1 h, then incubated for 3 h after washout). Scale bars: 10 µm.
B. Representative images of transiently expressed mNG-STK38 (green) and immunostained LAMP1 (magenta) in WT or autophagy receptor penta KO HeLa cells. Cells were treated with LLOMe (1 mM for 1 h, then incubated for 3 h after washout). Scale bars: 10 µm.

C. Representative images of transiently expressed mNG-STK38 (green) and immunostained LAMP1 (magenta) in WT or ATG8 hexa KO HeLa cells. Cells were treated with LLOMe (1 mM for 1 h, then incubated for 3 h after washout). Scale bars: 10 µm.

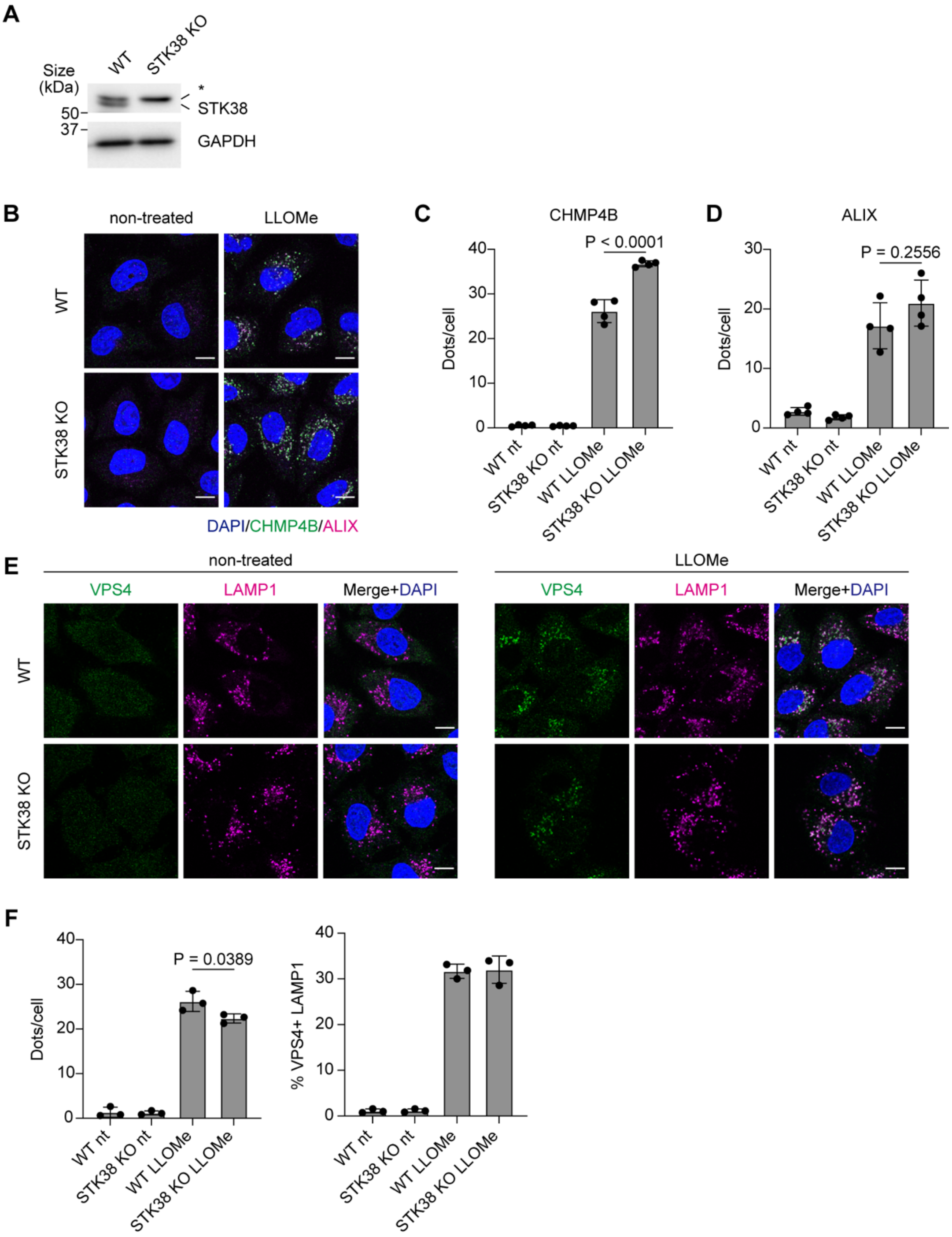

Appendix Figure S3. Recruitment of ESCRTs in STK38 KO cells.

A. Representative immunoblots of STK38 in WT or STK38 KO cells. The asterisk in STK38 blot represents STK38L.

B. Representative images of immunostained CHMP4B (green), ALIX (magenta), and DAPI (blue) in WT or STK38 KO HeLa cells. Cells were treated with LLOMe (1 mM for 30 min). Scale bars: 10 µm.

C. Quantification of CHMP4B dots shown in (B). ≥ 100 cells were analyzed per experiment for each condition.

D. Quantification of ALIX dots shown in (B). ≥ 100 cells were analyzed per experiment for each condition.

E. Representative images of immunostained VPS4 (green), LAMP1 (magenta), and DAPI (blue) in WT or

STK38 KO HeLa cells. Cells were treated with LLOMe (1 mM for 30 min). Scale bars: 10 µm.

F. Quantification of VPS4 dots (left) and co-localization between VPS4 and LAMP1 (right) shown in (E). ≥ 100 cells were analyzed per experiment for each condition.

Data information: All data presented as means ± SD, from $n \geq 3$ independent experiments. P-values were determined using one-way ANOVA with Tukey's multiple comparisons test.

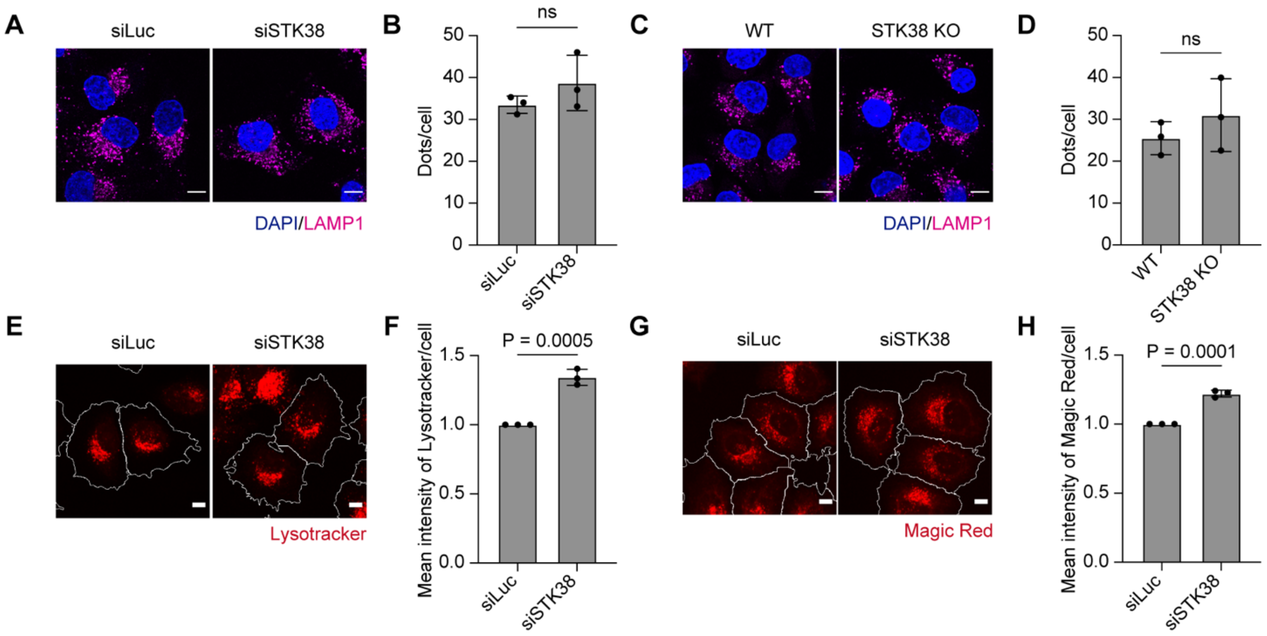

Appendix Figure S4. Depletion of STK38 does not affect basal lysosomal integrity.

A. Representative images of immunostained LAMP1 (magenta) and DAPI (blue) in siSTK38-transfected HeLa cells. Scale bars: 10 µm.

B. Quantification of LAMP1 dots shown in (A). ≥ 100 cells were analyzed per experiment for each condition.

C. Representative images of immunostained LAMP1 (magenta) and DAPI (blue) in WT or STK38 KO HeLa cells. Scale bars: 10 µm.

D. Quantification of LAMP1 dots shown in (C). ≥ 100 cells were analyzed per experiment for each condition.

E. Representative images of Lysotracker (Red) in siSTK38-transfected HeLa cells. Scale bars: 10 µm.

F. Quantification of intensity of Lysotracker per cell shown in (E). ≥ 200 cells were analyzed per experiment for each condition.

G. Representative images of Magic Red (Red) in siSTK38-transfected HeLa cells. Scale bars: 10 µm.

H. Quantification of intensity of Magic Red per cell shown in (G). ≥ 200 cells were analyzed per experiment for each condition.

Data information: All data presented as means ± SD, from $n \geq 3$ independent experiments. P-values were determined using the unpaired t-test.

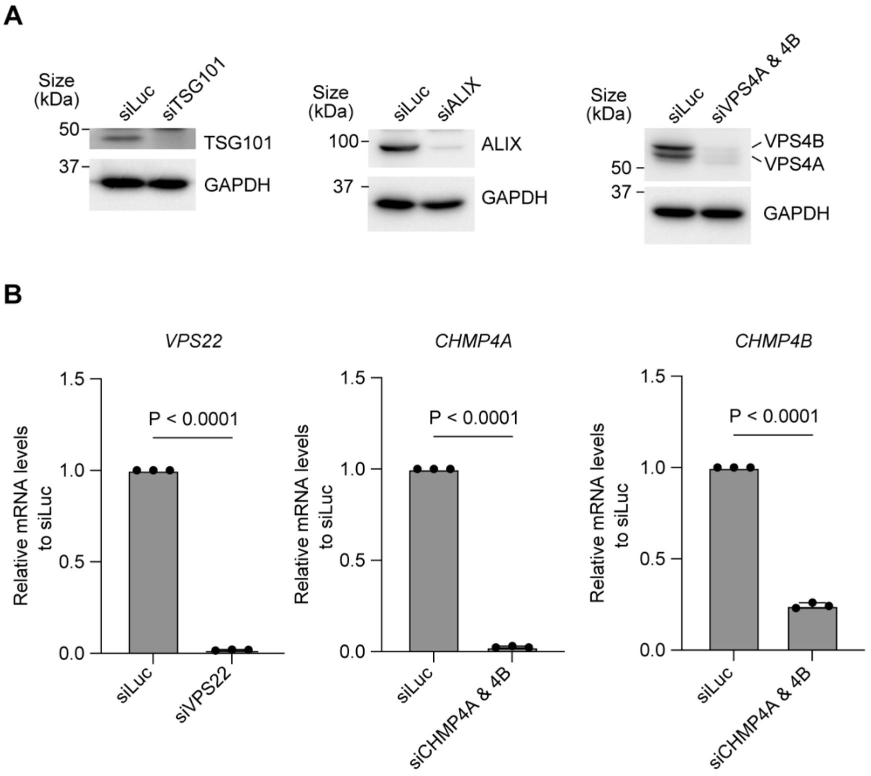

Appendix Figure S5. Knockdown efficiency of siRNAs of ESCRT components.

A. Representative immunoblots of indicated ESCRT components in siRNA-transfected MCF10A cells.

B. Relative expression levels of indicated mRNAs in siRNA-transfected MCF10A cells.

Data information: All data presented as means ± SD, from $n \geq 3$ independent experiments. P-values were determined using the unpaired t-test.

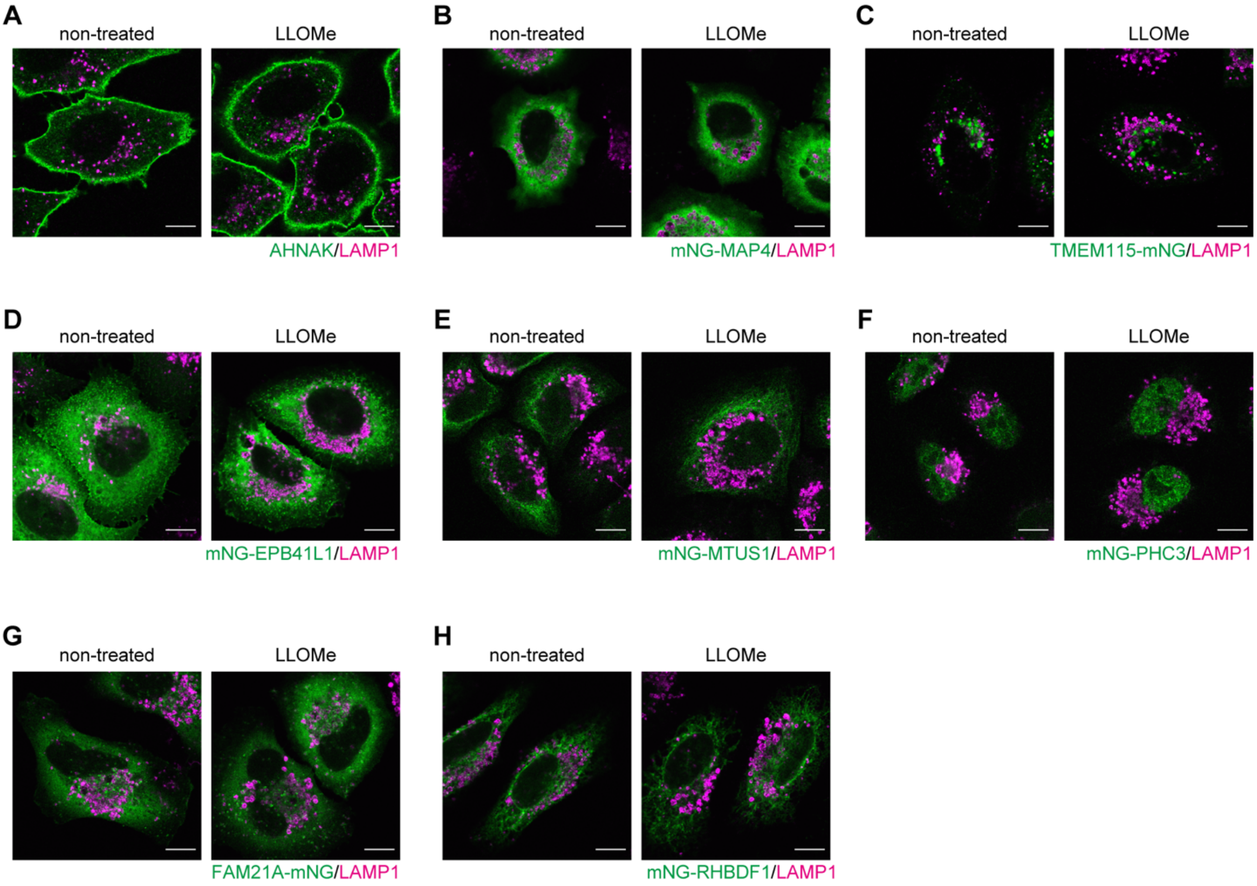

Appendix Figure S6. Localization of 10 candidates of STK38 substrate in LLOMe treated cells.

A. Representative images of immunostained AHNAK (green) and LAMP1 (magenta) in WT HeLa cells. Cells were treated with LLOMe (1 mM for 1 h). Scale bars: 10 µm.

B-H. Representative images of transiently expressed mNG-tagged candidates (green) and immunostained LAMP1 (magenta) in WT HeLa cells. Cells were treated with LLOMe (1 mM for 1 h, then incubated for 3 h after washout). Scale bars: 10 µm.

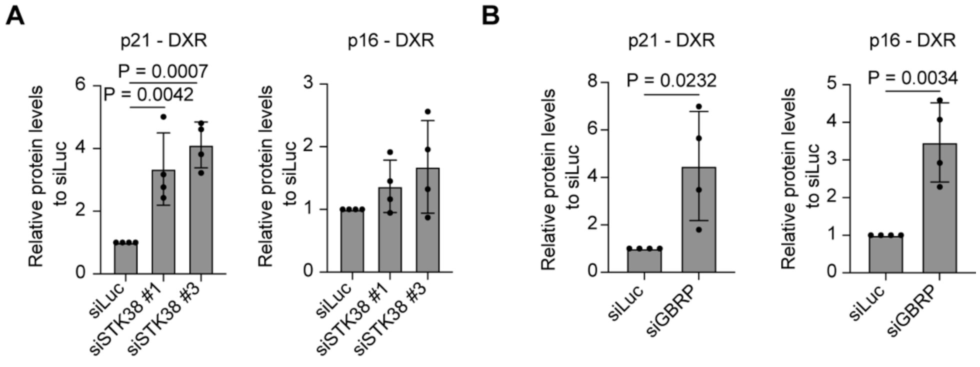

Appendix Figure S7. Quantification of expression levels of senescent markers in non-induced cells.

A. Quantification of p21 and p16 in siSTK38-transfected non-induced cells shown in Fig 7A.

B. Quantification of p21 and p16 in siGABARAP-transfected non-induced cells shown in Fig 7C.

Data information: All data presented as means ± SD, from $n \geq 3$ independent experiments. P-values were determined using one-way ANOVA with Dunnett's multiple comparisons test (A) or the unpaired t-test (B).

Appendix Table S1. siRNAs used in this study.

siRNA	Source	Identifier
siLuciferase: 5’-CGUACGCGGAAUACUUCGAdTdT-3’	Sigma-Aldrich	Custom
siMST1: 5’-GACUGAUGGAGCCAAUACUdTdT-3	Sigma-Aldrich	Custom
siMST2	Sigma-Aldrich	SASI_Hs01_00015485
siMST3	Sigma-Aldrich	SASI_Hs01_00146086
siLATS1	Sigma-Aldrich	SASI_Hs01_00046130
siLATS2	Sigma-Aldrich	SASI_Hs01_00158804
siYAP1	Sigma-Aldrich	SASI_Hs01_00182402
siSTK38 #1: 5’-CGUCGGCCAUAAACAGCUdTdT-3’	Sigma-Aldrich	Custom (Joffre *et al*, 2015)
siSTK38 #2: 5’-GUAAUAGGCAGAGGAGCAdTdT-3’	Sigma-Aldrich	Custom (Joffre *et al.*, 2015)
siSTK38 #3	Sigma-Aldrich	SASI_Hs01_00224995
siSTK38L	Sigma-Aldrich	SASI_Hs01_00074234
siMOB1A	Sigma-Aldrich	SASI_Hs01_00231852
siMOB1B	Sigma-Aldrich	SASI_Hs01_00171109
siMOB2	Sigma-Aldrich	SASI_Hs01_00177401
siTSG101: 5’-CCUCCAGUCUUCUCUCGUCdTdT-3’	Sigma-Aldrich	Custom (Skowyra *et al*, 2018)
siALIX: 5’-GAAGGAUGCUUUCGAUAAAdTdT-3’	Sigma-Aldrich	Custom
siVPS22: 5’-CUUGCAGAGGCCAAGUAUAdTdT-3’	Sigma-Aldrich	Custom (Zhang *et al*, 2021)
siCHMP4A	Sigma-Aldrich	SASI_Hs01_00039745
siCHMP4B	Sigma-Aldrich	SASI_Hs01_00129003
siVPS4A: 5’-CUGUGGUUUGCAUGUCGGAdTdT-3’	Sigma-Aldrich	Custom (Mejlvang *et al*, 2018)
siVPS4B: 5’-CCAAAGAAGCACUGAAAGAdTdT-3’	Sigma-Aldrich	Custom (Mejlvang *et al.*, 2018)
siGABARAP	Sigma-Aldrich	SASI_Hs01_00197526
siGABARAPL1	Sigma-Aldrich	SASI_Hs01_00200246
siGABARAPL2	Sigma-Aldrich	SASI_Hs01_00165606
siMAP4	Thermo Fisher Scientific	4427030; s200446

siPSMA5	Thermo Fisher Scientific	4427030; s11339
siEPHA2	Thermo Fisher Scientific	4427030; s4565
siSOS1	Thermo Fisher Scientific	4427030; s13286
siARHGAP1	Thermo Fisher Scientific	4427030; s1584
siGOLGA3	Thermo Fisher Scientific	4427030; s5946
siAHNAK	Thermo Fisher Scientific	4427030; s200548
siTMEM115	Thermo Fisher Scientific	4427030; s21818
siARHGAP21	Thermo Fisher Scientific	4427030; s33373
siFAM21A	Thermo Fisher Scientific	4427030; s51898
siRHBDF2	Thermo Fisher Scientific	4427030; s36014
siPHLDB2	Thermo Fisher Scientific	4427030; s40251
siZZZ3	Thermo Fisher Scientific	4427030; s24923
siTAB3	Thermo Fisher Scientific	4427030; s223653
siEHBP1	Thermo Fisher Scientific	4427030; s225944
siPHC3	Thermo Fisher Scientific	4427030; s36819
siGOLGA5	Thermo Fisher Scientific	4427030; s19321
siRHBDF1	Thermo Fisher Scientific	4427030; s34612
siPOM121	Thermo Fisher Scientific	4427030; s19145
siDOK1	Thermo Fisher Scientific	4427030; s4236
siEPB41L1	Thermo Fisher Scientific	4427030; s4706
siNCKAP5L	Thermo Fisher Scientific	4427030; s49193
siBCLAF1	Thermo Fisher Scientific	4427030; s225184
siMTUS1	Thermo Fisher Scientific	4427030; s33189
siBTRC	Thermo Fisher Scientific	4427030; s17111
Negative Control	Thermo Fisher Scientific	4390843

Appendix Table S2. qPCR primers used in this study.

Target	Forward (5'-3')	Reverse (5'-3')
MST1	GCCCCCTTATGCTGATATCCAT	GGGAGGAGGATTTGTAGGAATCAT
MST2	AACTGTGTGGCCGACATCTG	GAGGTTTTCCTTCAGCCATTTCT
MST3	GACATTAAAGCGGCCAACGT	CAAAGTCCGCCAGCTTCAC
LATS1	CATTCCACCACAAGCTAAACTCAGT	GGGTCCTCGGCAAAGTTTAAT
LATS2	GAATTTCGACCCCGTAGATGAA	GTGTGTCCCAGGCCTTGGT
YAP1	CCAGTGCAGCAGAATATGATGAA	GGCTTGTTCCCATCCATCAG
STK38L	CACAACACGCTCGCAAAGA	TCCAAGCCAAGTCTGGTCCTT
MOB1A	TGTCCCATTTCCCAAAAACTTT	AACCCTGAACAGACGCTTTAGAA
MOB1B	GGTTCAGGACCAGTTGGATGA	TGGGAACGGGACACCAAT
MOB2	GGAGCTGCACGGACACTTG	CTCCCGAGCAAAGAGGATGA
VPS22	ATCAGAGCCATCAAGAAACTAAAGG	CCGCCCACAGGGATGA
CHMP4A	ACAGCAGCTGGCACAAACTG	TGGCCTCACGCTGAAACTC
CHMP4B	AGGCGGCCCATGACAA	CAGCAATGTCCTGCATTAACTCA
DOK1	TGGCGCCTACCGATAACC	TGTACAAGGAGTTCTCCAGCATCT
IL-6	CCAGGAGCCCAGCTATGAAC	CCCAGGGAGAAGGCAACTG
IL-1α	AACCAGTGCTGCTGAAGGA	TTCTTAGTGCCGTGAGTTTCC
GAPDH	TGCACCACCAACTGCTTAGC	GGCATGGACTGTGGTCATGAG
