# Supplementary material for: Microautophagy regulated by STK38 and GABARAPs is essential to repair lysosomes and prevent aging
Source: EMBO Rep. 2023 Nov 21;24(12):e57300. doi: 10.15252/embr.202357300 (PMC10702834; doi:10.15252/embr.202357300)
Supplement: Supplementary file 2 — Expanded View Figures PDF [file EMBR-24-e57300-s003.pdf]

# Expanded View Figures

**Figure EV1. siRNA-based screening identifies several Hippo pathway components required for clearance of damaged lysosomes.**

A Representative images of stably expressed GFP-Gal3 (green) in HeLa cells transfected with siRNAs of Hippo pathway components. Cells were treated with LLOMe (1 mM for 1 h, then incubated for the indicated number of hours after washout). Scale bars: 10 μm.
B Percentage of residual GFP-Gal3 dots (10 h/0 h [%]) in (A). ≥ 100 cells were analyzed per experiment for each condition.
C Representative images of transiently expressed mNG-MOB2 (green) and immunostained LAMP1 (magenta) in HeLa cells treated with LLOMe (1 mM for 1 h, then incubated for 3 h after washout) or EBSS (for 4 h). Scale bars: 10 μm.
D Representative images of transiently expressed mNG-STK38 (green), immunostained LAMP1 (magenta) and Gal3 (cyan) in HeLa cells treated with LLOMe (1 mM for 1 h, then incubated for 3 h after washout). Scale bars: 10 μm.
E Representative immunoblot of phosphorylated STK38 T444 (pT444) in HeLa cells. Cells were treated with LLOMe (1 mM for 30 min). Asterisks in STK38 and STK38 pT444 blots represent STK38L and STK38L pT442, respectively.
F Quantification of phosphorylation of STK38 T444 shown in (E).

Data information: All data presented as means ± SD, from $n \geq 3$ independent experiments. *P*-values were determined using one-way ANOVA with Dunnett's multiple comparisons test (B) or the unpaired *t*-test (F).

 © 2023 The Authors

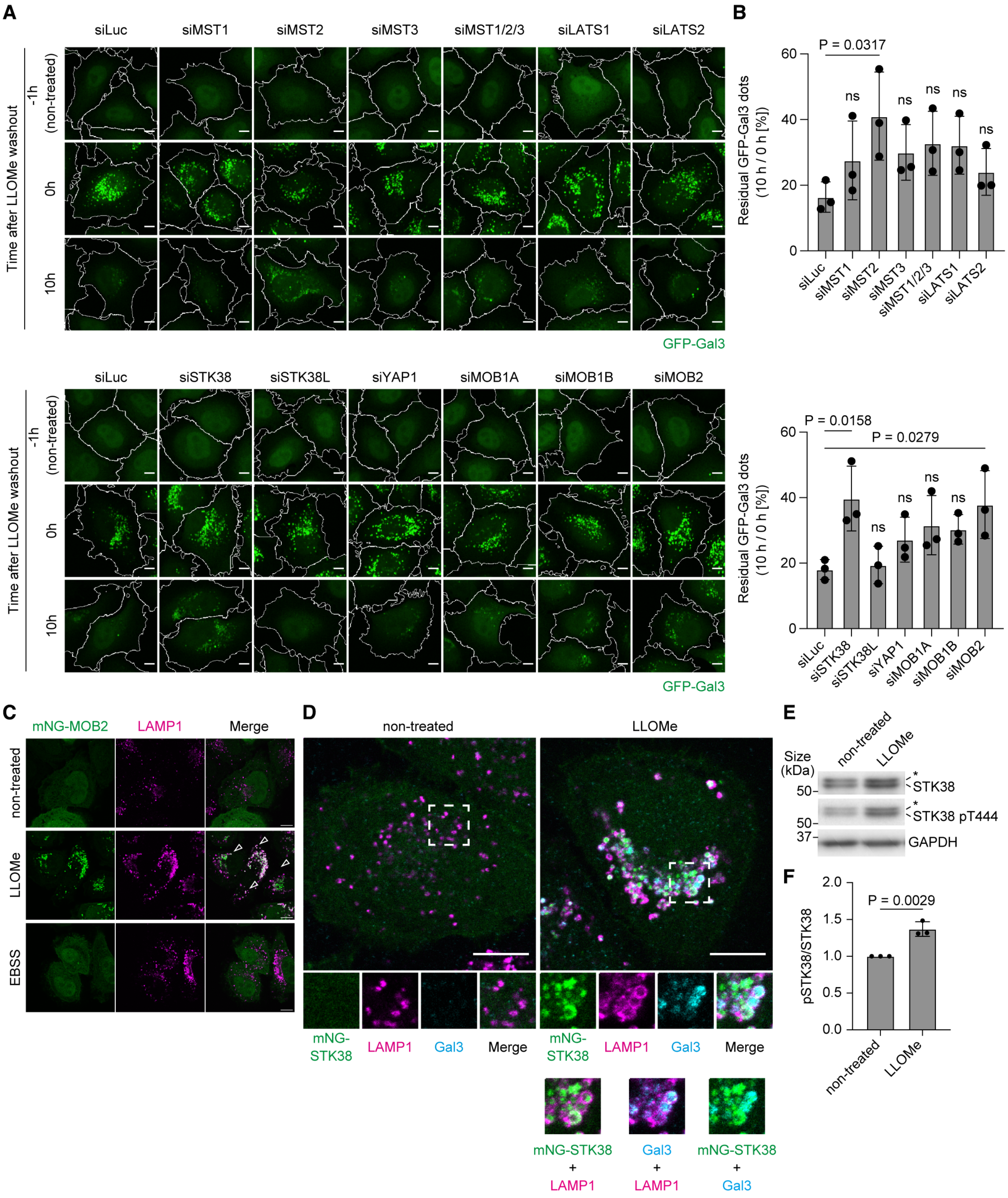
A
siLuc
siMST1
siMST2
siMST3
siMST1/2/3
siLATS1
siLATS2
Time after LLOMe washout
-1h (non-treated)
0h
10h
GFP-Gal3
B
P = 0.0317
ns
Residual GFP-Gal3 dots (10 h / 0 h [%])
siSTK38
siSTK38L
siYAP1
siMOB1A
siMOB1B
siMOB2
P = 0.0158
P = 0.0279
C
mNG-MOB2
LAMP1
Merge
non-treated
LLOMe
EBSS
D
mNG-STK38
Gal3
mNG-STK38 + LAMP1
Gal3 + LAMP1
mNG-STK38 + Gal3
E
Size (kDa)
STK38
STK38 pT444
GAPDH
F
P = 0.0029
pSTK38/STK38

**Figure EV1.**

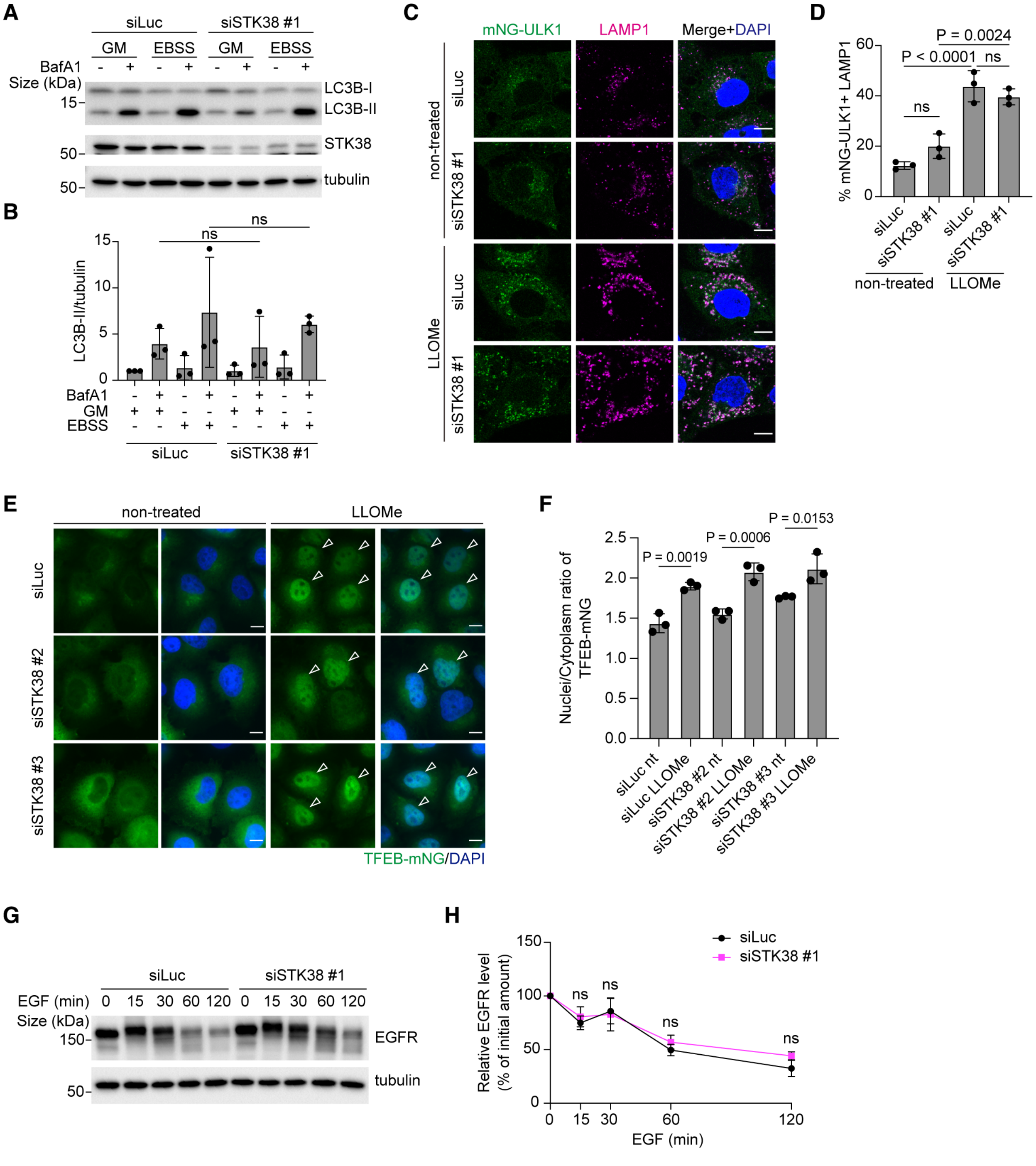


**Figure EV2.**

 © 2023 The Authors

**Figure EV2. STK38 is not required for macroautophagy, TFEB activation, and EGF-stimulated MVB formation.**

A Representative immunoblots of LC3B in siSTK38-transfected U2OS cells. Cells were incubated in normal growth media (GM) or EBSS with or without bafilomycinA1 (BafA1) for 2 h.
B Quantification of LC3B shown in (A).
C Representative images of stably expressed mNG-ULK1 (green), LAMP1 (magenta), and DAPI (blue) in siSTK38-transfected HeLa cells. Cells were treated with LLOMe (1 mM for 1 h, then incubated for 3 h after washout). Scale bars: 10 μm.
D Quantification of co-localization between mNG-ULK1 and LAMP1 shown in (C). $\geq$ 50 cells were analyzed per experiment for each condition.
E Representative images of stably expressed TFEB-mNG (green) and DAPI (blue) in siSTK38-transfected HeLa cells. Cells were treated with LLOMe (1 mM for 1 h, then incubated for 3 h after washout). Scale bars: 10 μm.
F The nucleus/cytoplasm ratio of TFEB-mNG in non-treated (nt) or LLOMe treated cells shown in (E). $\geq$ 80 cells were analyzed per experiment for each condition.
G Representative immunoblot of EGFR in siSTK38-transfected HeLa cells. Cells were treated with EGF (50 ng/ml for the indicated number of minutes).
H Quantification of EGFR shown in (G).

Data information: All data presented as means $\pm$ SD, from $n \geq 3$ independent experiments. $P$-values were determined using the unpaired $t$-test (B and H) or one-way ANOVA with Tukey's multiple comparisons test (D and F).
Source data are available online for this figure.

**Figure EV3. ATG8 lipidation–dependent degradation of TRPML1 by microautophagy is occurred in endolysosomes in response to lysosomal stress or damage stimulation.**

A Representative immunoblots of EGFP-TRPML1 in WT, ATG5 KO, and ATG13 KO MCF10A cells. Cells were treated with monensin (10 μM), nigericin (5 μM), or LLOMe (0.5 mM) for 8 h.
B Quantification of cleaved EGFP shown in (A).
C Representative images of macroautophagy-related factors (LC3, Gal3, ubiquitin-K48 chain (Ub-K48), and p62, shown in green), LAMP1 (magenta), and DAPI (blue) in MCF10A cells. Cells were treated with LLOMe (0.5 mM for 1 h), nigericin (5 μM for 2 h), monensin (10 μM for 2 h), or $NH_4Cl$ (5 mM for 2 h). Scale bars: 10 μm.
D Representative images of stably expressed EGFP-TRPML1 (green), immunostained LAMP1 (magenta), and DAPI (blue) in MCF10A cells. Cells were treated with monensin (50 μM) for 1 h, followed by the co-treatment with Apilimod (200 nM) for 2 h. Scale bars: 10 μm.
E Representative images of stably expressed EGFP-TRPML1 (green), immunostained CD63 (magenta), and DAPI (blue) in MCF10A cells. Cells were treated with monensin (50 μM) for 1 h, followed by the co-treatment with Apilimod (200 nM) for 2 h. Scale bars: 10 μm.

Data information: All data presented as means $\pm$ SD, from $n \geq 3$ independent experiments. $P$-values were determined using one-way ANOVA with Tukey's multiple comparisons test.

© 2023 The Authors

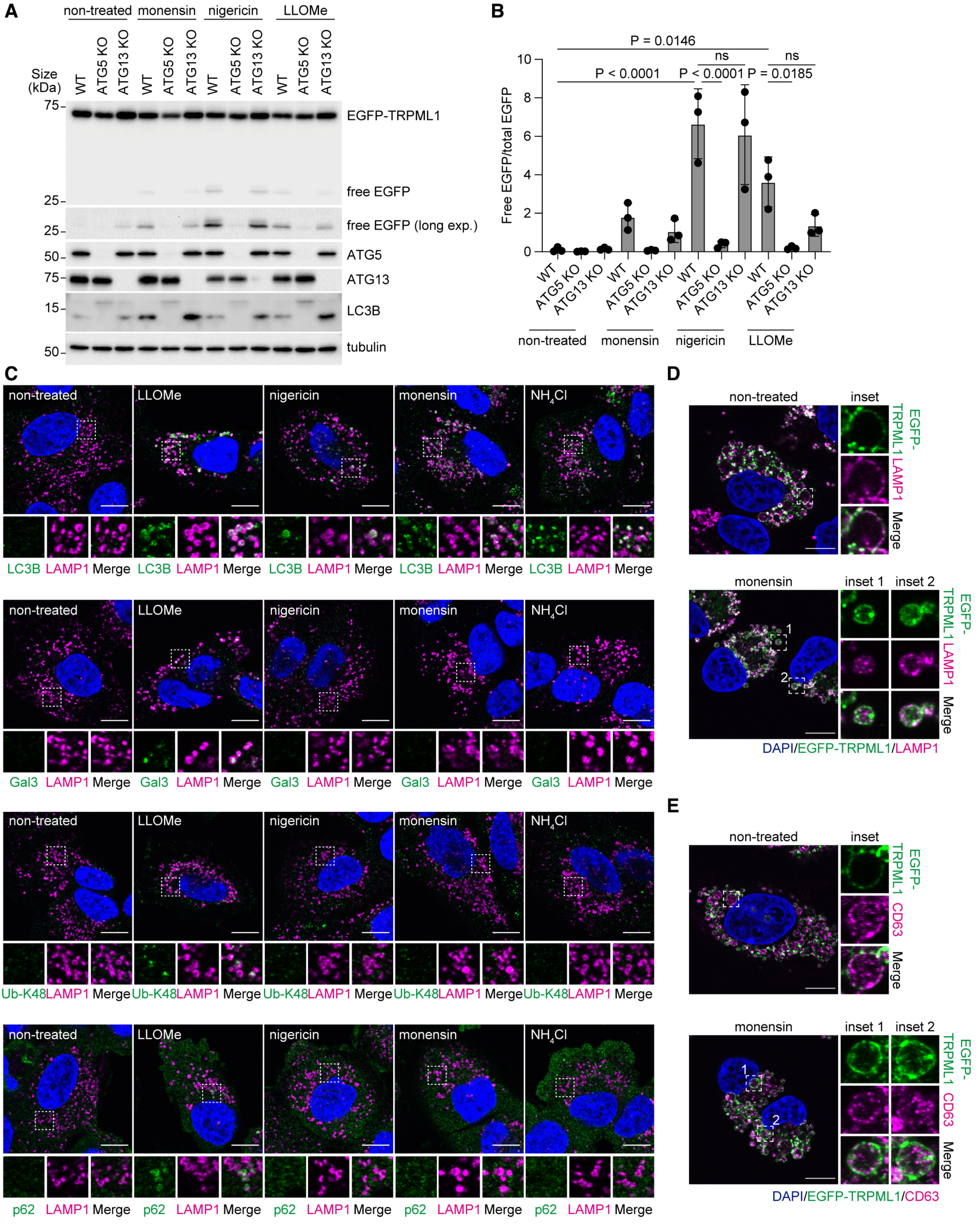


**Figure EV3.**

 © 2023 The Authors

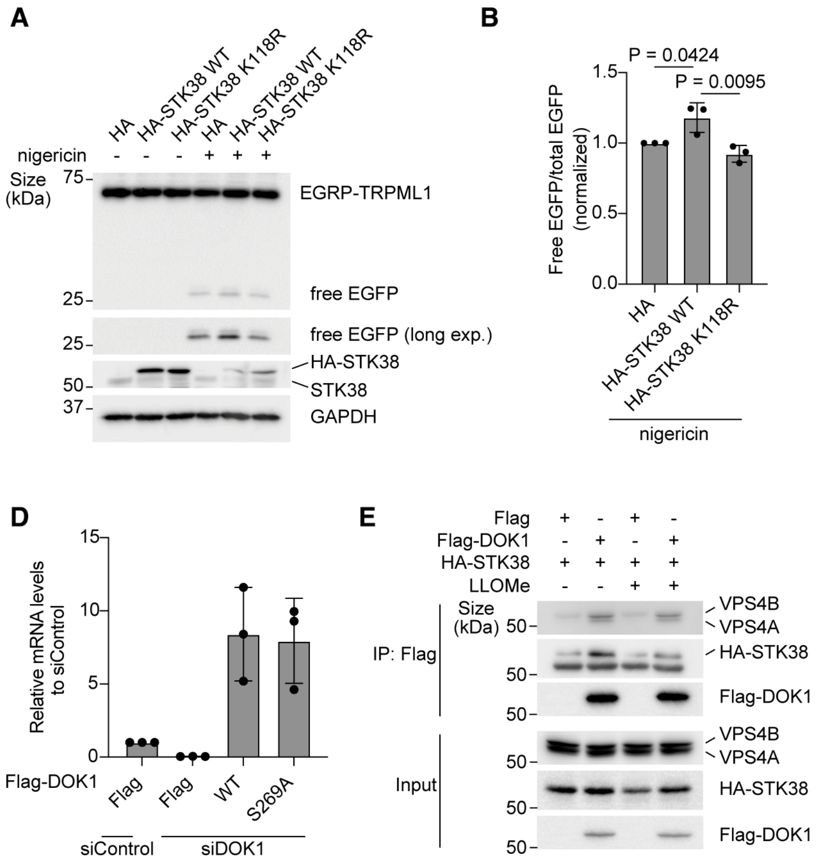


**Figure EV4. STK38 kinase activity is essential for microlysophagy.**

A Representative immunoblots of EGFP-TRPML1 in MCF10A cells expressing HA tag only, WT HA-STK38, or kinase-dead HA-STK38 (K118R). Cells were treated with nigericin (50 μM for 8 h).
B Quantification of cleaved EGFP shown in (A).
C Representative images of transiently expressed kinase-dead K118R mutant of mNG-STK38 (green) and immunostained LAMP1 (magenta) in HeLa cells treated with LLOMe (1 mM for 1 h, then incubated for 3 h after washout). Scale bars: 10 μm.
D Relative expression levels of *DOK1* mRNA in DOK1-reconstituted HeLa cells shown in Fig 5C and D.
E Representative immunoblots from a co-immunoprecipitation experiment. HeLa cells expressing Flag-DOK1 were treated with LLOMe (1 mM for 30 min). Cell lysates were immunoprecipitated using anti-Flag-beads agarose.

Data information: All data presented as means ± SD, from $n \geq 3$ independent experiments. *P*-values were determined using one-way ANOVA with Tukey's multiple comparisons test (B).
Source data are available online for this figure.

© 2023 The Authors

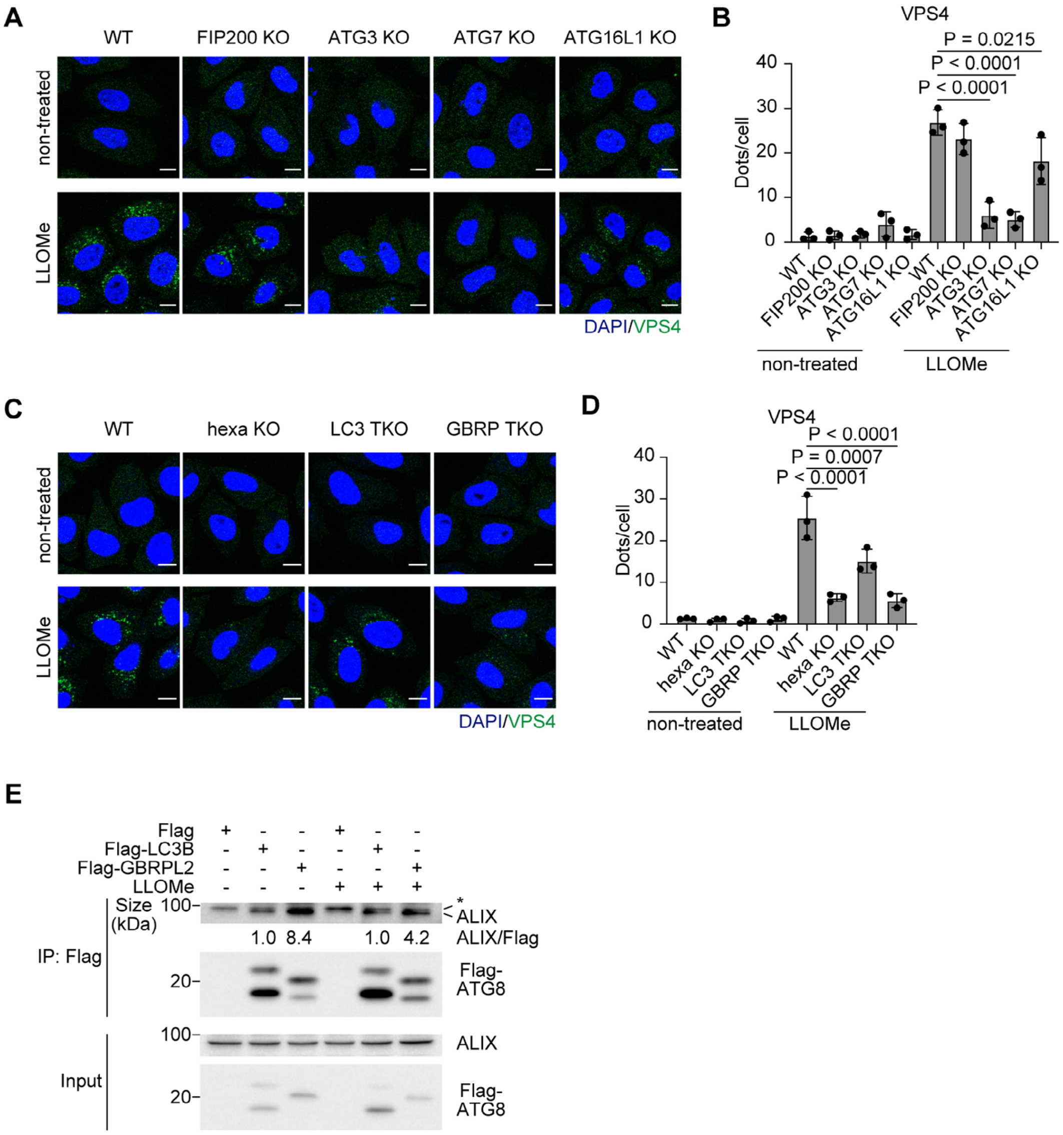


**Figure EV5. Among ATG8s, GABARAPs interact preferentially with ALIX and contribute to subsequent VPS4 recruitment to damaged lysosomes.**

A Representative images of immunostained VPS4 (green) and DAPI (blue) in WT, FIP200, ATG3, ATG7, and ATG16L1 KO HeLa cells. Cells were treated with LLOMe (1 mM for 30 min). Scale bars: 10 μm.

B Quantification of VPS4 dots shown in (A). ≥ 100 cells were analyzed per experiment for each condition.

C Representative images of immunostained VPS4 (green) and DAPI (blue) in WT, ATG8 hexa KO, LC3 TKO, and GABARAP TKO HeLa cells. Cells were treated with LLOMe (1 mM for 30 min). Scale bars: 10 μm.

D Quantification of VPS4 dots shown in (C). ≥ 100 cells were analyzed per experiment for each condition.

E Representative immunoblots from a co-immunoprecipitation experiment. Flag-LC3B- or GABARAPL2-expressing hexa KO HeLa cells were treated with LLOMe (1 mM for 30 min). Cell lysates were immunoprecipitated using anti-Flag-beads agarose. Fold changes in co-immunoprecipitated ALIX were quantified (Fold change vs. non-treated Flag-LC3B, normalized to Flag). The asterisk in ALIX blot represents a non-specific band.

Data information: All data presented as means ± SD, from $n \geq 3$ independent experiments. *P*-values were determined using one-way ANOVA with Tukey's multiple comparisons test.
Source data are available online for this figure.

 © 2023 The Authors
