# Supplementary material for: Microautophagy regulated by STK38 and GABARAPs is essential to repair lysosomes and prevent aging
Source: EMBO Rep. 2023 Nov 21;24(12):e57300. doi: 10.15252/embr.202357300 (PMC10702834; doi:10.15252/embr.202357300)
Supplement: Supplementary file 3 — PDF+ [file EMBR-24-e57300-s004.pdf]

Article

SOURCE DATA | TRANSPARENT PROCESS | OPEN ACCESS

EMBO reports

Microautophagy regulated by STK38 and GABARAPs is essential to repair lysosomes and prevent aging

Monami Ogura[1], Tatsuya Kaminishi[2], Takayuki Shima[2,†], Miku Torigata[2], Nao Bekku[2], Keisuke Tabata[1,2], Satoshi Minami[2], Kohei Nishino[3], Akiko Nezu[2], Maho Hamasaki[1,2], Hidetaka Kosako[3], Tamotsu Yoshimori[1,2,4,*] & Shuhei Nakamura[1,2,5,**,†]

Abstract

Lysosomes are degradative organelles and signaling hubs that maintain cell and tissue homeostasis, and lysosomal dysfunction is implicated in aging and reduced longevity. Lysosomes are frequently damaged, but their repair mechanisms remain unclear. Here, we demonstrate that damaged lysosomal membranes are repaired by microautophagy (a process termed "microlysophagy") and identify key regulators of the first and last steps. We reveal the AGC kinase STK38 as a novel microlysophagy regulator. Through phosphorylation of the scaffold protein DOK1, STK38 is specifically required for the lysosomal recruitment of the AAA+ ATPase VPS4, which terminates microlysophagy by promoting the disassembly of ESCRT components. By contrast, microlysophagy initiation involves non-canonical lipidation of ATG8s, especially the GABARAP subfamily, which is required for ESCRT assembly through interaction with ALIX. Depletion of STK38 and GABARAPs accelerates DNA damage-induced cellular senescence in human cells and curtails lifespan in *C. elegans*, respectively. Thus, microlysophagy is regulated by STK38 and GABARAPs and could be essential for maintaining lysosomal integrity and preventing aging.

Keywords ESCRT; lysosome; microautophagy; non-canonical ATG8 lipidation
Subject Categories Autophagy & Cell Death; Molecular Biology of Disease; Organelles
DOI 10.15252/embr.202357300 | Received 5 April 2023 | Revised 24 October 2023 | Accepted 25 October 2023
EMBO Reports (2023) e57300

Introduction

Lysosomes are the main digestive organelles and serve as a signaling hub linking environmental cues to cellular metabolism (Ballabio & Bonifacino, 2020). Through these functions, lysosomes play crucial roles in maintaining cell and tissue homeostasis. Lysosomal dysfunction is associated with age-associated pathologies, cellular senescence, and a decline in lifespan, suggesting that preserving lysosomal integrity is important for delaying the aging process (Li *et al*, 2016; Ballabio & Bonifacino, 2020; Johmura *et al*, 2021).

Lysosomes are often damaged by various intra- and extracellular factors such as crystals, amyloid, pathogens, lipids, lysosomotropic drugs, and reactive oxygen species (Terman *et al*, 2006; Rajamäki *et al*, 2010; Maejima *et al*, 2013; Gabandé-Rodríguez *et al*, 2014; Chauhan *et al*, 2016; Papadopoulos *et al*, 2017; Bussi *et al*, 2018). Damaged lysosomes release luminal enzymes and protons, which can induce oxidative stress, inflammation, and cell death, and thus are detrimental to cells and tissues (Wang *et al*, 2018). Accumulation of damaged lysosomes therefore contributes to aging and prominent diseases such as crystal nephropathy and neurodegenerative and infectious diseases (Chauhan *et al*, 2016; Li *et al*, 2016; Papadopoulos & Meyer, 2017; Nakamura *et al*, 2020). To restore lysosomal function after damage, cells have developed several defense mechanisms collectively called "lysosomal damage responses." We and others have previously determined that the induction of lysosome-targeting selective macroautophagy (called "macrolysophagy") and the activation of TFEB, a master transcriptional regulator of lysosomal biogenesis, are parts of the lysosomal damage response (Sardiello *et al*, 2009; Thurston *et al*, 2012; Maejima *et al*, 2013; Nakamura *et al*, 2020). During macrolysophagy, lysosomes with a relatively large amount of damage are first engulfed by double-membraned autophagosomes associated with the key autophagy proteins ATG8s and then delivered to remaining normal lysosomes for sequestration. Concomitant activation of TFEB-mediated lysosomal biogenesis is essential to supply functional lysosomes for progression of macrolysophagy. In addition, it has been shown that lysosomes with small membrane ruptures are rapidly repaired by several mechanisms such as endosomal sorting complex required for transport (ESCRT) machinery and lipid transport from ER (Radulovic *et al*, 2018, 2022; Skowyra *et al*, 2018; Tan & Finkel, 2022).

1 Department of Intracellular Membrane Dynamics, Graduate School of Frontier Biosciences, Osaka University, Osaka, Japan
2 Department of Genetics, Graduate School of Medicine, Osaka University, Osaka, Japan
3 Fujii Memorial Institute of Medical Sciences, Institute of Advanced Medical Sciences, Tokushima University, Tokushima, Japan
4 Integrated Frontier Research for Medical Science Division, Institute for Open and Transdisciplinary Research Initiatives (OTRI), Osaka University, Osaka, Japan
5 Institute for Advanced Co-Creation Studies, Osaka University, Osaka, Japan
*Corresponding author. Tel: +81 6 6879 3580; E-mail: yoshimori.tamotsu.med@osaka-u.ac.jp
**Corresponding author. Tel: +81 744 29 8837; E-mail: shuhei.nakamura@naramed-u.ac.jp
†Present address: Department of Biochemistry, Nara Medical University, Nara, Japan

© 2023 The Authors. Published under the terms of the CC BY 4.0 license

ESCRT machinery consists of functionally distinct complexes (ESCRT-I, II, and III) and VPS4 (Schöneberg *et al*, 2017; Vietri *et al*, 2020). These components are recruited to damaged lysosomes soon after lysosomal damage, where they repair the damaged lysosomal membrane. Efflux of Ca^{2+} from damaged lysosomes or reduction of lysosomal membrane tension has been proposed as a trigger of ESCRT recruitment (Skowyra *et al*, 2018; Mercier *et al*, 2020). Previous reports have shown that this repair is independent of HRS or PtdIns3P, which are involved in intraluminal vesicle (ILV) formation by ESCRTs, suggesting a distinct repair mechanism from ILV formation (Radulovic *et al*, 2018; Skowyra *et al*, 2018). Nevertheless, the exact mechanisms of ESCRT component recruitment and repair of damaged lysosomes remain elusive. A recent study identified lysosomal membrane turnover caused by another type of autophagy, microautophagy, which incorporates cytoplasmic materials into the lysosomal lumen by direct invagination of the lysosomal membrane (Lee *et al*, 2020). This pathway (termed “microlysophagy”) is induced by lysosomal stressors such as lysosomal damage and osmotic stress and contributes to lysosomal homeostasis by regulating lysosomal size and function. In multicellular organisms, less is known about the molecular mechanism and the physiological significance of microautophagy compared to macroautophagy, but non-canonical (macroautophagy-independent) lipidation of ATG8s on damaged lysosomes has been shown to be required for microlysophagy (Lee *et al*, 2020). However, no studies thus far have explored the means by which non-canonically lipidated lysosomal ATG8s regulate microlysophagy, or the functional relationship between ESCRT-dependent repair and microlysophagy.

In this study, we demonstrated that lysosomal membrane repair by ESCRT machinery is a part of microlysophagy. We identified key factors regulating the distinct steps of microlysophagy, and the critical roles of these factors in lysosomal homeostasis and aging. Serine–threonine kinase 38 (STK38, also known as NDR1), an AGC kinase previously known to play a role in the Hippo pathway, was specifically recruited to damaged lysosomes and repaired damaged membranes through microlysophagy. While STK38 regulated disassembly of ESCRTs through phosphorylation of a scaffold protein DOK1 and subsequent recruitment of VPS4, non-canonically lipidated ATG8s, mainly GABARAPs, were required for the assembly of ESCRTs on damaged lysosomes via interaction with ALIX, an early-acting ESCRT component. Remarkably, depletion of STK38 or GABARAPs accelerated cellular senescence and organismal aging with an increase in damaged lysosomes, implying that the maintenance of lysosomal integrity by microlysophagy could be critical to prevent aging.

Results

STK38 is required for lysosomal damage responses

Although several studies have suggested an association between the Hippo pathway and autophagy (Wang *et al*, 2020), it remains elusive whether this pathway plays a pivotal role during the lysosomal damage response. The Hippo pathway consists of the following: the transcription factor YAP; NDR kinases (STK38, STK38L, LATS1, and LATS2) that phosphorylate YAP; upstream kinases (MST1/2/3); and MOB proteins (MOB1A, MOB1B, and MOB2), which are co-activators of NDR kinases (Hergovich *et al*, 2006). To test whether any of these factors is required for the clearance of damaged lysosomes, we induced lysosomal damage by L-leucyl-L-leucine methyl ester (LLOMe), a well-characterized lysosomotropic reagent (Uchimoto *et al*, 1999), and monitored the turnover of damaged lysosomes in cells after knockdown of each factor. As a marker of damaged lysosomes, we used GFP-tagged Galectin-3 (Gal3), which is a beta-galactoside–binding lectin that can also bind to glycans on the luminal side of ruptured lysosomes (Maejima *et al*, 2013). This screening revealed that depletion of MST2, STK38, and MOB2 significantly increased the Gal3 dot residual rate, indicating the suppression of lysosomal damage response (Fig EV1A and B, Appendix Fig S1). Among them, STK38 is thought to play a role in macroautophagy (Joffre *et al*, 2015; Klimek *et al*, 2019; Martin *et al*, 2019); however, its function in the lysosomal damage response and its physiological relevance are completely unknown. Moreover, it has been reported that MST2 and MOB2 are an upstream kinase and co-factor of STK38, respectively (Hergovich *et al*, 2006; Duhart & Raftery, 2020). Indeed, we found that mNeonGreen (mNG)-tagged STK38 and MOB2 colocalized with the lysosomal marker LAMP1 by lysosomal damage (LLOMe) but not by starvation conditions (EBSS), suggesting that these factors preferentially play a role in the lysosomal damage response (Figs 1A and EV1C). Of note, we confirmed that STK38 was partially colocalized with Gal3 positive damaged lysosomes after LLOMe treatment (Fig EV1D). In addition, the phosphorylation of STK38 T444 which is crucial for the kinase activity was increased after LLOMe treatment suggesting the activation of this kinase in response to lysosomal damage (Fig EV1E and F). Given these facts and our own results, it is possible that MST2-STK38-MOB2 cascade exists to regulate lysosomal damage response and STK38 would be the core of this cascade, which prompted us to further characterize the role of STK38.

STK38 is recruited to lysosomes in response to lysosomal damage

First, we examined the mechanism whereby lysosomal localization of STK38 is regulated in the context of lysosomal damage. Lysosomes are known to be intracellular stores for Ca^{2+}, and Ca^{2+} efflux from damaged lysosomes triggers lysosomal damage responses that induce recruitment of ESCRT components and TFEB activation (Skowyra *et al*, 2018; Nakamura *et al*, 2020). Since a previous study suggested that activation of STK38 is Ca^{2+} dependent (Tamaskovic *et al*, 2003), we evaluated whether Ca^{2+} was necessary for lysosomal localization of STK38 even under lysosomal damage conditions. We found that lysosomal localization of mNG-STK38 was suppressed by pre-treatment with the Ca^{2+} chelator BAPTA-AM, suggesting that STK38 is specifically recruited to damaged lysosomes in a Ca^{2+}-dependent manner (Fig 1B and C).

Next, we asked which domain of STK38 is essential for its lysosomal localization. STK38 contains central kinase domain and N- and C-terminal regulatory domains. In addition, STK38 has three phosphorylation sites (T74, S281, and T444) whose phosphorylation is required for full kinase activity of STK38 (Tamaskovic *et al*, 2003). We constructed truncation mutants of regulatory domains (deltaN and deltaC) and phospho-dead mutants (T74A, S281A, and T444A) and observed localization of these mutants after LLOMe treatment (Fig 1D). Intriguingly, we found that deletion of C-terminal of STK38 (deltaC mutant) completely abolished its lysosomal localization after LLOMe treatment, while other mutants did

 © 2023 The Authors

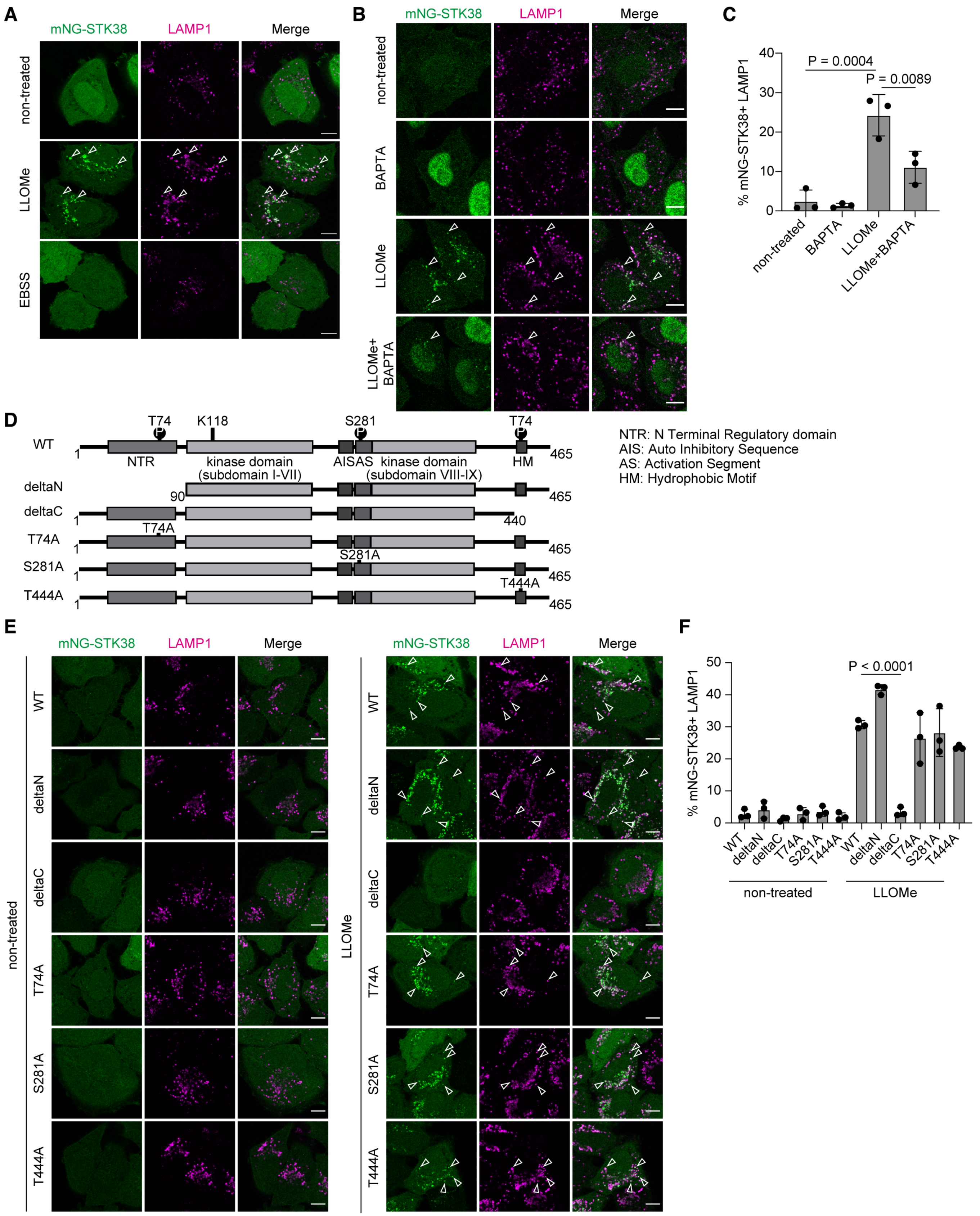
A
mNG-STK38
LAMP1
Merge
non-treated
LLOMe
EBSS
B
mNG-STK38
LAMP1
Merge
non-treated
BAPTA
LLOMe
LLOMe+ BAPTA
C
% mNG-STK38+ LAMP1
P = 0.0004
P = 0.0089
non-treated
BAPTA
LLOMe
LLOMe+BAPTA
D
WT
T74
K118
S281
T74
1
NTR
kinase domain (subdomain I-VII)
AISAS
kinase domain (subdomain VIII-IX)
HM
465
deltaN
90
465
deltaC
1
440
T74A
T74A
1
465
S281A
S281A
1
465
T444A
T444A
1
465
NTR: N Terminal Regulatory domain
AIS: Auto Inhibitory Sequence
AS: Activation Segment
HM: Hydrophobic Motif
E
mNG-STK38
LAMP1
Merge
non-treated
LLOMe
WT
deltaN
deltaC
T74A
S281A
T444A
F
% mNG-STK38+ LAMP1
P < 0.0001
WT
deltaN
deltaC
T74A
S281A
T444A
non-treated
LLOMe

Figure 1.

Figure 1. STK38 is recruited to lysosomes in response to lysosomal damage.

A Representative images of transiently expressed mNeonGreen (mNG)-STK38 (green) and immunostained LAMP1 (magenta) in HeLa cells treated with LLOMe (1 mM for 1 h, then incubated for 3 h after washout) or EBSS (for 4 h). Scale bars: 10 μm.
B Representative images of stably expressed mNG-STK38 (green) and immunostained LAMP1 (magenta) in HeLa cells treated with LLOMe (1 mM for 1 h) with or without pre-treatment with BAPTA-AM (10 μM for 1 h). Scale bars: 10 μm.
C Quantification of colocalization between mNG-STK38 and LAMP1 shown in (B). ≥ 50 cells were analyzed per experiment for each condition.
D Schematic diagram of WT and mutant STK38.
E Representative images of transiently expressed WT or mutant mNG-STK38 (green) and immunostained LAMP1 (magenta) in HeLa cells treated with LLOMe (1 mM for 1 h, then incubated for 3 h after washout). Scale bars: 10 μm.
F Quantification of colocalization between mNG-STK38 and LAMP1 shown in (E). ≥ 50 cells were analyzed per experiment for each condition.

Data information: All data presented as means ± SD, from $n \geq 3$ independent experiments. *P*-values were determined using one-way ANOVA with Tukey's multiple comparisons test (C) or Dunnett's multiple comparisons test (F). See also Fig EV1.
Source data are available online for this figure.

not show significant suppression of it (Fig 1E and F). The C-terminal region includes a hydrophobic motif, a common feature of AGC kinases, known to be involved in the structural change of STK38 to maximize its kinase activity (Gógl *et al*, 2015). These data suggest that the C-terminal of STK38, conceivably the hydrophobic motif, is necessary for STK38 lysosomal localization through the structural change.

STK38 is required for lysosomal membrane repair by ESCRT machinery

We next sought to determine how STK38 contributes to lysosomal damage responses. To ascertain if this occurs through regulation of macrolysophagy, we depleted STK38 and monitored the turnover of damaged lysosomes in macrolysophagy-deficient FIP200 knockout (KO) cells. We found that depletion of STK38 further delayed the clearance of GFP-Gal3, indicating that STK38 is essential for the clearance of damaged lysosomes in a macrolysophagy-independent manner (Fig 2A and B). Furthermore, we examined the localization of STK38 in several macrolysophagy-deficient cells, as follows: cells lacking FIP200, ATG14, ATG7, ATG9, or STX17; penta KO cells lacking five macroautophagy receptors (p62, TAX1BP1, OPTN, NBR1, and NDP52); and hexa KO cells lacking six human ATG8 paralogs (Lazarou *et al*, 2015; Nguyen *et al*, 2016). In all KO cells we tested, mNG-STK38 was recruited to lysosomes after LLOMe treatment, suggesting that STK38 localization occurred independently of macroautophagy machinery (Appendix Fig S2). Of note, although STK38 has been previously implicated in the regulation of macroautophagy (Joffre *et al*, 2015; Klimek *et al*, 2019; Martin *et al*, 2019), its depletion had no significant effect on basal or starvation-induced macroautophagy in our experimental conditions (Fig EV2A and B). In addition, the recruitment of ULK1 to lysosomes after LLOMe treatment in STK38 depleted cells was comparable to control cells, suggesting STK38 had no effect on induction of macrolysophagy (Fig EV2C and D). We next tested the contribution of STK38 to lysosomal damage responses other than macrolysophagy, specifically by assessing TFEB activation after lysosomal damage. Depletion of STK38 had no effect on the nuclear translocation of TFEB after LLOMe treatment, suggesting that this pathway is independent of STK38 (Fig EV2E and F).

Next, in order to investigate the relationship between STK38 and lysosomal repair performed by ESCRT machinery, we examined the recruitment of ESCRT components. Depletion of STK38 increased the numbers of CHMP4B and ALIX puncta after LLOMe treatment compared to control cells (Fig 2C–E). In control cells, the numbers of these puncta were decreased after 1-h LLOMe treatment, while they remained high in STK38-depleted cells. By contrast, the recruitment of VPS4 to lysosomes after LLOMe treatment was suppressed in STK38-depleted cells compared to control cells (Fig 2F and G). We also confirmed similar phenotypes in STK38 KO cells, although they were weaker than in knockdown cells probably due to the compensation of other NDR-kinases such as STK38L (Appendix Fig S3). Given that VPS4 plays a role in the disassembly of ESCRT components, these results suggest that STK38 regulates recruitment of VPS4 to damaged lysosomes and that STK38 depletion inhibits the disassembly of ESCRT components. Of note, the suppression of the lysosomal damage response in STK38-depleted cells was not due to deterioration of basal lysosomal integrity since STK38 depletion

Figure 2. STK38 is required for VPS4 recruitment to lysosomes.

A Representative images of stably expressed GFP-Gal3 (green) in siSTK38-transfected WT or FIP200 KO HeLa cells. Cells were treated with LLOMe (1 mM for 1 h, then incubated for the indicated number of hours after washout). Scale bars: 10 μm.
B Percentage of residual GFP-Gal3 dots (10 h/0 h [%]) in (A). ≥ 80 cells were analyzed per experiment for each condition.
C Representative images of immunostained CHMP4B (green), ALIX (magenta), and DAPI (blue) in siSTK38-transfected HeLa cells. Cells were treated with LLOMe (1 mM for 30 min or 1 h). Scale bars: 10 μm.
D Quantification of CHMP4B dots shown in (C). ≥ 100 cells were analyzed per experiment for each condition.
E Quantification of ALIX dots shown in (C). ≥ 100 cells were analyzed per experiment for each condition.
F Representative images of immunostained VPS4 (green), LAMP1 (magenta), and DAPI (blue) in siSTK38-transfected HeLa cells. Cells were treated with LLOMe (1 mM for 30 min or 1 h). Scale bars: 10 μm.
G Quantification of co-localization between VPS4 and LAMP1 shown in (F). ≥ 50 cells were analyzed per experiment for each condition.

Data information: All data presented as means ± SD, from $n \geq 3$ independent experiments. *P*-values were determined using one-way ANOVA with Tukey's multiple comparisons test. See also Fig EV2.
Source data are available online for this figure.

© 2023 The Authors

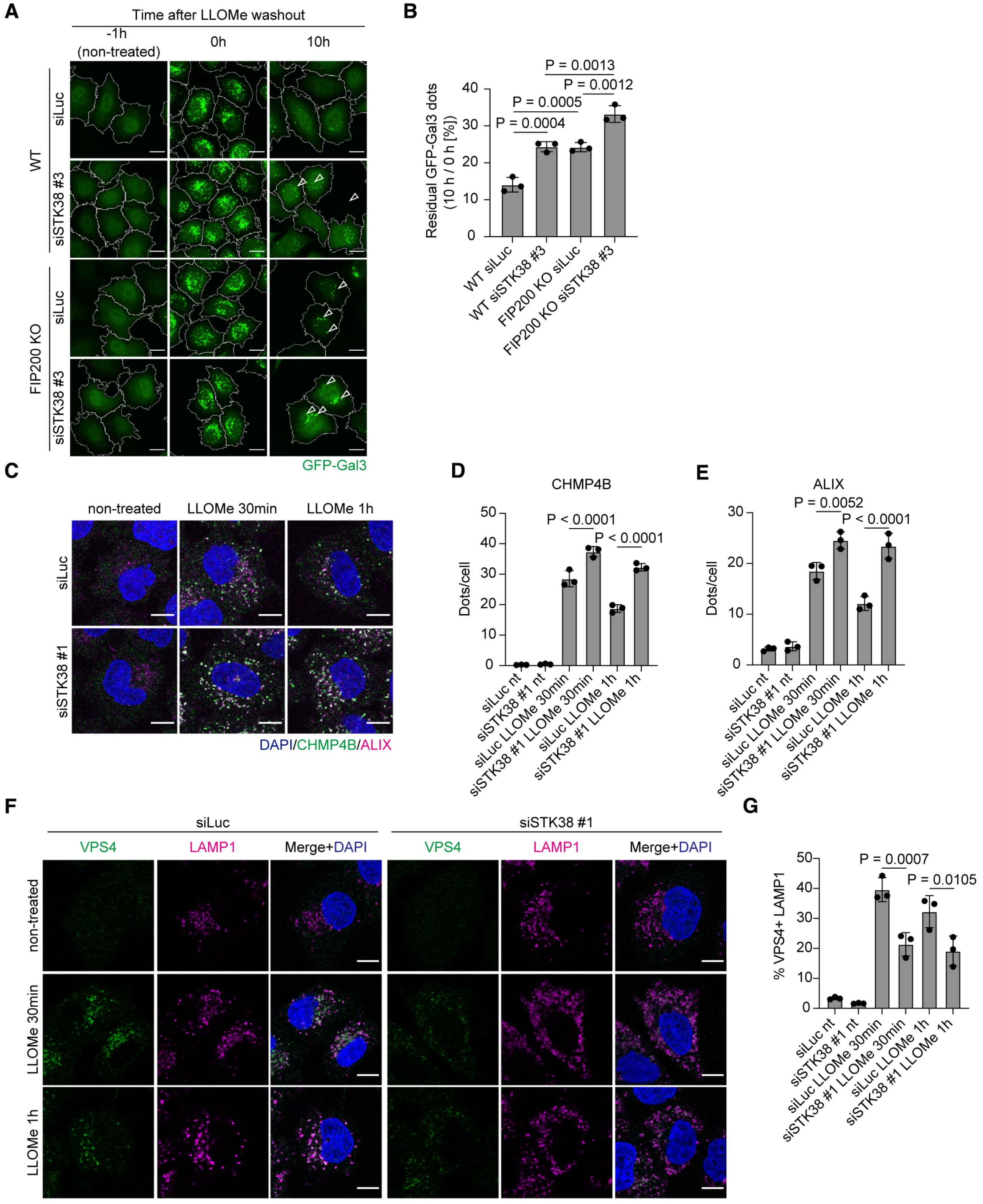

Figure 2.

did not suppress the number, acidity, or enzyme activity of lysosomes (Appendix Fig S4). ESCRT machinery is essential for the formation of multivesicular bodies (MVBs); thus, we tested the contribution of STK38 to degradation of epidermal growth factor receptor (EGFR), which is incorporated into MVBs for lysosomal degradation. We found that STK38 depletion had no effect on EGFR

degradation after epidermal growth factor (EGF) stimulation (Fig EV2G and H). Taken together, these findings suggest that STK38 is specifically required for the recruitment of ESCRT machinery to repair damaged lysosomes.

STK38 and ESCRTs are required for lysosomal repair by microlysophagy

Next, we sought to determine how STK38 and ESCRTs repair damaged lysosomes. In many types of microautophagy, ESCRT machinery plays essential roles in membrane invagination and scission (Schuck, 2020; Wang *et al*, 2022). Recent studies reported that lysosomal membrane proteins are selectively degraded by microautophagy during lysosomal stress conditions such as lysosomal damage and lysosomal osmotic stress (referred to as microlysophagy) (Lee *et al*, 2020). Based on these backgrounds, we hypothesized that the ESCRT-dependent lysosomal repair observed in previous studies (Radulovic *et al*, 2018; Skowyra *et al*, 2018) was mediated by microlysophagy. To test this hypothesis, we conducted an EGFP-TRPML1 cleavage assay that allowed us to preferentially assess microlysophagy activity by monitoring an increase in free EGFP cleaved from EGFP-TRPML1 after lysosomal stress induction (Figs 3A and EV3A and B) (Lee *et al*, 2020). As inducers of microlysophagy, we used ionophores (monensin and nigericin) or NH_4Cl which increase luminal pH of lysosomes and induce non-canonical lipidation of ATG8 to lysosomes (Jacquin *et al*, 2017, Fig EV3C). Different from LLOMe, these drugs did not induce severe lysosomal damage labeled by Gal3 and macrolysophagy as indicated by its markers p62 and ubiquitin (Maejima *et al*, 2013), enabling us to induce and assess microlysophagy specifically (Fig EV3C). Indeed, immunofluorescence-based imaging showed the presence of EGFP-TRPML1 positive Intraluminal vesicles (ILVs), indicative of microautophagy, in pharmacologically swollen LAMP1 or CD63 positive endolysosomes upon lysosomal stress (Fig EV3D and E). Consistent with our hypothesis, individual depletion of most ESCRT components, including TSG101, ALIX, CHMP4A/B, and VPS4A/B, resulted in decreased cleavage of EGFP-TRPML1 after nigericin treatment, indicating suppression of microlysophagy (Fig 3B and C, Appendix Fig S5). Depletion of VPS22 (ESCRT-II) had no effect on this cleavage, probably because ALIX can interact with ESCRT-III and therefore bypass the function of VPS22. These data suggest that ESCRT components are required for microlysophagy.

We further tested whether STK38 contributes to lysosomal homeostasis after lysosomal stress or damage via microlysophagy. An EGFP-TRPML1 cleavage assay showed that STK38 depletion suppressed cleavage after nigericin treatment both in WT and macroautophagy-deficient ATG13 KO cells (Fig 3D and E). Similar findings were observed under lysosomal damage conditions (Fig 3F and G). Consistent with the results of the cleavage assay, Transmission Electron Microscopy (TEM) images showed that NH_4Cl treatment led to a decreased number of ILVs in multivesicular structures in STK38-depleted cells (Fig 3H and I). Correlate light with electron microscopy (CLEM) analysis confirmed these ILVs colocalize with LAMP1-GFP signals after NH_4Cl treatment (Fig 3J). Taken together, ESCRT is essential for lysosomal repair through microlysophagy, and STK38 is required for this pathway by recruiting VPS4.

STK38 regulates microlysophagy in a kinase activity-dependent manner

To further understand the mechanism by which STK38 regulates microlysophagy, we first investigated the contribution of its kinase activity in this pathway. We expressed STK38 wild-type (WT), kinase dead mutant (K118R), truncation mutants, or phospho-dead mutants to STK38 knocked down cells, and observed VPS4 recruitment to lysosomes after LLOMe treatment. Among these STK38 constructs, only STK38 WT rescued VPS4 recruitment (Fig 4A–C). In addition to K118R mutant, truncation and phospho-dead mutants abolished the kinase activity of STK38 since they lack one of phosphorylation sites which are required for activation of STK38 (Tamaskovic *et al*, 2003). Given that, it is suggested that the kinase activity of STK38 is important for VPS4 recruitment. Furthermore, overexpression of STK38 WT increased cleaved EGFP, whereas K118R mutant did not (Fig EV4A and B), indicating that STK38 regulates microlysophagy in a kinase activity–dependent manner. Of note, we found that kinase-dead mutant of STK38 was recruited on lysosomes after LLOMe treatment, suggesting that the kinase activity of STK38 itself is dispensable for its localization (Fig EV4C).

We next sought to obtain the profile of STK38 phosphorylation targets essential for lysosomal microautophagy. For this purpose,

Figure 3. ESCRTs and STK38 are required for microlysophagy.

A Schematic diagram of EGFP-TRPML1 cleavage assay.
B Representative immunoblots of EGFP-TRPML1 in MCF10A cells transfected with siRNAs of ESCRT components. Cells were treated with nigericin (50 μM for 8 h).
C Quantification of cleaved EGFP shown in (B).
D Representative immunoblots of EGFP-TRPML1 in siSTK38-transfected WT or ATG13 KO MCF10A cells. Cells were treated with nigericin (50 μM for 8 h). The asterisk in STK38 blot represents non-specific band.
E Quantification of cleaved EGFP shown in (D).
F Representative immunoblots of EGFP-TRPML1 in siSTK38-transfected MCF10A cells. Cells were treated with LLOMe (0.5 mM for 1 h, then incubated for 7 h after washout).
G Quantification of cleaved EGFP shown in (F).
H Representative images of multivesicular structures (arrows) obtained by transmission electron microscopy (TEM). siSTK38-transfected MCF10A cells were treated with NH_4Cl (5 mM for 24 h). Scale bars: 1 μm.
I Quantification of ILVs per multivesicular structure shown in (H). ≥ 20 multivesicular structures from 10 independent cells were analyzed per condition.
J Representative images of CLEM analysis of LAMP1-GFP positive endolysosomes. LAMP1-GFP expressing MCF10A cells were treated with NH_4Cl (5 mM for 24 h). Scale bars: 10 μm (low magnification images) or 1 μm (magnified images).

Data information: Data presented as means ± SD, from $n \geq 3$ independent experiments (C, E, and G). *P*-values were determined using one-way ANOVA with Dunnett's multiple comparisons test (C and G) or Tukey's multiple comparisons test (E and I). See also Fig EV3.
Source data are available online for this figure.

© 2023 The Authors

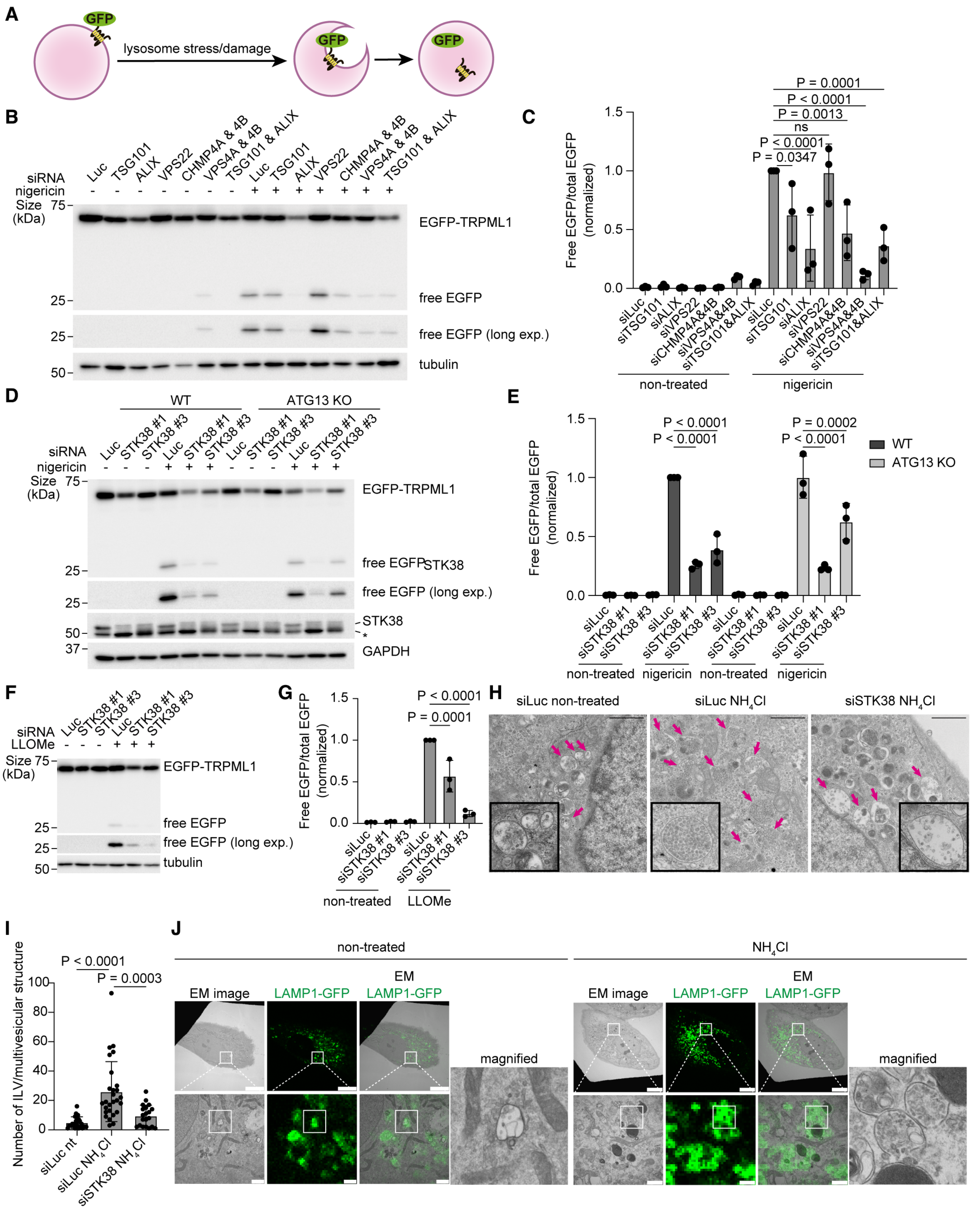
A
GFP
lysosome stress/damage
B
siRNA
nigericin
Size (kDa)
Luc TSG101 ALIX VPS22 CHMP4A & 4B VPS4A & 4B TSG101 & ALIX
EGFP-TRPML1
free EGFP
free EGFP (long exp.)
tubulin
C
Free EGFP/total EGFP (normalized)
P = 0.0001
P < 0.0001
P = 0.0013
ns
P < 0.0001
P = 0.0347
non-treated
nigericin
D
WT
ATG13 KO
STK38 #1
STK38 #3
STK38
GAPDH
E
P < 0.0001
P = 0.0002
ATG13 KO
F
LLOMe
G
H
siLuc non-treated
siLuc NH4Cl
siSTK38 NH4Cl
I
Number of ILV/multivesicular structure
P = 0.0003
J
EM image
LAMP1-GFP
EM LAMP1-GFP
magnified
NH4Cl

Figure 3.

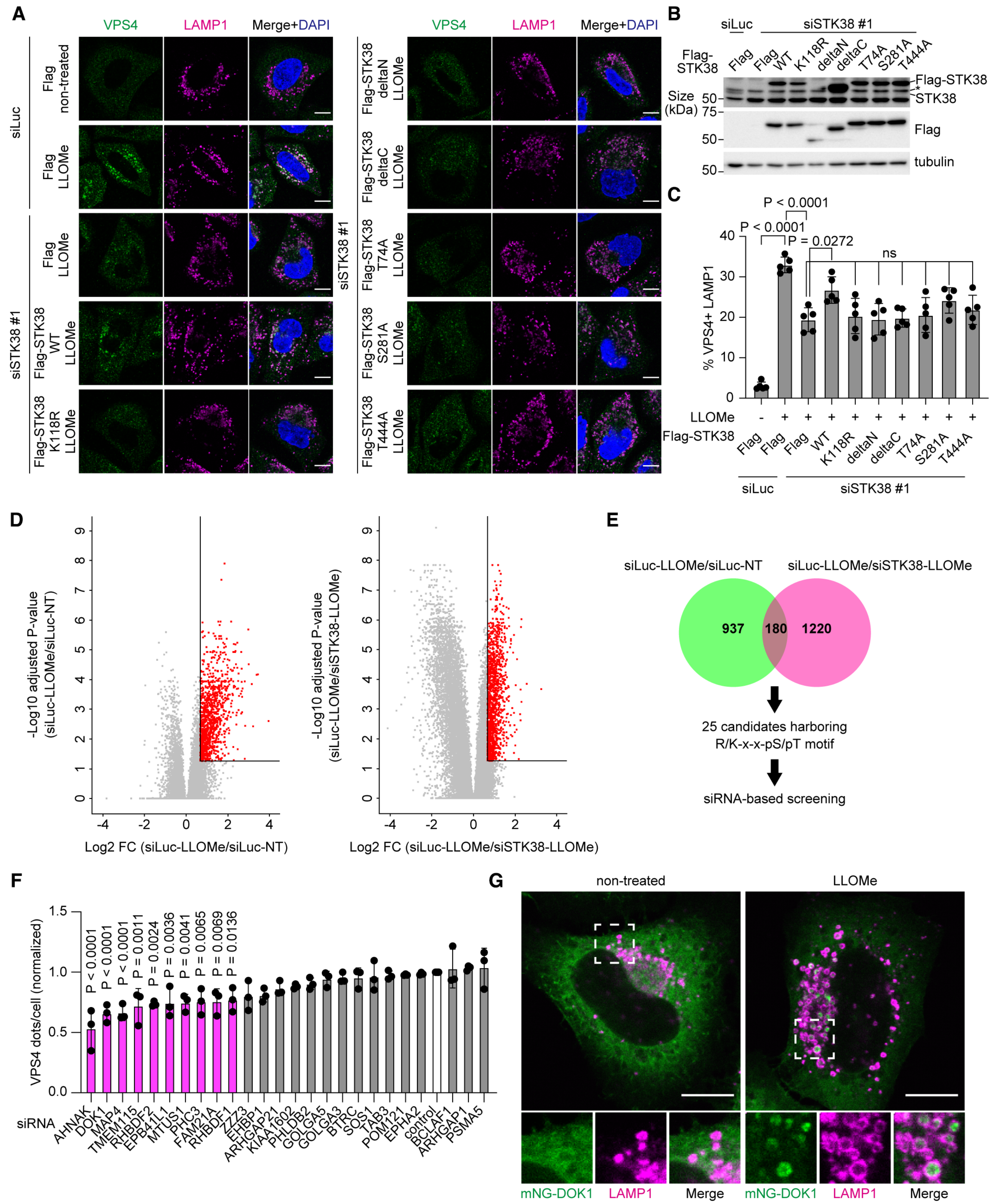

Figure 4.

 © 2023 The Authors

Figure 4. Quantitative phosphorylation reveals STK38 targets under lysosomal damage conditions.

A Representative images of immunostained VPS4 (green), LAMP1 (magenta), and DAPI (blue) in STK38 reconstituted HeLa cells. Cells were transfected with siSTK38 for 48 h, then transfected with Flag-tagged STK38. After 24 h of plasmid transfection, cells were treated with LLOMe (1 mM for 30 min). Scale bars: 10 μm.
B Representative immunoblots of STK38 in Flag-STK38 reconstituted HeLa cells in (A). The asterisk in STK38 blot represents STK38L.
C Quantification of co-localization between VPS4 and LAMP1 shown in (A). ≥ 50 cells were analyzed per experiment for each condition.
D Volcano plots of tandem mass tag phosphoproteomics data. Phosphopeptides from siLuciferace (Luc)-transfected HeLa cells treated with LLOMe (siLuc-LLOMe) were compared with siLuc-transfected non-treated cells (siLuc-NT) (left) or with siSTK38-transfected cells treated with LLOMe (siSTK38-LLOMe) (right). The horizontal line indicates the significance cutoff (adjusted *P*-value < 0.05). The vertical line indicates the fold-change cutoff (FC ≥ 1.6). Red dots indicate phosphopeptides above the significance and fold-change thresholds. The data was obtained from three independent experiments.
E Schematic diagram of candidate selection based on phosphoproteomics results. The Venn diagram represents the number of phosphopeptides above the thresholds from the two comparisons shown in (D). From 180 phosphopeptides, we selected 25 candidates that shared a common AGC kinase phosphorylation motif (R/K-x-x-pS/pT).
F Quantification of VPS4 dots in HeLa cells transfected with siRNAs of 25 candidates. Cells were treated with LLOMe (1 mM for 30 min). ≥ 70 cells were analyzed per experiment for each condition.
G Representative images of transiently expressed mNG-DOK1 (green) and immunostained LAMP1 (magenta) in HeLa cells treated with LLOMe (1 mM for 1 h, then incubated for 3 h after washout). Scale bars: 10 μm.

Data information: All data presented as means ± SD, from *n* ≥ 3 independent experiments. *P*-values were determined using one-way ANOVA with Tukey's multiple comparisons test (C) or Dunnett's multiple comparisons test (F). See also Fig EV4.
Source data are available online for this figure.

we conducted tandem mass tag (TMT)-based quantitative phosphoproteomics to compare control and STK38-depleted cells treated with or without LLOMe. Among peptides that were phosphorylated after LLOMe treatment and whose phosphorylation was suppressed in STK38-depleted cells, we selected 25 candidates with a common AGC kinase phosphorylation motif (R/K-x-x-pS/pT) (Fig 4D and E). We evaluated the contribution of these candidates to VPS4 recruitment after LLOMe treatment. Indeed, the depletion of 10 candidates significantly suppressed VPS4 recruitment after LLOMe treatment (Fig 4F). We examined the subcellular localization of these candidates (excluding RHBDF2) and found that mNG tagged DOK1, which function as an adapter protein downstream of several signaling pathways (Carpino *et al*, 1997; Yamanashi & Baltimore, 1997; Mashima *et al*, 2009; Brummer *et al*, 2010), was exclusively localized on lysosomes upon lysosomal damage (Fig 4G, Appendix Fig S6). Of note, DOK1 was localized inside of LAMP1 positive structure, which was very similar to that of STK38, further implying they work together (Figs 4G and EV1D).

Phosphorylation of DOK1 S269 is required for its lysosomal localization and subsequent VPS4 recruitment to lysosomes

From TMT-based quantitative phosphoproteomics, S269 of DOK1 was identified as a putative phosphorylation site mediated by STK38. To confirm this, we quantified this phosphorylation site by parallel reaction monitoring (PRM) mass spectrometry. This analysis revealed that phosphorylation of DOK1 on S269 was increased after LLOMe treatment, and it was suppressed in STK38 depleted cells, supporting our phosphoproteomics analysis (Fig 5A and B).

To evaluate the functional importance of STK38-mediated phosphorylation of DOK1 on S269 during lysosomal damage condition, we expressed DOK1 WT or phospho-dead S269A mutant to DOK1 depleted cells. Expression of DOK1 WT but not S269A rescued lysosomal localization of VPS4, suggesting that this phosphorylation is required for VPS4 recruitment to lysosomes (Figs 5C and D, and EV4D). Next, we tested whether STK38-mediated phosphorylation of DOK1 affected its localization. Lysosomal localization of mNG-DOK1 after LLOMe treatment was significantly suppressed in STK38 KD cells (Fig 5E and F). Furthermore, phospho-dead mutation on S269 suppressed co-localization between DOK1 and LAMP1 after LLOMe treatment, suggesting importance of phosphorylation of this site for lysosomal localization (Fig 5G and H). We also investigated whether DOK1 interacts with STK38 and VPS4. Co-immunoprecipitation showed that Flag-DOK1 interacted with HA-STK38 and VPS4 (Fig EV4E). Of note, this interaction was detected without LLOMe, probably due to overexpression of Flag-DOK1. In conclusion, it is suggested that STK38 recruits DOK1 to lysosomes via phosphorylation

Figure 5. DOK1 phosphorylated by STK38 is required for VPS4 recruitment to lysosomes.

A Phosphorylation of DOK1 S269 was demonstrated by the MS/MS spectrum of the *m/z* 561.25214 ion from siLuc-transfected and LLOMe-treated cells.
B PRM-based quantification of the phosphopeptide shown in (A). HeLa cells stably expressing mNG-DOK1 were transfected with siLuc or siSTK38. Then, cells were treated with LLOMe (1 mM) for (i) 30 min or (ii) 1 h plus incubation for 5 h after washout (1 h + wo 5 h).
C Representative images of immunostained VPS4 (green), LAMP1 (magenta), and DAPI (blue) in DOK1 reconstituted HeLa cells. Cells were transfected with siDOK1 for 48 h, then transfected with Flag-tagged DOK1. After 24 h of plasmid transfection, cells were treated with LLOMe (1 mM for 30 min). Scale bars: 10 μm.
D Quantification of co-localization between VPS4 and LAMP1 shown in (C). ≥ 50 cells were analyzed per experiment for each condition.
E Representative images of stably expressed mNG-DOK1 (green), LAMP1 (magenta), and DAPI (blue) in siSTK38-transfected HeLa cells. Cells were treated with LLOMe (1 mM for 1 h). Scale bars: 10 μm.
F Quantification of co-localization between mNG-DOK1 and LAMP1 shown in (E). ≥ 50 cells were analyzed per experiment for each condition.
G Representative images of transiently expressed WT or S269A mNG-DOK1 (green), LAMP1 (magenta), and DAPI (blue) in HeLa cells. Cells were treated with LLOMe (1 mM for 1 h). Scale bars: 10 μm.
H Quantification of co-localization between mNG-DOK1 and LAMP1 shown in (G). ≥ 50 cells were analyzed per experiment for each condition.

Data information: Data presented as means ± SD, from *n* ≥ 3 independent experiments (D, F and H). *P*-values were determined using one-way ANOVA with Tukey's multiple comparisons test. See also Fig EV4.
Source data are available online for this figure.

© 2023 The Authors

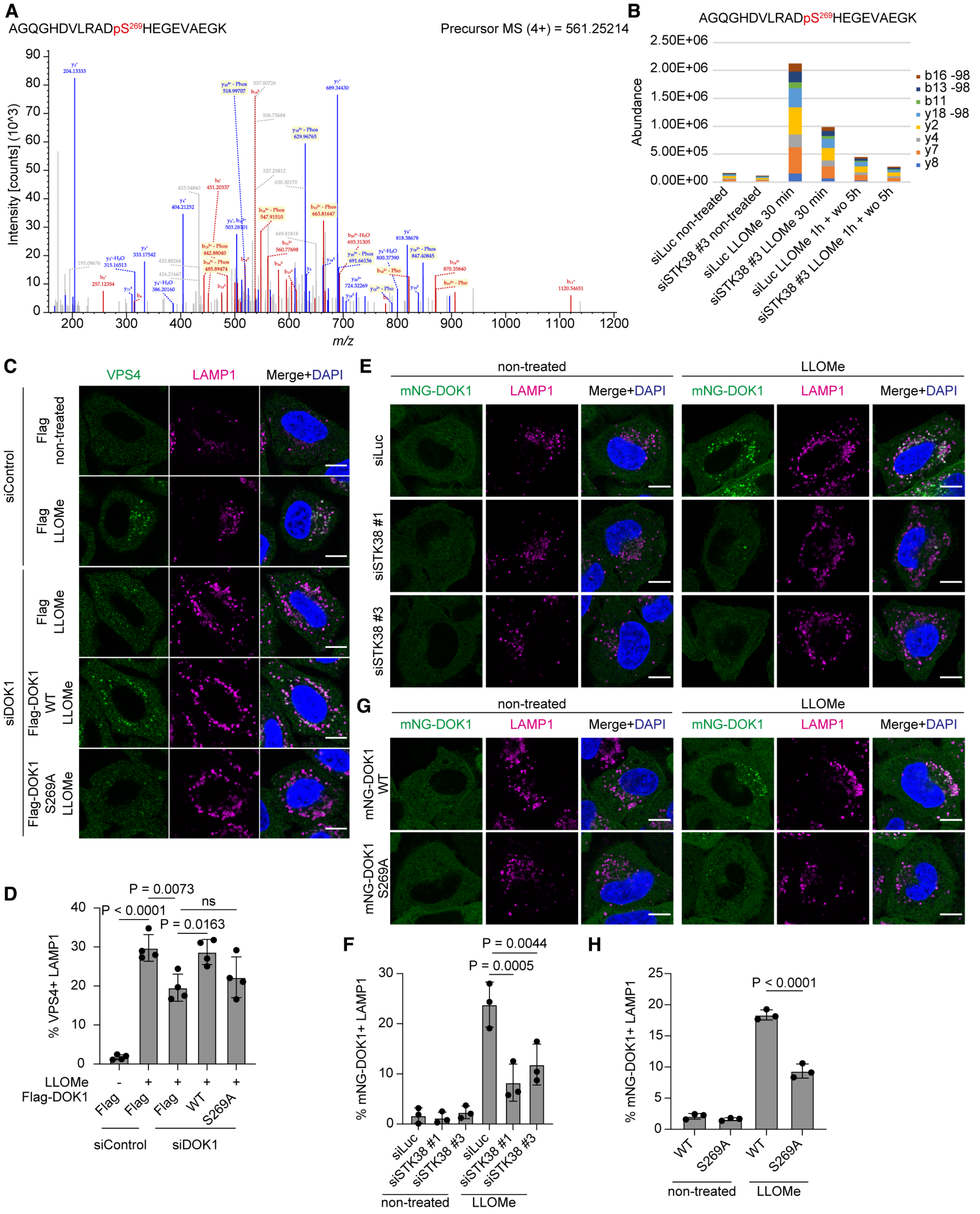

Figure 5.

© 2023 The Authors

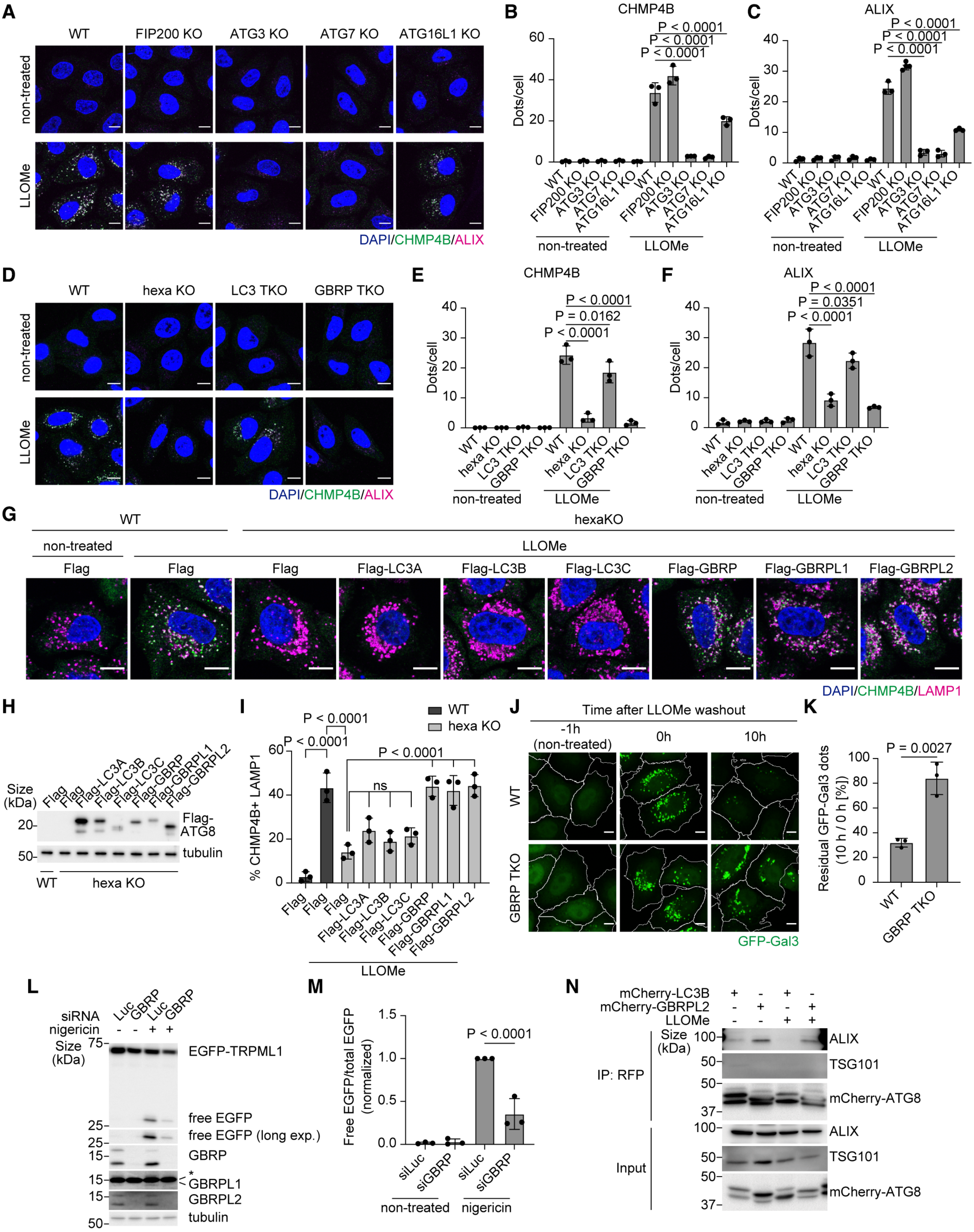

A
WT
FIP200 KO
ATG3 KO
ATG7 KO
ATG16L1 KO
non-treated
LLOMe
DAPI/CHMP4B/ALIX
B
CHMP4B
Dots/cell
P < 0.0001
P < 0.0001
P < 0.0001
non-treated
LLOMe
C
ALIX
D
WT
hexa KO
LC3 TKO
GBRP TKO
DAPI/CHMP4B/ALIX
E
CHMP4B
P < 0.0001
P = 0.0162
P < 0.0001
F
ALIX
P < 0.0001
P = 0.0351
P < 0.0001
G
WT
hexaKO
non-treated
LLOMe
Flag
Flag-LC3A
Flag-LC3B
Flag-LC3C
Flag-GBRP
Flag-GBRPL1
Flag-GBRPL2
DAPI/CHMP4B/LAMP1
H
Size (kDa)
Flag-ATG8
tubulin
WT
hexa KO
I
% CHMP4B+ LAMP1
WT
hexa KO
P < 0.0001
P < 0.0001
P < 0.0001
ns
LLOMe
J
Time after LLOMe washout
-1h (non-treated)
0h
10h
WT
GBRP TKO
GFP-Gal3
K
Residual GFP-Gal3 dots (10 h / 0 h [%])
P = 0.0027
WT
GBRP TKO
L
siRNA
Luc
GBRP
nigericin
Size (kDa)
EGFP-TRPML1
free EGFP
free EGFP (long exp.)
GBRP
GBRPL1
GBRPL2
tubulin
M
Free EGFP/total EGFP (normalized)
P < 0.0001
siLuc
siGBRP
non-treated
nigericin
N
mCherry-LC3B
mCherry-GBRPL2
LLOMe
IP: RFP
Input
Size (kDa)
ALIX
TSG101
mCherry-ATG8

Figure 6.

Figure 6. GABARAPs are required for ESCRT recruitment to damaged lysosomes.

A Representative images of immunostained CHMP4B (green), ALIX (magenta), and DAPI (blue) in WT, FIP200, ATG3, ATG7, and ATG16L1 KO HeLa cells. Cells were treated with LLOMe (1 mM for 30 min). Scale bars: 10 μm.
B Quantification of CHMP4B dots shown in (A). ≥ 100 cells were analyzed per experiment for each condition.
C Quantification of ALIX dots shown in (A). ≥ 100 cells were analyzed per experiment for each condition.
D Representative images of immunostained CHMP4B (green), ALIX (magenta), and DAPI (blue) in WT, ATG8 hexa KO, LC3 TKO, and GABARAP (GBRP) TKO HeLa cells. Cells were treated with LLOMe (1 mM for 30 min). Scale bars: 10 μm.
E Quantification of CHMP4B dots shown in (D). ≥ 100 cells were analyzed per experiment for each condition.
F Quantification of ALIX dots shown in (D). ≥ 100 cells were analyzed per experiment for each condition.
G Representative images of immunostained CHMP4B (green), LAMP1 (magenta), and DAPI (blue) in WT and ATG8 hexa KO HeLa cells expressing 3×Flag-ATG8 paralogs. Cells were treated with LLOMe (1 mM for 30 min). Scale bars: 10 μm.
H Representative immunoblots of Flag-ATG8s expressed in HeLa cells.
I Quantification of co-localization between CHMP4B and LAMP1 shown in (G). ≥ 100 cells were analyzed per experiment for each condition.
J Representative images of stably expressed GFP-Gal3 (green) in WT or GABARAP TKO HeLa cells. Cells were treated with LLOMe (1 mM for 1 h, then incubated for the indicated number of hours after washout). Scale bars: 10 μm.
K Percentage of residual GFP-Gal3 dots (10 h/0 h [%]) in (J). ≥ 100 cells were analyzed per experiment for each condition.
L Representative immunoblots of EGFP-TRPML1 and GABARAPs in MCF10A cells transfected with siGABARAP (siRNAs of GABARAP, GABARAPL1, and GABARAPL2). Cells were treated with nigericin (50 μM for 8 h). The asterisk in GABARAPL1 blot represents a non-specific band.
M Quantification of cleaved EGFP shown in (L).
N Representative immunoblots from a co-immunoprecipitation experiment. mCherry-LC3B- or GABARAPL2-expressing hexa KO HeLa cells were treated with LLOMe (1 mM for 30 min). Cell lysates were immunoprecipitated using RFP-trap.

Data information: All data presented as means ± SD, from $n \geq 3$ independent experiments. *P*-values were determined using one-way ANOVA with Tukey's multiple comparisons test (B, C, E, F, and I), Dunnett's multiple comparisons test (M), or the unpaired *t*-test (K). See also Fig EV5.
Source data are available online for this figure.

on S269. It is plausible that they form ternary complex with VPS4 and regulate VPS4 recruitment to lysosomes.

Non-canonical lipidation of GABARAPs is essential for the recruitment of ESCRT components to damaged lysosomes

Our data revealed that STK38 is required for the recruitment of VPS4, but how are other core ESCRT components recruited to damaged lysosomes? A previous report suggested that ATG8 lipidation, which is mediated by ATG conjugation machinery consisting of a series of enzymes such as ATG7, ATG3, and ATG16L1, is required for microlysophagy in macroautophagy-independent manner (Lee *et al*, 2020). Consistent with this report, we confirmed that microlysophagy was suppressed in ATG8 lipidation–deficient ATG5 KO cells but not in ATG13 KO cells (Fig EV3A and B). However, the role of non-canonical lipidated ATG8s in microlysophagy is unclear. Strikingly, we found that recruitment of ALIX, CHMP4B, and VPS4 to damaged lysosomes was completely suppressed in ATG8 lipidation–deficient ATG7 KO cells (Figs 6A–C and EV5A and B). This phenomenon was also seen in other conjugation machinery–depleted cells (ATG3 KO and ATG16L1 KO cells), but not in FIP200 KO cells (Figs 6A–C and EV5A and B), suggesting that non-canonical lipidation of ATG8s is required for recruitment of ESCRT components to damaged lysosomes. Of note, LLOMe treatment led to a decreased number of ESCRT puncta in ATG16L1 KO cells, although to a lesser extent than in ATG3 or ATG7 KO cells, probably due to redundant contributions by ATG16L2 or TECPR1, a recently reported novel E3-like protein required for non-canonical ATG8 lipidation (Boyle *et al*, 2023; Corkery *et al*, 2023; Kaur *et al*, 2023).

We next tested the contribution of ATG8s to ESCRT recruitment to damaged lysosomes. In ATG8 hexa KO cells, ESCRTs did not form puncta, suggesting that ATG8s are necessary for their recruitment (Figs 6D–F and EV5C and D). Recent studies revealed that human ATG8 paralogs, which are classified into the LC3 subfamily (LC3A, LC3B, and LC3C) and the GABARAP subfamily (GABARAP, GABARAPL1, and GABARAPL2), have different functions depending on the context (Weidberg *et al*, 2010; Goodwin *et al*, 2021). We thus used LC3 TKO, and GABARAP TKO cells to determine if there is any preferential function of these subfamilies for recruiting ESCRT machinery. ESCRT recruitment was suppressed in both LC3 TKO and GABARAP TKO cells, but the effect was more severe in the latter than in the former (Figs 6D–F and EV5C and D). In addition, among six paralogs, the expression of GABARAPs rescued CHMP4B recruitment in hexa KO cells, while LC3s did not (Fig 6G–I). Furthermore, in GABARAP TKO/TKD cells, both the clearance of GFP-Gal3 and the cleavage of EGFP-TRPML1 was impaired (Fig 6J–M). Taken together, our data suggest that non-canonical lipidation of ATG8s, mainly GABARAPs, is required for ESCRT recruitment to damaged lysosomes and following lysosomal damage response.

To further clarify the role of GABARAPs in ESCRT recruitment, we compared the interaction between GABARAPs and ESCRT components, specifically TSG101 and ALIX, which play a necessary early role in the assembly of ESCRT components. Importantly, immunoprecipitation showed that ALIX interacted preferentially with GABARAPL2 compared to LC3B (Figs 6N and EV5E), although the interaction was observed with or without LLOMe probably due to the effect of overexpression of LC3s or GABARAPs. Of note, TSG101 was co-immunoprecipitated with neither GABARAPL2 nor LC3B (Fig 6N). Thus, we concluded that non-canonical lipidated GABARAPs regulate ESCRT recruitment to damaged lysosomes by interacting with ALIX.

STK38 and GABARAPs have evolutionally conserved roles in the prevention of cellular senescence and organismal aging

Recent studies have shown that increased lysosomal damage is well correlated with organismal aging and cellular senescence (Li *et al*, 2016; Johmura *et al*, 2021), but it remains unknown if this is due to an impaired lysosomal damage response, and if failure of this response indeed accelerates aging. Using a doxorubicin (DXR)-

 © 2023 The Authors

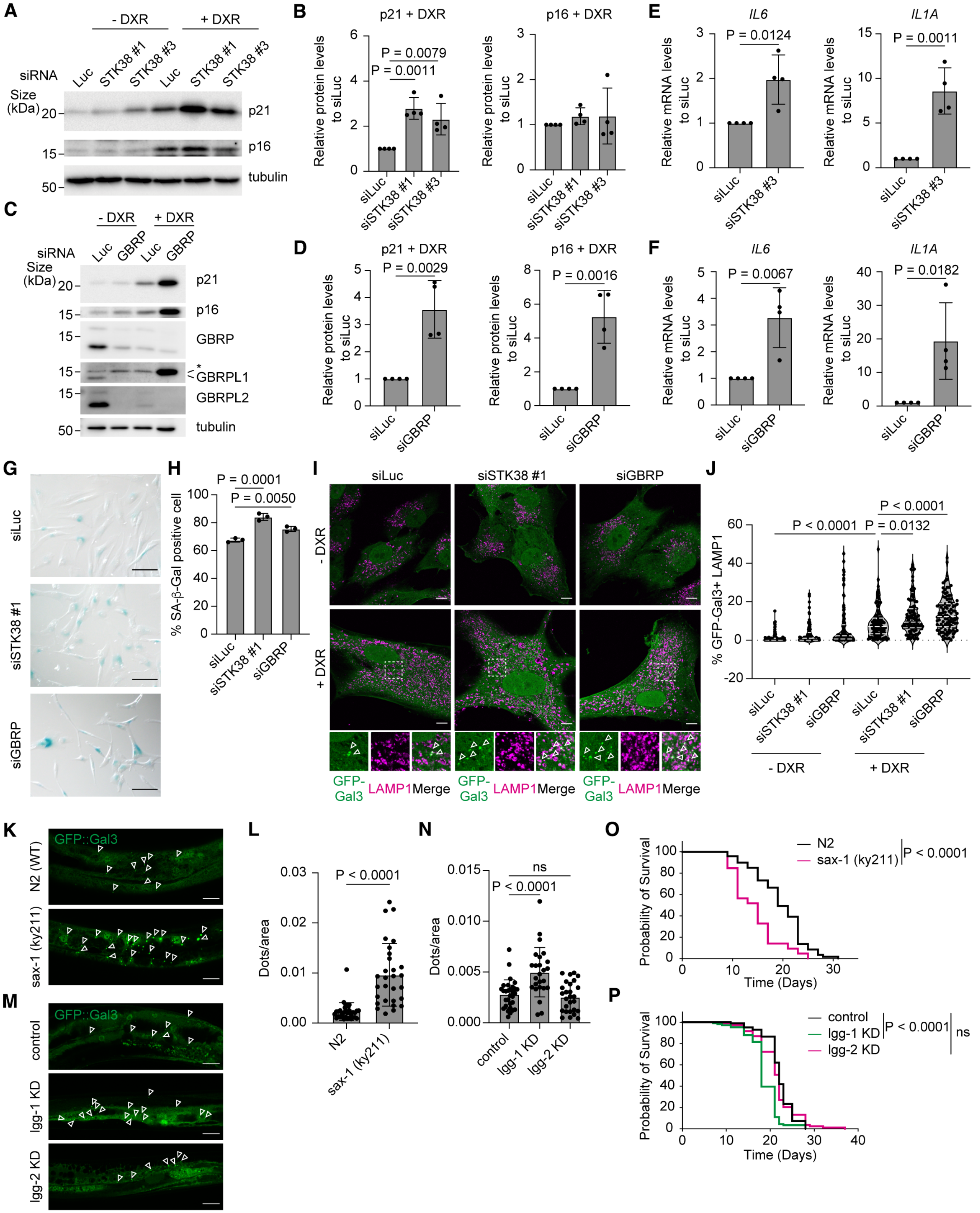

A
- DXR
+ DXR
siRNA
Luc
STK38 #1
STK38 #3
Size (kDa)
p21
p16
tubulin
B
p21 + DXR
p16 + DXR
Relative protein levels to siLuc
P = 0.0079
P = 0.0011
siLuc
siSTK38 #1
siSTK38 #3
C
GBRP
GBRPL1
GBRPL2
D
P = 0.0029
P = 0.0016
siGBRP
E
IL6
IL1A
Relative mRNA levels to siLuc
P = 0.0124
P = 0.0011
F
P = 0.0067
P = 0.0182
G
H
% SA-β-Gal positive cell
P = 0.0001
P = 0.0050
I
GFP-Gal3
LAMP1
Merge
J
% GFP-Gal3+ LAMP1
P < 0.0001
P = 0.0132
K
GFP::Gal3
N2 (WT)
sax-1 (ky211)
L
Dots/area
P < 0.0001
M
control
lgg-1 KD
lgg-2 KD
N
ns
O
N2
Probability of Survival
Time (Days)
P

Figure 7.

Figure 7. STK38 and GABARAPs have conserved roles in the prevention of cellular senescence and organismal aging.

A Representative immunoblots of p21 and p16 in siSTK38-transfected senescent (+ DXR) or non-induced (− DXR) RPE1 cells. Cellular senescence was induced by doxorubicin for 3 days.
B Quantification of p21 and p16 in senescent induced cells shown in (A).
C Representative immunoblots of p21 and p16 in siGABARAP-transfected senescent (+ DXR) or non-induced (− DXR) RPE1 cells. Cellular senescence was induced by doxorubicin for 3 days.
D Quantification of p21 and p16 in senescent induced cells shown in (C).
E Relative expression levels of *IL6* and *IL1A* mRNA in siSTK38-transfected senescent RPE1 cells. Cellular senescence was induced by doxorubicin for 3 days.
F Relative expression levels of *IL6* and *IL1A* mRNA in siGABARAP-transfected senescent RPE1 cells. Cellular senescence was induced by doxorubicin for 3 days.
G Representative images of SA-β-Gal–positive cells in siSTK38- or siGABARAP-transfected senescent RPE1 cells. Cellular senescence was induced by doxorubicin for 3 days. Scale bars: 100 μm.
H Percentage of SA-β-Gal–positive cells in (G). ≥ 500 cells were analyzed per experiment for each condition.
I Representative images of stably expressed GFP-Gal3 (green) and immunostainied LAMP1 (magenta) in siSTK38- or siGABARAP-transfected senescent RPE1 cells. Cellular senescence was induced by doxorubicin for 10 days. Scale bars: 10 μm.
J Quantification of co-localization between GFP-Gal3 and LAMP1 shown in (I). ≥ 100 cells were analyzed for each condition from 5 independent experiments.
K Representative images of *sax-1* KO *C. elegans* expressing GFP-Gal3 at day 7 of the adult stage. Scale bars: 20 μm.
L Quantification of GFP-Gal3 dots shown in (K). ≥ 29 worms were analyzed for each condition from 3 independent experiments.
M Representative images of *lgg-1* or *lgg-2* knocked down (KD) *C. elegans* expressing GFP-Gal3 at day 7 of the adult stage. Scale bars: 20 μm.
N Quantification of GFP-Gal3 dots shown in (M). ≥ 29 worms were analyzed for each condition from 3 independent experiments.
O Lifespan curves of N2 (WT) and *sax-1* KO worms.
P Lifespan curves of N2 (WT), *lgg-1* KD, and *lgg-2* KD worms.

Data information: All data presented as means ± SD, from $n \geq 3$ independent experiments. *P*-values were determined using one-way ANOVA with Dunnett's multiple comparisons test (B, H, and N), Tukey's multiple comparisons test (J), the unpaired *t*-test (D–F and L), or the log-rank test (O and P).
Source data are available online for this figure.

induced cellular senescence model in human RPE1 cells, we found that knockdown of STK38 and GABARAPs increased the protein levels of senescence markers p21 and/or p16 (Fig 7A–D, Appendix Fig S7), the gene expression of the senescence-associated secretory phenotype (SASP) factors IL-6 and IL-1α (Fig 7E and F), and the numbers of SA-β-Gal positive cells (Fig 7G and H), suggesting that STK38 and GABARAPs prevent cellular senescence. Consistent with previous report, Gal3 positive damaged lysosomes were increased in senescent cells, and they were further increased in STK38 or GABARAPs depleted senescent cells (Fig 7I and J). Moreover, in *C. elegans*, we found that damaged lysosomes labeled by Gal3::GFP were increased in aged worms depleted for *sax-1* (a *C. elegans* homolog of STK38) and *lgg-1* (a homolog of GABARAPs in mammals) but not *lgg-2* (a homolog of LC3s in mammals) indicating that *sax-1* and *lgg-1* are critical to maintain lysosomal homeostasis during normal aging (Fig 7K–N). Furthermore, the inhibition of *sax-1* and *lgg-1* but not *lgg-2* curtailed lifespan (Fig 7O and P). Taken together, these results suggest that STK38 and GABARAPs have evolutionally conserved roles to prevent aging, presumably by maintaining lysosomal homeostasis through microlysophagy.

Discussion

In this study, we showed that STK38 and GABARAPs are key regulators of lysosomal membrane repair, which is necessary to maintain lysosomal integrity through ESCRT-mediated microlysophagy. As shown in Fig 8, GABARAPs and STK38 are involved in the first and last steps of microlysophagy, respectively. GABARAPs are required for ESCRT assembly, specifically the recruitment of early-acting ESCRTs (ESCRT-I and ALIX) as a result of interaction with ALIX. On the other hand, STK38 and its phosphorylation target DOK1 are required for recruitment of VPS4, which disassembles ESCRTs in the last step of membrane invagination. Intriguingly, our data also suggest that STK38 and GABARAPs preserve lysosomal integrity and are essential to prevent aging.

Membrane repair occurs soon after lysosomal damage and can seal lysosomes that have subtle membrane damage and prevent severe lysosomal permeabilization followed by cell death. The ESCRT machinery plays a pivotal role in the repair pathway (Radulovic *et al*, 2018; Skowyra *et al*, 2018), but the exact mechanism was previously unclear. Our study demonstrates that ESCRTs drive microautophagy to remove the damaged portion of the lysosomal membrane, indicating that lysosomal membrane repair by ESCRT is identical to microlysophagy, a selective degradation of lysosomal membrane protein in response to lysosomal stress (Lee *et al*, 2020).

There are three types of autophagy in eukaryotic cells: macroautophagy, chaperone-mediated autophagy (CMA), and microautophagy. However, unlike the first two types, very little is known about the regulatory mechanism of microautophagy, especially in multicellular organisms. Nevertheless, previous studies have revealed that like macroautophagy, microautophagy can target specific cargoes such as lysosomes (microlysophagy), the endoplasmic reticulum (microERphagy), mitochondria (micromitophagy), lipid droplets (microlipophagy), and proteins (Sahu *et al*, 2011; Lemasters, 2014; Hammerling *et al*, 2017; Mejlvang *et al*, 2018; Omari *et al*, 2018; Loi *et al*, 2019; Lee *et al*, 2020; Schulze *et al*, 2020; Zhang *et al*, 2021). In mammals, most types of microautophagy reported thus far involve fission (incorporating cargoes by invagination of the lysosomal membrane) and require ESCRTs (Wang *et al*, 2022). Some selective forms of microautophagy require ATG8 lipidation (Mejlvang *et al*, 2018; Loi *et al*, 2019), although the function of lipidated ATG8s and its connection to ESCRTs are poorly understood. Our identification of microlysophagy that requires both ESCRTs and ATG8s will further the understanding of microautophagy overall.

Recent studies reported emerging evidence of non-canonical ATG8 function in diverse cellular processes such as LC3-associated phagocytosis, activation of TFEB in response to lysosomal damage,

© 2023 The Authors

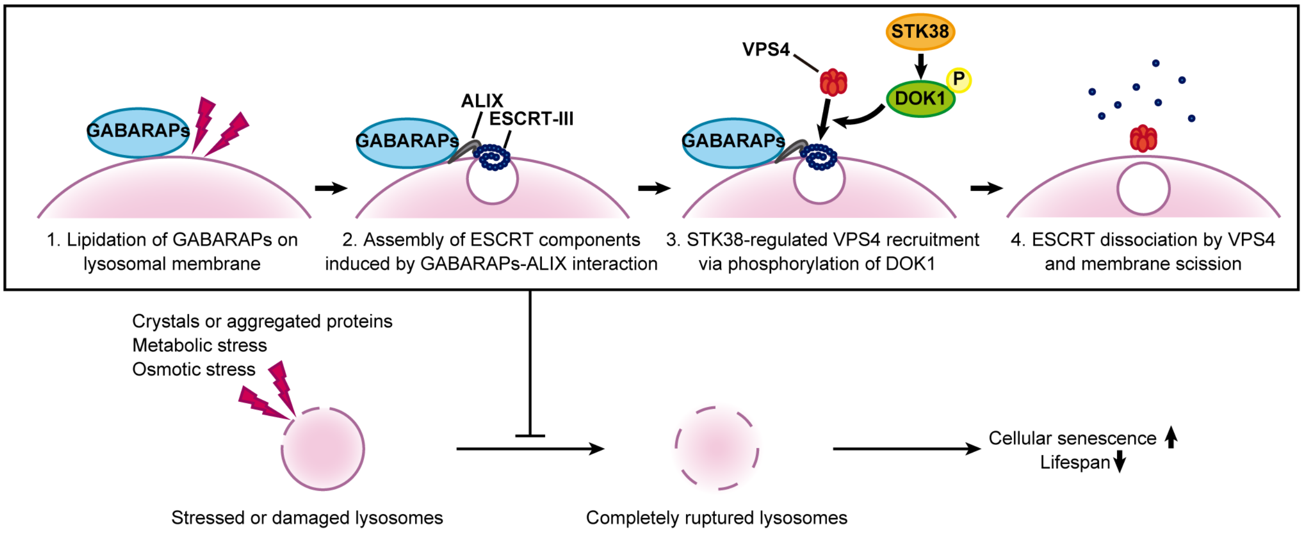

Figure 8. Working model of microlysophagy.

Schematic of a current model of the regulation of microlysophagy. Noncanonically lipidated GABARAPs on damaged or stressed lysosomes initiate ESCRT assembly by interacting with ALIX. VPS4 is then recruited to lysosomes, where it disassembles ESCRTs to complete microlysophagy; STK38 and its phosphorylation target, DOK1 are required for this step. Preserving lysosomal integrity by STK38- and GABARAPs-regulated microlysophagy presumably contributes to preventing aging.

and secretion of extracellular vesicles (Sanjuan *et al*, 2007; Leidal *et al*, 2020; Nakamura *et al*, 2020; Kumar *et al*, 2021). ATG8s are ubiquitin-like proteins that are covalently attached to lipids or proteins by a ubiquitination-like conjugation system in response to various stress signals (Kumar *et al*, 2021). Since the ubiquitination of membrane proteins acts as a tag for the assembly of ESCRTs during MVB biogenesis (Raiborg & Stenmark, 2009; Shields & Piper, 2011), we speculate that ATG8s function as the tag for the damaged site that is to be repaired by the ESCRT machinery. Our findings regarding the initiation of ESCRT assembly by GABARAPs provide novel insights into the non-canonical function of ATG8s. It will be interesting to determine whether GABARAPs also regulate other ESCRT-related processes such as closure of autophagosomes (Takahashi *et al*, 2018). It remains unclear what triggers the lipidation of ATG8s on damaged lysosomes, but our previous study showed that non-canonical ATG8 lipidation occurred by pharmacological induction of Ca^{2+} efflux from lysosomes (Nakamura *et al*, 2020). In addition, Ca^{2+} leakage from lysosomes has been proposed as a trigger for ESCRT recruitment to damaged lysosomes (Skowyra *et al*, 2018). Therefore, Ca^{2+} efflux may be the key to GABARAPs-mediated ESCRT recruitment, although further studies are needed.

The Hippo pathway is an evolutionally conserved pathway that controls cellular growth and division, organ size, and tissue homeostasis. The pathway is known to be regulated by diverse signals such as cell–cell contact, cell polarity, cellular mechanotransduction, and G protein–coupled receptor ligands. In addition, recent studies have shown crosstalk between the Hippo pathway and macroautophagy (Tang & Christofori, 2020; Wang *et al*, 2020). However, no crosstalk has been identified between the Hippo pathway and microautophagy. Here we showed that STK38 plays a key role in microlysophagy by regulating VPS4-mediated disassembly of ESCRTs. STK38 has been reported to positively regulate macroautophagy by binding with Beclin1, and to regulate the nuclear export of Beclin1 or YAP via phosphorylation of XPO1 (Joffre *et al*, 2015; Martin *et al*, 2019). On the other hand, STK38 negatively regulates BAG3-mediated autophagy in a kinase activity–independent manner (Klimek *et al*, 2019). Our study reveals an additional function of STK38 in autophagy and identified DOK1 as a downstream factor of STK38. STK38 phosphorylates DOK1 on S269, which is required for lysosomal localization of DOK1 and subsequent recruitment of VPS4. We hypothesize that DOK1 recruited on lysosomes via STK38-mediated phosphorylation acts as a docking site to support VPS4 recruitment to lysosomes. The exact role of DOK1 on VPS4 recruitment is still unclear and needs to be revealed in the future study. Besides this, our screening of Hippo pathway revealed that MST2 and MOB2, an upstream kinase and a co-factor of STK38, respectively, could also work in lysosomal damage response, implying the possible involvement of whole MST2-STK38-MOB2 cascade to regulate microlysophagy.

While compromised macroautophagy is well established as a hallmark of aging (López-Otín *et al*, 2023), roles of microautophagy in aging are poorly understood. Our data show for the first time the possibility that failure of microautophagy that targets damaged lysosomes would lead to cellular senescence and organismal aging. How macroautophagy and microautophagy coordinate the aging process is of particular interest and should be addressed in future studies. A previous study showed that an increased number of damaged lysosomes in senescent cells triggered a decrease in pH, which induced glutaminolysis and thereby sustained the survival of senescent cells (Johmura *et al*, 2021). This process is therefore a promising target of senolysis. Moreover, inhibiting the function of the lysosomal membrane protein SCAV-3, the *C. elegans* homolog of human LIMP-2, was shown to increase the number of damaged lysosomes and shorten lifespan in worms (Li *et al*, 2016), suggesting that longevity requires the maintenance of lysosomal integrity. How this integrity is maintained during cellular senescence and organismal aging is

currently unclear, but further research into microlysophagy could answer this question and pave the way for the prevention of aging and age-related diseases that accompany lysosomal damage.

Materials and Methods

Cell culture

HeLa, Plat-E, U2OS, and RPE1 (Clontech, C4000-1) cells were cultured in Dulbecco's Modified Eagle's Medium (DMEM) with high glucose supplemented with 10% fetal bovine serum and penicillin–streptomycin at 37°C, 5% CO_2. MCF10A cells (ATCC, CRL-10317) were cultured in DMEM/F12 supplemented with 5% horse serum, 20 ng/ml EGF, 10 mg/ml insulin, 0.5 mg/ml hydrocortisone, 100 ng/ml cholera toxin, and penicillin–streptomycin at 37°C, 5% CO_2.

C. elegans strains and growth conditions

Using standard techniques, nematodes were cultured at 20°C on nematode growth medium (NGM) agar plates with the *E. coli* strain OP50 unless otherwise noted. WT N2 Bristol and CX4103 kyIs150 [tax-2 (delta)::GFP + lin-15(+)]; sax-1(ky491)X were obtained from the Caenorhabditis Genetics Center (Minneapolis, MN). The strain qxIs257 [ced-1p::nuc-1::mCherry + unc-76(+)]; qxIs582 (Phyp-7sfGFP::Gal3) was a gift from Dr. Xiaochen Wang (Chinese Academy of Science). CX4103 was outcrossed with our N2 for 4 times to obtain sax-1 (ky491) mutant. This sax-1 mutant was further crossed with qxIs257; qxIs582 to generate SHU28 sax-1 (ky491) X;qxIs257 [ced-1p::nuc-1::mCherry + unc-76(+)]; qxIs582 (Phyp-7sfGFP::Gal3).

Antibodies and reagents

The following antibodies were used in this study: anti-LAMP1 (mouse, 1/1,000; Santa Cruz Biotechnology, sc-20011), anti-Galectin3 (rat, 1/1,000; Santa Cruz Biotechnology, sc-23938), anti-STK38 (mouse, 1/1,000; Santa Cruz Biotechnology, sc-100404), anti-NDR1/2 (Phospho-Thr444/442) (rabbit, 1/1,000; Signalway Antibody, 12521), anti-GAPDH (rabbit, 1/64,000; Cell Signaling Technology, 2118), anti-CHMP4B (rabbit, 1/1,000; Proteintech, 13683-1-AP), anti-ALIX (for immunofluorescence, mouse, 1/1,000; BioLegend, 634502), anti-VPS4 (rabbit, 1/500–1,000; Sigma-Aldrich, SAB4200025), anti-LC3B (rabbit, 1/1,000; MBL, PM036), anti-α-tubulin (rabbit, 1/64,000; MBL, PM054), anti-GFP (rabbit, 1/1,000; Cell Signaling Technology, 2555), anti-α-tubulin (mouse, 1/10,000; Abcam, ab11304), anti-EGFR (sheep, 1/1,000; Fitzgerald, 20-ES04), anti-Atg5 (rabbit, 1/1,000; Cell Signaling Technology, 12994), anti-Atg13 (rabbit, 1/1,000; Cell Signaling Technology, 13468), anti-ubiquitin Lys48-specific (rabbit, 1/1,000; Millipore, 05-1307), anti-p62 (rabbit, 1/1,000; MBL, PM045), anti-CD63 (mouse, 1/500; BD Biosciences, 556019), anti-AHNAK (rabbit, 1/1,000; Proteintech, 16637-1-AP), anti-FLAG (mouse, 1/1,000; Sigma-Aldrich, F1804), anti-HA (rat, 1/1,000; Sigma), anti-RFP (rabbit, 1/1,000; MBL, PM005), anti-ALIX (for western blotting, rabbit, 1/1,000; Sigma-Aldrich, ABC40), anti-TSG101 (mouse, 1/1,000; BD Biosciences, 612696), anti-p21 (rabbit, 1/1,000; Abcam, ab109199), anti-p16 INK4a (mouse, 1 μg/ml; IBL, 11104), anti-LC3A (rabbit, 1/1,000; Cell Signaling Technology, 4599), anti-GABARAP (rabbit, 1/1,000; MBL, PM037), anti-GABARAPL1 (rabbit, 1/1,000; Abcam, ab86497), anti-GABARAPL2 (rabbit, 1/1,000; MBL, PM038). The secondary antibodies used for western blotting were as follows: horseradish peroxidase (HRP)-conjugated goat anti-rabbit IgG (1:5,000; Jackson ImmunoResearch, 111-035-003), HRP-conjugated goat anti-mouse IgG (1:5,000; Jackson ImmunoResearch, 115-035-003), HRP-conjugated rabbit anti-sheep IgG (1:2,000; Jackson Invitrogen, 81-8620), HRP-conjugated goat anti-rat IgG (1:5,000; Jackson ImmunoResearch, 112-035-003. The secondary antibodies used for immunofluorescence were as follows: goat anti-rabbit Alexa Fluor 488 pre-absorbed (1/2,000; Abcam, ab150085), goat anti-mouse IgG (H+L) cross-adsorbed secondary antibody, Alexa Fluor 568 (1/2,000; Invitrogen, A-11004), goat anti-rat Alexa Fluor 647 pre-absorbed (1/2,000; Abcam, ab150167), goat anti-rabbit IgG (H+L) highly cross-absorbed secondary antibody, Alexa Fluor 647 (1/2,000; Invitrogen, A-21245).

For lysosomal damage induction in HeLa cells, cells were treated with 1 mM LLOMe (Sigma-Aldrich, L7393). Cells treated with LLOMe for 1 h were washed with PBS and then cultured in growth media for the times indicated in the figures. BAPTA-AM (Dojindo, 348-05451) was used as a Ca^{2+} chelator. Cells were pre-treated with 10 μM BAPTA-AM for 1 h, then treated with LLOMe for 1 h. For the EGFP-TRPML1 cleavage assay, MCF10A cells were treated with 10 μM monensin (Sigma-Aldrich, M5273) or 5 μM nigericin (Tocris Bioscience, 4312) for 8 h or with 0.5 mM LLOMe for 1 h and cultured in growth medium for 7 h after washout. For the observation of ILVs in endolysosomes by electron microscopy, cells were treated with 5 mM NH_4Cl (Sigma-Aldrich, A9434) for 24 h. For the observation of ILVs in swollen endolysosomes, cells were treated with 50 μM monensin for 1 h, followed by co-treatment with 200 nM Apilimod (Axon Medchem, 1369) for 2 h. For the LC3 flux assay, cells were cultured in growth medium or EBSS (Sigma-Aldrich, E2888) with or without 250 nM bafilomycin A1 (Cayman Chemical, 11038) for 2 h. For the induction of cellular senescence, RPE1 cells were treated with 150 ng/ml doxorubicin (FUJIFILM Wako Chemicals, 040-21521) for 3 days (for WB, qPCR, and SA-β-Gal staining) or 10 days (for GFP-Gal3 imaging).

Plasmid construction

All inserts were amplified from a HeLa cDNA library. The inserts were cloned into pENTR1A vector, then subcloned into pcDNA3.1 vectors (for transient expression) or pMRX-IRES vectors (for stable expression) using the Gateway system (Invitrogen). The In-fusion HD Cloning Kit (Takara Bio) was used for mutagenesis. pcDNA3.1 3×FLAG- human ATG8 paralogs (LC3A, LC3B, LC3C, GABARAP, GABARAPL1, and GABARAPL2) were previously generated (Nakamura *et al*, 2020).

Establishment of stable cell lines

Cells stably expressing proteins were generated by retrovirus transduction using pMRX-IRES vectors. Recombinant retroviruses were prepared using Plat-E cells as previously described (Morita *et al*, 2000; Saitoh *et al*, 2003). After viral infection, cells were selected by antibiotics (puromycin or blasticidin) and flow cytometric cell sorting (optional, for the selection of cells expressing fluorescent protein–tagged targets). HeLa cells stably expressing TFEB-mNeonGreen and GFP-Gal3 were previously generated (Maejima *et al*, 2013; Nakamura *et al*, 2020).

© 2023 The Authors

Establishment of KO cell lines

Autophagy-deficient KO HeLa cell lines were previously generated (Nakamura *et al*, 2020). STK38 KO HeLa cells were generated using the following CRISPR guide RNA (gRNA): Fw: CACCTGTCA CGCTCCGCACGAATG; Rv: AAACCATTCGTGCGGAGCGTGACA. Annealed gRNA oligonucleotides were inserted into a px458 vector, and the gRNA construct was transfected into HeLa cells using ViaFect (Promega) transfection reagent. GFP-positive single cells were sorted by flow cytometry into 96-well plates. Candidate single-clone colonies were verified by immunoblotting and gene sequencing.

siRNA or plasmid transfection

siRNAs were purchased from Sigma-Aldrich or Thermo Fisher Scientific (for siRNA-based screening in Fig 4F). siRNAs were transfected into cells using Lipofectamine RNAiMAX (Invitrogen). Cells were used for subsequent experiments after 48 h of siRNA transfection. Sequences of siRNAs used in this study are listed in Appendix Table S1. The silencing efficacy was assessed by western blotting or quantitative PCR. For transient expression of proteins, plasmids were transfected into cells using Lipofectamine 2000 (Invitrogen). Cells were used for the subsequent experiment after 24 h of plasmid transfection.

Gal3 dot-count assay

HeLa cells expressing GFP-Gal3 were cultured on collagen-coated 96-well plates (PerkinElmer, 6055300). After LLOMe treatment, cells were fixed with 4% PFA for 20 min and washed with PBS twice. After fixation, cells were incubated with Hoechst 33342 (1/1,000) and Cell Mask Deep Red (1/100,000) in PBS for 40 min at room temperature. Sample observation and image acquisition were performed by CQ1 (Yokogawa), and the number of Gal3 dots per cell was counted using CQ1 software (Yokogawa, version 1.07.01.01).

Immunofluorescence and microscopy

Cells were cultured on collagen-coated coverslips and fixed with 4% PFA for 20 min. After fixation, cells were permeabilized with 50 μg/ml digitonin in PBS for 10 min, blocked with 0.1% gelatin in PBS, and then incubated with the indicated primary antibodies for 1 h. After incubation with secondary antibody (1/2,000) for 40 min, coverslips were mounted on slide glasses with VECTASHIELD Antifade Mounting Medium (Vector Laboratories). Sample observation and image acquisition were performed with an FLUOVIEW FV3000RS Confocal Laser Scanning Microscope (Olympus) operated using FV31S-SW software (version 2.3.1.163) or an IX83 Inverted Microscope (Olympus) operated using MetaMorph (version 7.10.1.161). The number of puncta and the colocalization rate were analyzed using Fiji (version 2.1.0/1.53c). The nucleus/cytoplasm ratio of TFEB was analyzed using CellProfiler (version 3.1.8) as previously described (Ogura *et al*, 2022).

Western blotting

Cells harvested in sample buffer were boiled at 90°C for 5 min or 60°C for 10 min (for the EGFP-TRPML1 cleavage assay). Protein concentrations of samples were measured using the Protein Quantification Assay Kit (MACHEREY-NAGEL). Samples were subjected to SDS–PAGE and transferred to PVDF membranes. The membranes were blocked with 1% skim milk in TBST and incubated overnight at 4°C with primary antibodies diluted in blocking solution. The membranes were washed three times with TBST, incubated for 1 h at room temperature with HRP-conjugated secondary antibodies (1/5,000 in blocking solution), and washed four times with TBST. The immunoreactive bands were detected using a ChemiDoc Touch imaging system (Bio-Rad).

Lysotracker and Magic Red staining

HeLa cells cultured on collagen-coated 96-well plates (PerkinElmer, 6055300) were incubated with Lysotracker (50 nM) or Magic Red (1/250) at 37°C for 30 min. Then, cells were fixed with 4% PFA for 20 min and washed with PBS twice. After fixation, cells were incubated with Hoechst 33342 (1/1,000) and Cell Mask Green (1/100,000) in PBS for 40 min at room temperature. Sample observation and image acquisition were performed by CQ1 (Yokogawa), and the mean intensity of Lysotracker or Magic Red per cell was calculated using CQ1 software (Yokogawa, version 1.07.01.01).

EGFR degradation assay

HeLa cells were washed with PBS twice and incubated in serum-free medium for 2 h. Then, cells were incubated in serum-free medium with EGF (50 ng/ml). Cells were harvested in lysis buffer (20 mM Tris–HCl pH 7.6, 150 mM NaCl, and 2% TritonX-100) supplemented with cOmplete EDTA-free protease inhibitor cocktail (Roche) and PhosSTOP (Roche) and subjected to immunoblot analysis.

Correlative light-electron microscopy (CLEM)

MCF10A cells were cultured on gridded 35 mm glass bottom dishes (MatTek, P35G-1.5-14-C-GRD). After treatment with NH_4Cl for 24 h, cells were fixed with 4% formaldehyde in 0.1 M phosphate buffer (pH 7.4) for 30 min, then washed with 0.1 M phosphate buffer containing 4% sucrose. For nuclear staining, cells were incubated with DAPI in 0.1 M phosphate buffer for 30 min. After washing with 0.1 M phosphate buffer, fluorescent images of the target cell were obtained by FLUOVIEW FV3000RS Confocal Laser Scanning Microscope (Olympus). At the same time, DIC images of the same cell were taken with letters around the target cell on the glass bottom dish at 100 fold magnification so as to confirm the location of the cell in the dish. After fluorescent imaging, cells were additionally fixed for 60 min with 1% formaldehyde and 1% glutaraldehyde in 0.1 M phosphate buffer and washed with 0.1 M phosphate buffer containing 4% sucrose. Then, cells were post-fixed for 60 min with 1% osmium tetroxide and 0.8% potassium ferrocyanide in 0.1 M phosphate buffer, washed in H_2O, dehydrated in graded series of ethanol and embedded in a plastic cup which is 8 mm diameter filled with Epon 812 (TAAB Co. Ltd., UK) resin. 80-nm ultrathin sections containing the target cell were stained with saturated uranyl acetate and Reynolds lead citrate solution. Electron micrographs were captured on a CCD camera Olympus Veleta 2K × 2K side-mounted on a JEM-1400plus transmission electron microscope (JEOL, Japan) at 80 kV.

© 2023 The Authors

Transmission electron microscopy (TEM)

Samples for TEM were prepared as previously described (Oe *et al*, 2022). Briefly, MCF10A cells cultured on 12-well plates were treated with NH_4Cl for 24 h. Cells were fixed with 2.5% glutaraldehyde in PBS and then incubated in 2% OsO_4 solution. After the samples were embedded in Quetol812 (Nisshin EM), 80-nm ultrathin sections were obtained using an Ultracut E ultramicrotome (Reichert-Jung). These sections were stained with a solution of uranyl acetate and lead and observed using a transmission electron microscope (Hitachi, H-7650).

TMT-based quantitative phosphoproteomics

HeLa cells transfected with siLuc or siSTK38 were cultured in 100-mm dishes. After 48 h transfection, cells were treated with LLOMe for 1 h and incubated for 5 h after washout. After washing with HBS, cells were lysed in three biological replicates in 500 µl guanidine buffer (6 M guanidine-HCl, 100 mM HEPES-NaOH pH 7.5, 10 mM TCEP, and 40 mM chloroacetamide). After heating and sonication, proteins (330 µg each) were purified by methanol–chloroform precipitation and resuspended in 37 µl 0.1% RapiGest SF (Waters) in 50 mM triethylammonium bicarbonate. After sonication and heating at 95°C for 10 min, the proteins were digested with 5 µg trypsin/Lys-C mix (Promega) at 37°C overnight. The digested peptides (200 µg each) were labeled with 0.5 mg TMTpro-18plex reagents (Thermo Fisher Scientific) at 25°C for 1 h. After the reaction was quenched with hydroxylamine, all the TMT-labeled samples were pooled, acidified with TFA, and subjected to phosphopeptide enrichment using the High-Select Fe-NTA phosphopeptide enrichment kit (Thermo Fisher Scientific). The eluate was fractionated by offline high-pH reversed-phase chromatography on a Vanquish DUO UHPLC (Thermo Fisher Scientific). Briefly, the peptides were loaded onto a 4.6 mm × 250 mm Xbridge BEH130 C18 column with 3.5-mm particles (Waters) and separated using a 30-min multistep gradient of solvents A (10 mM ammonium formate at pH 9.0 in 2% ACN) and B (10 mM ammonium formate pH 9.0 in 80% ACN) at a flow rate of 1 ml/min. Peptides were separated into 48 fractions, which were then consolidated into 16 fractions. Each fraction was evaporated in a SpeedVac concentrator and dissolved in 0.1% TFA and 3% ACN. LC–MS/MS analysis of the resultant peptides was performed on an EASY-nLC 1200 UHPLC connected to a Q Exactive Plus mass spectrometer through a nanoelectrospray ion source (Thermo Fisher Scientific). The peptides were separated on the analytical column (75 µm × 15 cm, 3 µm; Nikkyo Technos) with a linear gradient of 4–32% for 0–100 min, followed by an increase to 80% ACN for 10 min, and finally held at 80% ACN for 10 min. The mass spectrometer was operated in data-dependent acquisition mode with a top 10 MS/MS method. MS1 spectra were measured with a resolution of 70,000, an AGC target of 3e6, and a mass range from 375 to 1,400 *m/z*. MS/MS spectra were triggered at a resolution of 35,000, an AGC target of 1e5, an isolation window of 0.7 *m/z*, a maximum injection time of 150 ms, and a normalized collision energy of 33. Dynamic exclusion was set to 20 s. Raw data were directly analyzed against the SwissProt database restricted to *Homo sapiens* using Proteome Discoverer version 2.5 (Thermo Fisher Scientific) with the Sequest HT search engine for identification and TMT quantification. The search parameters were as follows: (a) trypsin as an enzyme with up to two missed cleavages; (b) precursor mass tolerance of 10 ppm; (c) fragment mass tolerance of 0.02 Da; (d) TMTpro of lysine and peptide N-terminus and carbamidomethylation of cysteine as fixed modifications; and (e) oxidation of methionine and phosphorylation of serine, threonine, and tyrosine as variable modifications. Peptides were filtered at a false discovery rate of 1% using the Percolator node. TMT quantification was performed using the Reporter Ions Quantifier node. Normalization was performed such that the total sum of the abundance values for each TMT channel was the same over all peptides. Volcano plots were created using Perseus (Max Planck Institute of Biochemistry).

Parallel reaction monitoring (PRM) mass spectrometry

mNG-DOK1 stably expressing HeLa cells were transfected with siLuc or siSTK38 in 100-mm dishes. After 48 h transfection, cells were treated with 1 mM LLOMe for 30 min or 1 h and incubated for 5 h after washout. After washing with HBS, cells were lysed in 1 ml HEPES-RIPA buffer (20 mM HEPES-NaOH pH7.5, 1 mM EGTA, 1 mM $MgCl_2$, 150 mM NaCl, 0.25% Na-deoxycholate, 0.05% SDS, and 1% NP40). After addition of 0.15% SDS and sonication, lysates were centrifuged at 20,400 *g* at 4°C for 15 min. Supernatants were incubated with a 2.5 µl slurry of mNG-Trap magnetic agarose (ChromoTek) at 4°C for 2 h. The beads were washed three times with HEPES-RIPA buffer and then twice with 50 mM ammonium bicarbonate and 8 mM $CaCl_2$. Proteins on the beads were digested with 200 ng trypsin/LysC mix (Promega) at 37°C overnight. The digests were reduced, alkylated, acidified, and desalted using GL-Tip SDB (GL Sciences). The eluates were evaporated and dissolved in 0.1% TFA and 3% ACN. LC–MS/MS analysis of the resultant peptides was performed on an EASY-nLC 1200 UHPLC connected to an Orbitrap Fusion mass spectrometer through a nanoelectrospray ion source (Thermo Fisher Scientific). The mass spectrometer was operated in data-dependent acquisition mode with a maximum duty cycle of 3 s. MS1 spectra were measured with a resolution of 60,000, an AGC target of 4e5, and a mass range from 350 to 1,500 *m/z*. HCD MS/MS spectra were acquired in the Orbitrap with a resolution of 30,000, an AGC target of 5e4, an isolation window of 1.6 *m/z*, a maximum injection time of 54 ms, and a normalized collision energy of 30. Dynamic exclusion was set to 15 s. Raw data were directly analyzed against the SwissProt database restricted to *Homo sapiens* using Proteome Discoverer version 2.5 with the Sequest HT search engine. The search parameters were as follows: (a) trypsin as an enzyme with up to two missed cleavages; (b) precursor mass tolerance of 10 ppm; (c) fragment mass tolerance of 0.02 Da; (d) carbamidomethylation of cysteine as a fixed modification; and (e) acetylation of protein N-terminus, oxidation of methionine, and phosphorylation of serine, threonine, and tyrosine as variable modifications. Then, several selected peptides of DOK1 were measured by PRM, an MS/MS-based targeted quantification method using high-resolution MS. Targeted HCD MS/MS scans were acquired by a time-scheduled inclusion list at a resolution of 30,000, an AGC target of 5e4, an isolation window of 1.6 *m/z*, a maximum injection time of 500 ms, and a normalized collision energy of 30. Time alignment and relative quantification of the transitions were performed using Skyline software.

 © 2023 The Authors

Co-immunoprecipitation

Before lysis, cells were treated with formaldehyde (final 0.1%) and incubated for 10 min at room temperature for protein crosslinking. Then, 1 M glycine-NaOH pH7.5 (final 0.1 M) was added, and cells were incubated for 4 min at room temperature. After washing with ice-cold HBS twice, cells were lysed in HEPES-RIPA buffer (20 mM HEPES-NaOH pH 7.5, 150 mM NaCl, 1 mM $MgCl_2$, 1 mM EGTA, 1% Nonidet P-40, 0.25% sodium deoxycholate, and 0.05% SDS) supplemented with cOmplete EDTA-free protease inhibitor cocktail, PhosSTOP, and Benzonase (Millipore). After sonication, lysates were centrifuged at 20,400 *g* at 4°C for 10 min. Supernatants were incubated with M2 anti-Flag-beads agarose (Sigma-Aldrich, A2220) or RFP-Trap agarose (Chromotek, gta) at 4°C for 2 h, then washed three times with HEPES-RIPA buffer, eluted with 2 × sample buffer, and subjected to immunoblot analysis.

RNA extraction and quantitative PCR

Total RNA was extracted from the cells using the RNeasy plus mini kit (Qiagen). cDNA was generated using an iScript cDNA synthesis kit (Bio-Rad). Quantitative PCR with reverse transcription was performed using Power SYBR Green (Thermo Fisher Scientific) on a QuantStudio7 system (Applied Biosystems). GAPDH was used as an internal control. The primer pair sequences used in this study are listed in Appendix Table S2.

SA-β-Gal staining

SA-β-Gal staining was performed using the Senescence Cells Histochemical Staining Kit (Sigma, CS0030). Briefly, senescence-induced cells cultured on collagen-coated coverslips were fixed with Fixation buffer for 7 min at room temperature. After washing with PBS three times, cells were stained with Staining mixture at 37°C for 18 h without CO_2 and then observed using a microscope (BX53, Olympus) operated using cellSens (version 1.16).

Lifespan assay

Synchronized eggs were obtained by 4–6-h egg lay. When they reached the day 1 adult stage, lifespan experiments were set up at a density of 20 animals per NGM plate or RNAi plate and carried out at 20°C. These animals were transferred to new plates every other day. Worm survival was assessed every other day. Death was defined as the absence of any movement after stimulation by a platinum wire. Strains and conditions were blinded in all lifespan experiments. Survival curves were plotted with GraphPad Prism 9 (GraphPad Software) and statistical analyses were performed with the Mantel-Cox log-rank method in Excel (Microsoft).

RNAi in *C. elegans*

RNAi was conducted by feeding *C. elegans* worms HT115 (DE3) bacteria transformed with an L4440 vector that produces dsRNA against the targeted gene (*luciferase*, *lgg-1* and *lgg-2*). Synchronized day-1 adult worms obtained by egg lay were placed on corresponding RNAi plates containing 1 mM IPTG and 100 mg/ml ampicillin. The *luciferase* RNAi clones *lgg-1* and *lgg-2* were gifts from Dr. Antebi (MPI-Age).

Microscopy and quantification of damaged lysosomes in *C. elegans*

Fluorescence images of GFP::Gal3 in hypodermal cells on day 8 were captured using an FV3000 confocal microscope (Olympus). Numbers of GFP::Gal3 dots were counted and normalized by area using Fiji. Experiments were repeated three times and data obtained from more than 29 worms per conditions were used.

Quantification and statistical analysis

All analyses were performed using GraphPad Prism 9 software. All quantitative data are denoted as means ± standard deviation (SD) from $n \geq 3$ independent experiments unless otherwise indicated. Statistical analyses were assessed using either the unpaired *t*-test or one-way ANOVA followed by Dunnett's or Tukey's multiple comparisons test. *P*-values below 0.05 were considered significant. ns = not significant. No blinding was done.

Data availability

The MS proteomics data have been deposited to the ProteomeXchange Consortium via the jPOST partner repository with the dataset identifier PXD039312 (http://www.ebi.ac.uk/pride/archive/projects/PXD039312).

Expanded View for this article is available online.

Acknowledgements
We thank Dr. Michael Lazarou (Monash University) for providing ATG8 deficient HeLa cells, Dr. Richard J. Youle (National Institutes of Health) for providing penta KO HeLa cells, Dr. Michael Overholtzer (Memorial Sloan Kettering Cancer Center) for providing EGFP-TRPML1 and LAMP1-GFP MCF10A cells, and Dr. Toshio Kitamura (The University of Tokyo) for providing Plat-E cells. We thank Dr. Shoji Yamaoka (Tokyo Medical and Dental University) for providing pMRX-IRES plasmids. We thank the Caenorhabditis Genetics Center (CGC) at the University of Minnesota, and Dr. Xiaochen Wang (Chinese Academy of Sciences) for providing *C. elegans* strains. We thank Dr. Adam Antebi (Max Planck Institute for Biology of Aging) for providing lgg-1 and lgg-2 RNA interference (RNAi) clones. We also thank Dr. Eiji Hara (Osaka University) for helpful suggestion on this project. TMT-based quantitative phosphoproteomics was performed under the Joint Usage and Joint Research Programs of the Institute of Advanced Medical Sciences, Tokushima University. The CLEM experiment was performed in cooperation with Hiroko Omori, a member of the Electron Microscopy Division of Core instrumentation facility of the Research Institute for Microbial Diseases, Osaka University. TEM imaging was supported by the Center for Medical Research and Education, Graduate School of Medicine, Osaka University. MO is supported by Japan Society for the Promotion of Science (JSPS) KAKENHI (21J21647). TY is supported by JST Core Research for Evolutional Science and Technology (CREST) (grant no. JPMJCR17H6), Japan Agency for Medical Research and Development (AMED) CREST (grant no. JP22gm1410014), JSPS KAKENHI (22H04982) and the Takeda Science Foundation. SN is supported by Ministry of Education, Culture, Sports, Science and Technology (MEXT) KAKENHI, a Grant-in-Aid for Transformative Research Areas B (21H05145), JSPS KAKENHI (21H02428, 23K18140), the Astellas Foundation for Research on Metabolic Disorders, the Mochida Memorial Foundation for Medical and Pharmaceutical Research, Research

© 2023 The Authors

grants in the natural sciences, the Mitsubishi Foundation, and the NOVARTIS Foundation (Japan) for the Promotion of Science.

Author contributions

Monami Ogura: Conceptualization; resources; investigation; methodology; writing – original draft. **Tatsuya Kaminishi:** Investigation. **Takayuki Shima:** Resources. **Miku Torigata:** Investigation. **Nao Bekku:** Investigation. **Keisuke Tabata:** Resources. **Satoshi Minami:** Resources. **Kohei Nishino:** Data curation; investigation. **Akiko Nezu:** Resources. **Maho Hamasaki:** Supervision. **Hidetaka Kosako:** Data curation; supervision. **Tamotsu Yoshimori:** Conceptualization; supervision; project administration; writing – review and editing. **Shuhei Nakamura:** Conceptualization; resources; supervision; investigation; methodology; writing – original draft; project administration; writing – review and editing.

Disclosure and competing interests statement

TY and SN are the founders of AutoPhagyGO.

References

Ballabio A, Bonifacino JS (2020) Lysosomes as dynamic regulators of cell and organismal homeostasis. *Nat Rev Mol Cell Biol* 21: 101 – 118

Boyle KB, Ellison CJ, Elliott PR, Schuschnig M, Grimes K, Dionne MS, Sasakawa C, Munro S, Martens S, Randow F (2023) TECPR1 conjugates LC3 to damaged endomembranes upon detection of sphingomyelin exposure. *EMBO J* 42: e113012

Brummer T, Schmitz-Peiffer C, Daly RJ (2010) Docking proteins. *FEBS J* 277: 4356 – 4369

Bussi C, Peralta Ramos JM, Arroyo DS, Gallea JI, Ronchi P, Kolovou A, Wang JM, Florey O, Celej MS, Schwab Y *et al* (2018) Alpha-synuclein fibrils recruit TBK1 and OPTN to lysosomal damage sites and induce autophagy in microglial cells. *J Cell Sci* 131: jcs226241

Carpino N, Wisniewski D, Strife A, Marshak D, Kobayashi R, Stillman B, Clarkson B (1997) p62(dok): a constitutively tyrosine-phosphorylated, GAP-associated protein in chronic myelogenous leukemia progenitor cells. *Cell* 88: 197 – 204

Chauhan S, Kumar S, Jain A, Ponpuak M, Mudd MH, Kimura T, Choi SW, Peters R, Mandell M, Bruun J-A *et al* (2016) TRIMs and galectins globally cooperate and TRIM16 and Galectin-3 co-direct autophagy in endomembrane damage homeostasis. *Dev Cell* 39: 13 – 27

Corkery DP, Castro-Gonzalez S, Knyazeva A, Herzog LK, Wu Y-W (2023) An ATG12-ATG5-TECPR1 E3-like complex regulates unconventional LC3 lipidation at damaged lysosomes. *EMBO Rep* 24: e56841

Duhart JC, Raftery LA (2020) Mob family proteins: regulatory partners in Hippo and Hippo-like intracellular signaling pathways. *Front Cell Dev Biol* 8: 161

Gabandé-Rodríguez E, Boya P, Labrador V, Dotti CG, Ledesma MD (2014) High sphingomyelin levels induce lysosomal damage and autophagy dysfunction in Niemann Pick disease type A. *Cell Death Differ* 21: 864 – 875

Gógl G, Schneider KD, Yeh BJ, Alam N, Nguyen Ba AN, Moses AM, Hetényi C, Reményi A, Weiss EL (2015) The structure of an NDR/LATS kinase-mob complex reveals a novel kinase-coactivator system and substrate docking mechanism. *PLoS Biol* 13: e1002146

Goodwin JM, Walkup WG IV, Hooper K, Li T, Kishi-Itakura C, Ng A, Lehmberg T, Jha A, Kommineni S, Fletcher K *et al* (2021) GABARAP sequesters the FLCN-FNIP tumor suppressor complex to couple autophagy with lysosomal biogenesis. *Sci Adv* 7: eabj2485

Hammerling BC, Najor RH, Cortez MQ, Shires SE, Leon LJ, Gonzalez ER, Boassa D, Phan S, Thor A, Jimenez RE *et al* (2017) A Rab5 endosomal pathway mediates Parkin-dependent mitochondrial clearance. *Nat Commun* 8: 14050

Hergovich A, Stegert MR, Schmitz D, Hemmings BA (2006) NDR kinases regulate essential cell processes from yeast to humans. *Nat Rev Mol Cell Biol* 7: 253 – 264

Jacquin E, Leclerc-Mercier S, Judon C, Blanchard E, Fraitag S, Florey O (2017) Pharmacological modulators of autophagy activate a parallel noncanonical pathway driving unconventional LC3 lipidation. *Autophagy* 13: 854 – 867

Joffre C, Dupont N, Hoa L, Gomez V, Pardo R, Gonçalves-Pimentel C, Achard P, Bettoun A, Meunier B, Bauvy C *et al* (2015) The pro-apoptotic STK38 kinase is a new Beclin1 partner positively regulating autophagy. *Curr Biol* 25: 2479 – 2492

Johmura Y, Yamanaka T, Omori S, Wang T-W, Sugiura Y, Matsumoto M, Suzuki N, Kumamoto S, Yamaguchi K, Hatakeyama S *et al* (2021) Senolysis by glutaminolysis inhibition ameliorates various age-associated disorders. *Science* 371: 265 – 270

Kaur N, de la Ballina LR, Haukaas HS, Torgersen ML, Radulovic M, Munson MJ, Sabirsh A, Stenmark H, Simonsen A, Carlsson SR *et al* (2023) TECPR1 is activated by damage-induced sphingomyelin exposure to mediate noncanonical autophagy. *EMBO J* 42: e113105

Klimek C, Jahnke R, Wördehoff J, Kathage B, Stadel D, Behrends C, Hergovich A, Höhfeld J (2019) The Hippo network kinase STK38 contributes to protein homeostasis by inhibiting BAG3-mediated autophagy. *Biochim Biophys Acta Mol Cell Res* 1866: 1556 – 1566

Kumar S, Jia J, Deretic V (2021) Atg8ylation as a general membrane stress and remodeling response. *Cell Stress Chaperones* 5: 128 – 142

Lazarou M, Sliter DA, Kane LA, Sarraf SA, Wang C, Burman JL, Sideris DP, Fogel AI, Youle RJ (2015) The ubiquitin kinase PINK1 recruits autophagy receptors to induce mitophagy. *Nature* 524: 309 – 314

Lee C, Lamech L, Johns E, Overholtzer M (2020) Selective lysosome membrane turnover is induced by nutrient starvation. *Dev Cell* 55: 289 – 297

Leidal AM, Huang HH, Marsh T, Solvik T, Zhang D, Ye J, Kai F, Goldsmith J, Liu JY, Huang Y-H *et al* (2020) The LC3-conjugation machinery specifies the loading of RNA-binding proteins into extracellular vesicles. *Nat Cell Biol* 22: 187 – 199

Lemasters JJ (2014) Variants of mitochondrial autophagy: types 1 and 2 mitophagy and micromitophagy (Type 3). *Redox Biol* 2: 749 – 754

Li Y, Chen B, Zou W, Wang X, Wu Y, Zhao D, Sun Y, Liu Y, Chen L, Miao L *et al* (2016) The lysosomal membrane protein SCAV-3 maintains lysosome integrity and adult longevity. *J Cell Biol* 215: 167 – 185

Loi M, Raimondi A, Morone D, Molinari M (2019) ESCRT-III-driven piecemeal micro-ER-phagy remodels the ER during recovery from ER stress. *Nat Commun* 10: 5058

López-Otín C, Blasco MA, Partridge L, Serrano M, Kroemer G (2023) Hallmarks of aging: an expanding universe. *Cell* 186: 243 – 278

Maejima I, Takahashi A, Omori H, Kimura T, Takabatake Y, Saitoh T, Yamamoto A, Hamasaki M, Noda T, Isaka Y *et al* (2013) Autophagy sequesters damaged lysosomes to control lysosomal biogenesis and kidney injury. *EMBO J* 32: 2336 – 2347

Martin AP, Jacquemyn M, Lipecka J, Chhuon C, Aushev VN, Meunier B, Singh MK, Carpi N, Piel M, Codogno P *et al* (2019) STK38 kinase acts as XPO1 gatekeeper regulating the nuclear export of autophagy proteins and other cargoes. *EMBO Rep* 20: e48150

Mashima R, Hishida Y, Tezuka T, Yamanashi Y (2009) The roles of Dok family adapters in immunoreceptor signaling. *Immunol Rev* 232: 273 – 285

 © 2023 The Authors

Mejlvang J, Olsvik H, Svenning S, Bruun J-A, Abudu YP, Larsen KB, Brech A, Hansen TE, Brenne H, Hansen T *et al* (2018) Starvation induces rapid degradation of selective autophagy receptors by endosomal microautophagy. *J Cell Biol* 217: 3640 – 3655

Mercier V, Larios J, Molinard G, Goujon A, Matile S, Gruenberg J, Roux A (2020) Endosomal membrane tension regulates ESCRT-III-dependent intra-lumenal vesicle formation. *Nat Cell Biol* 22: 947 – 959

Morita S, Kojima T, Kitamura T (2000) Plat-E: an efficient and stable system for transient packaging of retroviruses. *Gene Ther* 7: 1063 – 1066

Nakamura S, Shigeyama S, Minami S, Shima T, Akayama S, Matsuda T, Esposito A, Napolitano G, Kuma A, Namba-Hamano T *et al* (2020) LC3 lipidation is essential for TFEB activation during the lysosomal damage response to kidney injury. *Nat Cell Biol* 22: 1252 – 1263

Nguyen TN, Padman BS, Usher J, Oorschot V, Ramm G, Lazarou M (2016) Atg8 family LC3/GABARAP proteins are crucial for autophagosome-lysosome fusion but not autophagosome formation during PINK1/Parkin mitophagy and starvation. *J Cell Biol* 215: 857 – 874

Oe Y, Kakuda K, Yoshimura S-I, Hara N, Hasegawa J, Terawaki S, Kimura Y, Ikenaka K, Suetsugu S, Mochizuki H *et al* (2022) PACSIN1 is indispensable for amphisome-lysosome fusion during basal autophagy and subsets of selective autophagy. *PLoS Genet* 18: e1010264

Ogura M, Shima T, Yoshimori T, Nakamura S (2022) Protocols to monitor TFEB activation following lysosomal damage in cultured cells using microscopy and immunoblotting. *STAR Protoc* 3: 101018

Omari S, Makareeva E, Roberts-Pilgrim A, Mirigian L, Jarnik M, Ott C, Lippincott-Schwartz J, Leikin S (2018) Noncanonical autophagy at ER exit sites regulates procollagen turnover. *Proc Natl Acad Sci USA* 115: E10099 – E10108

Papadopoulos C, Meyer H (2017) Detection and clearance of damaged lysosomes by the endo-lysosomal damage response and lysophagy. *Curr Biol* 27: R1330 – R1341

Papadopoulos C, Kirchner P, Bug M, Grum D, Koerver L, Schulze N, Poehler R, Dressler A, Fengler S, Arhzaouy K *et al* (2017) VCP/p97 cooperates with YOD1, UBXD1 and PLAA to drive clearance of ruptured lysosomes by autophagy. *EMBO J* 36: 135 – 150

Radulovic M, Schink KO, Wenzel EM, Nähse V, Bongiovanni A, Lafont F, Stenmark H (2018) ESCRT-mediated lysosome repair precedes lysophagy and promotes cell survival. *EMBO J* 37: e99753

Radulovic M, Wenzel EM, Gilani S, Holland LK, Lystad AH, Phuyal S, Olkkonen VM, Brech A, Jäättelä M, Maeda K *et al* (2022) Cholesterol transfer via endoplasmic reticulum contacts mediates lysosome damage repair. *EMBO J* 41: e112677

Raiborg C, Stenmark H (2009) The ESCRT machinery in endosomal sorting of ubiquitylated membrane proteins. *Nature* 458: 445 – 452

Rajamäki K, Lappalainen J, Oörni K, Välimäki E, Matikainen S, Kovanen PT, Eklund KK (2010) Cholesterol crystals activate the NLRP3 inflammasome in human macrophages: a novel link between cholesterol metabolism and inflammation. *PLoS One* 5: e11765

Sahu R, Kaushik S, Clement CC, Cannizzo ES, Scharf B, Follenzi A, Potolicchio I, Nieves E, Cuervo AM, Santambrogio L (2011) Microautophagy of cytosolic proteins by late endosomes. *Dev Cell* 20: 131 – 139

Saitoh T, Nakayama M, Nakano H, Yagita H, Yamamoto N, Yamaoka S (2003) TWEAK induces NF-kappaB2 p100 processing and long lasting NF-kappaB activation. *J Biol Chem* 278: 36005 – 36012

Sanjuan MA, Dillon CP, Tait SWG, Moshiach S, Dorsey F, Connell S, Komatsu M, Tanaka K, Cleveland JL, Withoff S *et al* (2007) Toll-like receptor signalling in macrophages links the autophagy pathway to phagocytosis. *Nature* 450: 1253 – 1257

Sardiello M, Palmieri M, di Ronza A, Medina DL, Valenza M, Gennarino VA, Di Malta C, Donaudy F, Embrione V, Polishchuk RS *et al* (2009) A gene network regulating lysosomal biogenesis and function. *Science* 325: 473 – 477

Schöneberg J, Lee I-H, Iwasa JH, Hurley JH (2017) Reverse-topology membrane scission by the ESCRT proteins. *Nat Rev Mol Cell Biol* 18: 5 – 17

Schuck S (2020) Microautophagy - distinct molecular mechanisms handle cargoes of many sizes. *J Cell Sci* 133: jcs246322

Schulze RJ, Krueger EW, Weller SG, Johnson KM, Casey CA, Schott MB, McNiven MA (2020) Direct lysosome-based autophagy of lipid droplets in hepatocytes. *Proc Natl Acad Sci USA* 117: 32443 – 32452

Shields SB, Piper RC (2011) How ubiquitin functions with ESCRTs. *Traffic* 12: 1306 – 1317

Skowyra ML, Schlesinger PH, Naismith TV, Hanson PI (2018) Triggered recruitment of ESCRT machinery promotes endolysosomal repair. *Science* 360: eaar5078

Takahashi Y, He H, Tang Z, Hattori T, Liu Y, Young MM, Serfass JM, Chen L, Gebru M, Chen C *et al* (2018) An autophagy assay reveals the ESCRT-III component CHMP2A as a regulator of phagophore closure. *Nat Commun* 9: 2855

Tamaskovic R, Bichsel SJ, Rogniaux H, Stegert MR, Hemmings BA (2003) Mechanism of Ca^{2+} –mediated regulation of NDR protein kinase through autophosphorylation and phosphorylation by an upstream kinase. *J Biol Chem* 278: 6710 – 6718

Tan JX, Finkel T (2022) A phosphoinositide signalling pathway mediates rapid lysosomal repair. *Nature* 609: 815 – 821

Tang F, Christofori G (2020) The cross-talk between the Hippo signaling pathway and autophagy:implications on physiology and cancer. *Cell Cycle* 19: 2563 – 2572

Terman A, Kurz T, Gustafsson B, Brunk UT (2006) Lysosomal labilization. *IUBMB Life* 58: 531 – 539

Thurston TLM, Wandel MP, von Muhlinen N, Foeglein A, Randow F (2012) Galectin 8 targets damaged vesicles for autophagy to defend cells against bacterial invasion. *Nature* 482: 414 – 418

Uchimoto T, Nohara H, Kamehara R, Iwamura M, Watanabe N, Kobayashi Y (1999) Mechanism of apoptosis induced by a lysosomotropic agent, L-Leucyl-L-Leucine methyl ester. *Apoptosis* 4: 357 – 362

Vietri M, Radulovic M, Stenmark H (2020) The many functions of ESCRTs. *Nat Rev Mol Cell Biol* 21: 25 – 42

Wang F, Gómez-Sintes R, Boya P (2018) Lysosomal membrane permeabilization and cell death. *Traffic* 19: 918 – 931

Wang D, He J, Huang B, Liu S, Zhu H, Xu T (2020) Emerging role of the Hippo pathway in autophagy. *Cell Death Dis* 11: 880

Wang L, Klionsky DJ, Shen H-M (2022) The emerging mechanisms and functions of microautophagy. *Nat Rev Mol Cell Biol* 24: 186 – 203

Weidberg H, Shvets E, Shpilka T, Shimron F, Shinder V, Elazar Z (2010) LC3 and GATE-16/GABARAP subfamilies are both essential yet act differently in autophagosome biogenesis. *EMBO J* 29: 1792 – 1802

Yamanashi Y, Baltimore D (1997) Identification of the Abl- and rasGAP-associated 62 kDa protein as a docking protein, Dok. *Cell* 88: 205 – 211

Zhang W, Yang X, Chen L, Liu Y-Y, Venkatarangan V, Reist L, Hanson P, Xu H, Wang Y, Li M (2021) A conserved ubiquitin- and ESCRT-dependent pathway internalizes human lysosomal membrane proteins for degradation. *PLoS Biol* 19: e3001361

License: This is an open access article under the terms of the Creative Commons Attribution License, which permits use, distribution and reproduction in any medium, provided the original work is properly cited.

Expanded View Figures

Figure EV1. siRNA-based screening identifies several Hippo pathway components required for clearance of damaged lysosomes.

A Representative images of stably expressed GFP-Gal3 (green) in HeLa cells transfected with siRNAs of Hippo pathway components. Cells were treated with LLOMe (1 mM for 1 h, then incubated for the indicated number of hours after washout). Scale bars: 10 μm.
B Percentage of residual GFP-Gal3 dots (10 h/0 h [%]) in (A). ≥ 100 cells were analyzed per experiment for each condition.
C Representative images of transiently expressed mNG-MOB2 (green) and immunostained LAMP1 (magenta) in HeLa cells treated with LLOMe (1 mM for 1 h, then incubated for 3 h after washout) or EBSS (for 4 h). Scale bars: 10 μm.
D Representative images of transiently expressed mNG-STK38 (green), immunostained LAMP1 (magenta) and Gal3 (cyan) in HeLa cells treated with LLOMe (1 mM for 1 h, then incubated for 3 h after washout). Scale bars: 10 μm.
E Representative immunoblot of phosphorylated STK38 T444 (pT444) in HeLa cells. Cells were treated with LLOMe (1 mM for 30 min). Asterisks in STK38 and STK38 pT444 blots represent STK38L and STK38L pT442, respectively.
F Quantification of phosphorylation of STK38 T444 shown in (E).

Data information: All data presented as means ± SD, from $n \geq 3$ independent experiments. P-values were determined using one-way ANOVA with Dunnett's multiple comparisons test (B) or the unpaired t-test (F).

 © 2023 The Authors

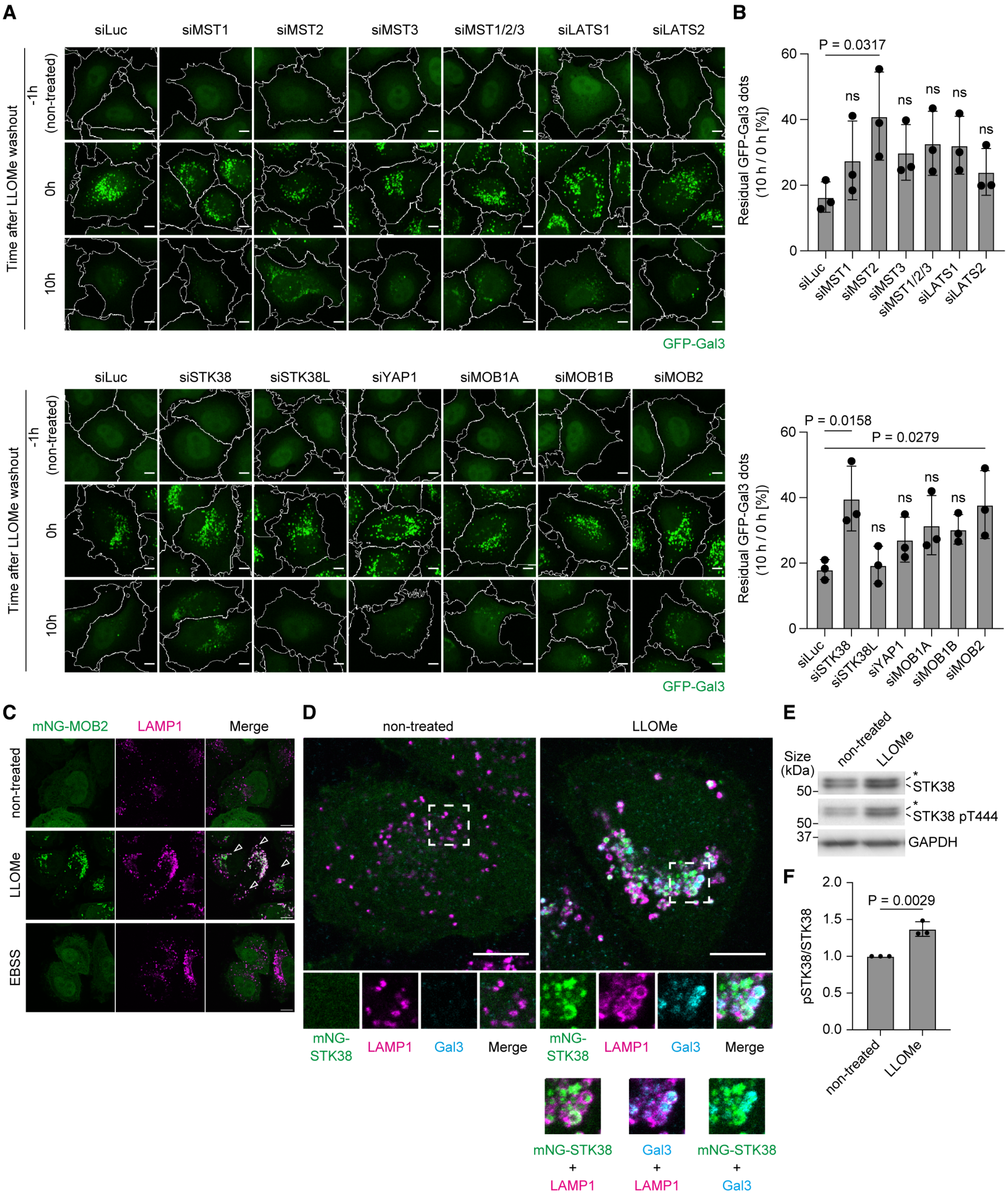
A
siLuc
siMST1
siMST2
siMST3
siMST1/2/3
siLATS1
siLATS2
Time after LLOMe washout
-1h (non-treated)
0h
10h
GFP-Gal3
siLuc
siSTK38
siSTK38L
siYAP1
siMOB1A
siMOB1B
siMOB2
B
Residual GFP-Gal3 dots (10 h / 0 h [%])
P = 0.0317
ns
P = 0.0158
P = 0.0279
C
mNG-MOB2
LAMP1
Merge
non-treated
LLOMe
EBSS
D
non-treated
LLOMe
mNG-STK38
Gal3
mNG-STK38 + LAMP1
Gal3 + LAMP1
mNG-STK38 + Gal3
E
Size (kDa)
STK38
STK38 pT444
GAPDH
F
pSTK38/STK38
P = 0.0029

Figure EV1.

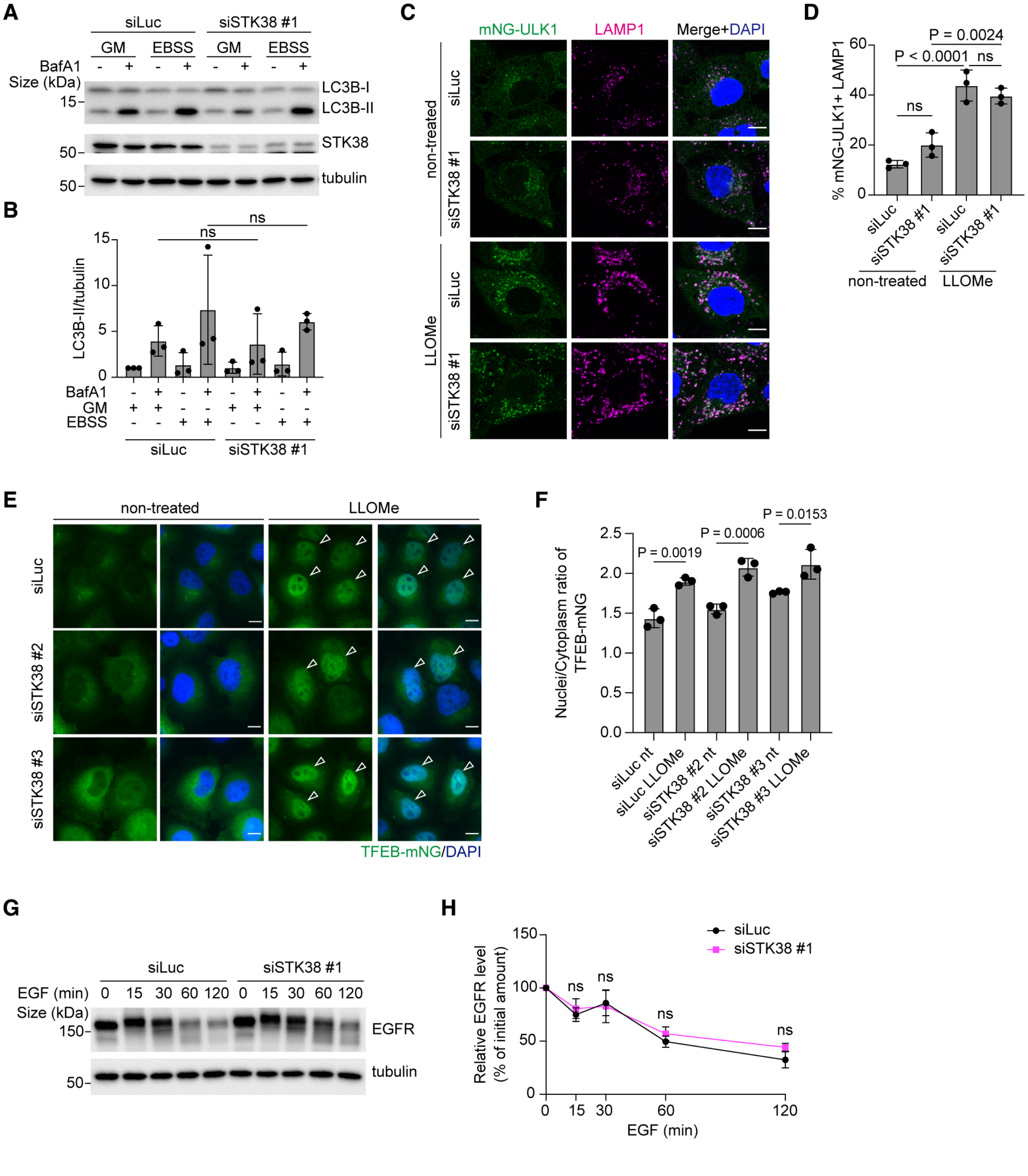

Figure EV2.

 © 2023 The Authors

Figure EV2. STK38 is not required for macroautophagy, TFEB activation, and EGF-stimulated MVB formation.

A Representative immunoblots of LC3B in siSTK38-transfected U2OS cells. Cells were incubated in normal growth media (GM) or EBSS with or without bafilomycinA1 (BafA1) for 2 h.
B Quantification of LC3B shown in (A).
C Representative images of stably expressed mNG-ULK1 (green), LAMP1 (magenta), and DAPI (blue) in siSTK38-transfected HeLa cells. Cells were treated with LLOMe (1 mM for 1 h, then incubated for 3 h after washout). Scale bars: 10 μm.
D Quantification of co-localization between mNG-ULK1 and LAMP1 shown in (C). ≥ 50 cells were analyzed per experiment for each condition.
E Representative images of stably expressed TFEB-mNG (green) and DAPI (blue) in siSTK38-transfected HeLa cells. Cells were treated with LLOMe (1 mM for 1 h, then incubated for 3 h after washout). Scale bars: 10 μm.
F The nucleus/cytoplasm ratio of TFEB-mNG in non-treated (nt) or LLOMe treated cells shown in (E). ≥ 80 cells were analyzed per experiment for each condition.
G Representative immunoblot of EGFR in siSTK38-transfected HeLa cells. Cells were treated with EGF (50 ng/ml for the indicated number of minutes).
H Quantification of EGFR shown in (G).

Data information: All data presented as means ± SD, from $n \geq 3$ independent experiments. P-values were determined using the unpaired t-test (B and H) or one-way ANOVA with Tukey's multiple comparisons test (D and F).
Source data are available online for this figure.

Figure EV3. ATG8 lipidation–dependent degradation of TRPML1 by microautophagy is occurred in endolysosomes in response to lysosomal stress or damage stimulation.

A Representative immunoblots of EGFP-TRPML1 in WT, ATG5 KO, and ATG13 KO MCF10A cells. Cells were treated with monensin (10 μM), nigericin (5 μM), or LLOMe (0.5 mM) for 8 h.
B Quantification of cleaved EGFP shown in (A).
C Representative images of macroautophagy-related factors (LC3, Gal3, ubiquitin-K48 chain (Ub-K48), and p62, shown in green), LAMP1 (magenta), and DAPI (blue) in MCF10A cells. Cells were treated with LLOMe (0.5 mM for 1 h), nigericin (5 μM for 2 h), monensin (10 μM for 2 h), or NH_4Cl (5 mM for 2 h). Scale bars: 10 μm.
D Representative images of stably expressed EGFP-TRPML1 (green), immunostained LAMP1 (magenta), and DAPI (blue) in MCF10A cells. Cells were treated with monensin (50 μM) for 1 h, followed by the co-treatment with Apilimod (200 nM) for 2 h. Scale bars: 10 μm.
E Representative images of stably expressed EGFP-TRPML1 (green), immunostained CD63 (magenta), and DAPI (blue) in MCF10A cells. Cells were treated with monensin (50 μM) for 1 h, followed by the co-treatment with Apilimod (200 nM) for 2 h. Scale bars: 10 μm.

Data information: All data presented as means ± SD, from $n \geq 3$ independent experiments. P-values were determined using one-way ANOVA with Tukey's multiple comparisons test.

© 2023 The Authors

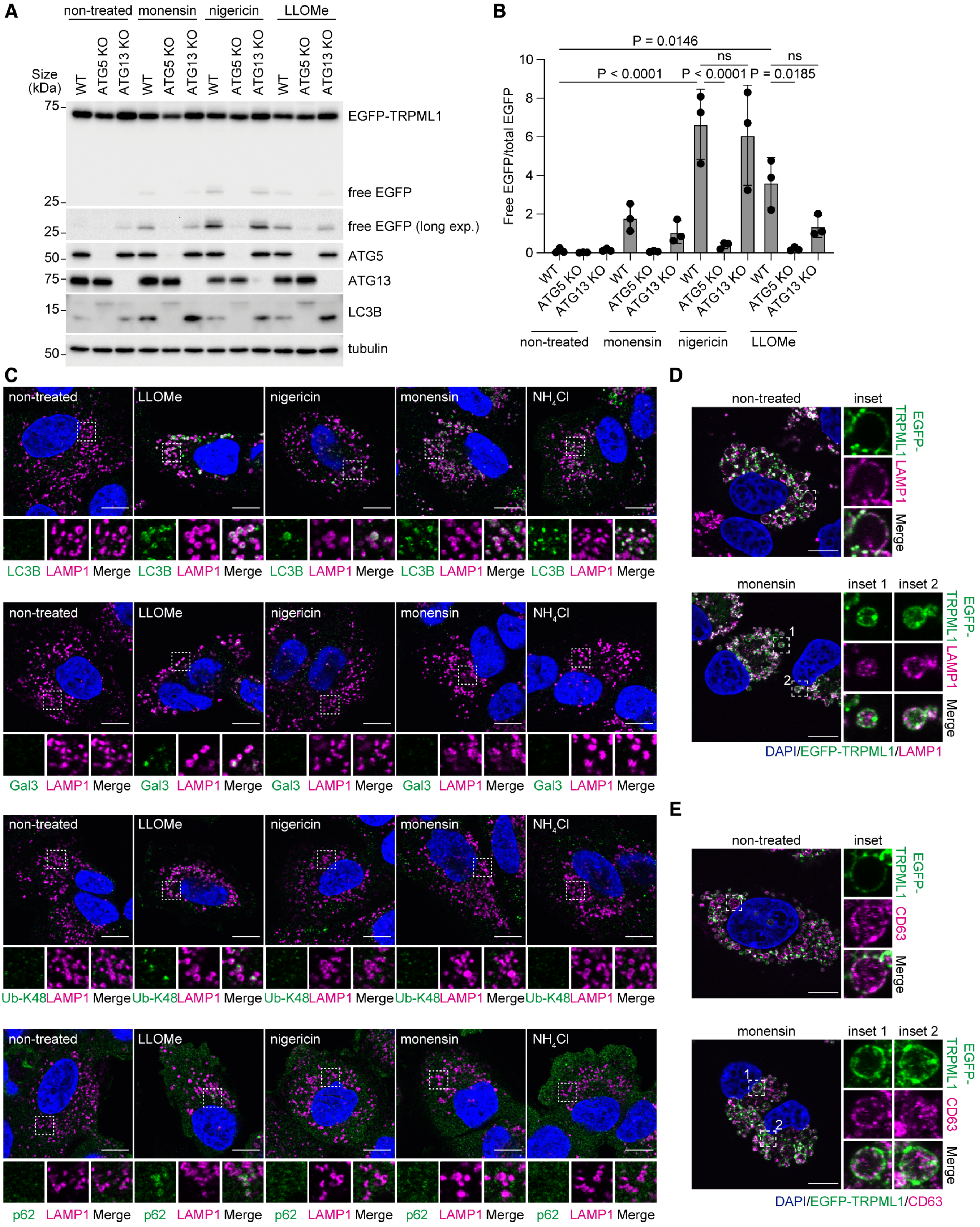

Figure EV3.

 © 2023 The Authors

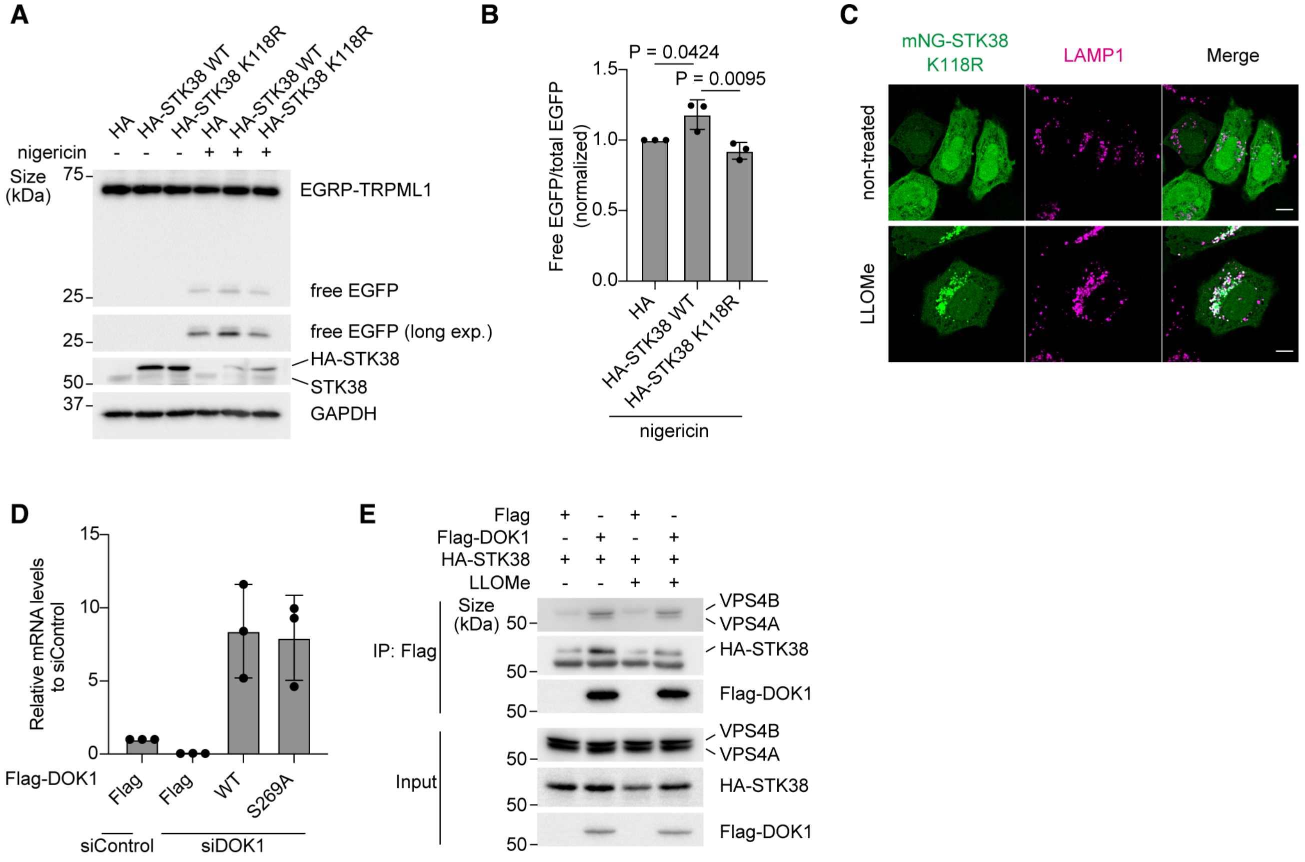

Figure EV4. STK38 kinase activity is essential for microlysophagy.

A Representative immunoblots of EGFP-TRPML1 in MCF10A cells expressing HA tag only, WT HA-STK38, or kinase-dead HA-STK38 (K118R). Cells were treated with nigericin (50 μM for 8 h).

B Quantification of cleaved EGFP shown in (A).

C Representative images of transiently expressed kinase-dead K118R mutant of mNG-STK38 (green) and immunostained LAMP1 (magenta) in HeLa cells treated with LLOMe (1 mM for 1 h, then incubated for 3 h after washout). Scale bars: 10 μm.

D Relative expression levels of *DOK1* mRNA in DOK1-reconstituted HeLa cells shown in Fig 5C and D.

E Representative immunoblots from a co-immunoprecipitation experiment. HeLa cells expressing Flag-DOK1 were treated with LLOMe (1 mM for 30 min). Cell lysates were immunoprecipitated using anti-Flag-beads agarose.

Data information: All data presented as means ± SD, from $n \geq 3$ independent experiments. *P*-values were determined using one-way ANOVA with Tukey's multiple comparisons test (B).
Source data are available online for this figure.

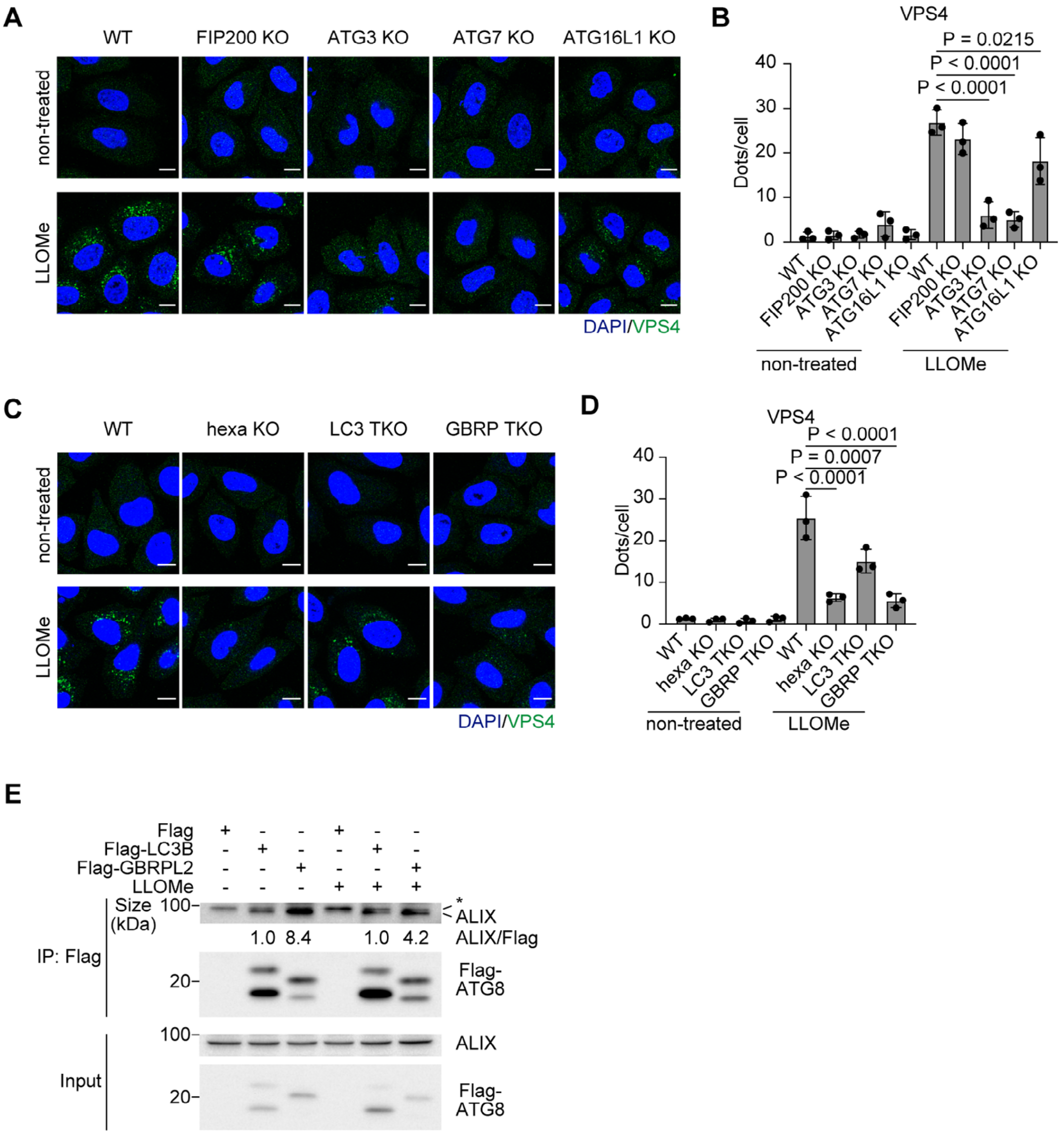

Figure EV5. Among ATG8s, GABARAPs interact preferentially with ALIX and contribute to subsequent VPS4 recruitment to damaged lysosomes.

A Representative images of immunostained VPS4 (green) and DAPI (blue) in WT, FIP200, ATG3, ATG7, and ATG16L1 KO HeLa cells. Cells were treated with LLOMe (1 mM for 30 min). Scale bars: 10 μm.

B Quantification of VPS4 dots shown in (A). ≥ 100 cells were analyzed per experiment for each condition.

C Representative images of immunostained VPS4 (green) and DAPI (blue) in WT, ATG8 hexa KO, LC3 TKO, and GABARAP TKO HeLa cells. Cells were treated with LLOMe (1 mM for 30 min). Scale bars: 10 μm.

D Quantification of VPS4 dots shown in (C). ≥ 100 cells were analyzed per experiment for each condition.

E Representative immunoblots from a co-immunoprecipitation experiment. Flag-LC3B- or GABARAPL2-expressing hexa KO HeLa cells were treated with LLOMe (1 mM for 30 min). Cell lysates were immunoprecipitated using anti-Flag-beads agarose. Fold changes in co-immunoprecipitated ALIX were quantified (Fold change vs. non-treated Flag-LC3B, normalized to Flag). The asterisk in ALIX blot represents a non-specific band.

Data information: All data presented as means ± SD, from $n \geq 3$ independent experiments. *P*-values were determined using one-way ANOVA with Tukey's multiple comparisons test.
Source data are available online for this figure.

 © 2023 The Authors

Table of contents

Page	Content
2	**Appendix Figure S1.** Knockdown efficiency of siRNAs of Hippo pathway components.
3	**Appendix Figure S2.** STK38 is recruited to lysosomes in macroautophagy independent manner in response to lysosomal damage.
5	**Appendix Figure S3.** Recruitment of ESCRTs in STK38 KO cells.
7	**Appendix Figure S4.** Depletion of STK38 does not affect basal lysosomal integrity.
8	**Appendix Figure S5.** Knockdown efficiency of siRNAs of ESCRT components.
9	**Appendix Figure S6.** Localization of 10 candidates of STK38 substrate in LLOMe treated cells.
10	**Appendix Figure S7.** Quantification of expression levels of senescent markers in non-induced cells**.**
11	**Appendix Table S1.** siRNAs used in this study.
13	**Appendix Table S2.** qPCR primers used in this study.

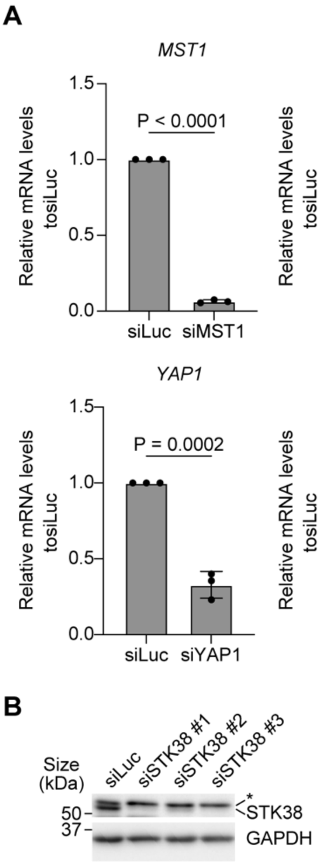

Appendix Figure S1. Knockdown efficiency of siRNAs of Hippo pathway components.

A. Relative expression levels of indicated mRNAs in siRNA-transfected HeLa cells.

B. Representative immunoblots of STK38 in siSTK38-transfected HeLa cells. The asterisk in STK38 blot represents STK38L.

Data information: All data presented as means ± SD, from $n \geq 3$ independent experiments. P-values were determined using the unpaired t-test.

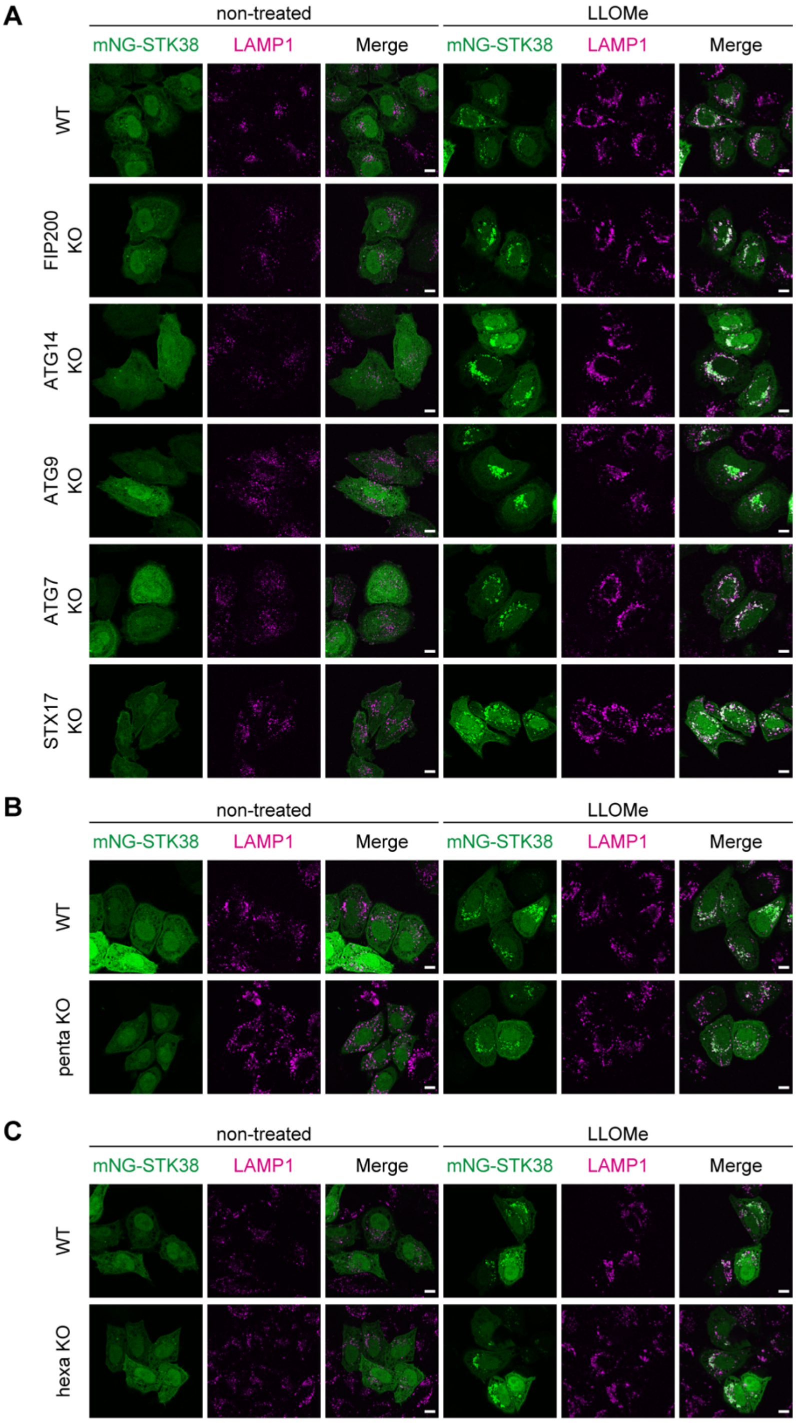

Appendix Figure S2. STK38 is recruited to lysosomes in macroautophagy independent manner in response to lysosomal damage.

A. Representative images of transiently expressed mNG-STK38 (green) and immunostained LAMP1 (magenta) in WT or macroautophagy-deficient FIP200, ATG14, ATG9, ATG7, and STX17 KO HeLa cells. Cells were treated with LLOMe (1 mM for 1 h, then incubated for 3 h after washout). Scale bars: 10 µm.

B. Representative images of transiently expressed mNG-STK38 (green) and immunostained LAMP1 (magenta) in WT or autophagy receptor penta KO HeLa cells. Cells were treated with LLOMe (1 mM for 1 h, then incubated for 3 h after washout). Scale bars: 10 µm.

C. Representative images of transiently expressed mNG-STK38 (green) and immunostained LAMP1 (magenta) in WT or ATG8 hexa KO HeLa cells. Cells were treated with LLOMe (1 mM for 1 h, then incubated for 3 h after washout). Scale bars: 10 µm.

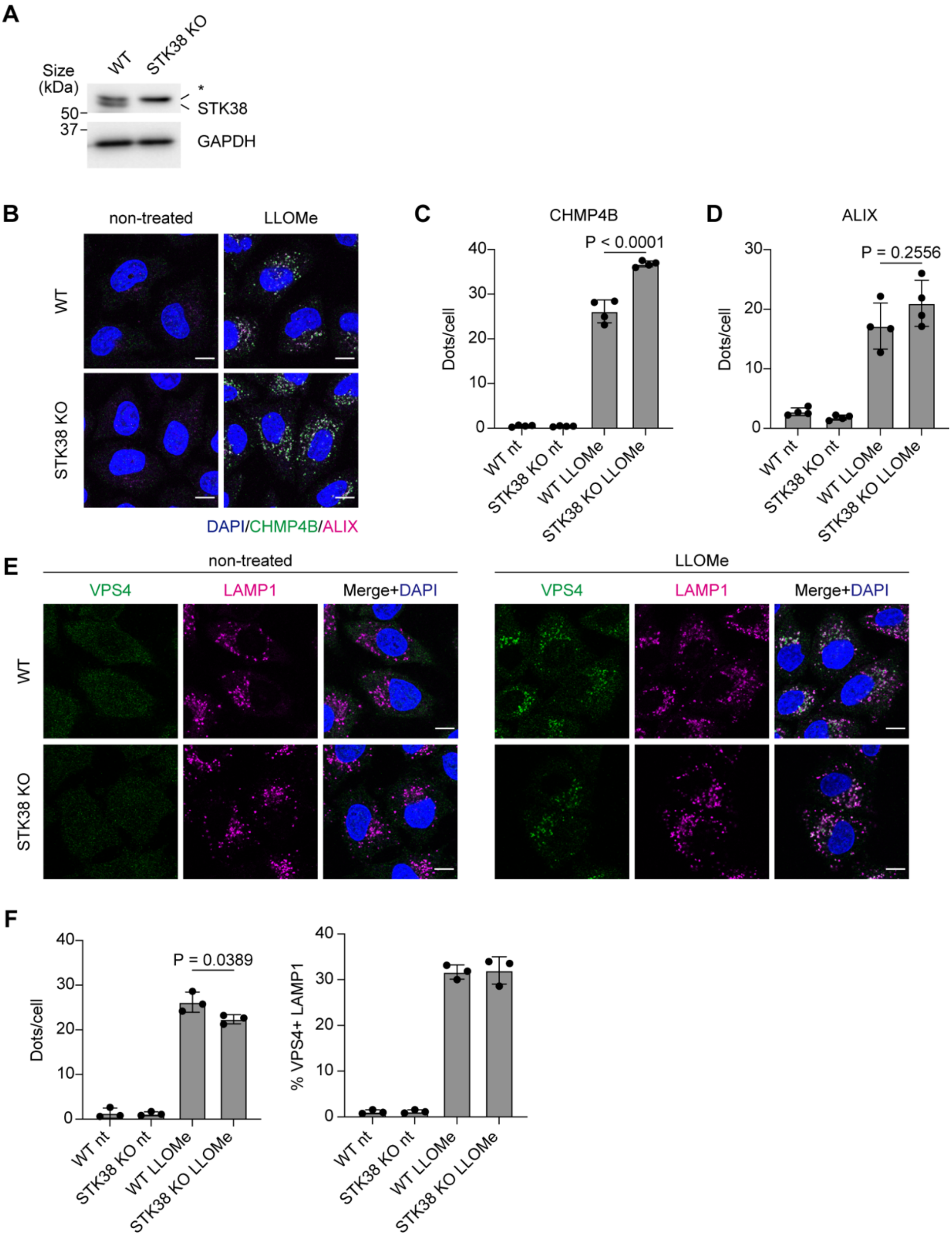

Appendix Figure S3. Recruitment of ESCRTs in STK38 KO cells.

A. Representative immunoblots of STK38 in WT or STK38 KO cells. The asterisk in STK38 blot represents STK38L.

B. Representative images of immunostained CHMP4B (green), ALIX (magenta), and DAPI (blue) in WT or STK38 KO HeLa cells. Cells were treated with LLOMe (1 mM for 30 min). Scale bars: 10 µm.

C. Quantification of CHMP4B dots shown in (B). ≥ 100 cells were analyzed per experiment for each condition.

D. Quantification of ALIX dots shown in (B). ≥ 100 cells were analyzed per experiment for each condition.

E. Representative images of immunostained VPS4 (green), LAMP1 (magenta), and DAPI (blue) in WT or

STK38 KO HeLa cells. Cells were treated with LLOMe (1 mM for 30 min). Scale bars: 10 µm.

F. Quantification of VPS4 dots (left) and co-localization between VPS4 and LAMP1 (right) shown in (E). ≥ 100 cells were analyzed per experiment for each condition.

Data information: All data presented as means ± SD, from *n* ≥ 3 independent experiments. P-values were determined using one-way ANOVA with Tukey's multiple comparisons test.

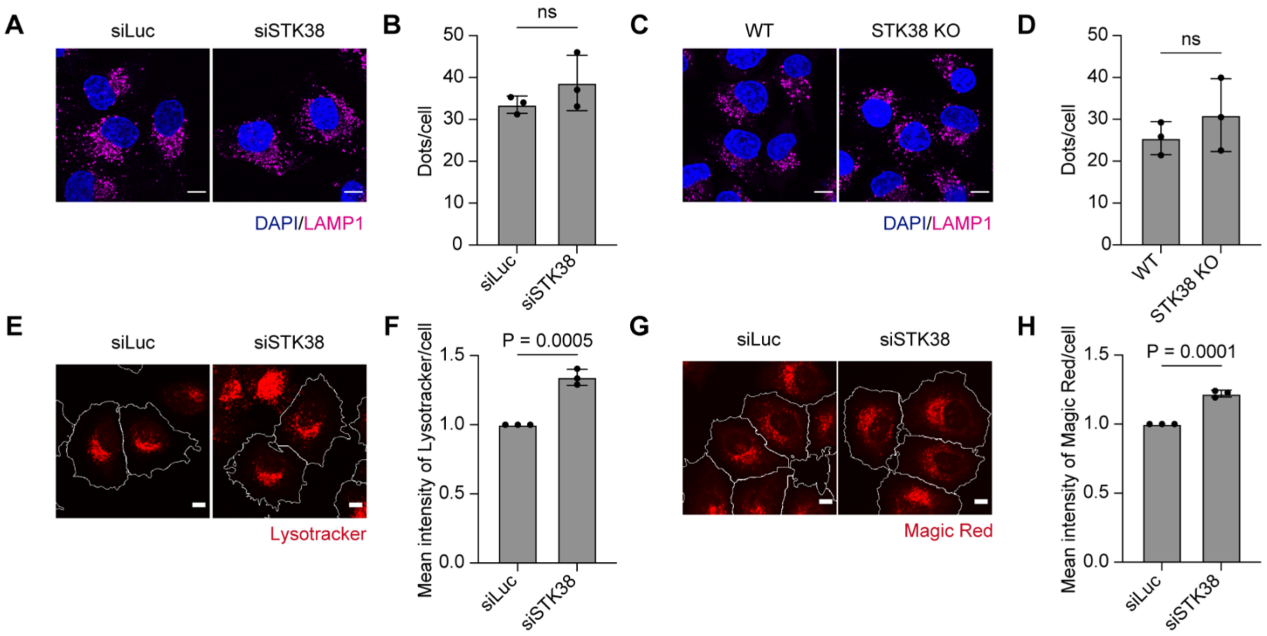

Appendix Figure S4. Depletion of STK38 does not affect basal lysosomal integrity.

A. Representative images of immunostained LAMP1 (magenta) and DAPI (blue) in siSTK38-transfected HeLa cells. Scale bars: 10 µm.

B. Quantification of LAMP1 dots shown in (A). ≥ 100 cells were analyzed per experiment for each condition.

C. Representative images of immunostained LAMP1 (magenta) and DAPI (blue) in WT or STK38 KO HeLa cells. Scale bars: 10 µm.

D. Quantification of LAMP1 dots shown in (C). ≥ 100 cells were analyzed per experiment for each condition.

E. Representative images of Lysotracker (Red) in siSTK38-transfected HeLa cells. Scale bars: 10 µm.

F. Quantification of intensity of Lysotracker per cell shown in (E). ≥ 200 cells were analyzed per experiment for each condition.

G. Representative images of Magic Red (Red) in siSTK38-transfected HeLa cells. Scale bars: 10 µm.

H. Quantification of intensity of Magic Red per cell shown in (G). ≥ 200 cells were analyzed per experiment for each condition.

Data information: All data presented as means ± SD, from $n \geq 3$ independent experiments. P-values were determined using the unpaired t-test.

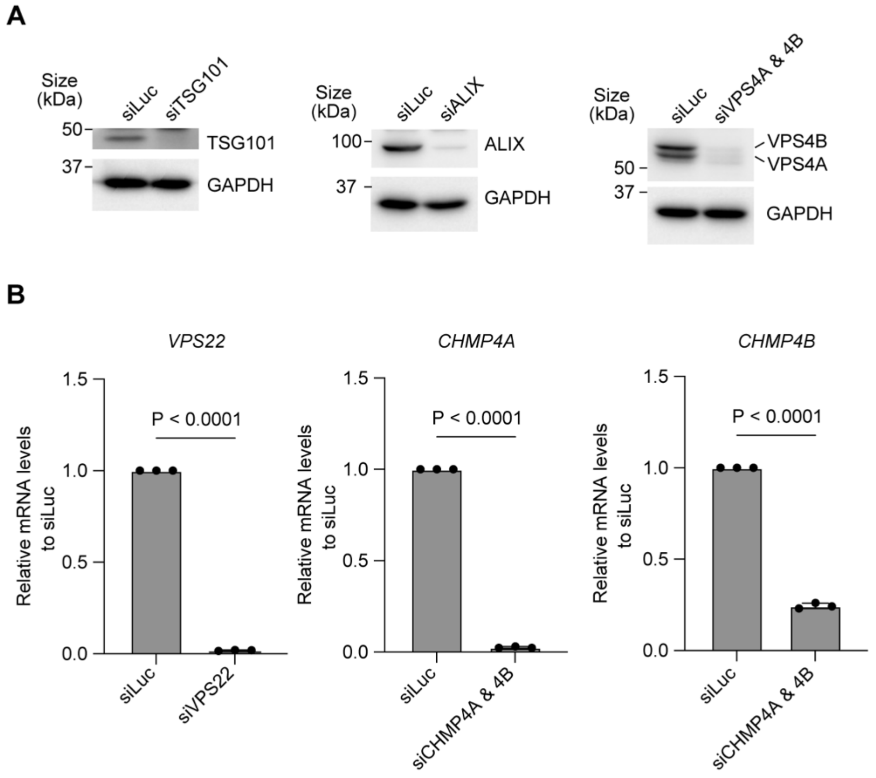

Appendix Figure S5. Knockdown efficiency of siRNAs of ESCRT components.

A. Representative immunoblots of indicated ESCRT components in siRNA-transfected MCF10A cells.

B. Relative expression levels of indicated mRNAs in siRNA-transfected MCF10A cells.

Data information: All data presented as means ± SD, from $n \geq 3$ independent experiments. P-values were determined using the unpaired t-test.

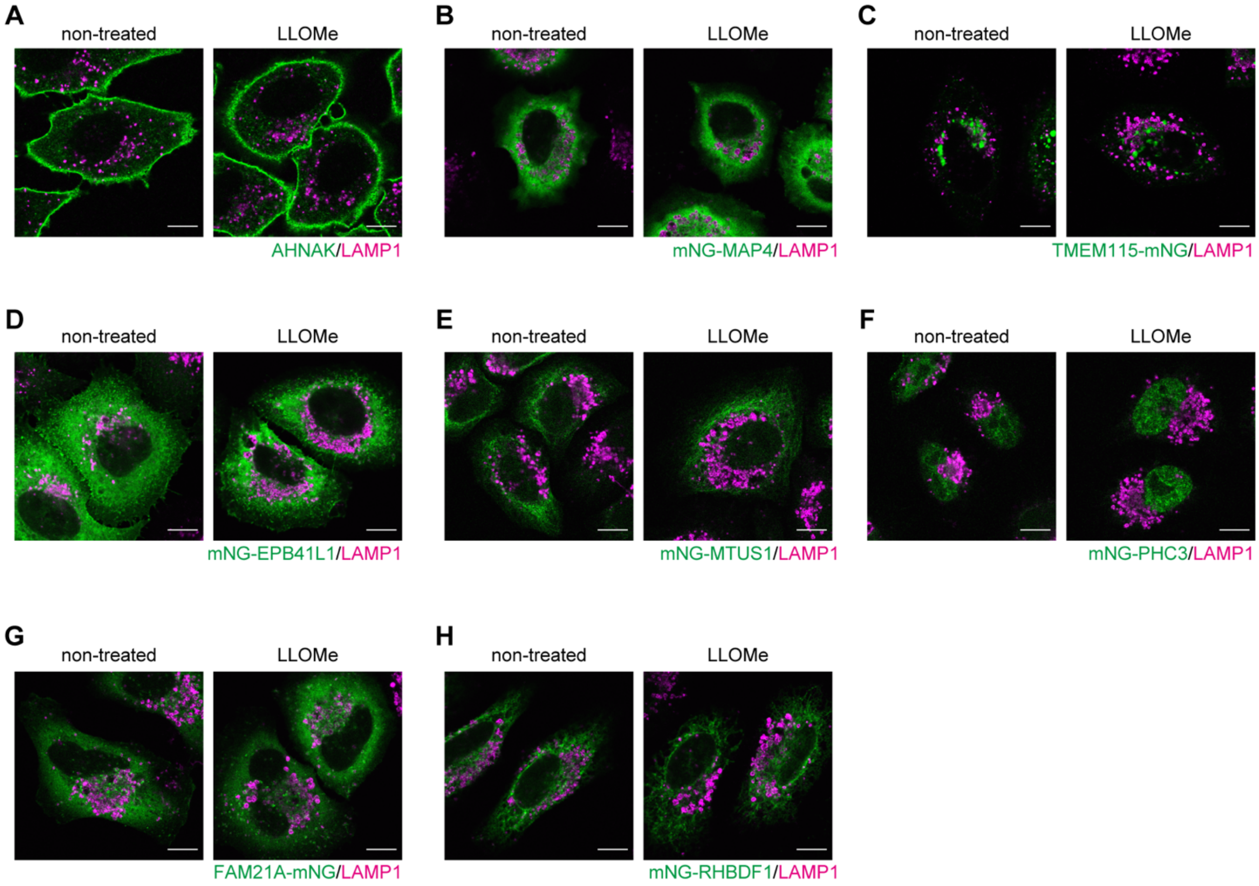

Appendix Figure S6. Localization of 10 candidates of STK38 substrate in LLOMe treated cells.

A. Representative images of immunostained AHNAK (green) and LAMP1 (magenta) in WT HeLa cells. Cells were treated with LLOMe (1 mM for 1 h). Scale bars: 10 µm.

B-H. Representative images of transiently expressed mNG-tagged candidates (green) and immunostained LAMP1 (magenta) in WT HeLa cells. Cells were treated with LLOMe (1 mM for 1 h, then incubated for 3 h after washout). Scale bars: 10 µm.

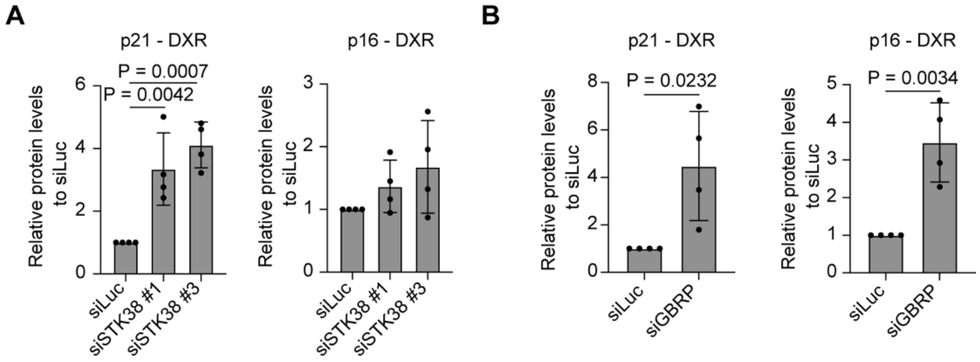

Appendix Figure S7. Quantification of expression levels of senescent markers in non-induced cells.

A. Quantification of p21 and p16 in siSTK38-transfected non-induced cells shown in Fig 7A.

B. Quantification of p21 and p16 in siGABARAP-transfected non-induced cells shown in Fig 7C.

Data information: All data presented as means ± SD, from $n \geq 3$ independent experiments. P-values were determined using one-way ANOVA with Dunnett's multiple comparisons test (A) or the unpaired t-test (B).

Appendix Table S1. siRNAs used in this study.

siRNA	Source	Identifier
siLuciferase: 5’-CGUACGCGGAAUACUUCGAdTdT-3’	Sigma-Aldrich	Custom
siMST1: 5’-GACUGAUGGAGCCAAUACUdTdT-3	Sigma-Aldrich	Custom
siMST2	Sigma-Aldrich	SASI_Hs01_00015485
siMST3	Sigma-Aldrich	SASI_Hs01_00146086
siLATS1	Sigma-Aldrich	SASI_Hs01_00046130
siLATS2	Sigma-Aldrich	SASI_Hs01_00158804
siYAP1	Sigma-Aldrich	SASI_Hs01_00182402
siSTK38 #1: 5’-CGUCGGCCAUAAACAGCUdTdT-3’	Sigma-Aldrich	Custom (Joffre *et al*, 2015)
siSTK38 #2: 5’-GUAAUAGGCAGAGGAGCAdTdT-3’	Sigma-Aldrich	Custom (Joffre *et al.*, 2015)
siSTK38 #3	Sigma-Aldrich	SASI_Hs01_00224995
siSTK38L	Sigma-Aldrich	SASI_Hs01_00074234
siMOB1A	Sigma-Aldrich	SASI_Hs01_00231852
siMOB1B	Sigma-Aldrich	SASI_Hs01_00171109
siMOB2	Sigma-Aldrich	SASI_Hs01_00177401
siTSG101: 5’-CCUCCAGUCUUCUCUCGUCdTdT-3’	Sigma-Aldrich	Custom (Skowyra *et al*, 2018)
siALIX: 5’-GAAGGAUGCUUUCGAUAAAdTdT-3’	Sigma-Aldrich	Custom
siVPS22: 5’-CUUGCAGAGGCCAAGUAUAdTdT-3’	Sigma-Aldrich	Custom (Zhang *et al*, 2021)
siCHMP4A	Sigma-Aldrich	SASI_Hs01_00039745
siCHMP4B	Sigma-Aldrich	SASI_Hs01_00129003
siVPS4A: 5’-CUGUGGUUUGCAUGUCGGAdTdT-3’	Sigma-Aldrich	Custom (Mejlvang *et al*, 2018)
siVPS4B: 5’-CCAAAGAAGCACUGAAAGAdTdT-3’	Sigma-Aldrich	Custom (Mejlvang *et al.*, 2018)
siGABARAP	Sigma-Aldrich	SASI_Hs01_00197526
siGABARAPL1	Sigma-Aldrich	SASI_Hs01_00200246
siGABARAPL2	Sigma-Aldrich	SASI_Hs01_00165606
siMAP4	Thermo Fisher Scientific	4427030; s200446

siPSMA5	Thermo Fisher Scientific	4427030; s11339
siEPHA2	Thermo Fisher Scientific	4427030; s4565
siSOS1	Thermo Fisher Scientific	4427030; s13286
siARHGAP1	Thermo Fisher Scientific	4427030; s1584
siGOLGA3	Thermo Fisher Scientific	4427030; s5946
siAHNAK	Thermo Fisher Scientific	4427030; s200548
siTMEM115	Thermo Fisher Scientific	4427030; s21818
siARHGAP21	Thermo Fisher Scientific	4427030; s33373
siFAM21A	Thermo Fisher Scientific	4427030; s51898
siRHBDF2	Thermo Fisher Scientific	4427030; s36014
siPHLDB2	Thermo Fisher Scientific	4427030; s40251
siZZZ3	Thermo Fisher Scientific	4427030; s24923
siTAB3	Thermo Fisher Scientific	4427030; s223653
siEHBP1	Thermo Fisher Scientific	4427030; s225944
siPHC3	Thermo Fisher Scientific	4427030; s36819
siGOLGA5	Thermo Fisher Scientific	4427030; s19321
siRHBDF1	Thermo Fisher Scientific	4427030; s34612
siPOM121	Thermo Fisher Scientific	4427030; s19145
siDOK1	Thermo Fisher Scientific	4427030; s4236
siEPB41L1	Thermo Fisher Scientific	4427030; s4706
siNCKAP5L	Thermo Fisher Scientific	4427030; s49193
siBCLAF1	Thermo Fisher Scientific	4427030; s225184
siMTUS1	Thermo Fisher Scientific	4427030; s33189
siBTRC	Thermo Fisher Scientific	4427030; s17111
Negative Control	Thermo Fisher Scientific	4390843

Appendix Table S2. qPCR primers used in this study.

Target	Forward (5'-3')	Reverse (5'-3')
MST1	GCCCCCTTATGCTGATATCCAT	GGGAGGAGGATTTGTAGGAATCAT
MST2	AACTGTGTGGCCGACATCTG	GAGGTTTTCCTTCAGCCATTTCT
MST3	GACATTAAAGCGGCCAACGT	CAAAGTCCGCCAGCTTCAC
LATS1	CATTCCACCACAAGCTAAACTCAGT	GGGTCCTCGGCAAAGTTTAAT
LATS2	GAATTTCGACCCCGTAGATGAA	GTGTGTCCCAGGCCTTGGT
YAP1	CCAGTGCAGCAGAATATGATGAA	GGCTTGTTCCCATCCATCAG
STK38L	CACAACACGCTCGCAAAGA	TCCAAGCCAAGTCTGGTCCTT
MOB1A	TGTCCCATTTCCCAAAAACTTT	AACCCTGAACAGACGCTTTAGAA
MOB1B	GGTTCAGGACCAGTTGGATGA	TGGGAACGGGACACCAAT
MOB2	GGAGCTGCACGGACACTTG	CTCCCGAGCAAAGAGGATGA
VPS22	ATCAGAGCCATCAAGAAACTAAAGG	CCGCCCACAGGGATGA
CHMP4A	ACAGCAGCTGGCACAAACTG	TGGCCTCACGCTGAAACTC
CHMP4B	AGGCGGCCCATGACAA	CAGCAATGTCCTGCATTAACTCA
DOK1	TGGCGCCTACCGATAACC	TGTACAAGGAGTTCTCCAGCATCT
IL-6	CCAGGAGCCCAGCTATGAAC	CCCAGGGAGAAGGCAACTG
IL-1α	AACCAGTGCTGCTGAAGGA	TTCTTAGTGCCGTGAGTTTCC
GAPDH	TGCACCACCAACTGCTTAGC	GGCATGGACTGTGGTCATGAG
