# Supplementary figures and images for: Microautophagy regulated by STK38 and GABARAPs is essential to repair lysosomes and prevent aging (part 1 of 4)
Source: EMBO Rep. 2023 Nov 21;24(12):e57300. doi: 10.15252/embr.202357300 (PMC10702834; doi:10.15252/embr.202357300)

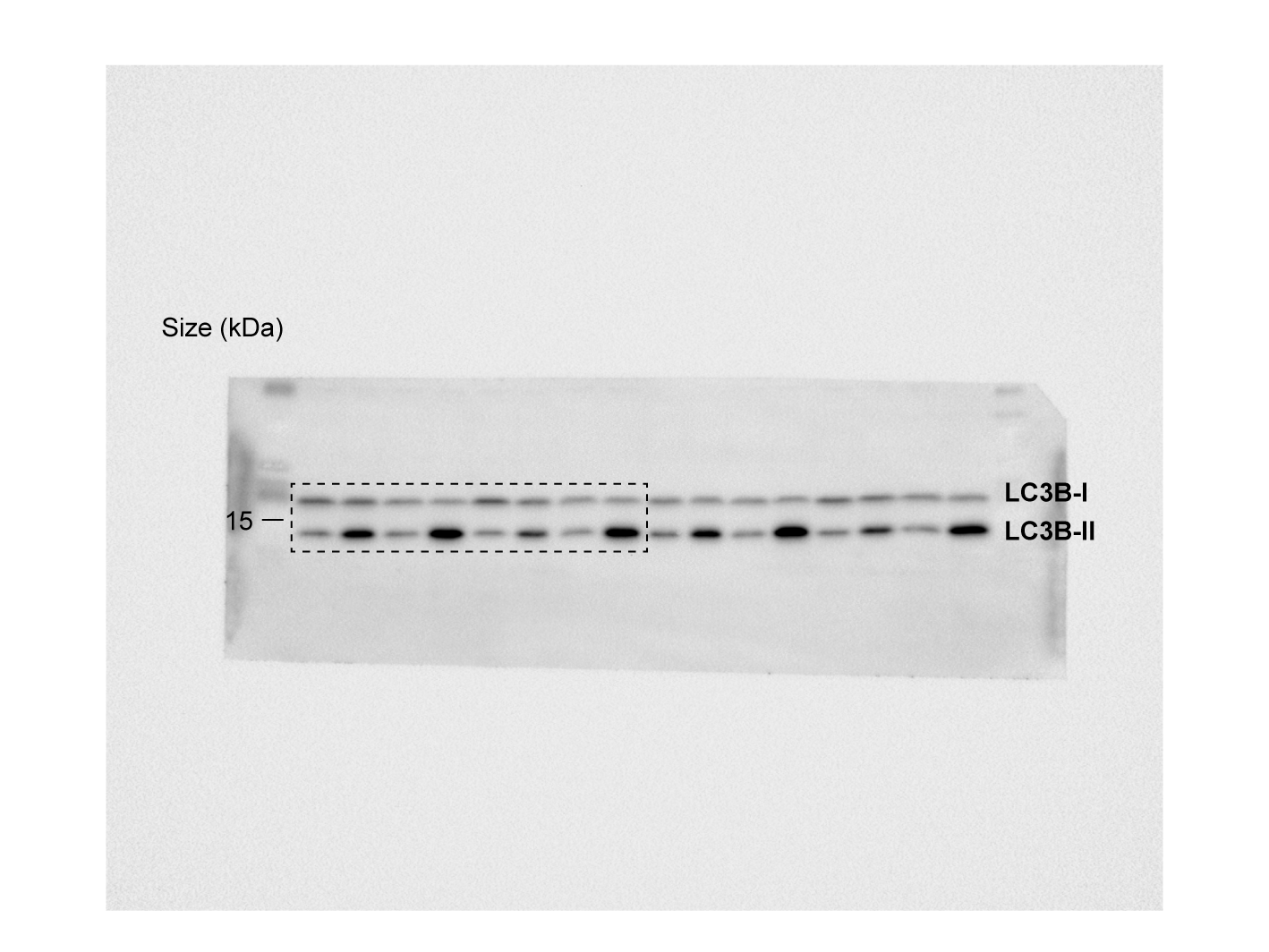

Supplement: Supplementary file 4 — Source Data for Expanded View [file EMBR-24-e57300-s011.zip › Fig EV2/EV2A/western_LC3B.tif]

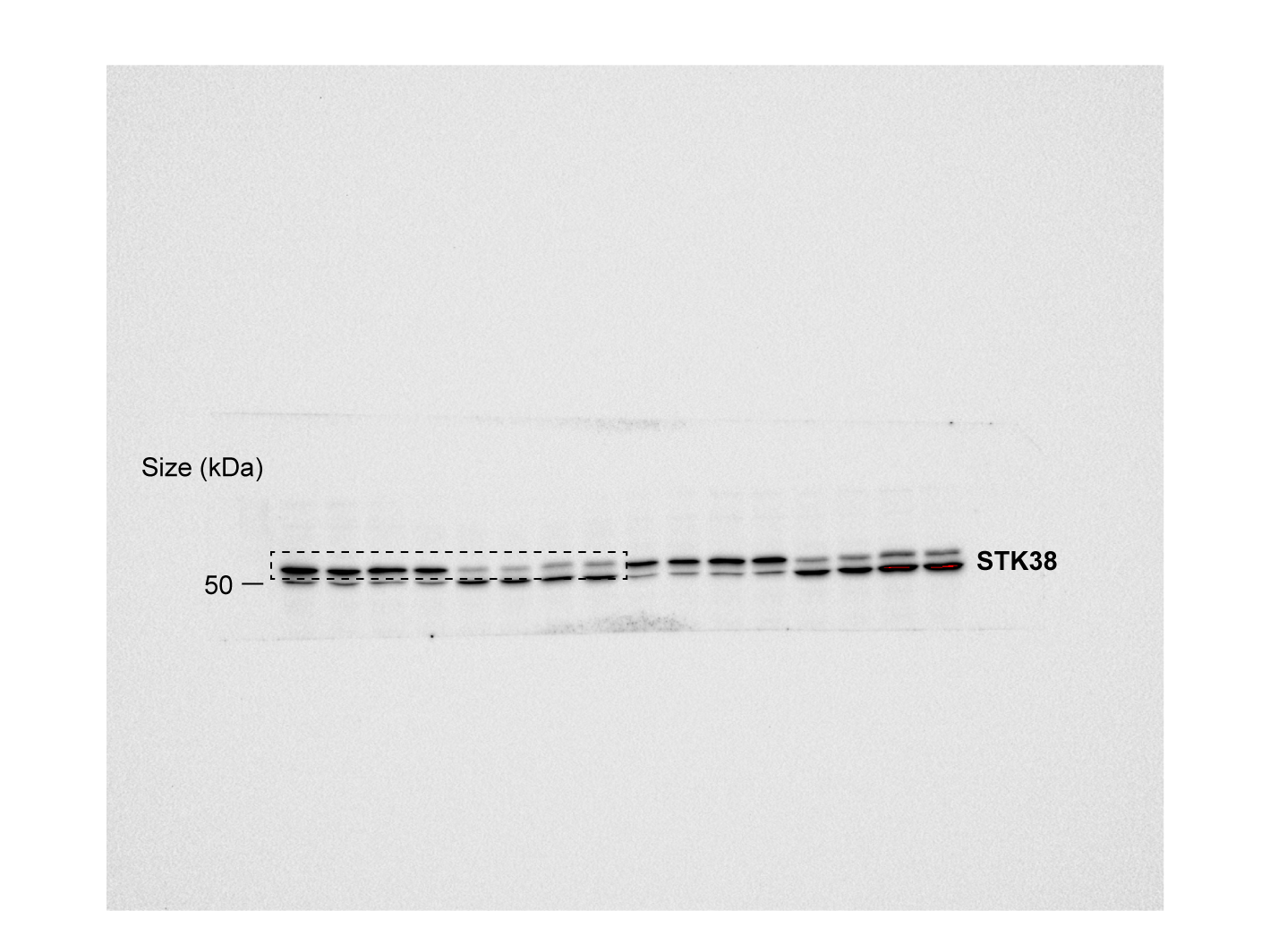

Supplement: Supplementary file 4 — Source Data for Expanded View [file EMBR-24-e57300-s011.zip › Fig EV2/EV2A/western_STK38.tif]

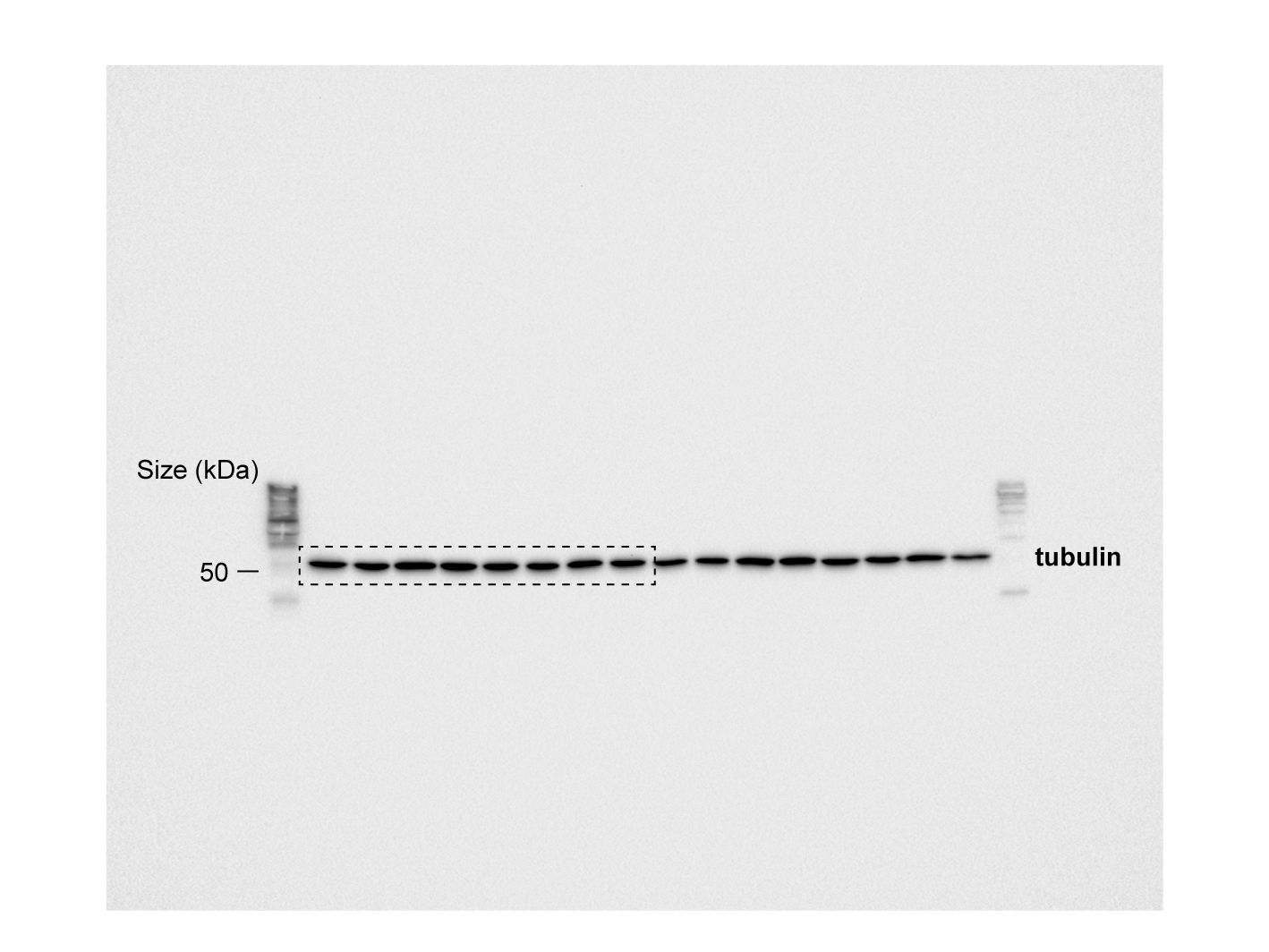

Supplement: Supplementary file 4 — Source Data for Expanded View [file EMBR-24-e57300-s011.zip › Fig EV2/EV2A/western_tubulin.tif]

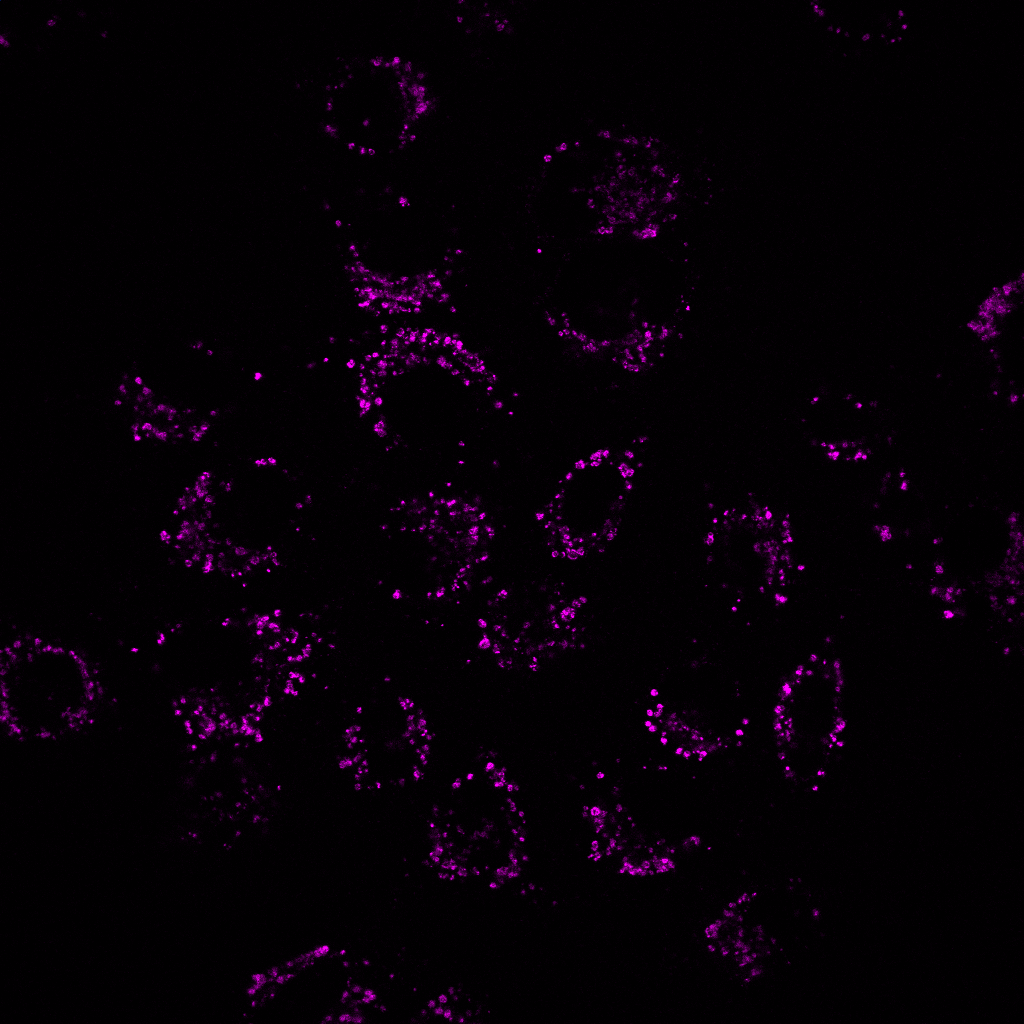

Supplement: Supplementary file 4 — Source Data for Expanded View [file EMBR-24-e57300-s011.zip › Fig EV2/EV2C/siLuc_LLOMe_LAMP1.tif]

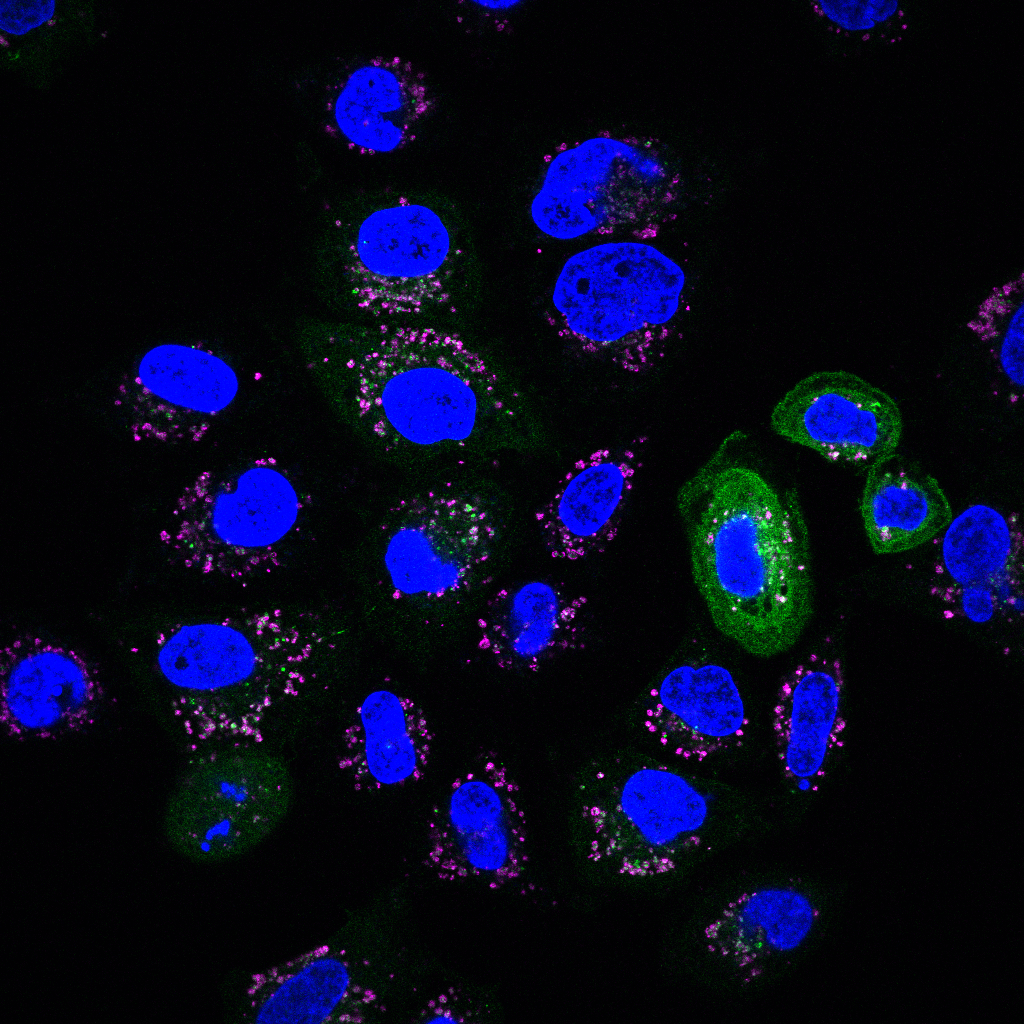

Supplement: Supplementary file 4 — Source Data for Expanded View [file EMBR-24-e57300-s011.zip › Fig EV2/EV2C/siLuc_LLOMe_Merge+DAPI.tif]

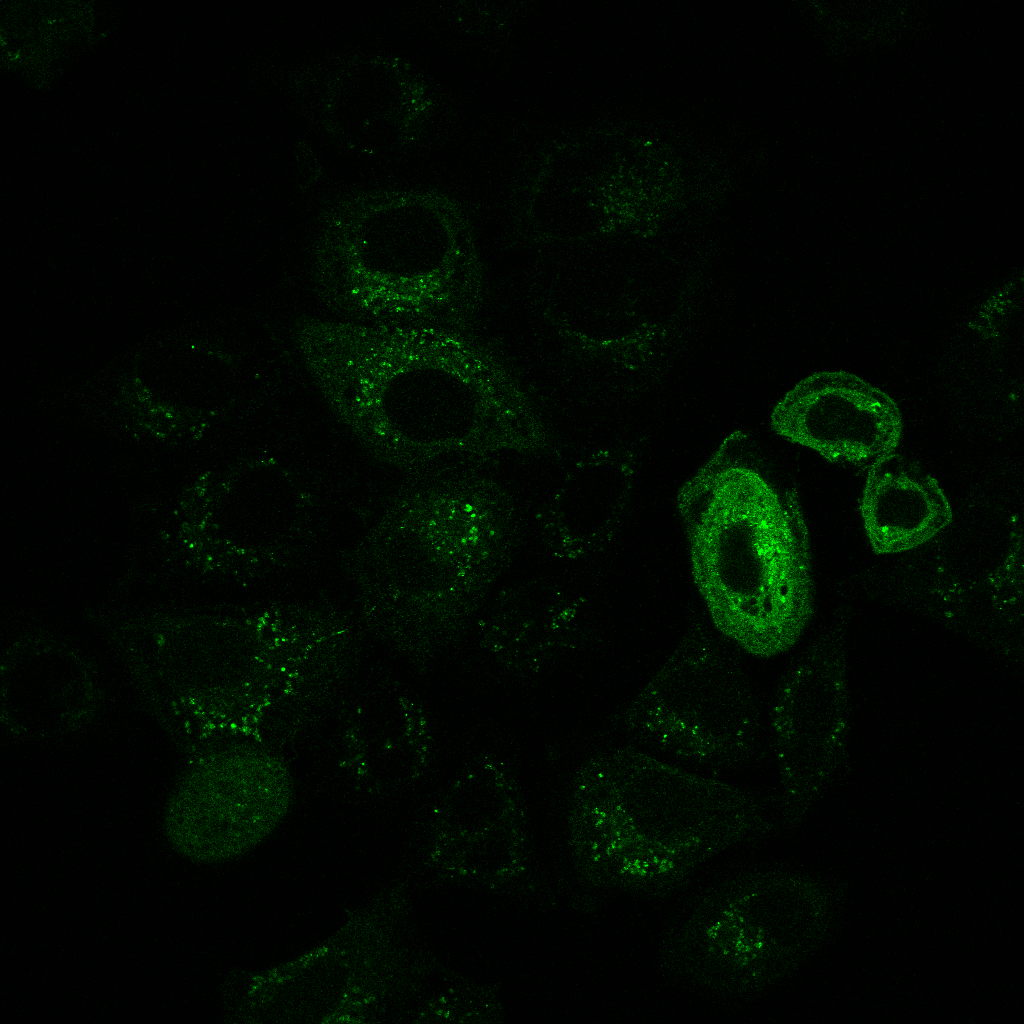

Supplement: Supplementary file 4 — Source Data for Expanded View [file EMBR-24-e57300-s011.zip › Fig EV2/EV2C/siLuc_LLOMe_mNG-ULK1.tif]

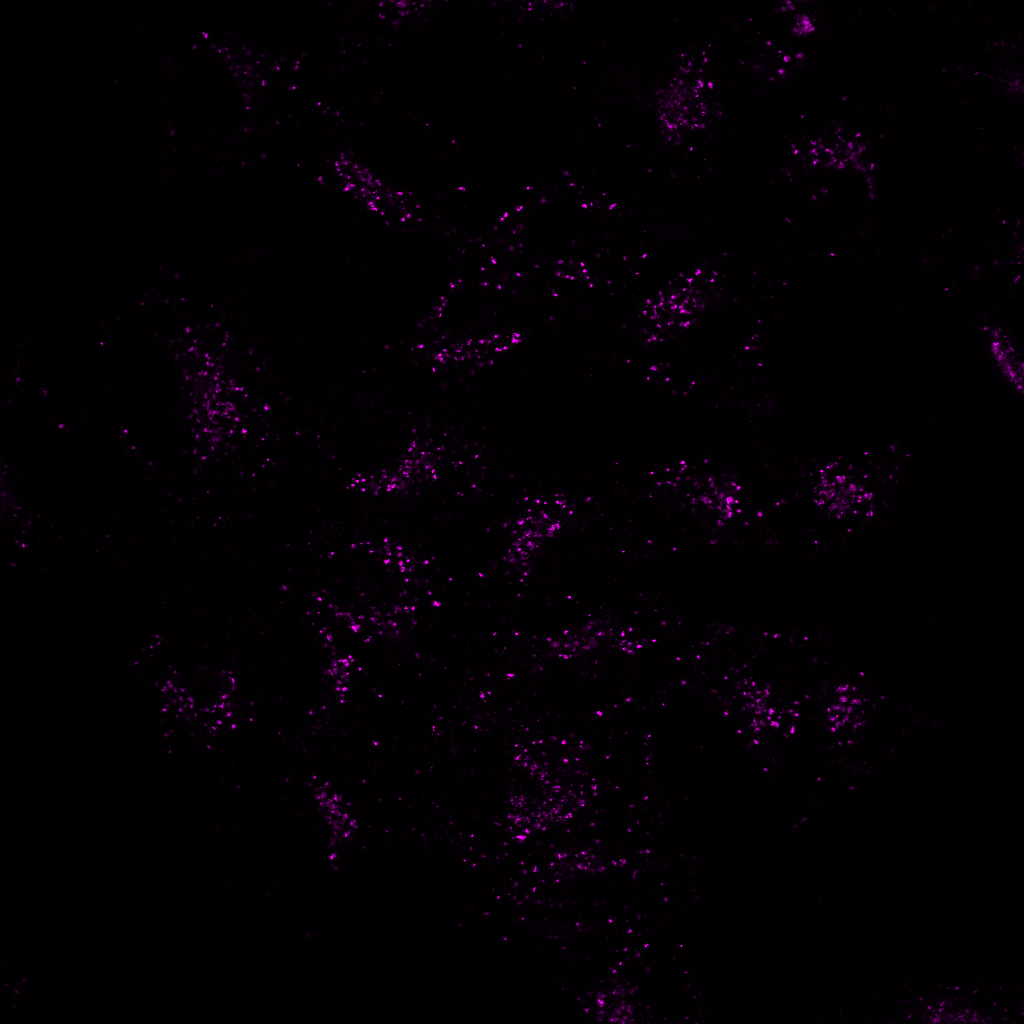

Supplement: Supplementary file 4 — Source Data for Expanded View [file EMBR-24-e57300-s011.zip › Fig EV2/EV2C/siLuc_non-treated_LAMP1.tif]

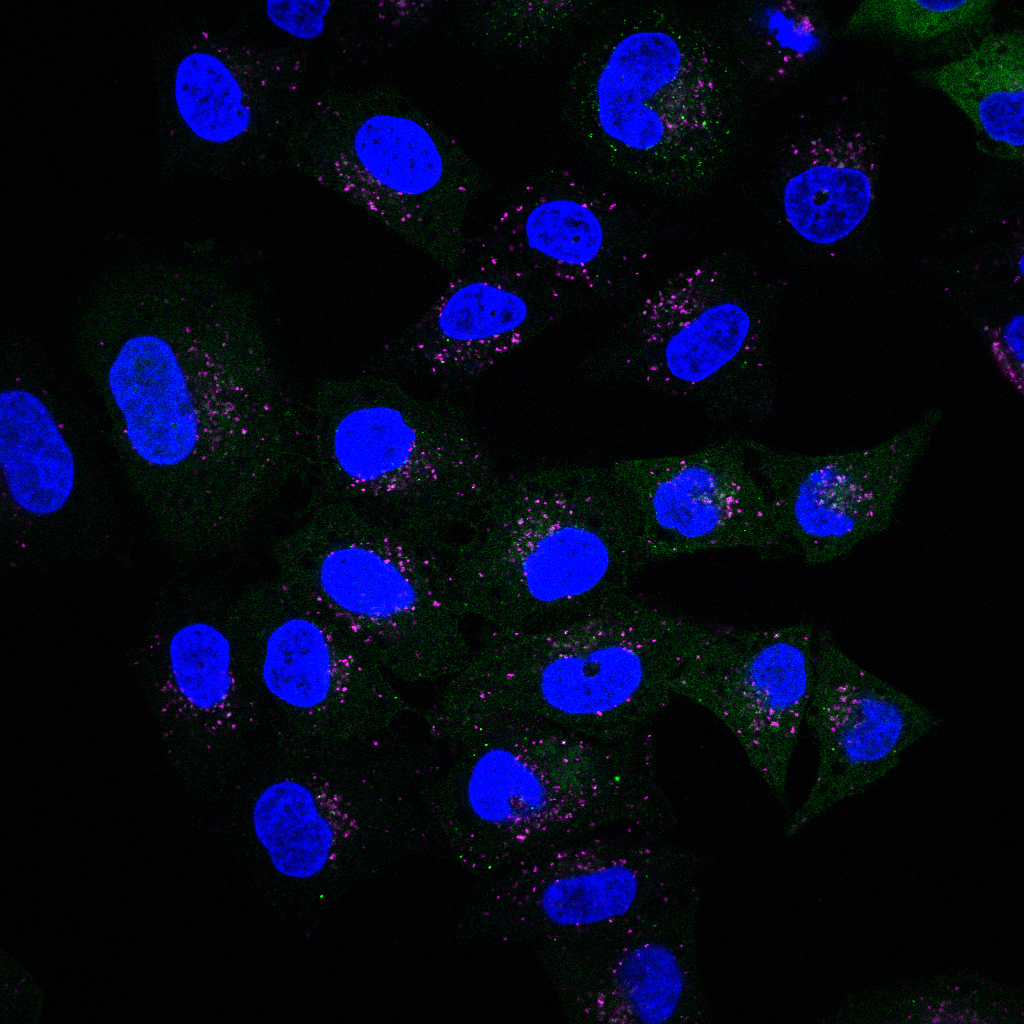

Supplement: Supplementary file 4 — Source Data for Expanded View [file EMBR-24-e57300-s011.zip › Fig EV2/EV2C/siLuc_non-treated_Merge+DAPI.tif]

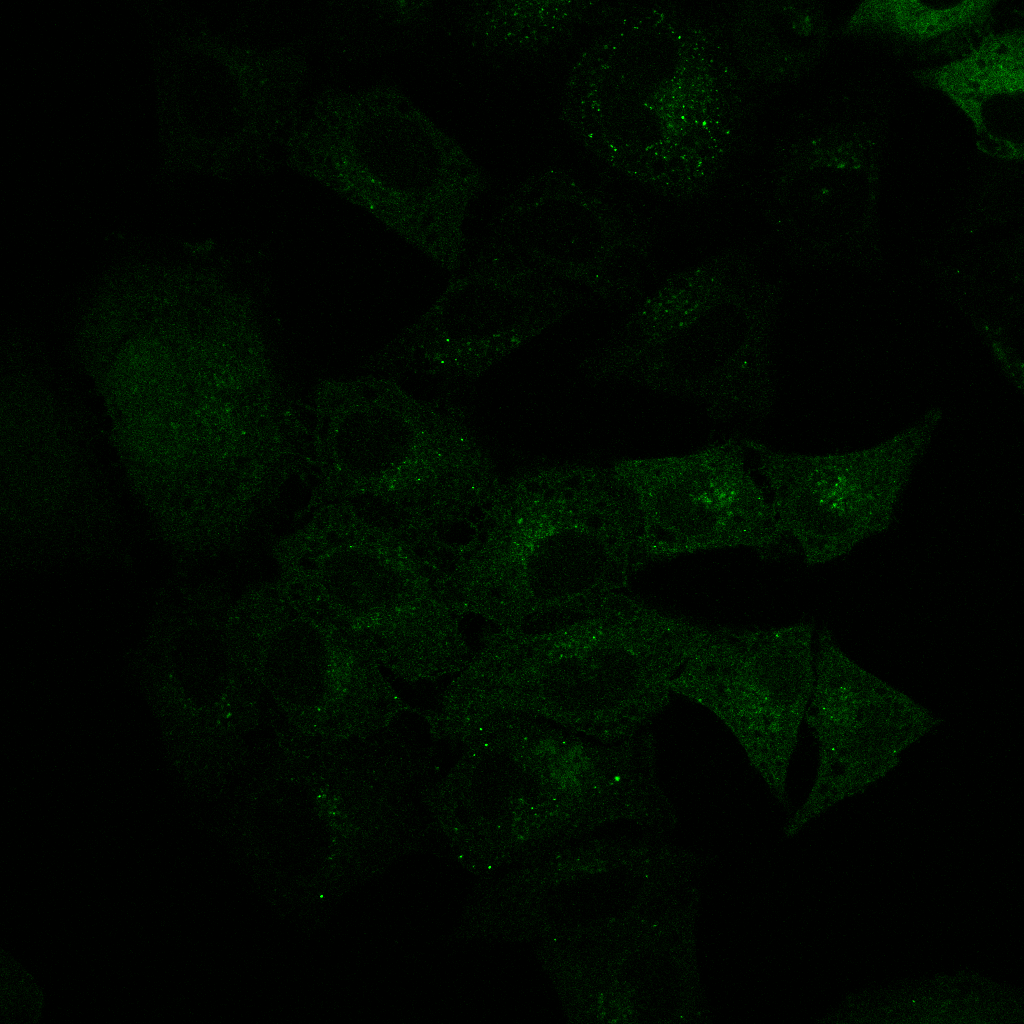

Supplement: Supplementary file 4 — Source Data for Expanded View [file EMBR-24-e57300-s011.zip › Fig EV2/EV2C/siLuc_non-treated_mNG-ULK1.tif]

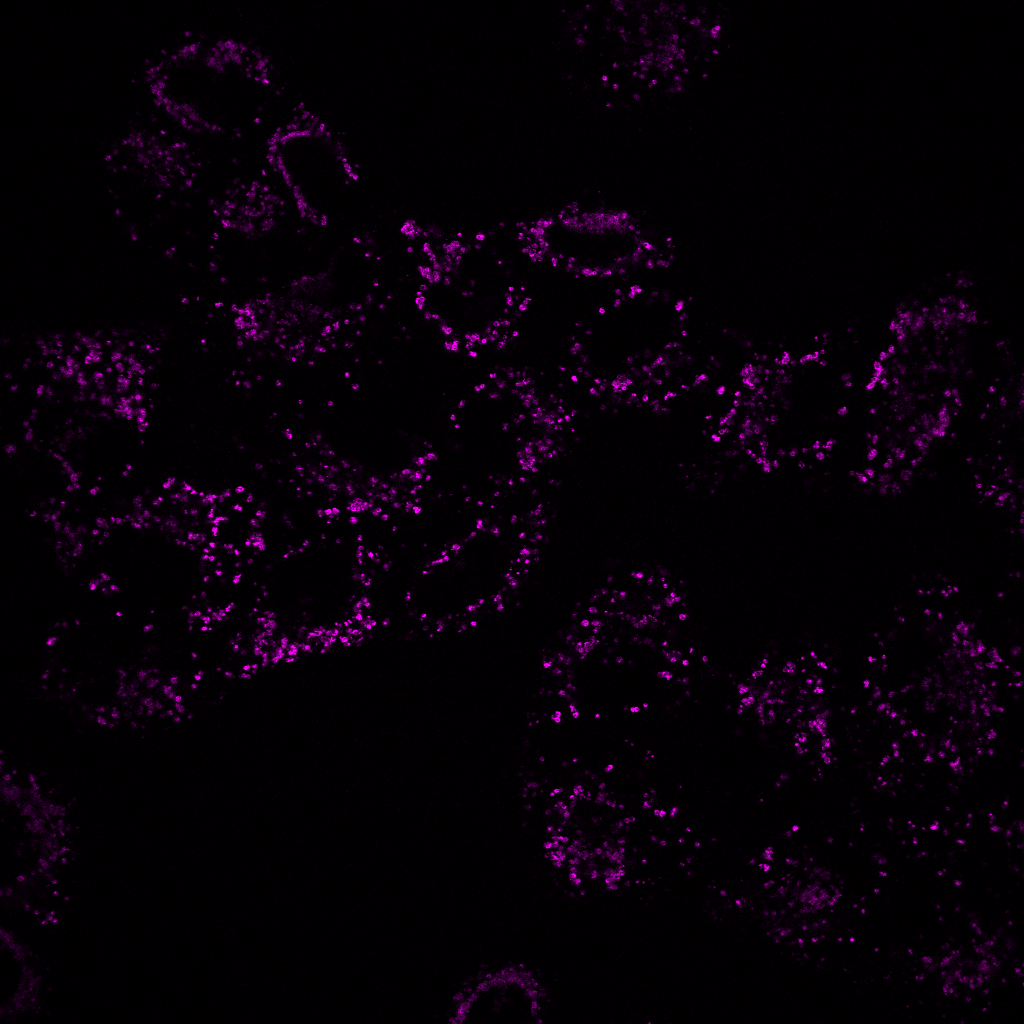

Supplement: Supplementary file 4 — Source Data for Expanded View [file EMBR-24-e57300-s011.zip › Fig EV2/EV2C/siSTK38 #1_LLOMe_LAMP1.tif]

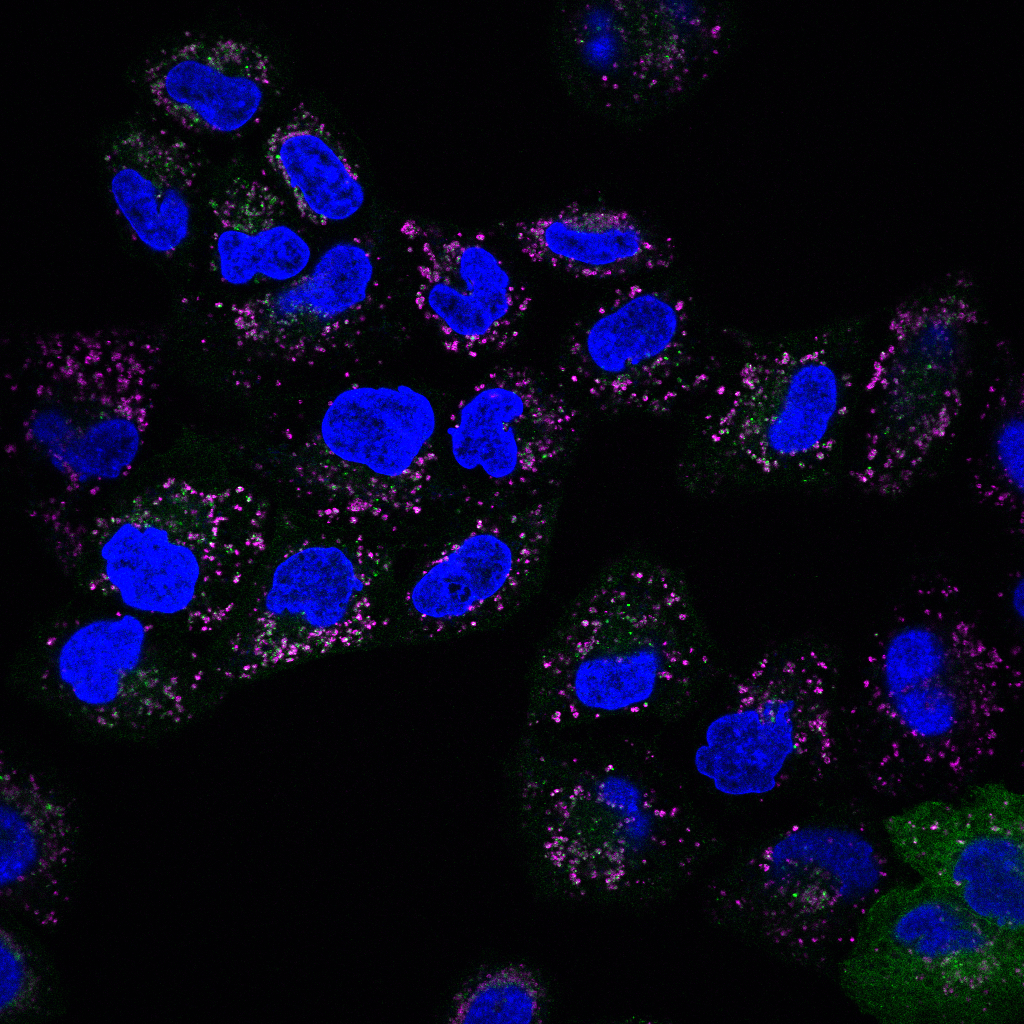

Supplement: Supplementary file 4 — Source Data for Expanded View [file EMBR-24-e57300-s011.zip › Fig EV2/EV2C/siSTK38 #1_LLOMe_Merge+DAPI.tif]

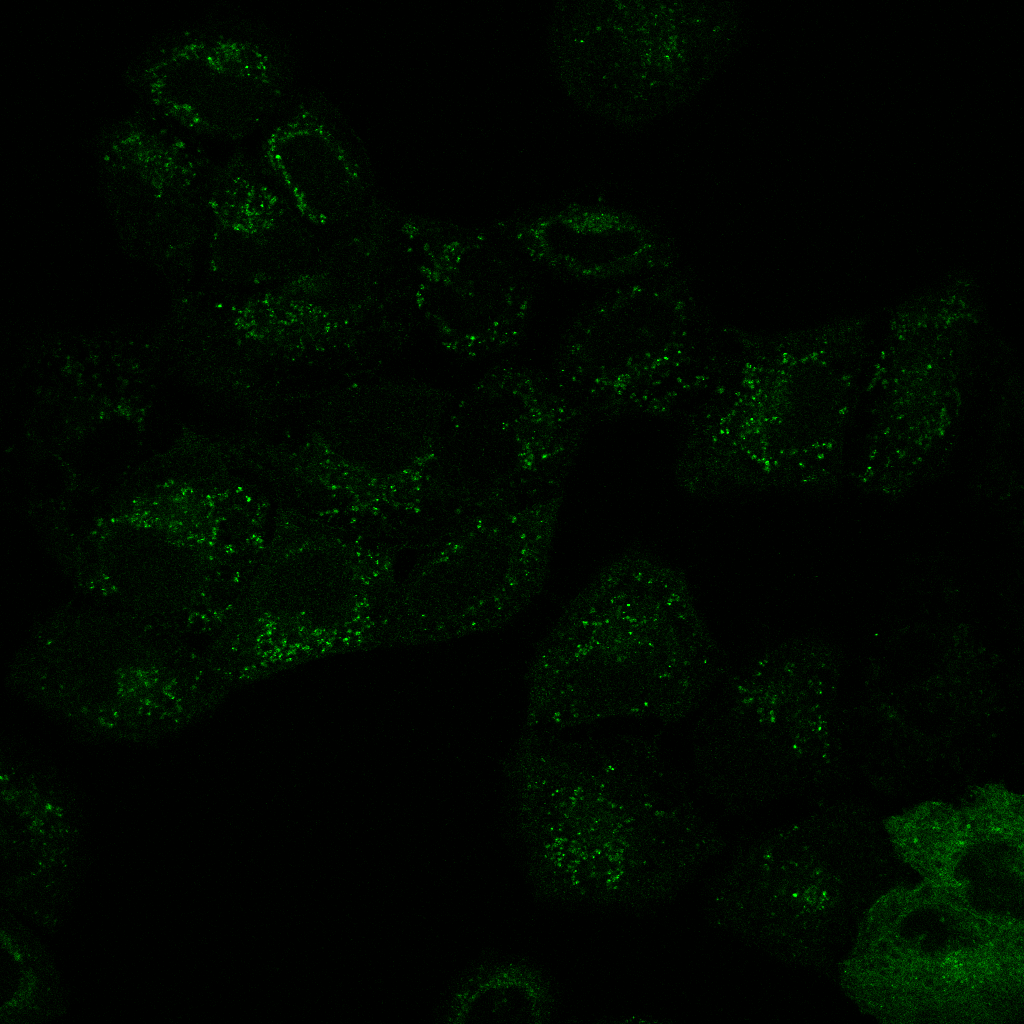

Supplement: Supplementary file 4 — Source Data for Expanded View [file EMBR-24-e57300-s011.zip › Fig EV2/EV2C/siSTK38 #1_LLOMe_mNG-ULK1.tif]

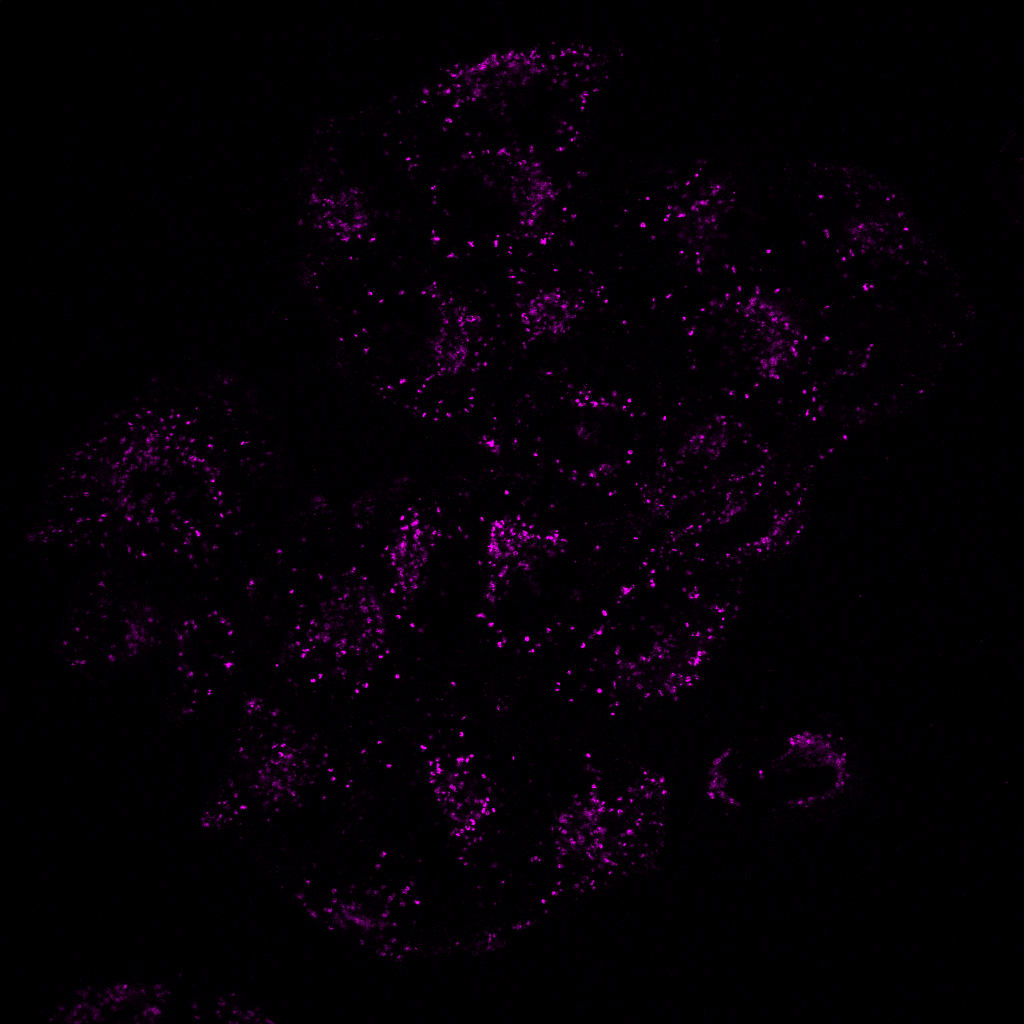

Supplement: Supplementary file 4 — Source Data for Expanded View [file EMBR-24-e57300-s011.zip › Fig EV2/EV2C/siSTK38 #1_non-treated_LAMP1.tif]

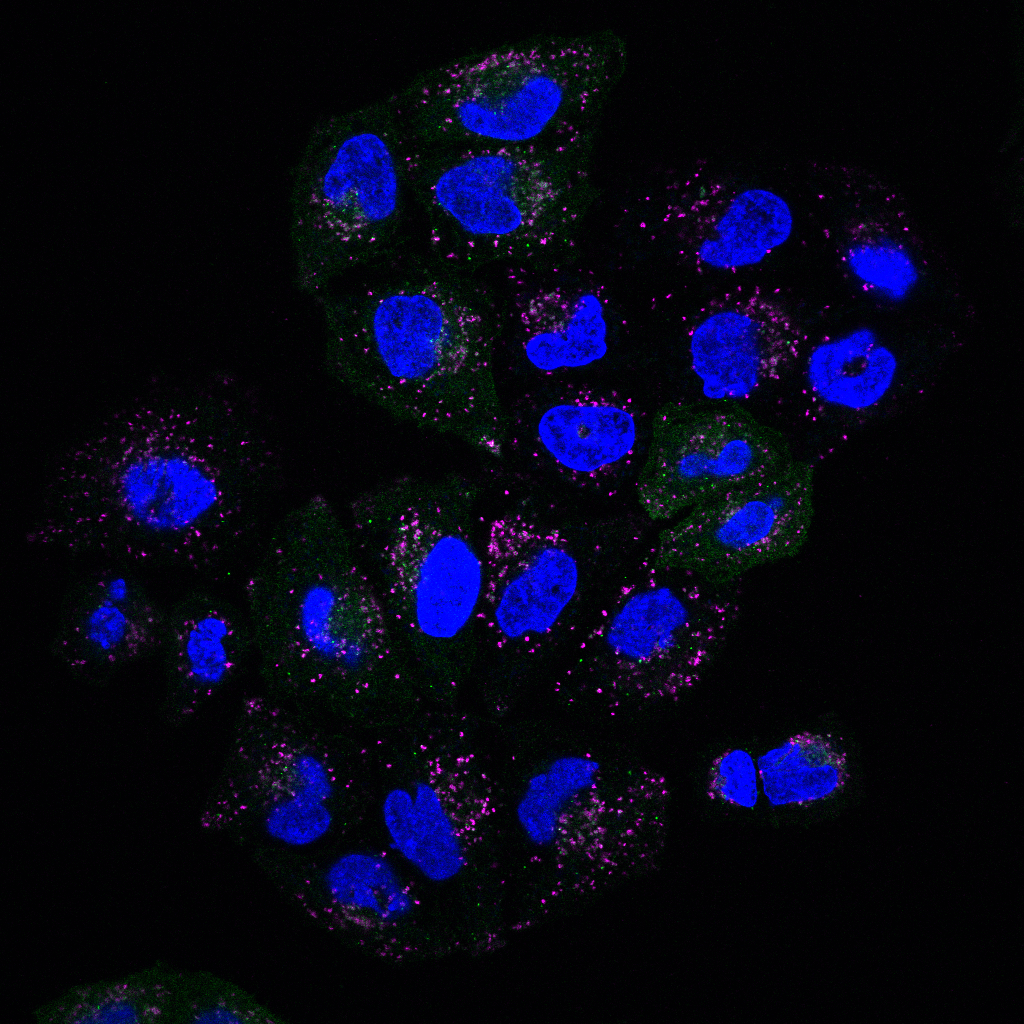

Supplement: Supplementary file 4 — Source Data for Expanded View [file EMBR-24-e57300-s011.zip › Fig EV2/EV2C/siSTK38 #1_non-treated_Merge+DAPI.tif]

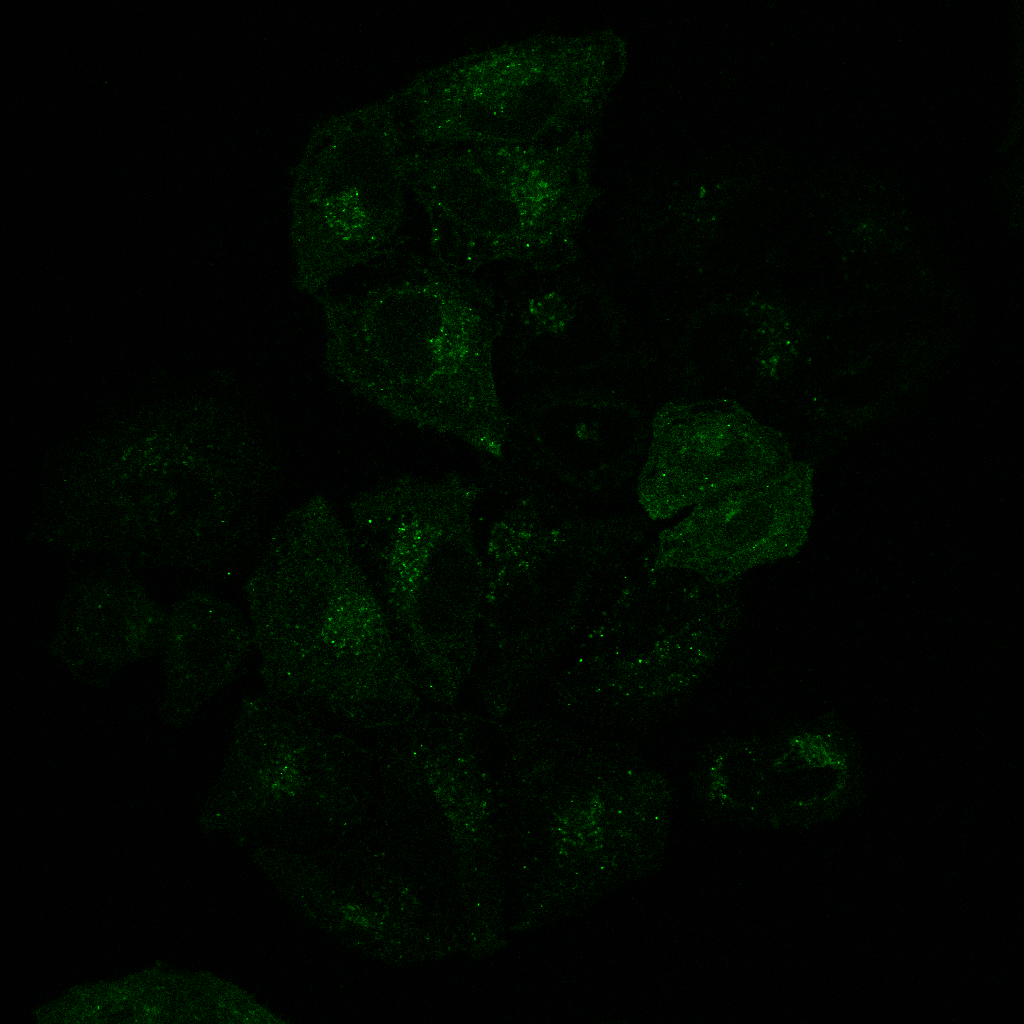

Supplement: Supplementary file 4 — Source Data for Expanded View [file EMBR-24-e57300-s011.zip › Fig EV2/EV2C/siSTK38 #1_non-treated_mNG-ULK1.tif]

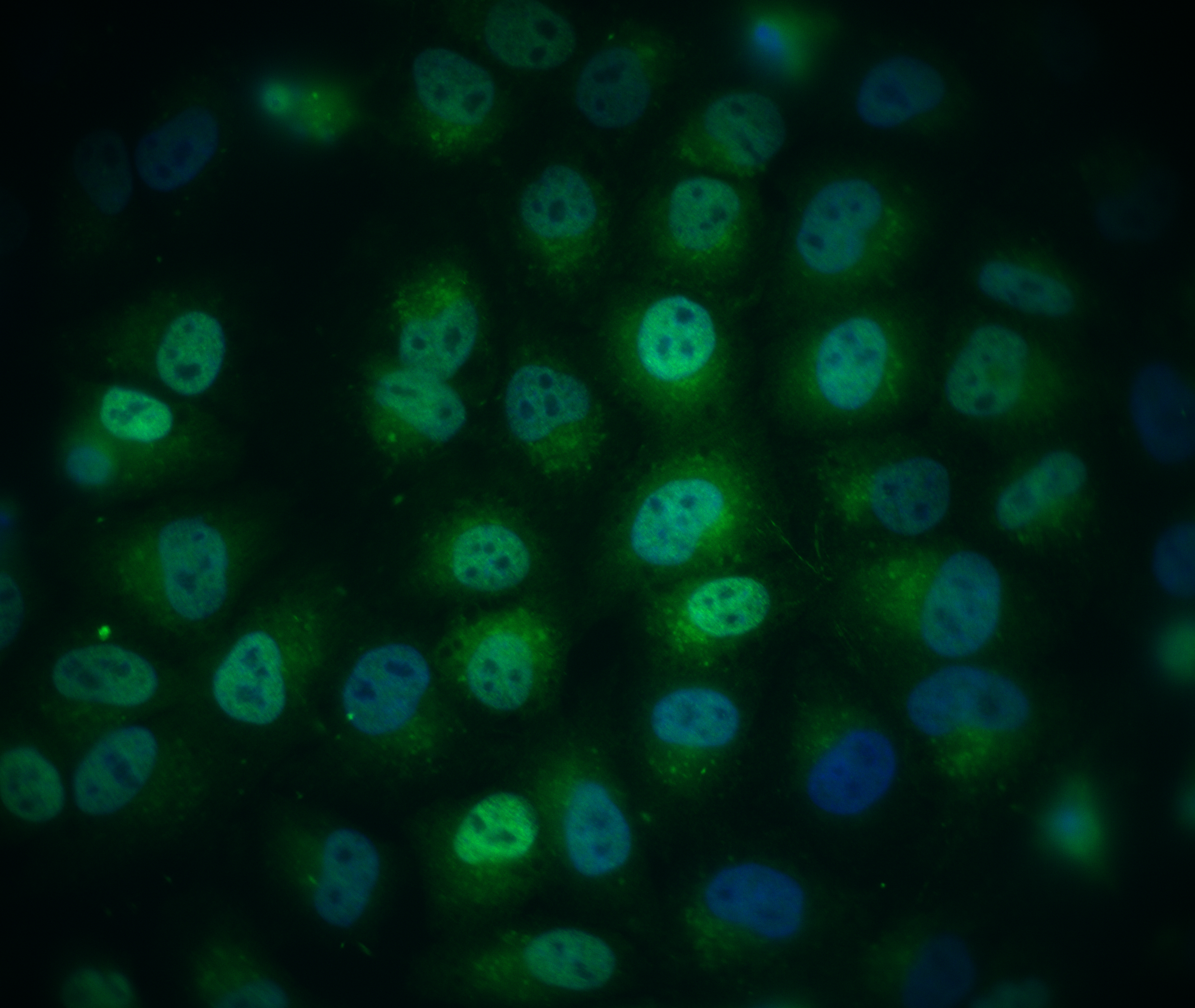

Supplement: Supplementary file 4 — Source Data for Expanded View [file EMBR-24-e57300-s011.zip › Fig EV2/EV2E/siLuc_LLOMe_TFEB-mNG+DAPI.tif]

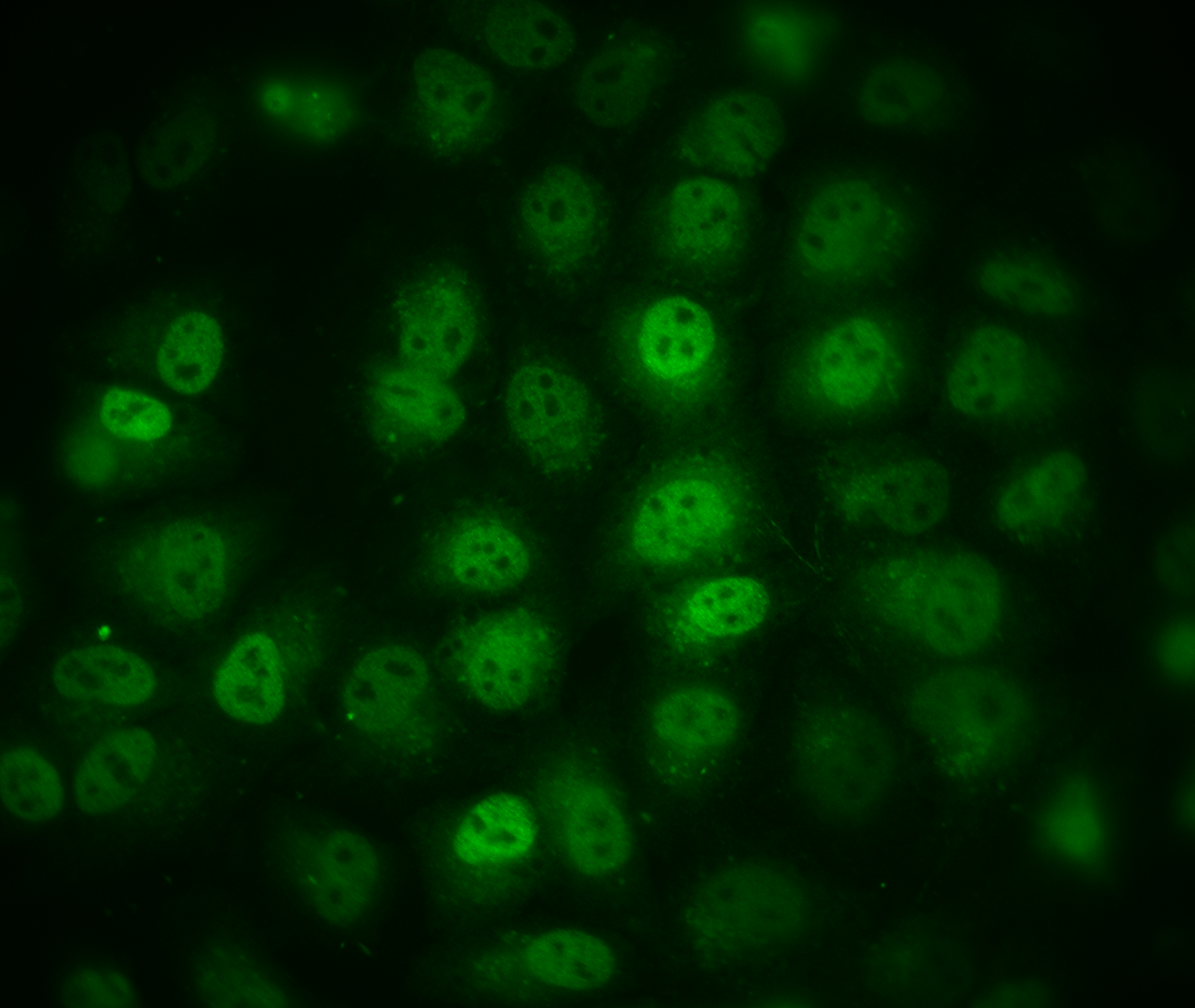

Supplement: Supplementary file 4 — Source Data for Expanded View [file EMBR-24-e57300-s011.zip › Fig EV2/EV2E/siLuc_LLOMe_TFEB-mNG.tif]

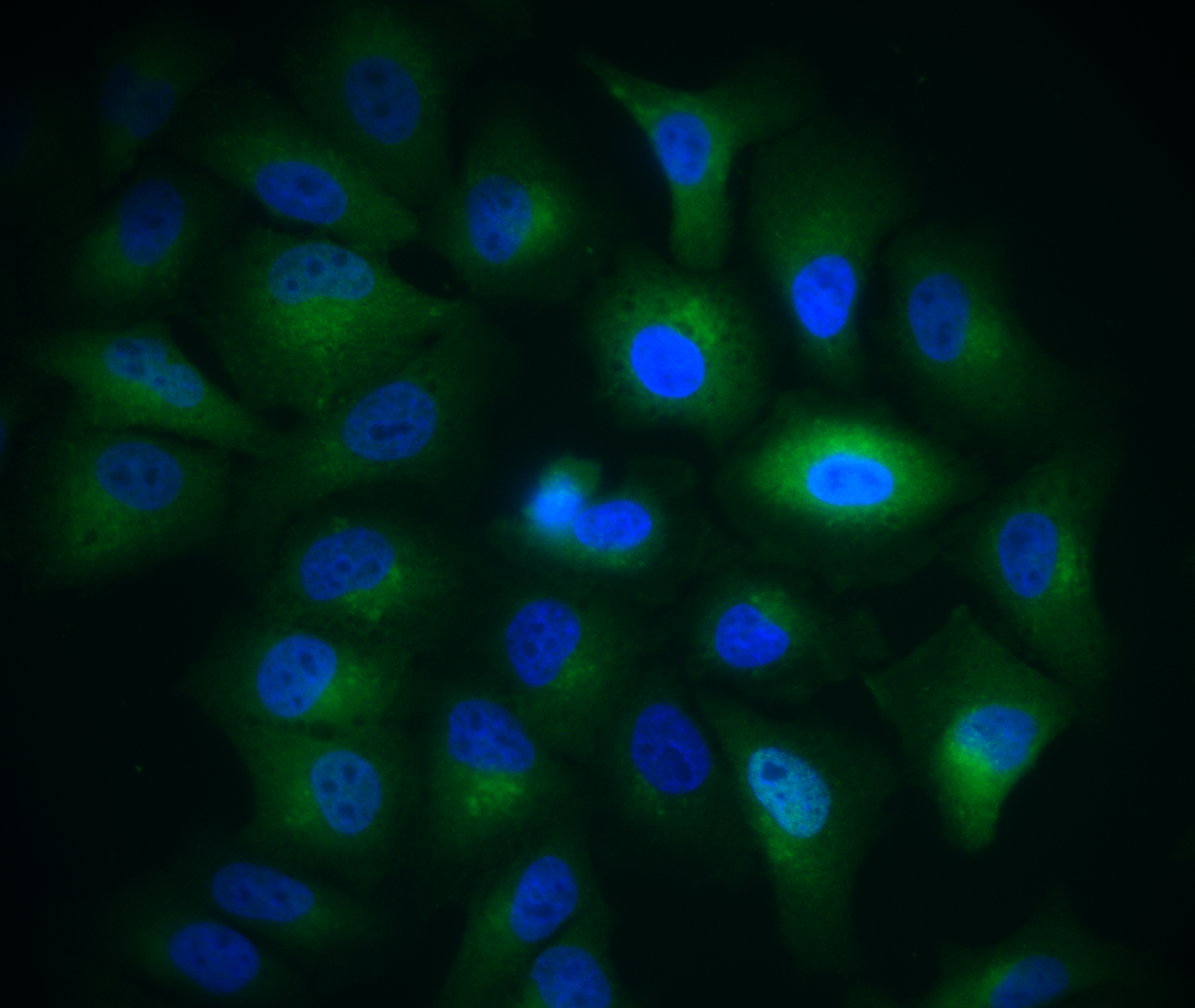

Supplement: Supplementary file 4 — Source Data for Expanded View [file EMBR-24-e57300-s011.zip › Fig EV2/EV2E/siLuc_non-treated_TFEB-mNG+DAPI.tif]

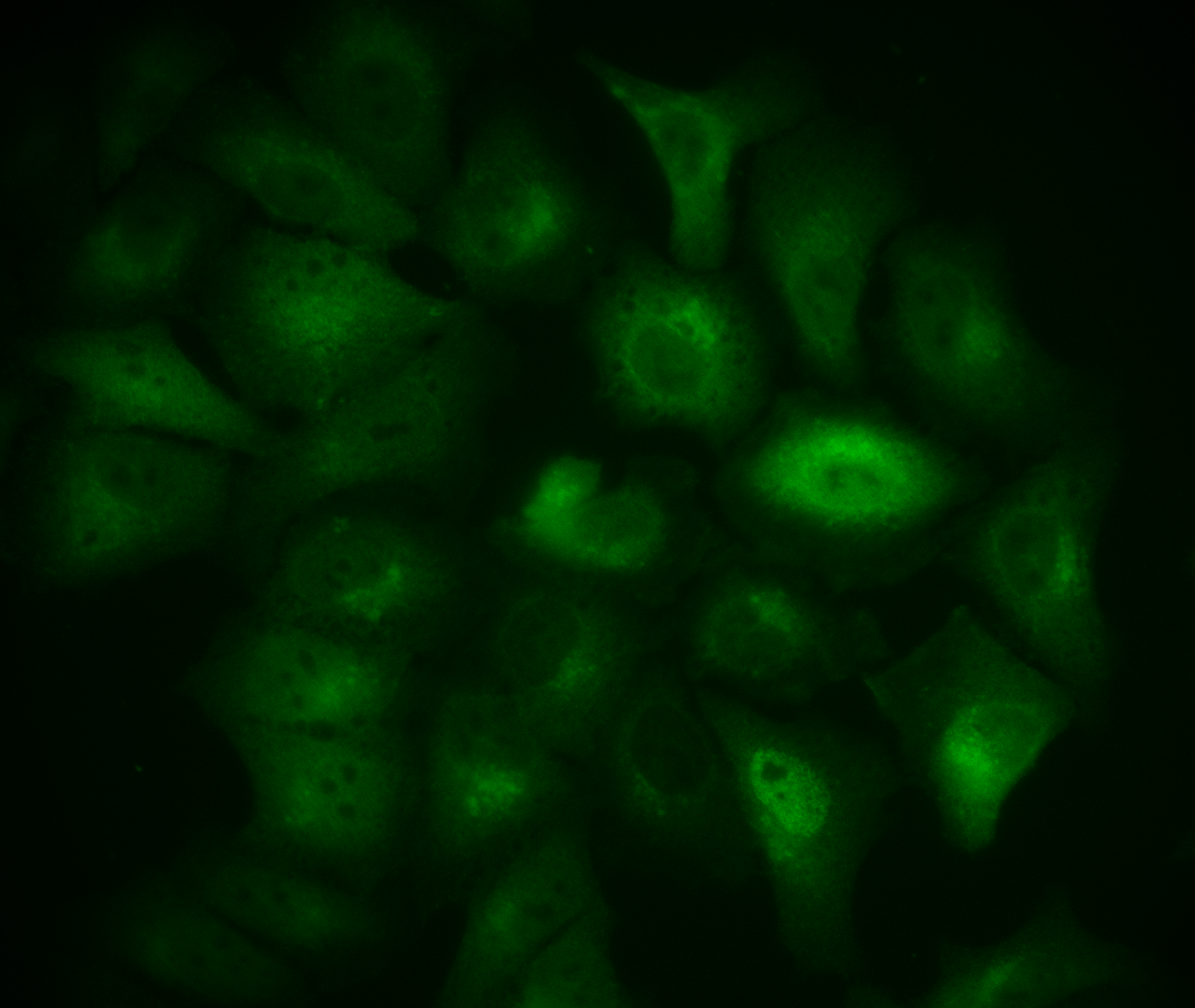

Supplement: Supplementary file 4 — Source Data for Expanded View [file EMBR-24-e57300-s011.zip › Fig EV2/EV2E/siLuc_non-treated_TFEB-mNG.tif]

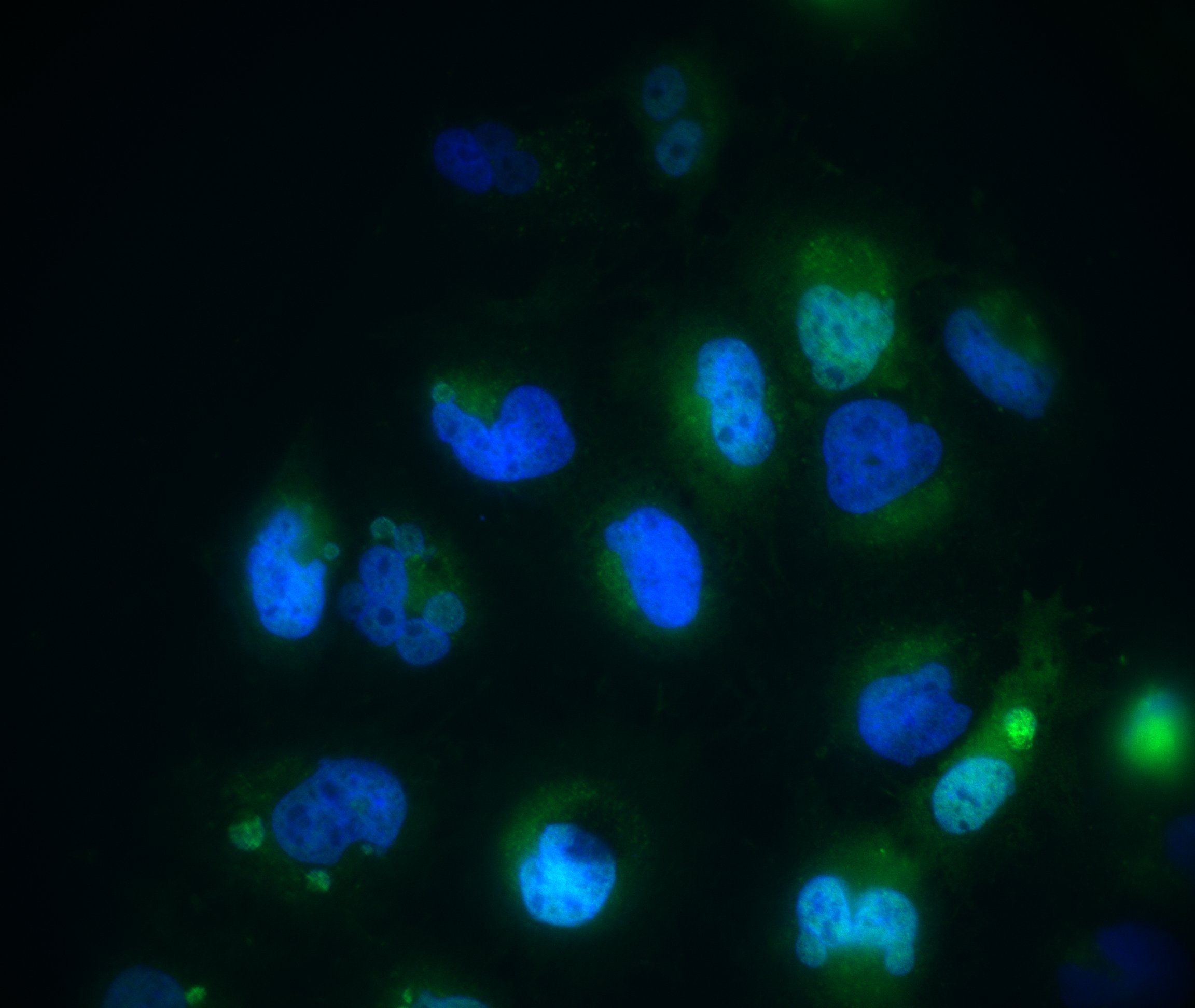

Supplement: Supplementary file 4 — Source Data for Expanded View [file EMBR-24-e57300-s011.zip › Fig EV2/EV2E/siSTK38 #2_LLOMe_TFEB-mNG+DAPI.tif]

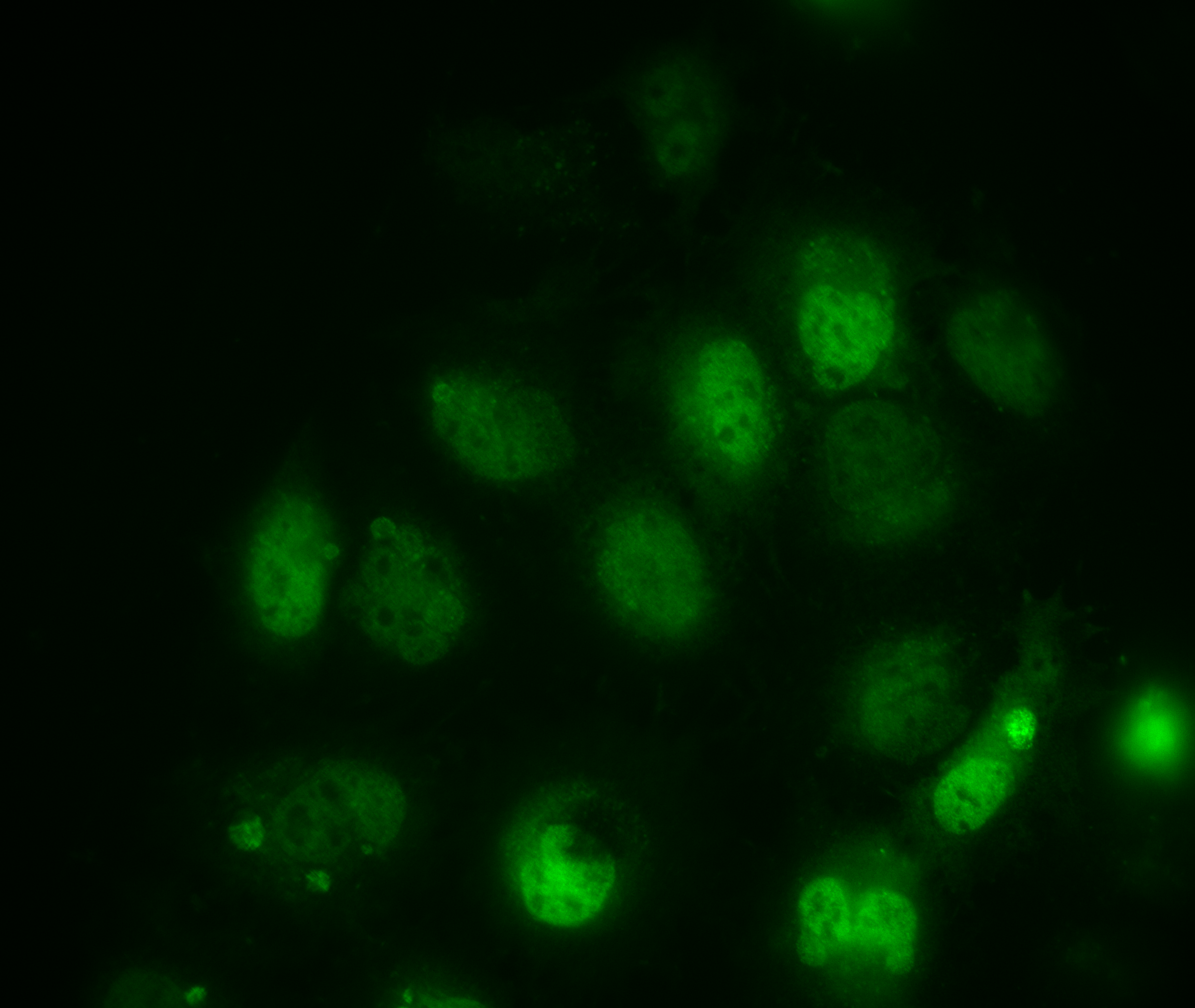

Supplement: Supplementary file 4 — Source Data for Expanded View [file EMBR-24-e57300-s011.zip › Fig EV2/EV2E/siSTK38 #2_LLOMe_TFEB-mNG.tif]

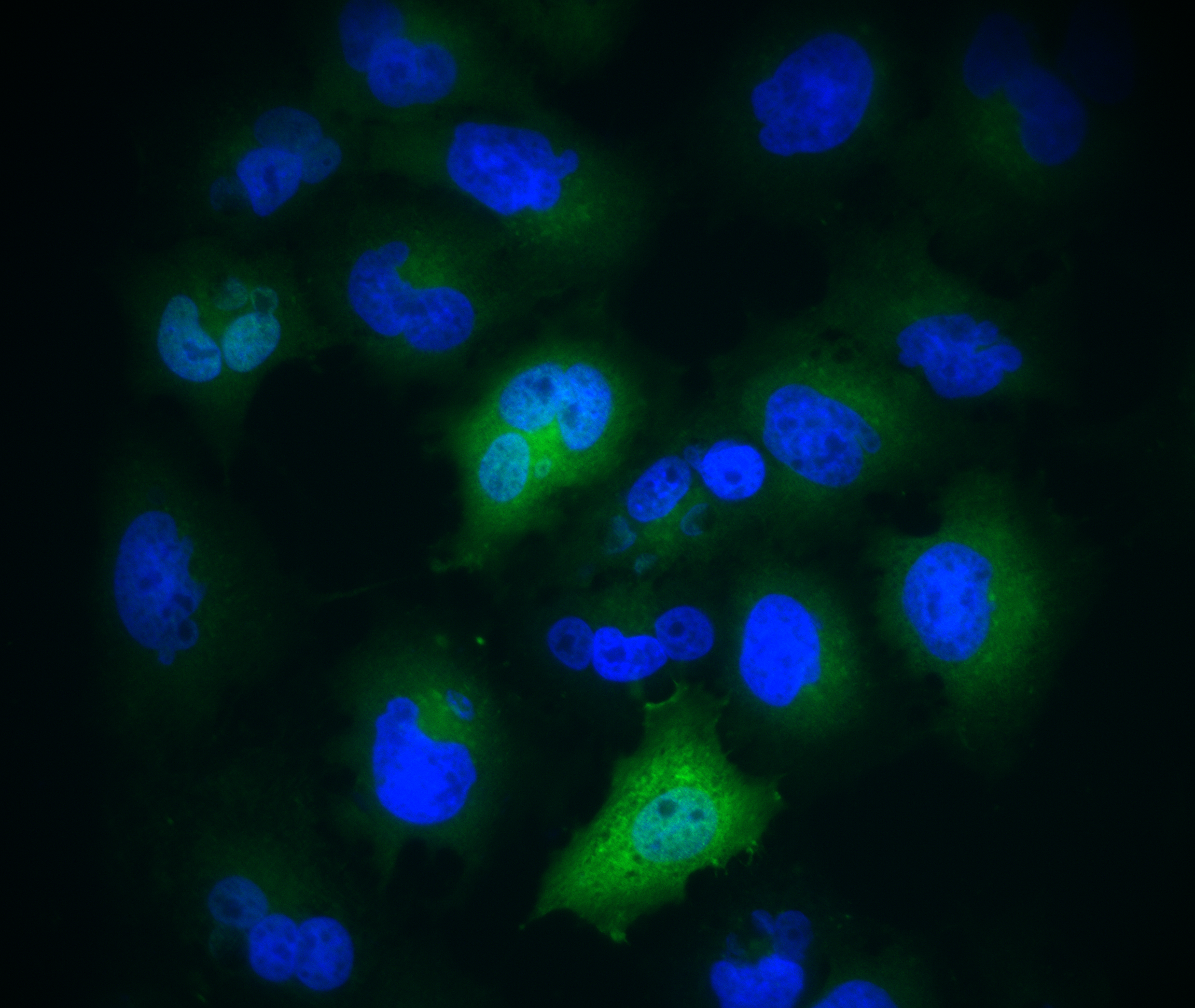

Supplement: Supplementary file 4 — Source Data for Expanded View [file EMBR-24-e57300-s011.zip › Fig EV2/EV2E/siSTK38 #2_non-treated_mNG-TFEB+DAPI.tif]

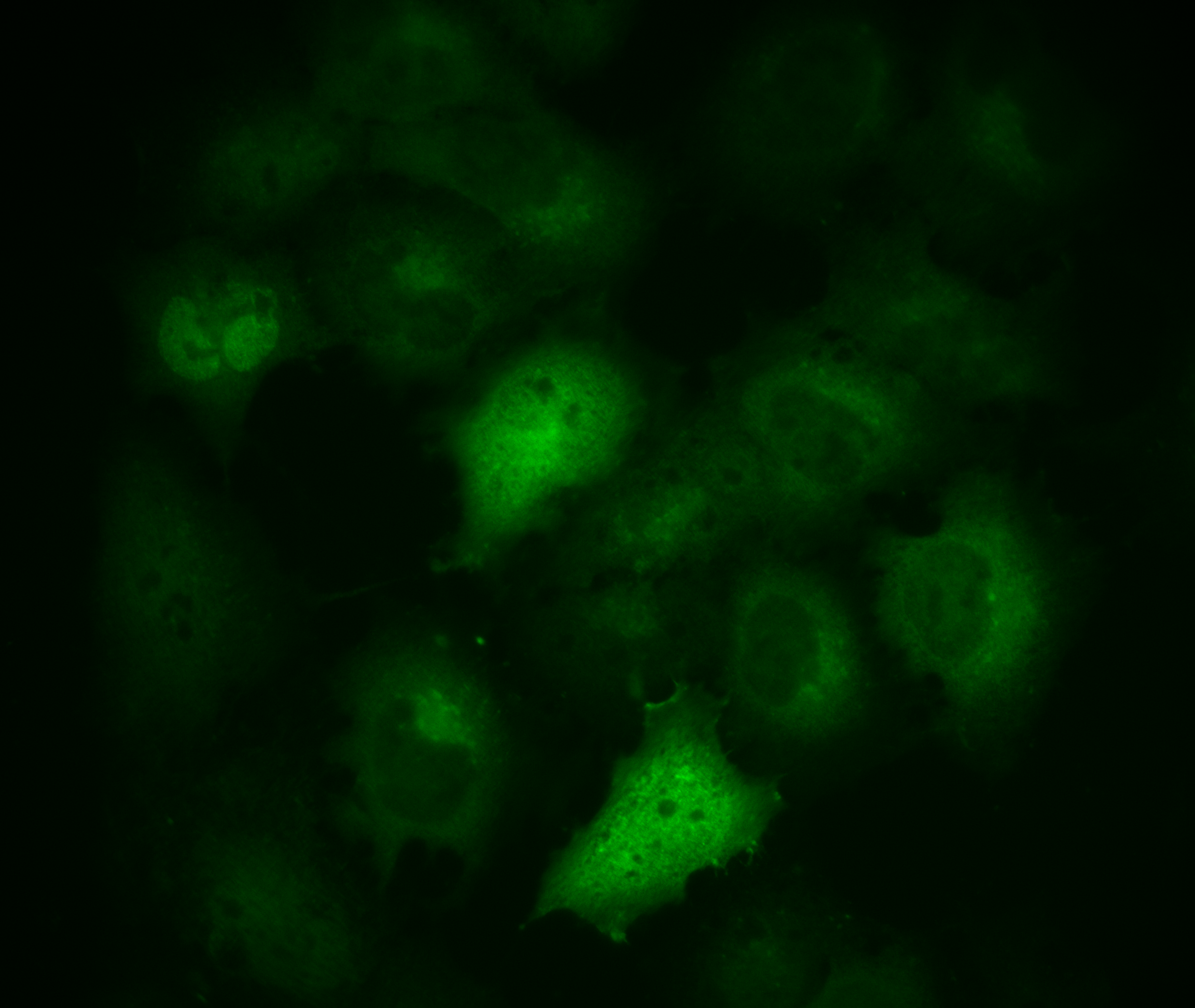

Supplement: Supplementary file 4 — Source Data for Expanded View [file EMBR-24-e57300-s011.zip › Fig EV2/EV2E/siSTK38 #2_non-treated_TFEB-mNG.tif]

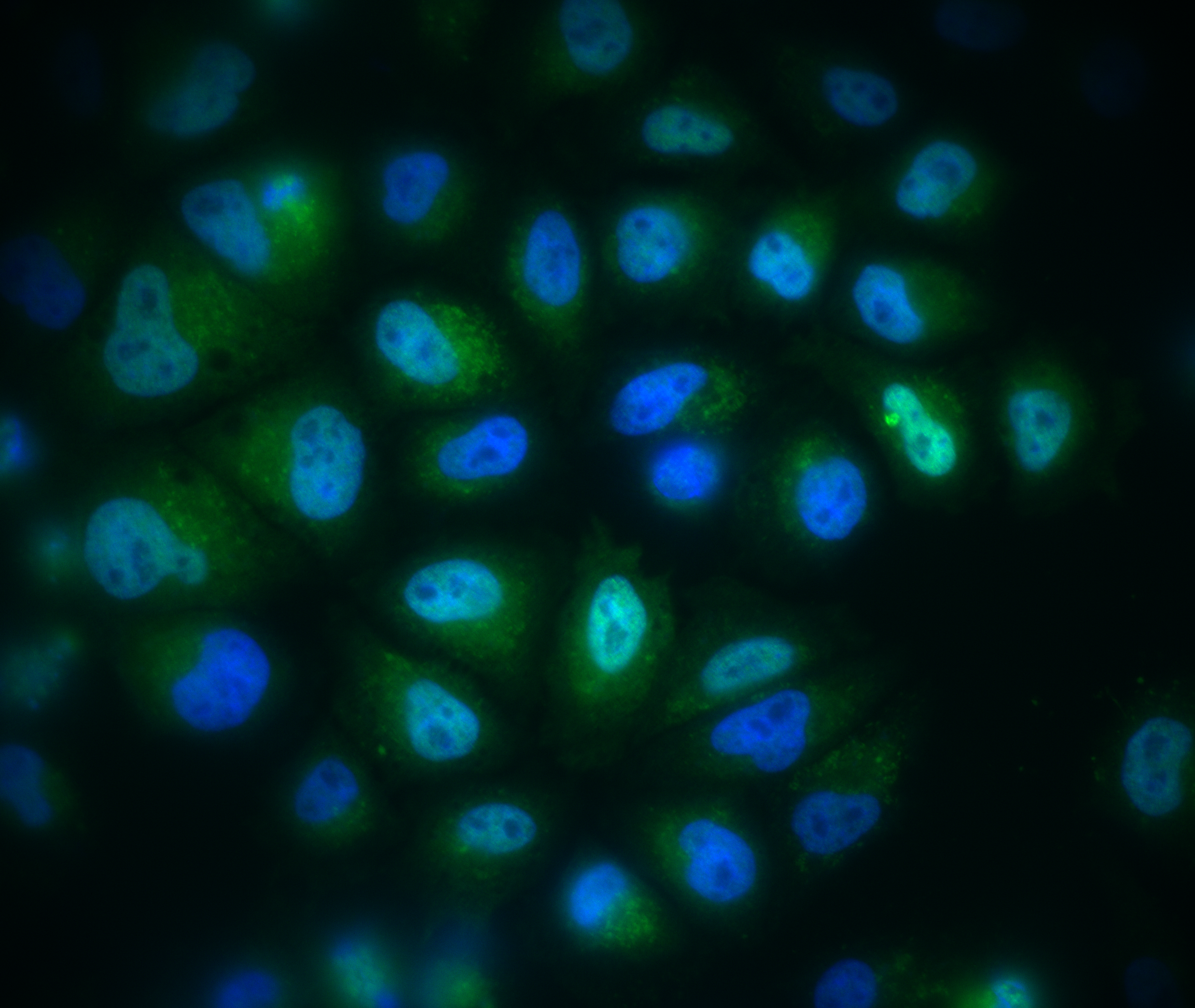

Supplement: Supplementary file 4 — Source Data for Expanded View [file EMBR-24-e57300-s011.zip › Fig EV2/EV2E/siSTK38 #3_LLOMe_TFEB-mNG+DAPI.tif]

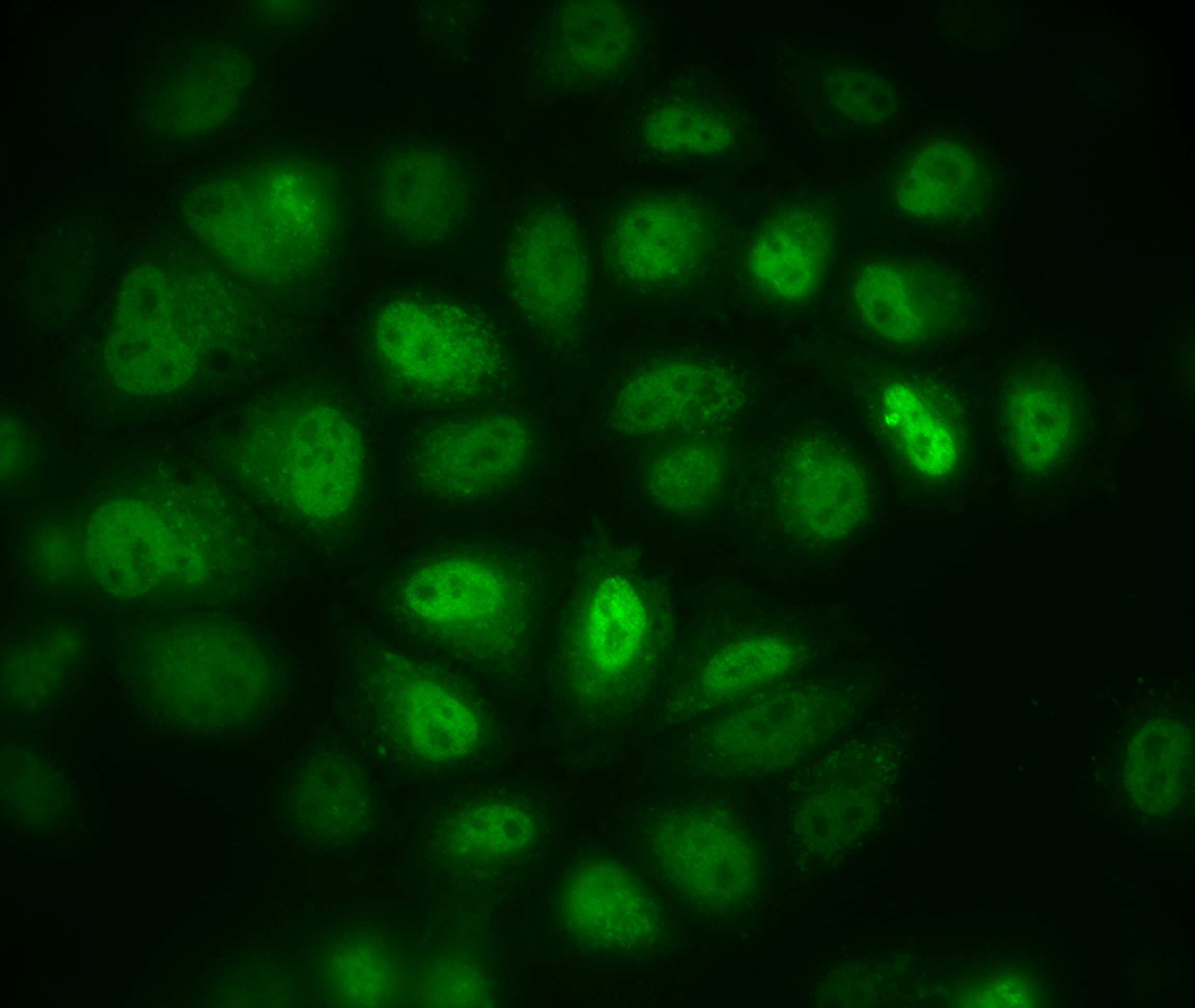

Supplement: Supplementary file 4 — Source Data for Expanded View [file EMBR-24-e57300-s011.zip › Fig EV2/EV2E/siSTK38 #3_LLOMe_TFEB-mNG.tif]

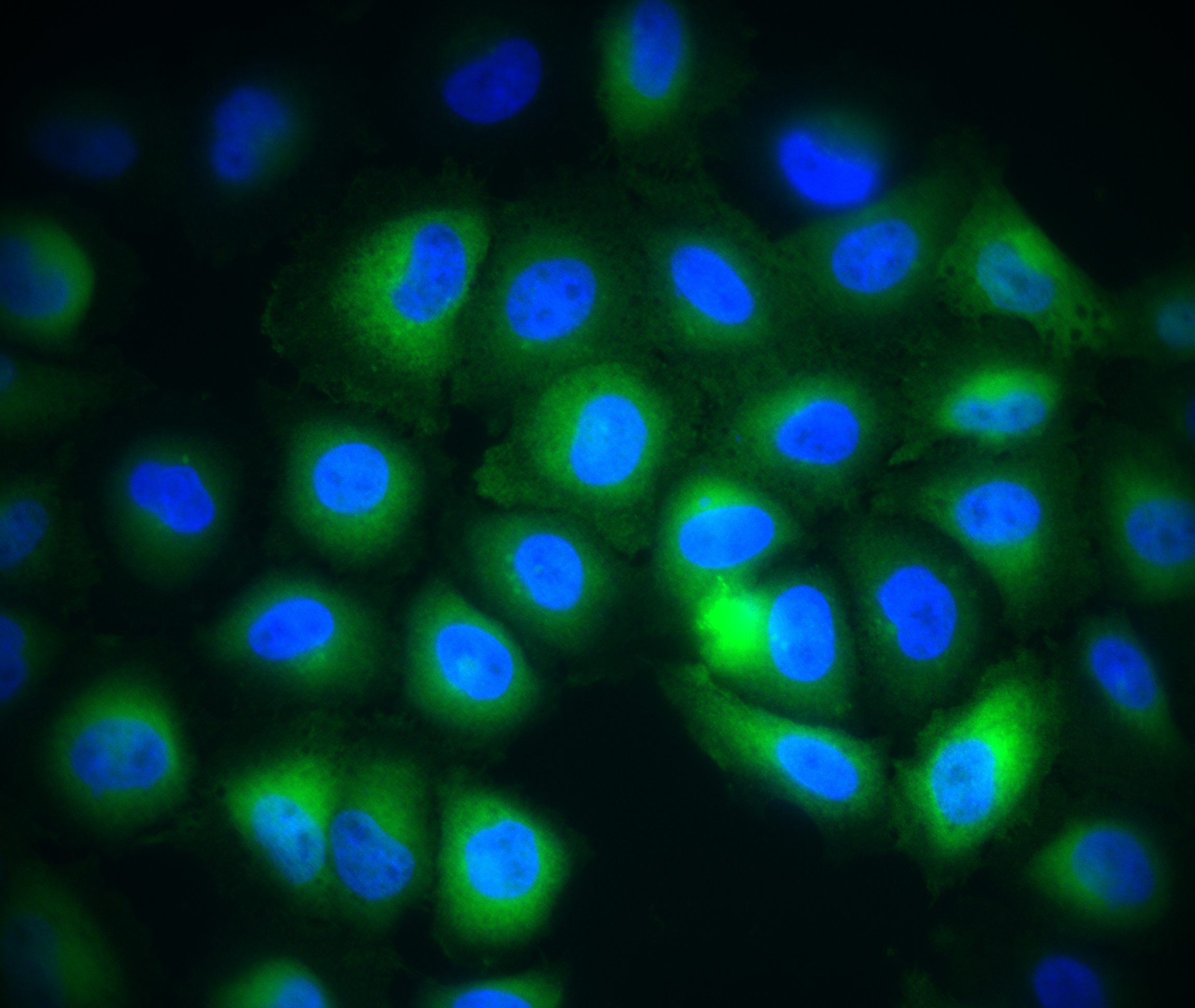

Supplement: Supplementary file 4 — Source Data for Expanded View [file EMBR-24-e57300-s011.zip › Fig EV2/EV2E/siSTK38 #3_non-treated_TFEB-mNG+DAPI.tif]

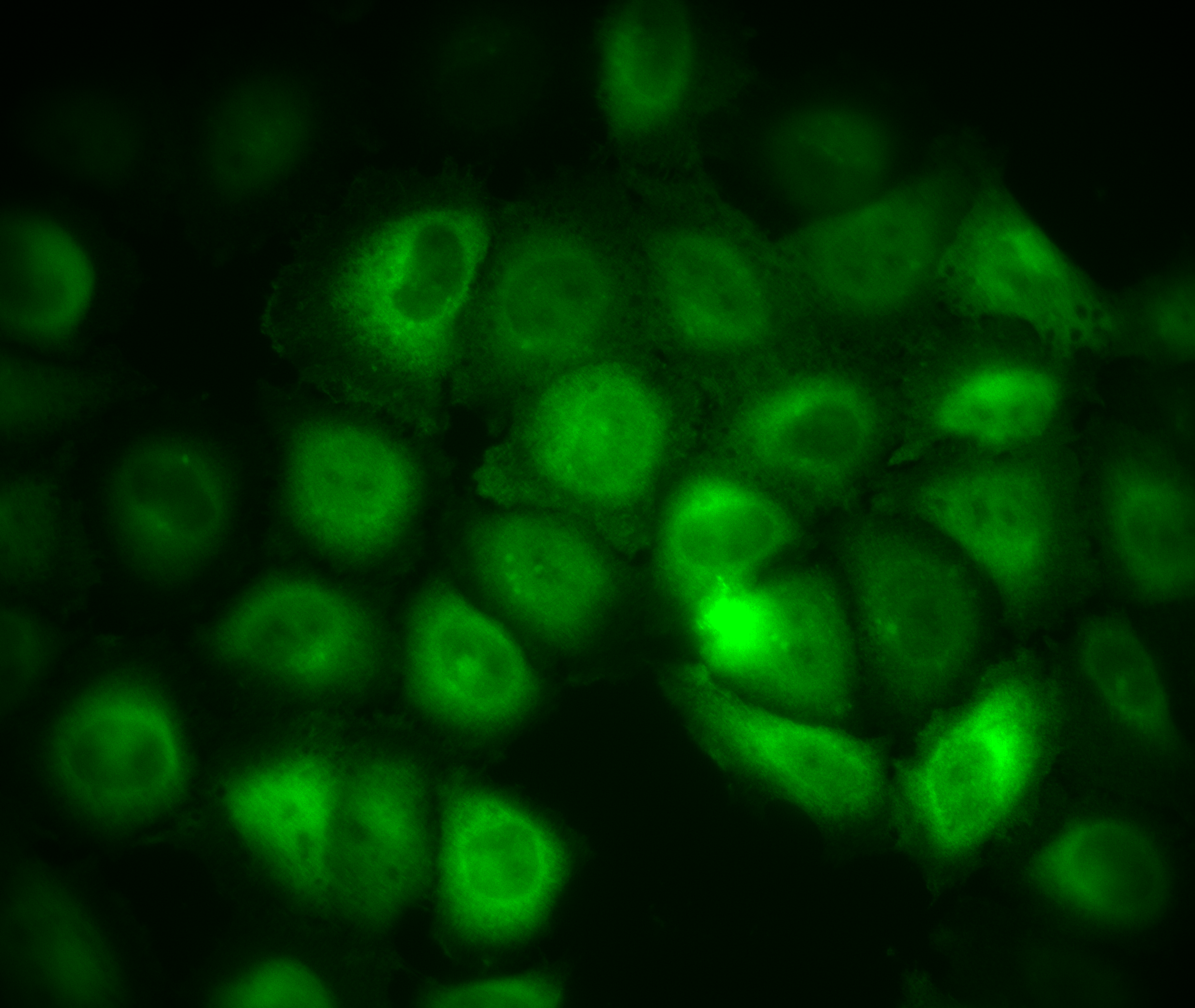

Supplement: Supplementary file 4 — Source Data for Expanded View [file EMBR-24-e57300-s011.zip › Fig EV2/EV2E/siSTK38 #3_non-treated_TFEB-mNG.tif]

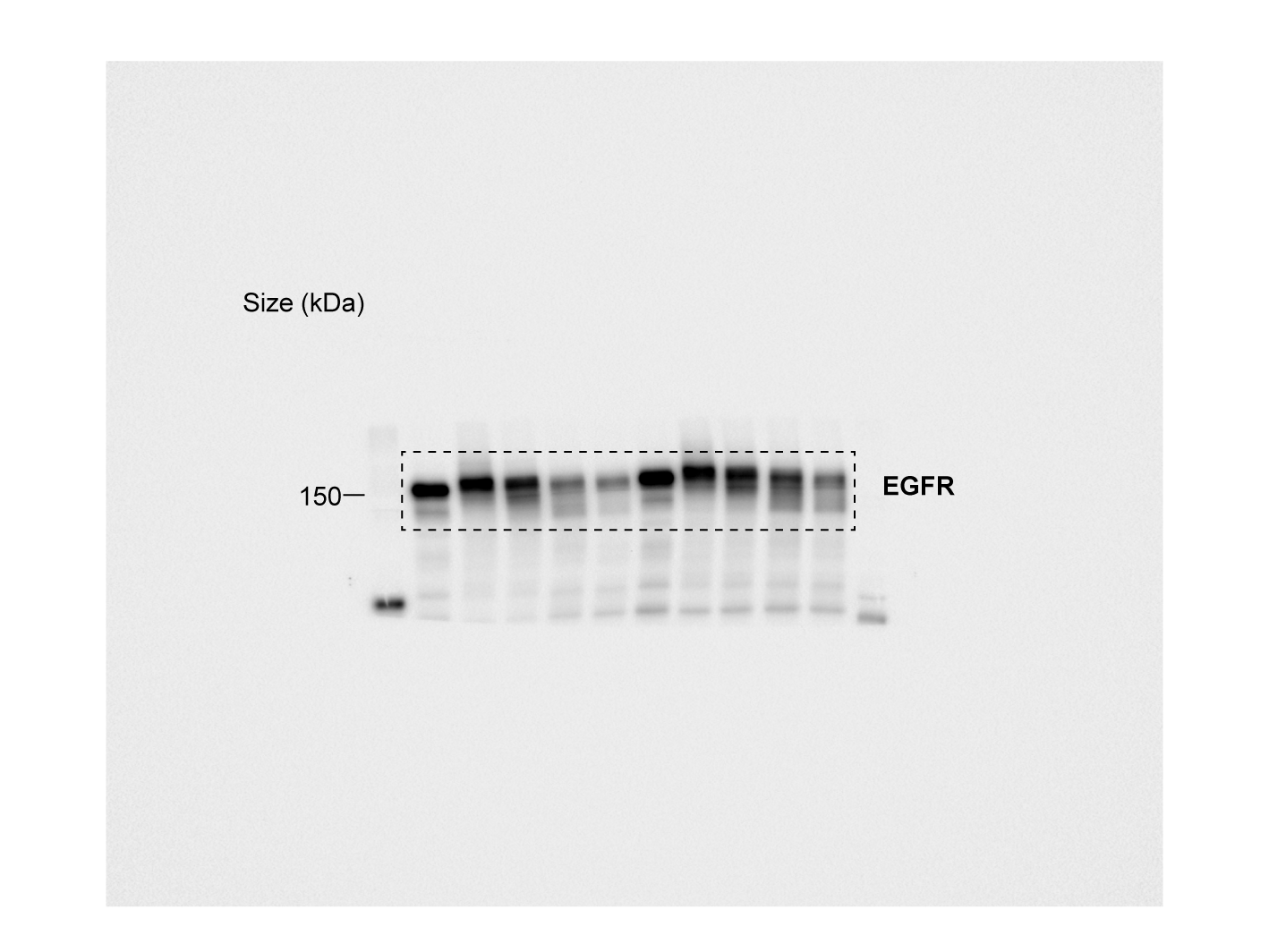

Supplement: Supplementary file 4 — Source Data for Expanded View [file EMBR-24-e57300-s011.zip › Fig EV2/EV2G/western_EGFR.tif]

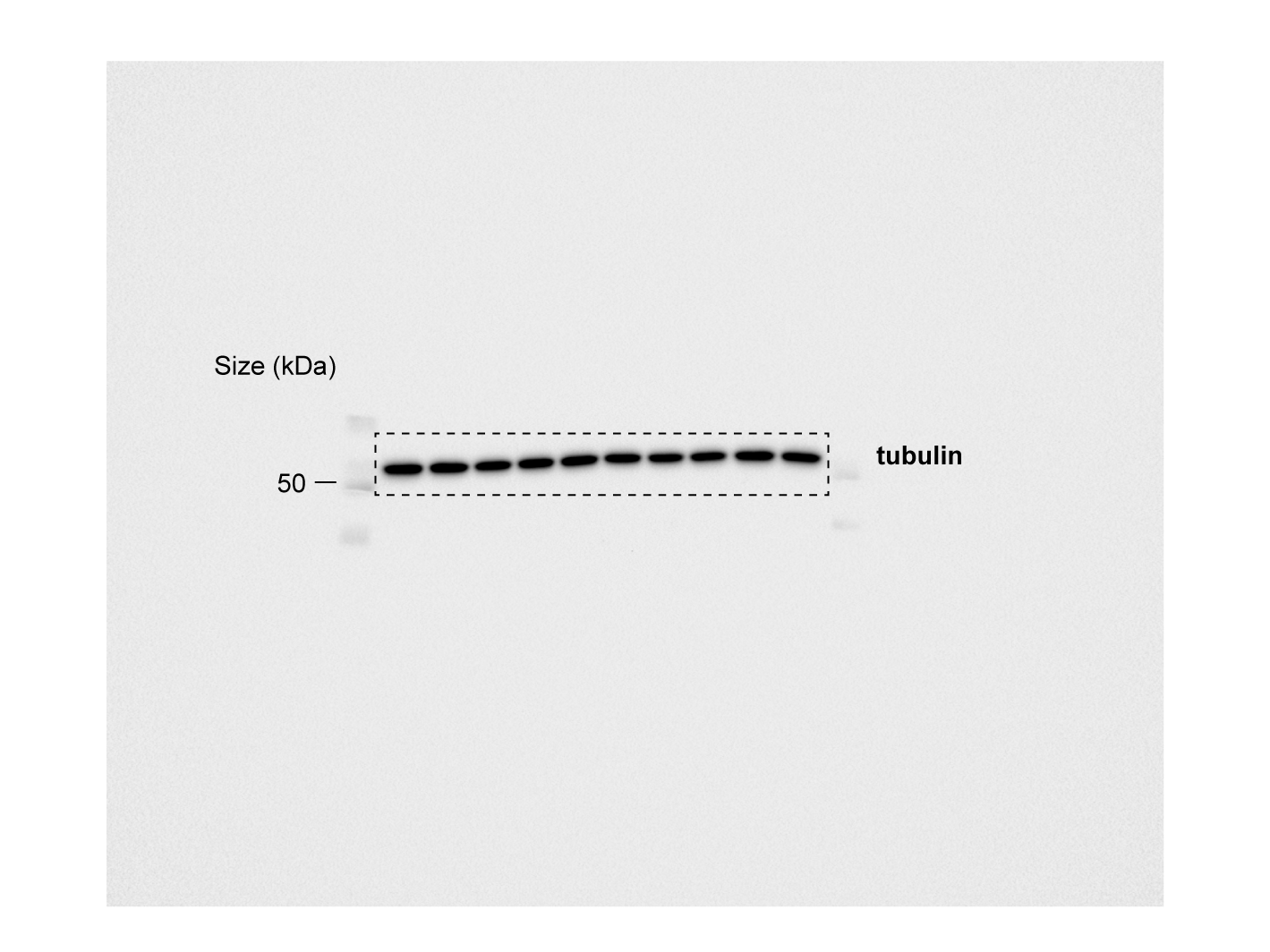

Supplement: Supplementary file 4 — Source Data for Expanded View [file EMBR-24-e57300-s011.zip › Fig EV2/EV2G/western_tubulin.tif]

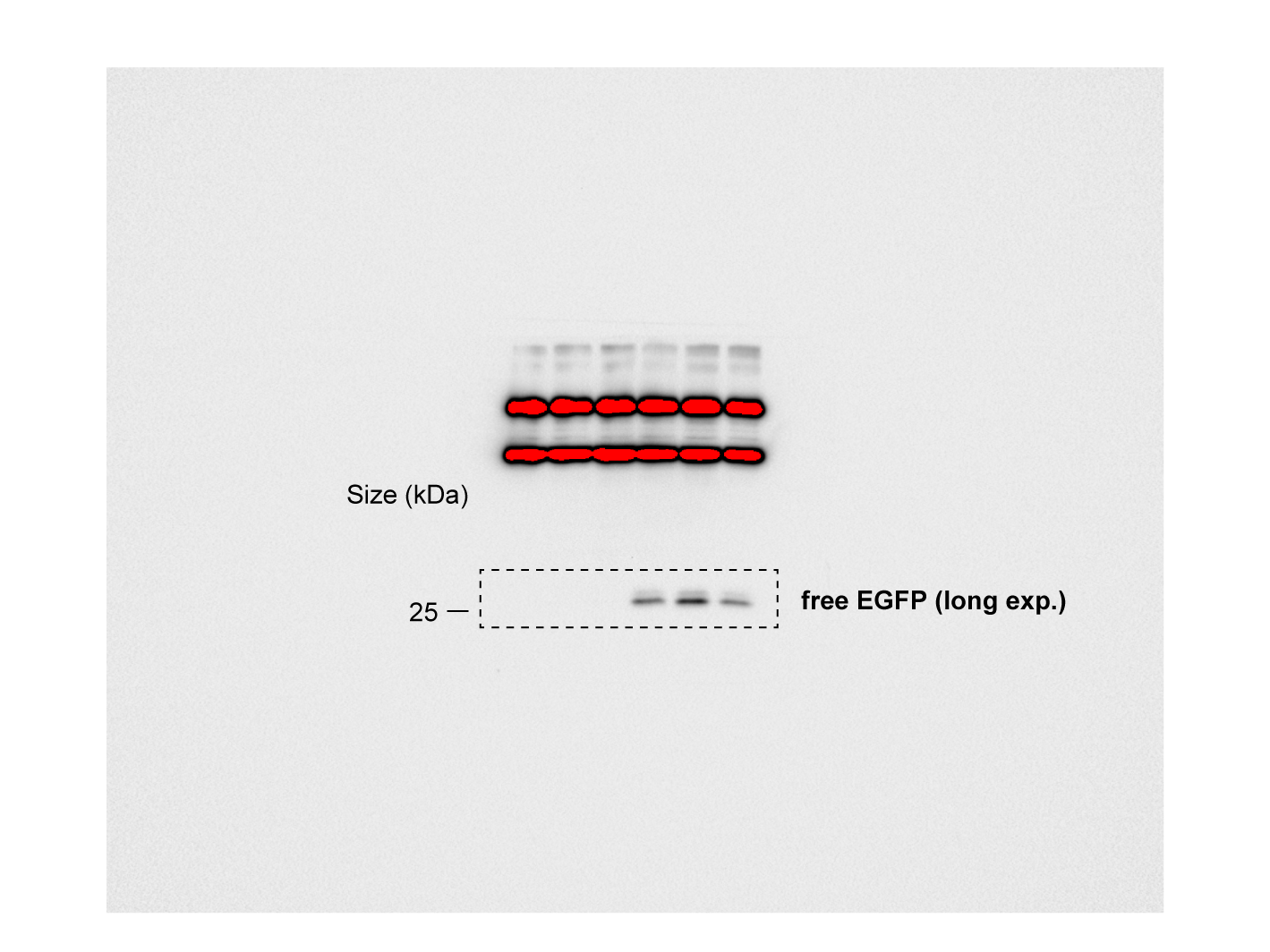

Supplement: Supplementary file 4 — Source Data for Expanded View [file EMBR-24-e57300-s011.zip › Fig EV4/EV4A/western_EGFP (long exp.).tif]

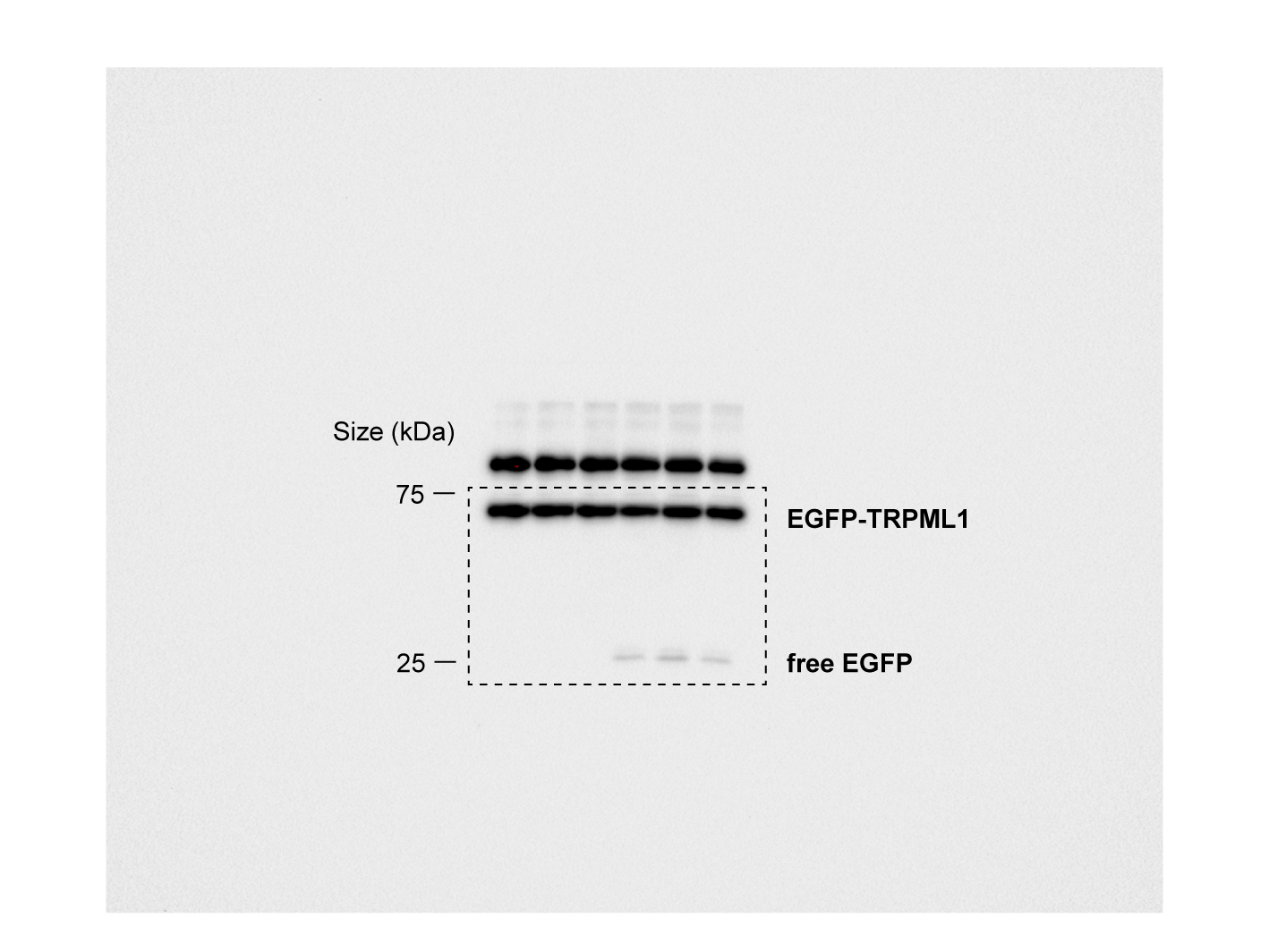

Supplement: Supplementary file 4 — Source Data for Expanded View [file EMBR-24-e57300-s011.zip › Fig EV4/EV4A/western_EGFP.tif]

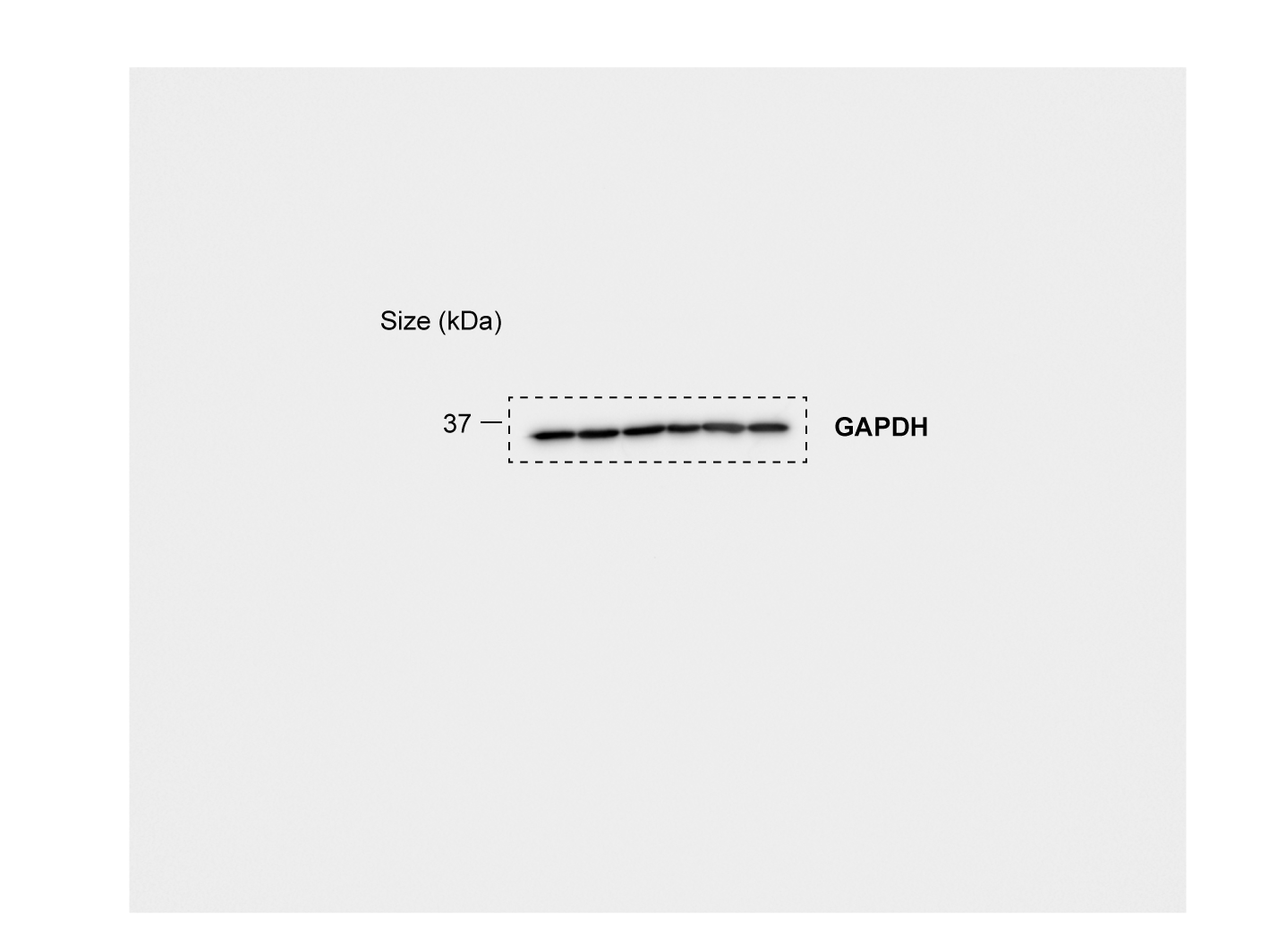

Supplement: Supplementary file 4 — Source Data for Expanded View [file EMBR-24-e57300-s011.zip › Fig EV4/EV4A/western_GAPDH.tif]

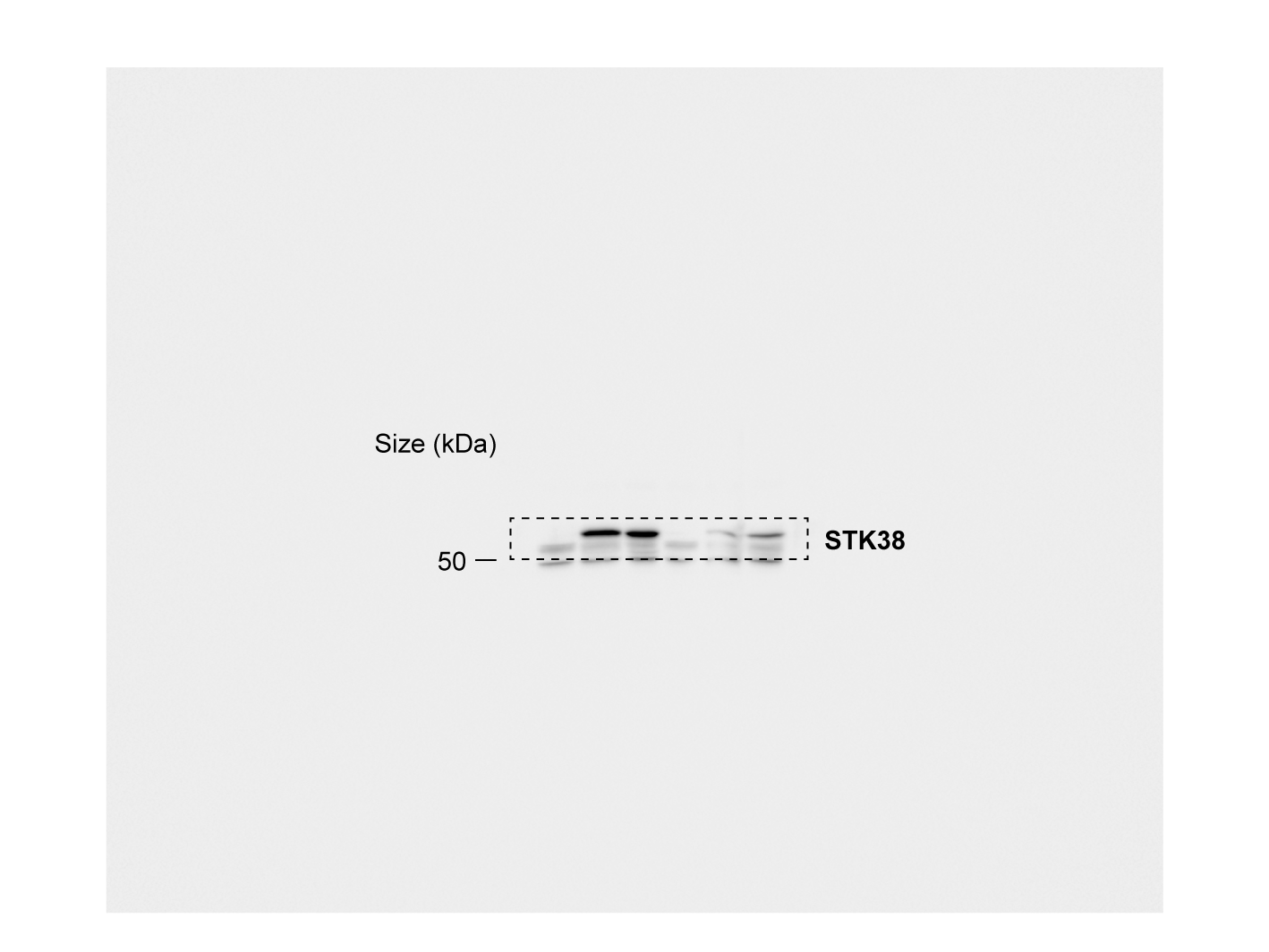

Supplement: Supplementary file 4 — Source Data for Expanded View [file EMBR-24-e57300-s011.zip › Fig EV4/EV4A/western_STK38.tif]

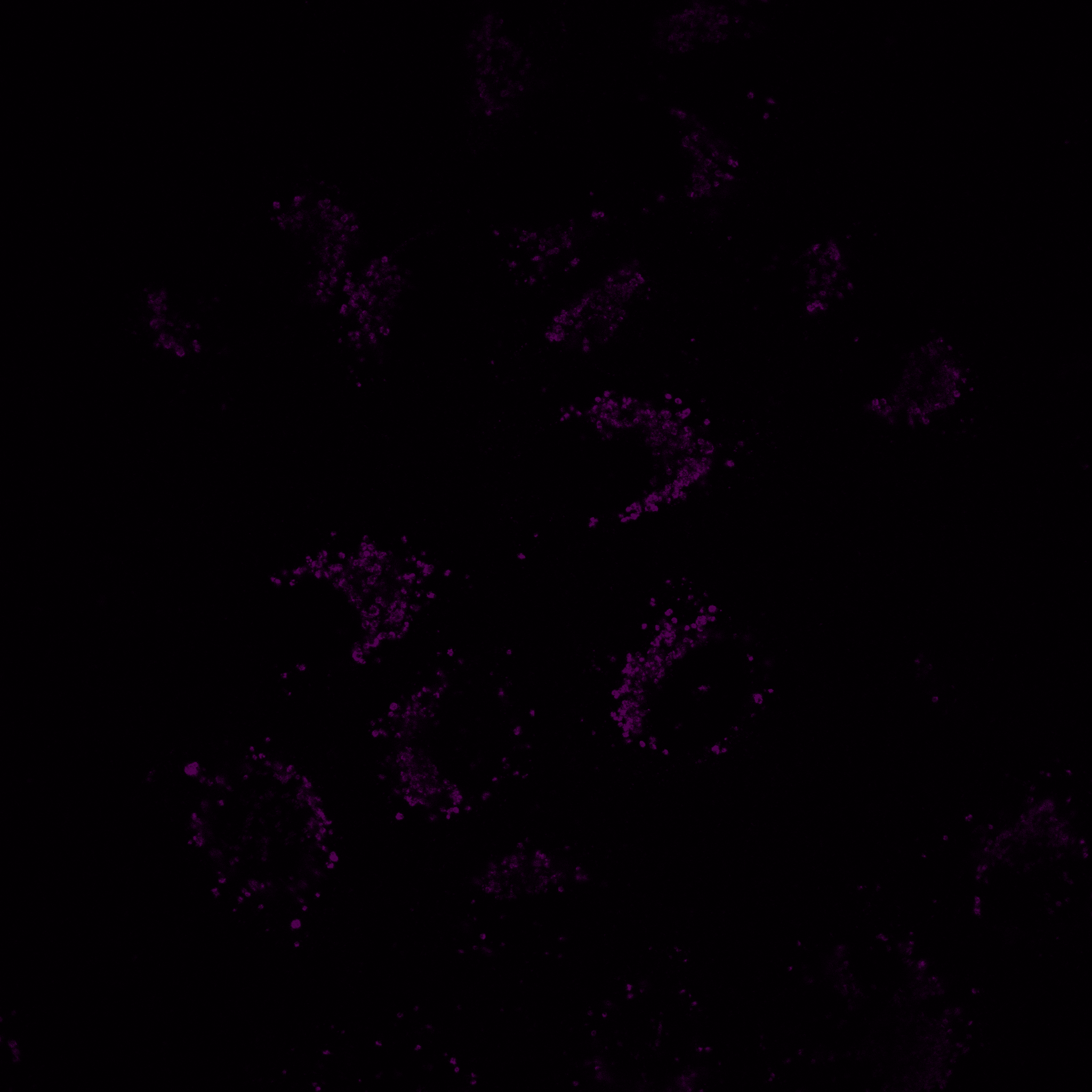

Supplement: Supplementary file 4 — Source Data for Expanded View [file EMBR-24-e57300-s011.zip › Fig EV4/EV4C/LLOMe_LAMP1.tif]

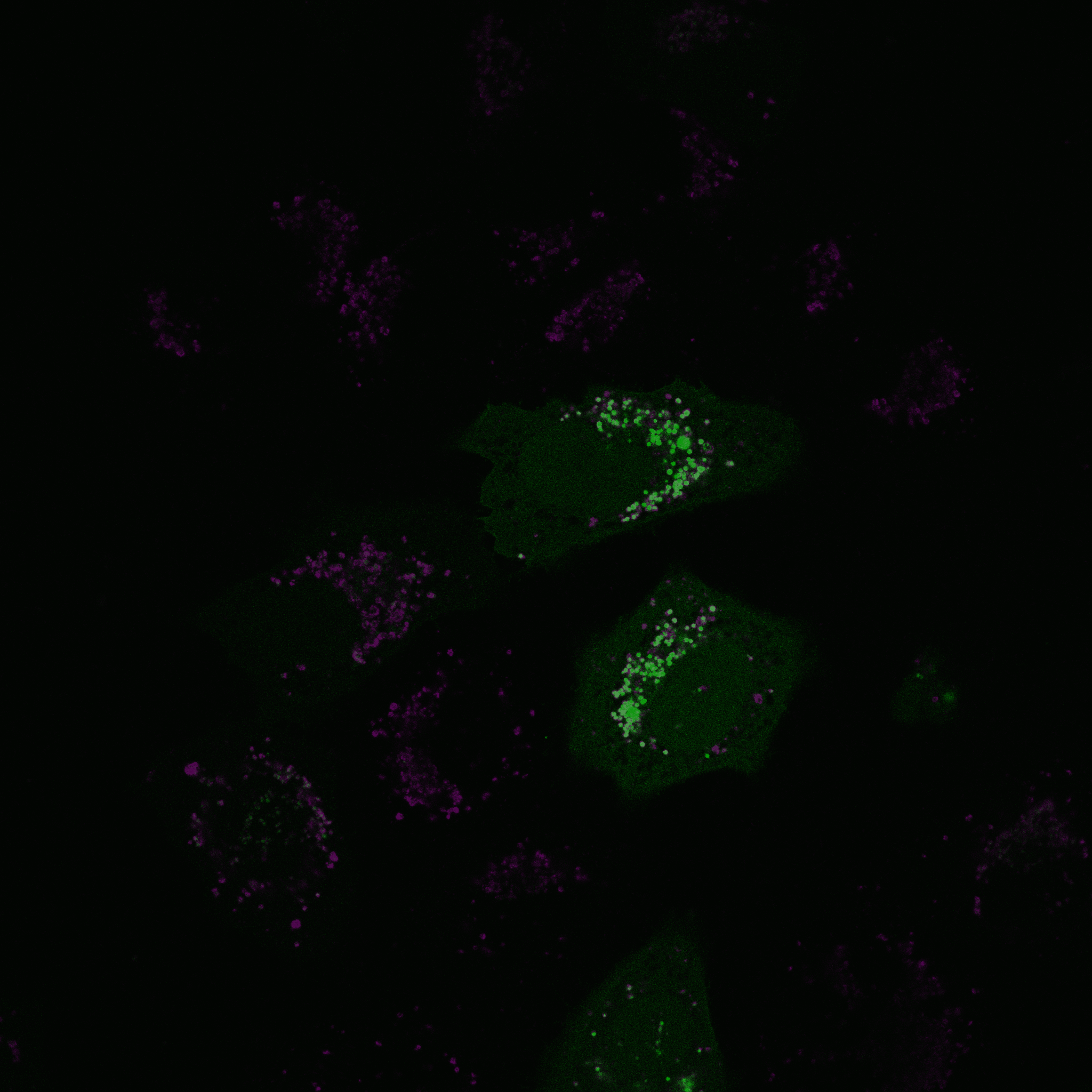

Supplement: Supplementary file 4 — Source Data for Expanded View [file EMBR-24-e57300-s011.zip › Fig EV4/EV4C/LLOMe_Merge.tif]

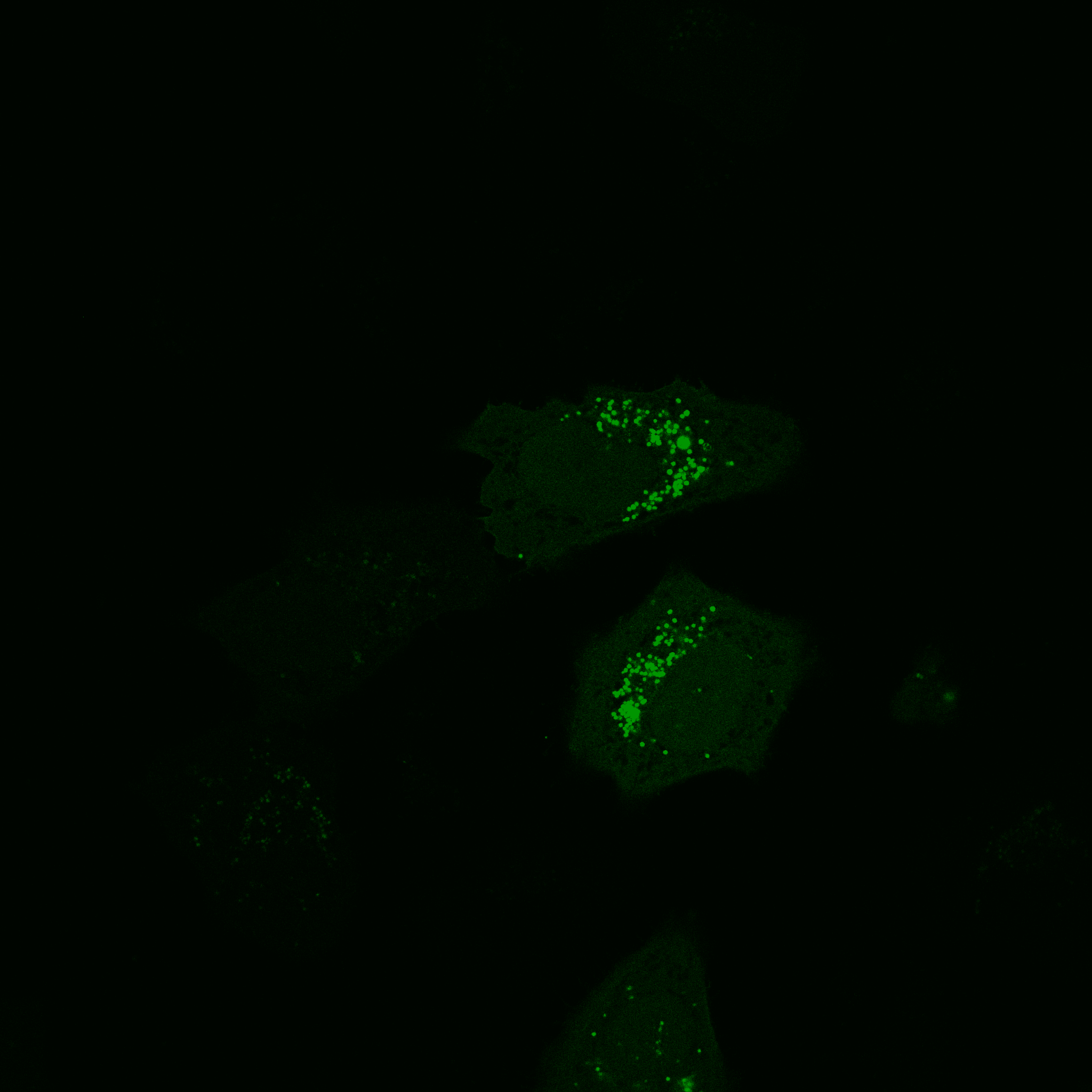

Supplement: Supplementary file 4 — Source Data for Expanded View [file EMBR-24-e57300-s011.zip › Fig EV4/EV4C/LLOMe_mNG-STK38 K118R.tif]

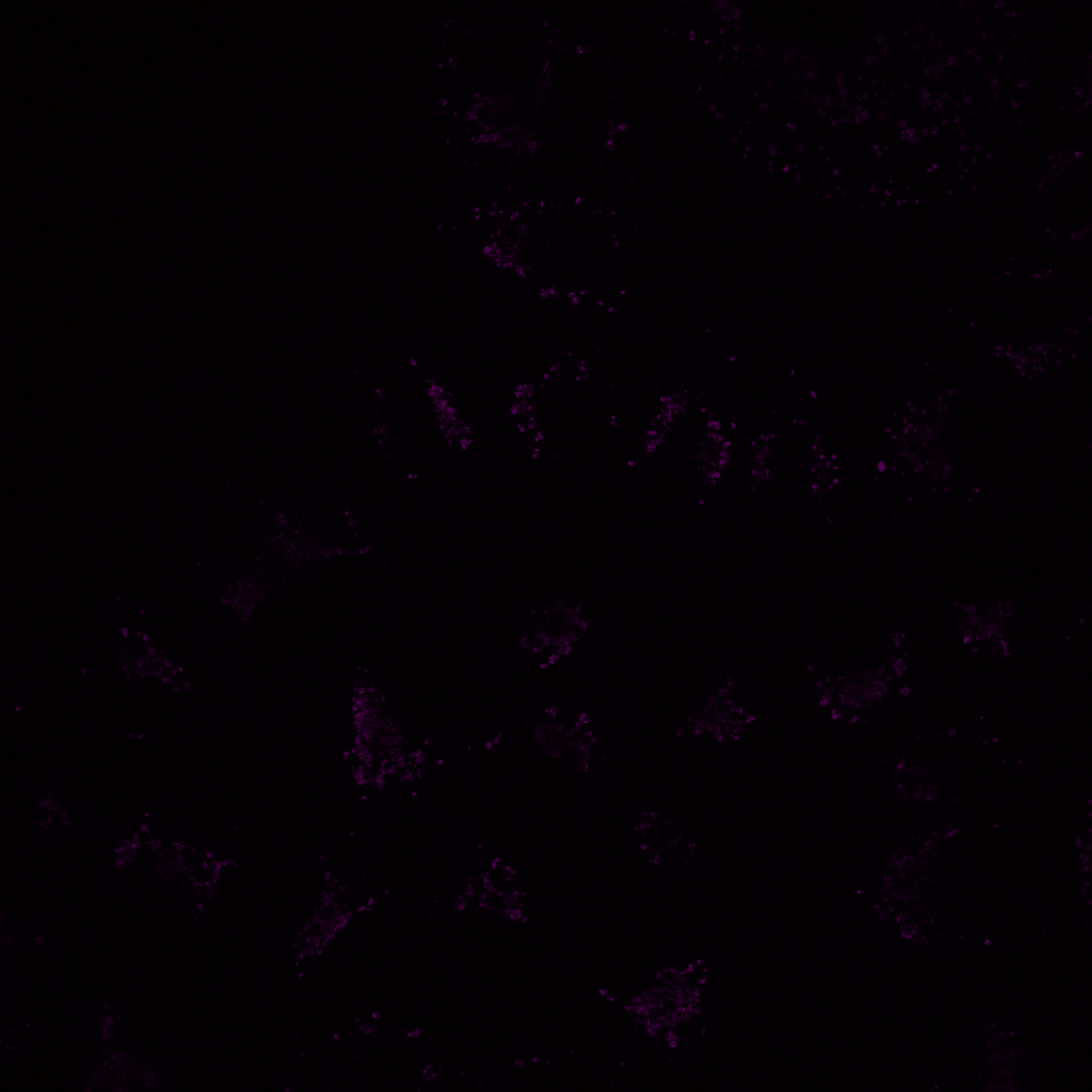

Supplement: Supplementary file 4 — Source Data for Expanded View [file EMBR-24-e57300-s011.zip › Fig EV4/EV4C/non-treated_LAMP1.tif]

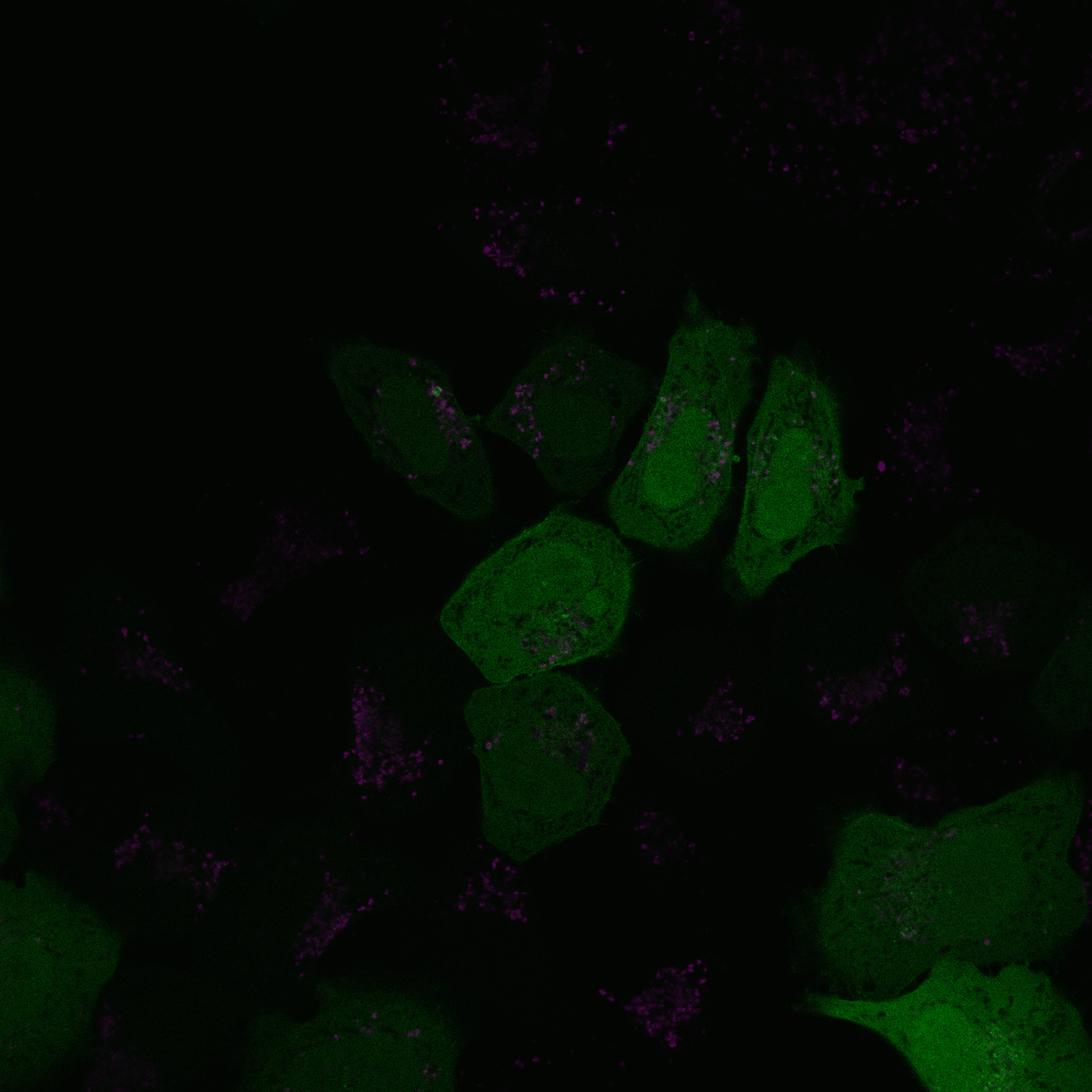

Supplement: Supplementary file 4 — Source Data for Expanded View [file EMBR-24-e57300-s011.zip › Fig EV4/EV4C/non-treated_Merge.tif]

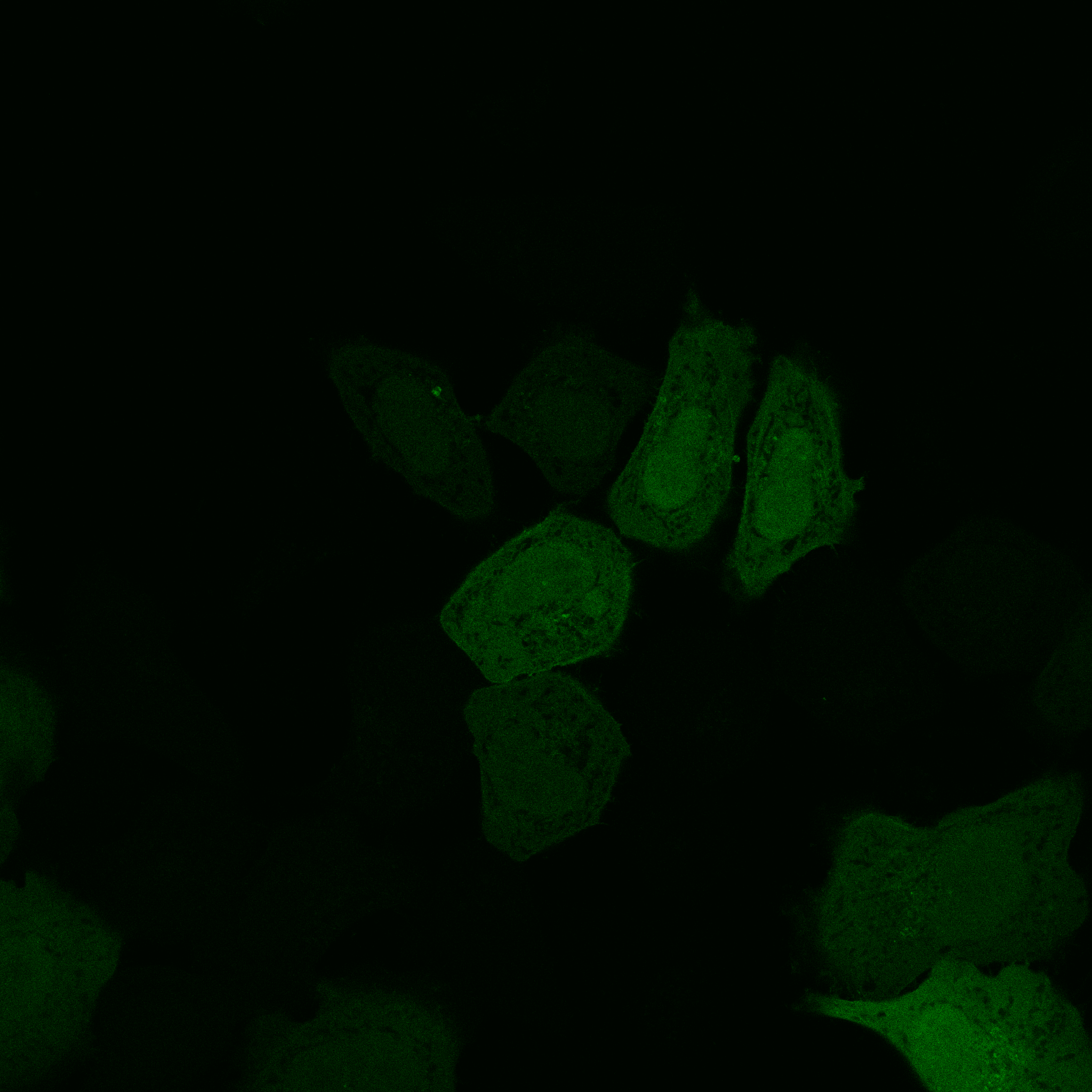

Supplement: Supplementary file 4 — Source Data for Expanded View [file EMBR-24-e57300-s011.zip › Fig EV4/EV4C/non-treated_mNG-STK38 K118R.tif]

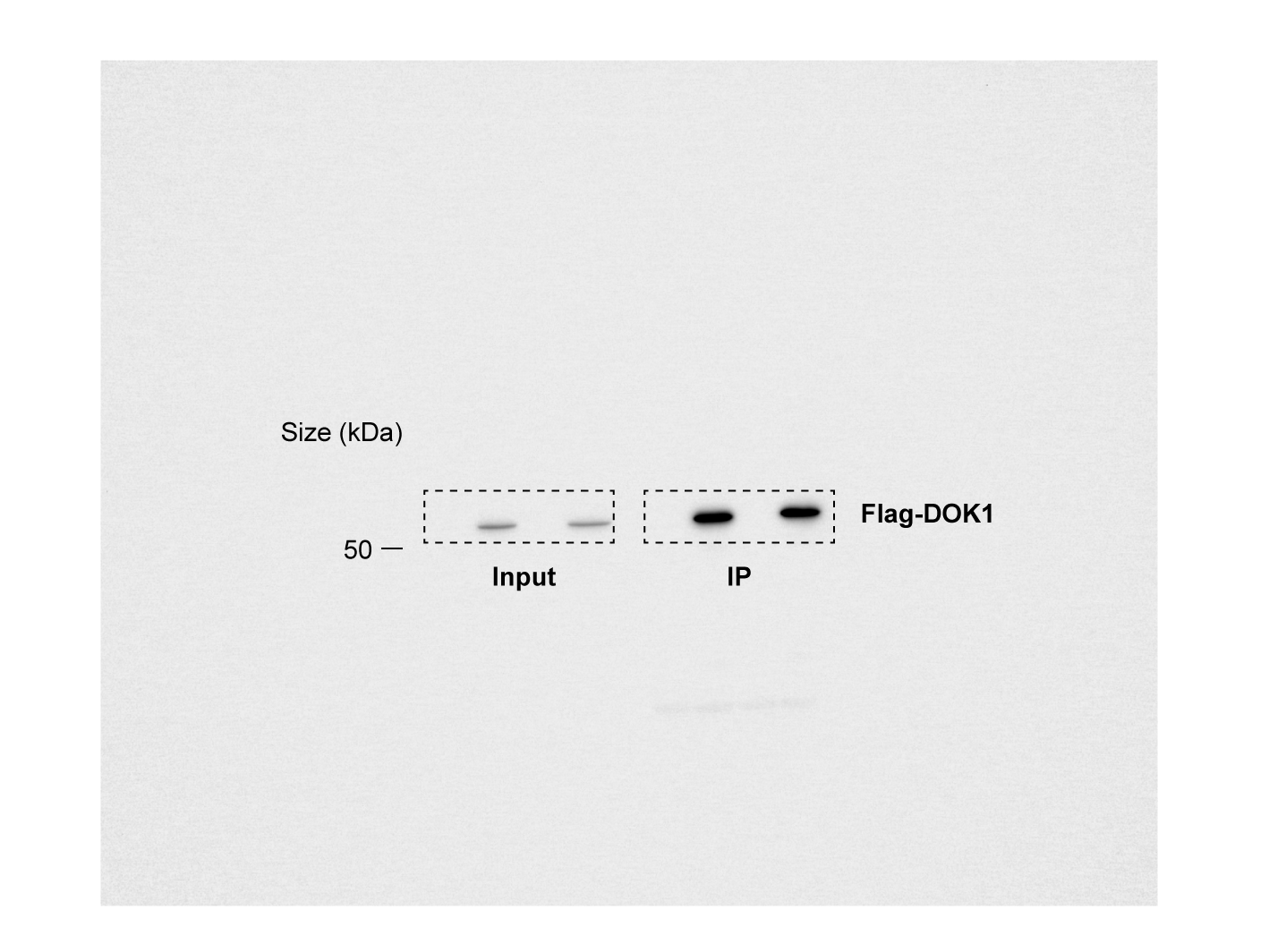

Supplement: Supplementary file 4 — Source Data for Expanded View [file EMBR-24-e57300-s011.zip › Fig EV4/EV4E/western_Flag-DOK1.tif]

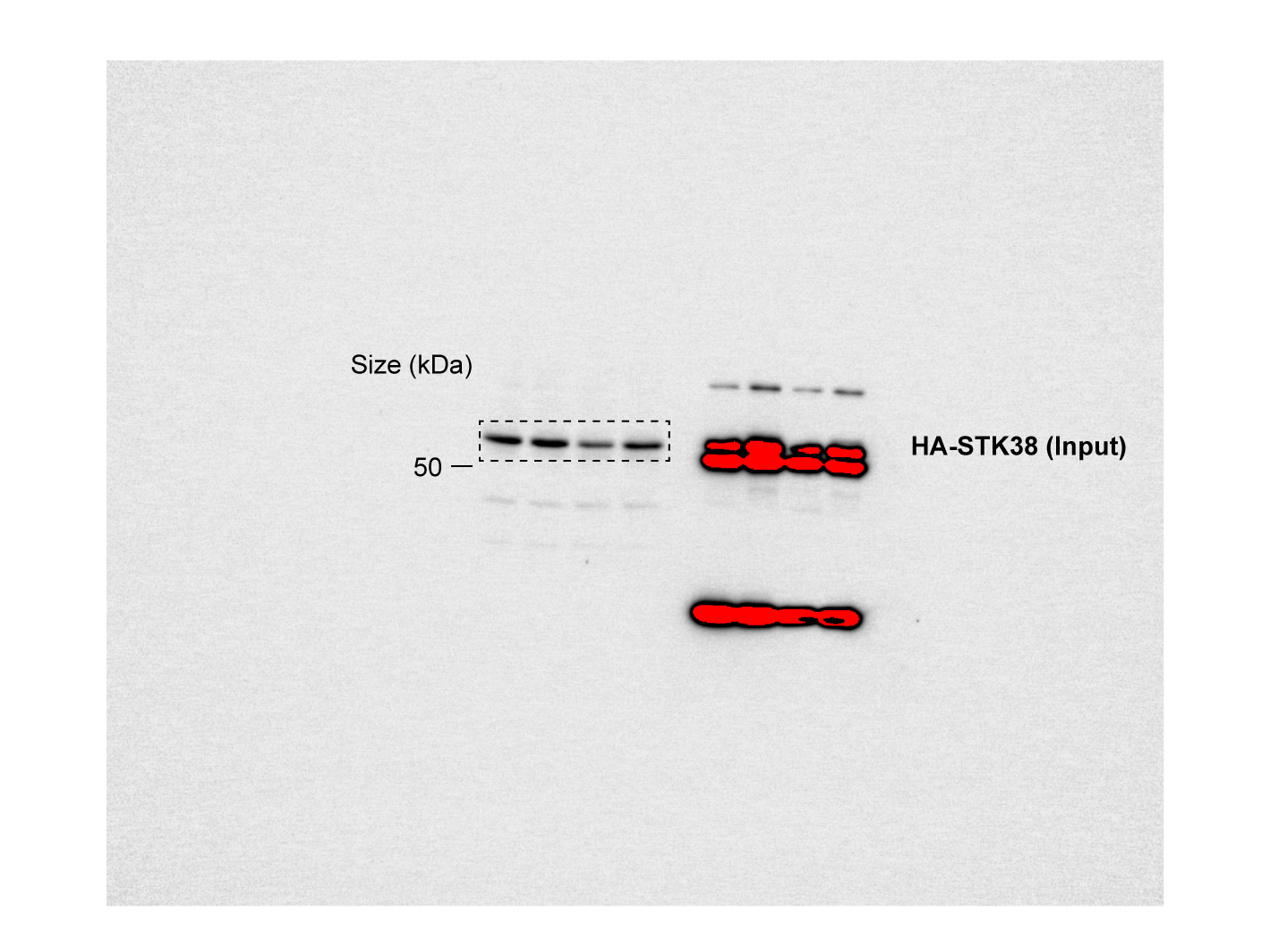

Supplement: Supplementary file 4 — Source Data for Expanded View [file EMBR-24-e57300-s011.zip › Fig EV4/EV4E/western_HA-STK38 (Input).tif]

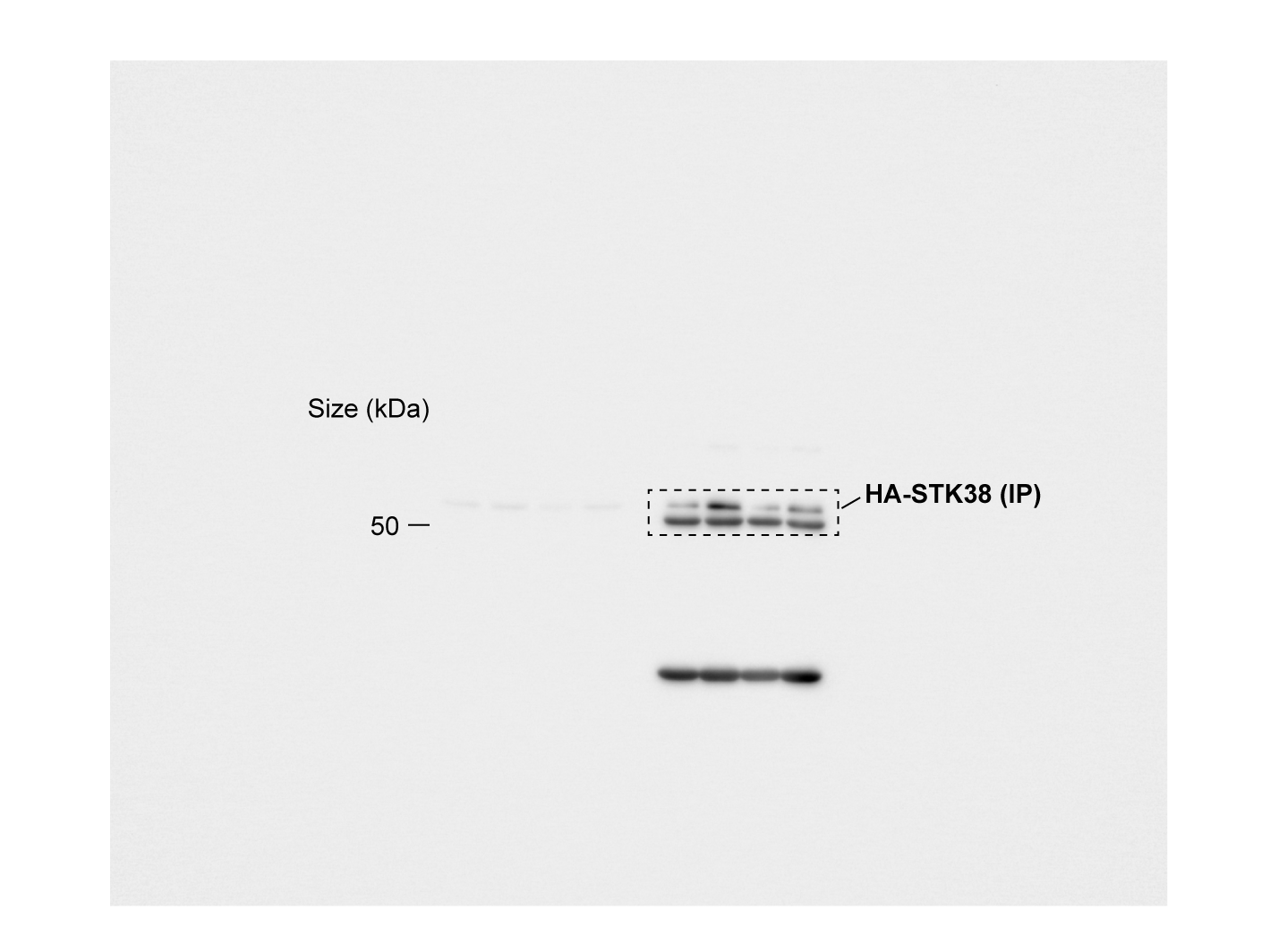

Supplement: Supplementary file 4 — Source Data for Expanded View [file EMBR-24-e57300-s011.zip › Fig EV4/EV4E/western_HA-STK38 (IP).tif]

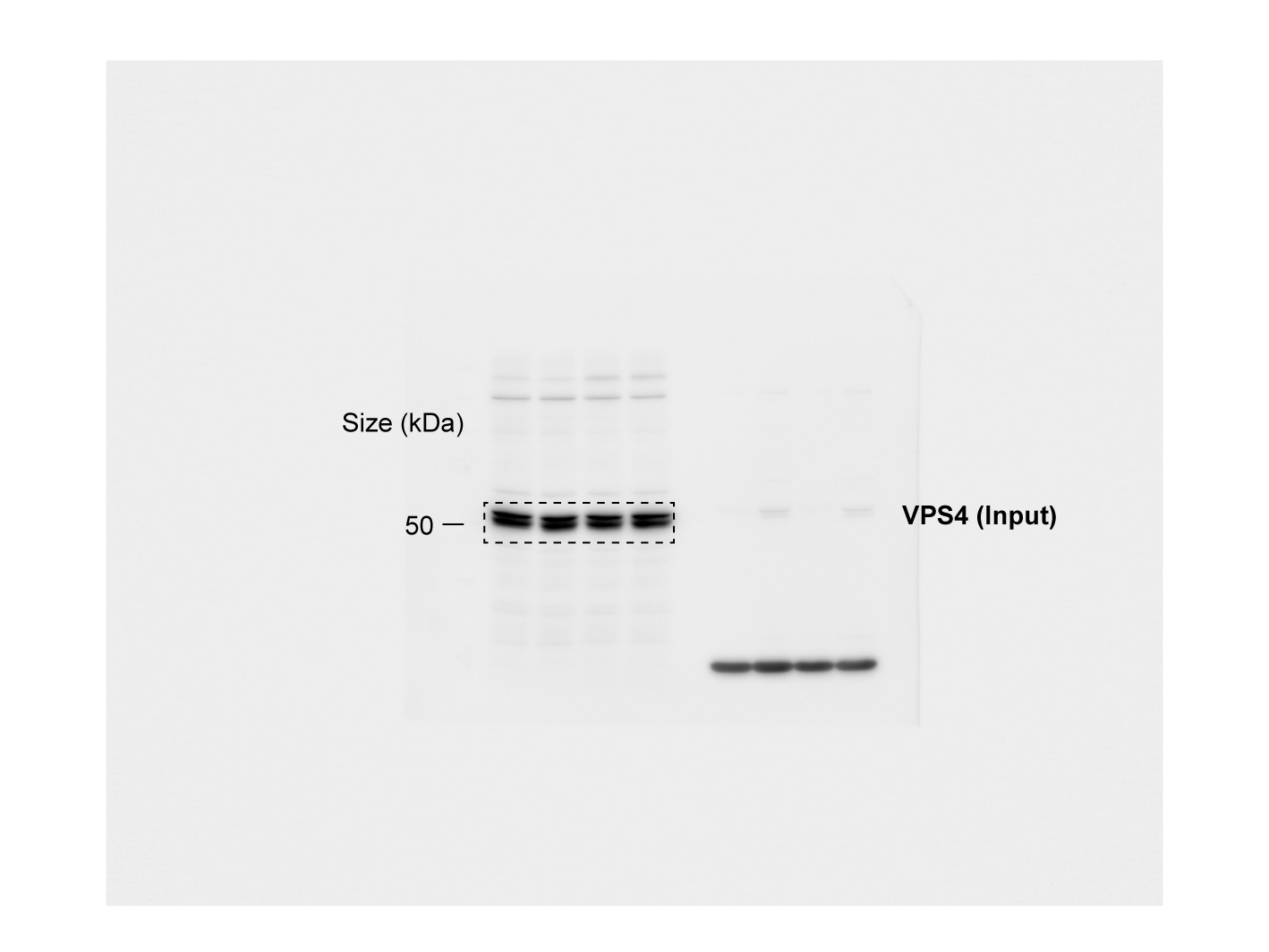

Supplement: Supplementary file 4 — Source Data for Expanded View [file EMBR-24-e57300-s011.zip › Fig EV4/EV4E/western_VPS4 (Input).tif]

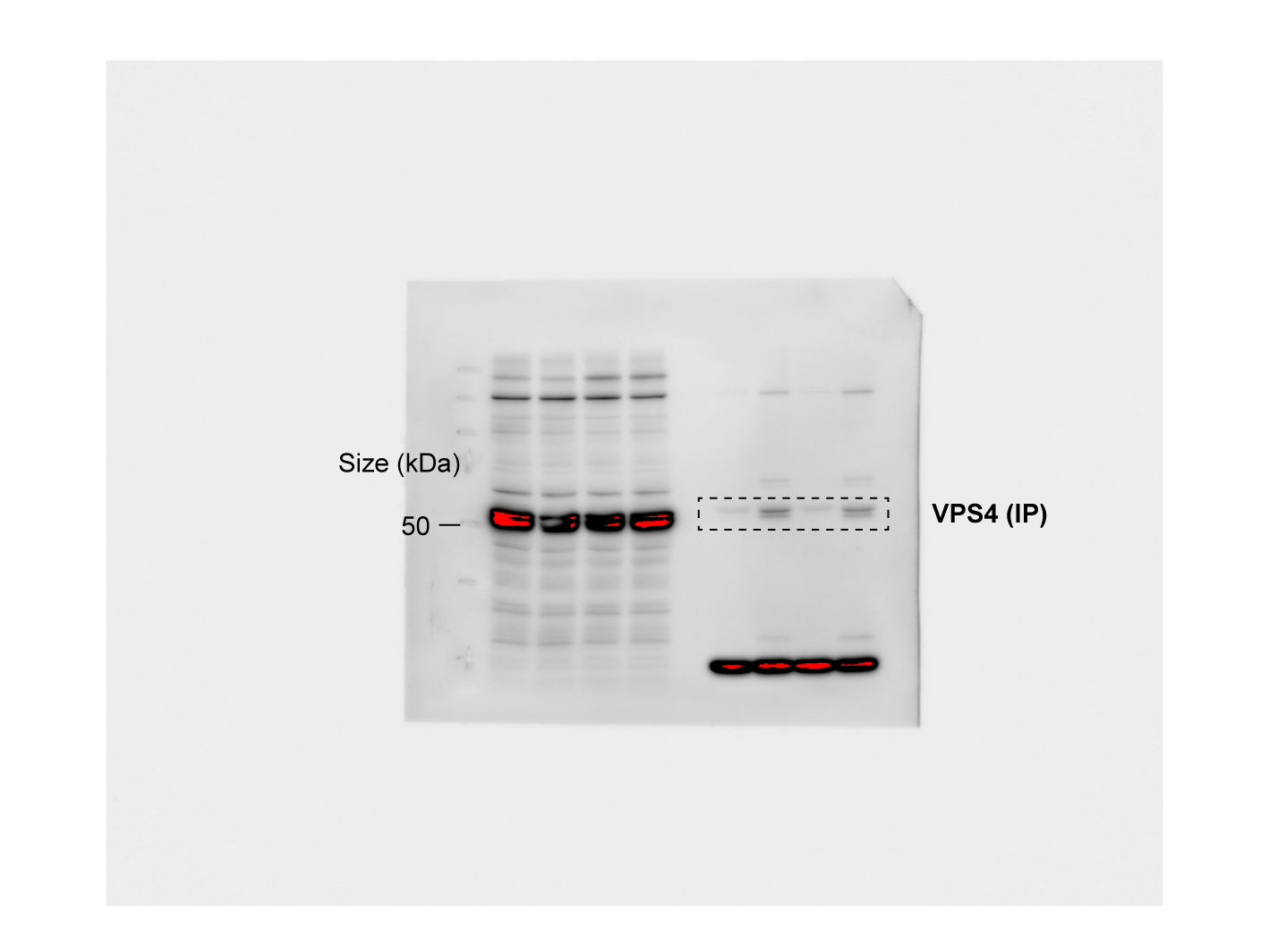

Supplement: Supplementary file 4 — Source Data for Expanded View [file EMBR-24-e57300-s011.zip › Fig EV4/EV4E/western_VPS4 (IP).tif]

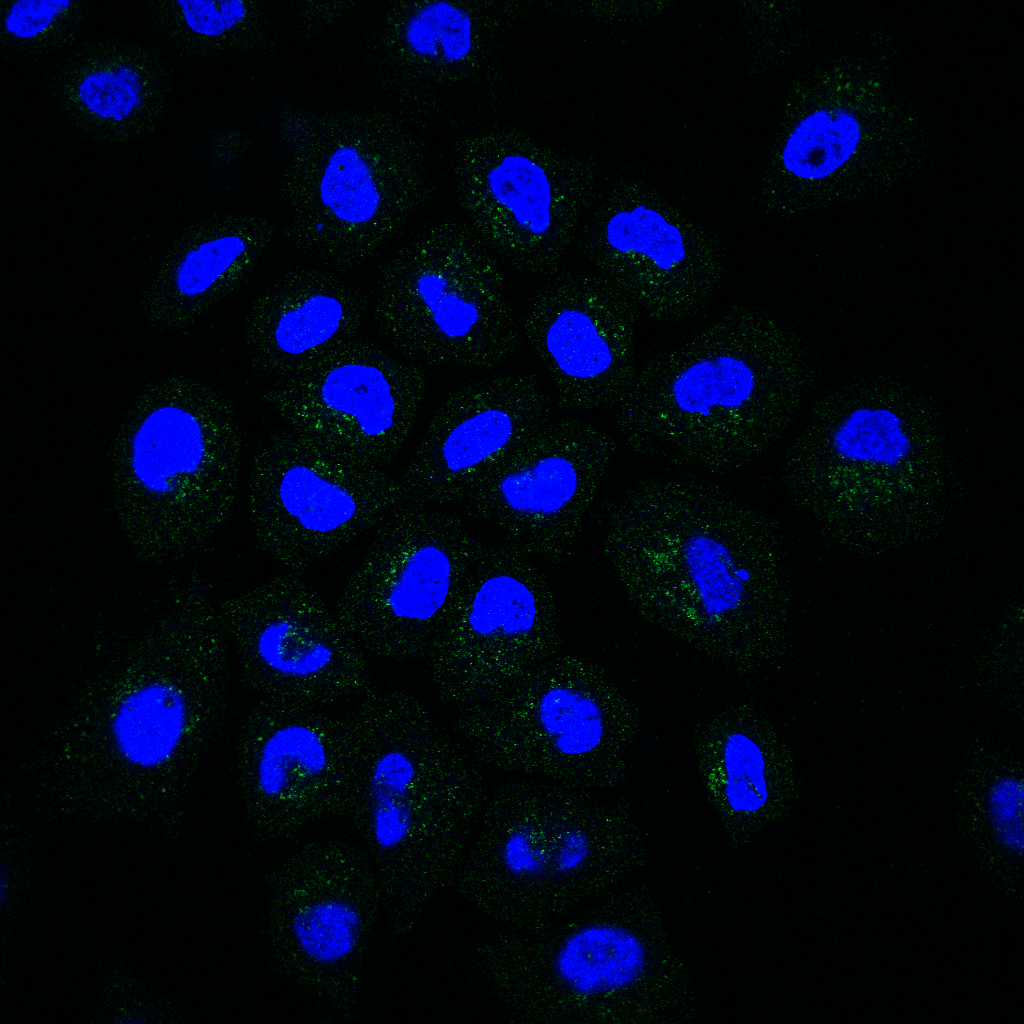

Supplement: Supplementary file 4 — Source Data for Expanded View [file EMBR-24-e57300-s011.zip › Fig EV5/EV5A/ATG16L1 KO_LLOMe.tif]

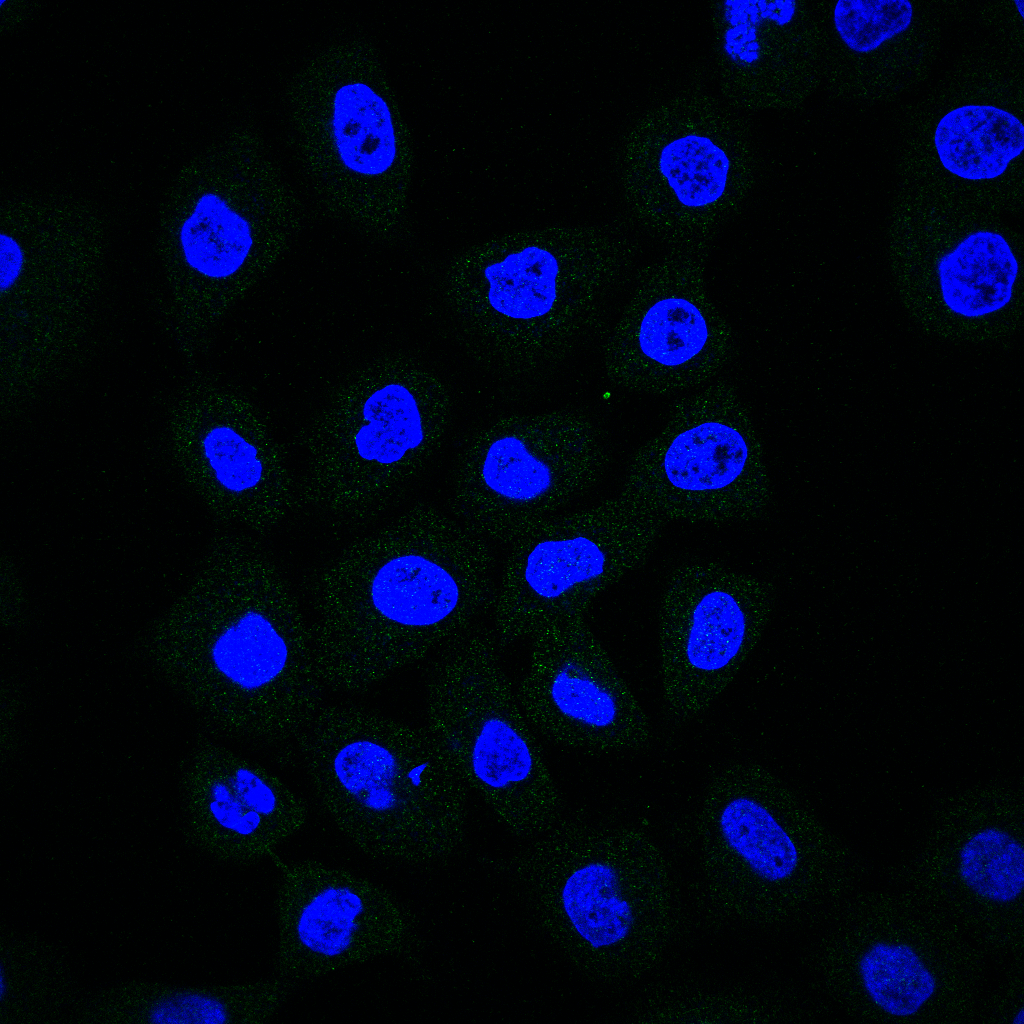

Supplement: Supplementary file 4 — Source Data for Expanded View [file EMBR-24-e57300-s011.zip › Fig EV5/EV5A/ATG16L1 KO_non-treated.tif]

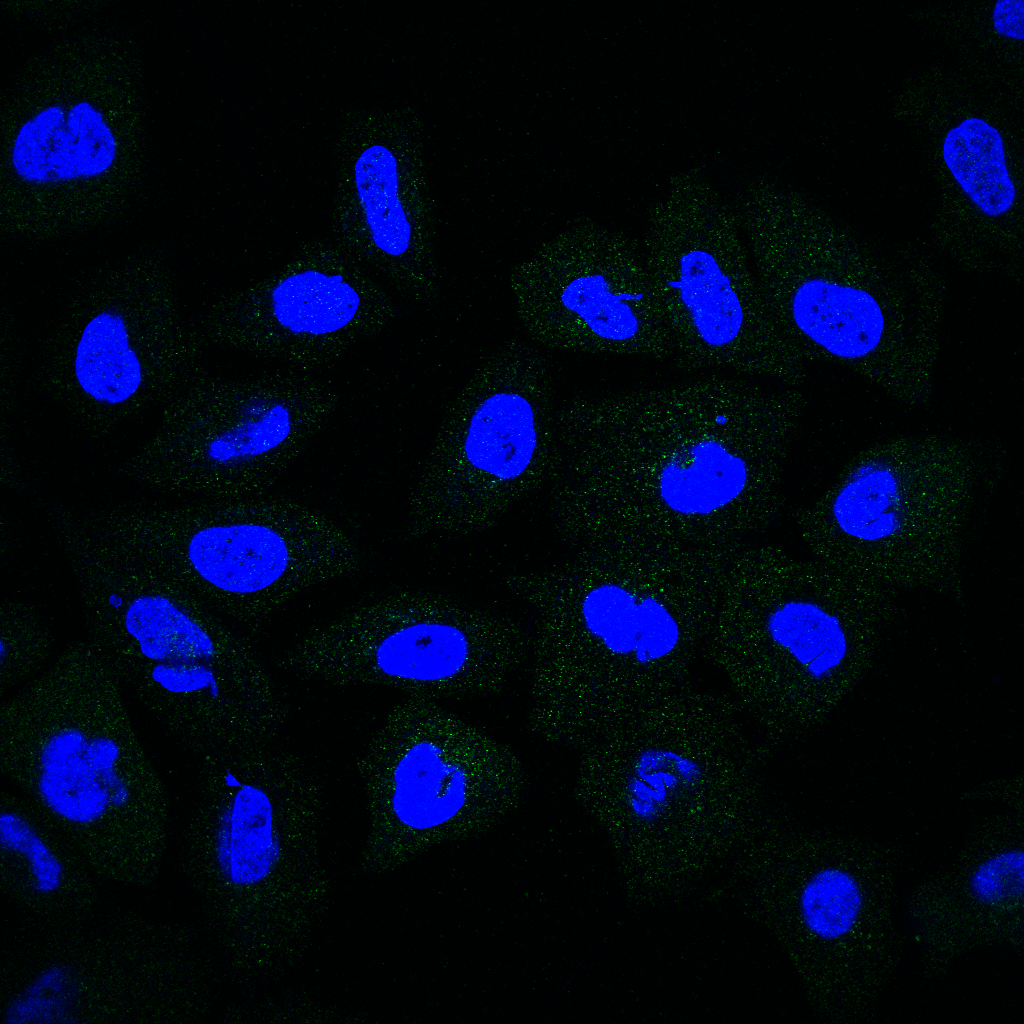

Supplement: Supplementary file 4 — Source Data for Expanded View [file EMBR-24-e57300-s011.zip › Fig EV5/EV5A/ATG3 KO_LLOMe.tif]

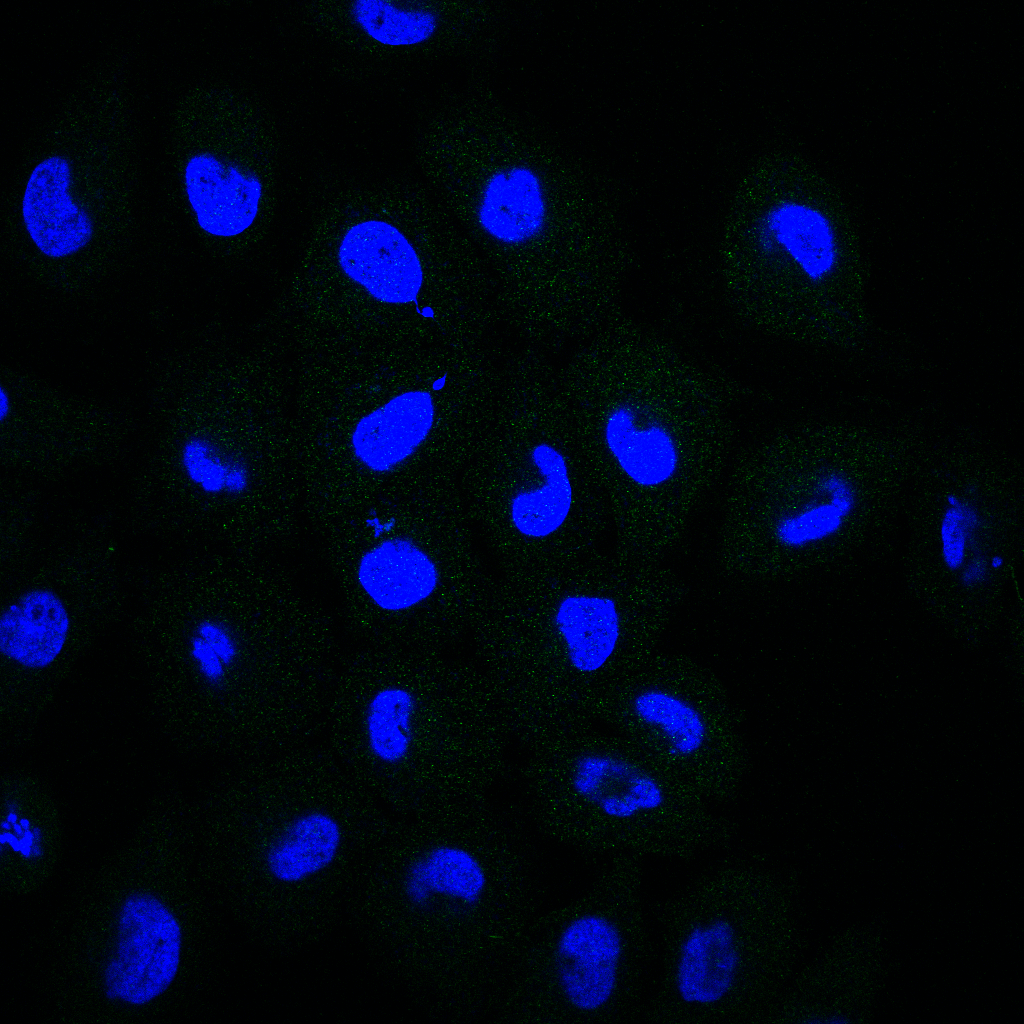

Supplement: Supplementary file 4 — Source Data for Expanded View [file EMBR-24-e57300-s011.zip › Fig EV5/EV5A/ATG3 KO_non-treated.tif]

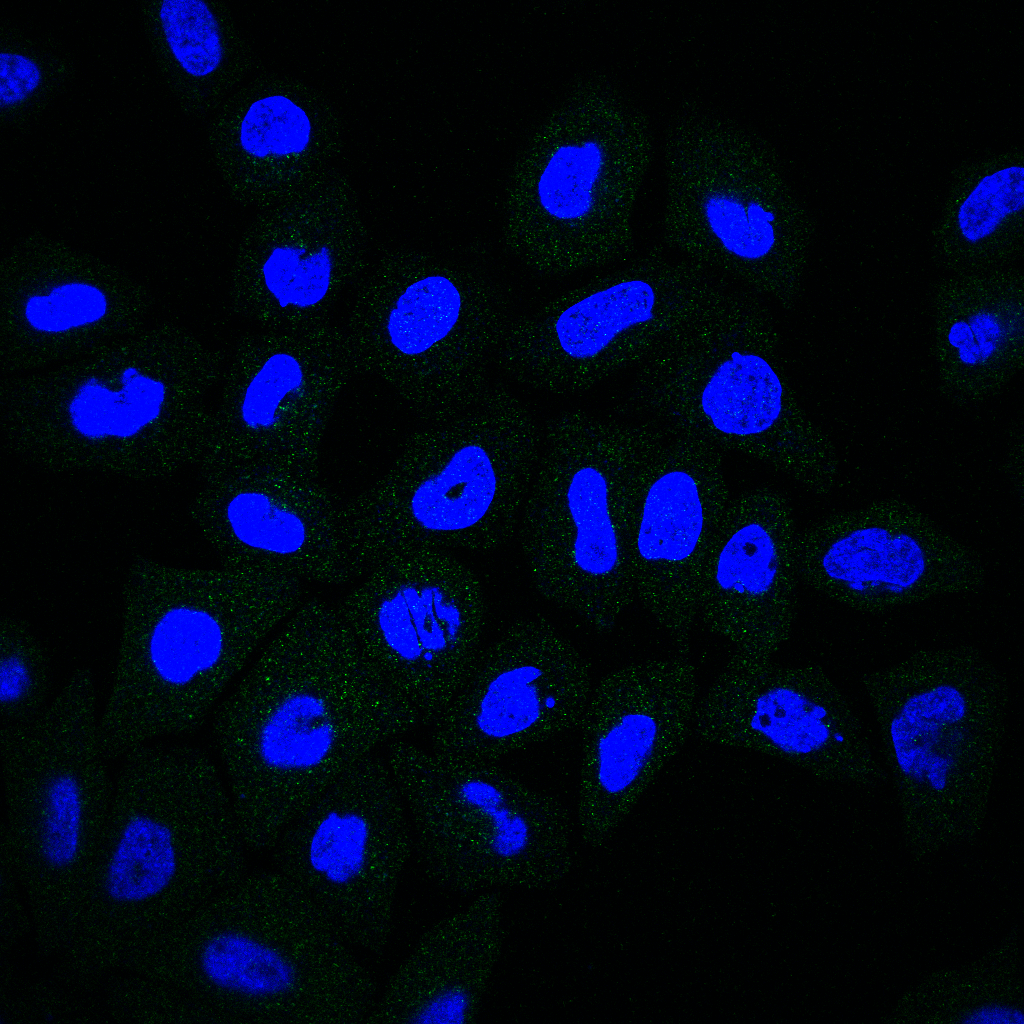

Supplement: Supplementary file 4 — Source Data for Expanded View [file EMBR-24-e57300-s011.zip › Fig EV5/EV5A/ATG7 KO_LLOMe.tif]

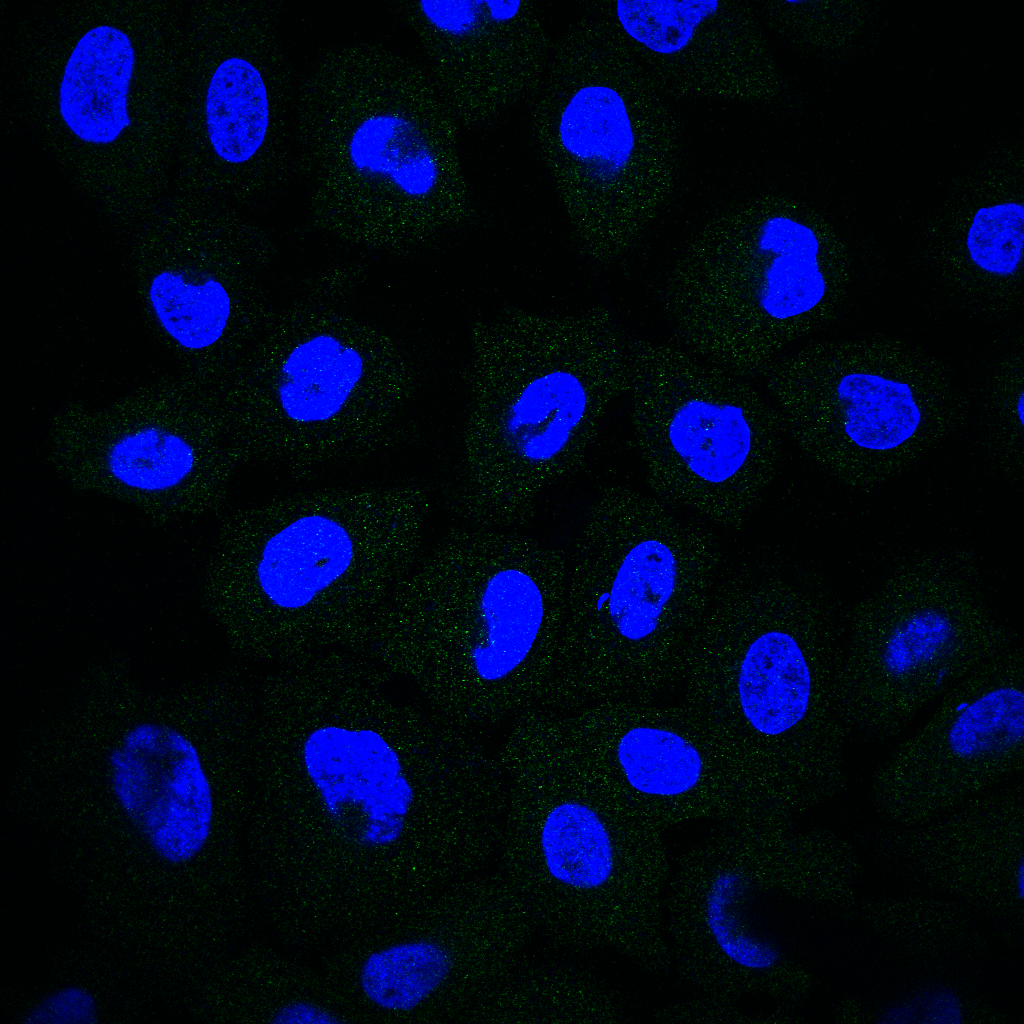

Supplement: Supplementary file 4 — Source Data for Expanded View [file EMBR-24-e57300-s011.zip › Fig EV5/EV5A/ATG7 KO_non-treated.tif]

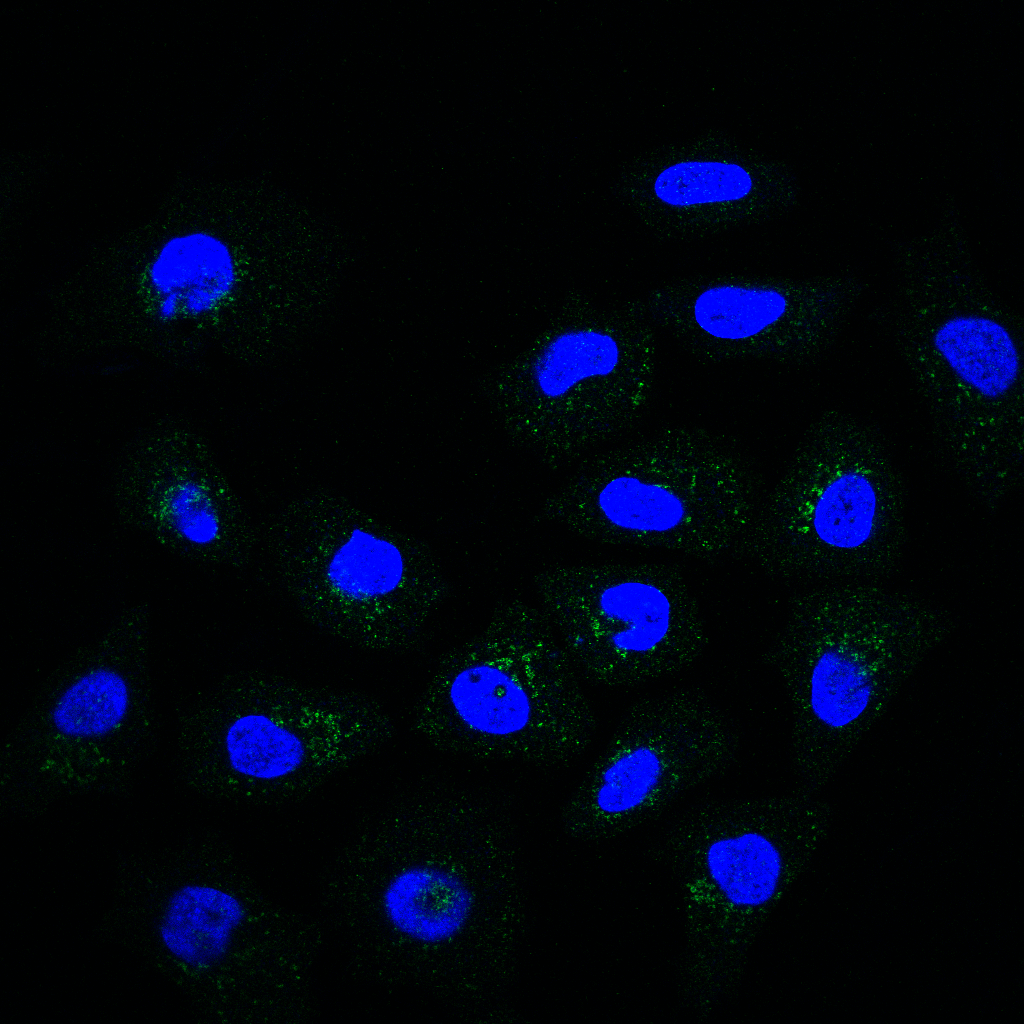

Supplement: Supplementary file 4 — Source Data for Expanded View [file EMBR-24-e57300-s011.zip › Fig EV5/EV5A/FIP200 KO_LLOMe.tif]

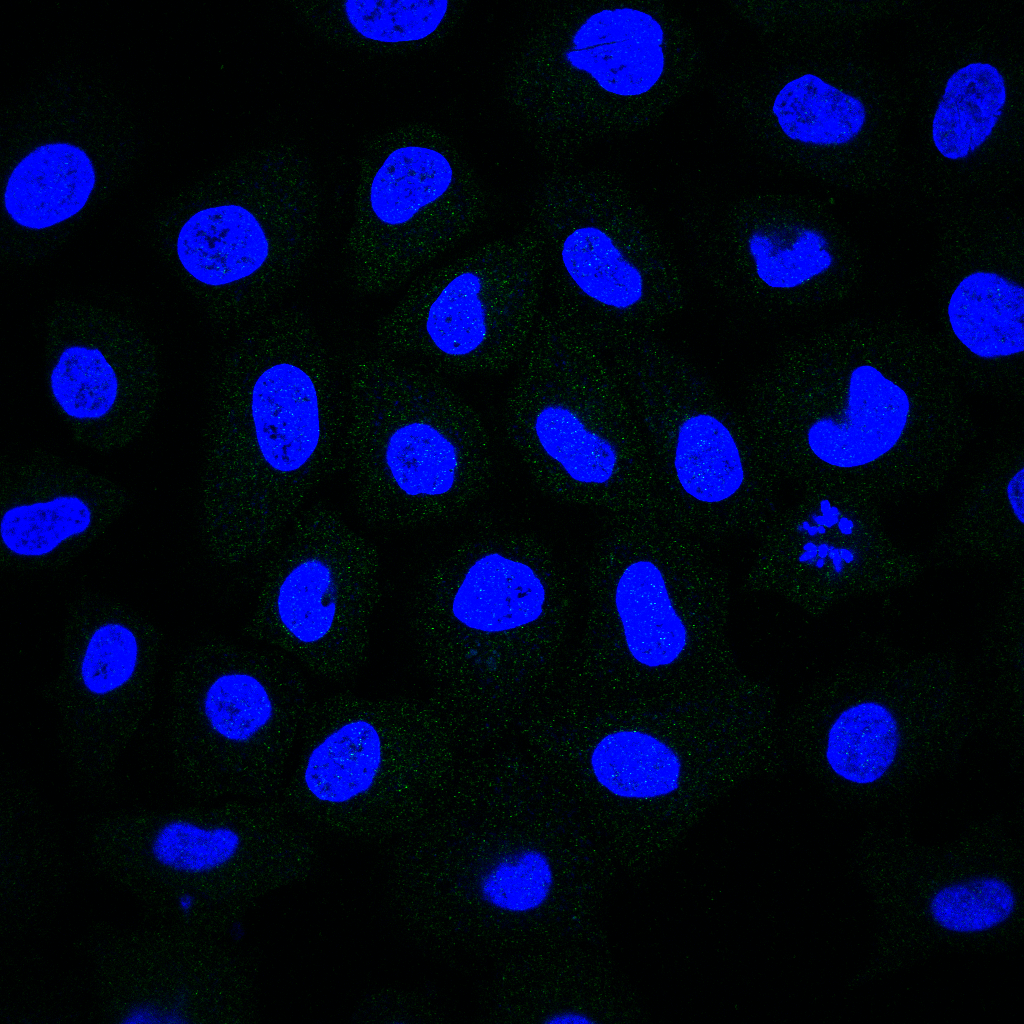

Supplement: Supplementary file 4 — Source Data for Expanded View [file EMBR-24-e57300-s011.zip › Fig EV5/EV5A/FIP200 KO_non-treated.tif]

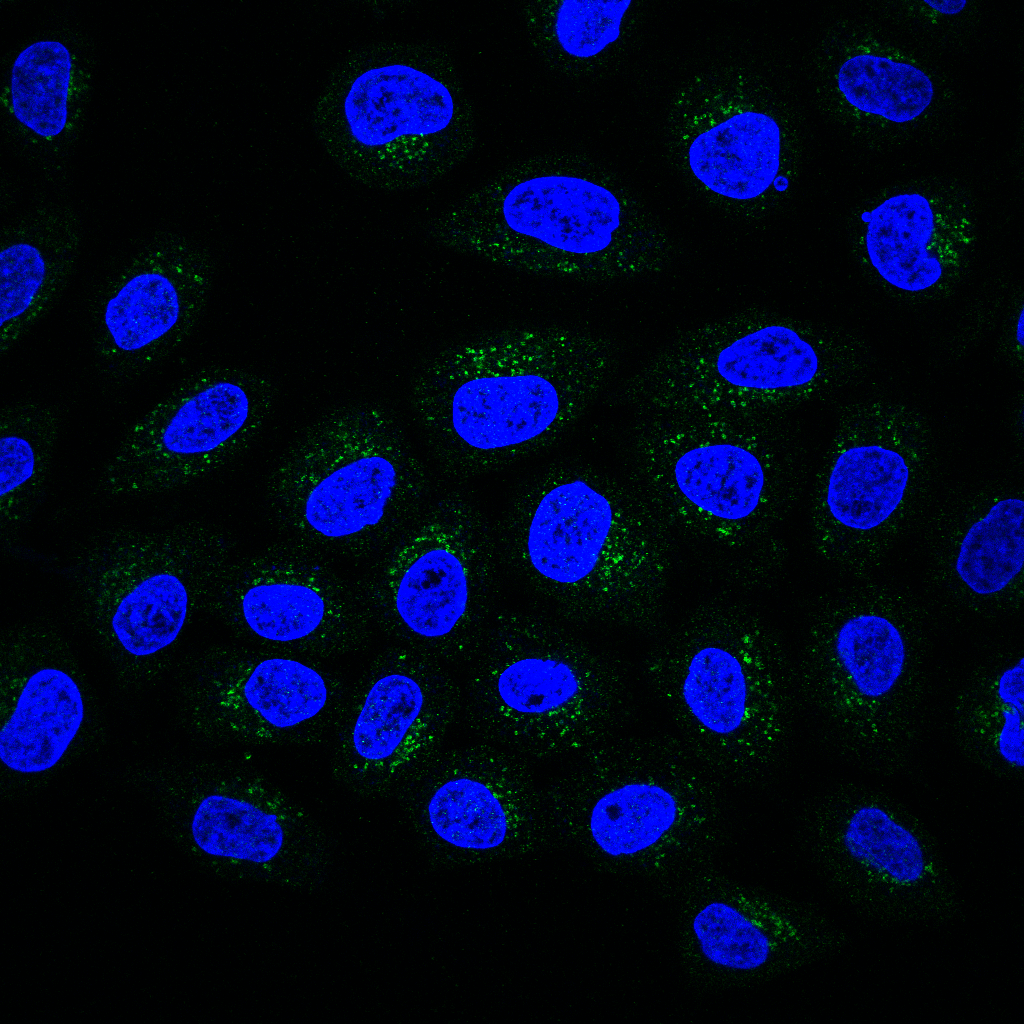

Supplement: Supplementary file 4 — Source Data for Expanded View [file EMBR-24-e57300-s011.zip › Fig EV5/EV5A/WT_LLOMe.tif]

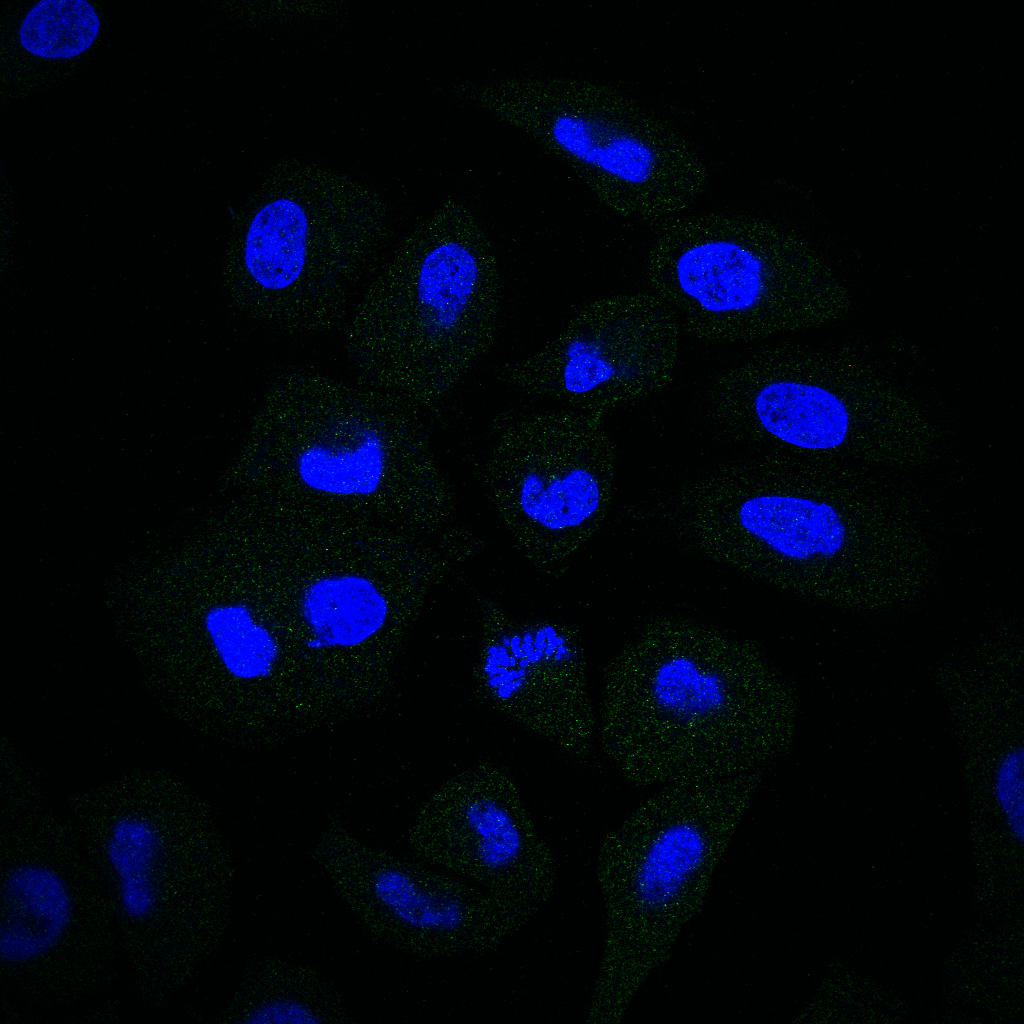

Supplement: Supplementary file 4 — Source Data for Expanded View [file EMBR-24-e57300-s011.zip › Fig EV5/EV5A/WT_non-treated.tif]

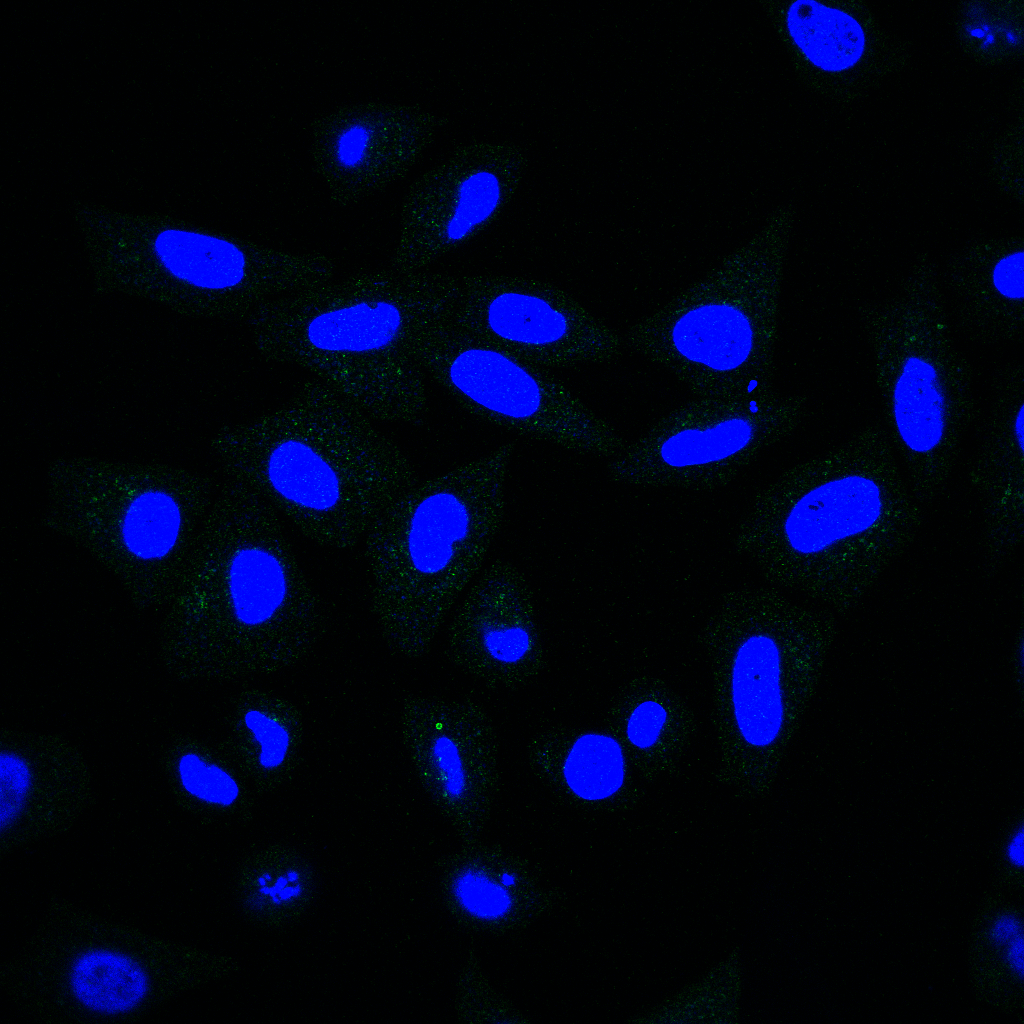

Supplement: Supplementary file 4 — Source Data for Expanded View [file EMBR-24-e57300-s011.zip › Fig EV5/EV5C/GABARAP TKO_LLOMe.tif]

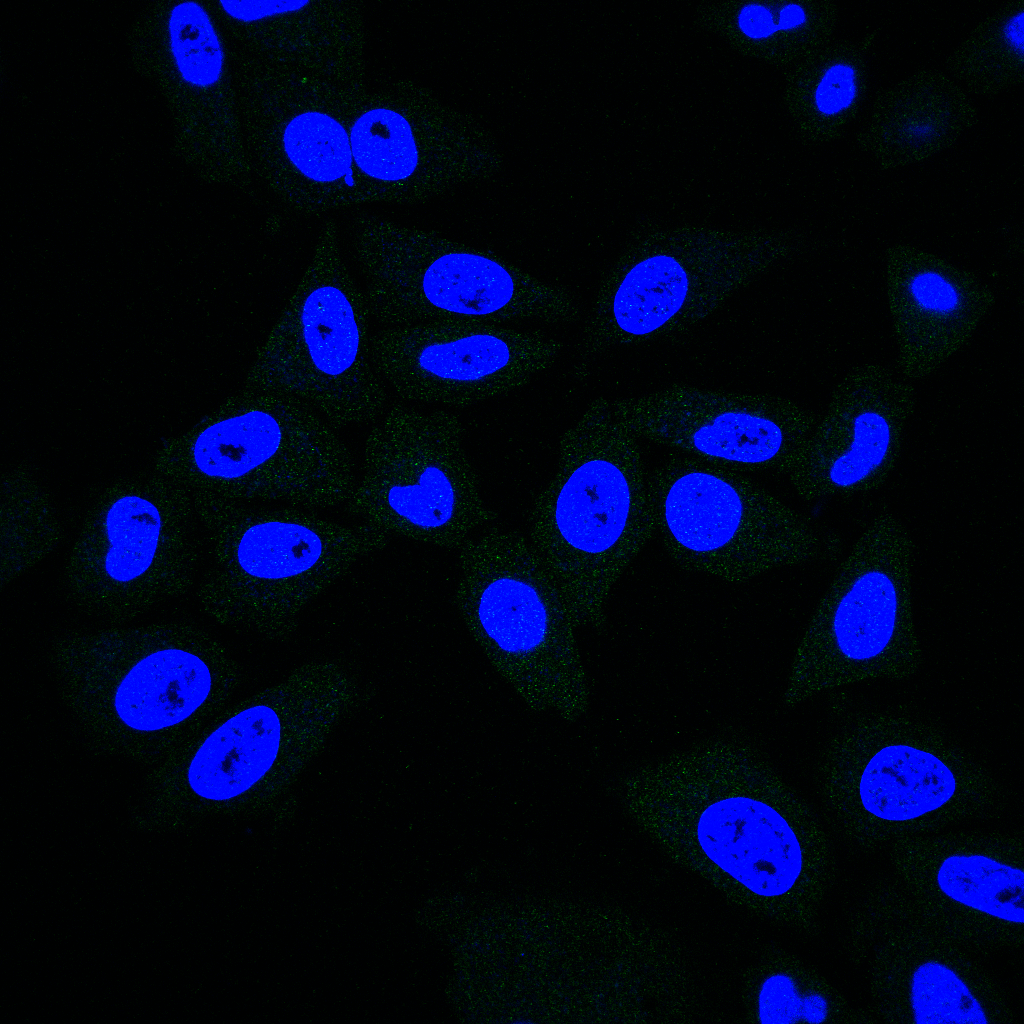

Supplement: Supplementary file 4 — Source Data for Expanded View [file EMBR-24-e57300-s011.zip › Fig EV5/EV5C/GABARAP TKO_non-treated.tif]

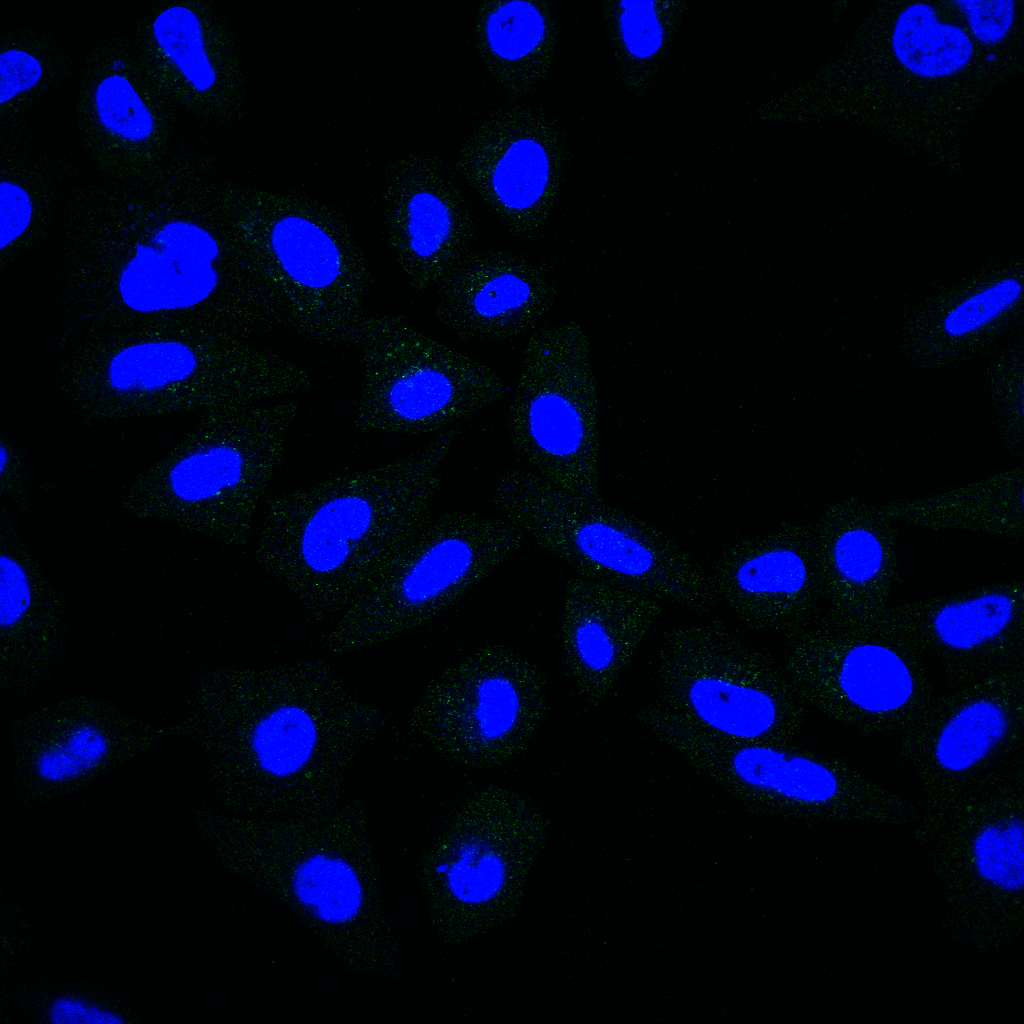

Supplement: Supplementary file 4 — Source Data for Expanded View [file EMBR-24-e57300-s011.zip › Fig EV5/EV5C/hexa KO_LLOMe.tif]

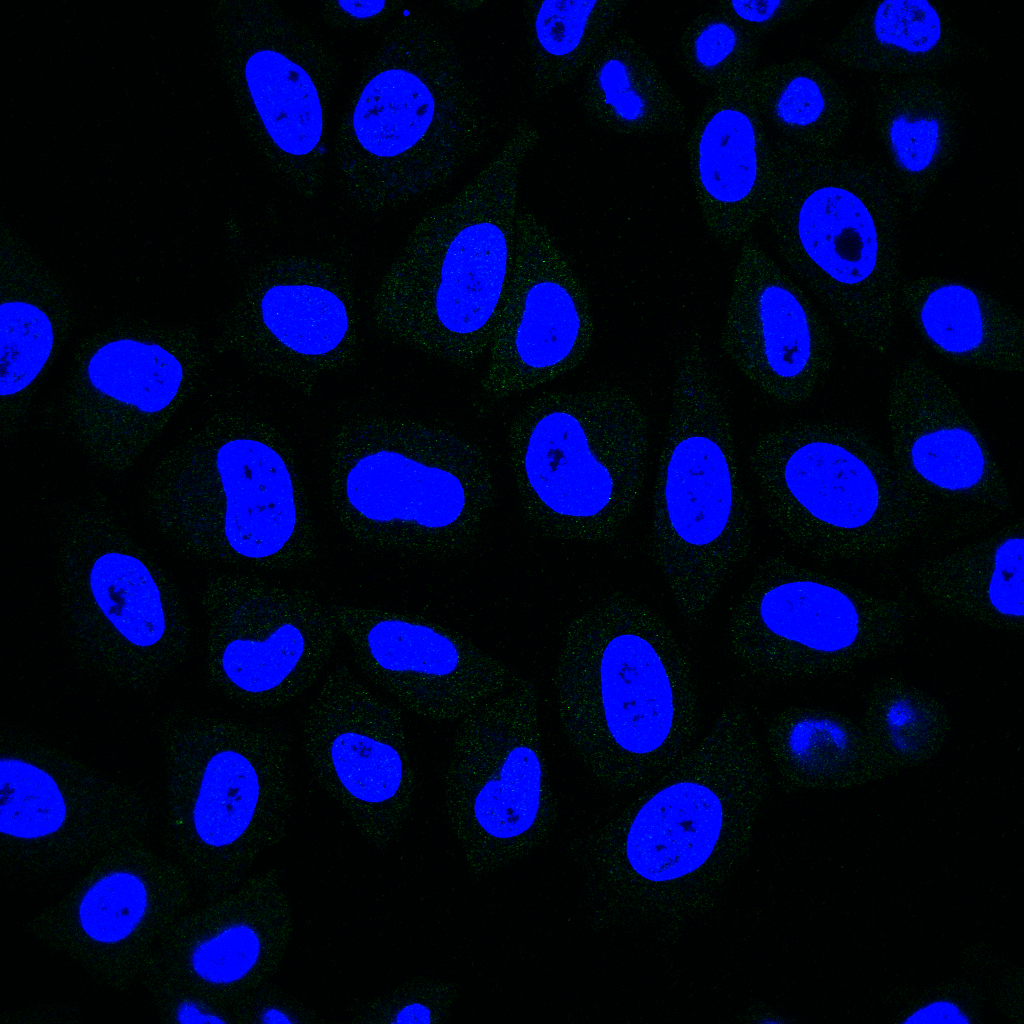

Supplement: Supplementary file 4 — Source Data for Expanded View [file EMBR-24-e57300-s011.zip › Fig EV5/EV5C/hexa KO_non-treated.tif]

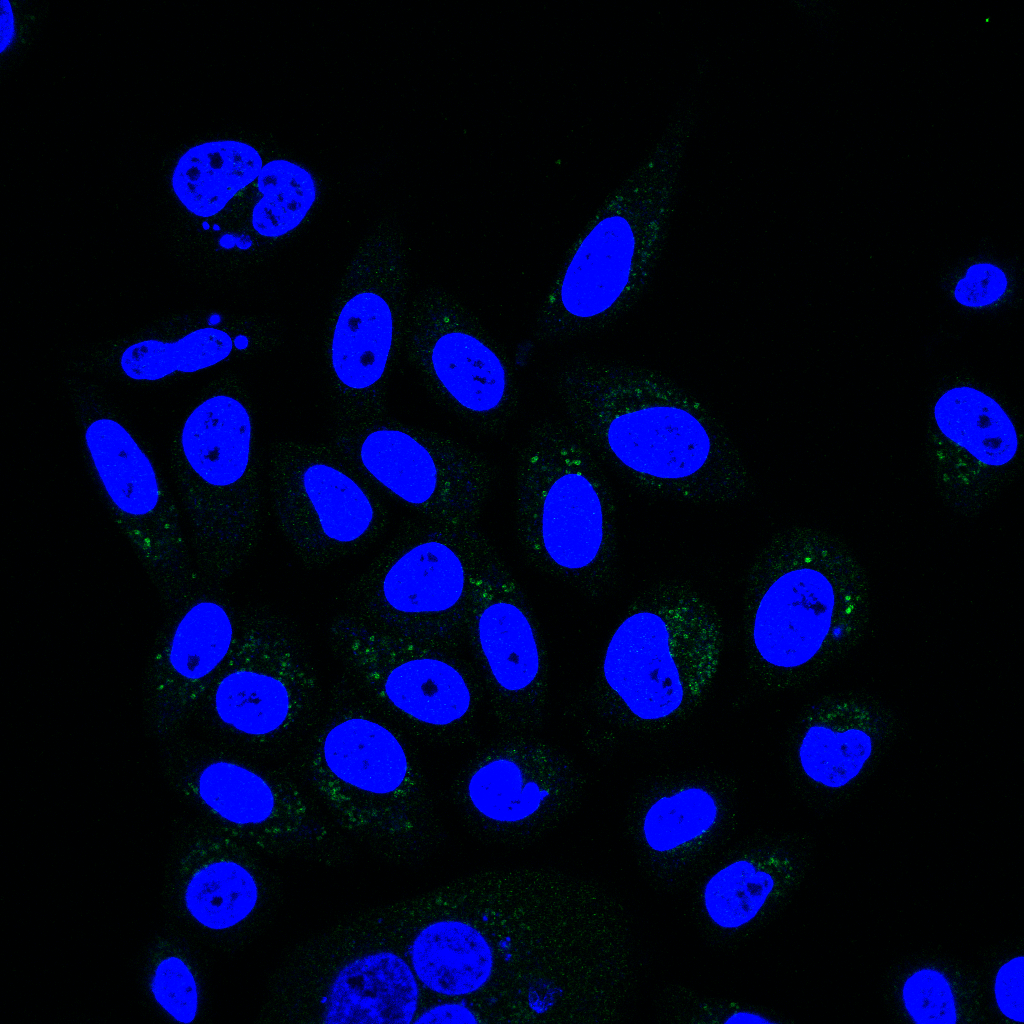

Supplement: Supplementary file 4 — Source Data for Expanded View [file EMBR-24-e57300-s011.zip › Fig EV5/EV5C/LC3 TKO_LLOMe.tif]

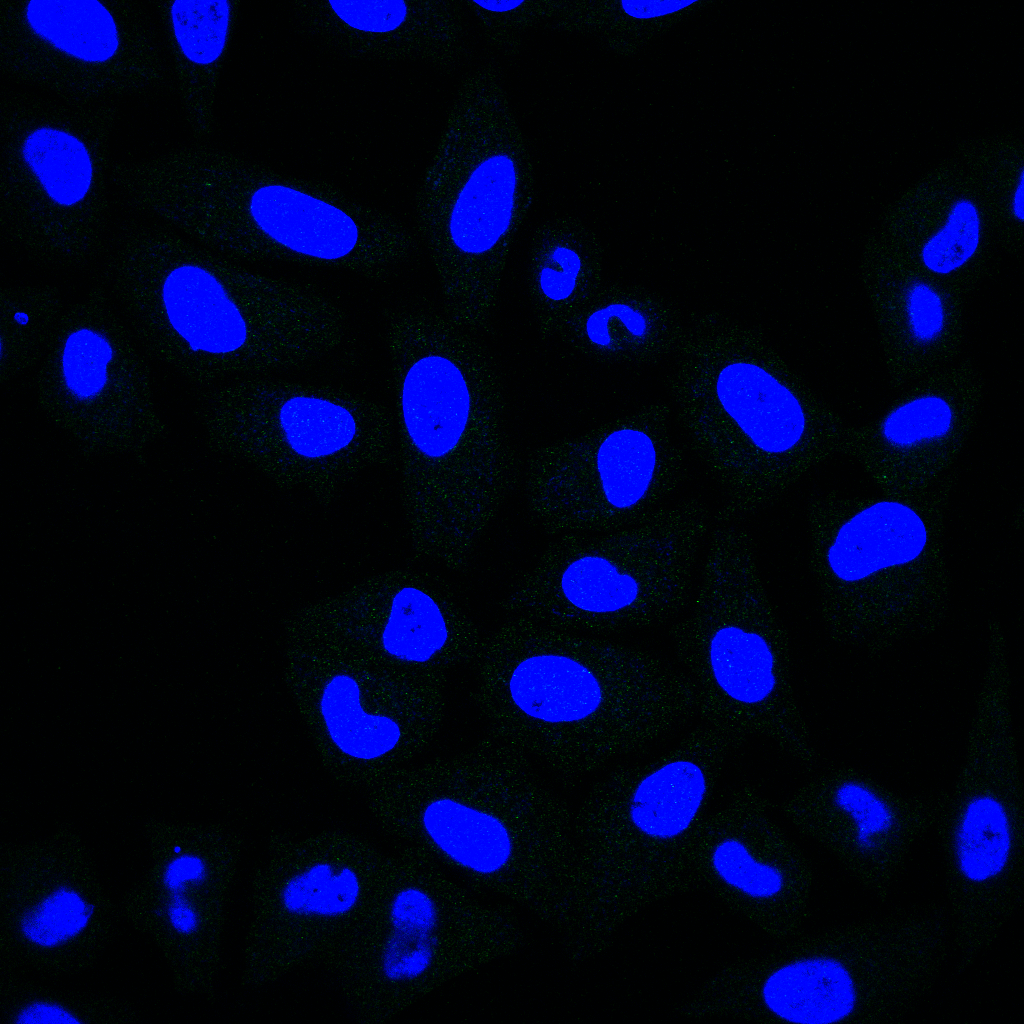

Supplement: Supplementary file 4 — Source Data for Expanded View [file EMBR-24-e57300-s011.zip › Fig EV5/EV5C/LC3 TKO_non-treated.tif]

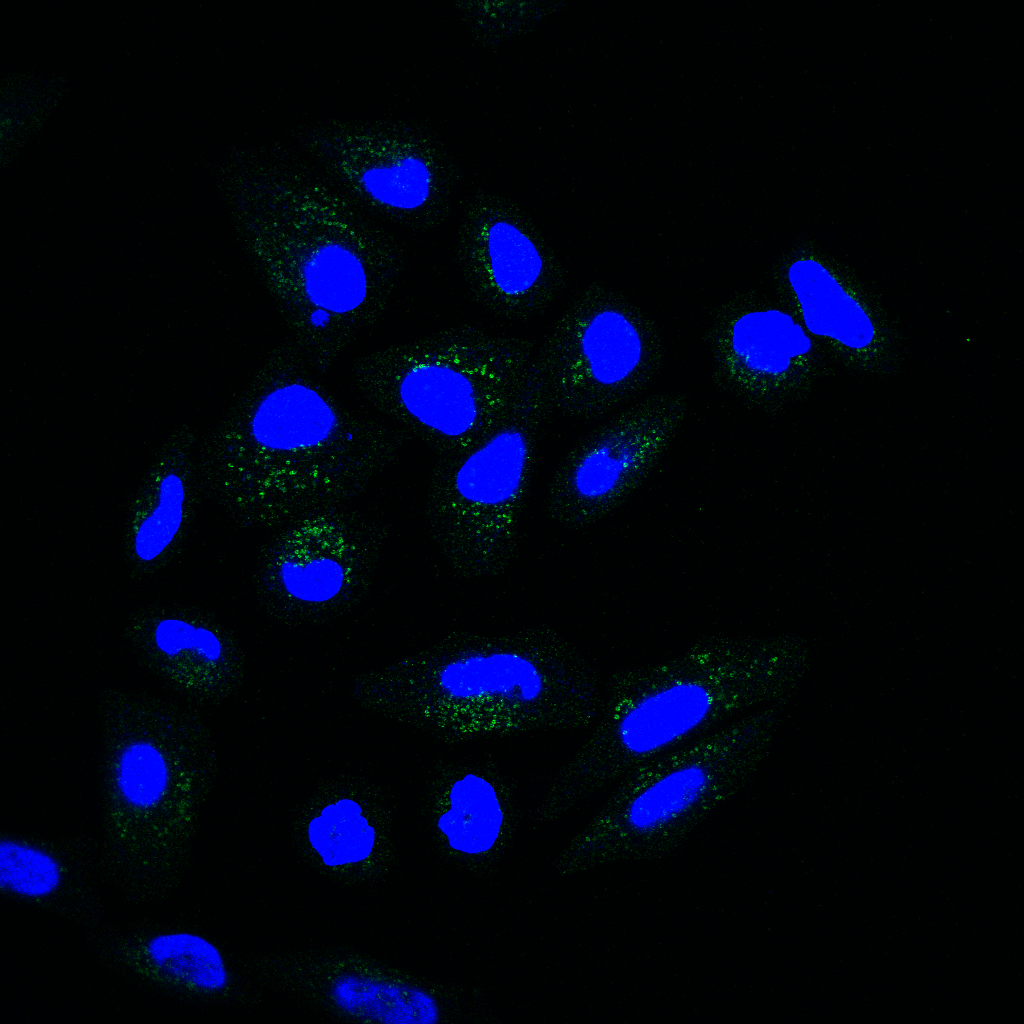

Supplement: Supplementary file 4 — Source Data for Expanded View [file EMBR-24-e57300-s011.zip › Fig EV5/EV5C/WT_LLOMe.tif]

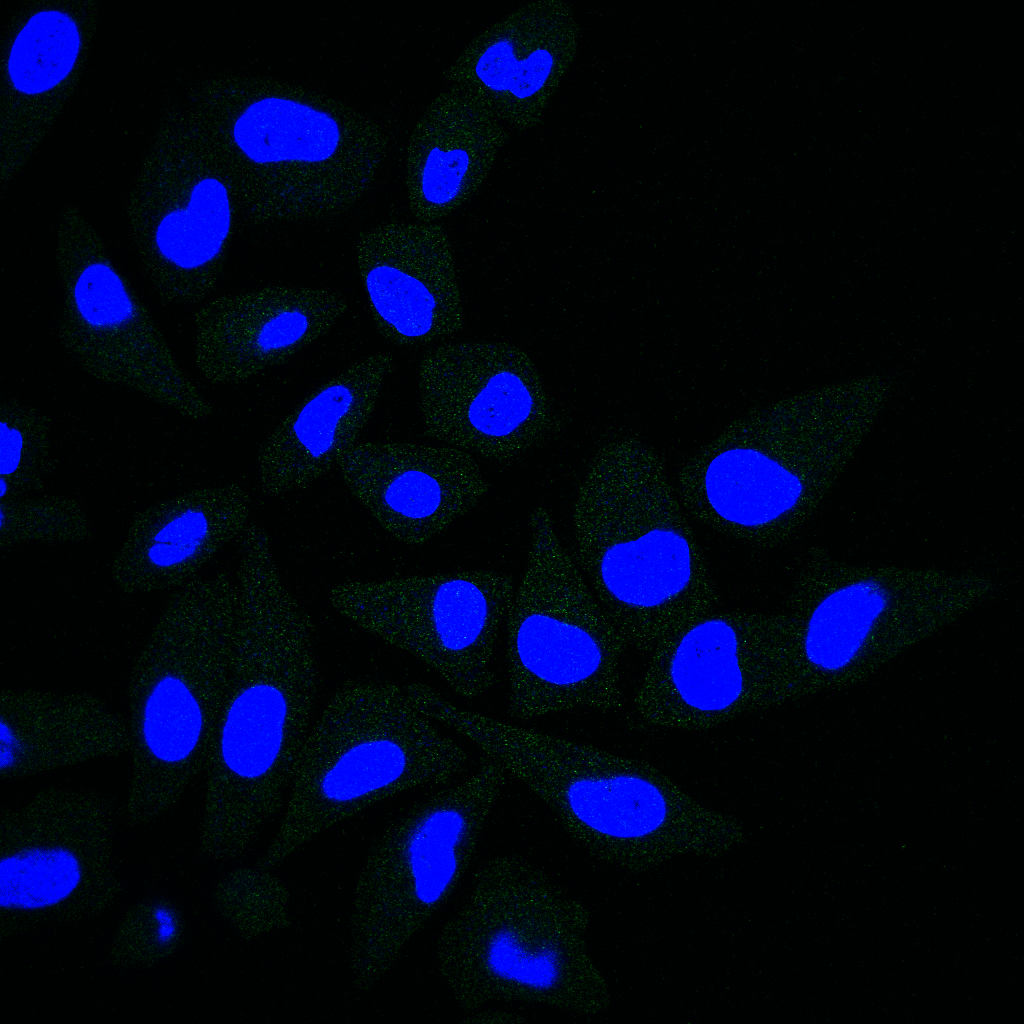

Supplement: Supplementary file 4 — Source Data for Expanded View [file EMBR-24-e57300-s011.zip › Fig EV5/EV5C/WT_non-treated.tif]

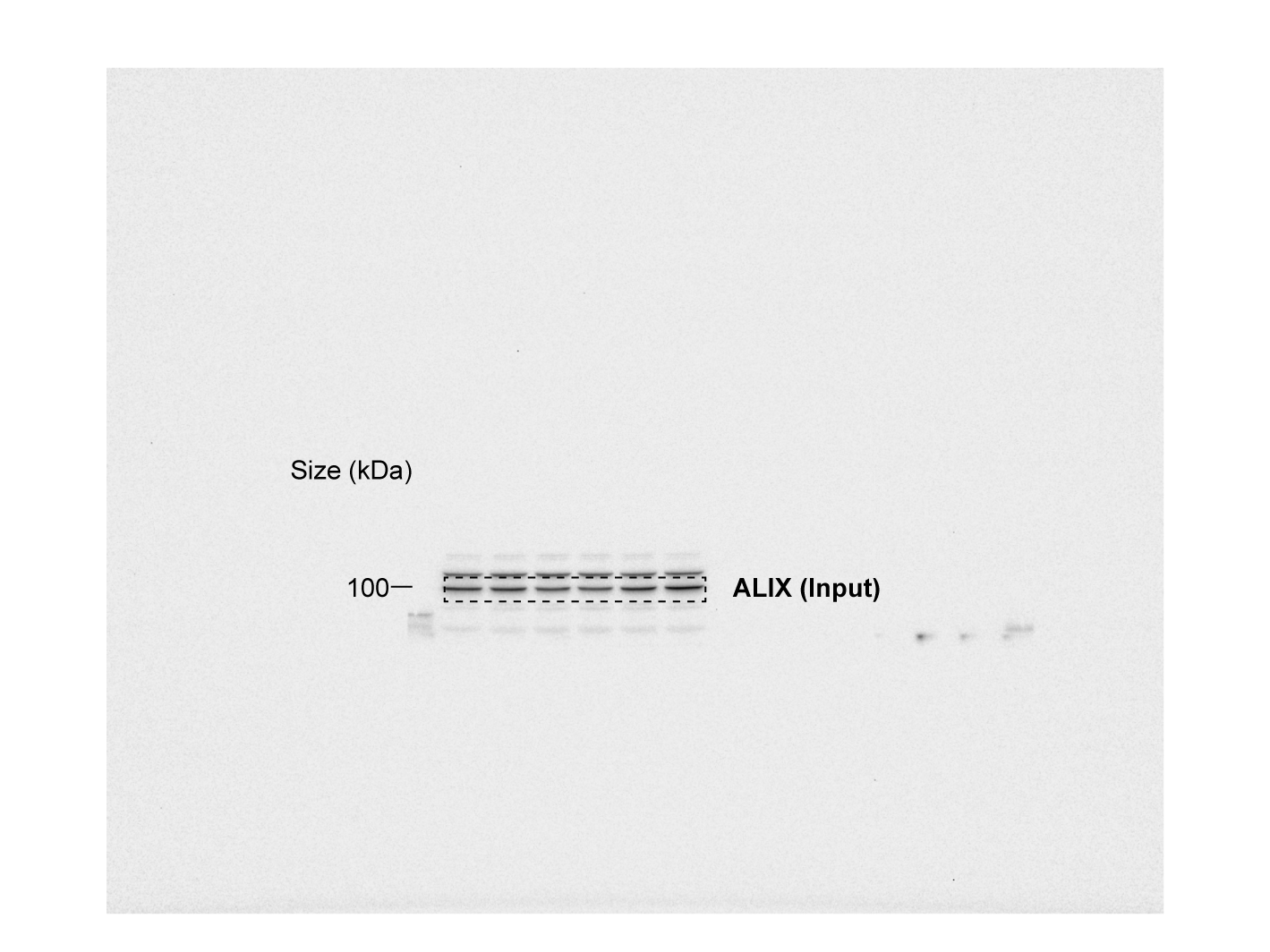

Supplement: Supplementary file 4 — Source Data for Expanded View [file EMBR-24-e57300-s011.zip › Fig EV5/EV5E/western_ALIX (Input).tif]

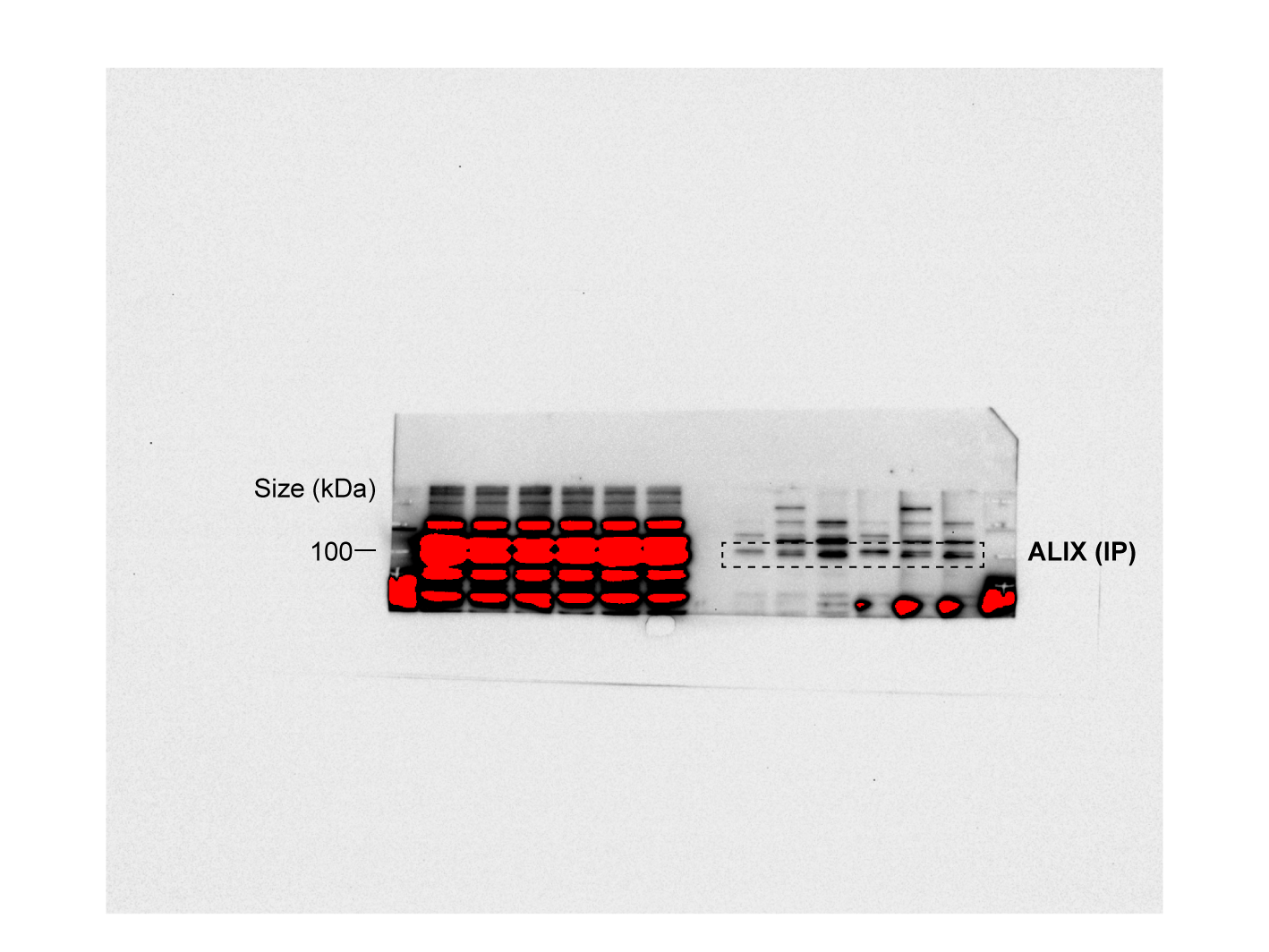

Supplement: Supplementary file 4 — Source Data for Expanded View [file EMBR-24-e57300-s011.zip › Fig EV5/EV5E/western_ALIX (IP).tif]

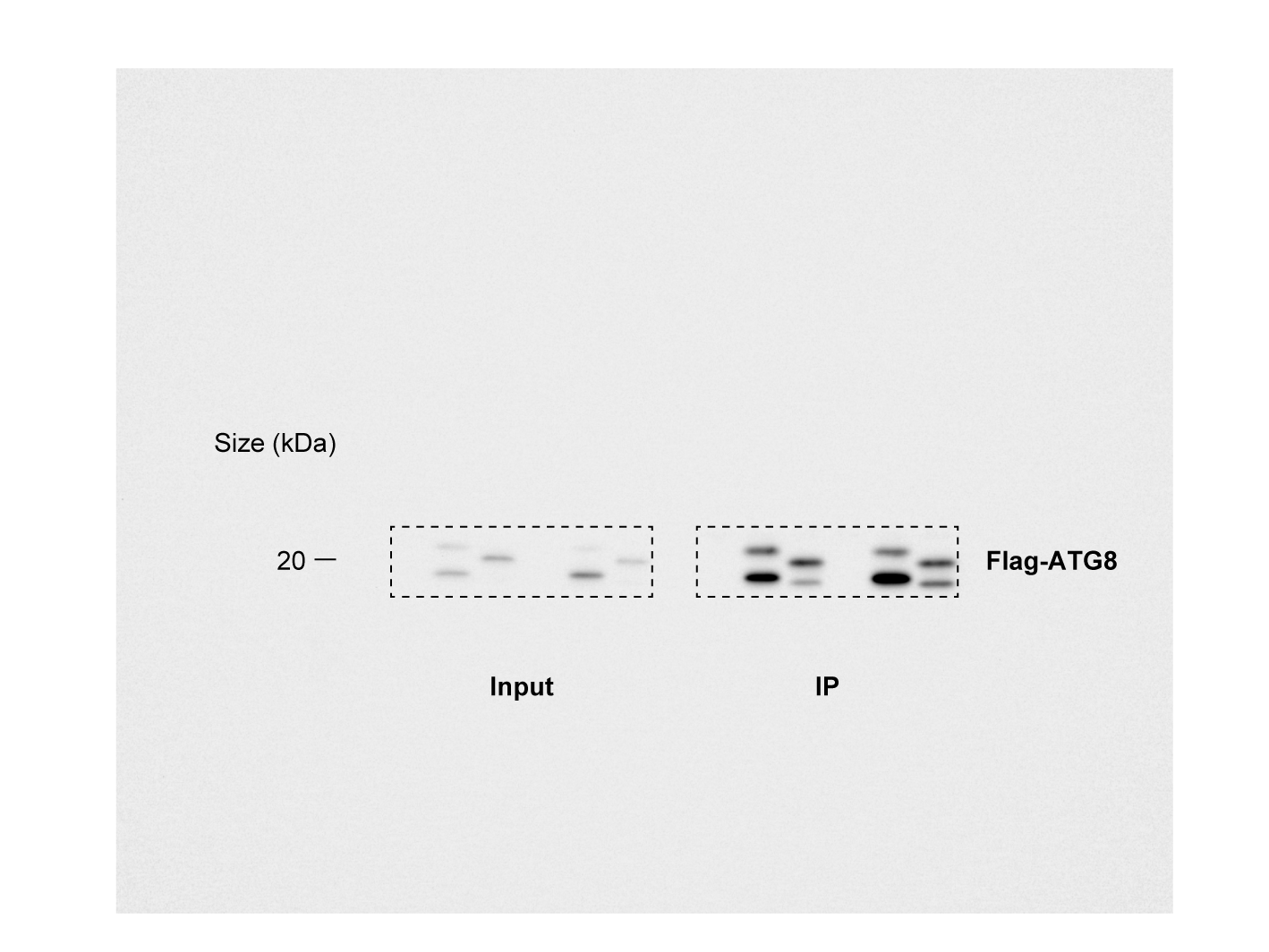

Supplement: Supplementary file 4 — Source Data for Expanded View [file EMBR-24-e57300-s011.zip › Fig EV5/EV5E/western_Flag-ATG8.tif]

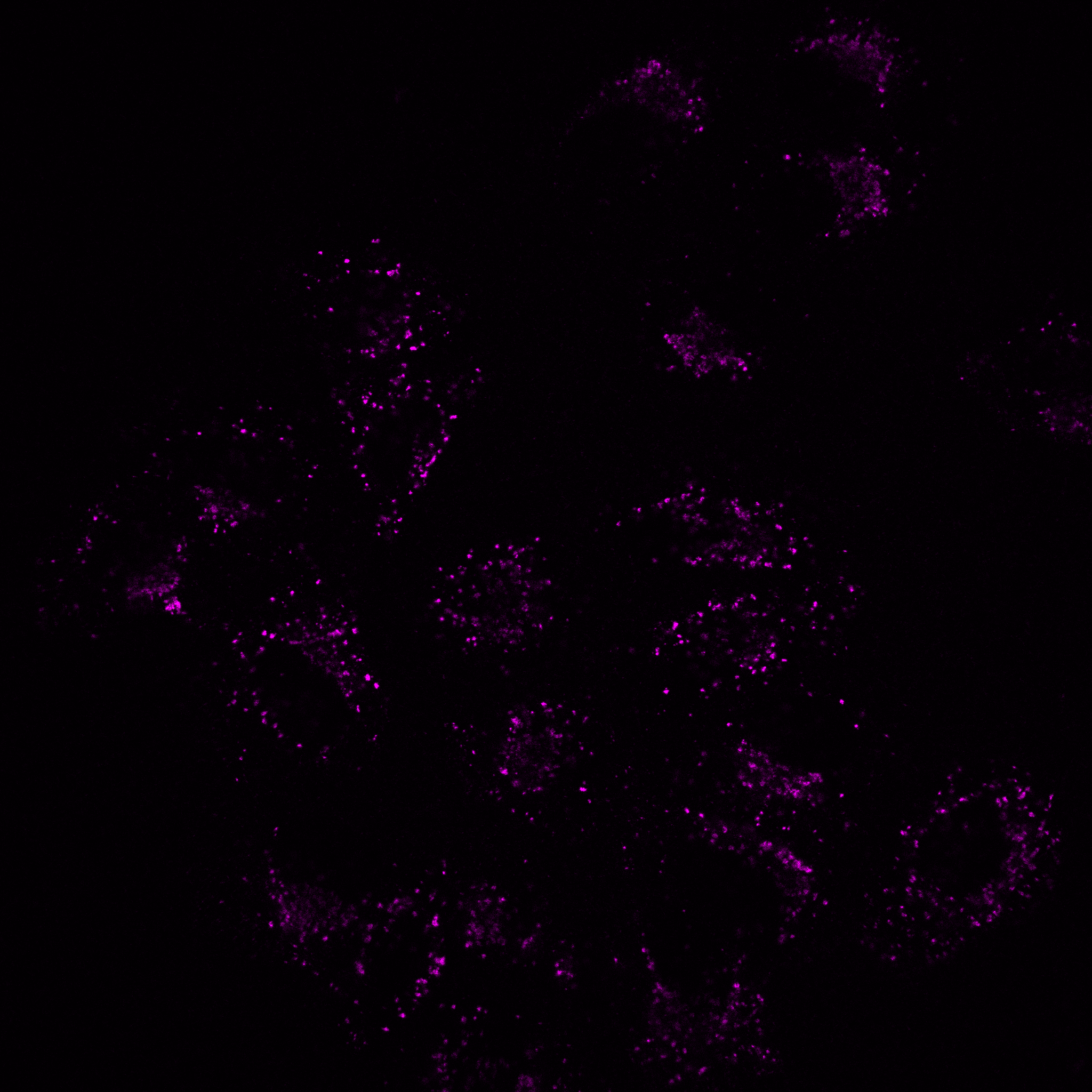

Supplement: Supplementary file 5 — Source Data for Figure 1 [file EMBR-24-e57300-s009.zip › Fig 1/1A/non-treated_LAMP1.tif]

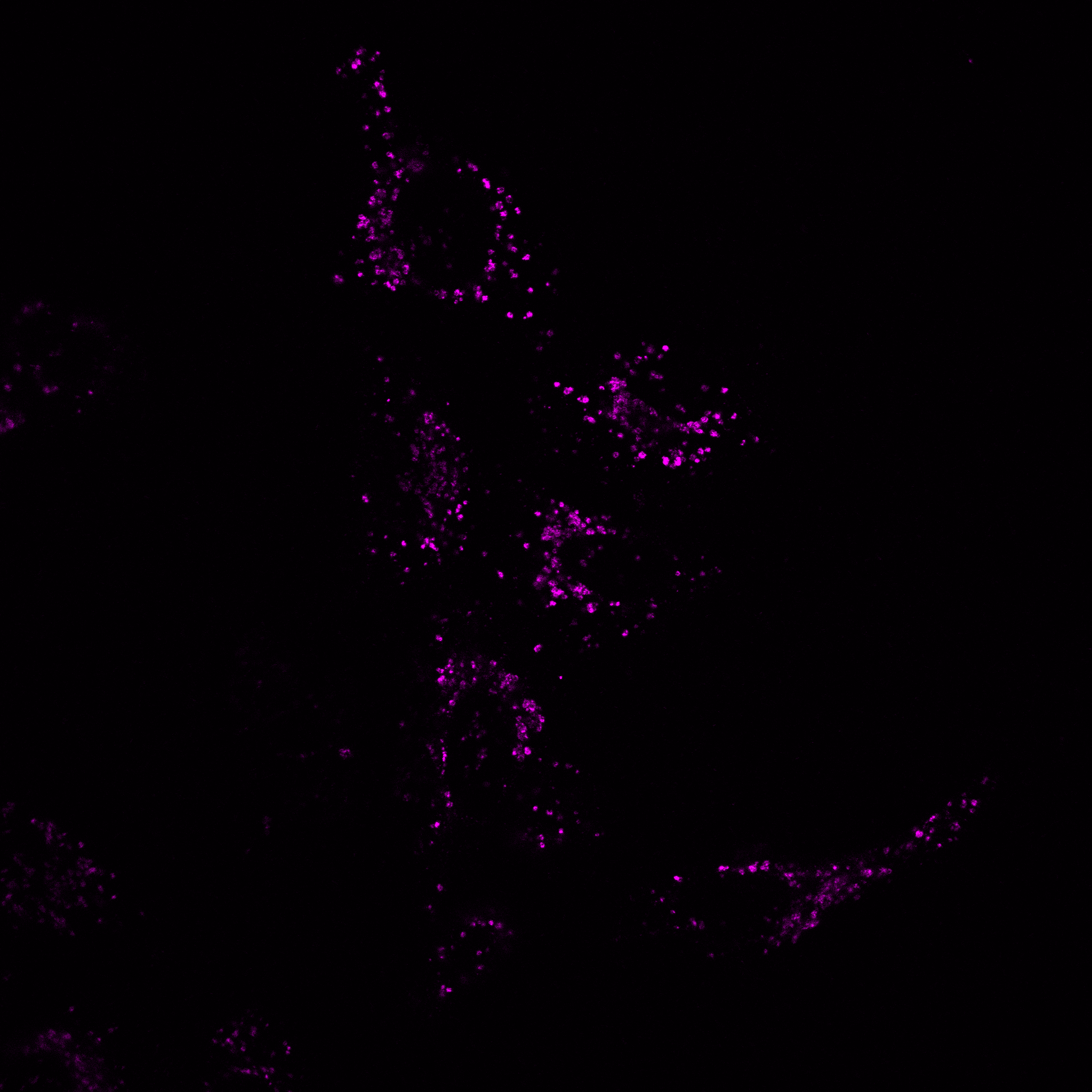

Supplement: Supplementary file 5 — Source Data for Figure 1 [file EMBR-24-e57300-s009.zip › Fig 1/1A/LLOMe_LAMP1.tif]

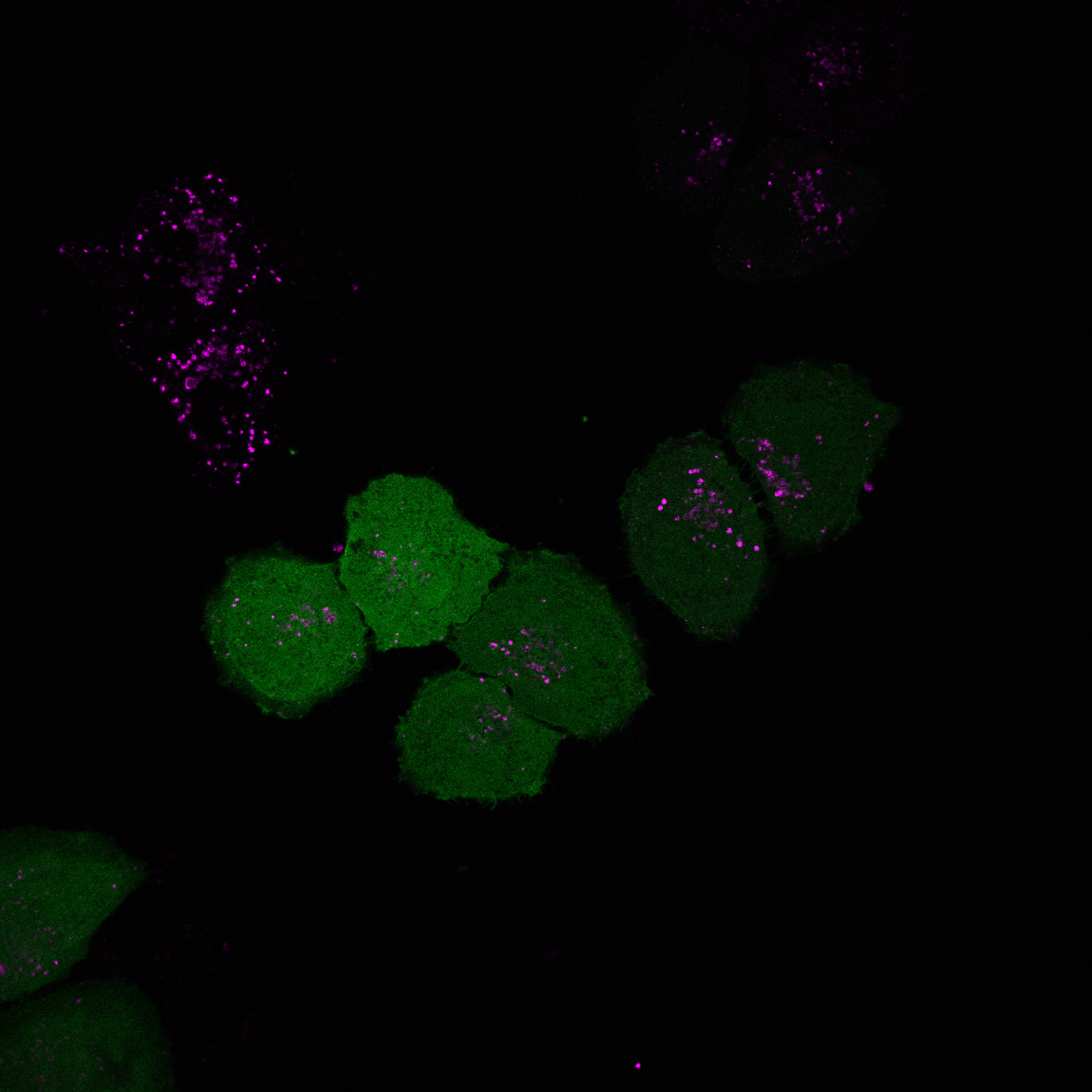

Supplement: Supplementary file 5 — Source Data for Figure 1 [file EMBR-24-e57300-s009.zip › Fig 1/1A/EBSS_Merge.tif]

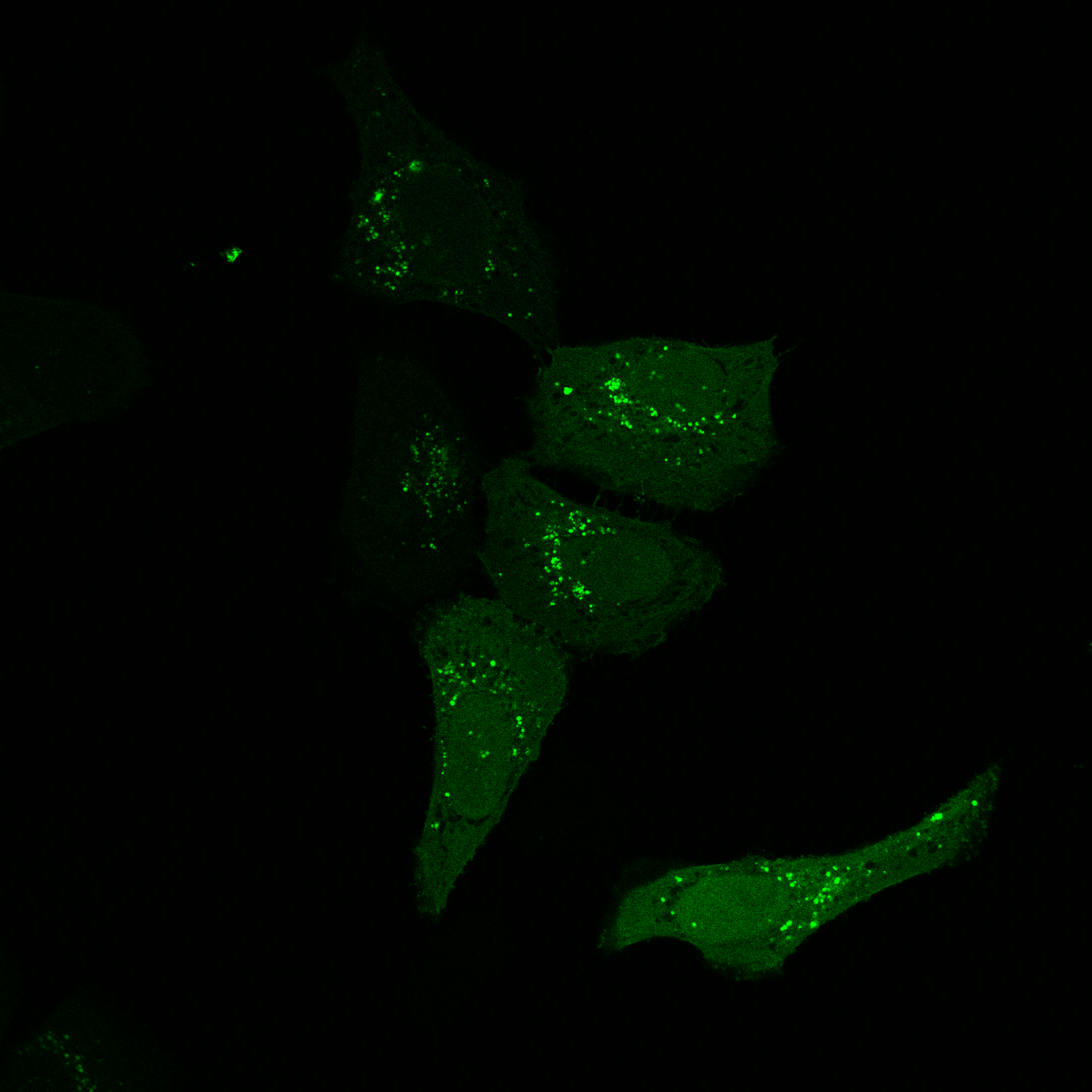

Supplement: Supplementary file 5 — Source Data for Figure 1 [file EMBR-24-e57300-s009.zip › Fig 1/1A/LLOMe_mNG-STK38.tif]

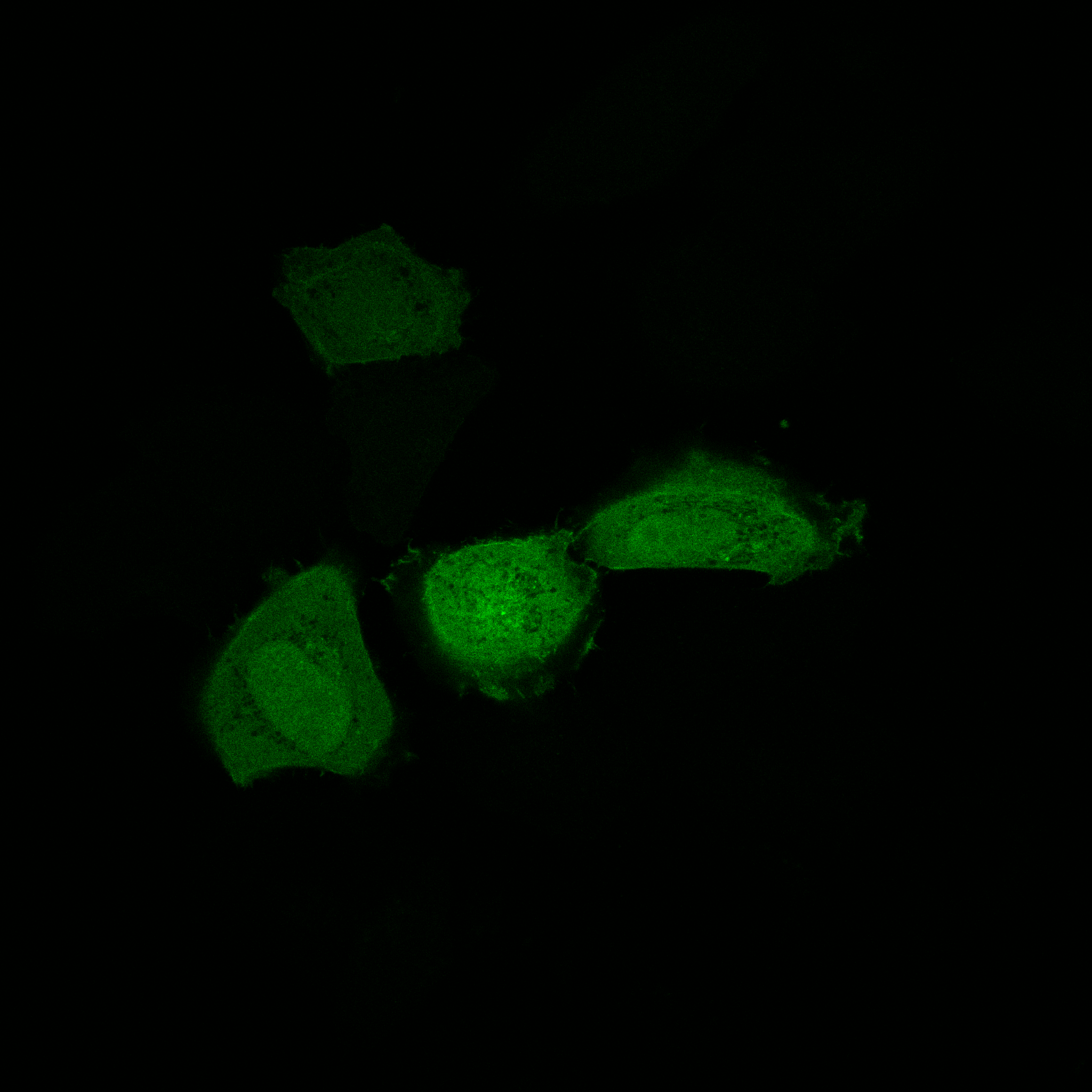

Supplement: Supplementary file 5 — Source Data for Figure 1 [file EMBR-24-e57300-s009.zip › Fig 1/1A/non-treated_mNG-STK38.tif]

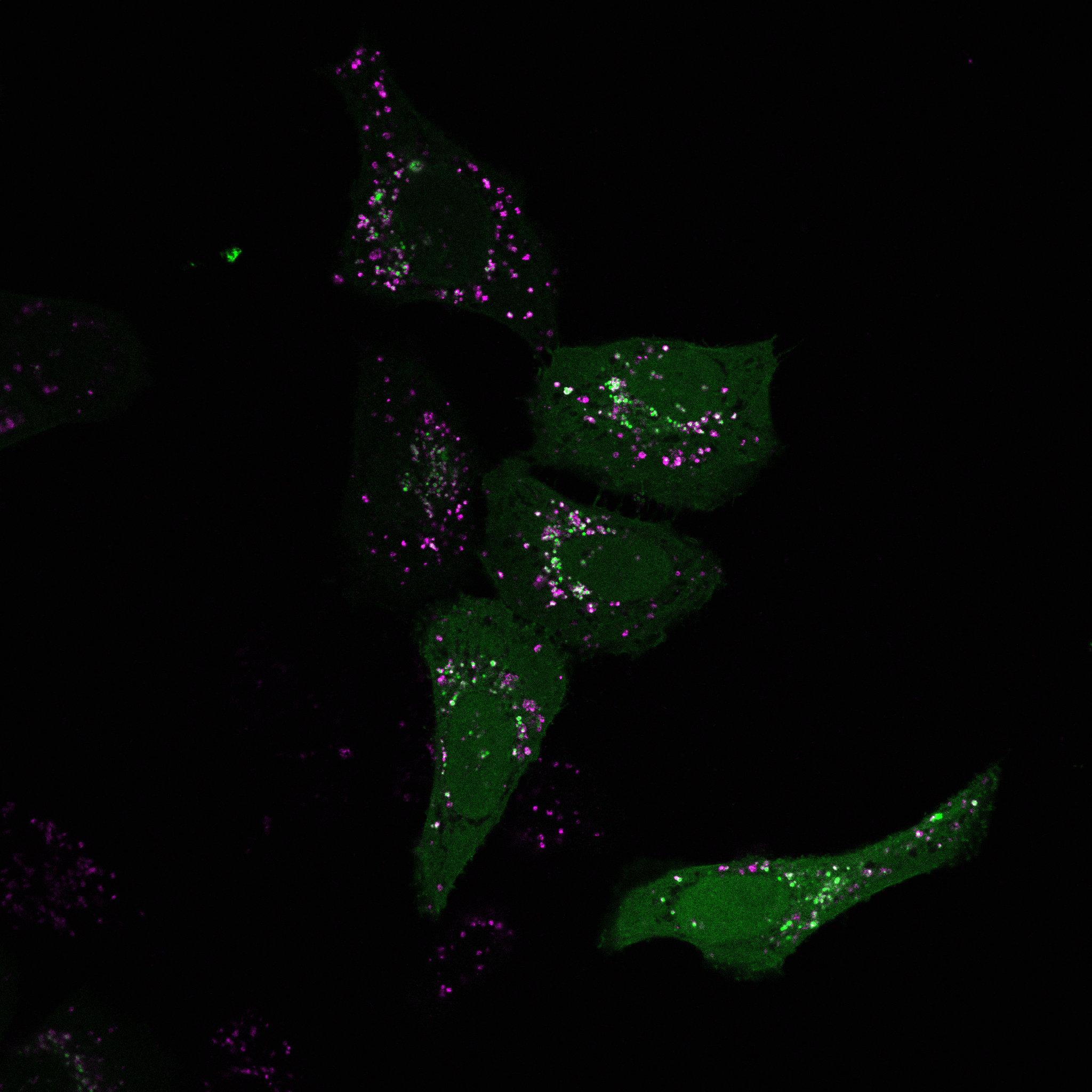

Supplement: Supplementary file 5 — Source Data for Figure 1 [file EMBR-24-e57300-s009.zip › Fig 1/1A/LLOMe_Merge.tif]

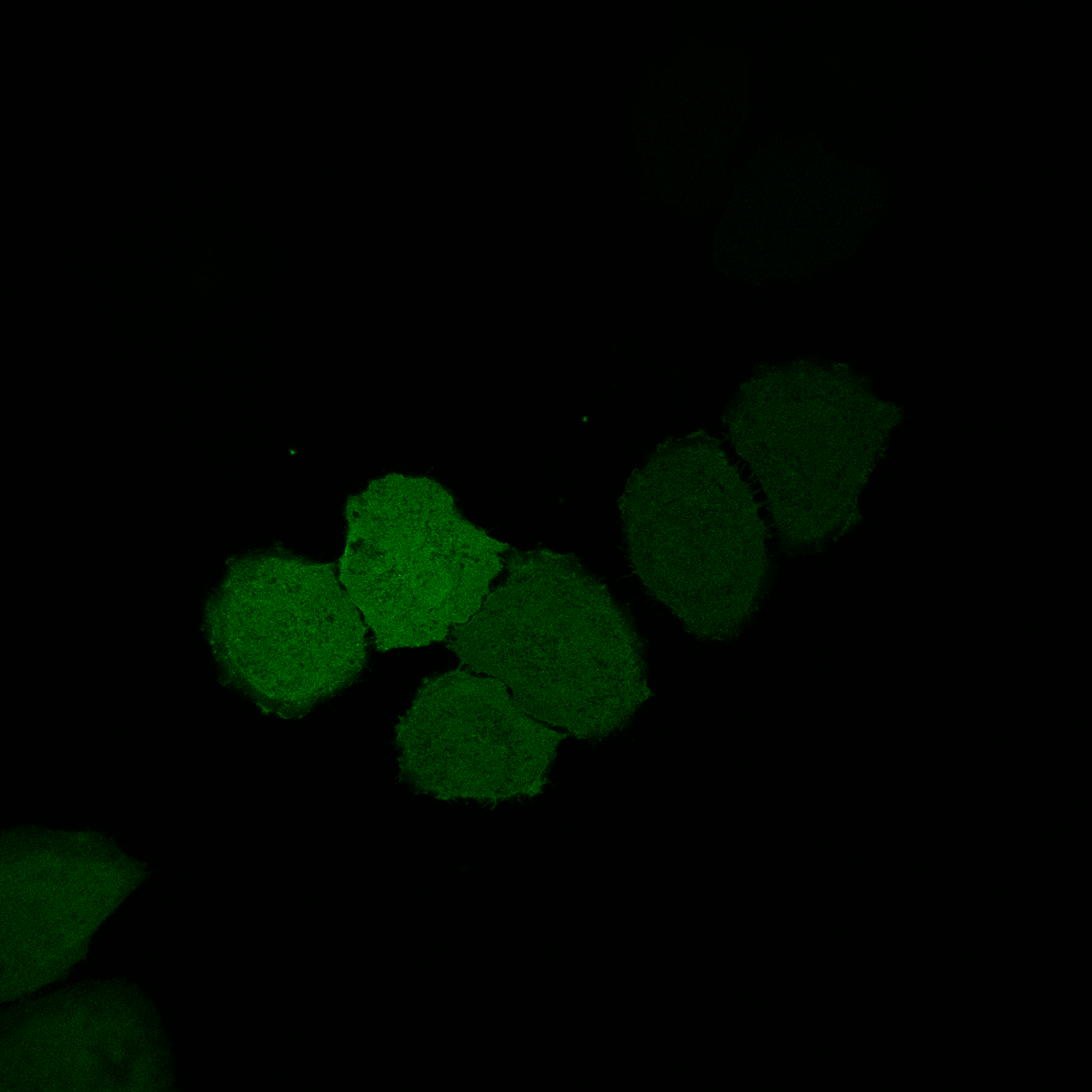

Supplement: Supplementary file 5 — Source Data for Figure 1 [file EMBR-24-e57300-s009.zip › Fig 1/1A/EBSS_mNG-STK38.tif]

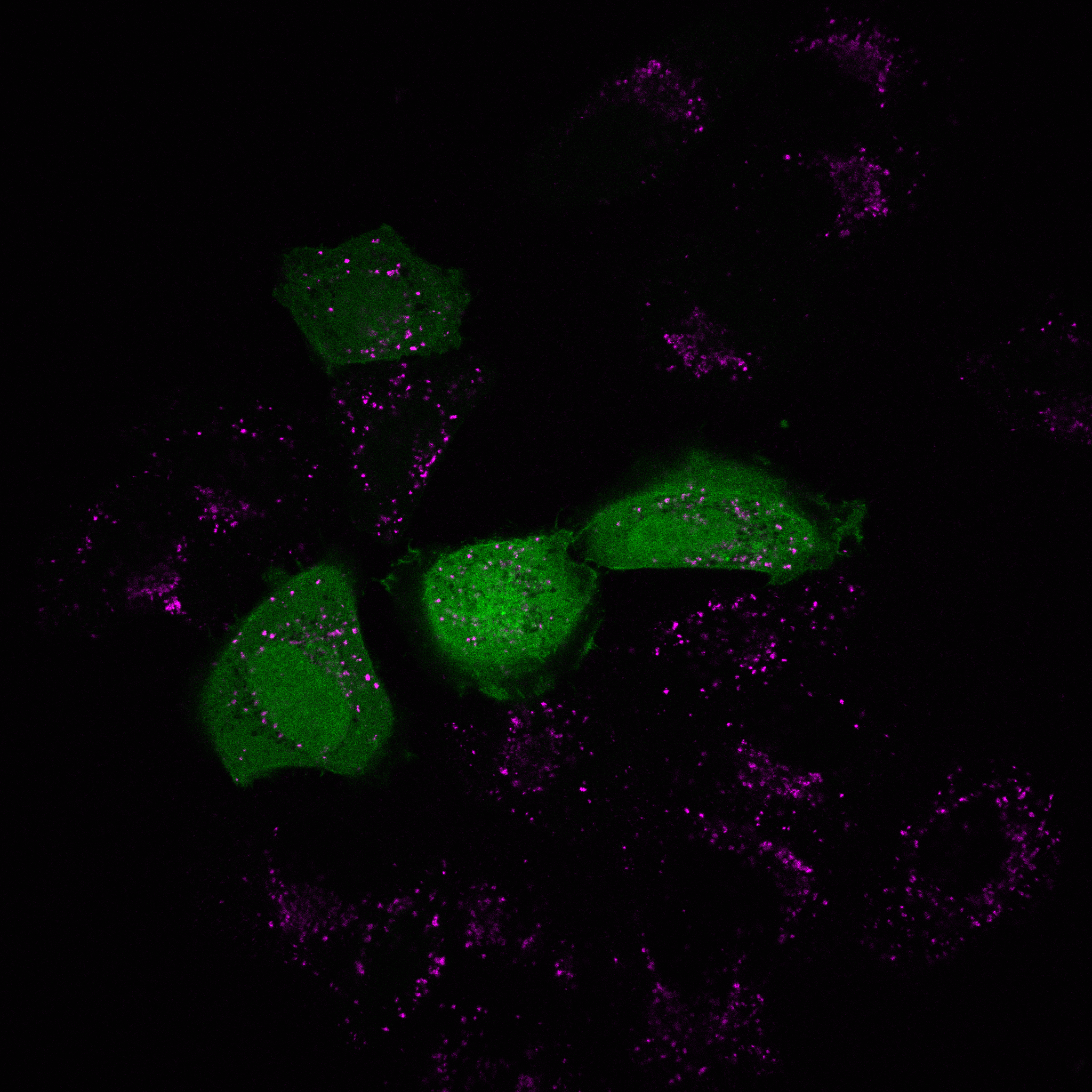

Supplement: Supplementary file 5 — Source Data for Figure 1 [file EMBR-24-e57300-s009.zip › Fig 1/1A/non-treated_Merge.tif]

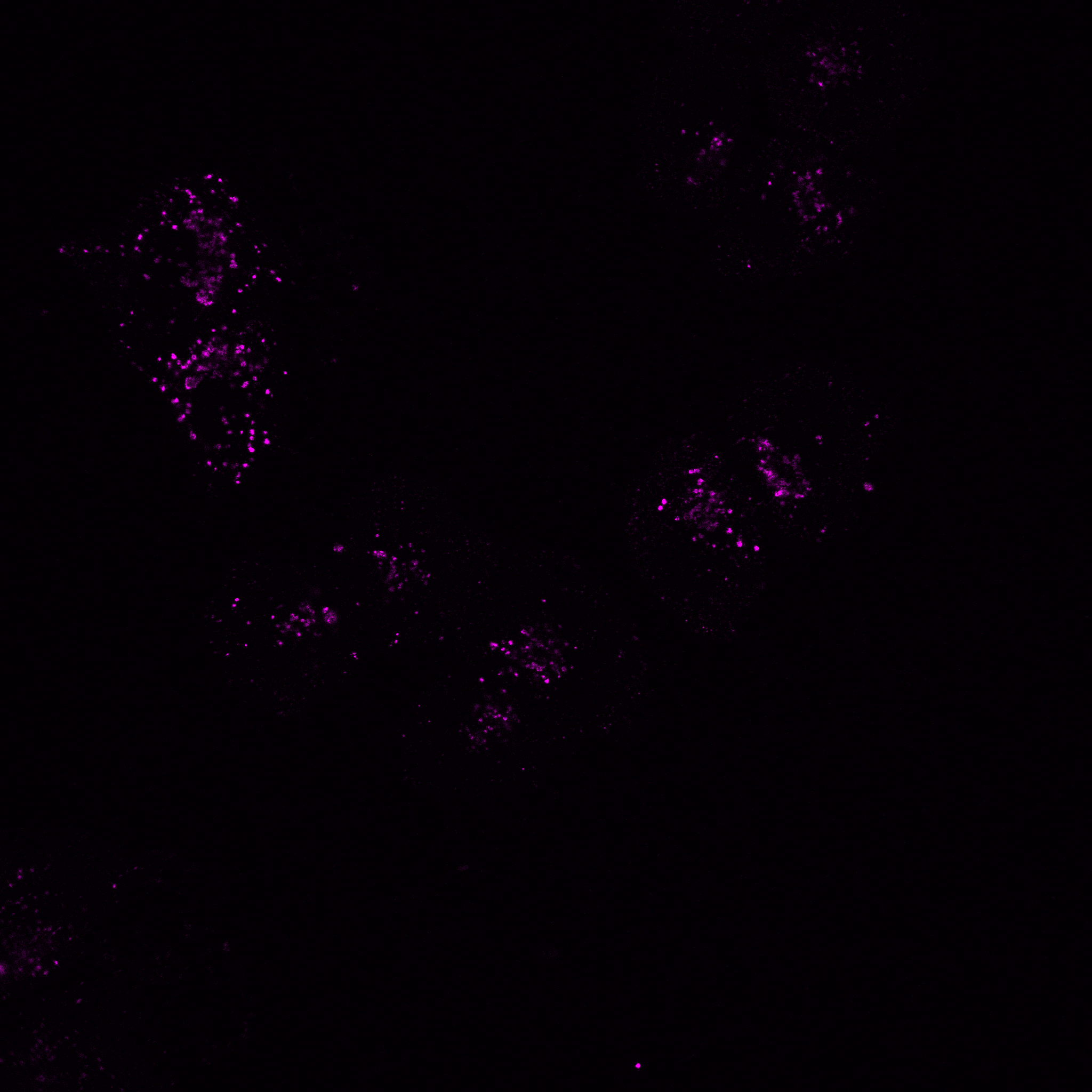

Supplement: Supplementary file 5 — Source Data for Figure 1 [file EMBR-24-e57300-s009.zip › Fig 1/1A/EBSS_LAMP1.tif]

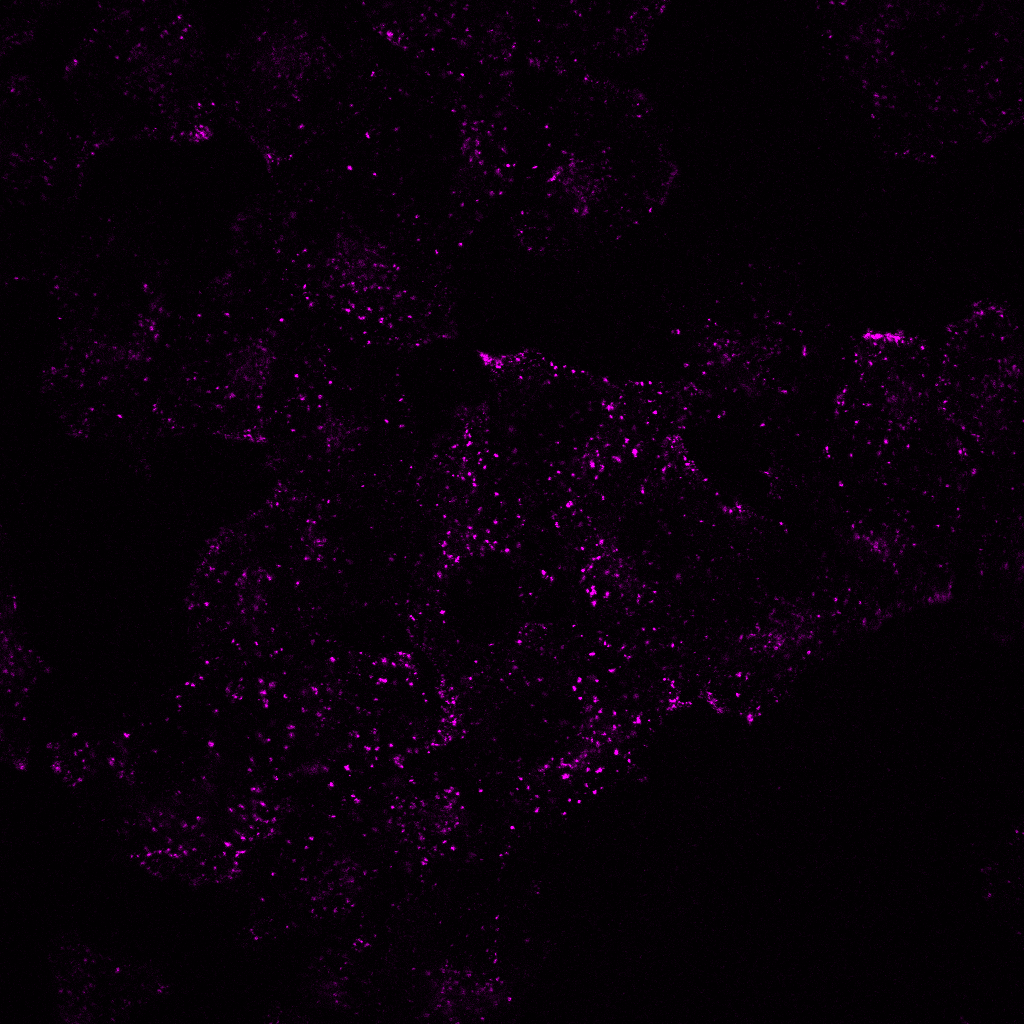

Supplement: Supplementary file 5 — Source Data for Figure 1 [file EMBR-24-e57300-s009.zip › Fig 1/1B/non-treated_LAMP1.tif]

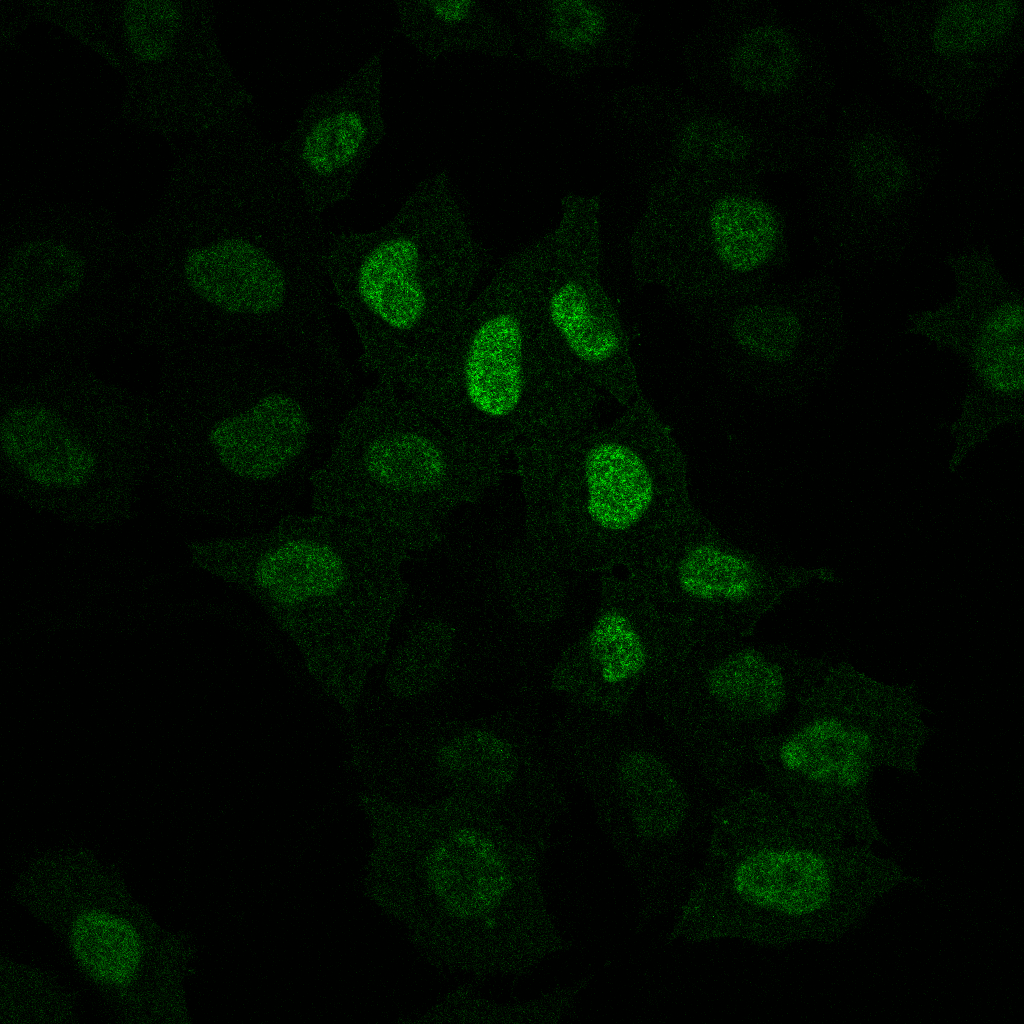

Supplement: Supplementary file 5 — Source Data for Figure 1 [file EMBR-24-e57300-s009.zip › Fig 1/1B/BAPTA_mNG-STK38.tif]

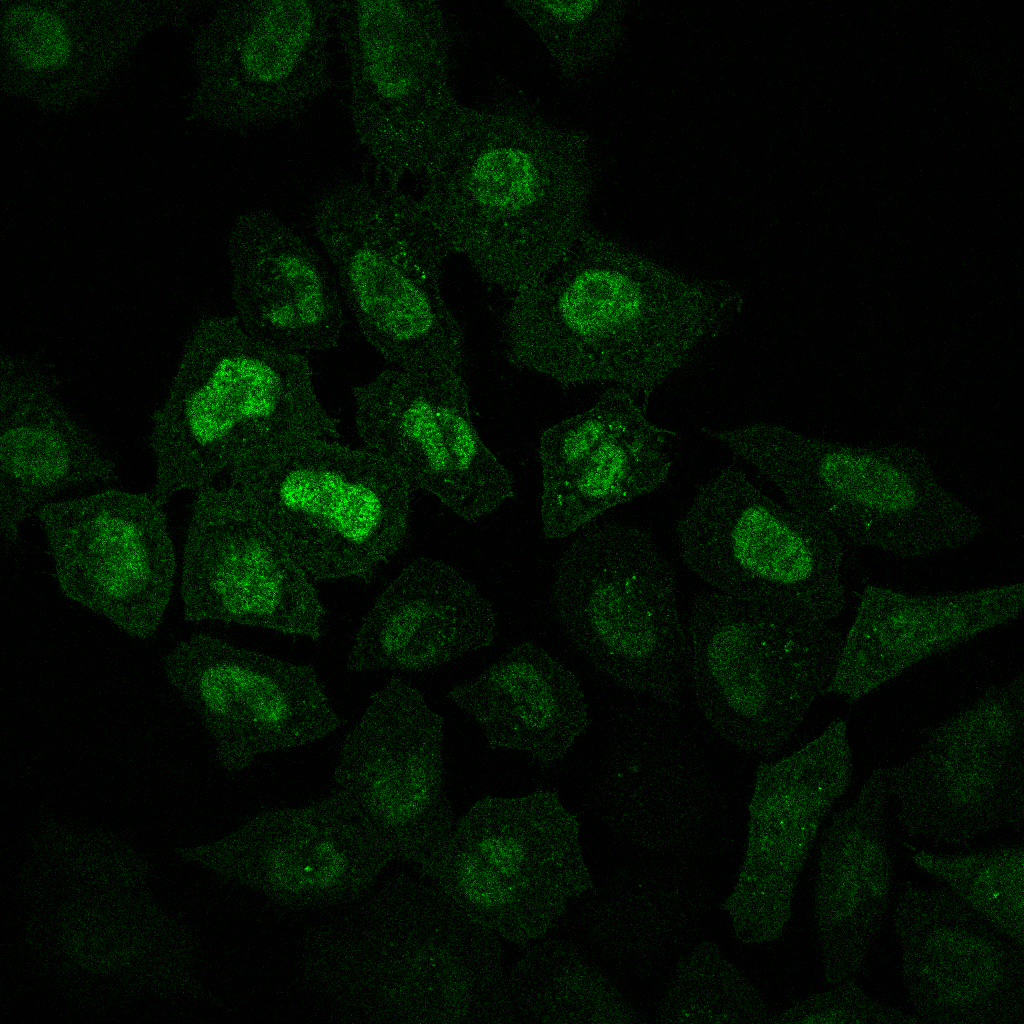

Supplement: Supplementary file 5 — Source Data for Figure 1 [file EMBR-24-e57300-s009.zip › Fig 1/1B/LLOMe+BAPTA_mNG-STK38.tif]

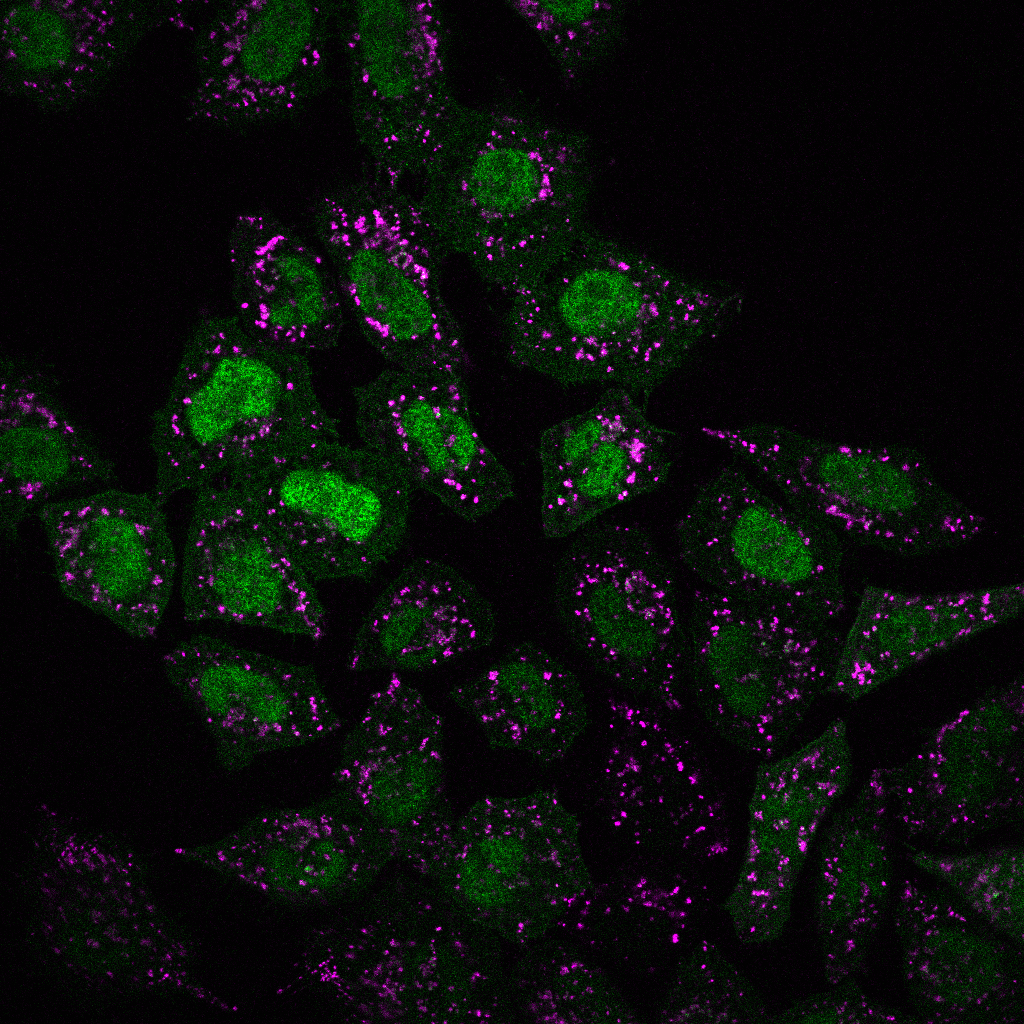

Supplement: Supplementary file 5 — Source Data for Figure 1 [file EMBR-24-e57300-s009.zip › Fig 1/1B/LLOMe+BAPTA_Merge.tif]

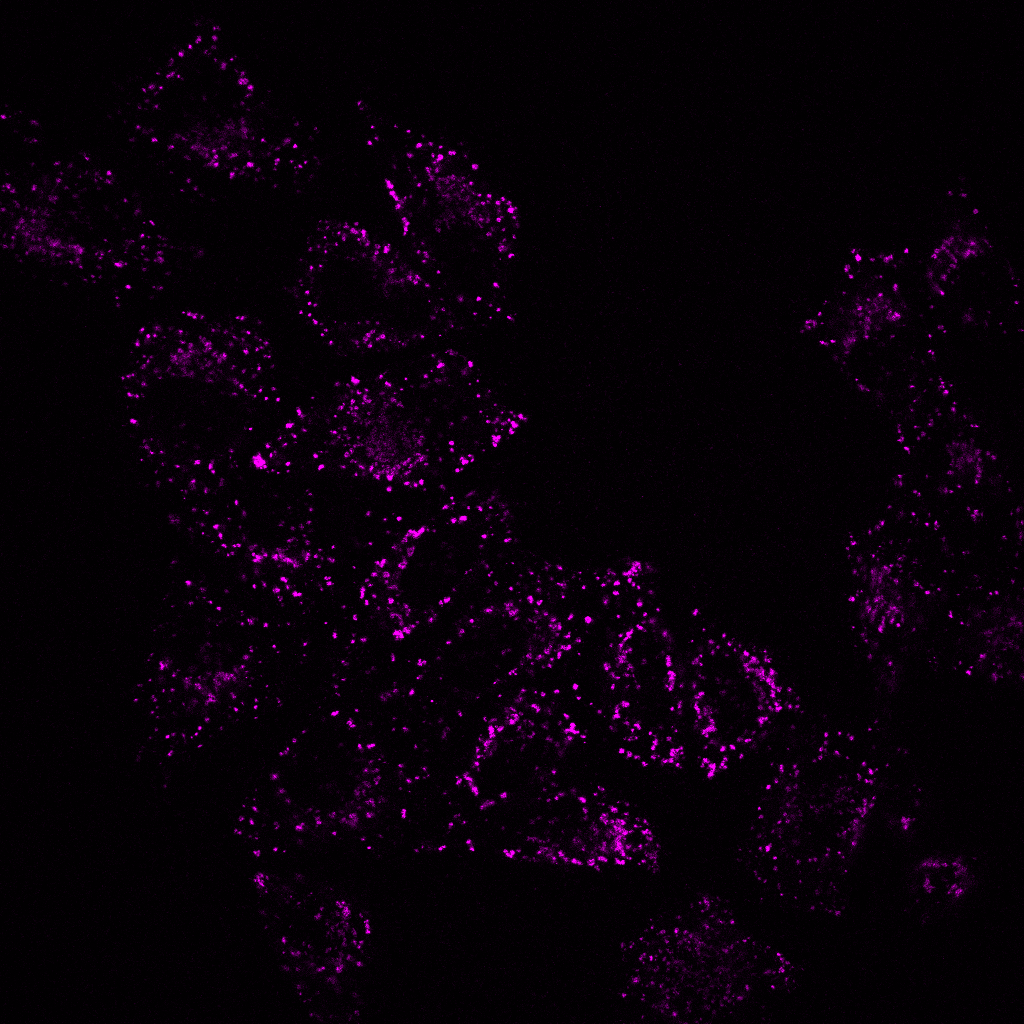

Supplement: Supplementary file 5 — Source Data for Figure 1 [file EMBR-24-e57300-s009.zip › Fig 1/1B/LLOMe_LAMP1.tif]

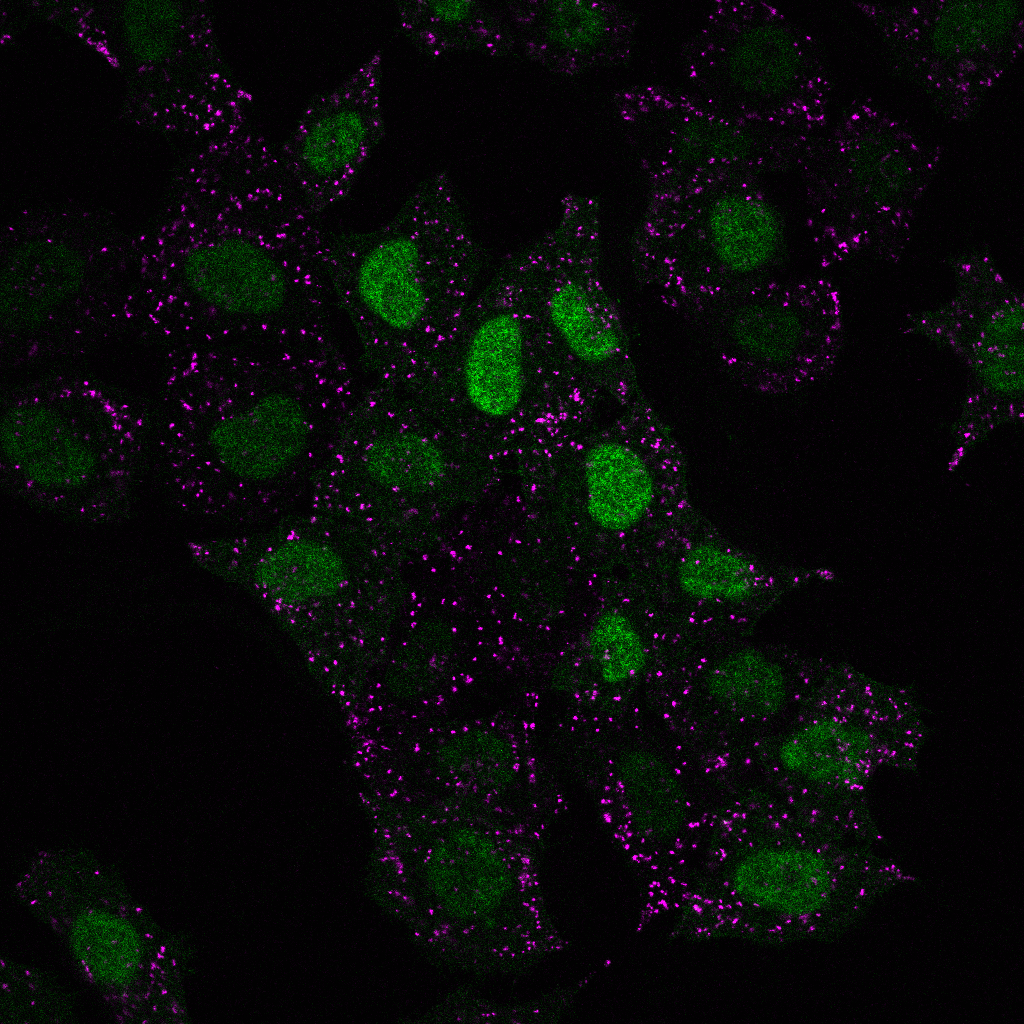

Supplement: Supplementary file 5 — Source Data for Figure 1 [file EMBR-24-e57300-s009.zip › Fig 1/1B/BAPTA_Merge.tif]

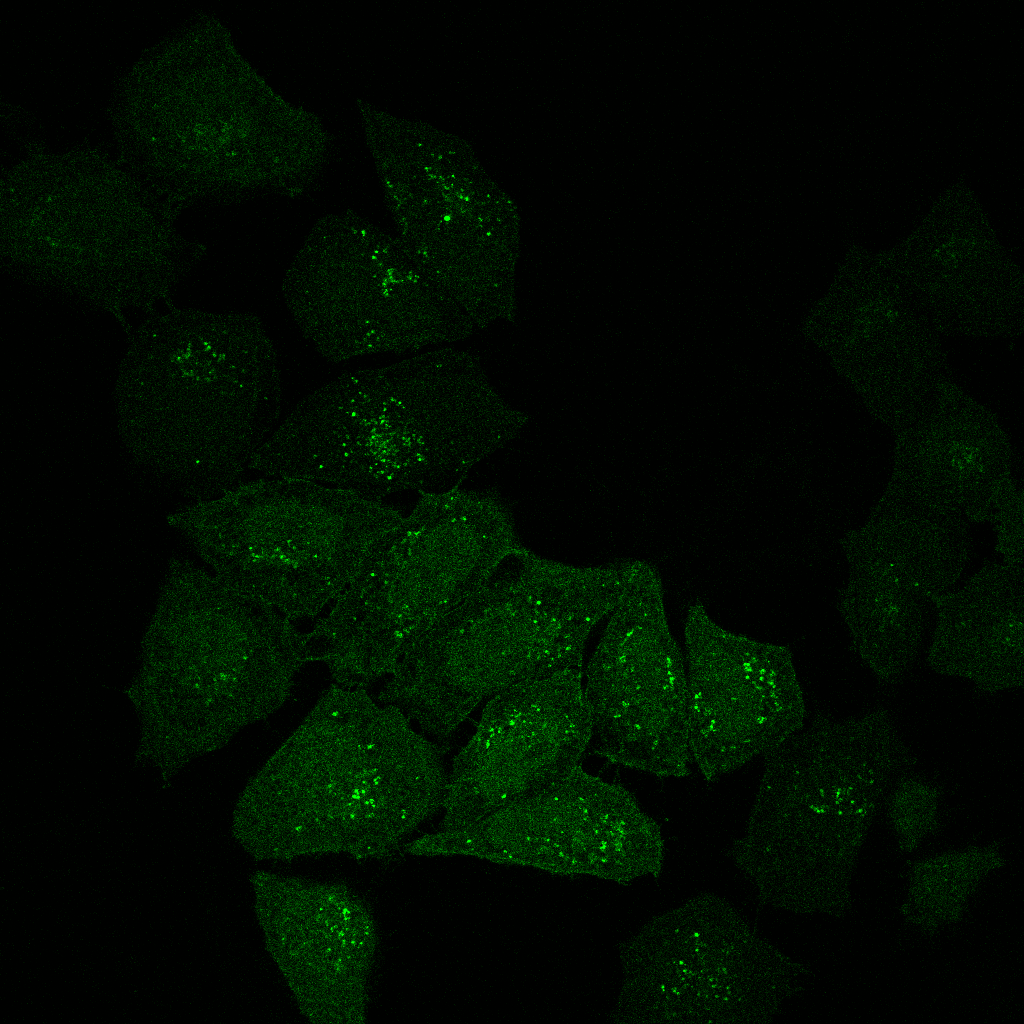

Supplement: Supplementary file 5 — Source Data for Figure 1 [file EMBR-24-e57300-s009.zip › Fig 1/1B/LLOMe_mNG-STK38.tif]

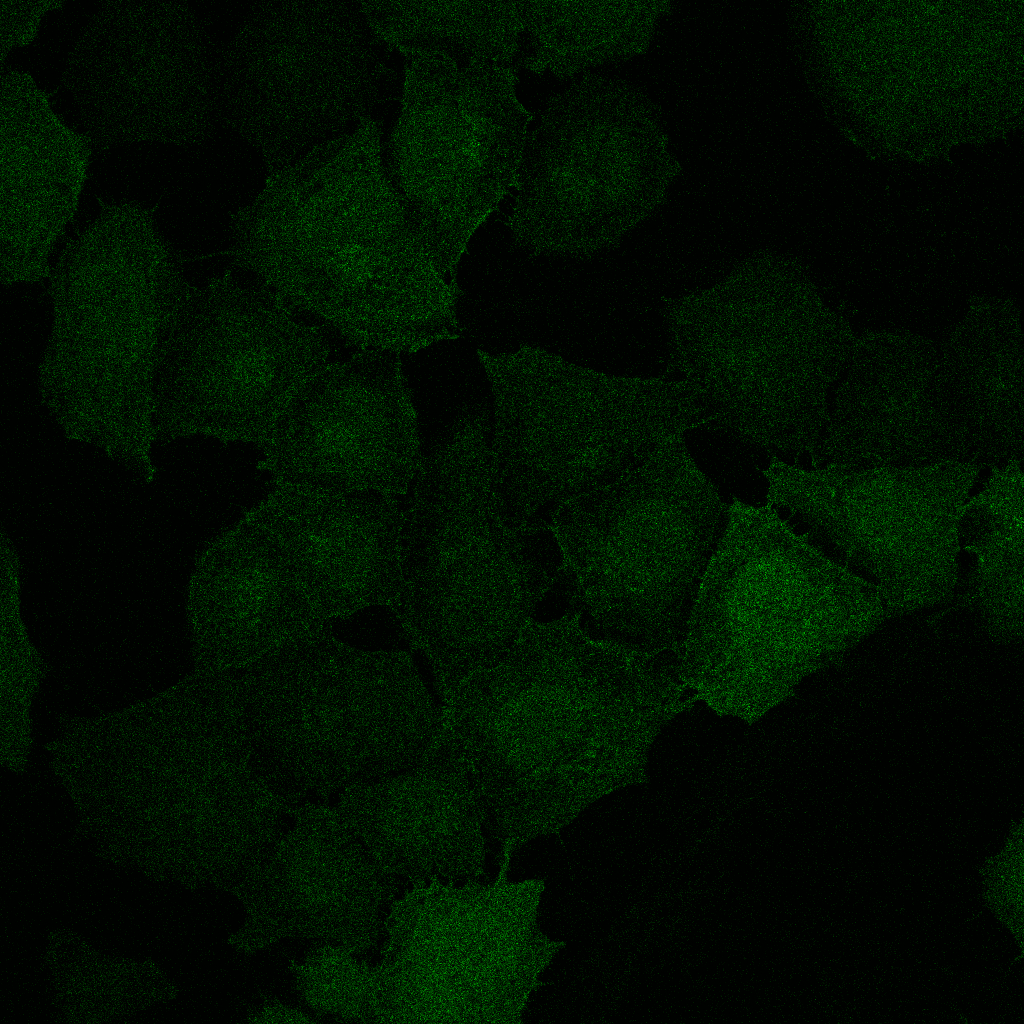

Supplement: Supplementary file 5 — Source Data for Figure 1 [file EMBR-24-e57300-s009.zip › Fig 1/1B/non-treated_mNG-STK38.tif]

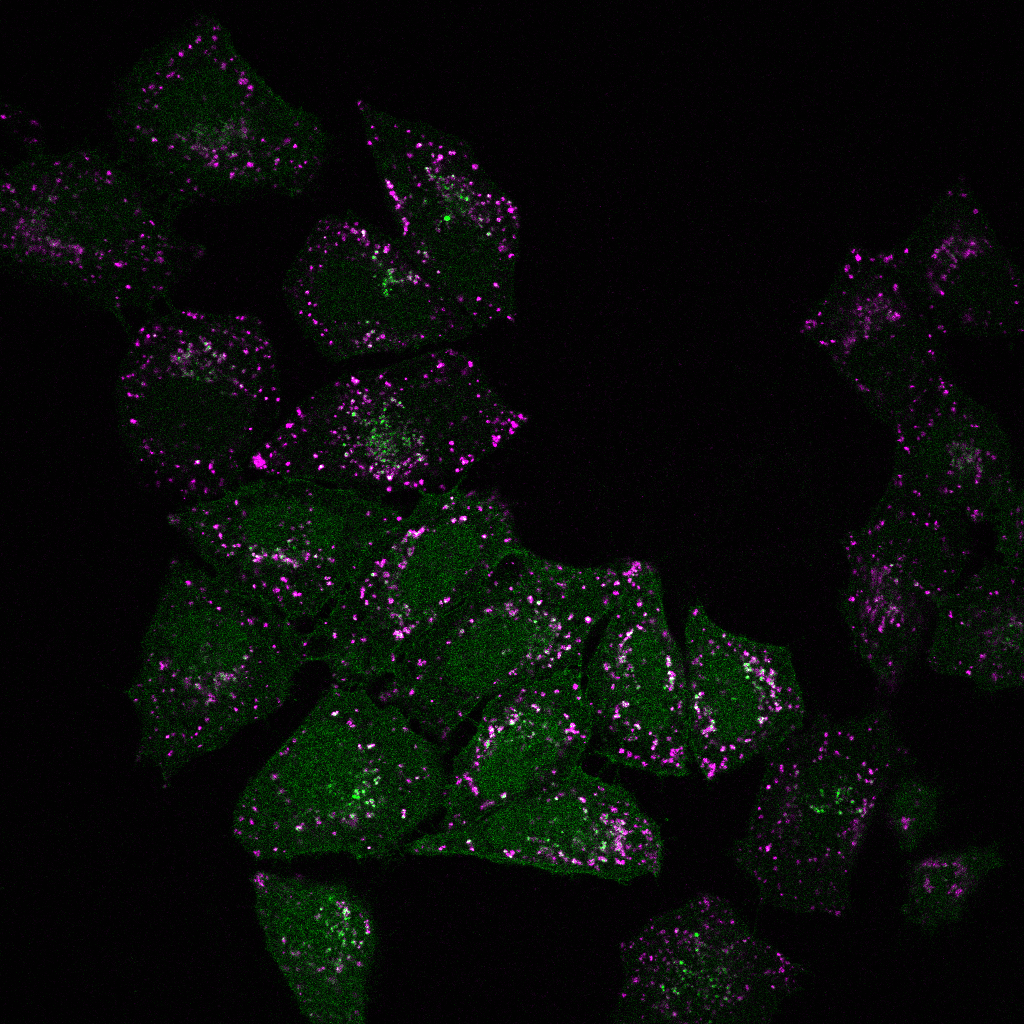

Supplement: Supplementary file 5 — Source Data for Figure 1 [file EMBR-24-e57300-s009.zip › Fig 1/1B/LLOMe_Merge.tif]

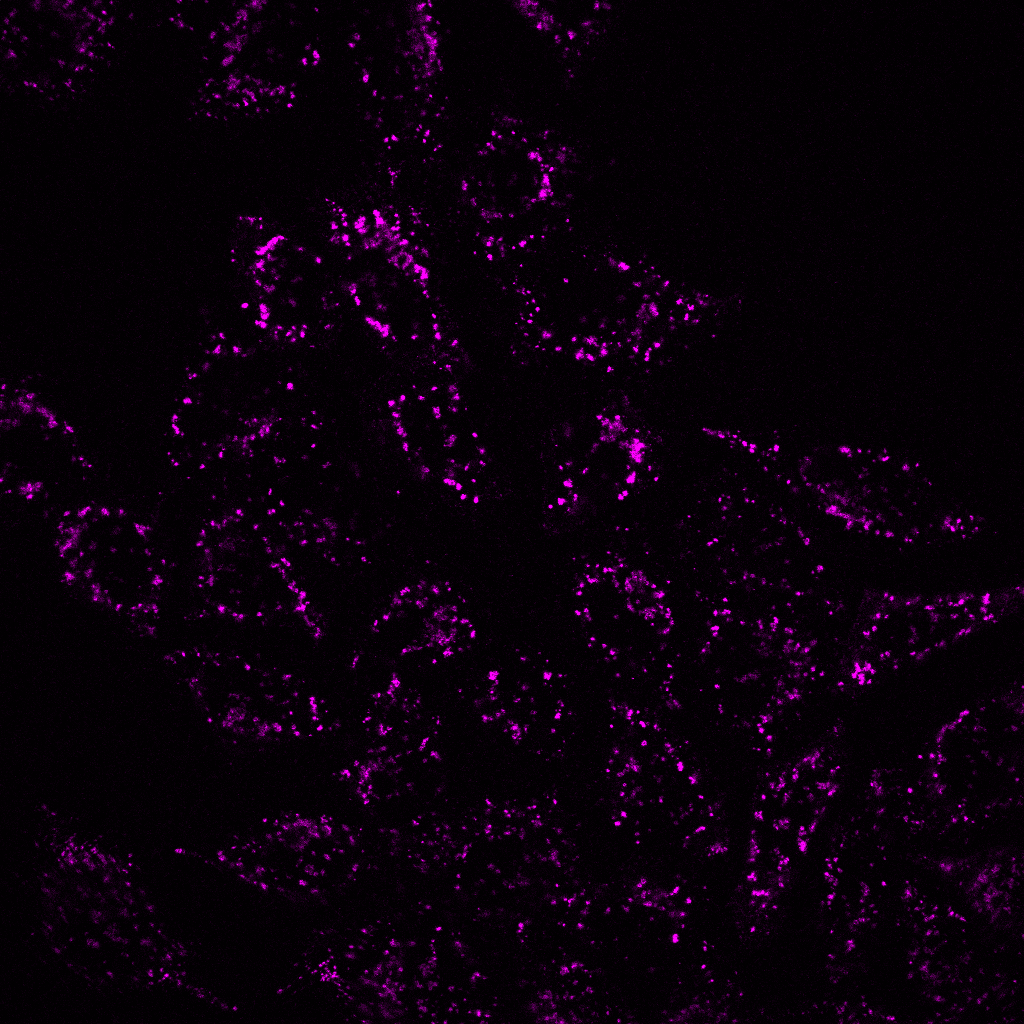

Supplement: Supplementary file 5 — Source Data for Figure 1 [file EMBR-24-e57300-s009.zip › Fig 1/1B/LLOMe+BAPTA_LAMP1.tif]

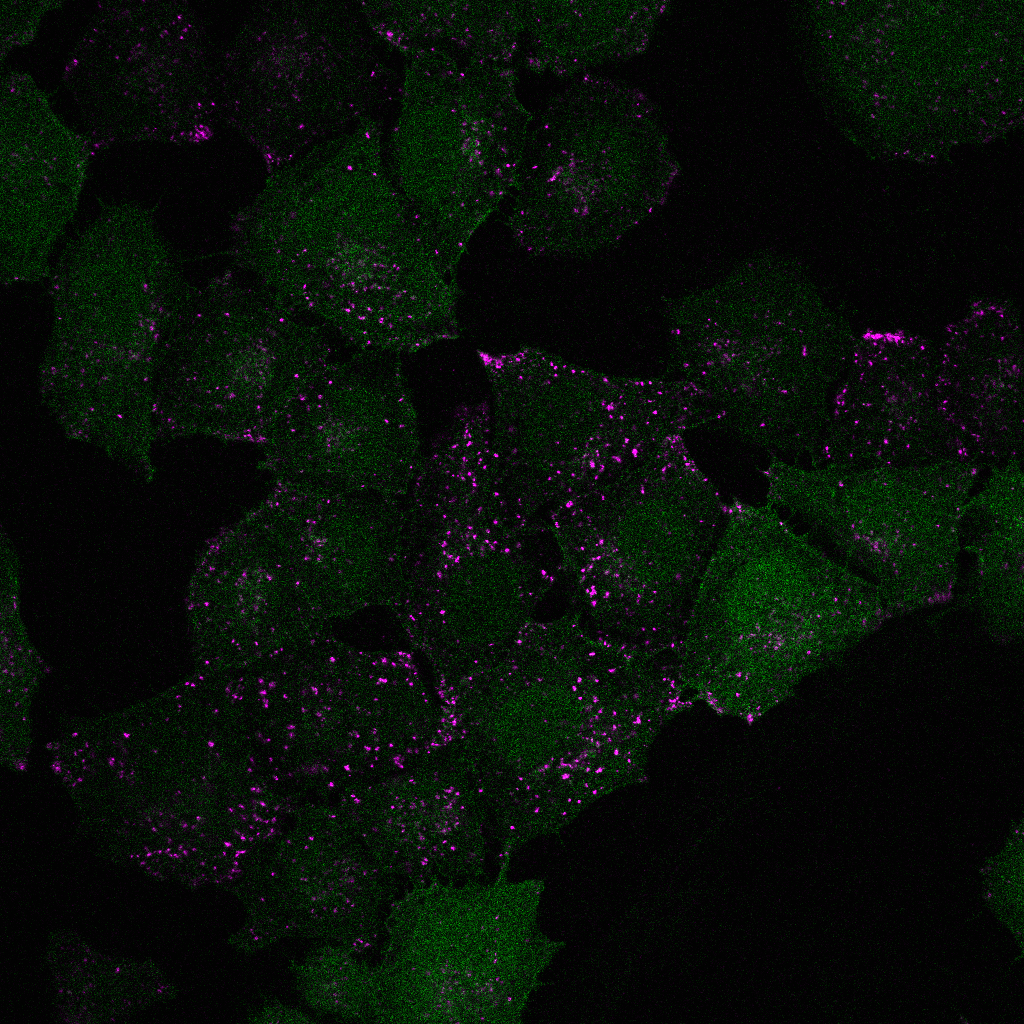

Supplement: Supplementary file 5 — Source Data for Figure 1 [file EMBR-24-e57300-s009.zip › Fig 1/1B/non-treated_Merge.tif]

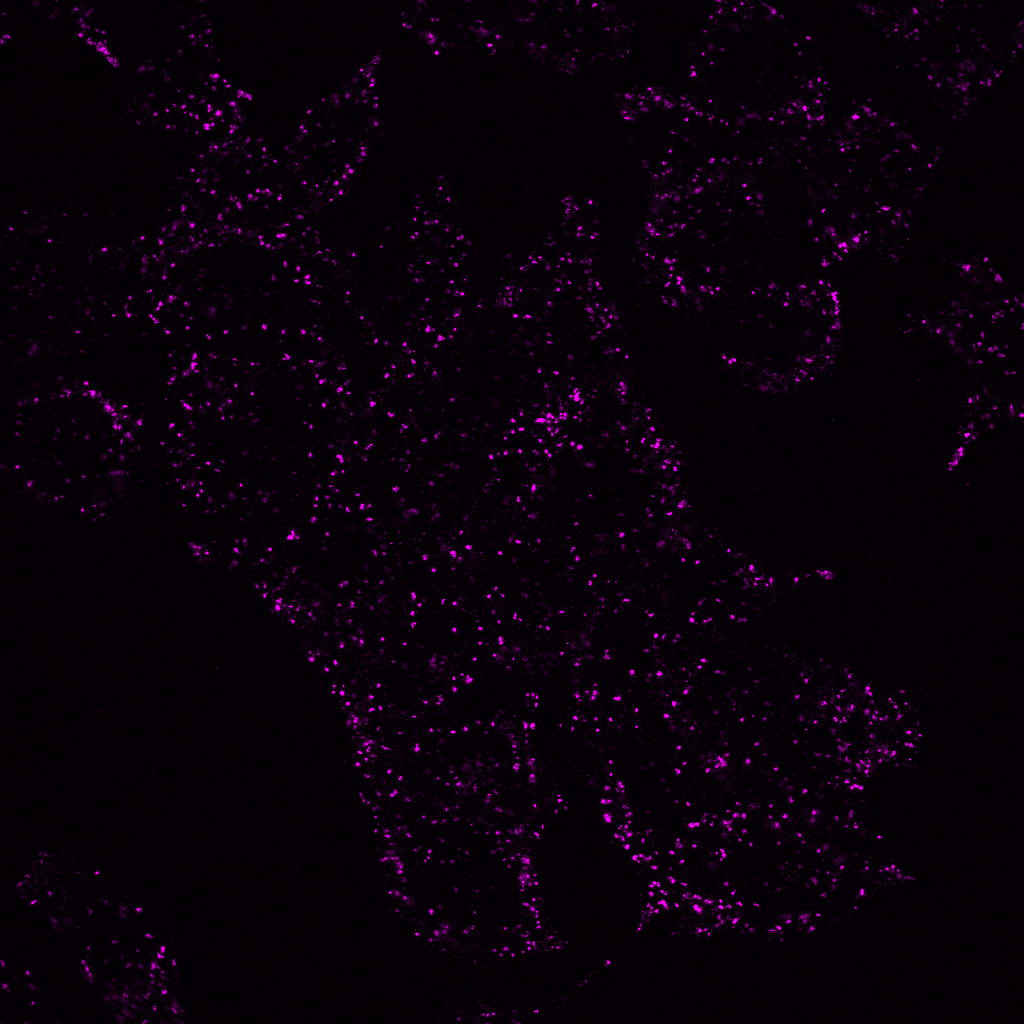

Supplement: Supplementary file 5 — Source Data for Figure 1 [file EMBR-24-e57300-s009.zip › Fig 1/1B/BAPTA_LAMP1.tif]

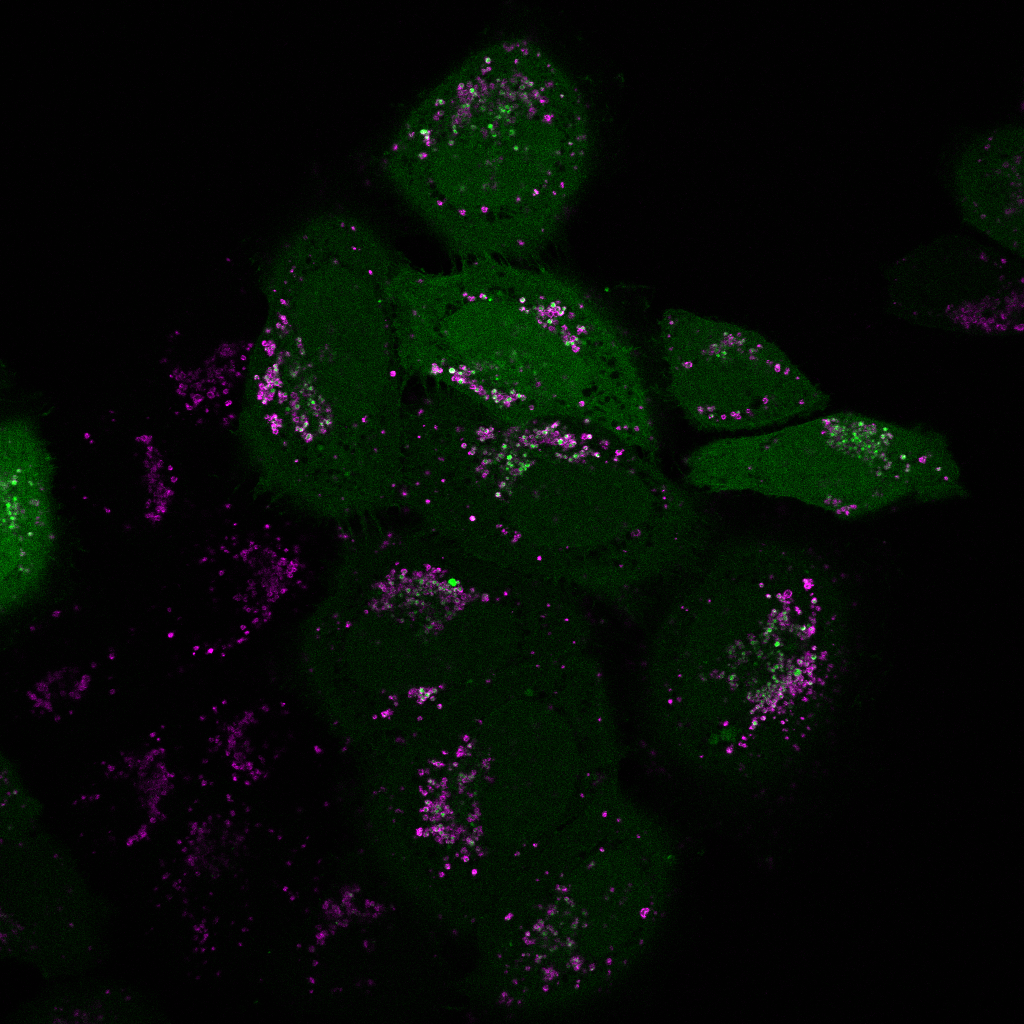

Supplement: Supplementary file 5 — Source Data for Figure 1 [file EMBR-24-e57300-s009.zip › Fig 1/1E/WT_LLOMe_Merge.tif]

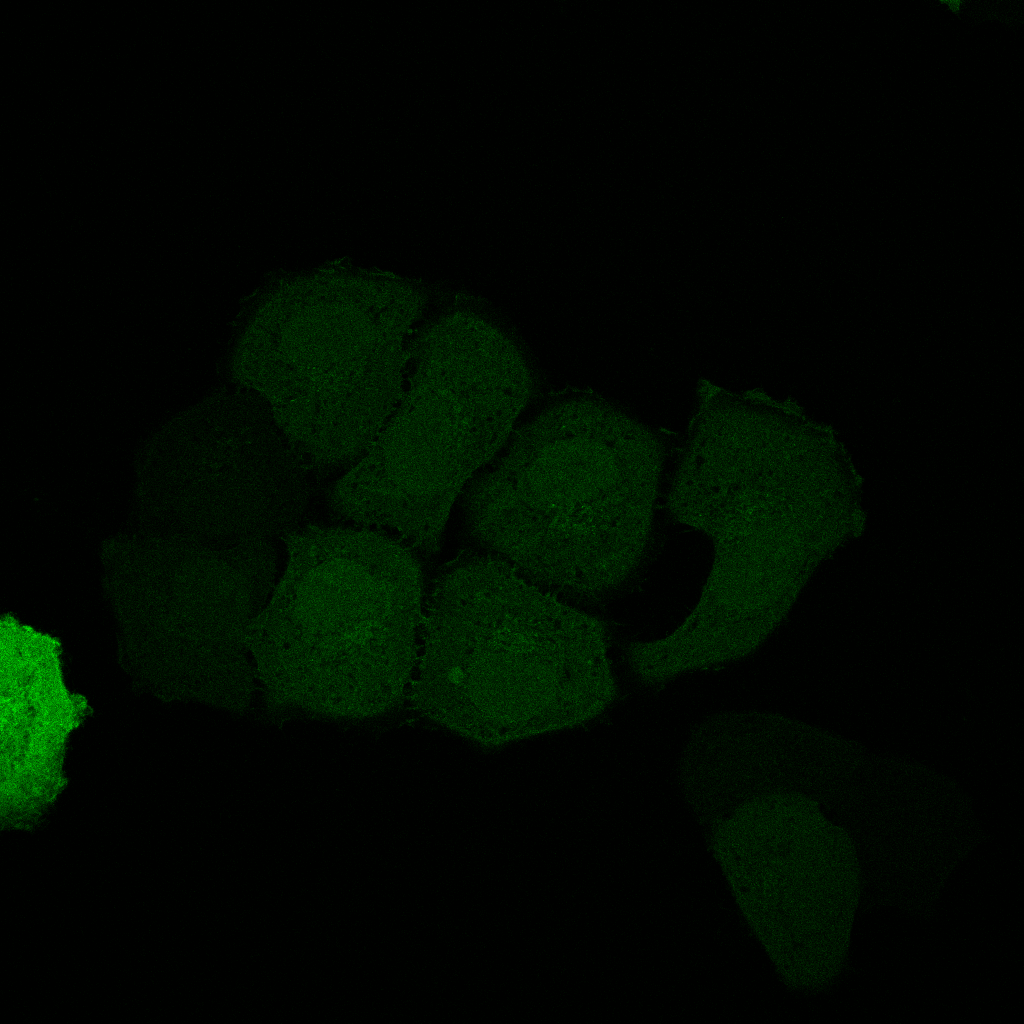

Supplement: Supplementary file 5 — Source Data for Figure 1 [file EMBR-24-e57300-s009.zip › Fig 1/1E/deltaC_non-treated_mNG-STK38.tif]

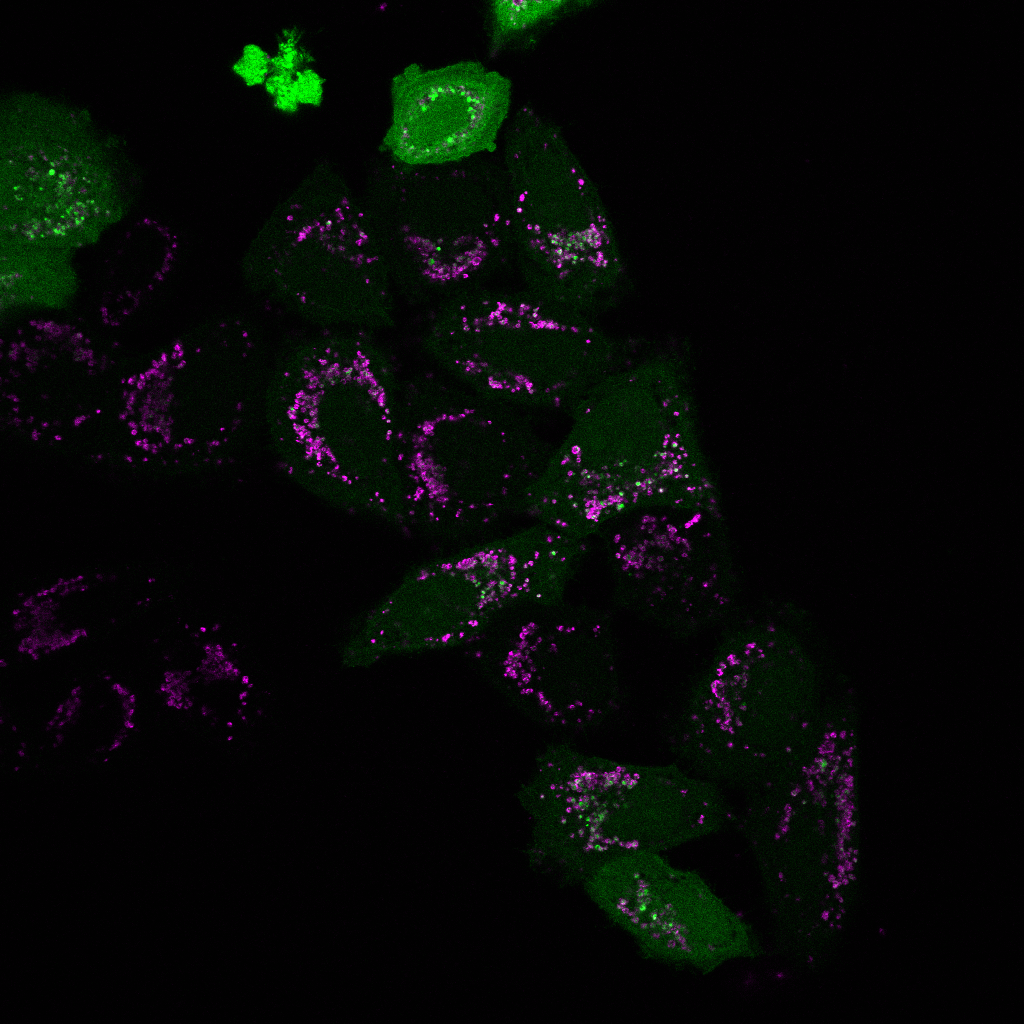

Supplement: Supplementary file 5 — Source Data for Figure 1 [file EMBR-24-e57300-s009.zip › Fig 1/1E/T444A_LLOMe_Merge.tif]

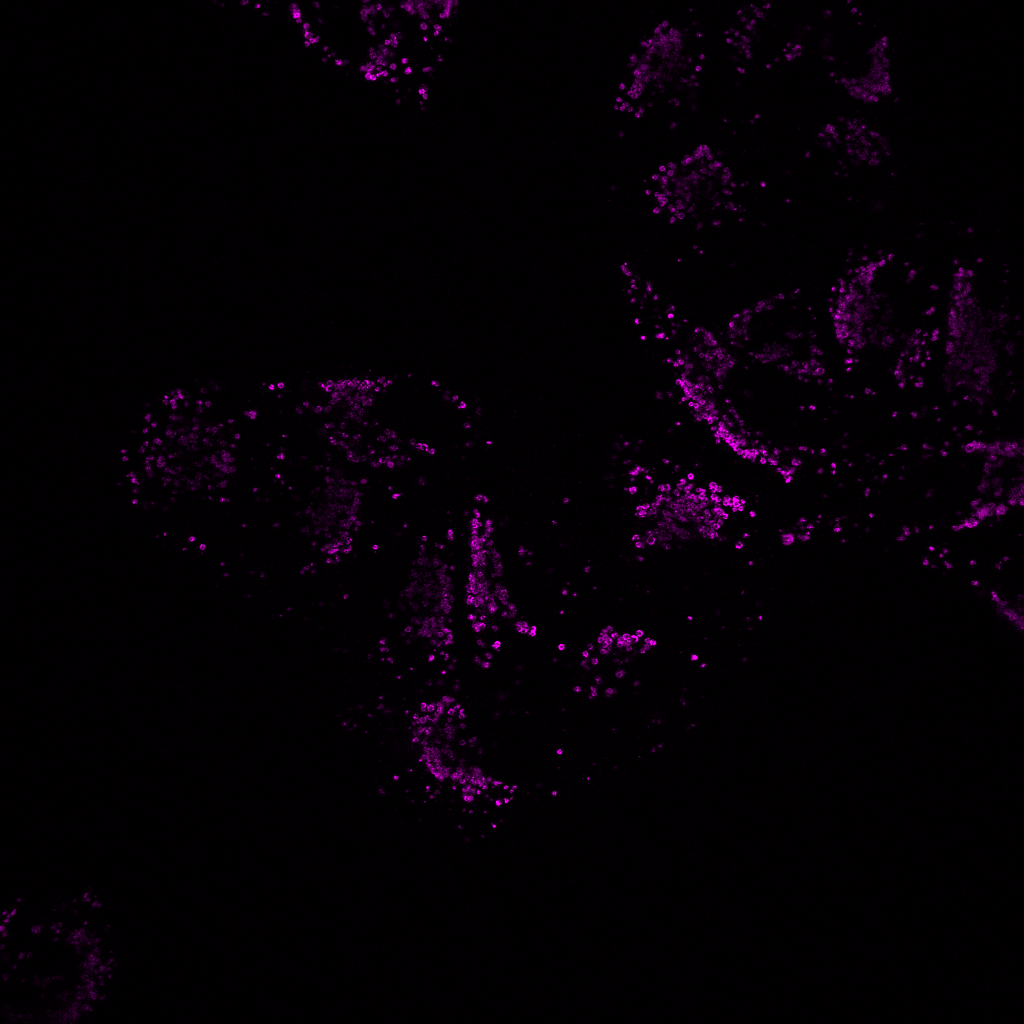

Supplement: Supplementary file 5 — Source Data for Figure 1 [file EMBR-24-e57300-s009.zip › Fig 1/1E/T74A_LLOMe_LAMP1.tif]

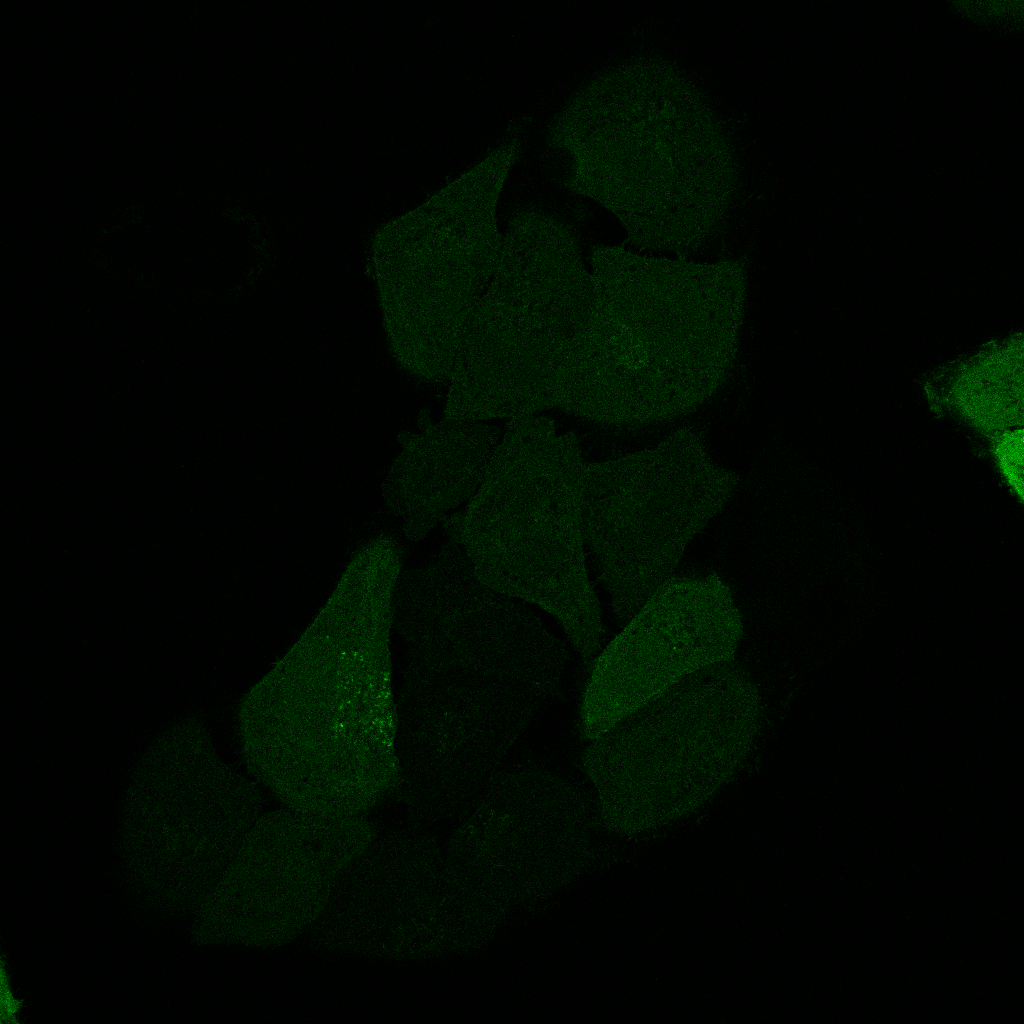

Supplement: Supplementary file 5 — Source Data for Figure 1 [file EMBR-24-e57300-s009.zip › Fig 1/1E/T444A_non-treated_mNG-STK38.tif]

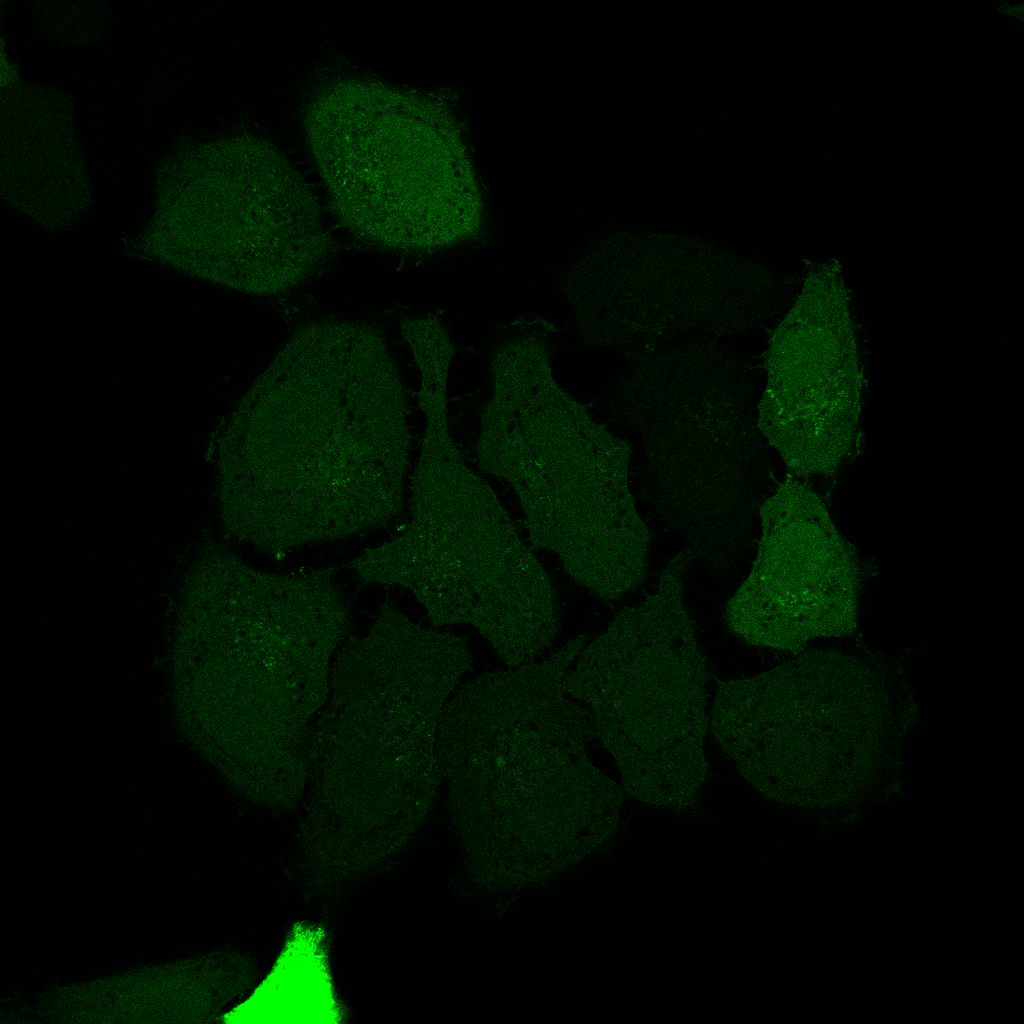

Supplement: Supplementary file 5 — Source Data for Figure 1 [file EMBR-24-e57300-s009.zip › Fig 1/1E/T74A_non-treated_mNG-STK38.tif]

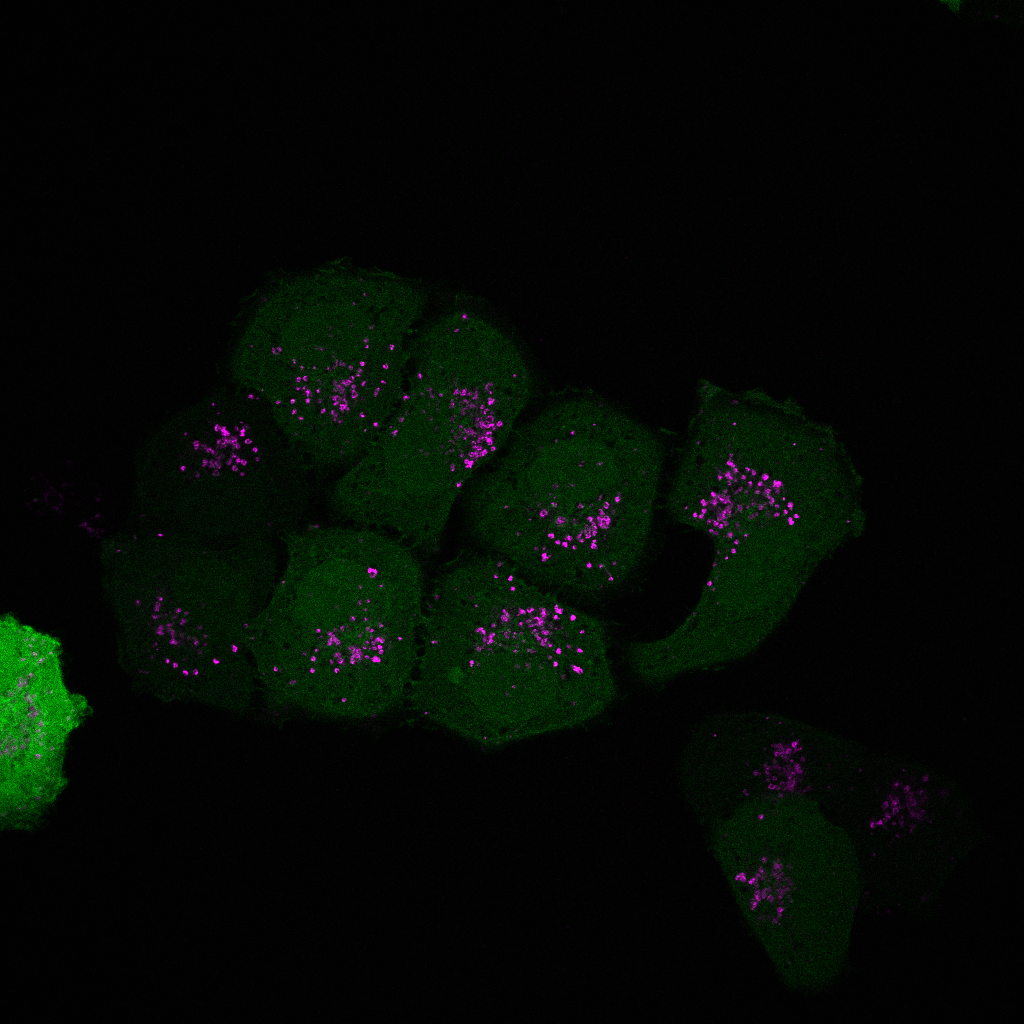

Supplement: Supplementary file 5 — Source Data for Figure 1 [file EMBR-24-e57300-s009.zip › Fig 1/1E/deltaC_non-treated_Merge.tif]

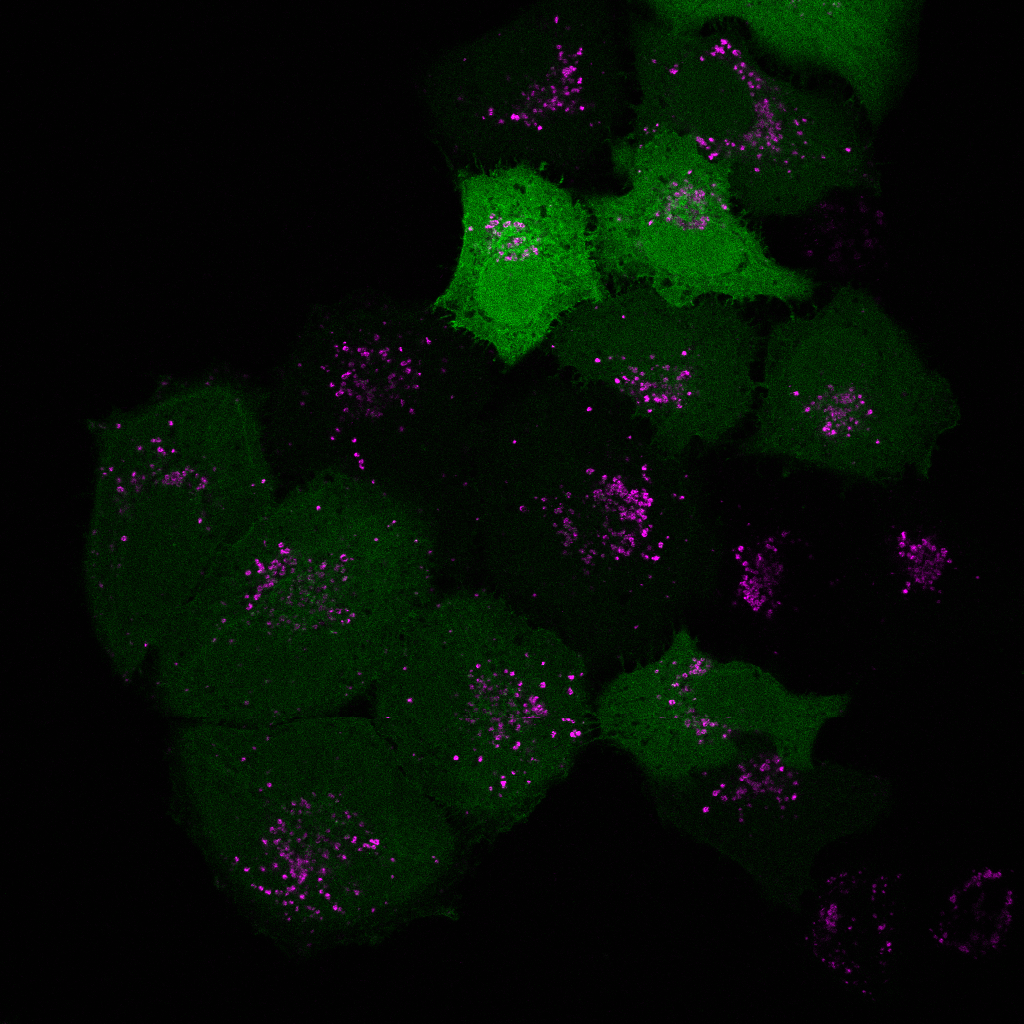

Supplement: Supplementary file 5 — Source Data for Figure 1 [file EMBR-24-e57300-s009.zip › Fig 1/1E/S281A_non-treated_Merge.tif]

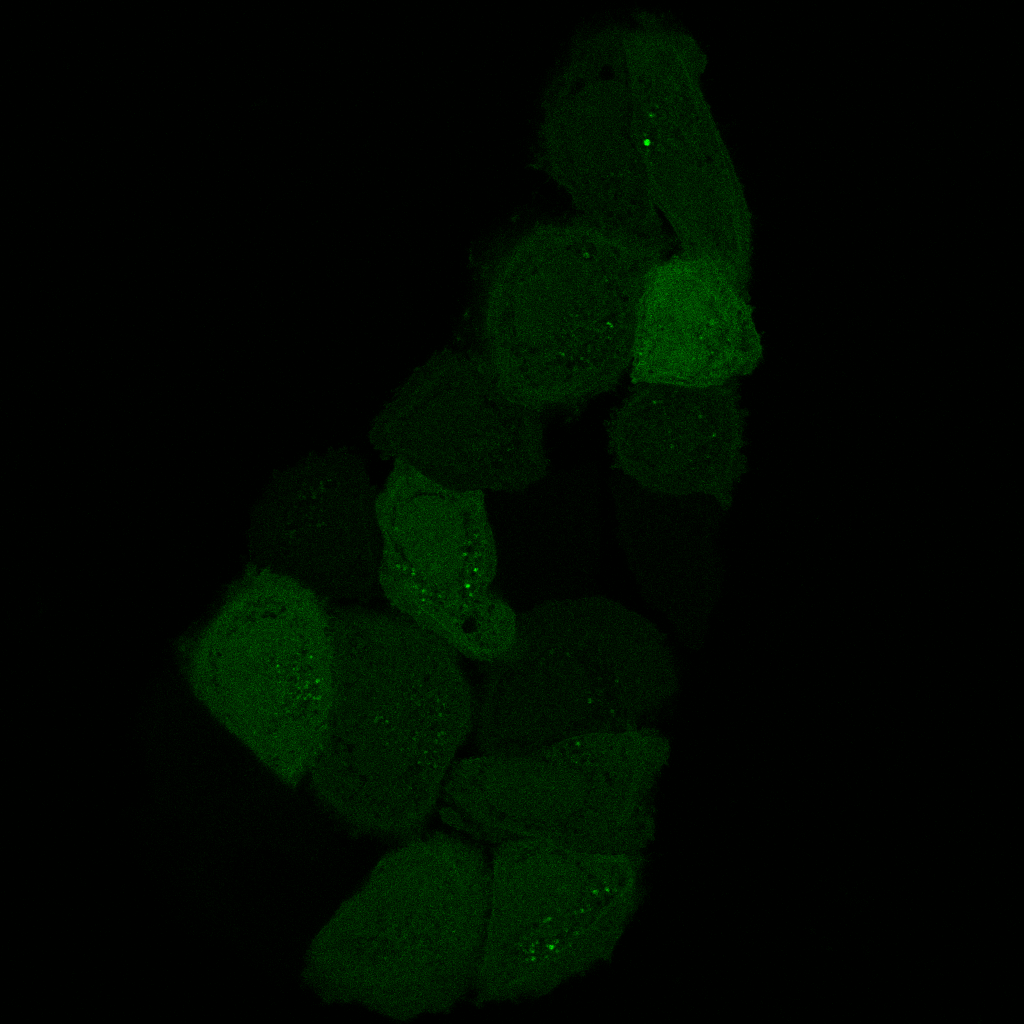

Supplement: Supplementary file 5 — Source Data for Figure 1 [file EMBR-24-e57300-s009.zip › Fig 1/1E/deltaC_LLOMe_mNG-STK38.tif]

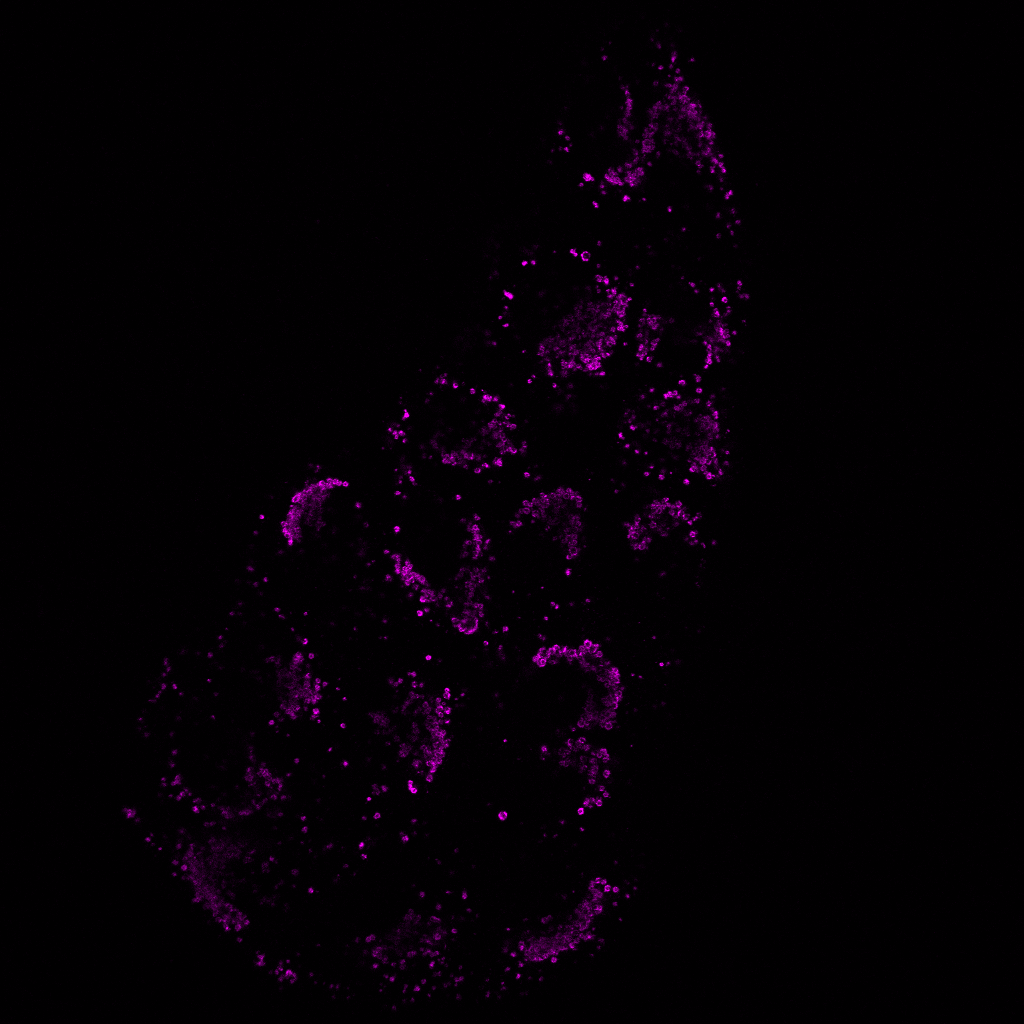

Supplement: Supplementary file 5 — Source Data for Figure 1 [file EMBR-24-e57300-s009.zip › Fig 1/1E/deltaC_LLOMe_LAMP1.tif]

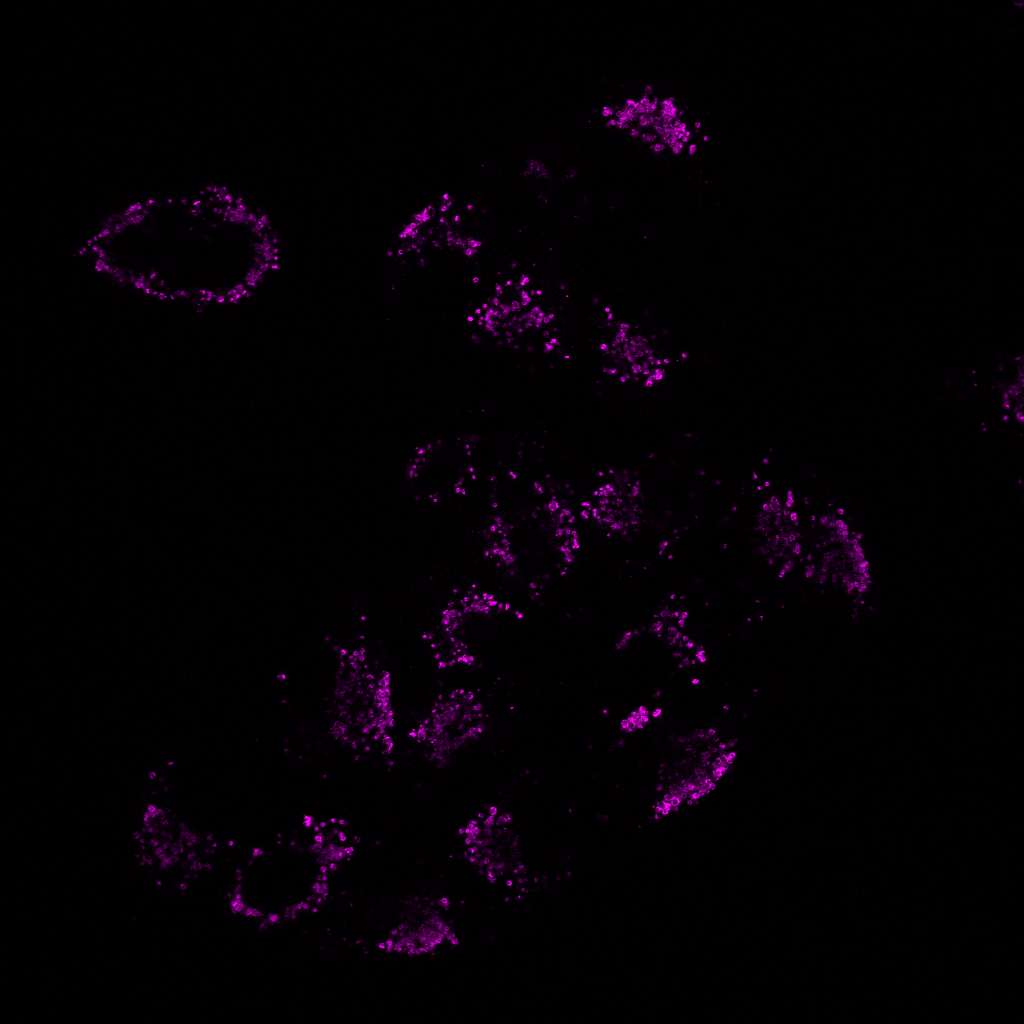

Supplement: Supplementary file 5 — Source Data for Figure 1 [file EMBR-24-e57300-s009.zip › Fig 1/1E/T444A_non-treated_LAMP1.tif]

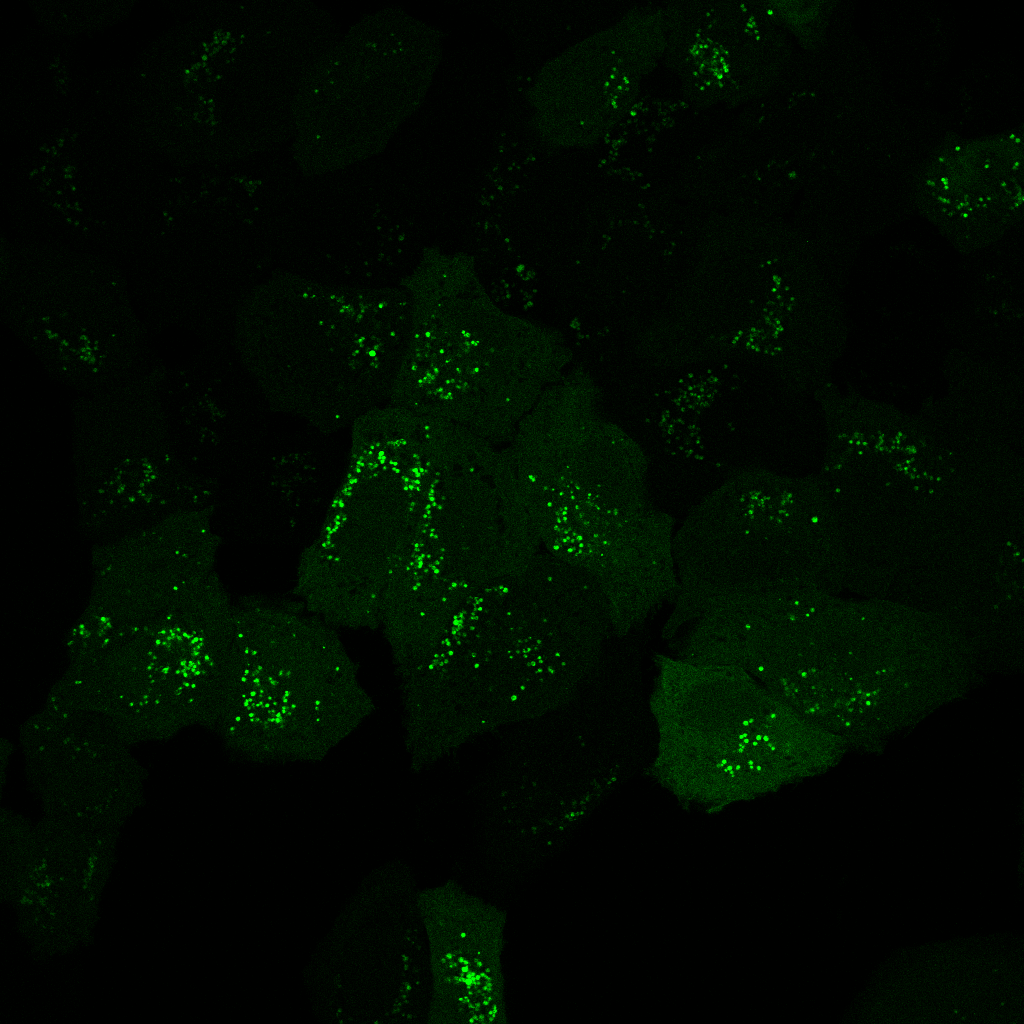

Supplement: Supplementary file 5 — Source Data for Figure 1 [file EMBR-24-e57300-s009.zip › Fig 1/1E/deltaN_LLOMe_mNG-STK38.tif]

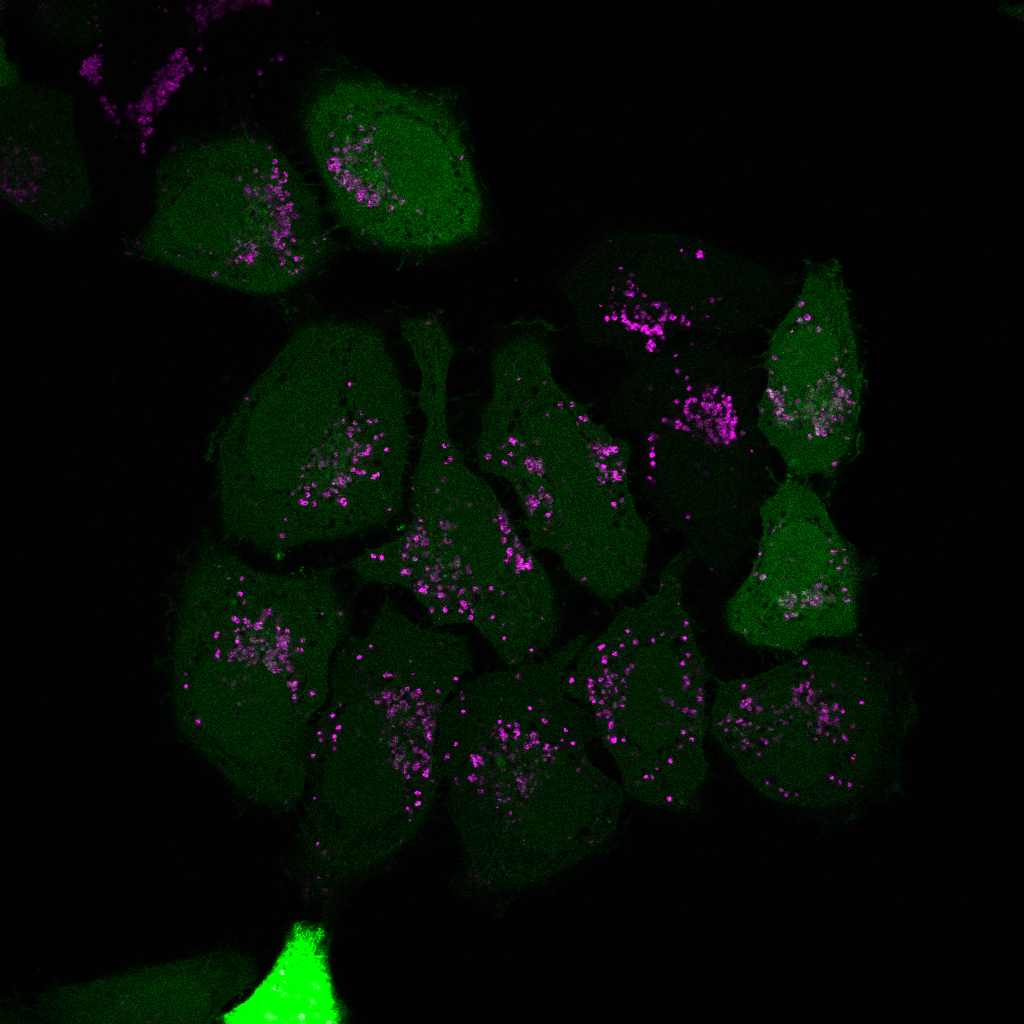

Supplement: Supplementary file 5 — Source Data for Figure 1 [file EMBR-24-e57300-s009.zip › Fig 1/1E/T74A_non-treated_Merge.tif]

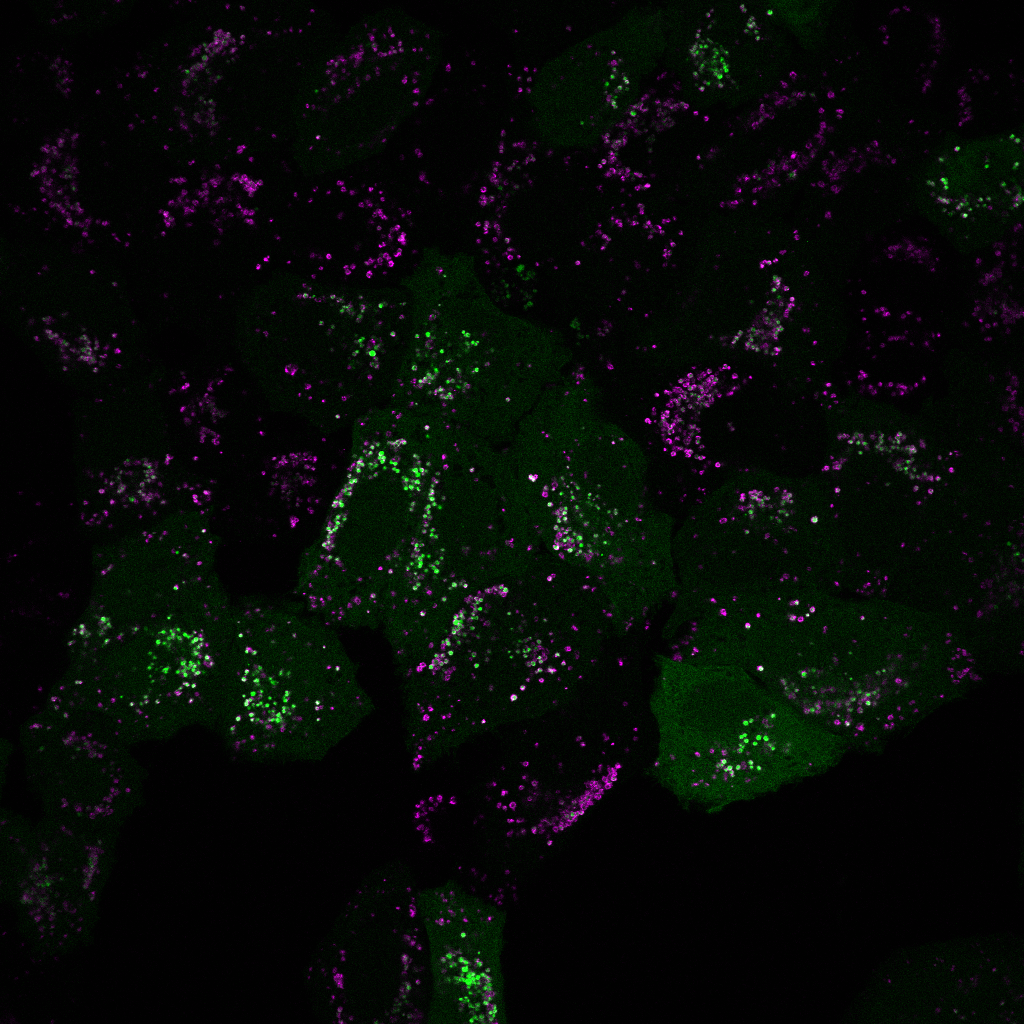

Supplement: Supplementary file 5 — Source Data for Figure 1 [file EMBR-24-e57300-s009.zip › Fig 1/1E/deltaN_LLOMe_Merge.tif]
