# Supplementary figures and images for: Microautophagy regulated by STK38 and GABARAPs is essential to repair lysosomes and prevent aging (part 2 of 4)
Source: EMBO Rep. 2023 Nov 21;24(12):e57300. doi: 10.15252/embr.202357300 (PMC10702834; doi:10.15252/embr.202357300)

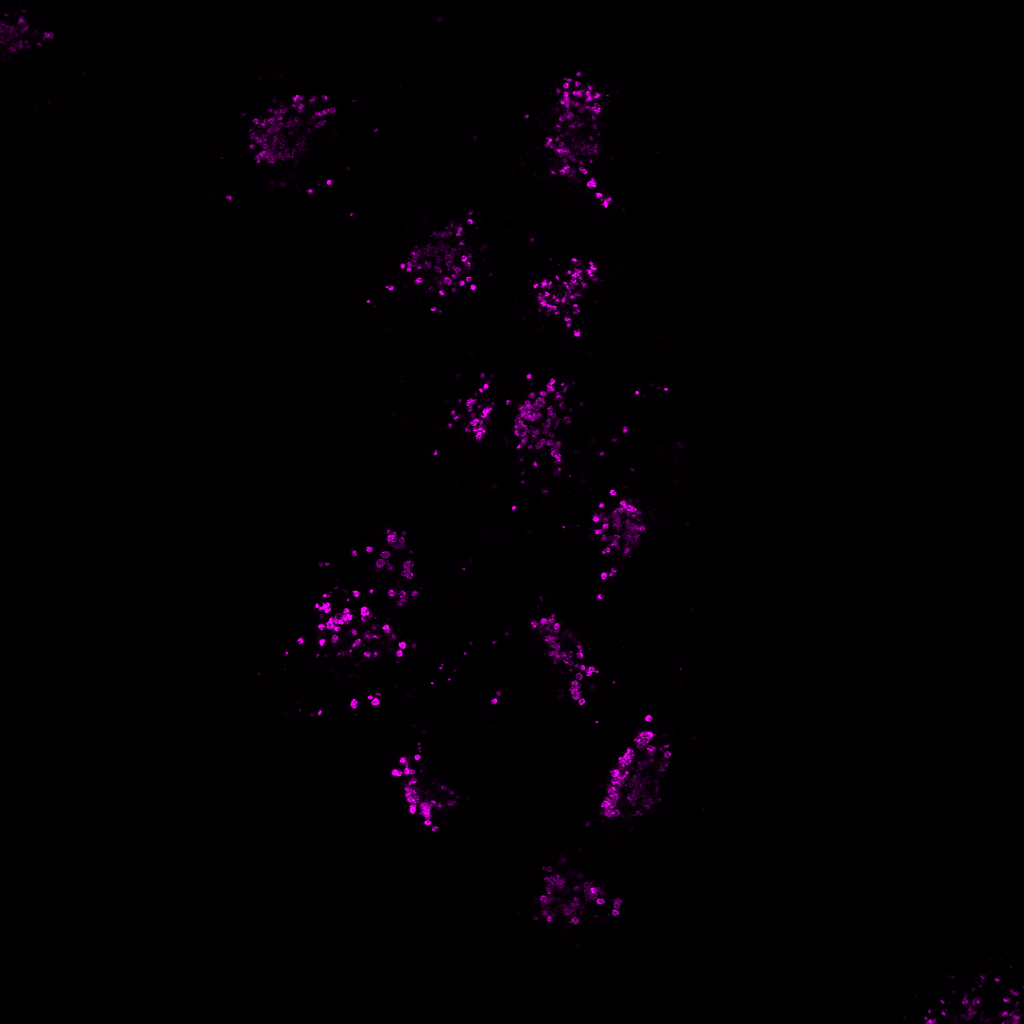

Supplement: Supplementary file 5 — Source Data for Figure 1 [file EMBR-24-e57300-s009.zip › Fig 1/1E/deltaN_non-treated_LAMP1.tif]

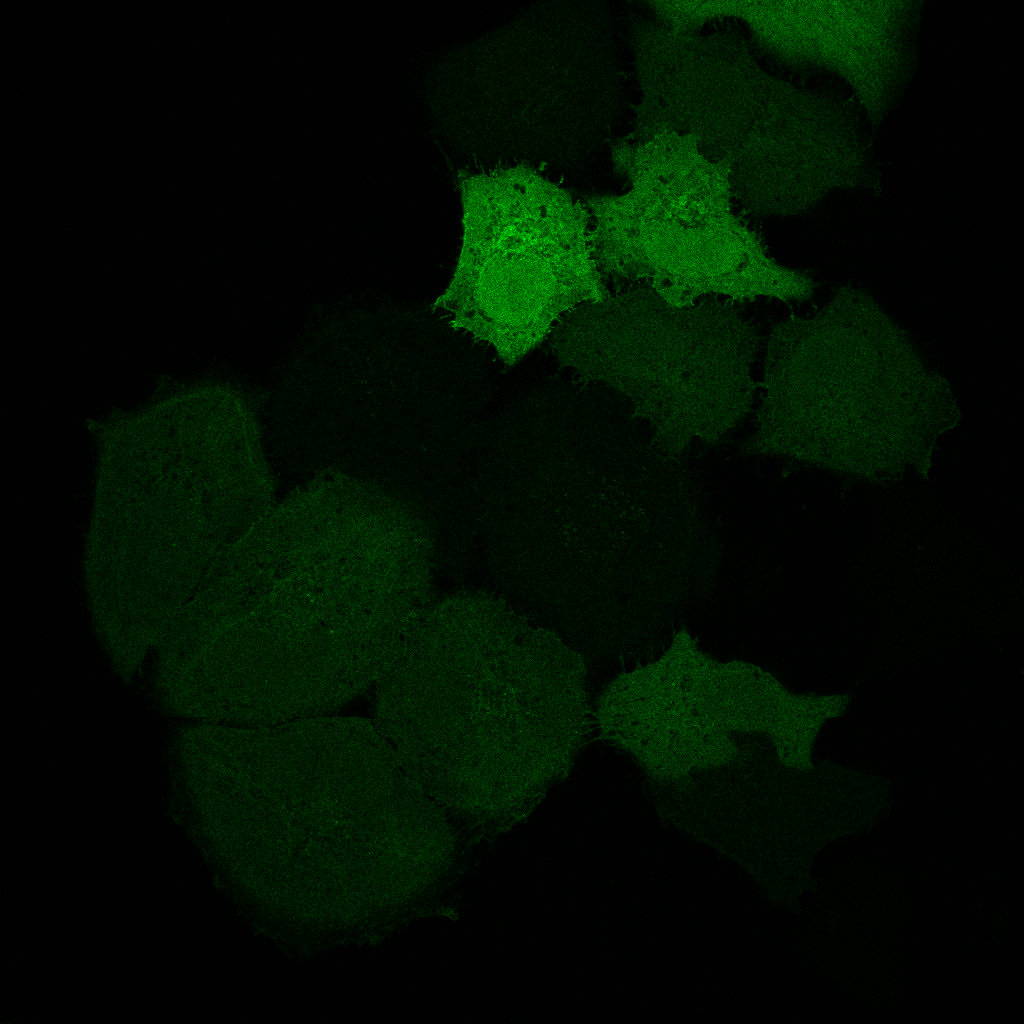

Supplement: Supplementary file 5 — Source Data for Figure 1 [file EMBR-24-e57300-s009.zip › Fig 1/1E/S281A_non-treated_mNG-STK38.tif]

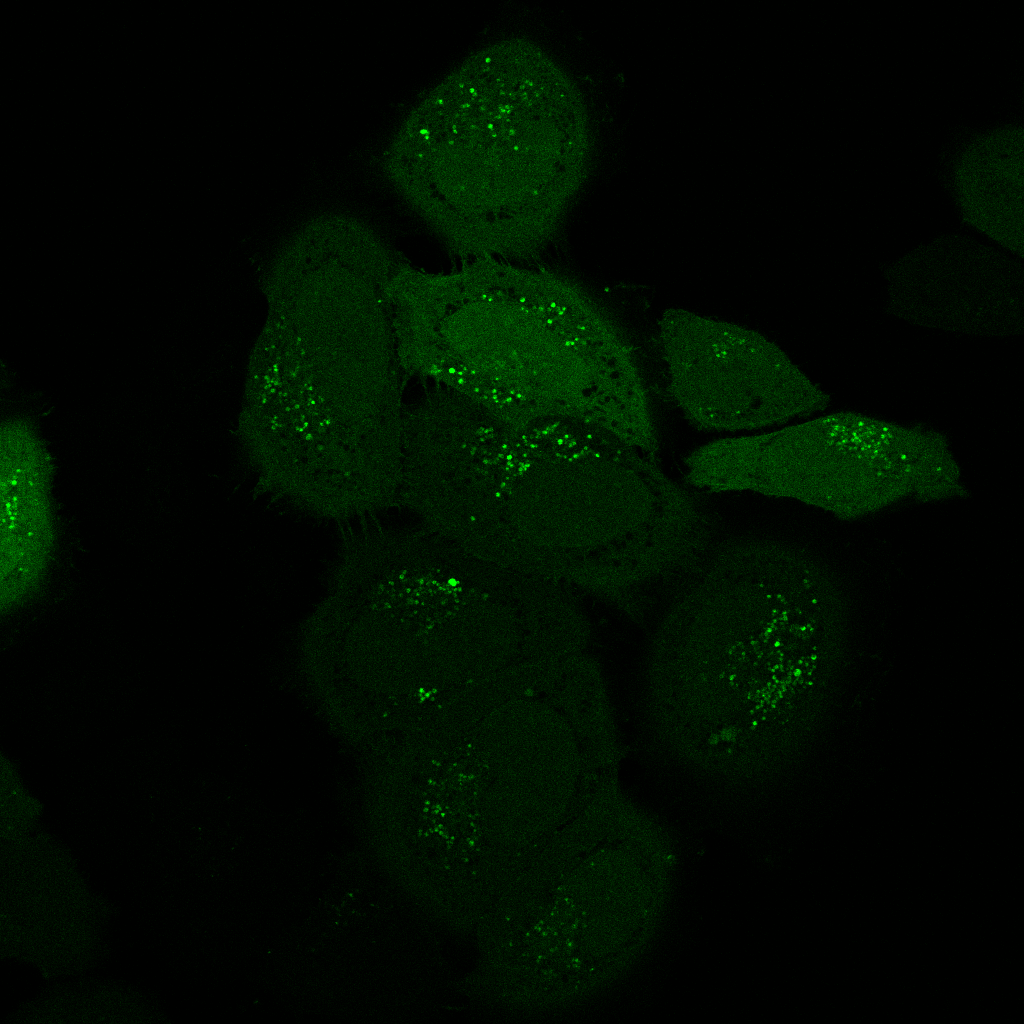

Supplement: Supplementary file 5 — Source Data for Figure 1 [file EMBR-24-e57300-s009.zip › Fig 1/1E/WT_LLOMe_mNG-STK38.tif]

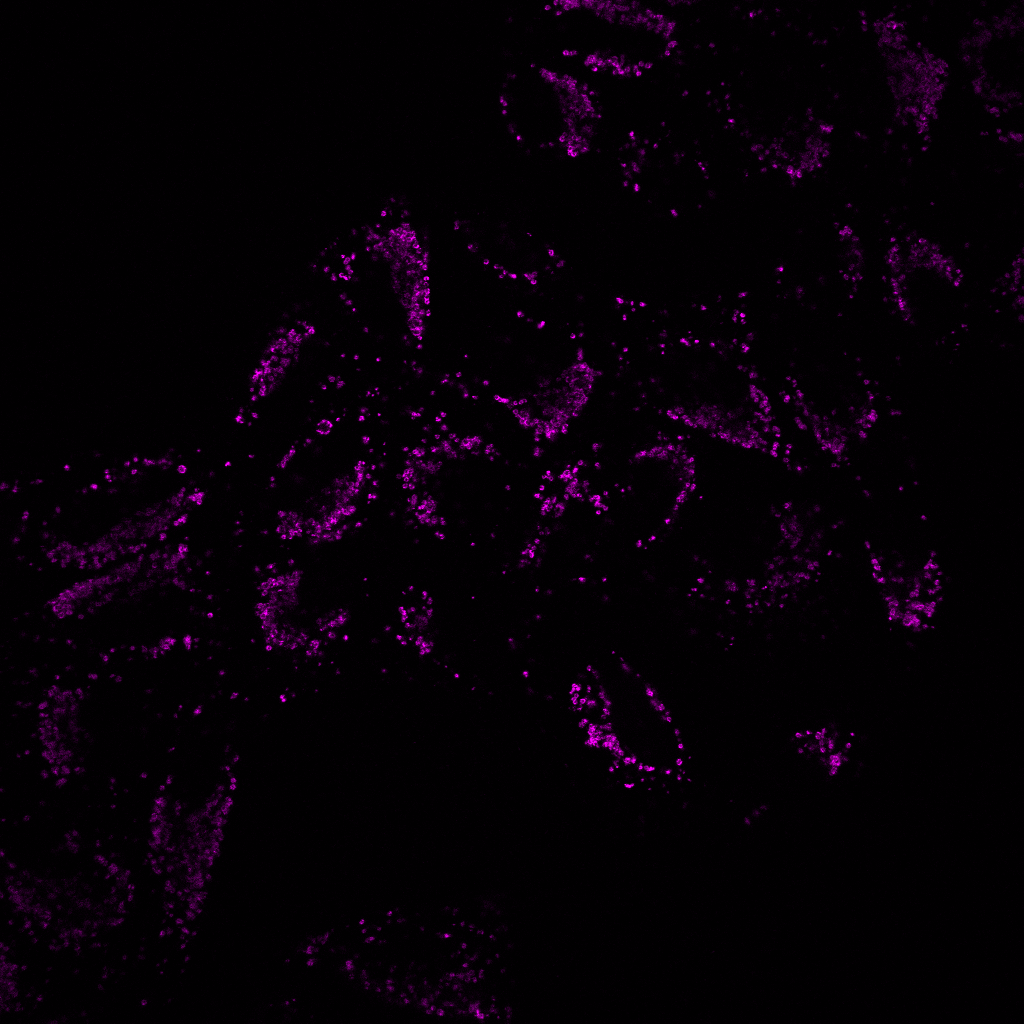

Supplement: Supplementary file 5 — Source Data for Figure 1 [file EMBR-24-e57300-s009.zip › Fig 1/1E/S281A_LLOMe_LAMP1.tif]

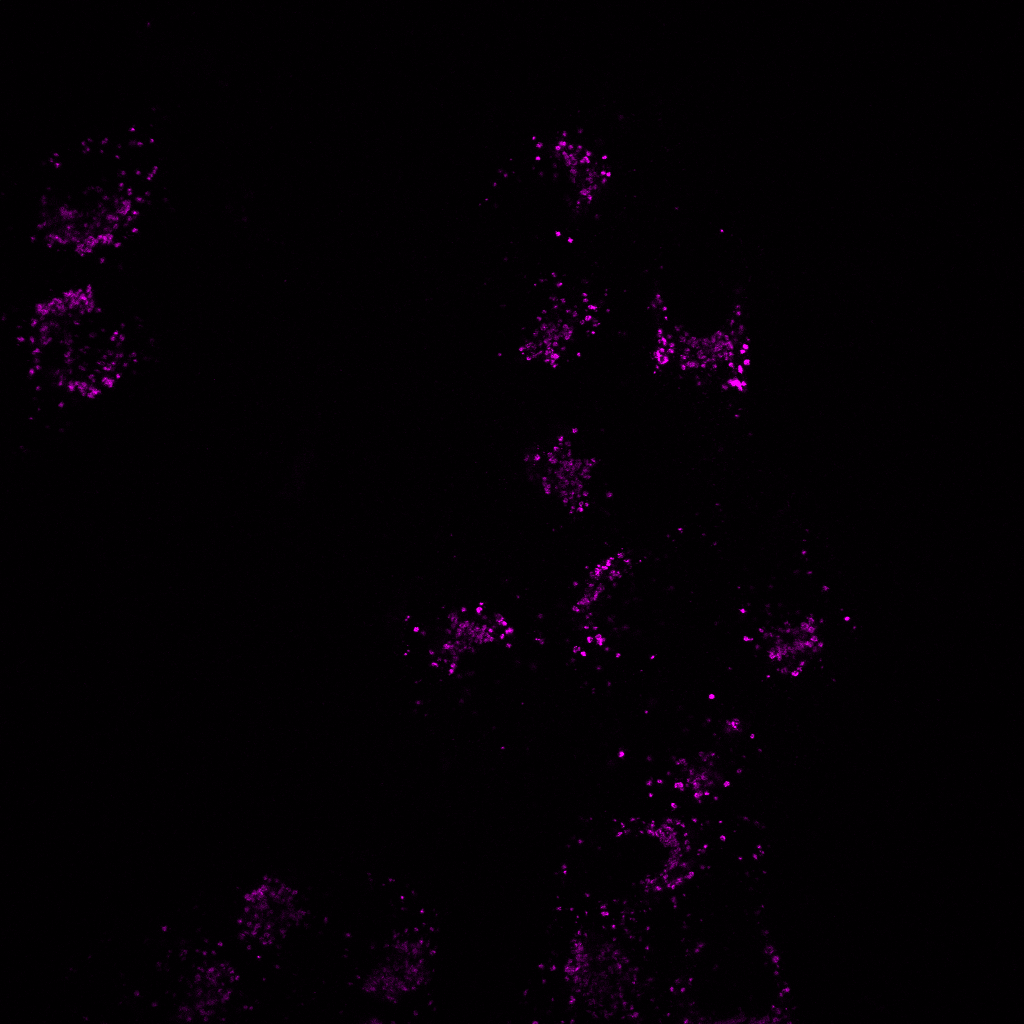

Supplement: Supplementary file 5 — Source Data for Figure 1 [file EMBR-24-e57300-s009.zip › Fig 1/1E/WT_non-treated_LAMP1.tif]

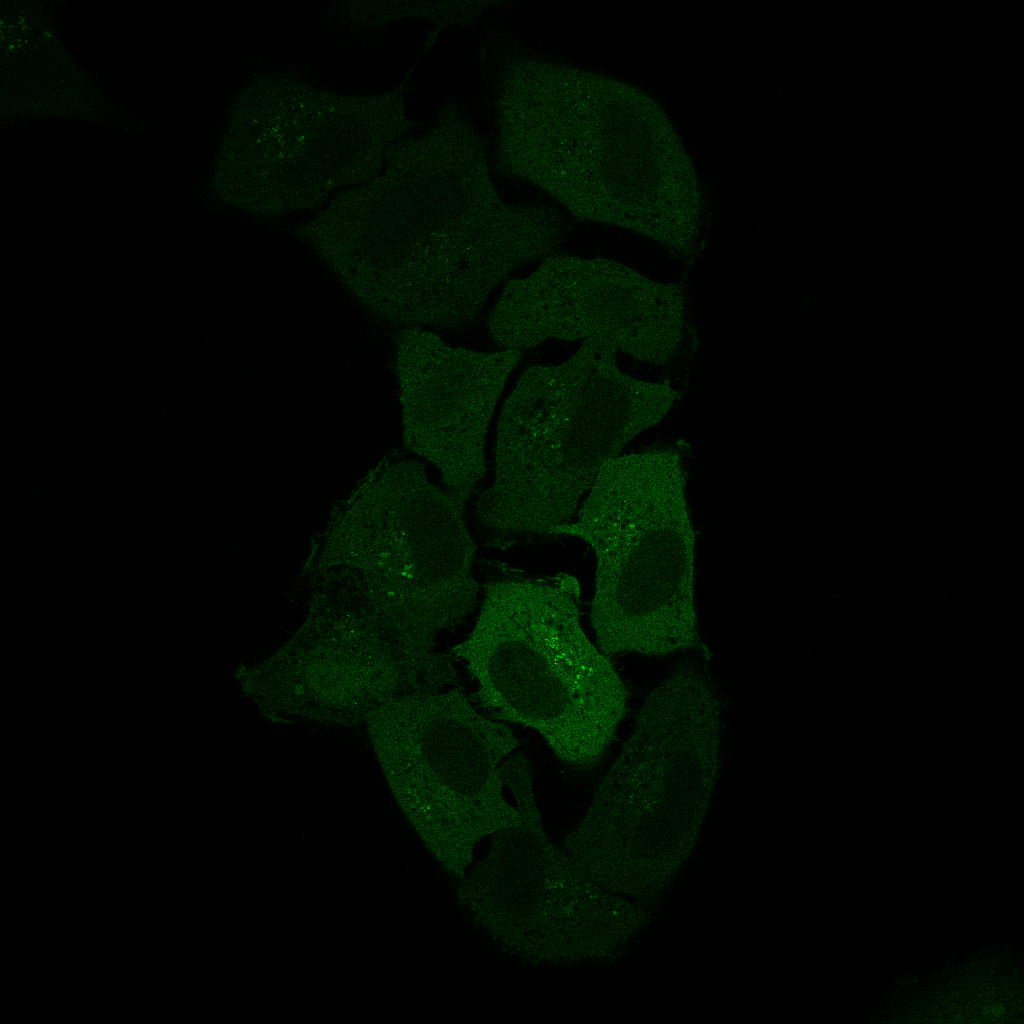

Supplement: Supplementary file 5 — Source Data for Figure 1 [file EMBR-24-e57300-s009.zip › Fig 1/1E/deltaN_non-treated_mNG-STK38.tif]

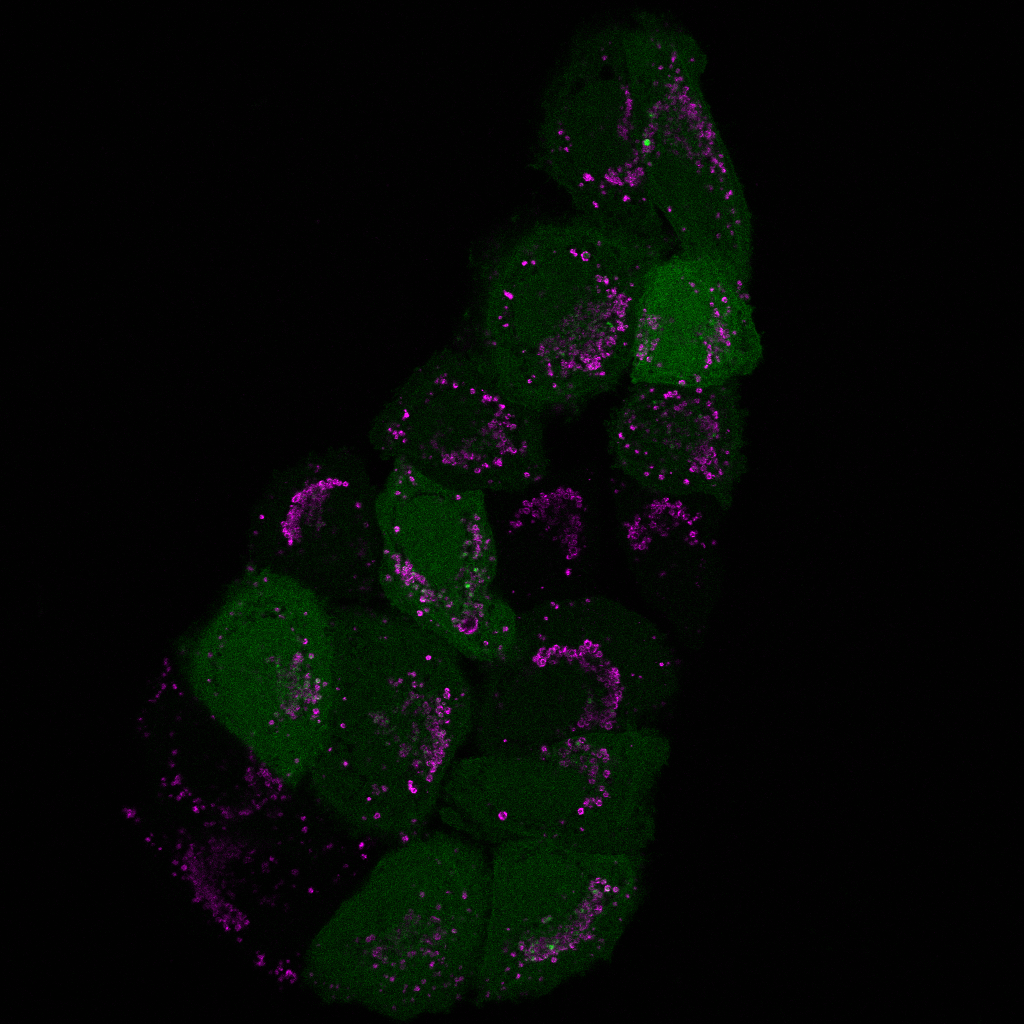

Supplement: Supplementary file 5 — Source Data for Figure 1 [file EMBR-24-e57300-s009.zip › Fig 1/1E/deltaC_LLOMe_Merge.tif]

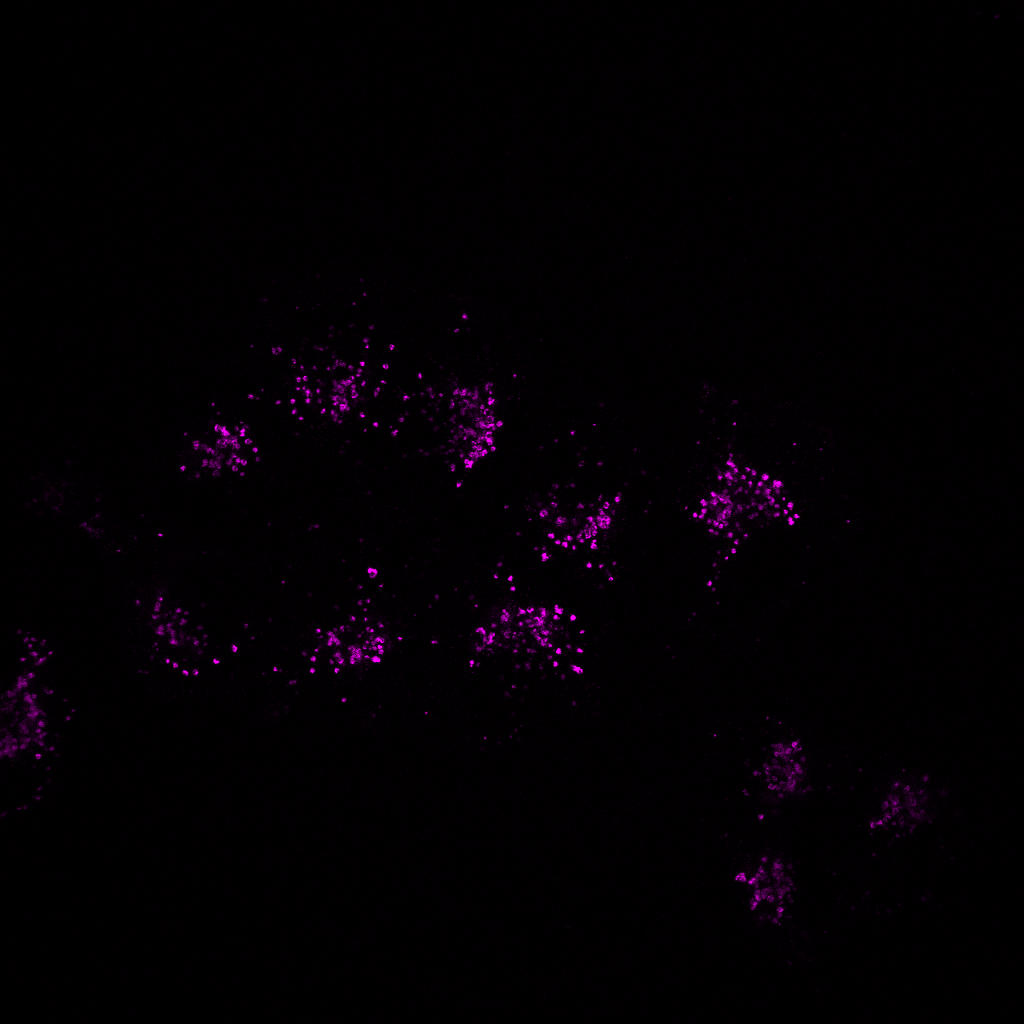

Supplement: Supplementary file 5 — Source Data for Figure 1 [file EMBR-24-e57300-s009.zip › Fig 1/1E/deltaC_non-treated_LAMP1.tif]

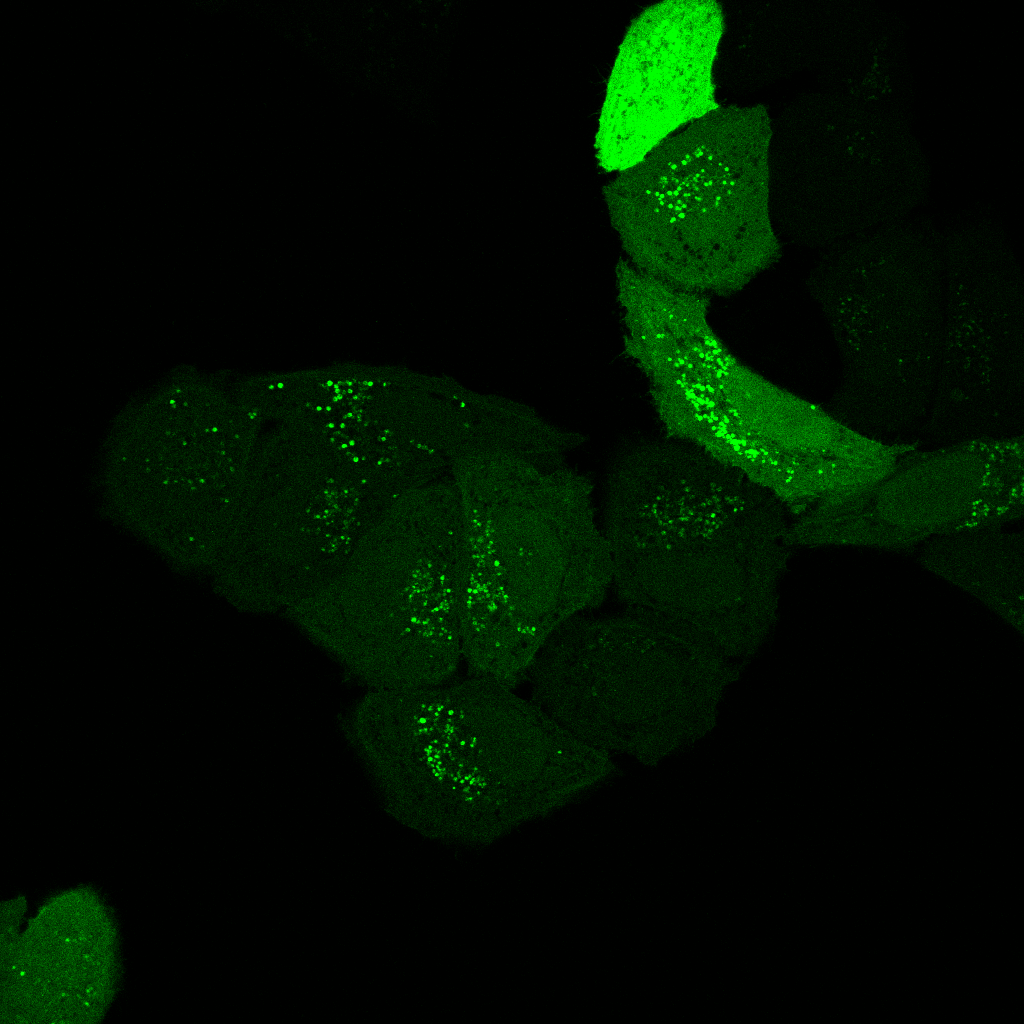

Supplement: Supplementary file 5 — Source Data for Figure 1 [file EMBR-24-e57300-s009.zip › Fig 1/1E/T74A_LLOMe_mNG-STK38.tif]

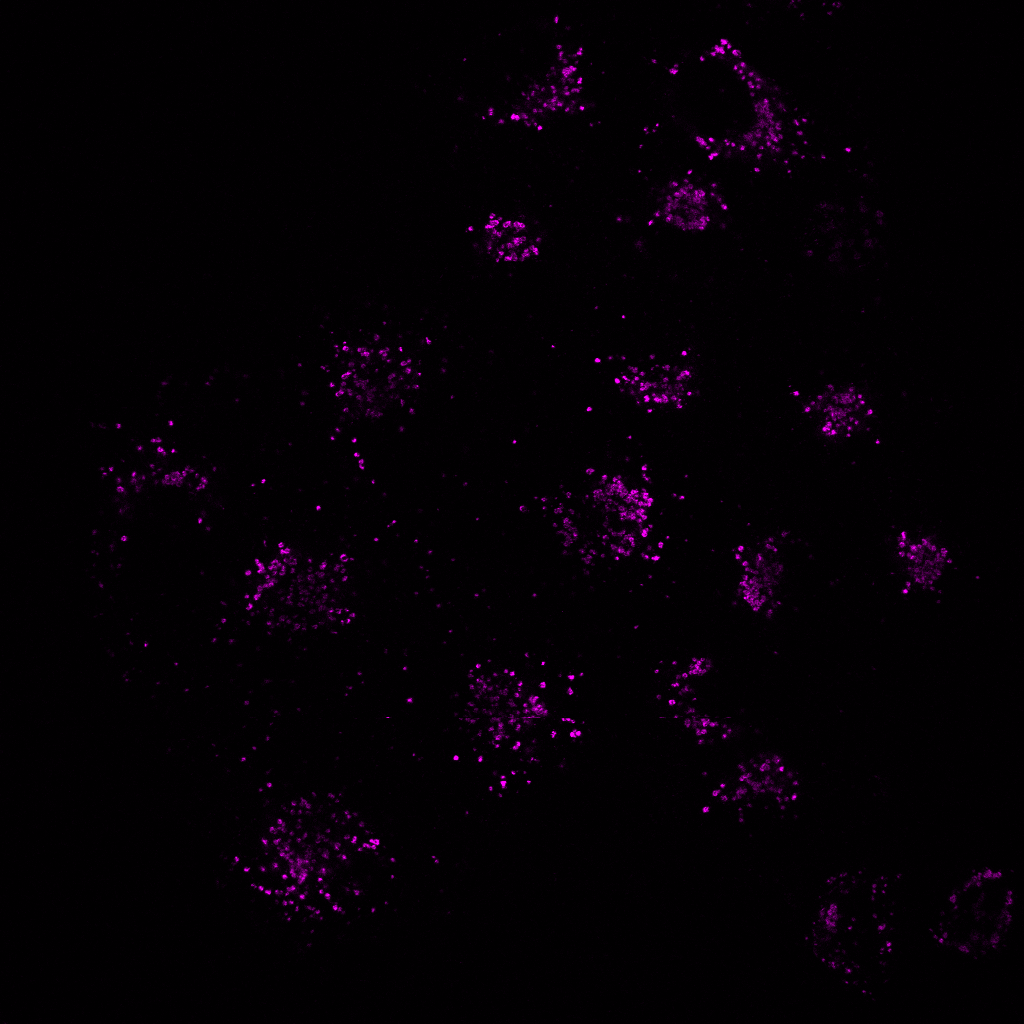

Supplement: Supplementary file 5 — Source Data for Figure 1 [file EMBR-24-e57300-s009.zip › Fig 1/1E/S281A_non-treated_LAMP1.tif]

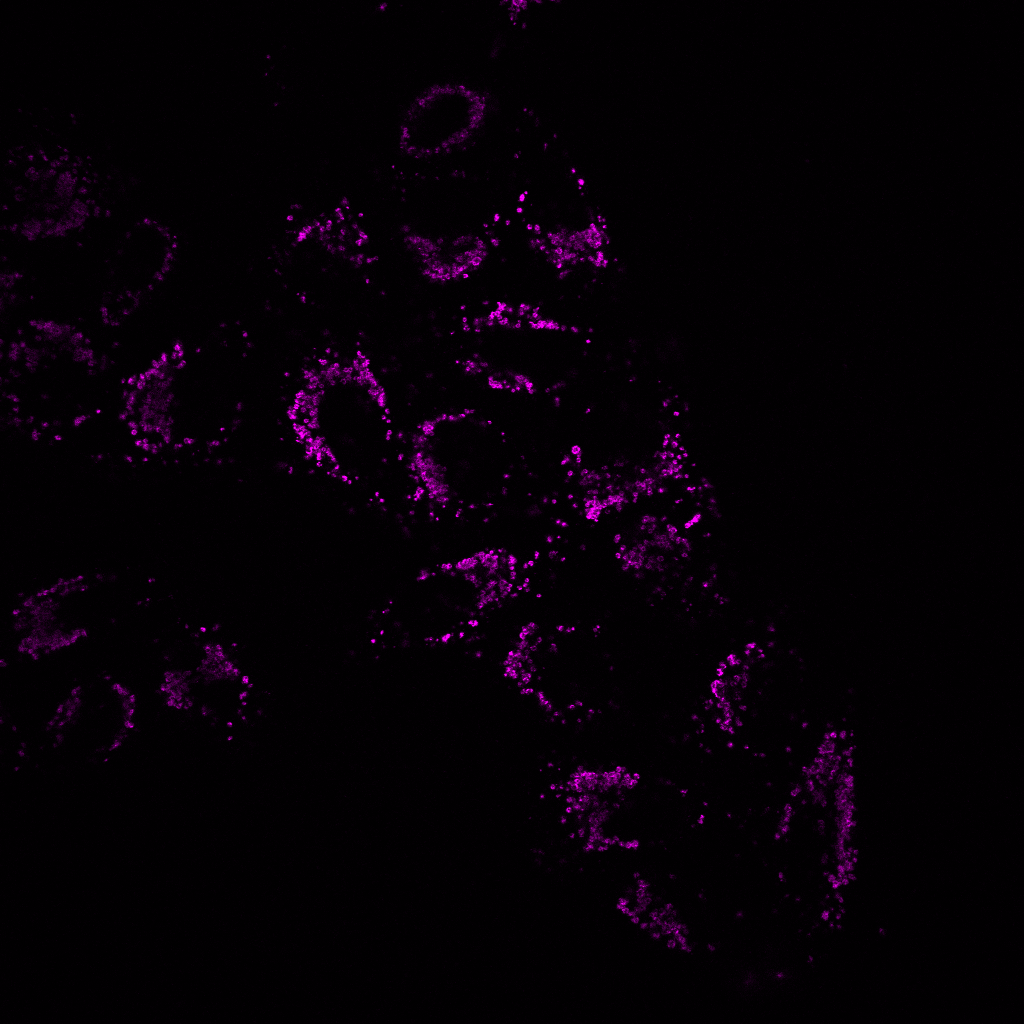

Supplement: Supplementary file 5 — Source Data for Figure 1 [file EMBR-24-e57300-s009.zip › Fig 1/1E/T444A_LLOMe_LAMP1.tif]

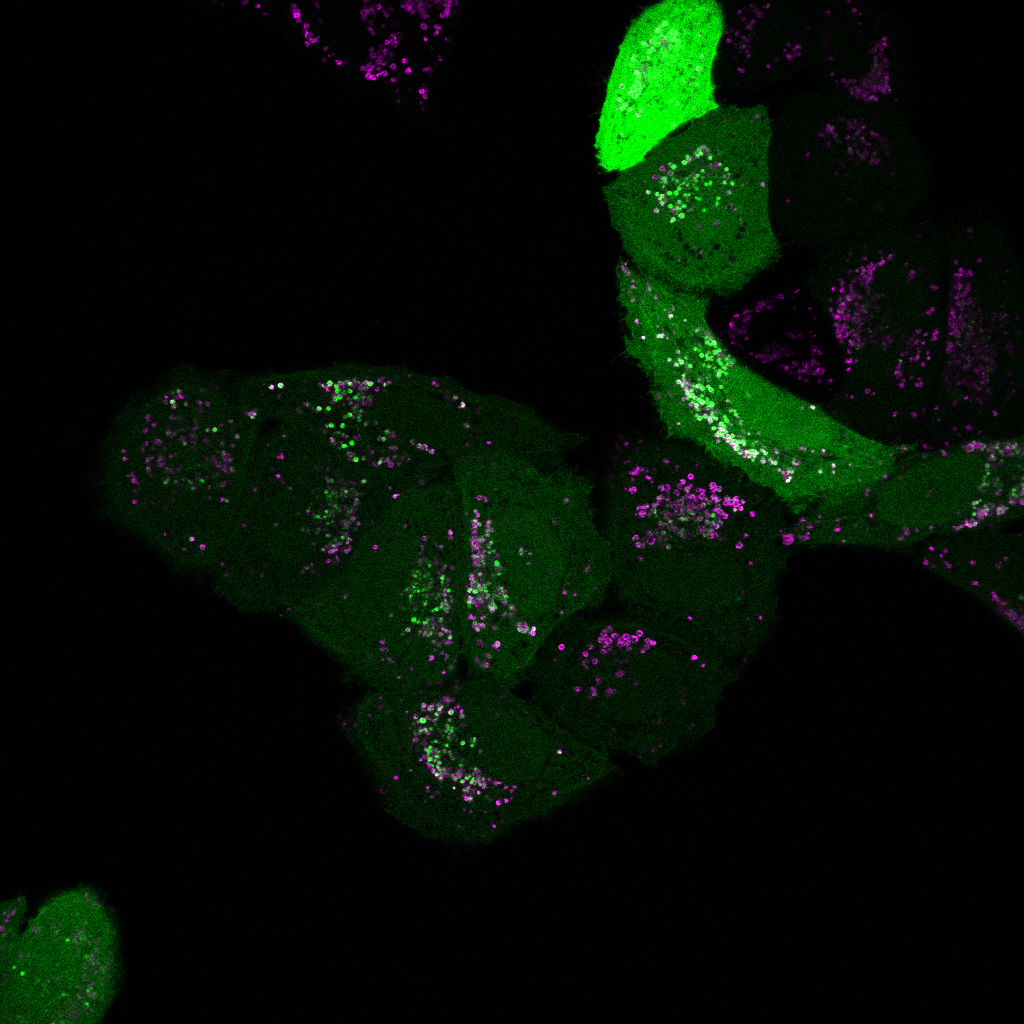

Supplement: Supplementary file 5 — Source Data for Figure 1 [file EMBR-24-e57300-s009.zip › Fig 1/1E/T74A_LLOMe_Merge.tif]

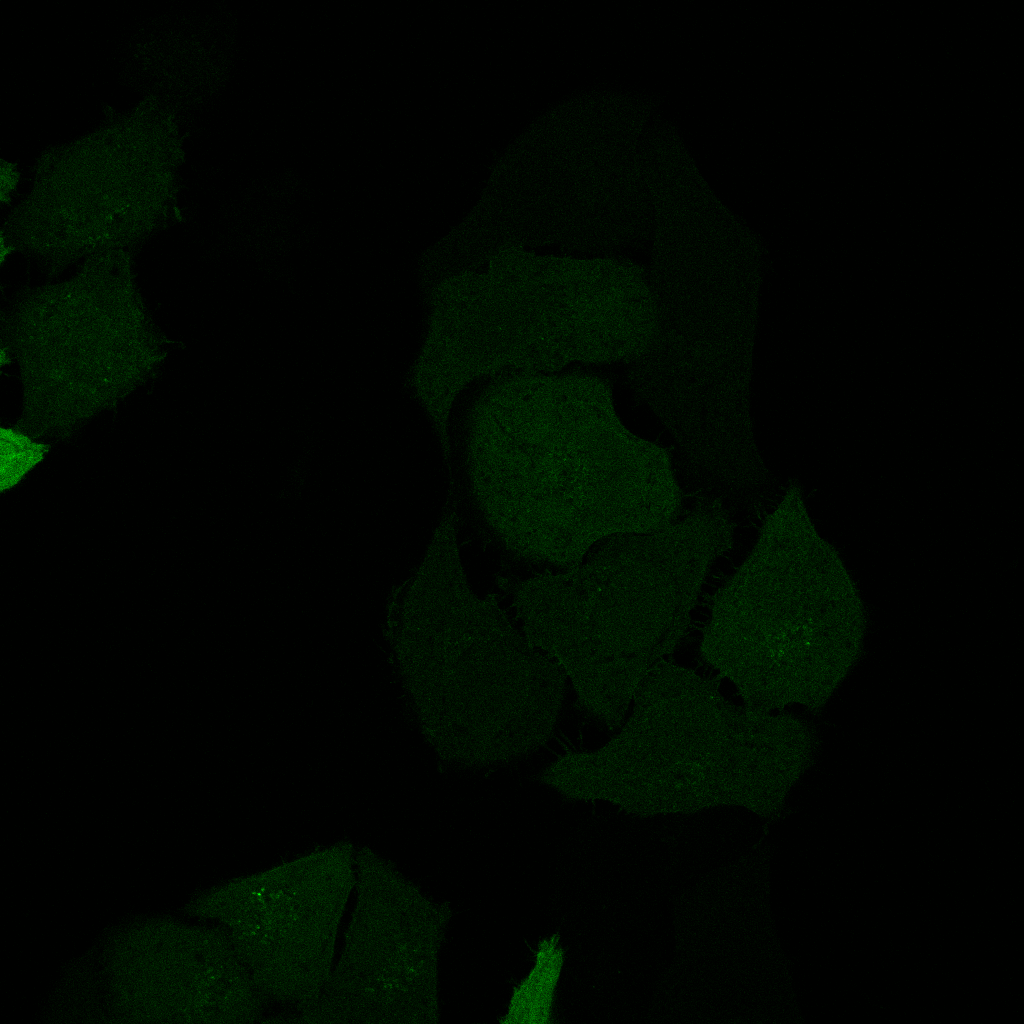

Supplement: Supplementary file 5 — Source Data for Figure 1 [file EMBR-24-e57300-s009.zip › Fig 1/1E/WT_non-treated_mNG-STK38.tif]

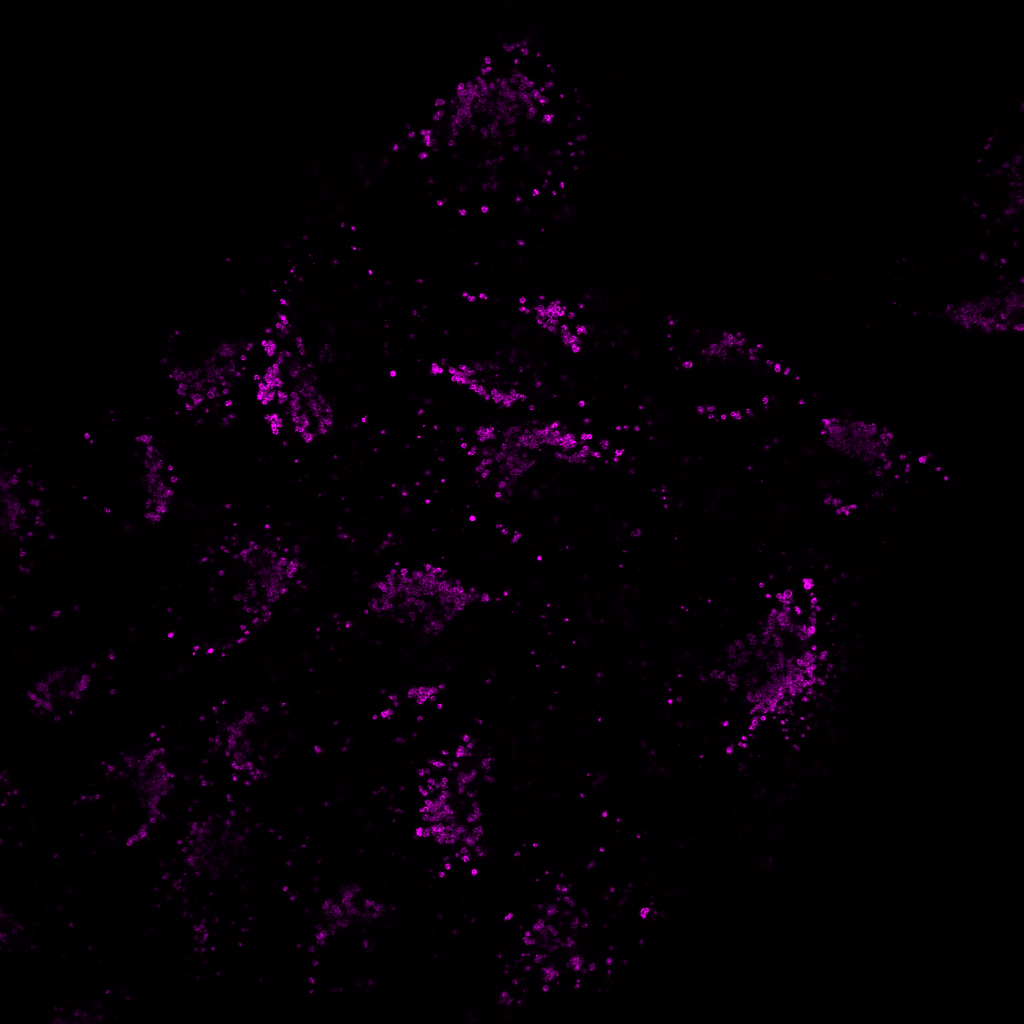

Supplement: Supplementary file 5 — Source Data for Figure 1 [file EMBR-24-e57300-s009.zip › Fig 1/1E/WT_LLOMe_LAMP1.tif]

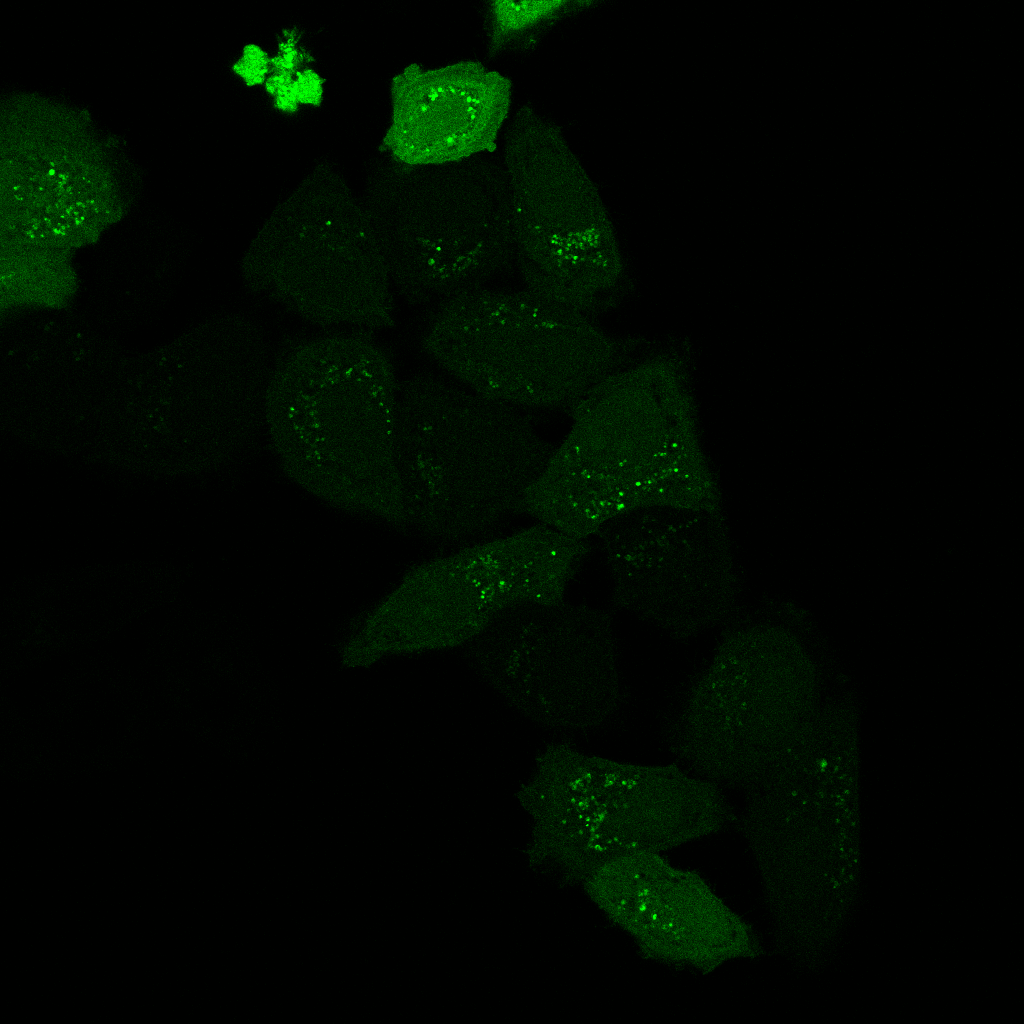

Supplement: Supplementary file 5 — Source Data for Figure 1 [file EMBR-24-e57300-s009.zip › Fig 1/1E/T444A_LLOMe_mNG-STK38.tif]

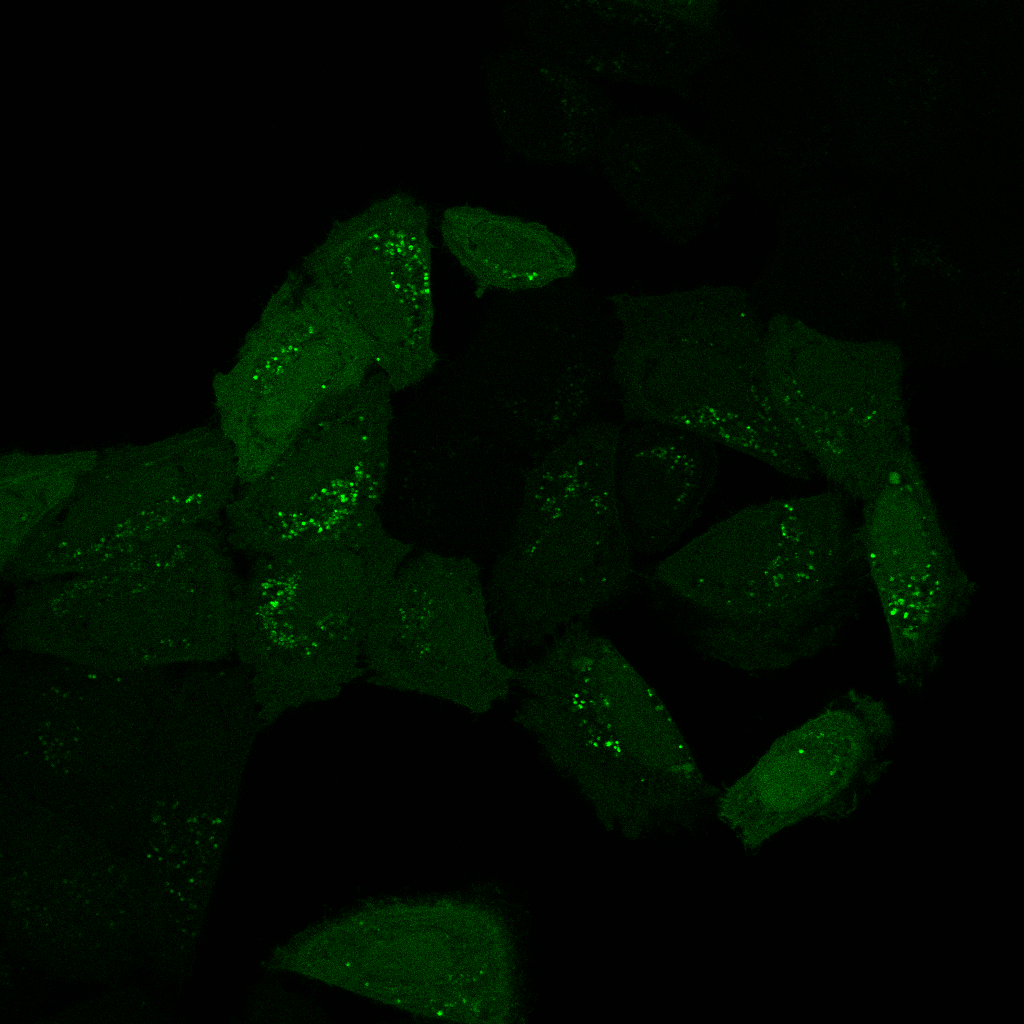

Supplement: Supplementary file 5 — Source Data for Figure 1 [file EMBR-24-e57300-s009.zip › Fig 1/1E/S281A_LLOMe_mNG-STK38.tif]

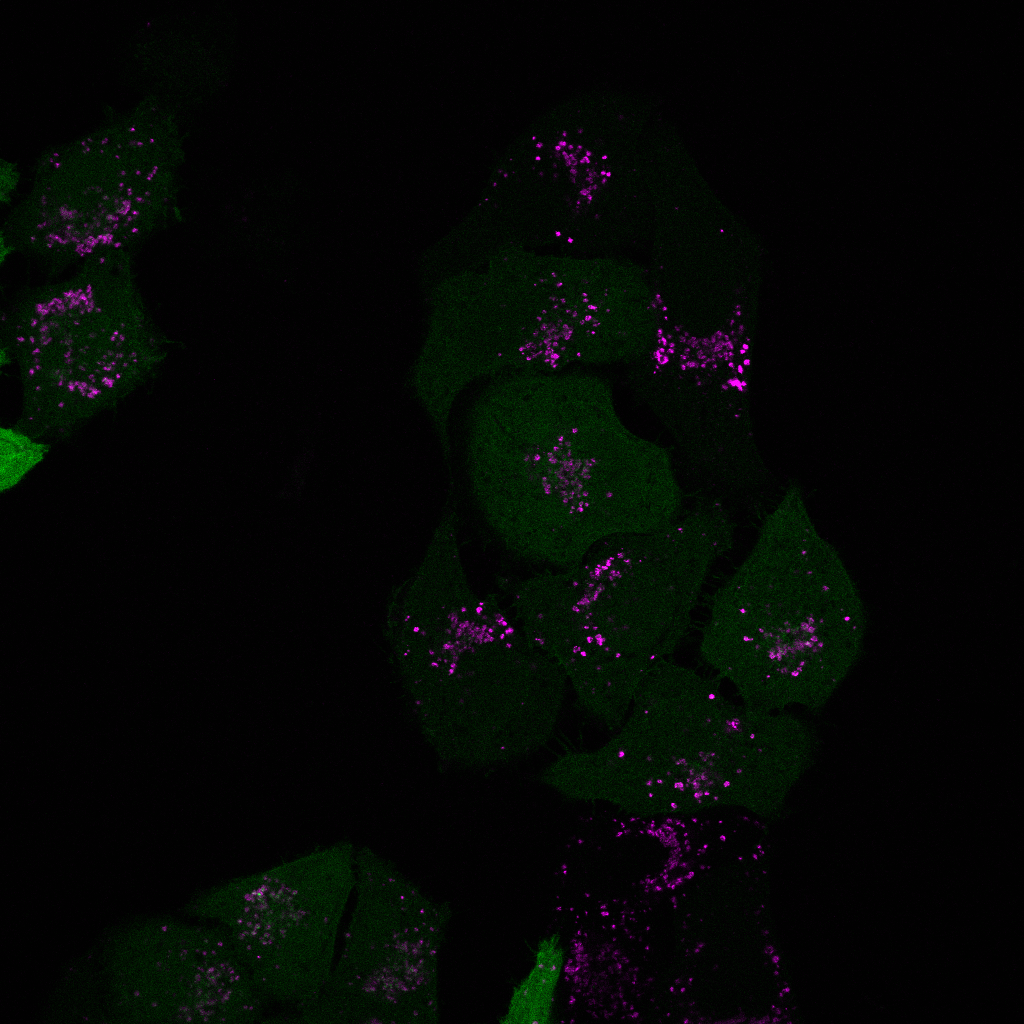

Supplement: Supplementary file 5 — Source Data for Figure 1 [file EMBR-24-e57300-s009.zip › Fig 1/1E/WT_non-treated_Merge.tif]

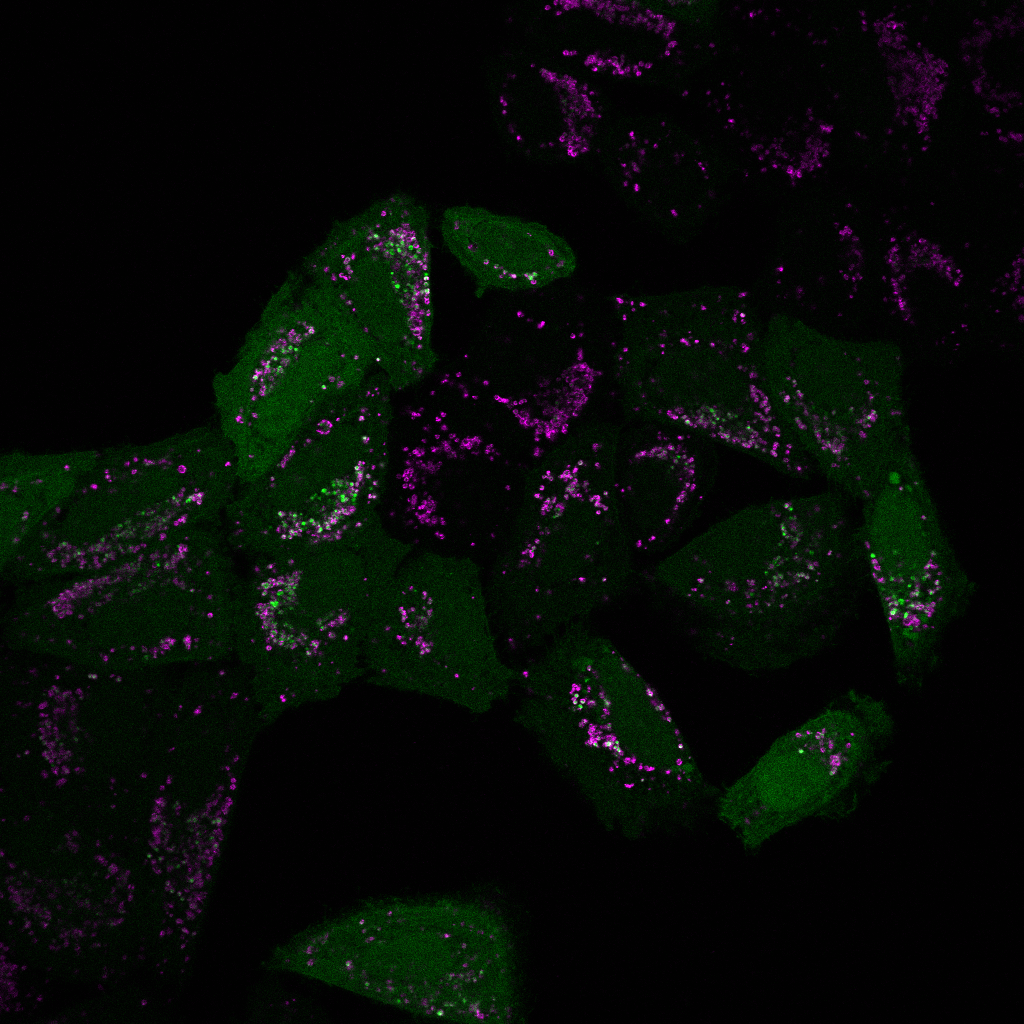

Supplement: Supplementary file 5 — Source Data for Figure 1 [file EMBR-24-e57300-s009.zip › Fig 1/1E/S281A_LLOMe_Merge.tif]

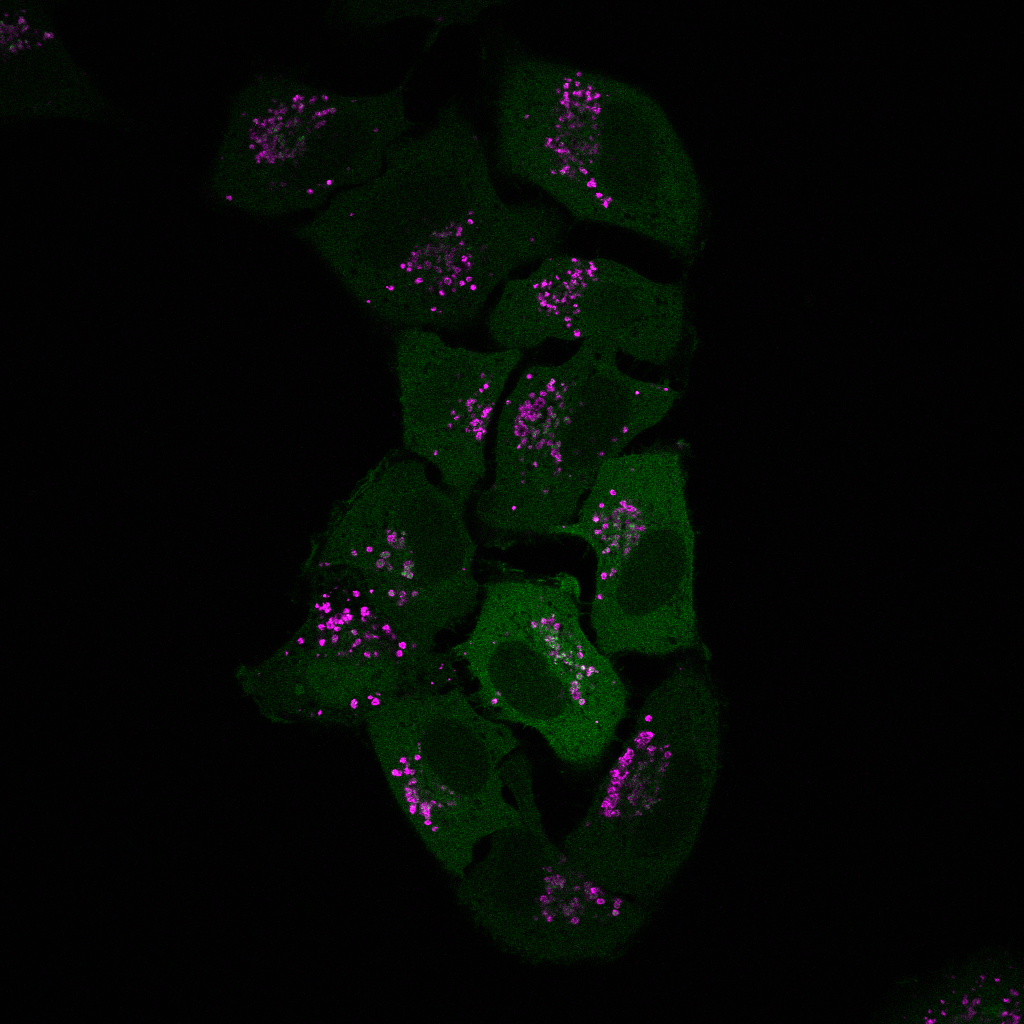

Supplement: Supplementary file 5 — Source Data for Figure 1 [file EMBR-24-e57300-s009.zip › Fig 1/1E/deltaN_non-treated_Merge.tif]

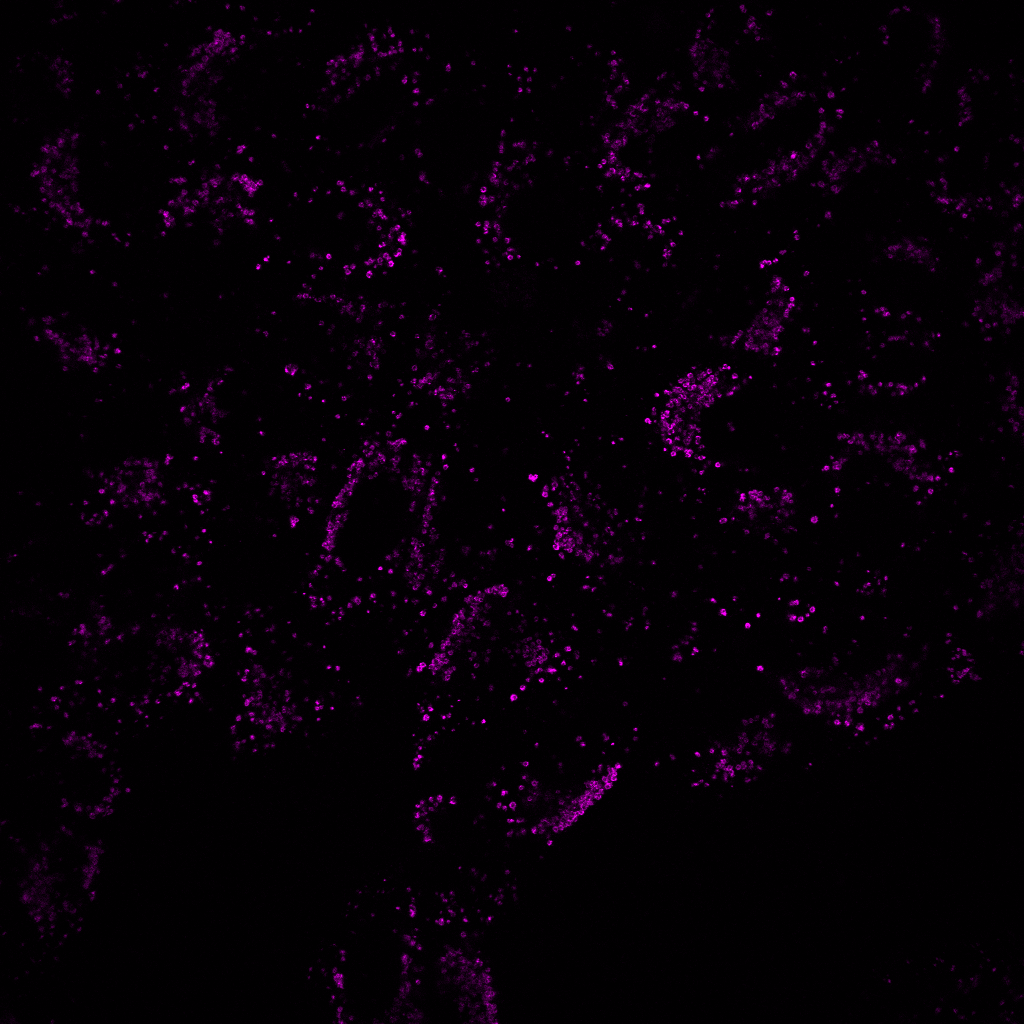

Supplement: Supplementary file 5 — Source Data for Figure 1 [file EMBR-24-e57300-s009.zip › Fig 1/1E/deltaN_LLOMe_LAMP1.tif]

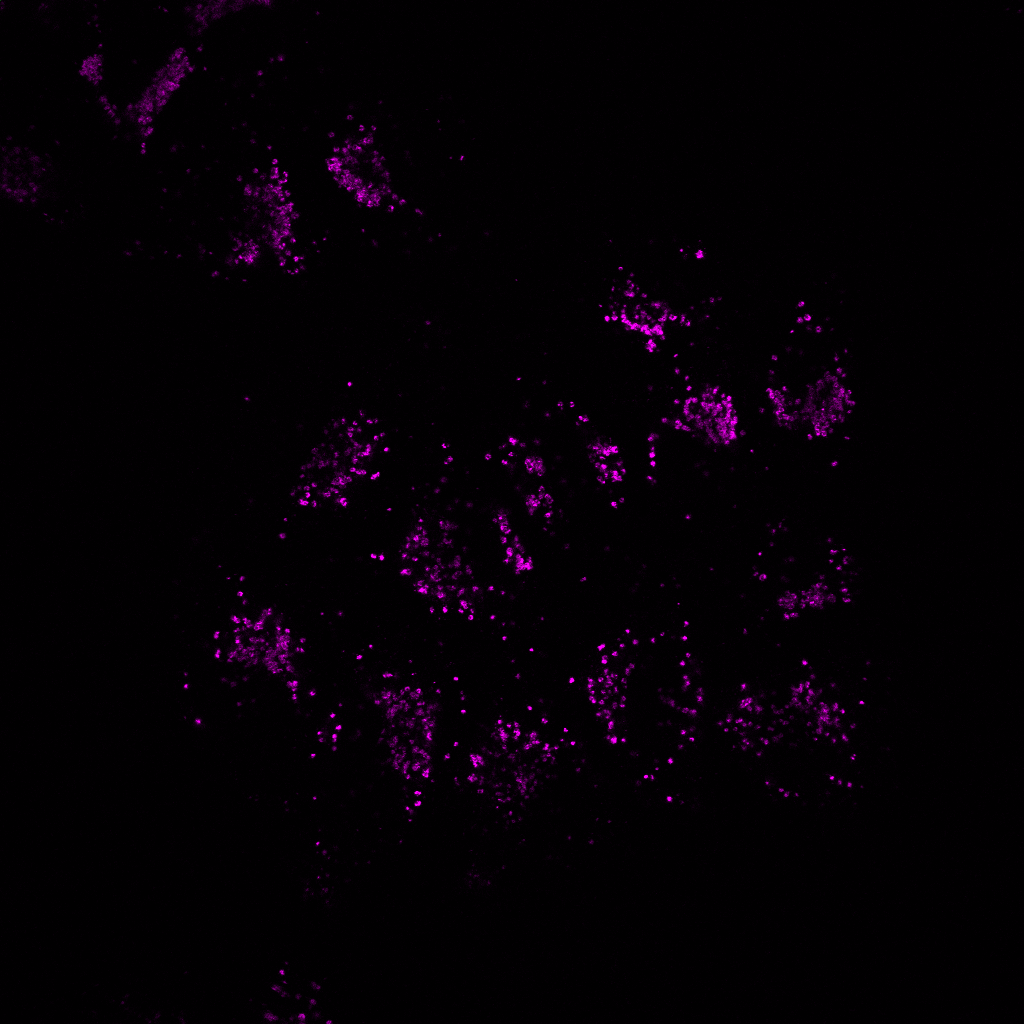

Supplement: Supplementary file 5 — Source Data for Figure 1 [file EMBR-24-e57300-s009.zip › Fig 1/1E/T74A_non-treated_LAMP1.tif]

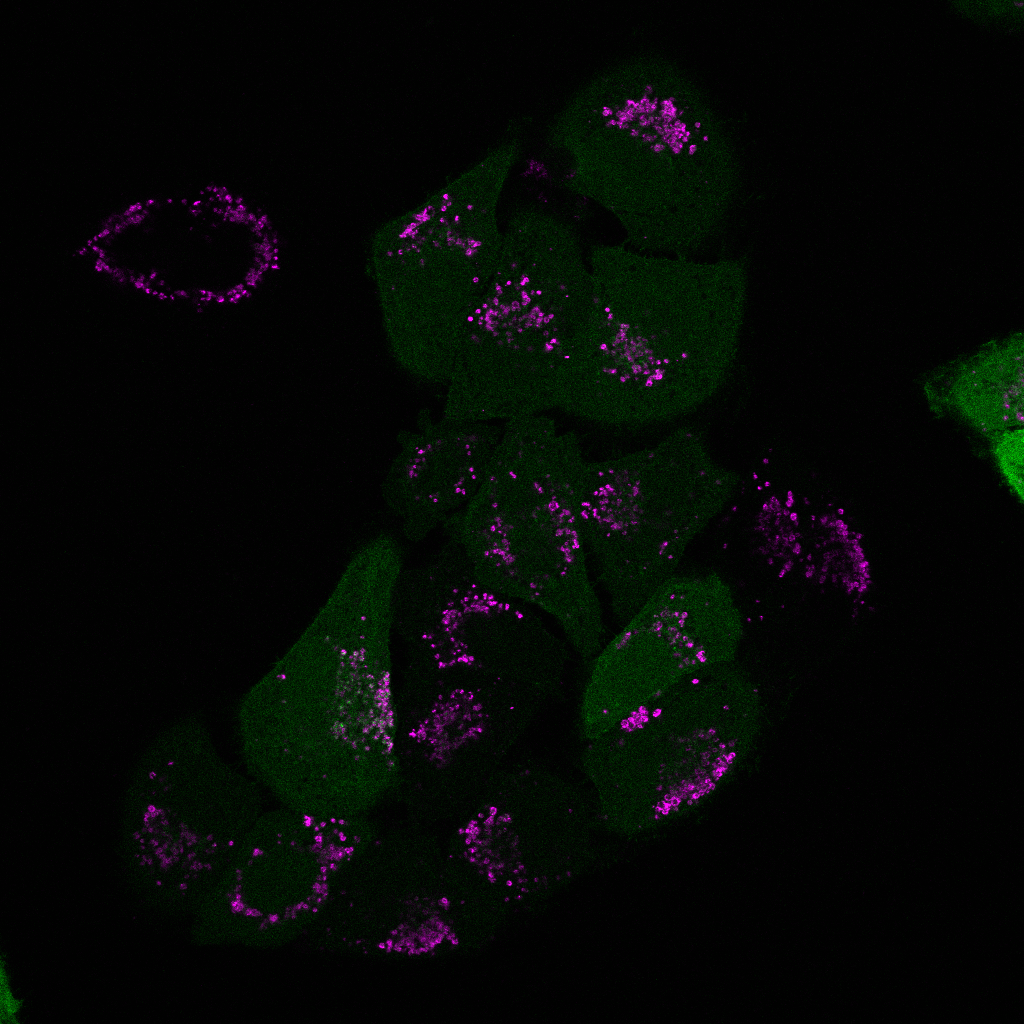

Supplement: Supplementary file 5 — Source Data for Figure 1 [file EMBR-24-e57300-s009.zip › Fig 1/1E/T444A_non-treated_Merge.tif]

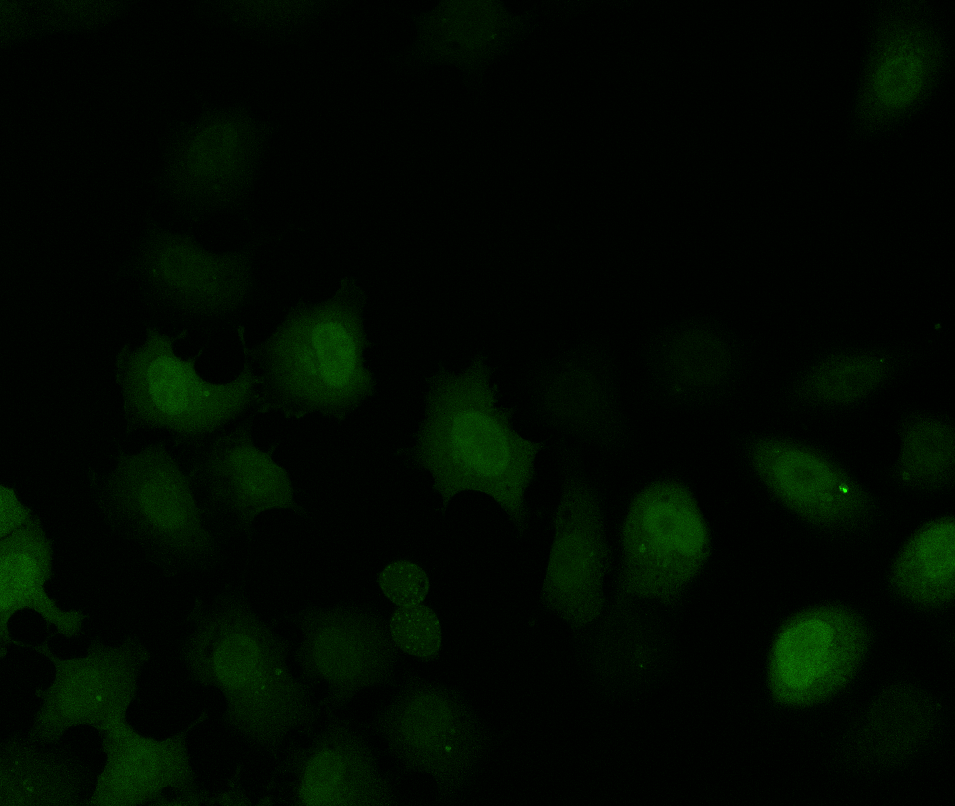

Supplement: Supplementary file 6 — Source Data for Figure 2 [file EMBR-24-e57300-s001.zip › Fig 2/2A/FIP200 KO_siSTK38 #3_-1h.tif]

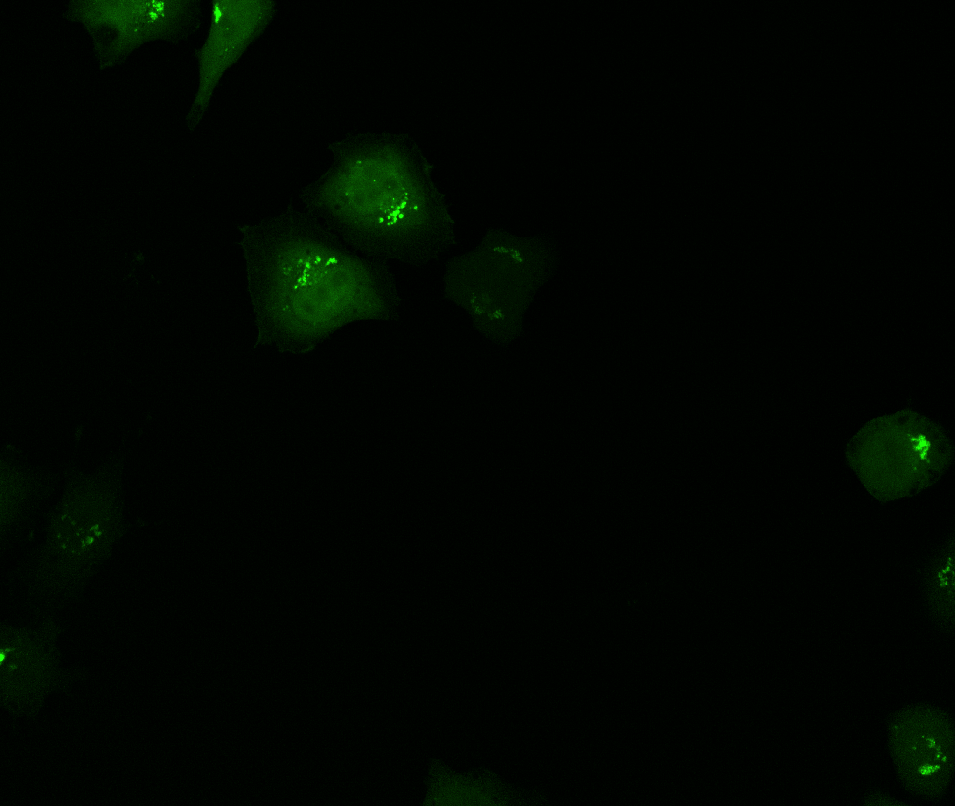

Supplement: Supplementary file 6 — Source Data for Figure 2 [file EMBR-24-e57300-s001.zip › Fig 2/2A/FIP200 KO_siSTK38 #3_10h.tif]

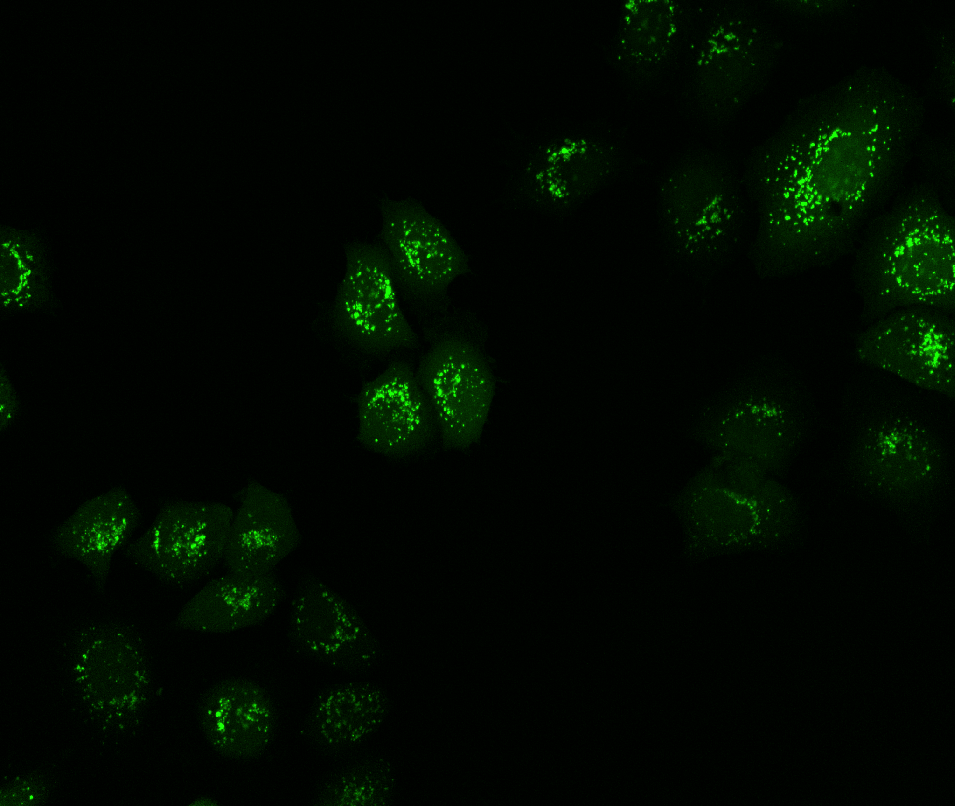

Supplement: Supplementary file 6 — Source Data for Figure 2 [file EMBR-24-e57300-s001.zip › Fig 2/2A/FIP200 KO_siSTK38 #3_0h.tif]

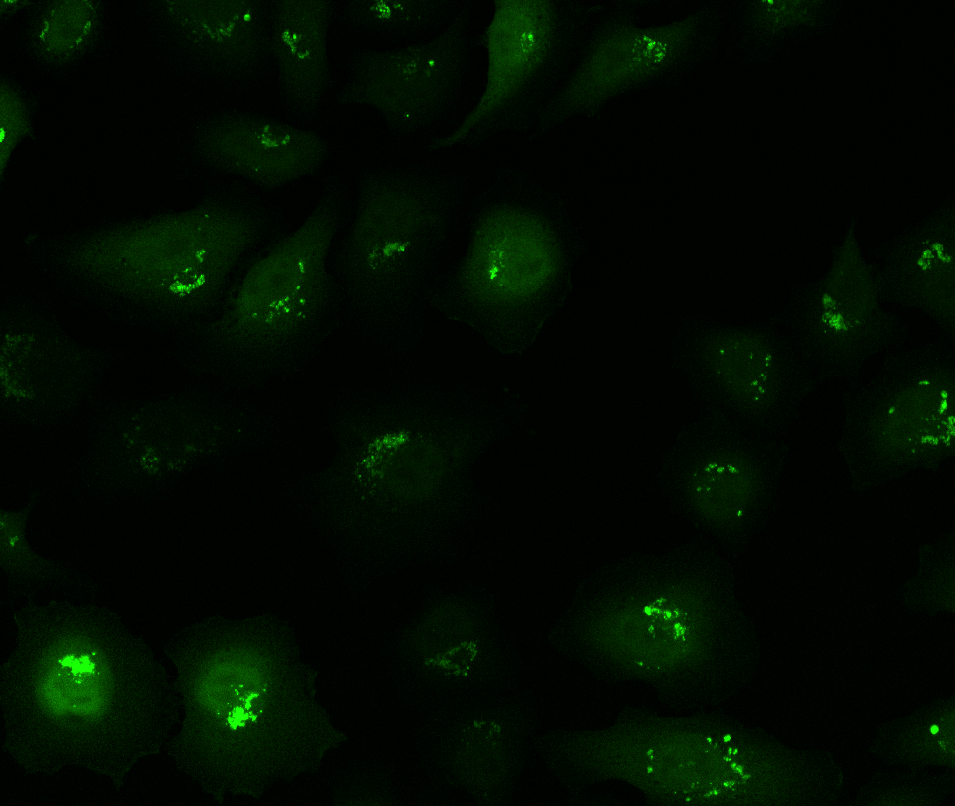

Supplement: Supplementary file 6 — Source Data for Figure 2 [file EMBR-24-e57300-s001.zip › Fig 2/2A/FIP200 KO_siLuc_10h.tif]

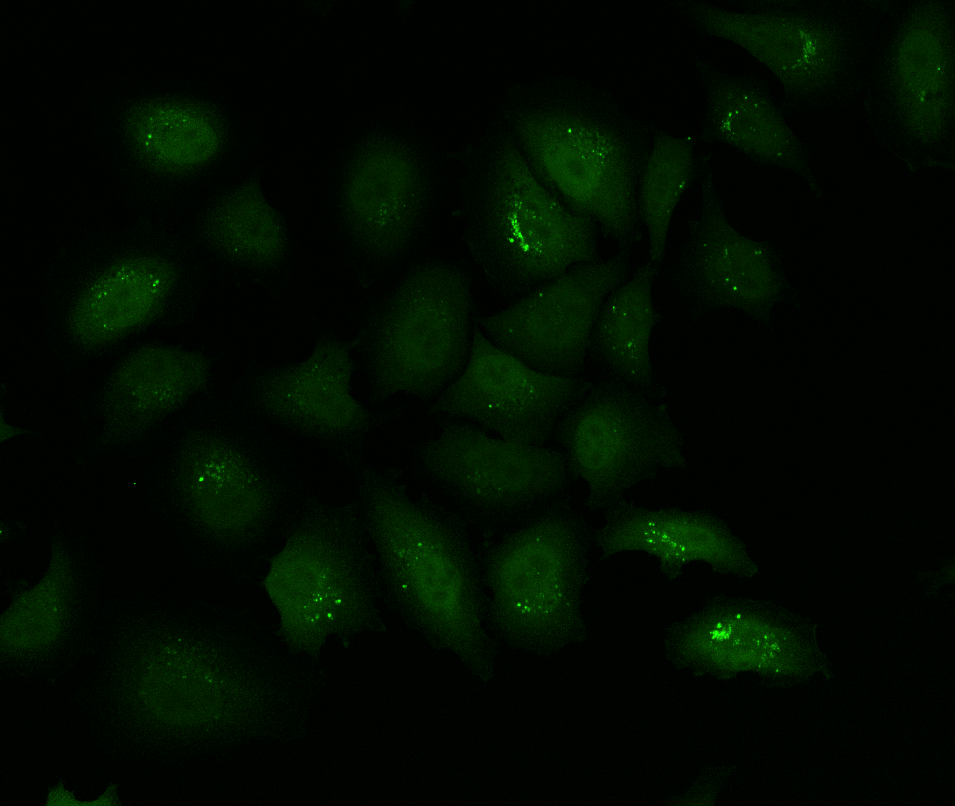

Supplement: Supplementary file 6 — Source Data for Figure 2 [file EMBR-24-e57300-s001.zip › Fig 2/2A/WT_siLuc_10h.tif]

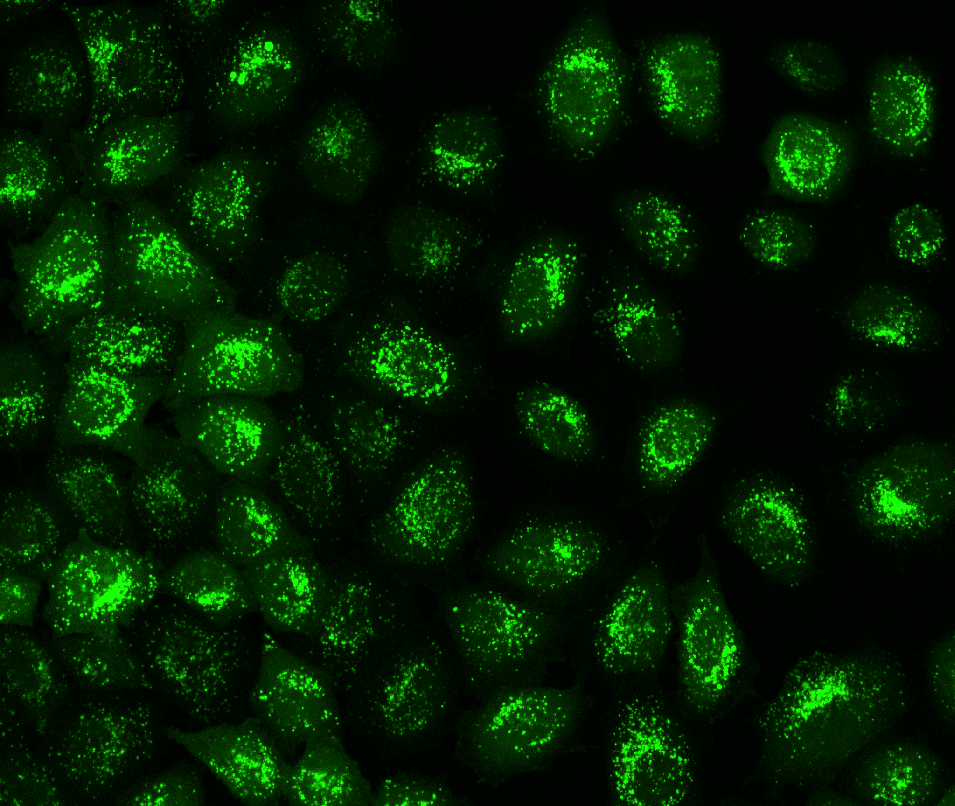

Supplement: Supplementary file 6 — Source Data for Figure 2 [file EMBR-24-e57300-s001.zip › Fig 2/2A/WT_siLuc_0h.tif]

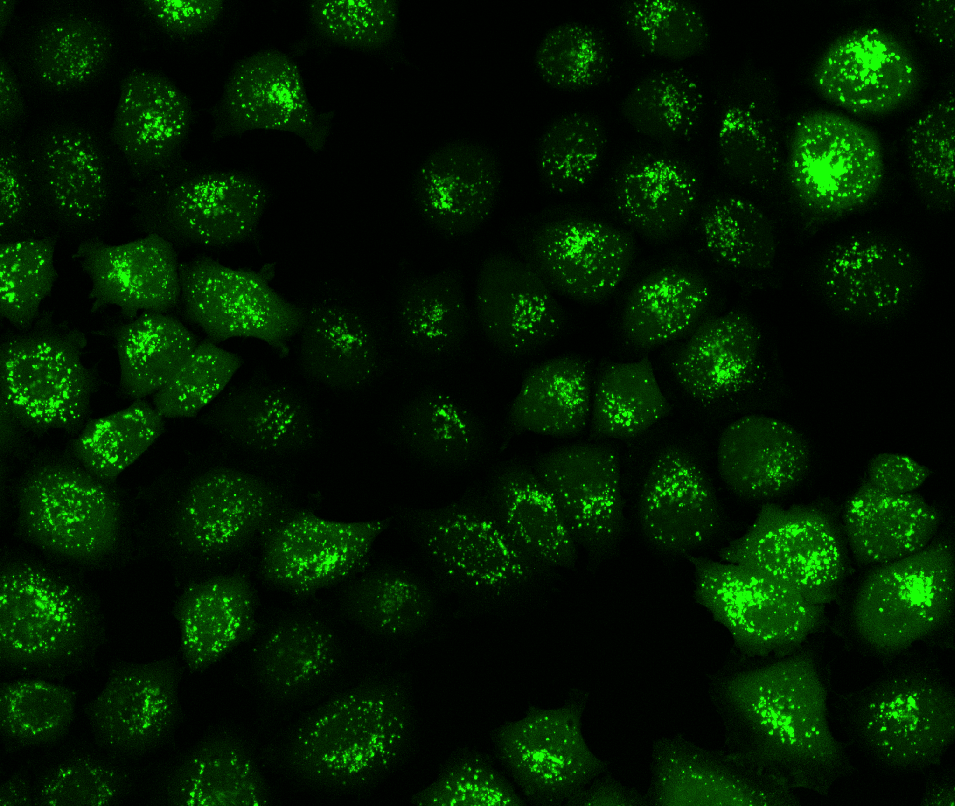

Supplement: Supplementary file 6 — Source Data for Figure 2 [file EMBR-24-e57300-s001.zip › Fig 2/2A/WT_siSTK38 #3_0h.tif]

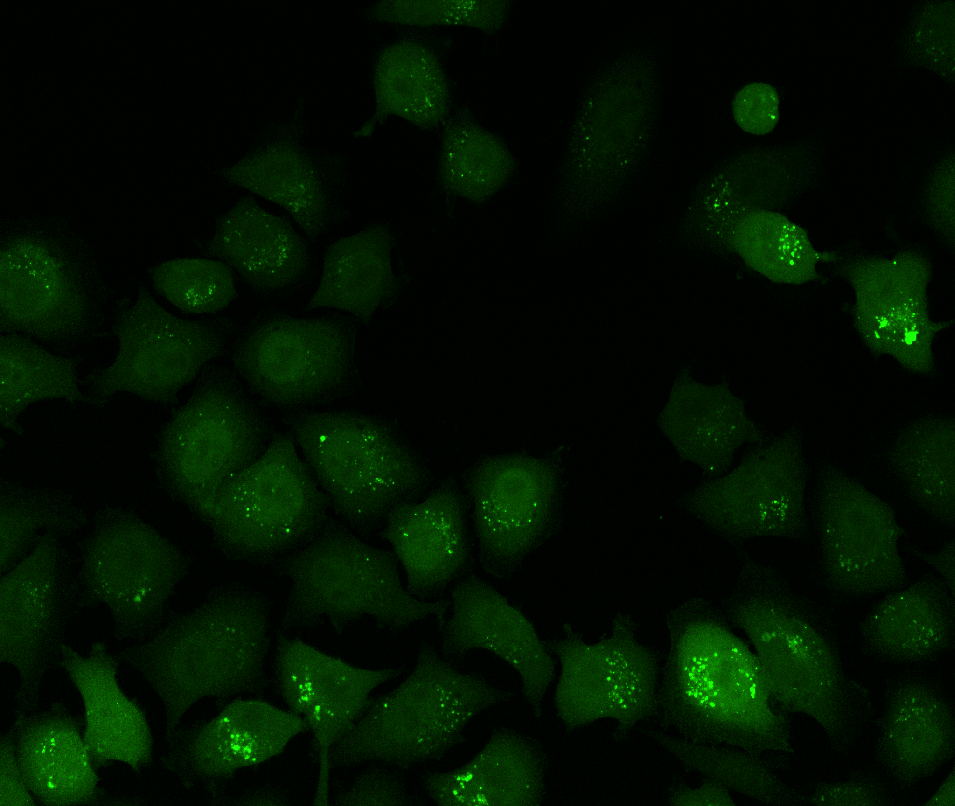

Supplement: Supplementary file 6 — Source Data for Figure 2 [file EMBR-24-e57300-s001.zip › Fig 2/2A/WT_siSTK38 #3_10h.tif]

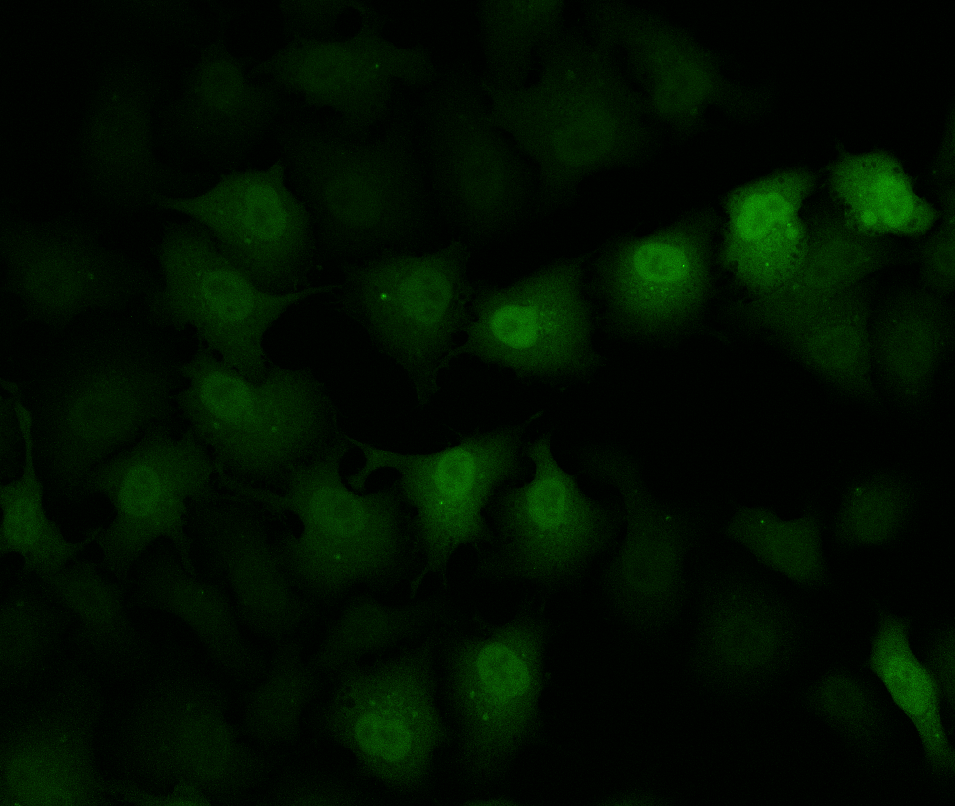

Supplement: Supplementary file 6 — Source Data for Figure 2 [file EMBR-24-e57300-s001.zip › Fig 2/2A/FIP200 KO_siLuc_-1h.tif]

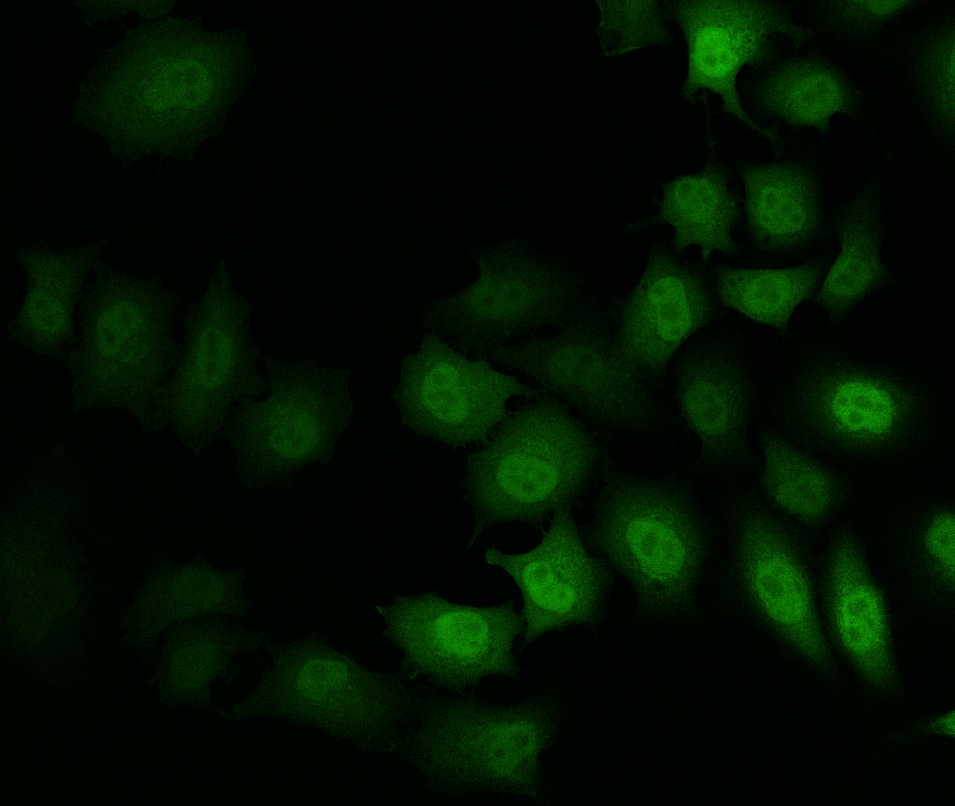

Supplement: Supplementary file 6 — Source Data for Figure 2 [file EMBR-24-e57300-s001.zip › Fig 2/2A/WT_siLuc_-1h.tif]

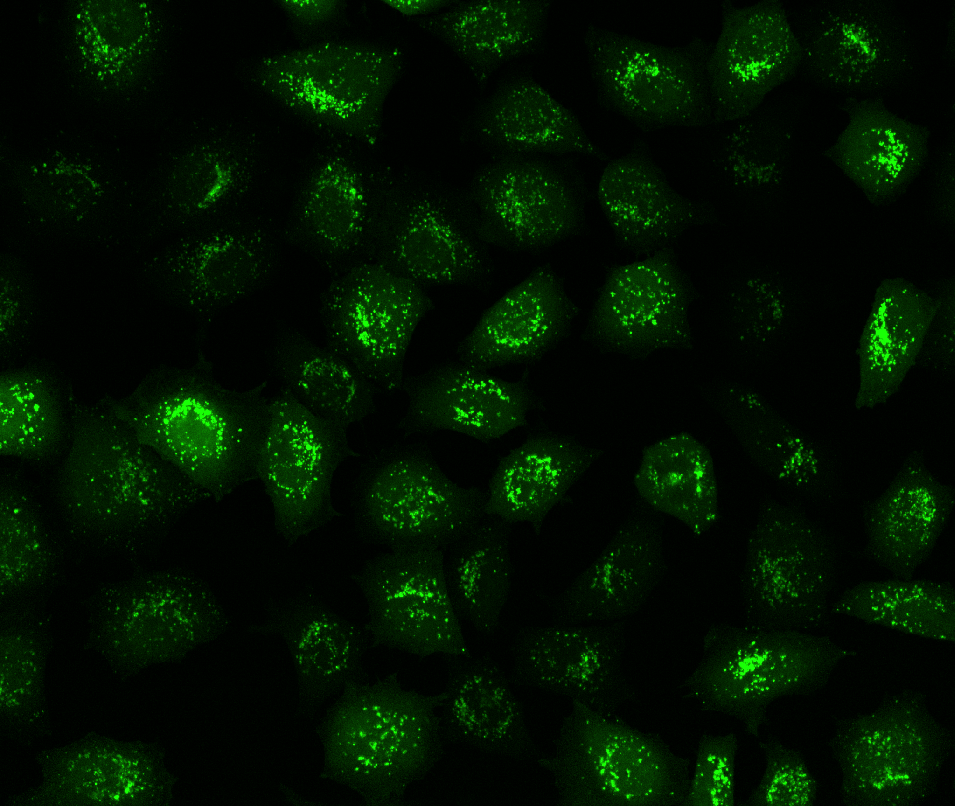

Supplement: Supplementary file 6 — Source Data for Figure 2 [file EMBR-24-e57300-s001.zip › Fig 2/2A/FIP200 KO_siLuc_0h.tif]

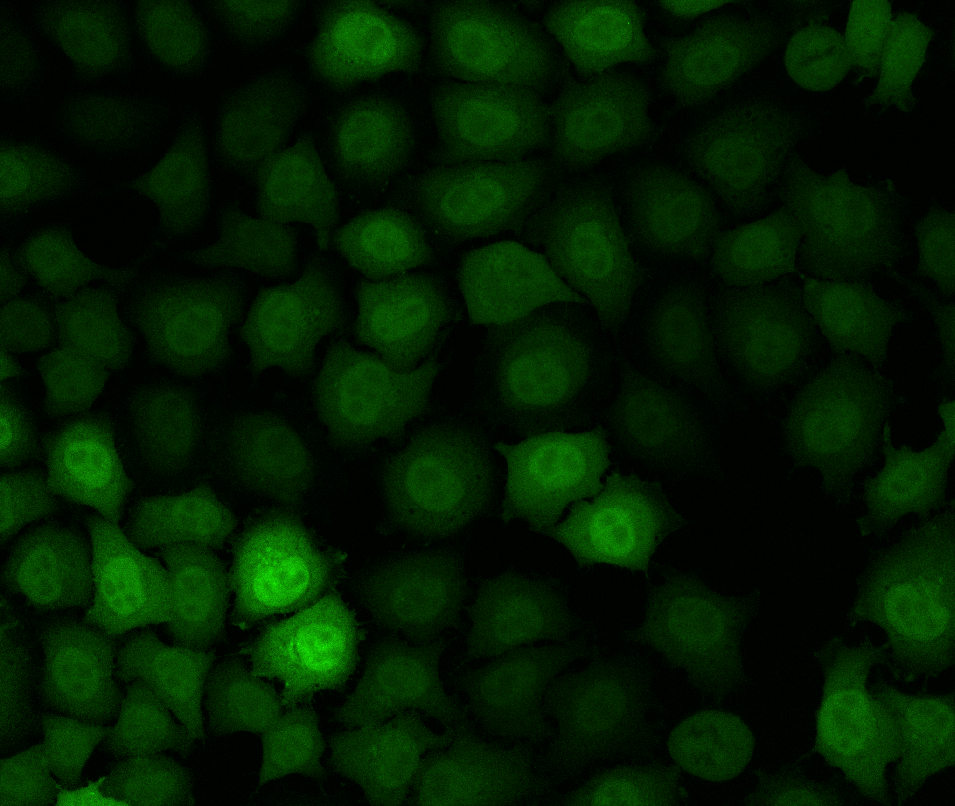

Supplement: Supplementary file 6 — Source Data for Figure 2 [file EMBR-24-e57300-s001.zip › Fig 2/2A/WT_siSTK38 #3_-1h.tif]

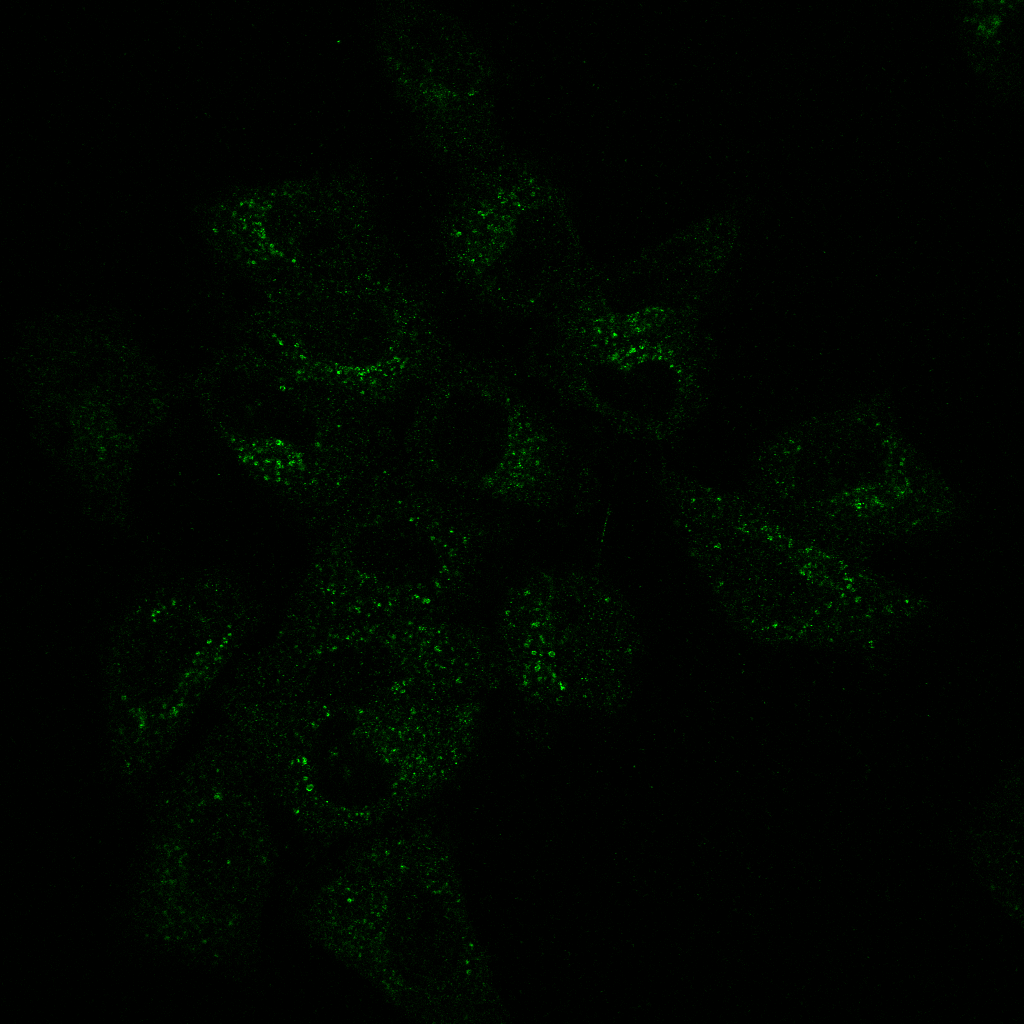

Supplement: Supplementary file 6 — Source Data for Figure 2 [file EMBR-24-e57300-s001.zip › Fig 2/2F/siLuc_LLOMe 1h_VPS4.tif]

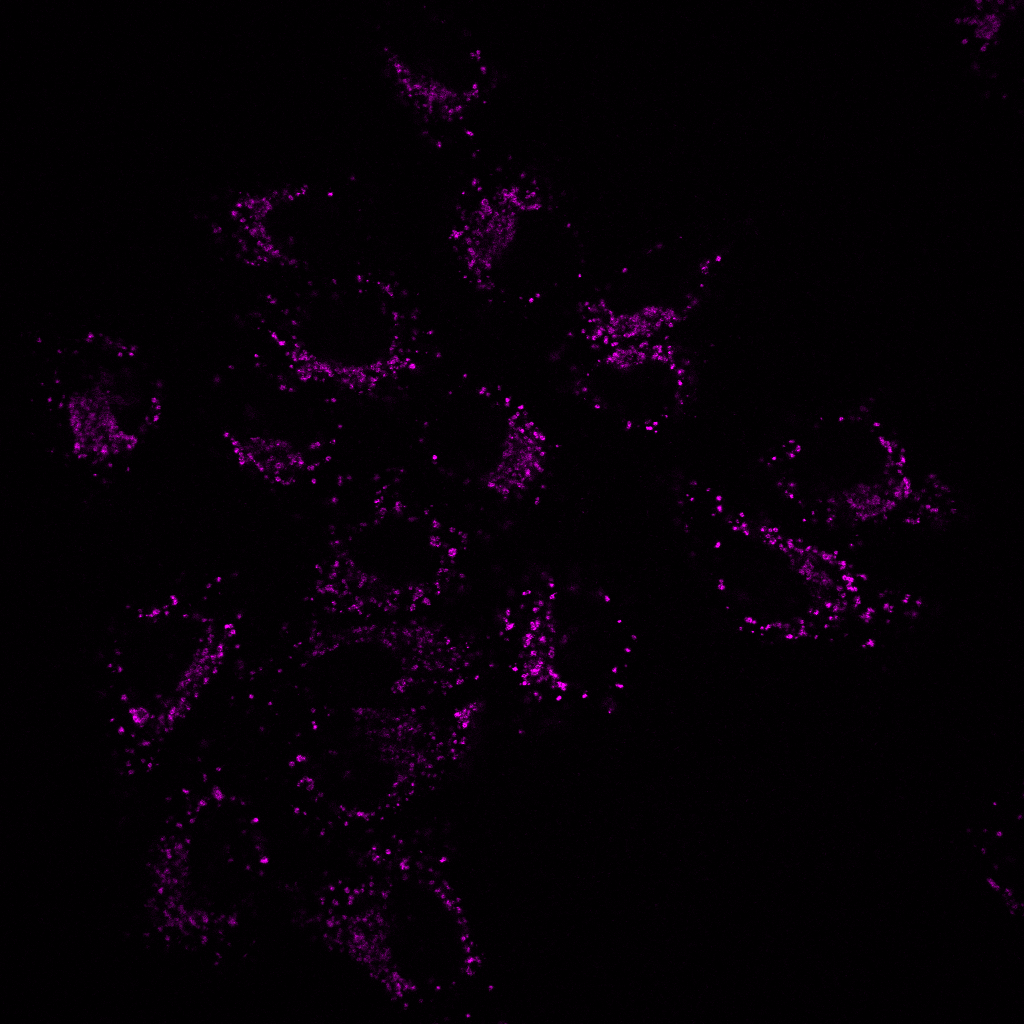

Supplement: Supplementary file 6 — Source Data for Figure 2 [file EMBR-24-e57300-s001.zip › Fig 2/2F/siLuc_LLOMe 1h_LAMP1.tif]

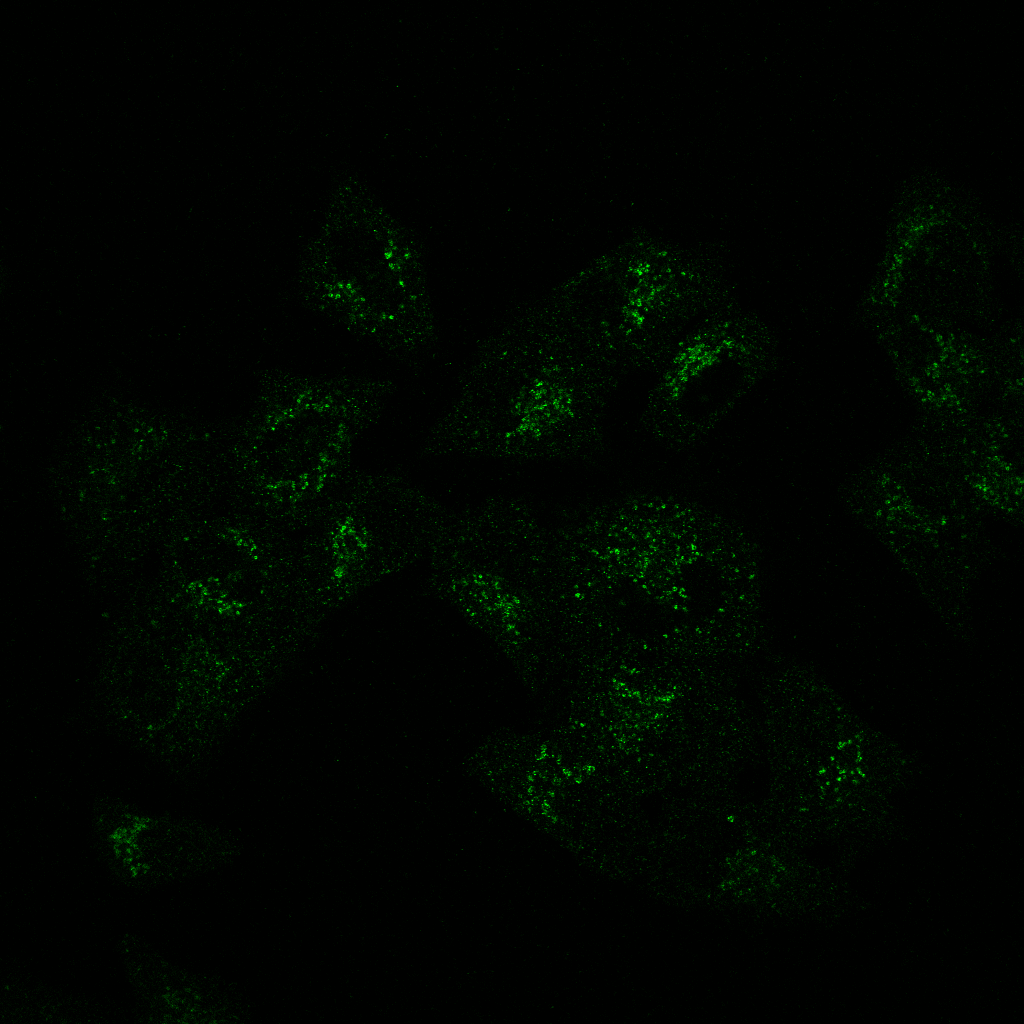

Supplement: Supplementary file 6 — Source Data for Figure 2 [file EMBR-24-e57300-s001.zip › Fig 2/2F/siLuc_LLOMe 30min_VPS4.tif]

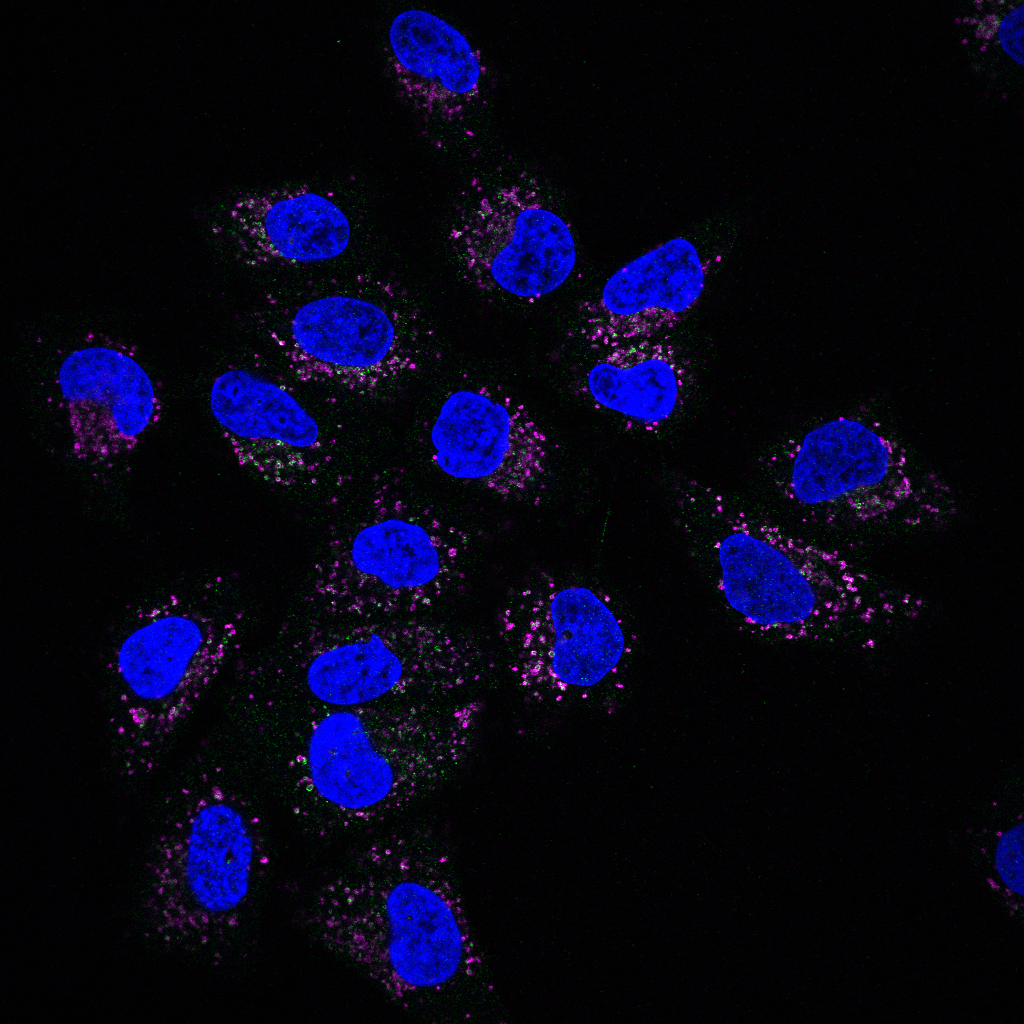

Supplement: Supplementary file 6 — Source Data for Figure 2 [file EMBR-24-e57300-s001.zip › Fig 2/2F/siLuc_LLOMe 1h_Merge+DAPI.tif]

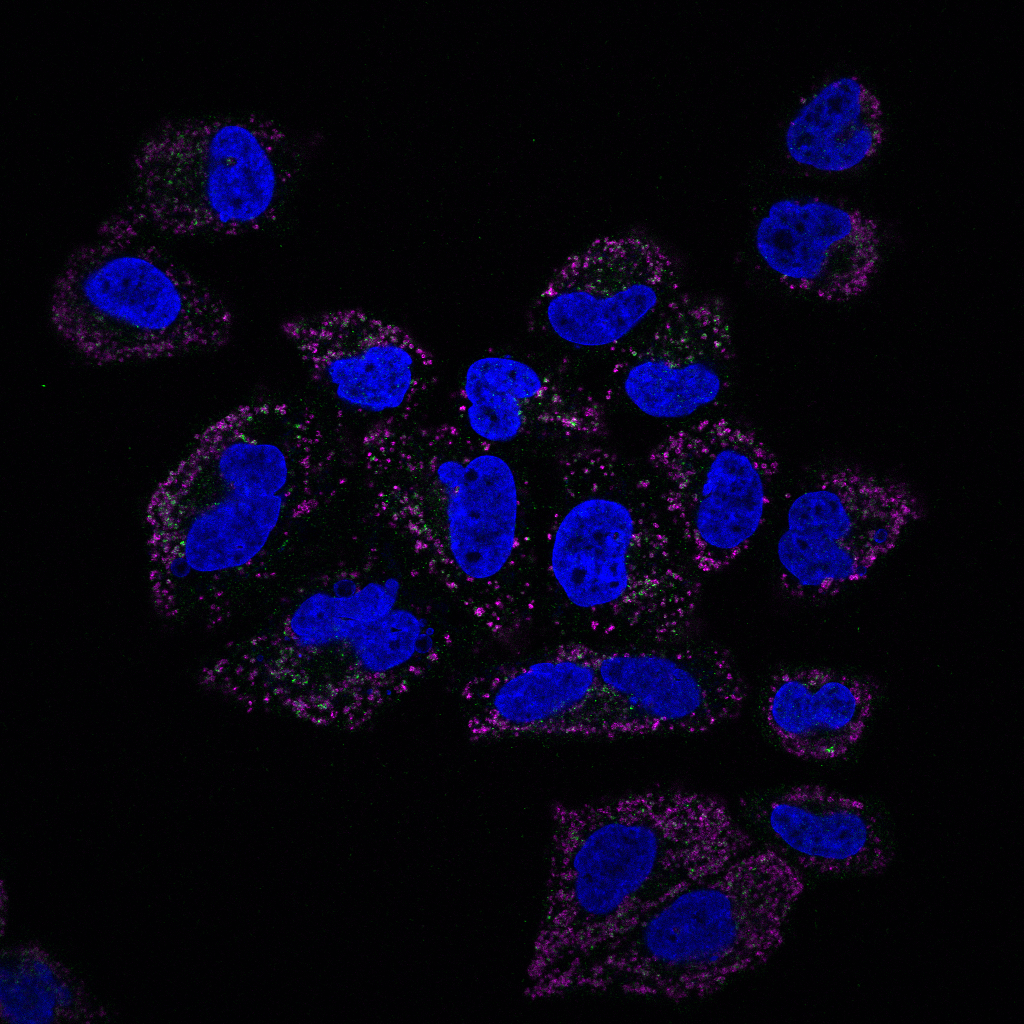

Supplement: Supplementary file 6 — Source Data for Figure 2 [file EMBR-24-e57300-s001.zip › Fig 2/2F/siSTK38 #1_LLOMe 1h_Merge+DAPI.tif]

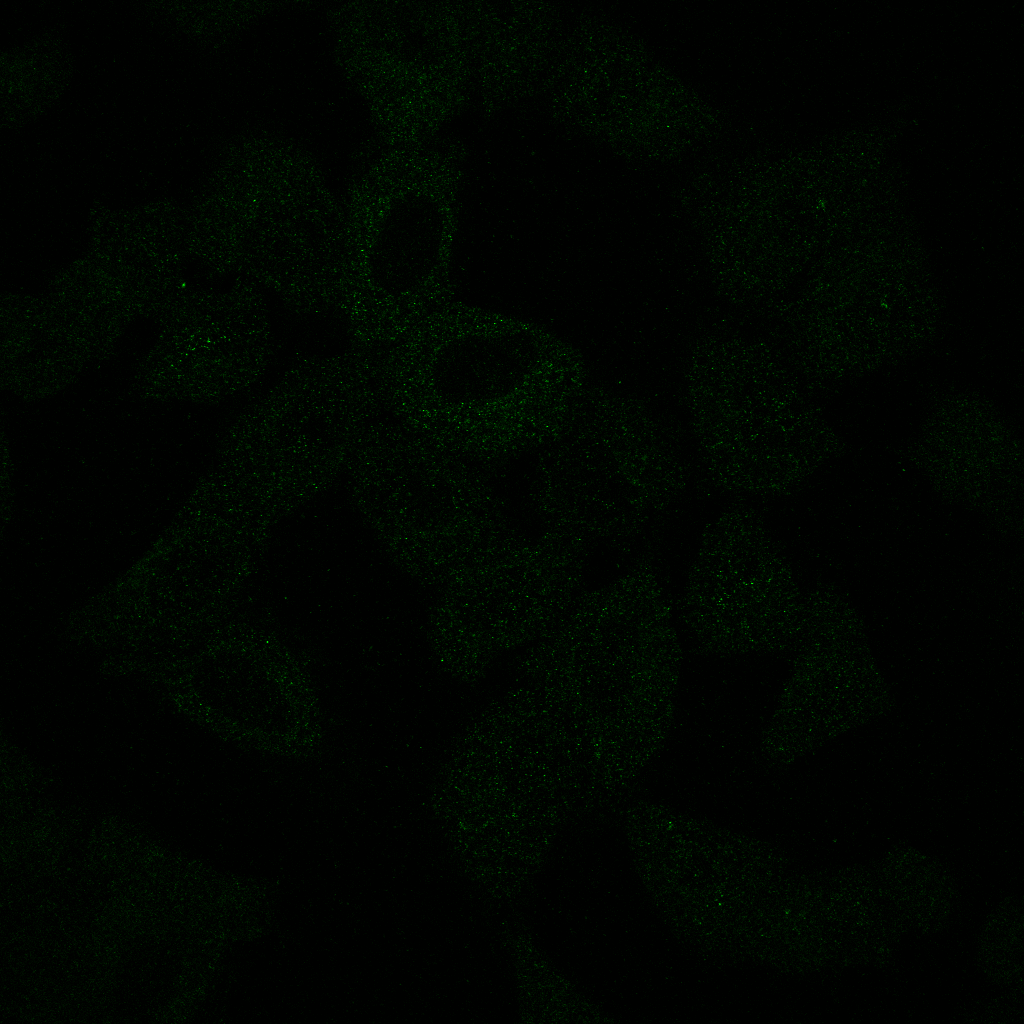

Supplement: Supplementary file 6 — Source Data for Figure 2 [file EMBR-24-e57300-s001.zip › Fig 2/2F/siLuc_non-treated_VPS4.tif]

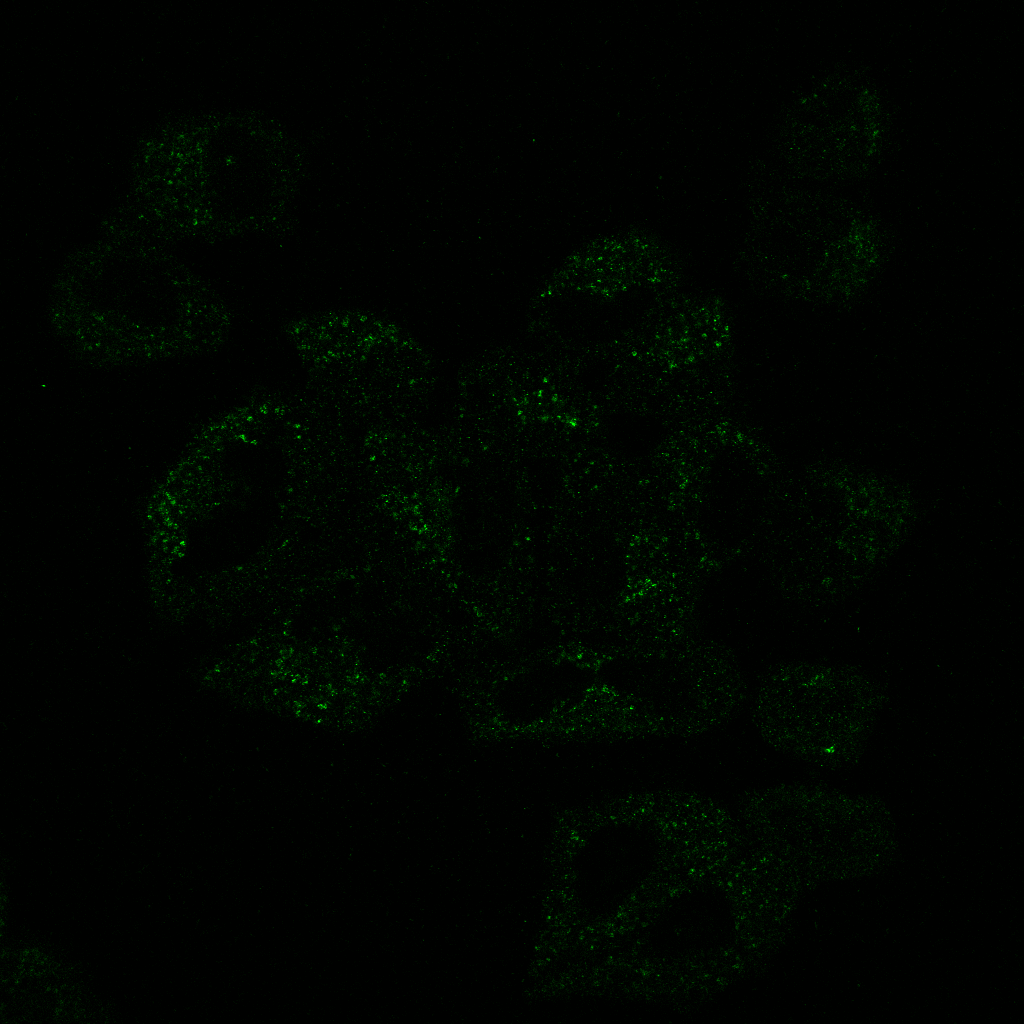

Supplement: Supplementary file 6 — Source Data for Figure 2 [file EMBR-24-e57300-s001.zip › Fig 2/2F/siSTK38 #1_LLOMe 1h_VPS4.tif]

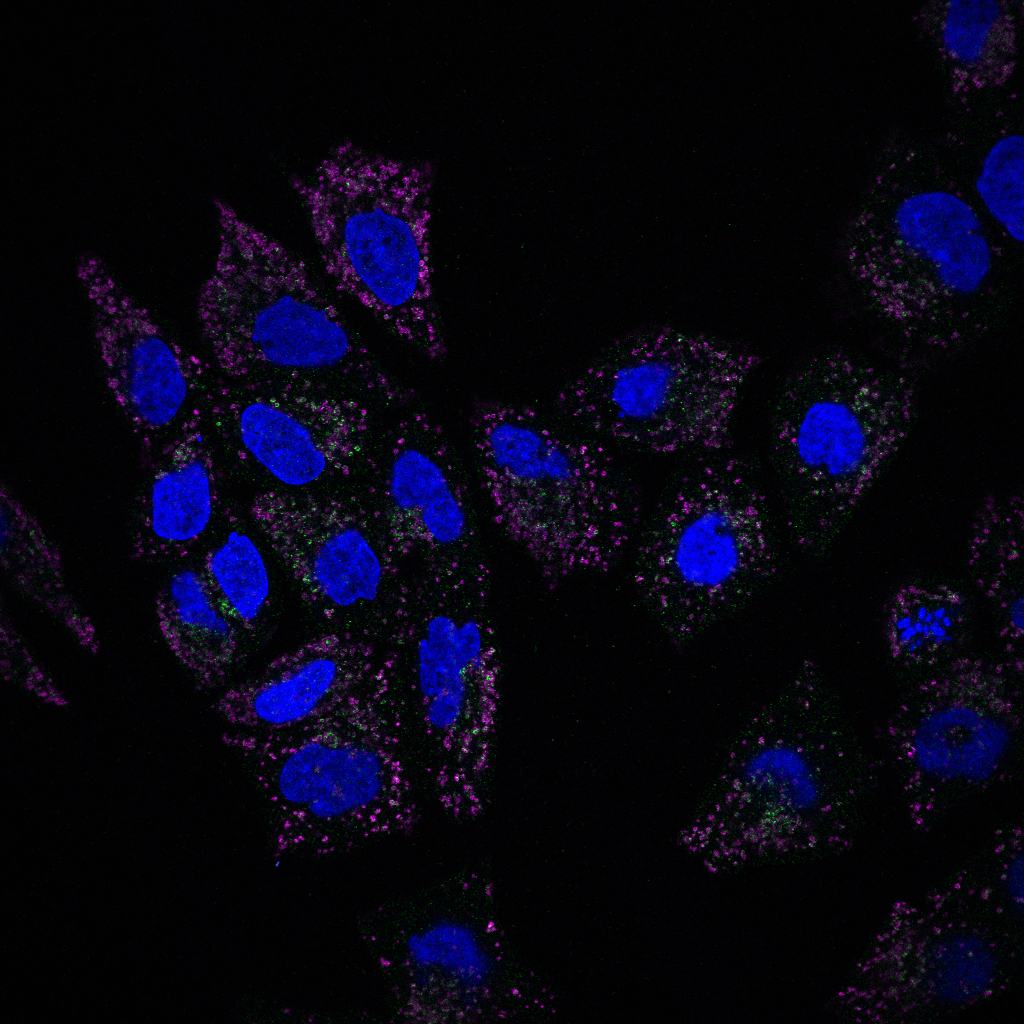

Supplement: Supplementary file 6 — Source Data for Figure 2 [file EMBR-24-e57300-s001.zip › Fig 2/2F/siSTK38 #1_LLOMe 30min_Merge+DAPI.tif]

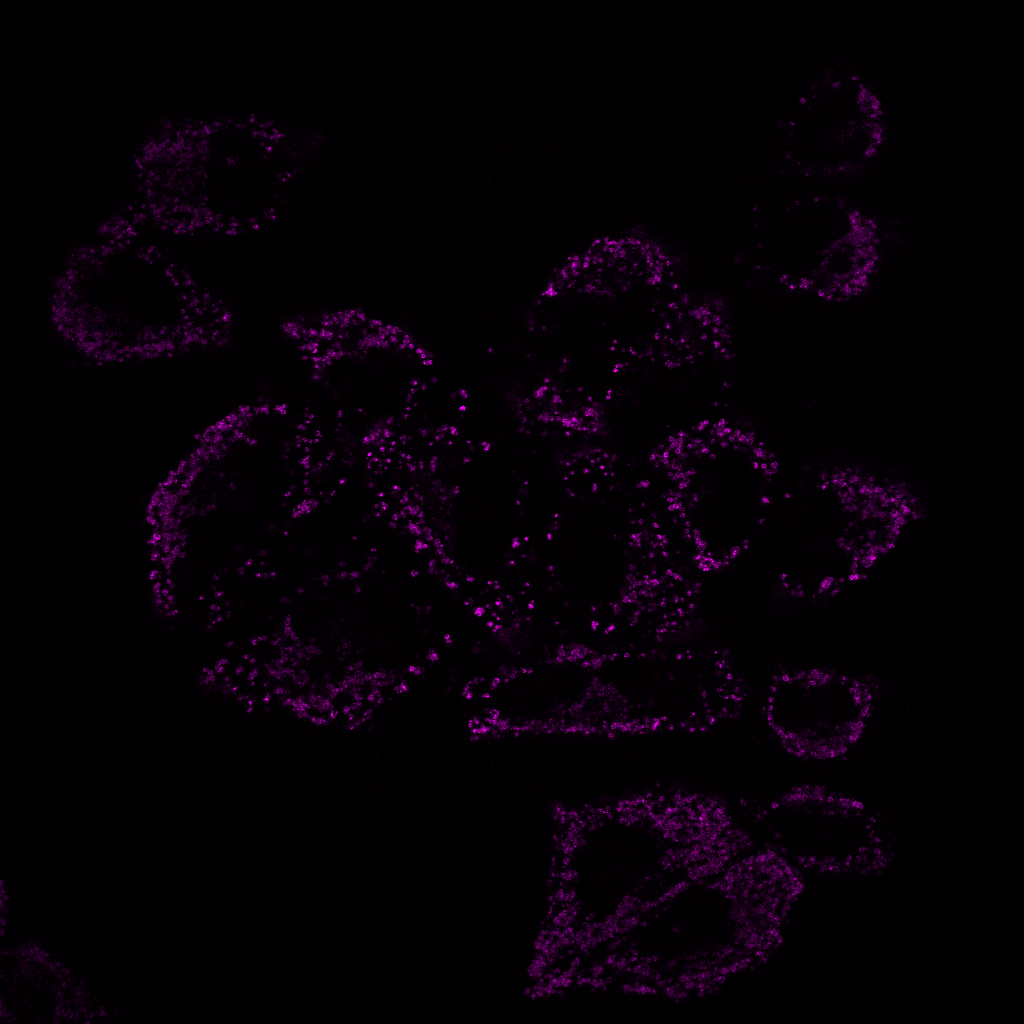

Supplement: Supplementary file 6 — Source Data for Figure 2 [file EMBR-24-e57300-s001.zip › Fig 2/2F/siSTK38 #1_LLOMe 1h_LAMP1.tif]

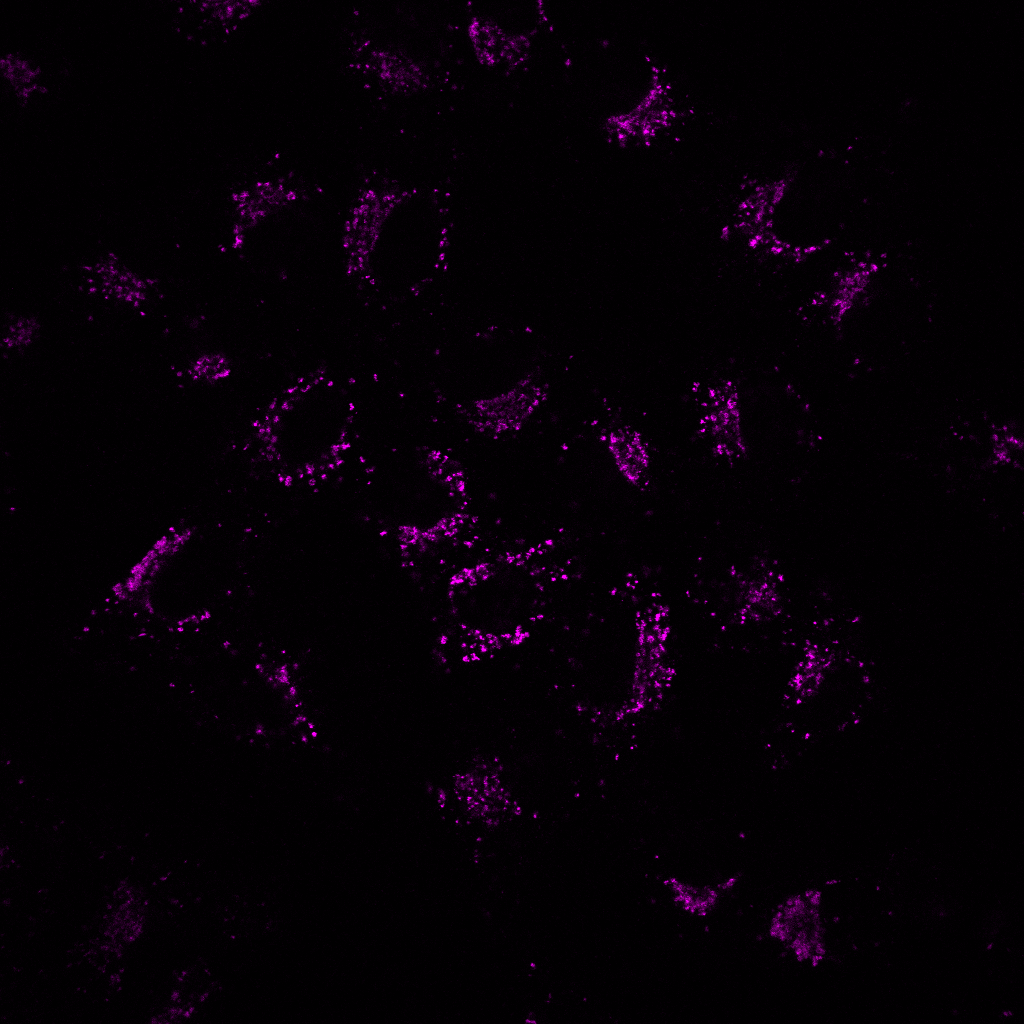

Supplement: Supplementary file 6 — Source Data for Figure 2 [file EMBR-24-e57300-s001.zip › Fig 2/2F/siLuc_non-treated_LAMP1.tif]

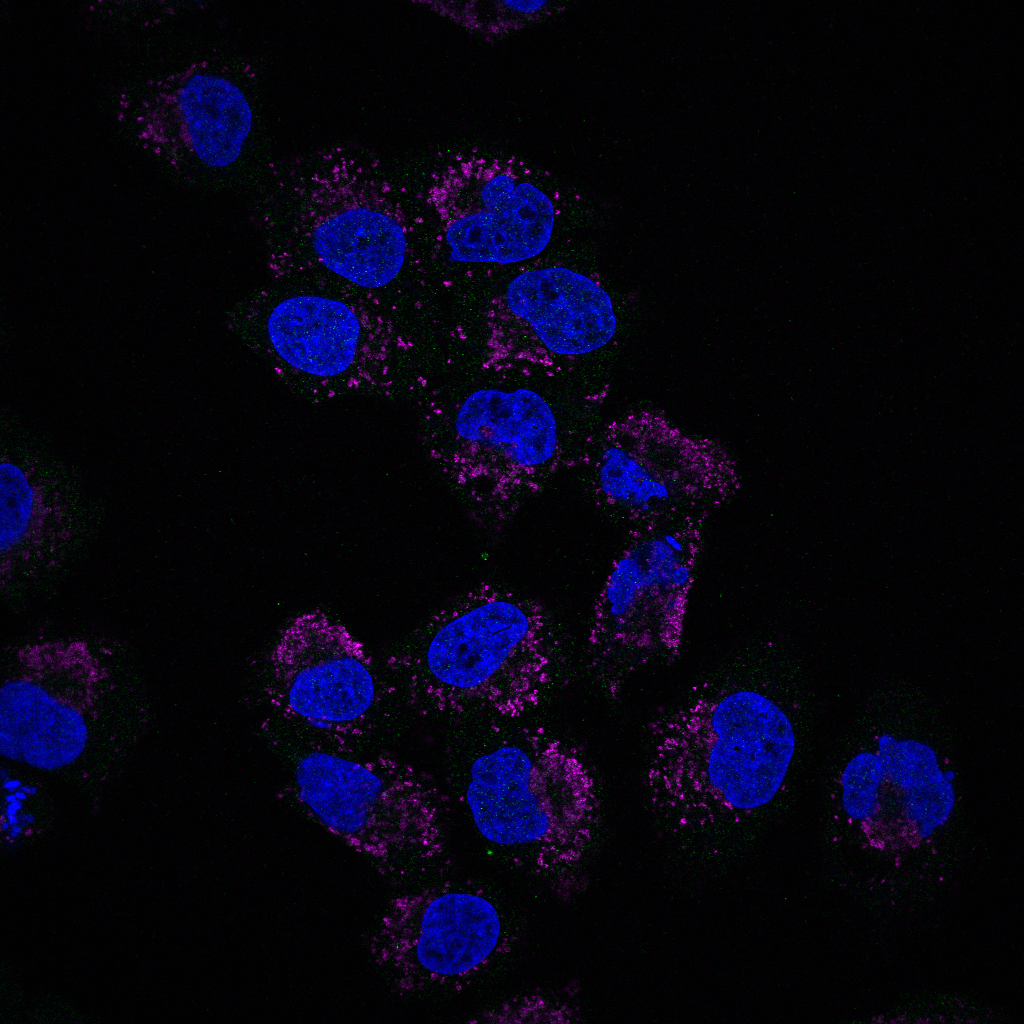

Supplement: Supplementary file 6 — Source Data for Figure 2 [file EMBR-24-e57300-s001.zip › Fig 2/2F/siSTK38 #1_non-treated_Merge+DAPI.tif]

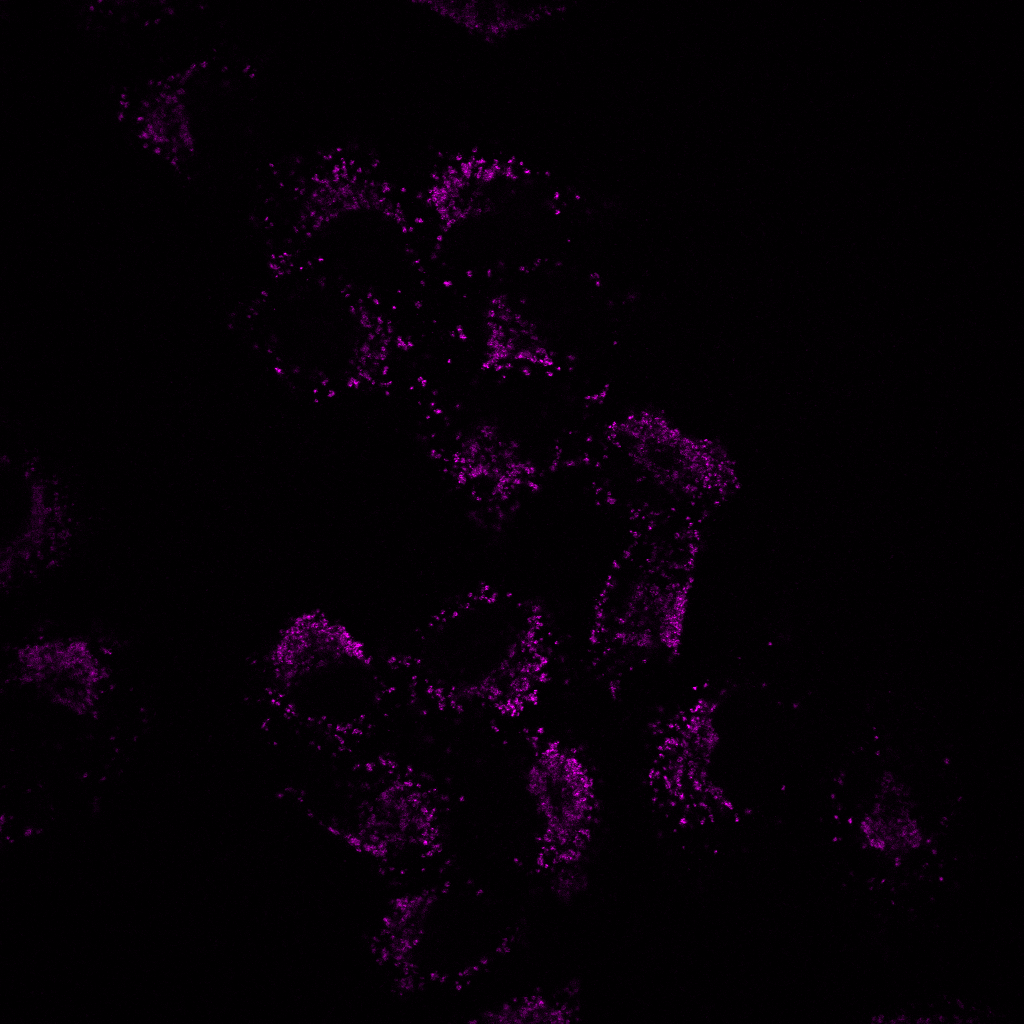

Supplement: Supplementary file 6 — Source Data for Figure 2 [file EMBR-24-e57300-s001.zip › Fig 2/2F/siSTK38 #1_non-treated_LAMP1.tif]

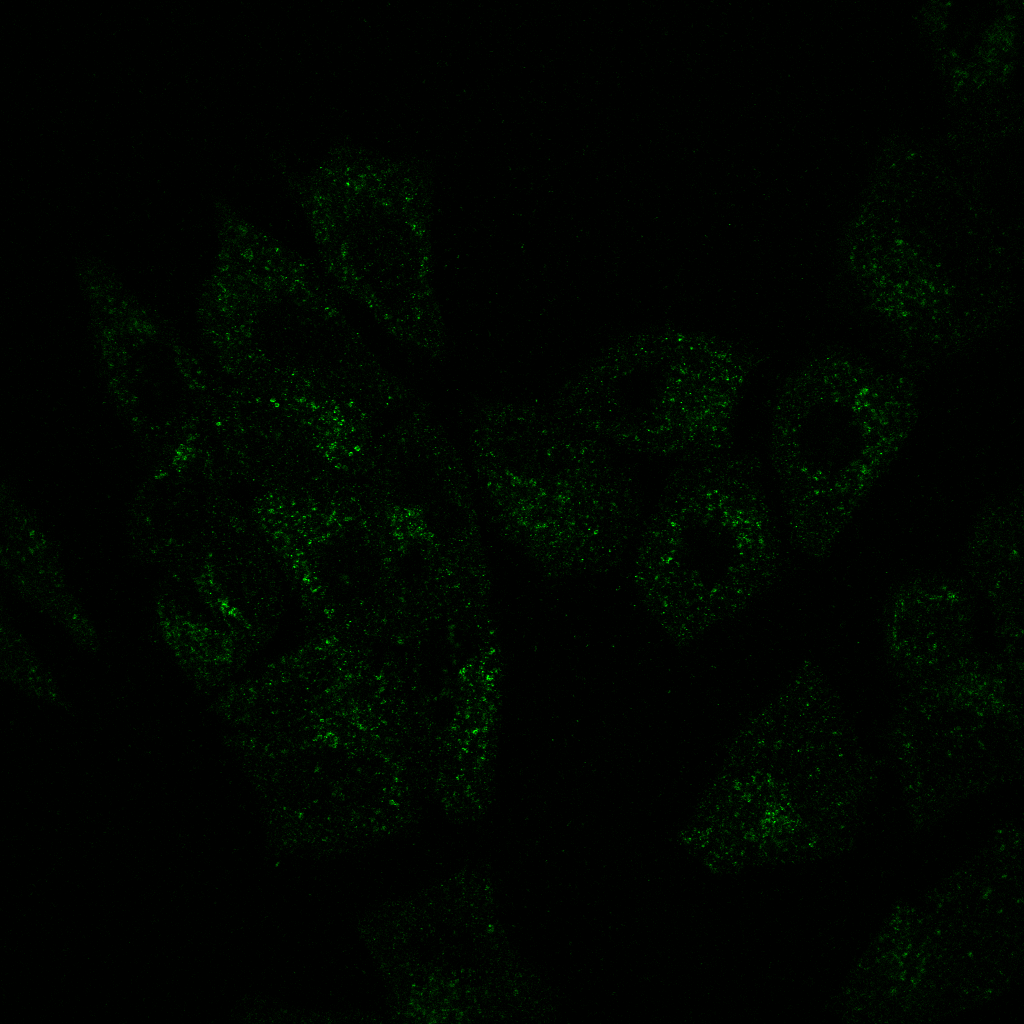

Supplement: Supplementary file 6 — Source Data for Figure 2 [file EMBR-24-e57300-s001.zip › Fig 2/2F/siSTK38 #1_LLOMe 30min_VPS4.tif]

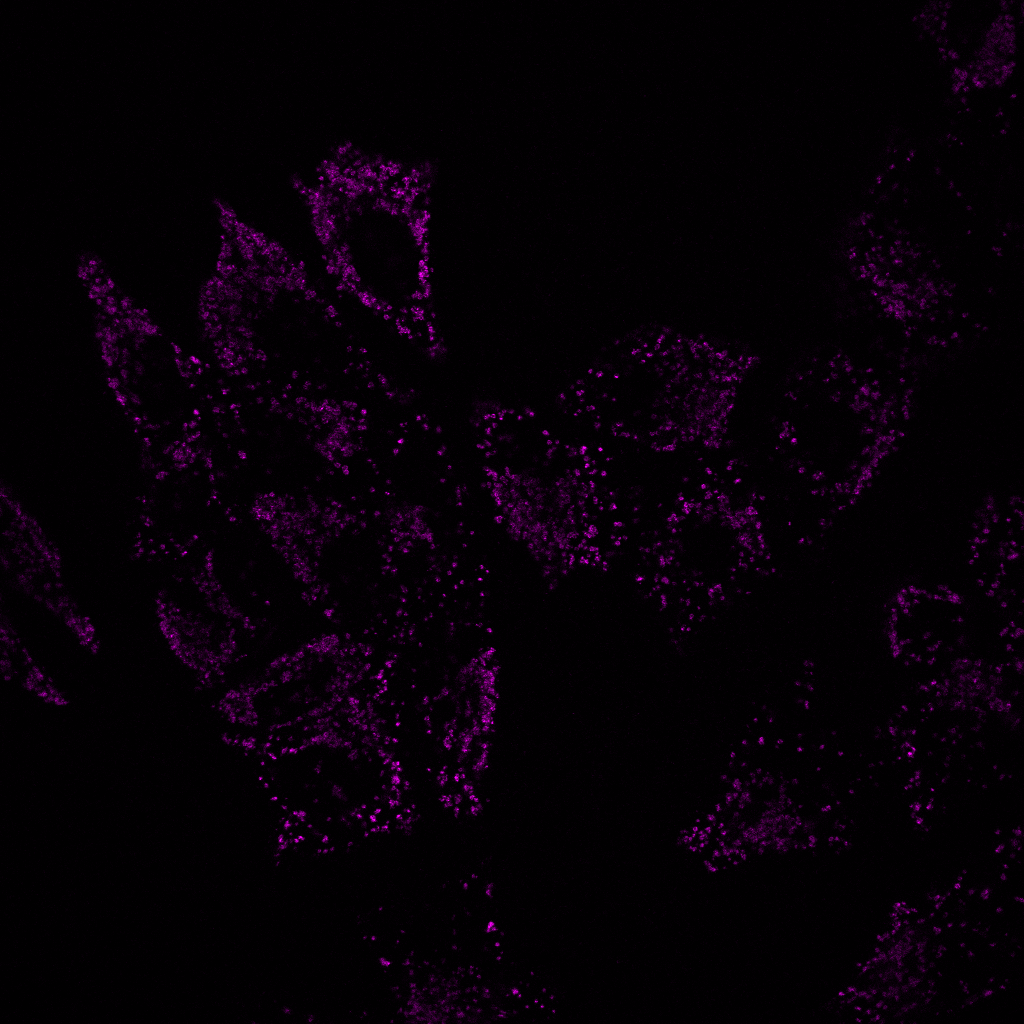

Supplement: Supplementary file 6 — Source Data for Figure 2 [file EMBR-24-e57300-s001.zip › Fig 2/2F/siSTK38 #1_LLOMe 30min_LAMP1.tif]

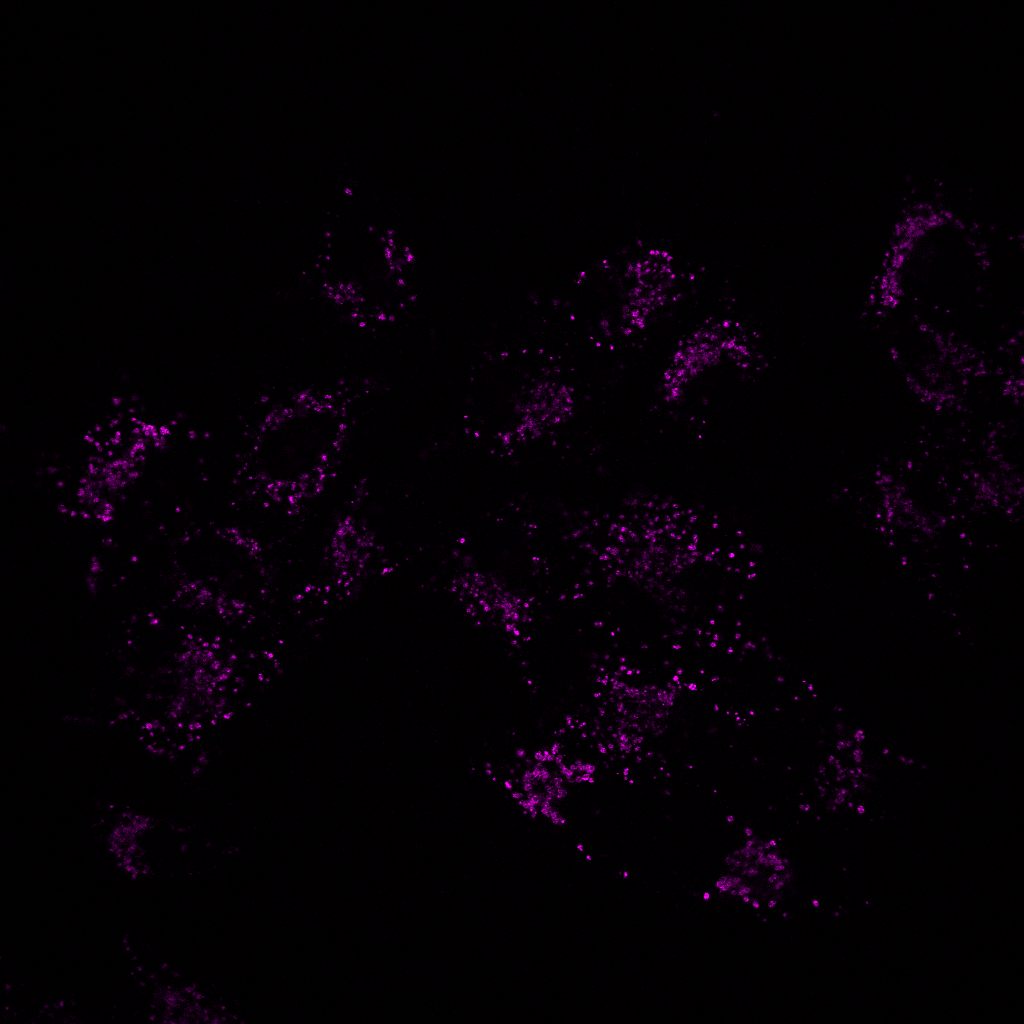

Supplement: Supplementary file 6 — Source Data for Figure 2 [file EMBR-24-e57300-s001.zip › Fig 2/2F/siLuc_LLOMe 30min_LAMP1.tif]

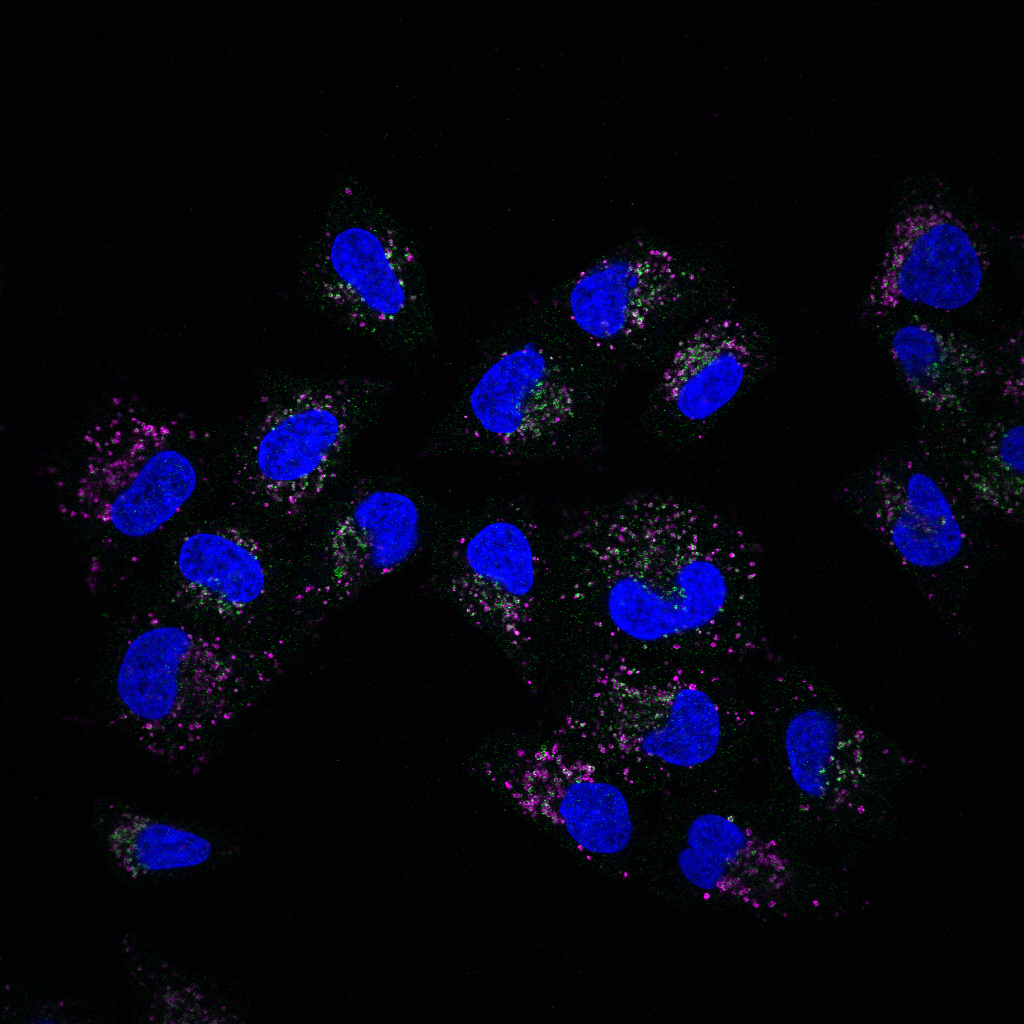

Supplement: Supplementary file 6 — Source Data for Figure 2 [file EMBR-24-e57300-s001.zip › Fig 2/2F/siLuc_LLOMe 30min_Merge+DAPI.tif]

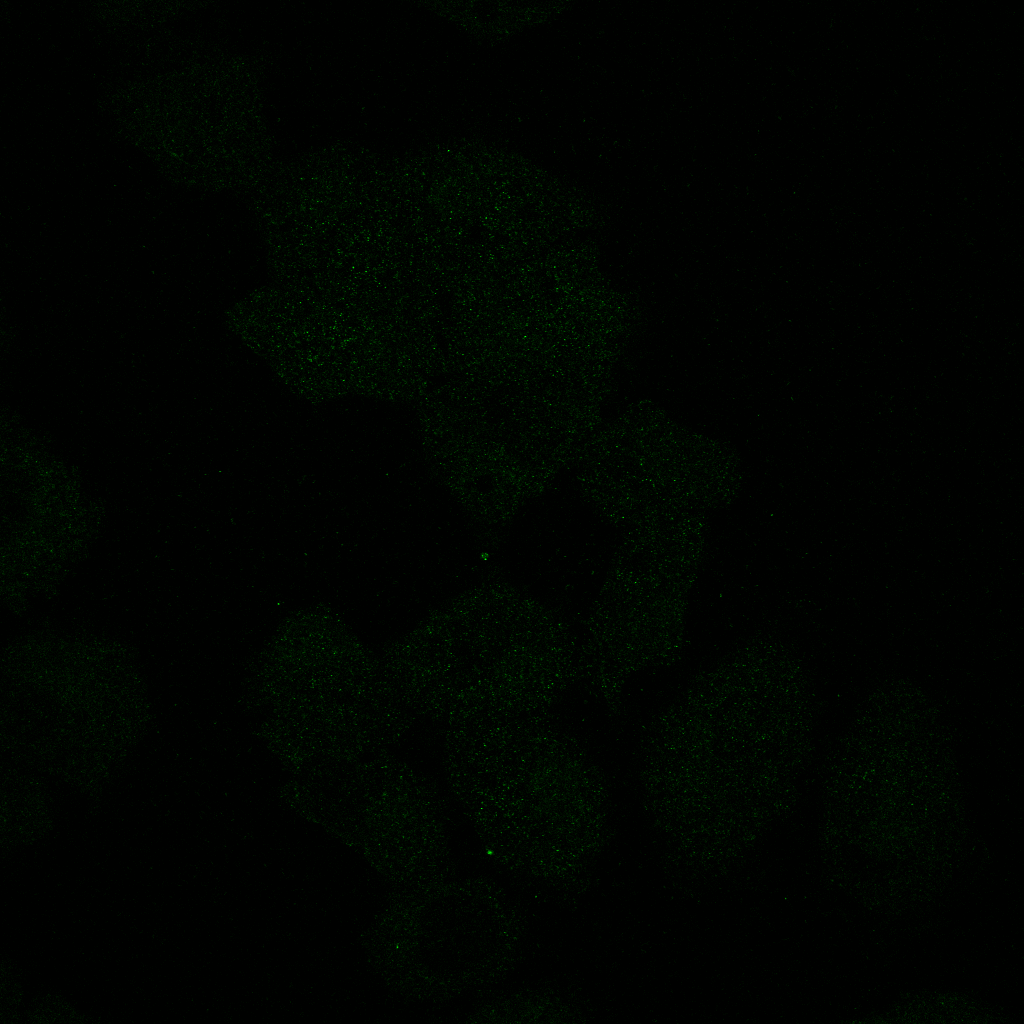

Supplement: Supplementary file 6 — Source Data for Figure 2 [file EMBR-24-e57300-s001.zip › Fig 2/2F/siSTK38 #1_non-treated_VPS4.tif]

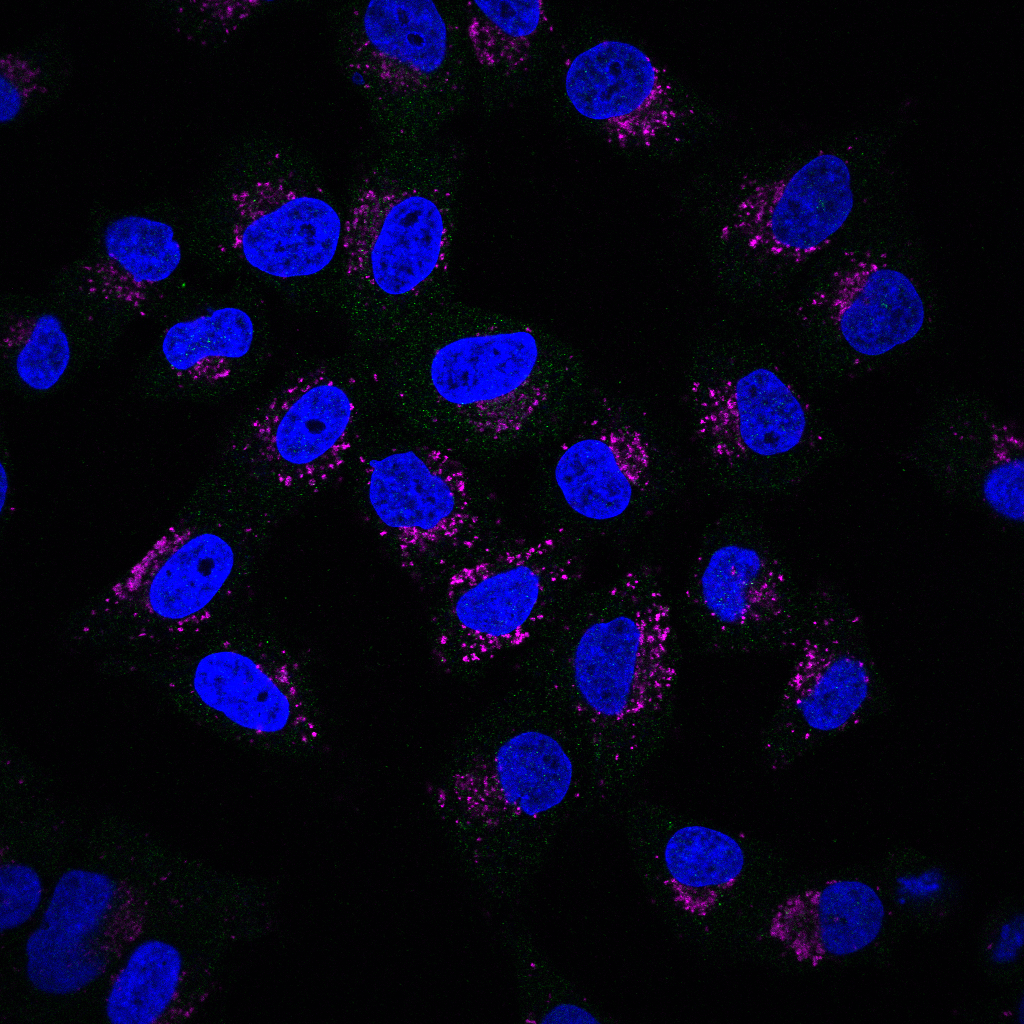

Supplement: Supplementary file 6 — Source Data for Figure 2 [file EMBR-24-e57300-s001.zip › Fig 2/2F/siLuc_non-treated_Merge+DAPI.tif]

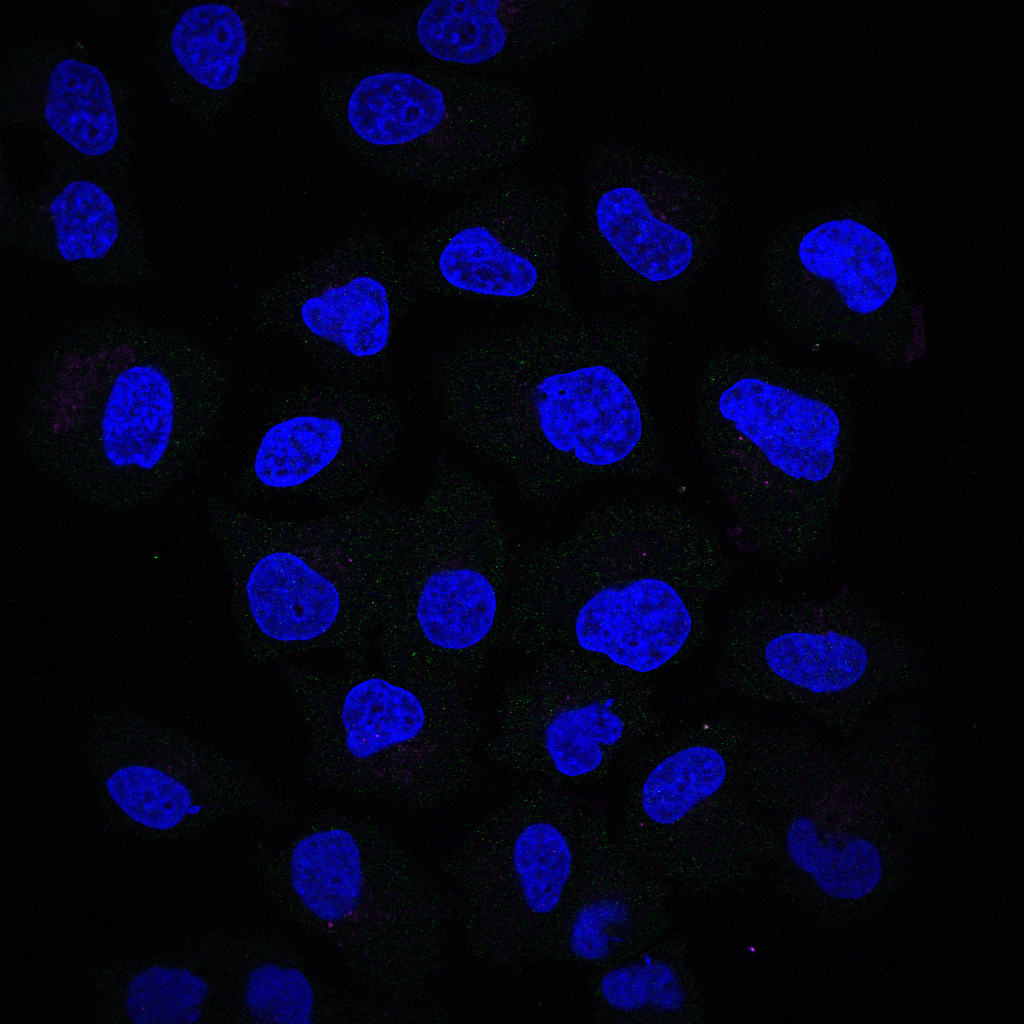

Supplement: Supplementary file 6 — Source Data for Figure 2 [file EMBR-24-e57300-s001.zip › Fig 2/2C/siLuc_non-treated.tif]

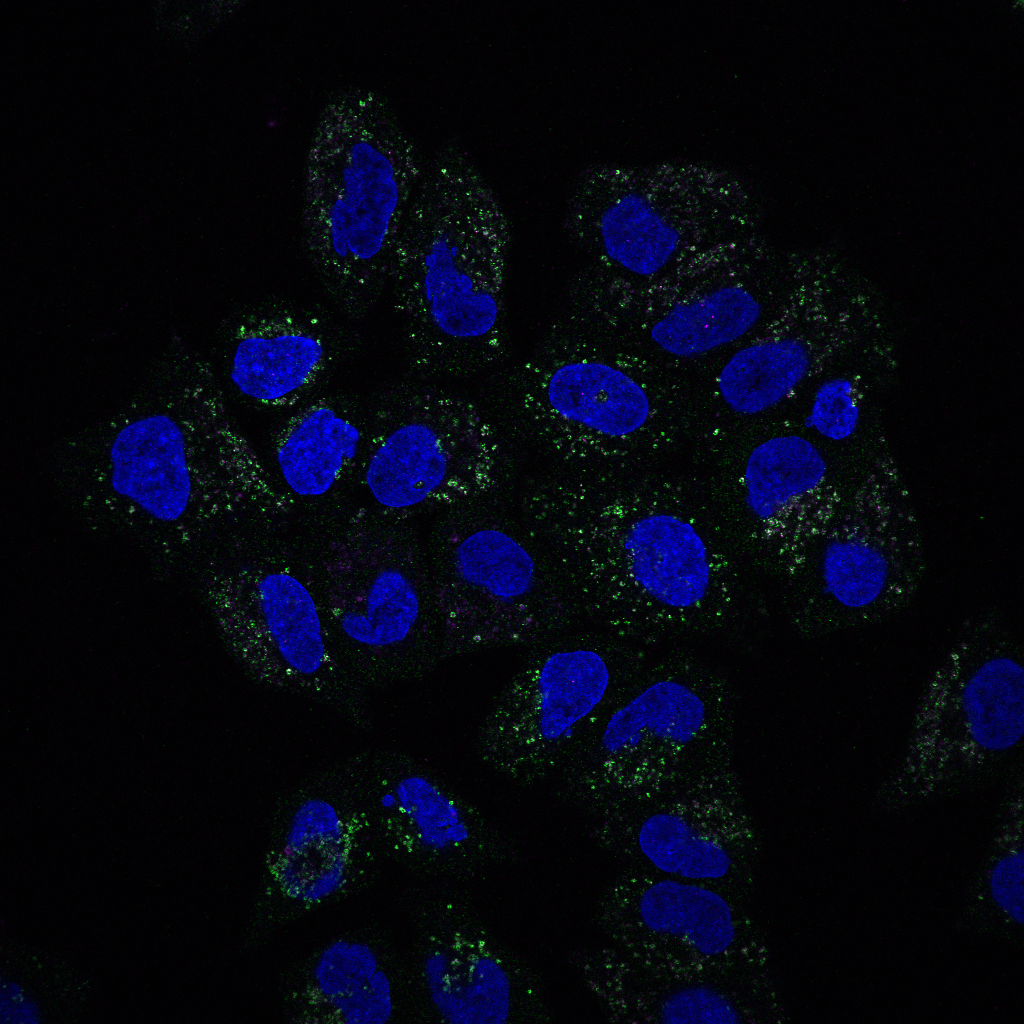

Supplement: Supplementary file 6 — Source Data for Figure 2 [file EMBR-24-e57300-s001.zip › Fig 2/2C/siSTK38 #1_LLOMe 30min.tif]

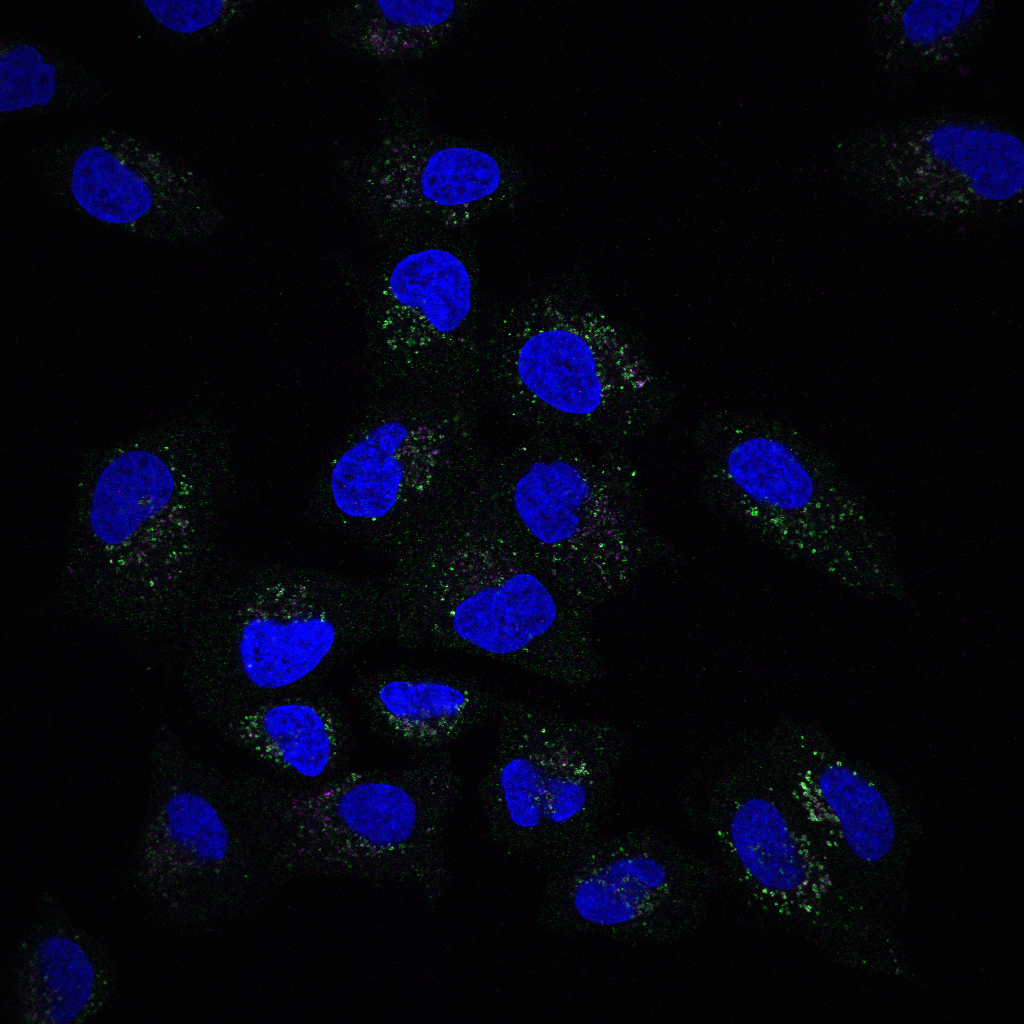

Supplement: Supplementary file 6 — Source Data for Figure 2 [file EMBR-24-e57300-s001.zip › Fig 2/2C/siLuc_LLOMe 30min.tif]

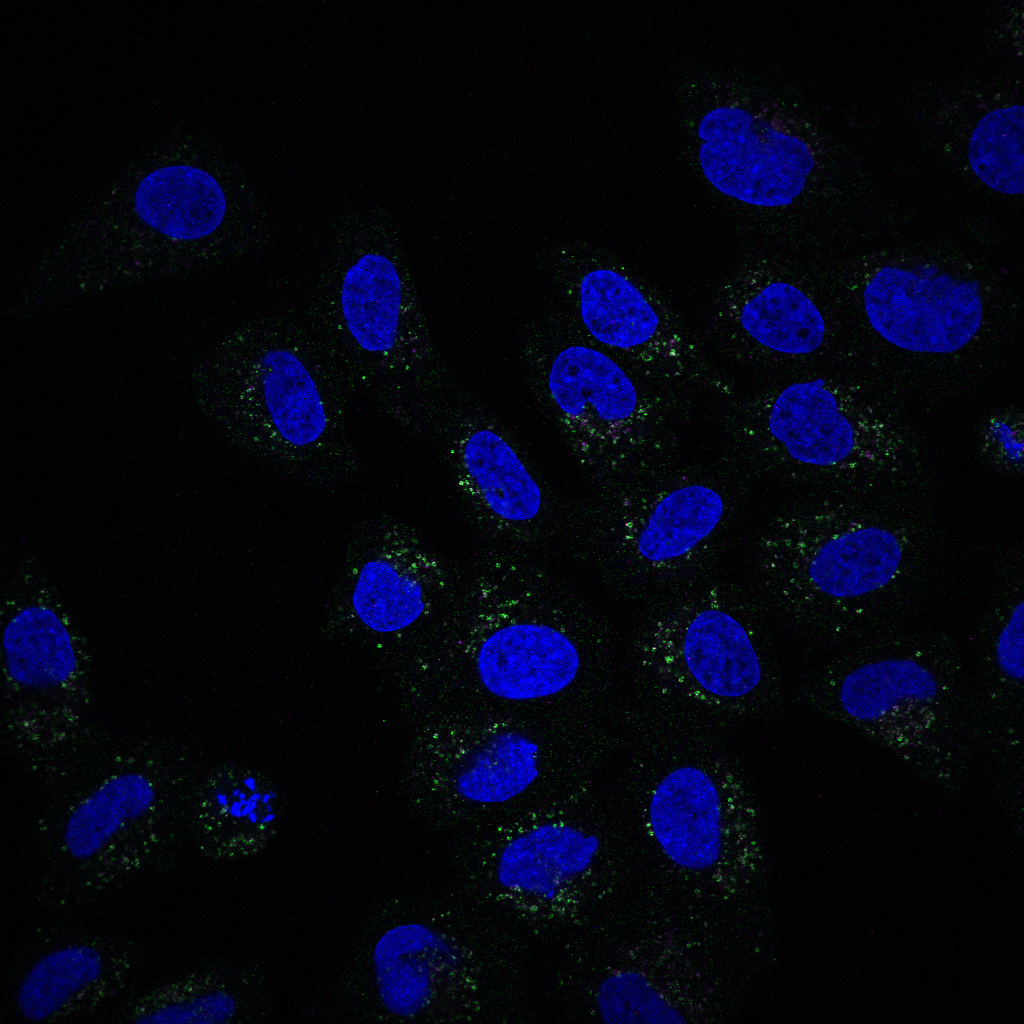

Supplement: Supplementary file 6 — Source Data for Figure 2 [file EMBR-24-e57300-s001.zip › Fig 2/2C/siLuc_LLOMe 1h.tif]

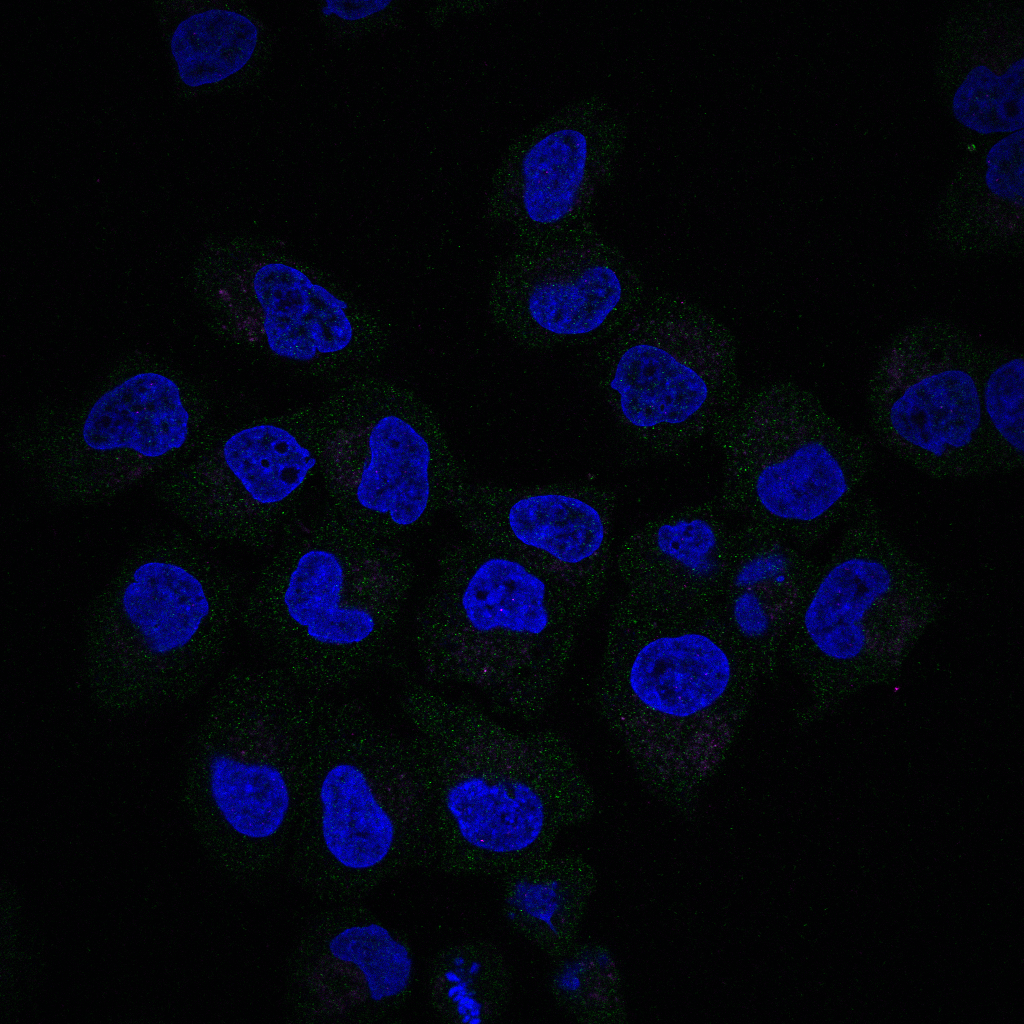

Supplement: Supplementary file 6 — Source Data for Figure 2 [file EMBR-24-e57300-s001.zip › Fig 2/2C/siSTK38 #1_non-treated.tif]

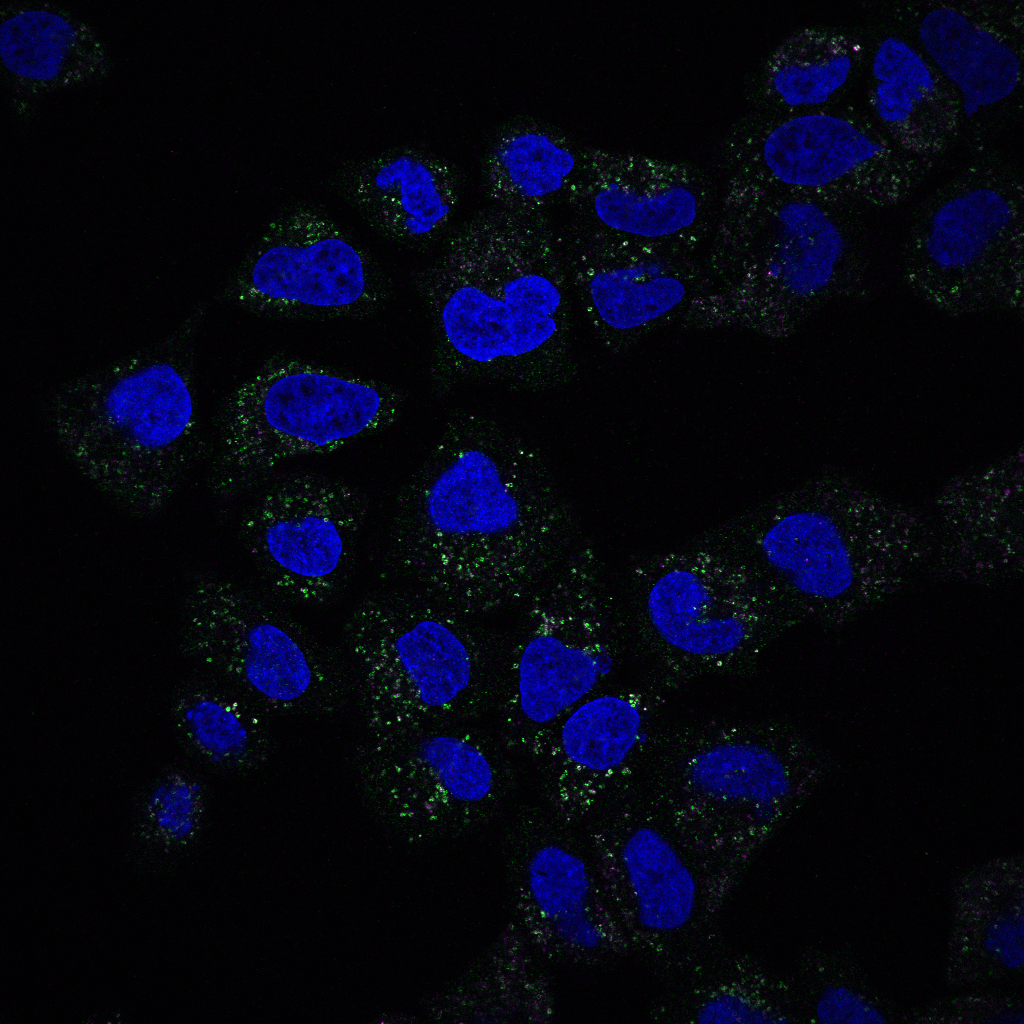

Supplement: Supplementary file 6 — Source Data for Figure 2 [file EMBR-24-e57300-s001.zip › Fig 2/2C/siSTK38 #1_LLOMe 1h.tif]

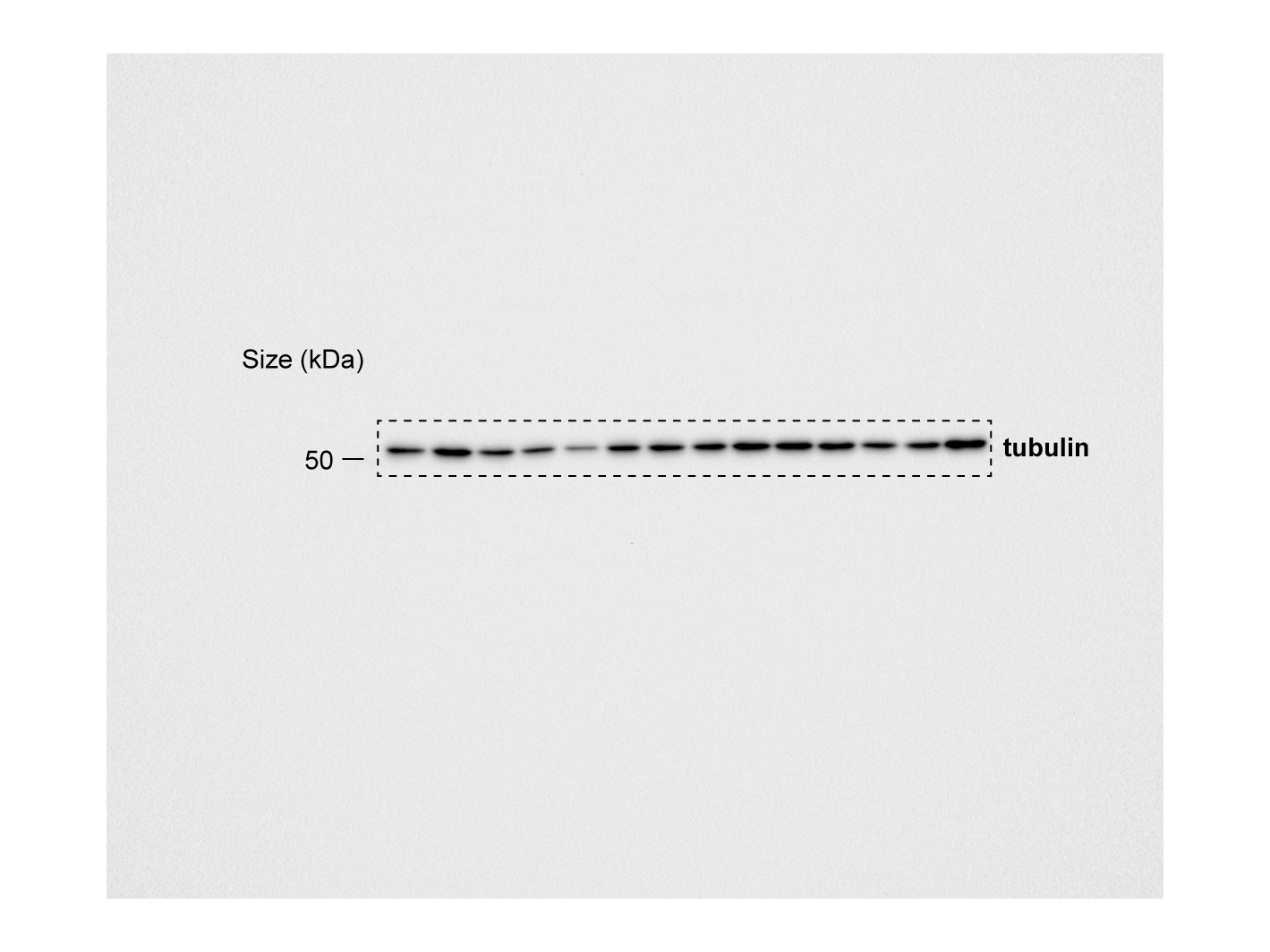

Supplement: Supplementary file 7 — Source Data for Figure 3 [file EMBR-24-e57300-s005.zip › Fig 3/3B/western tubulin.tif]

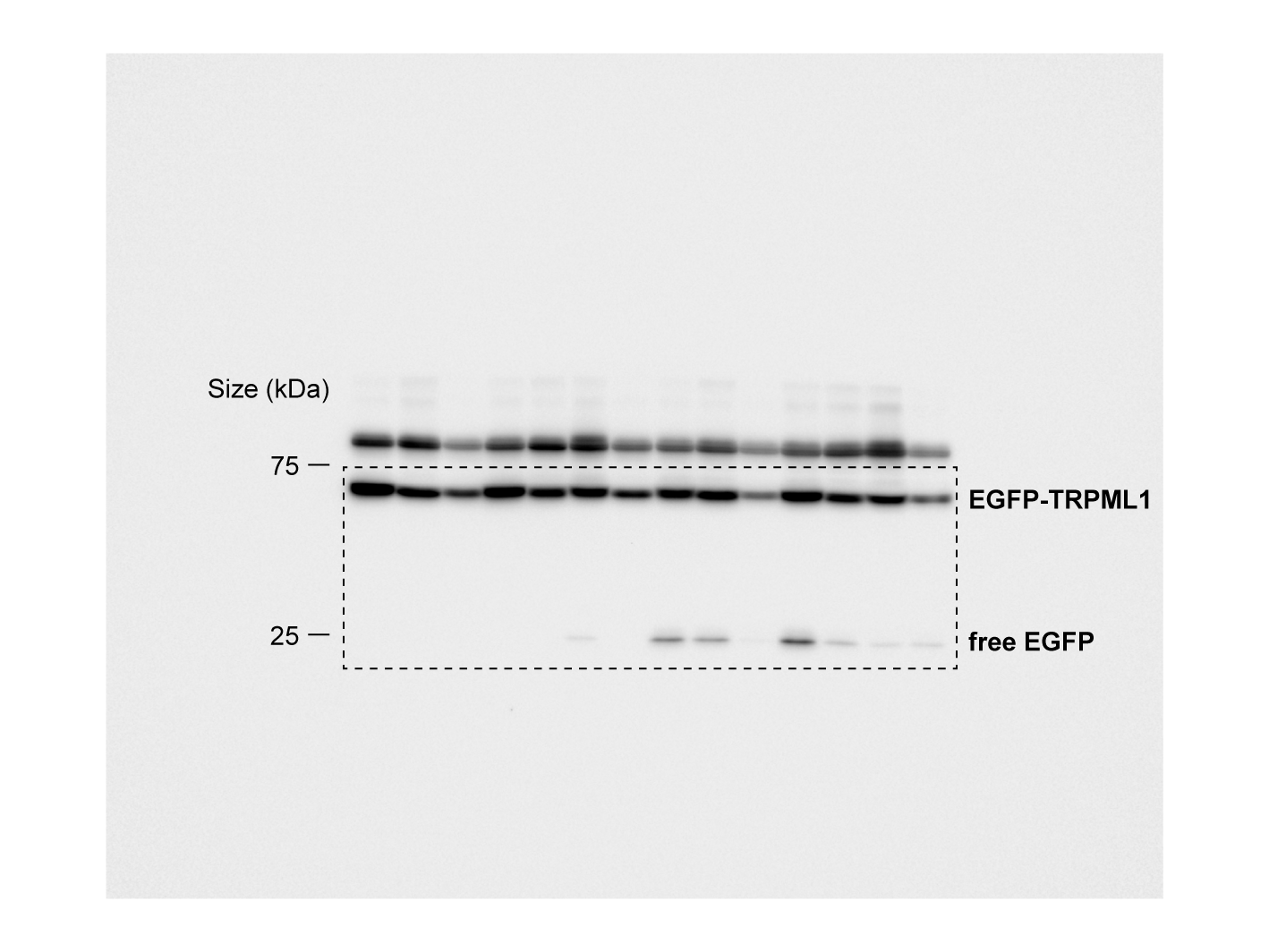

Supplement: Supplementary file 7 — Source Data for Figure 3 [file EMBR-24-e57300-s005.zip › Fig 3/3B/western EGFP.tif]

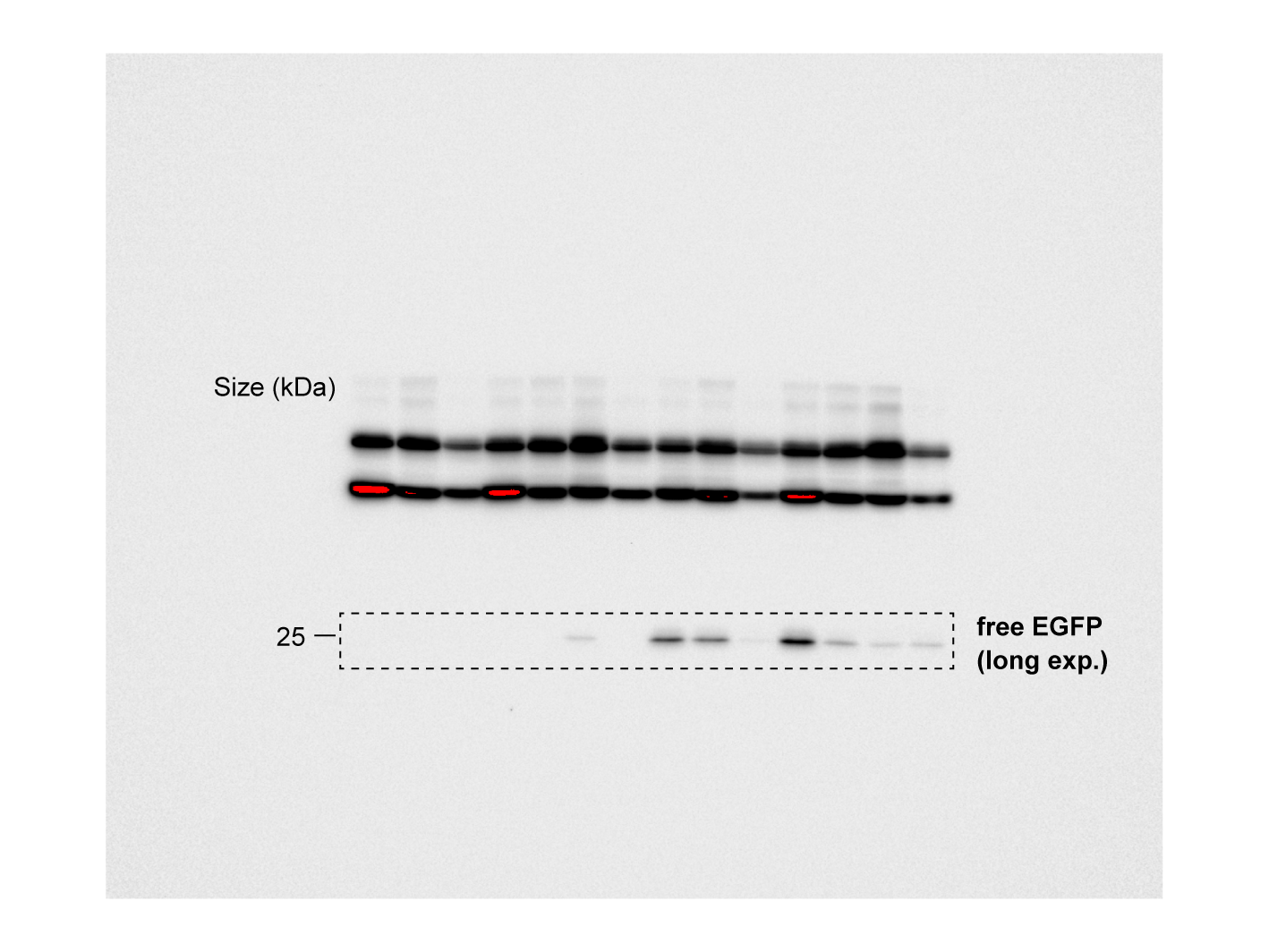

Supplement: Supplementary file 7 — Source Data for Figure 3 [file EMBR-24-e57300-s005.zip › Fig 3/3B/western EGFP (long exposure).tif]

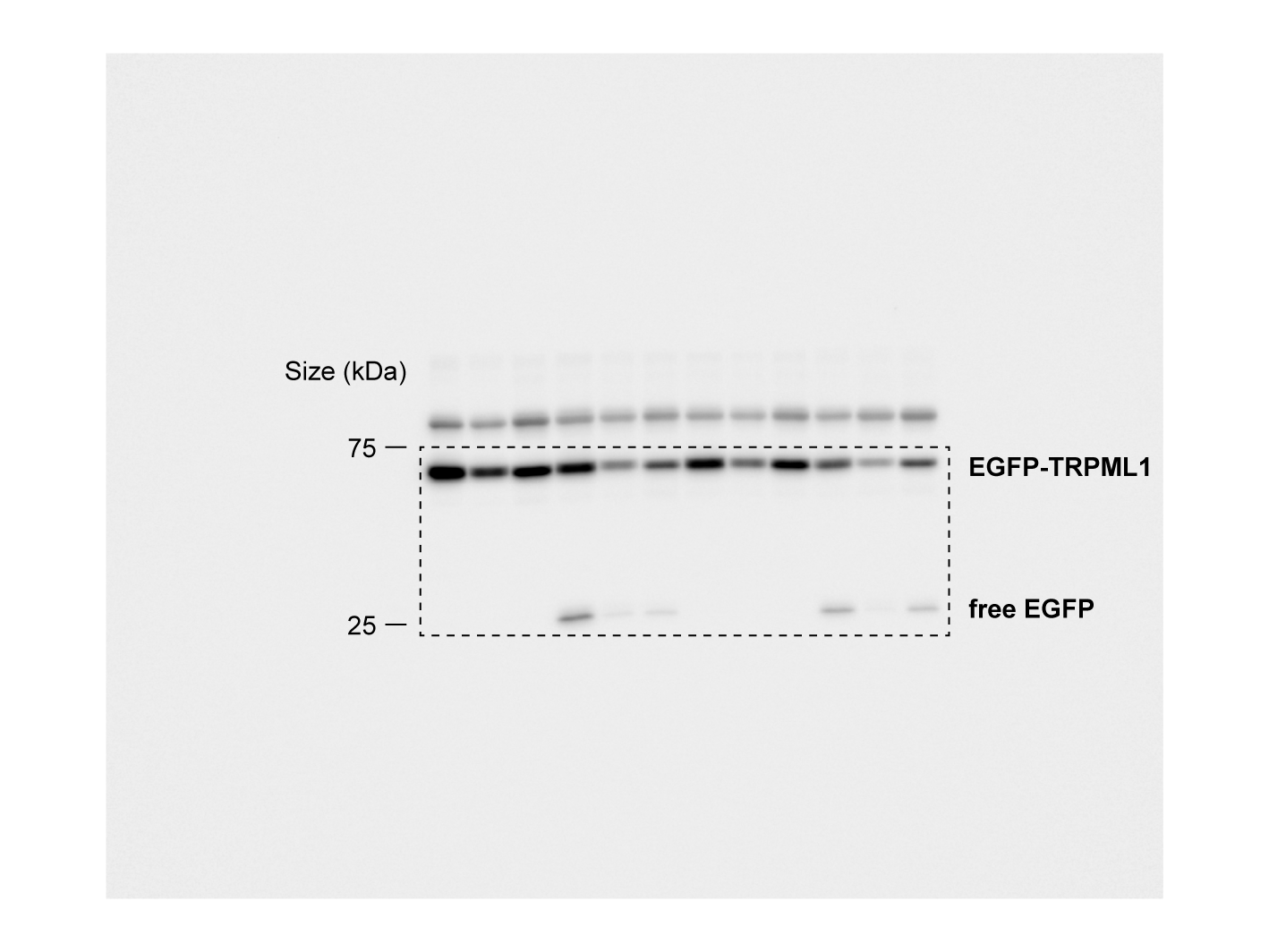

Supplement: Supplementary file 7 — Source Data for Figure 3 [file EMBR-24-e57300-s005.zip › Fig 3/3D/western EGFP.tif]

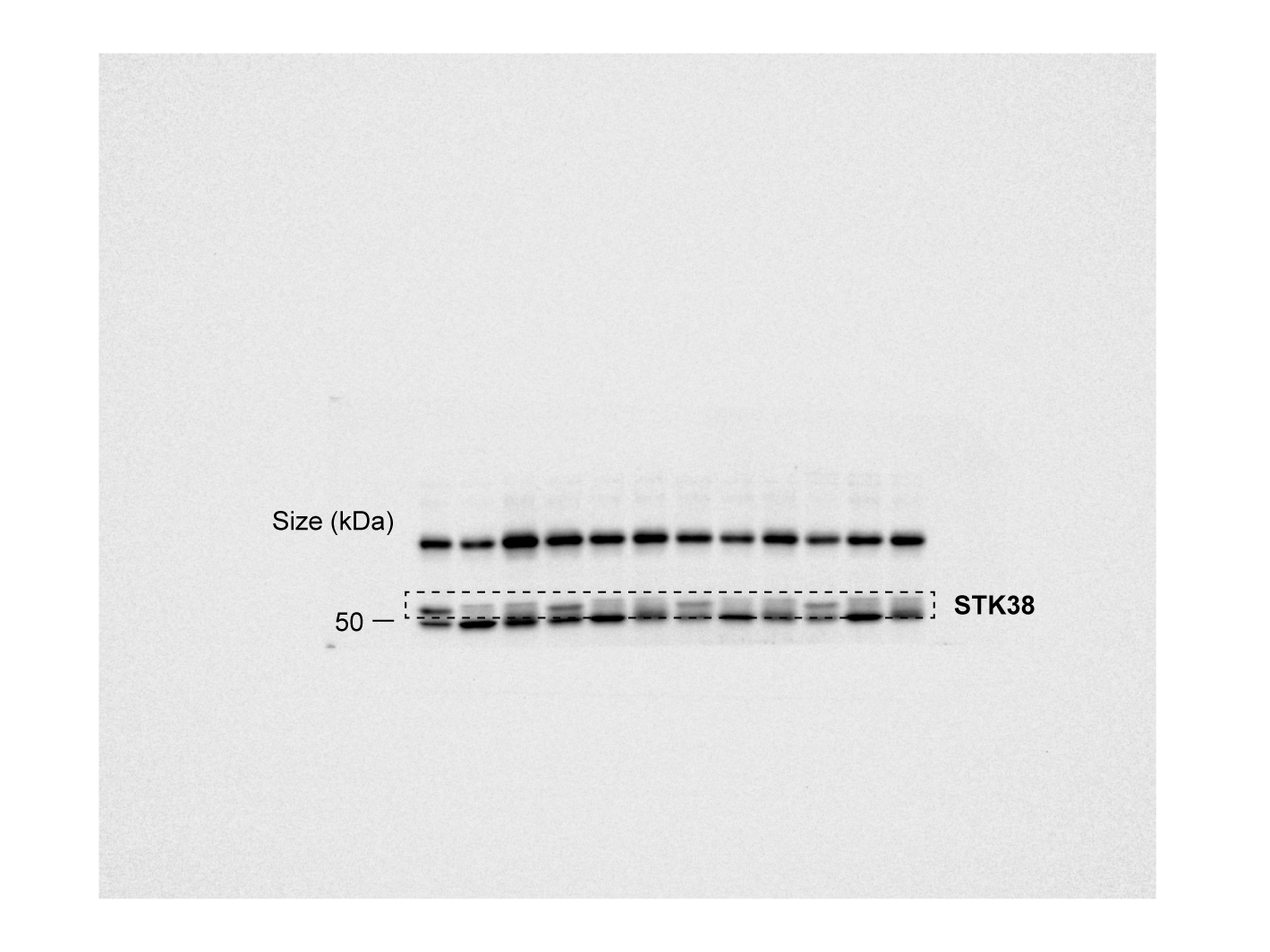

Supplement: Supplementary file 7 — Source Data for Figure 3 [file EMBR-24-e57300-s005.zip › Fig 3/3D/western STK38.tif]

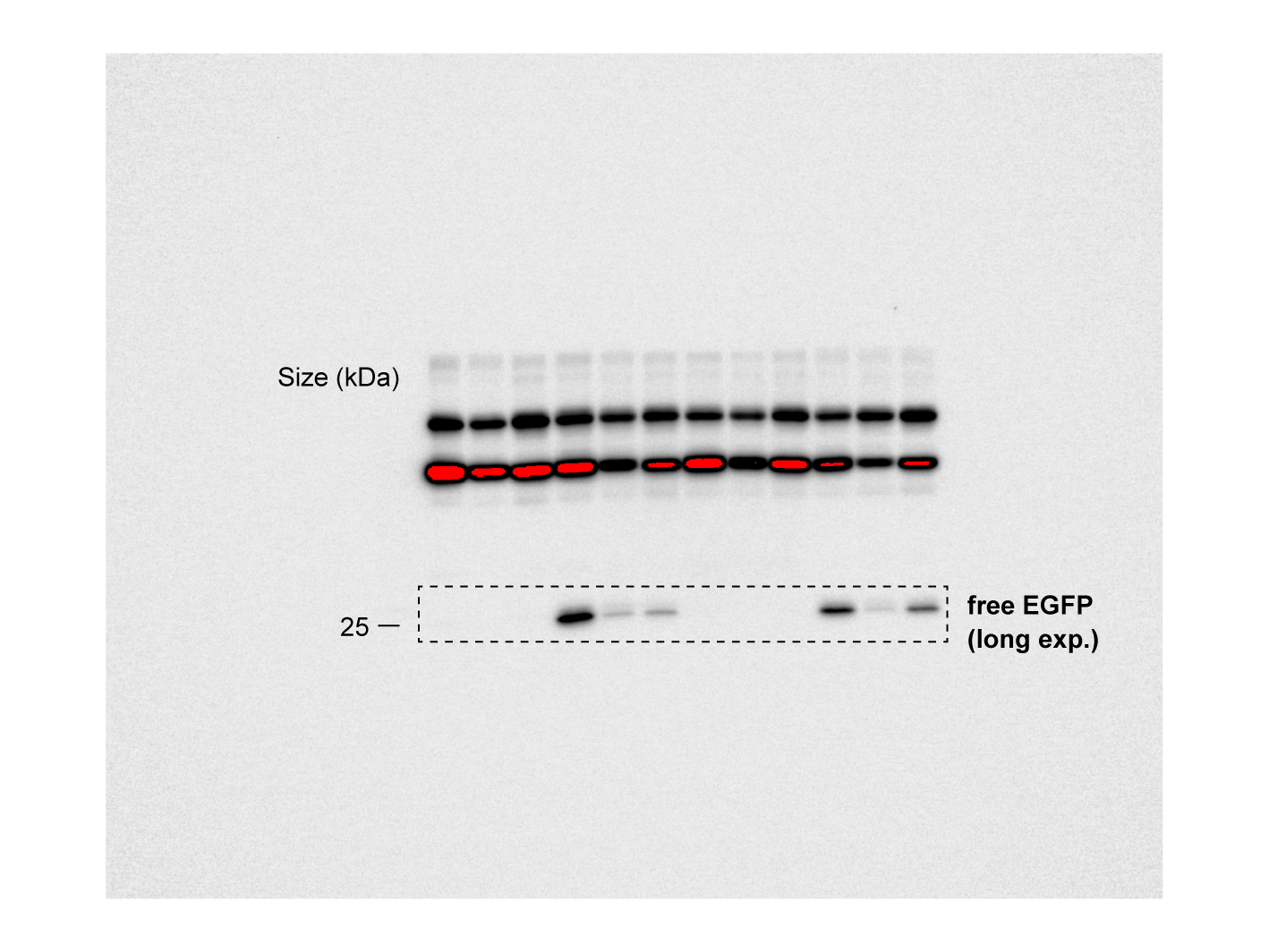

Supplement: Supplementary file 7 — Source Data for Figure 3 [file EMBR-24-e57300-s005.zip › Fig 3/3D/western EGFP (long exposure).tif]

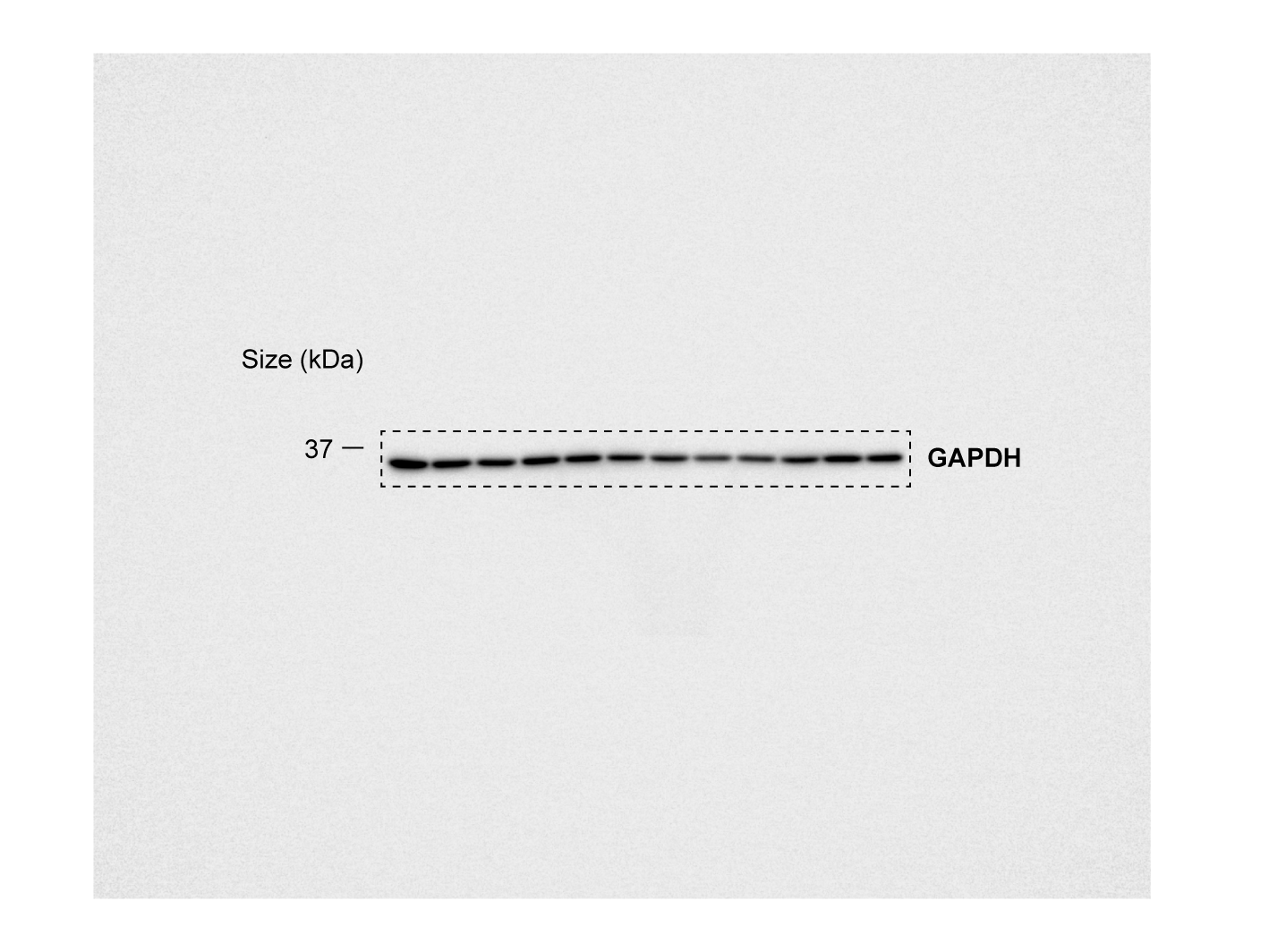

Supplement: Supplementary file 7 — Source Data for Figure 3 [file EMBR-24-e57300-s005.zip › Fig 3/3D/western GAPDH.tif]

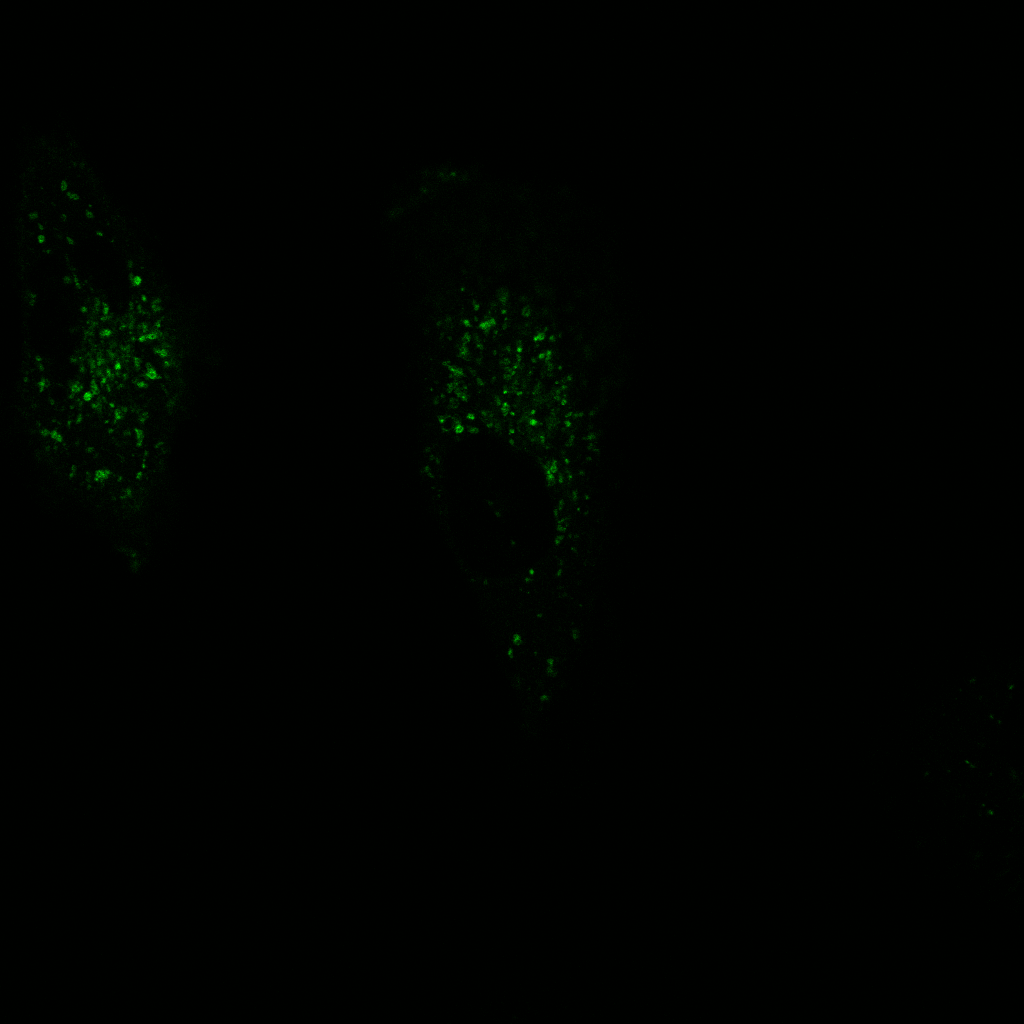

Supplement: Supplementary file 7 — Source Data for Figure 3 [file EMBR-24-e57300-s005.zip › Fig 3/3J/non-treated_LAMP1-GFP.tif]

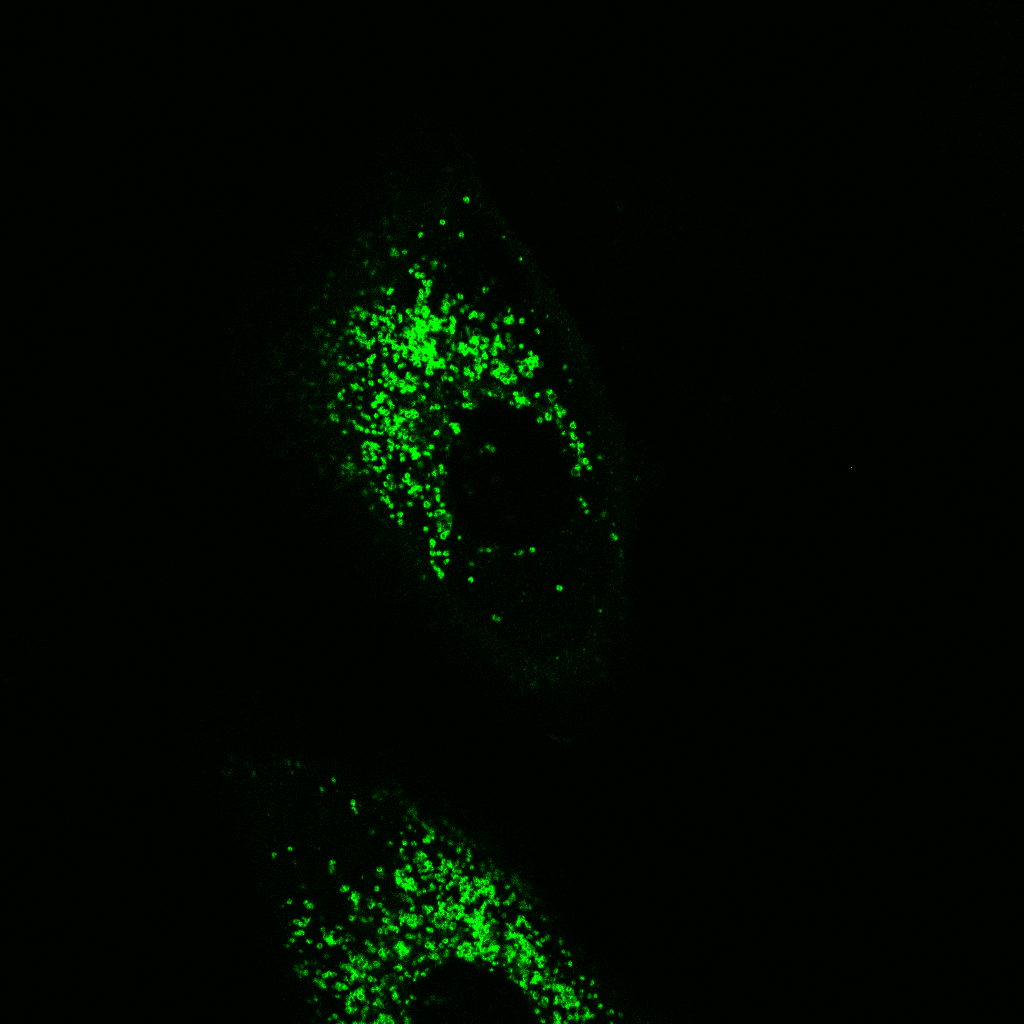

Supplement: Supplementary file 7 — Source Data for Figure 3 [file EMBR-24-e57300-s005.zip › Fig 3/3J/NH4Cl_LAMP1-GFP.tif]

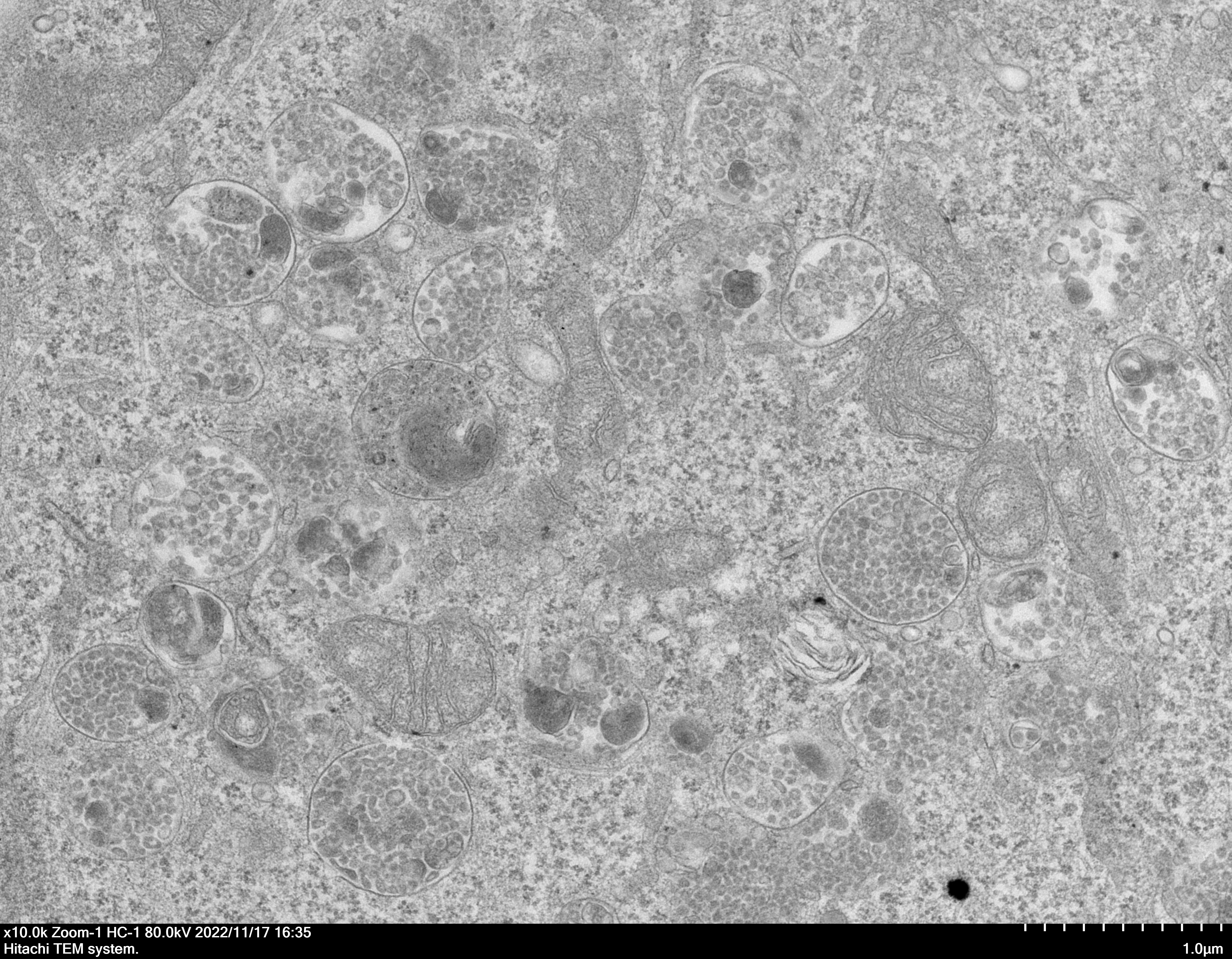

Supplement: Supplementary file 7 — Source Data for Figure 3 [file EMBR-24-e57300-s005.zip › Fig 3/3H/siLuc_NH4Cl.tif]

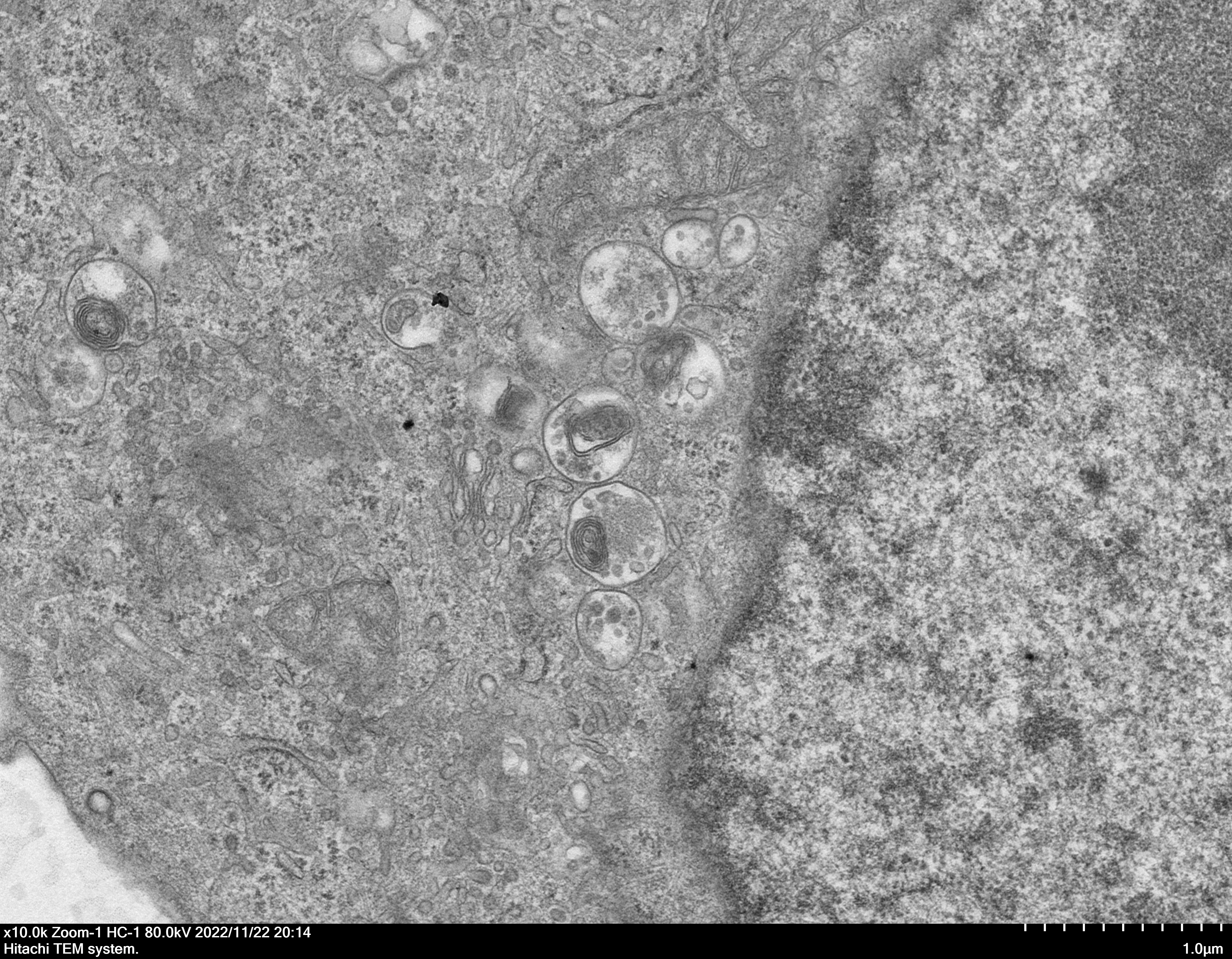

Supplement: Supplementary file 7 — Source Data for Figure 3 [file EMBR-24-e57300-s005.zip › Fig 3/3H/siLuc_non-treated.tif]

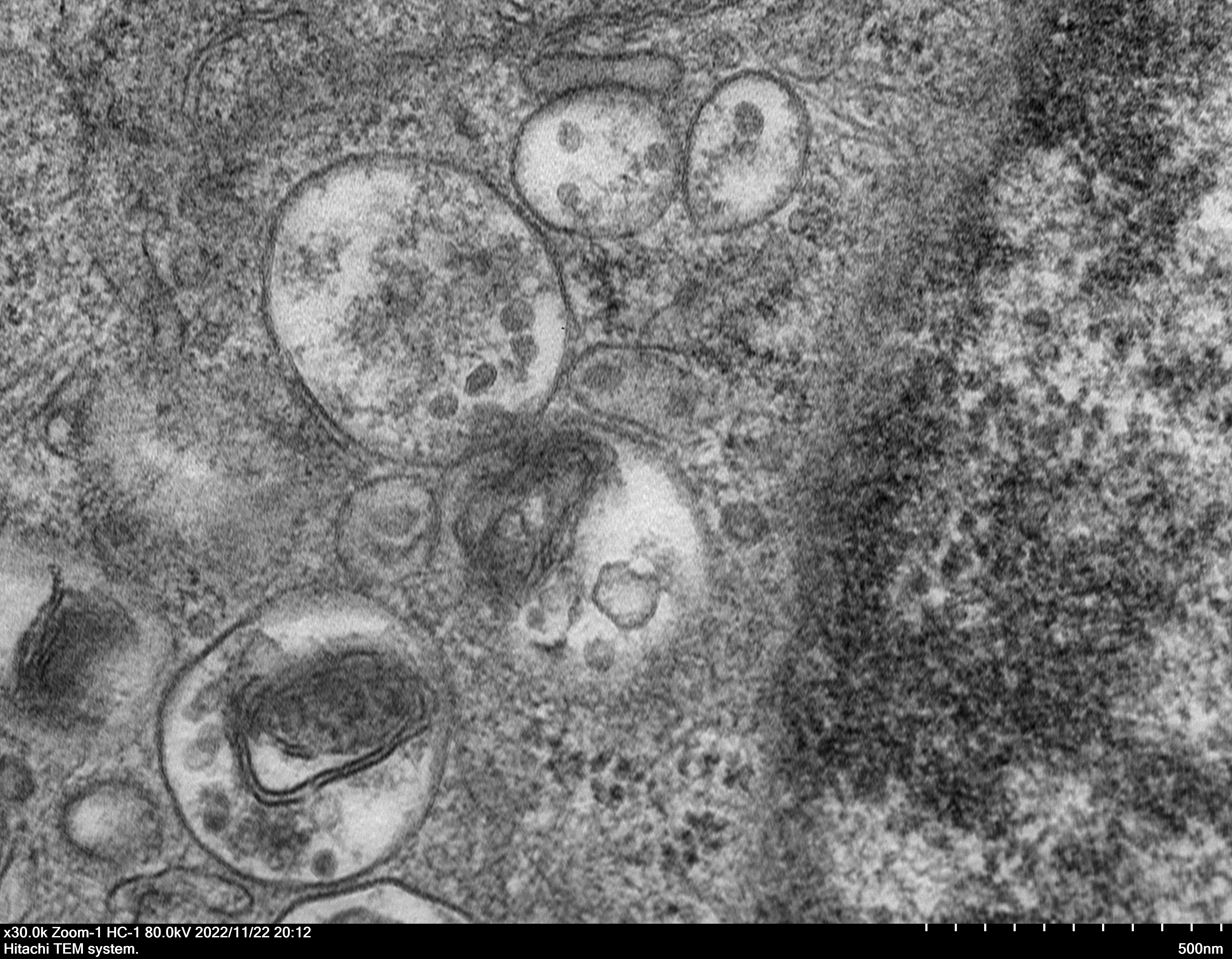

Supplement: Supplementary file 7 — Source Data for Figure 3 [file EMBR-24-e57300-s005.zip › Fig 3/3H/siLuc_non-treated_magnified.tif]

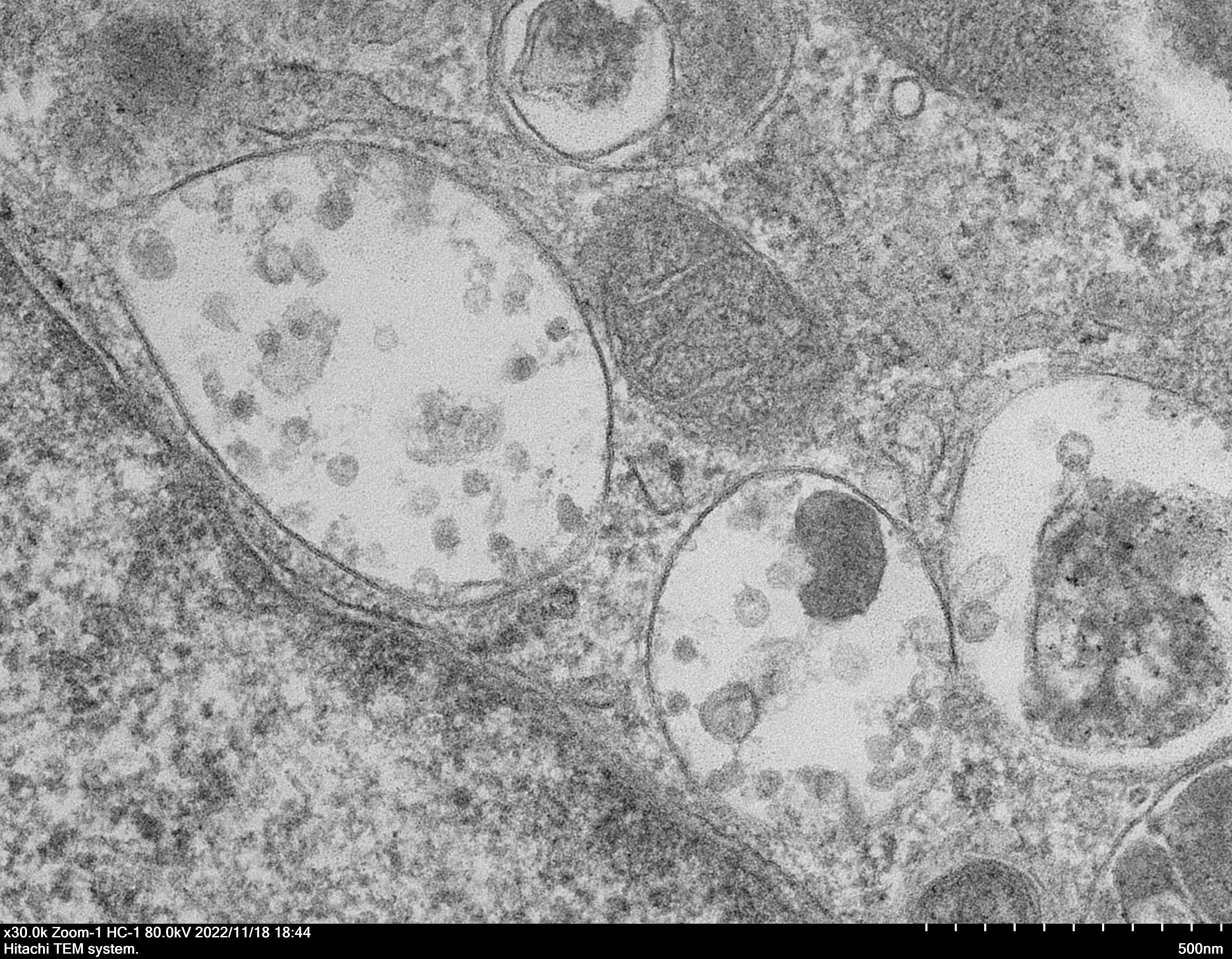

Supplement: Supplementary file 7 — Source Data for Figure 3 [file EMBR-24-e57300-s005.zip › Fig 3/3H/siSTK38_NH4Cl_magnified.tif]

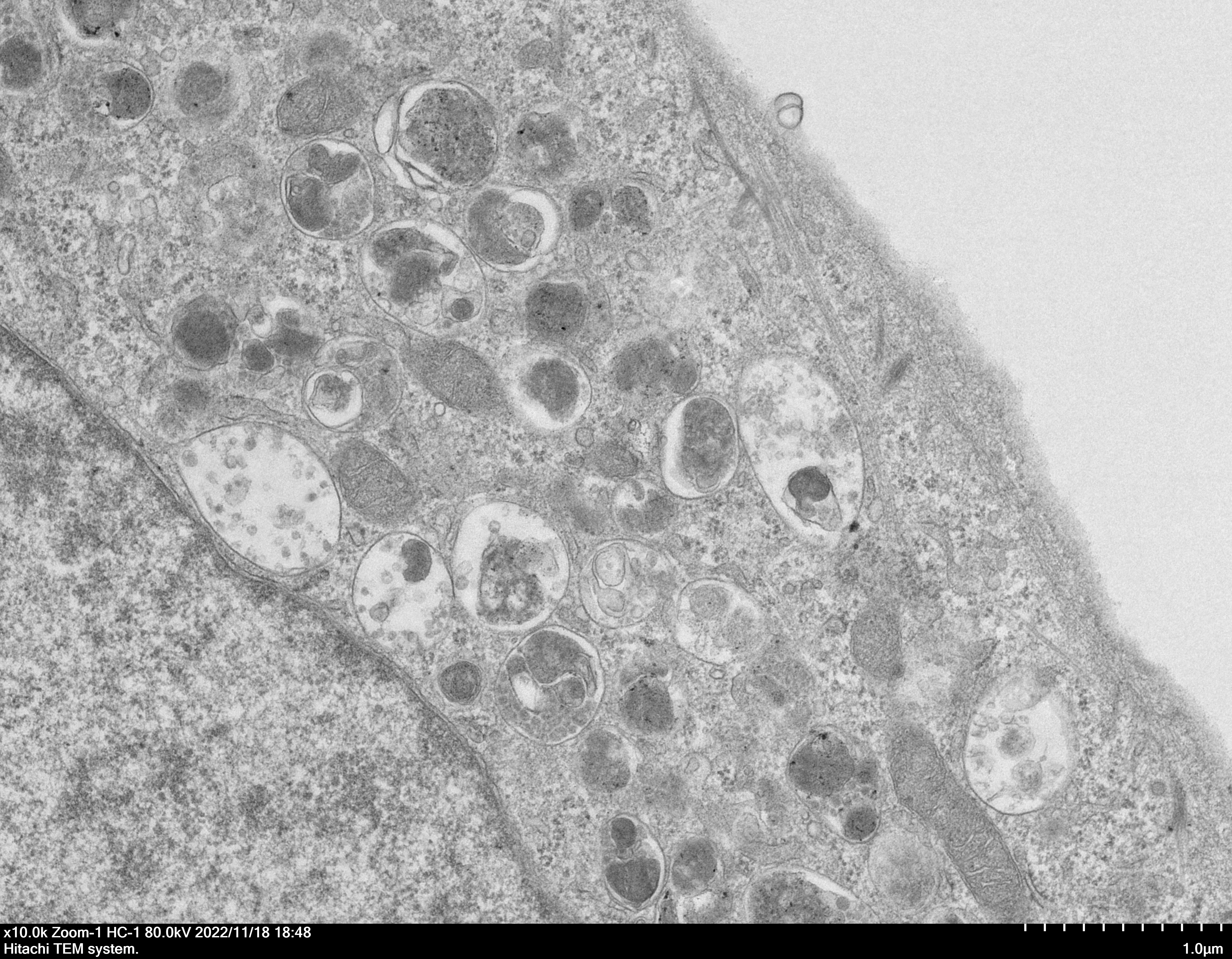

Supplement: Supplementary file 7 — Source Data for Figure 3 [file EMBR-24-e57300-s005.zip › Fig 3/3H/siSTK38_NH4Cl.tif]

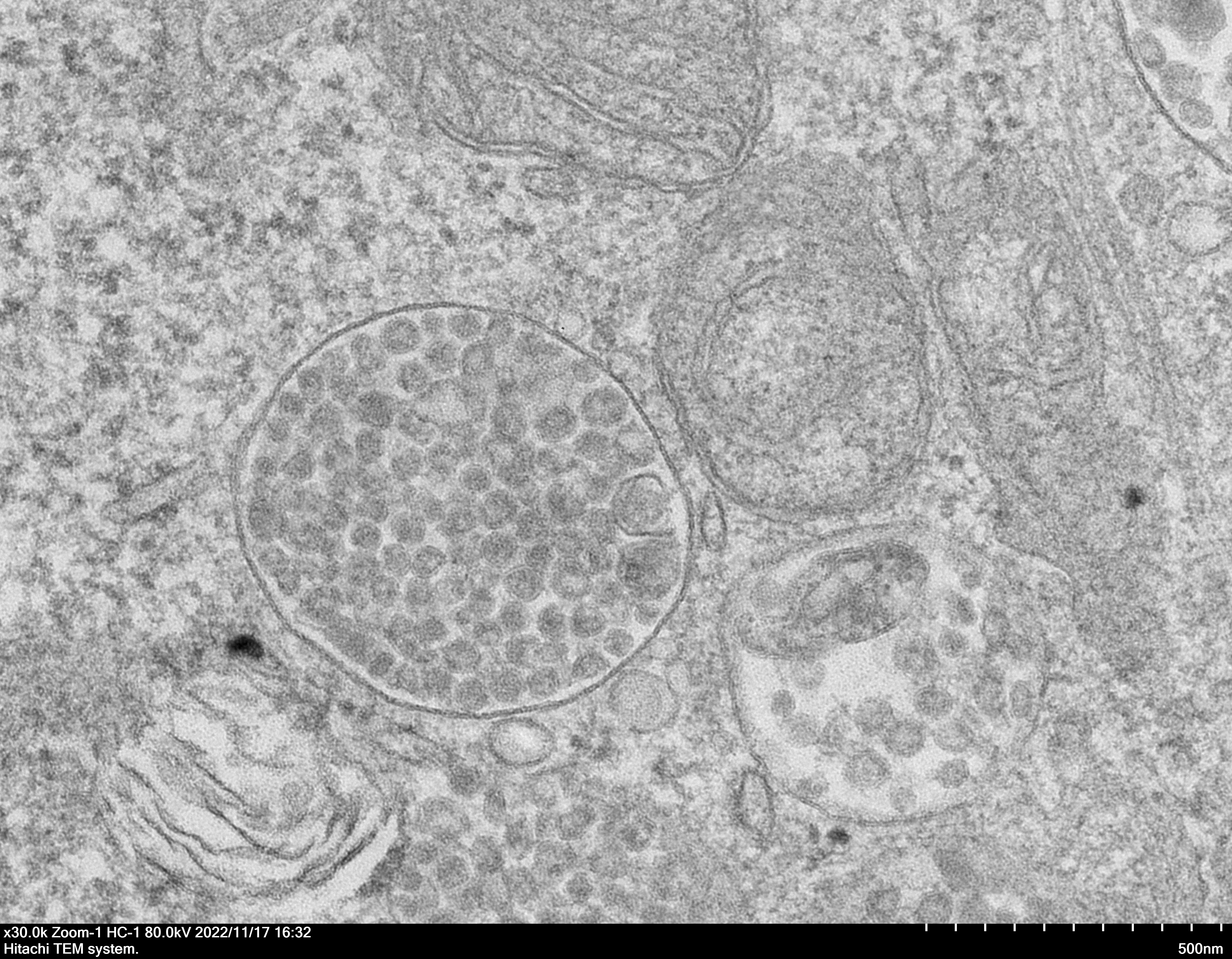

Supplement: Supplementary file 7 — Source Data for Figure 3 [file EMBR-24-e57300-s005.zip › Fig 3/3H/siLuc_NH4Cl_magnified.tif]

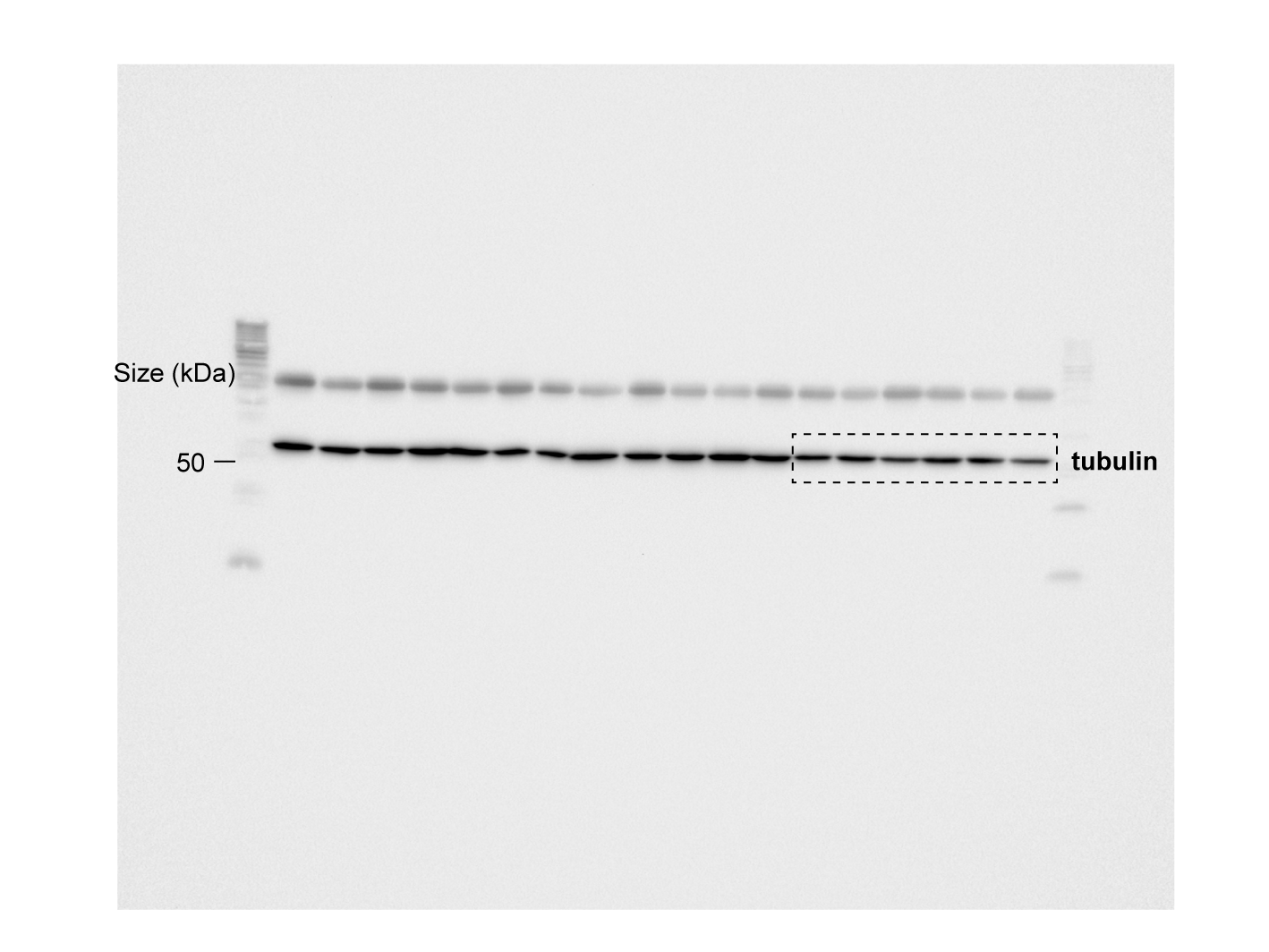

Supplement: Supplementary file 7 — Source Data for Figure 3 [file EMBR-24-e57300-s005.zip › Fig 3/3F/western tubulin.tif]

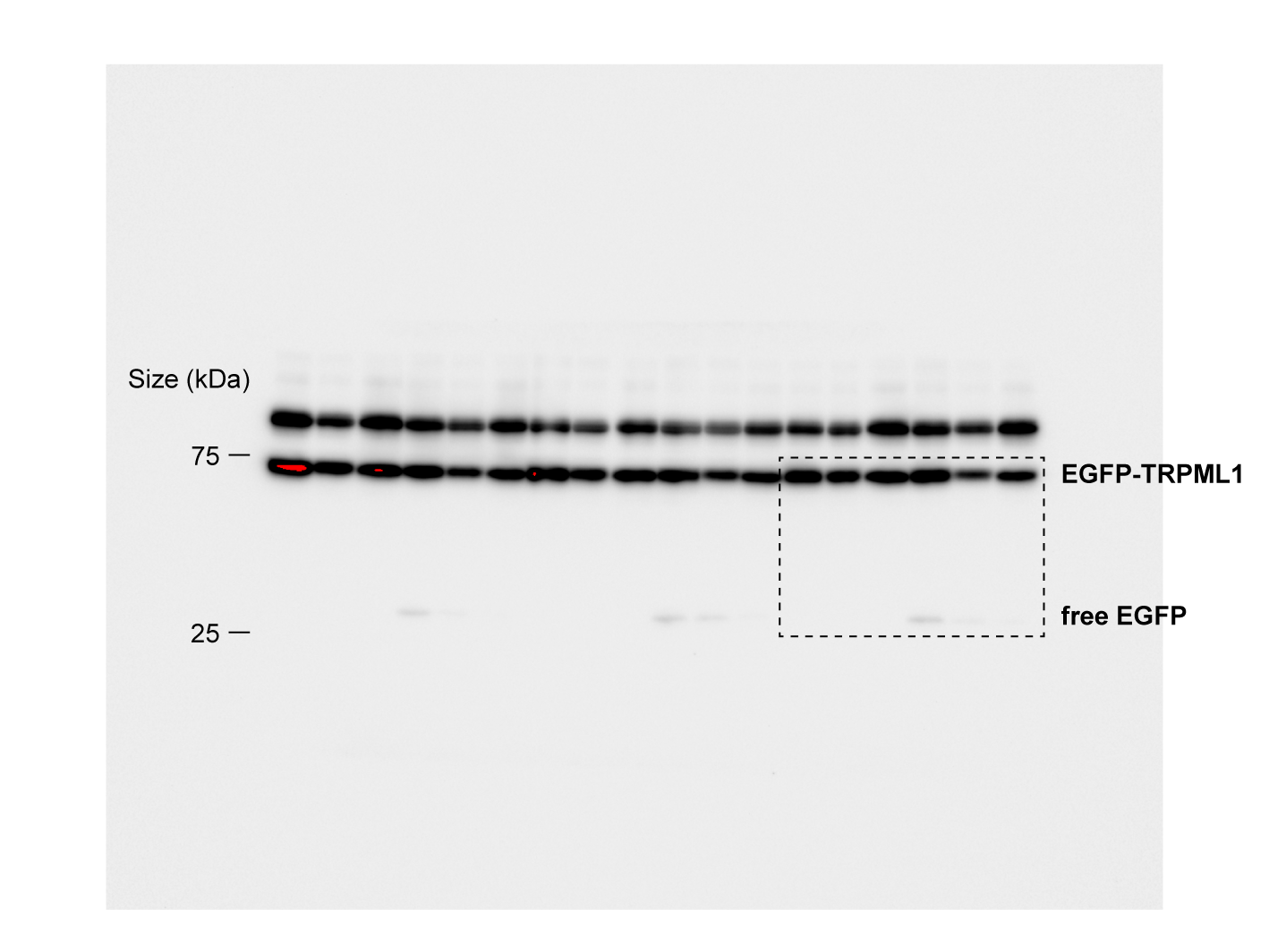

Supplement: Supplementary file 7 — Source Data for Figure 3 [file EMBR-24-e57300-s005.zip › Fig 3/3F/western EGFP.tif]

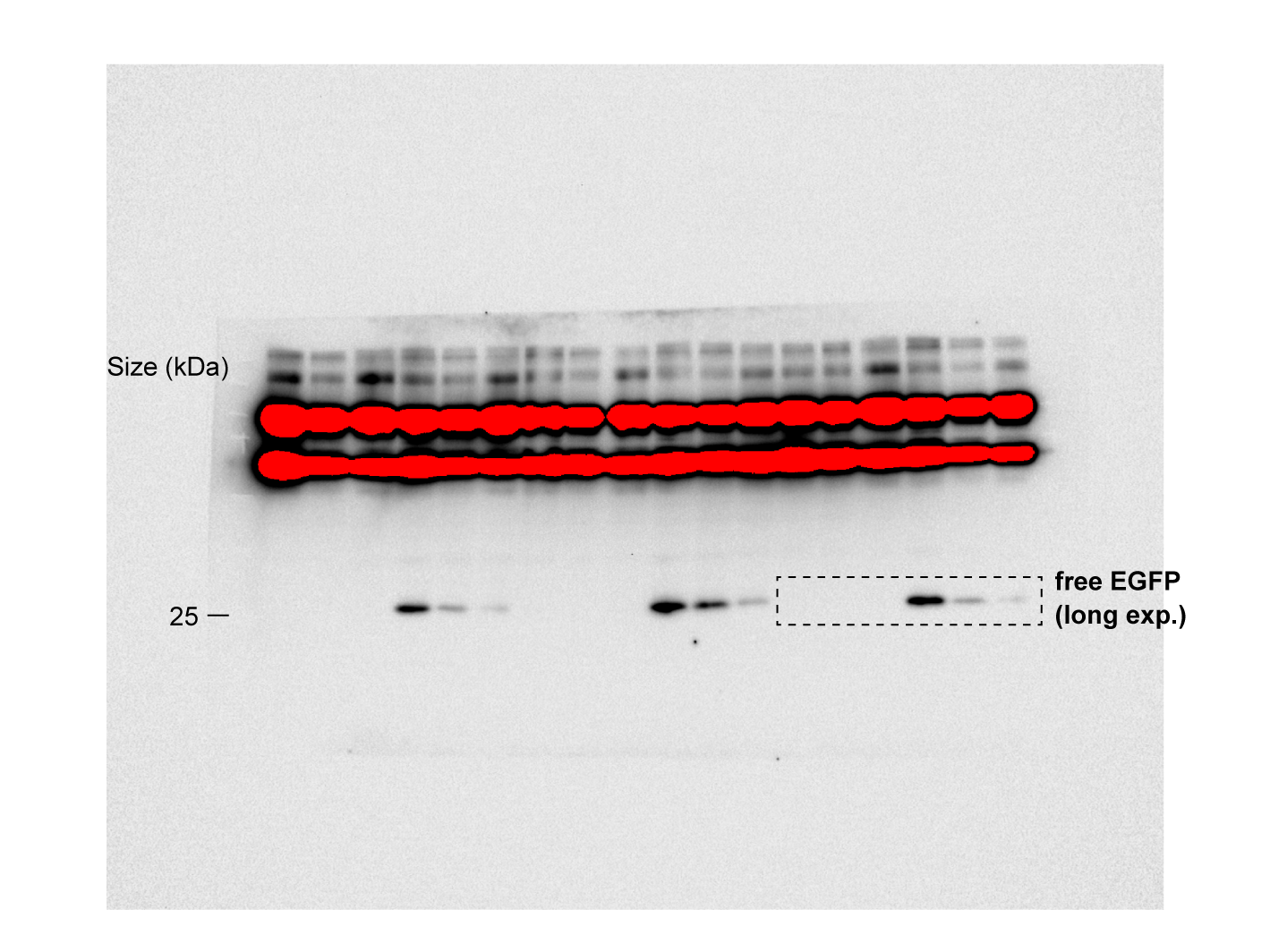

Supplement: Supplementary file 7 — Source Data for Figure 3 [file EMBR-24-e57300-s005.zip › Fig 3/3F/western EGFP (long exposure).tif]

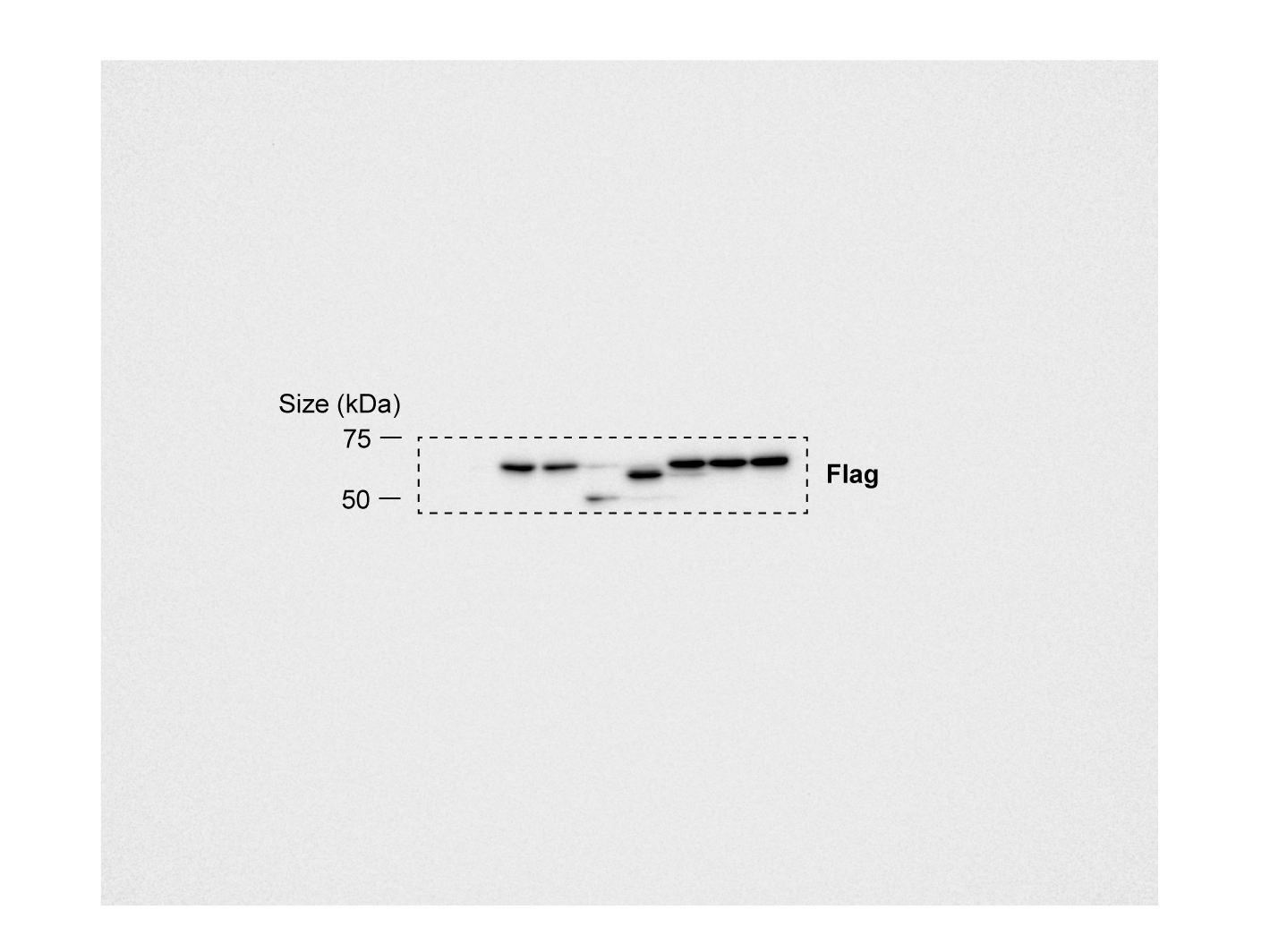

Supplement: Supplementary file 8 — Source Data for Figure 4 [file EMBR-24-e57300-s002.zip › Fig 4/4B/western Flag.tif]

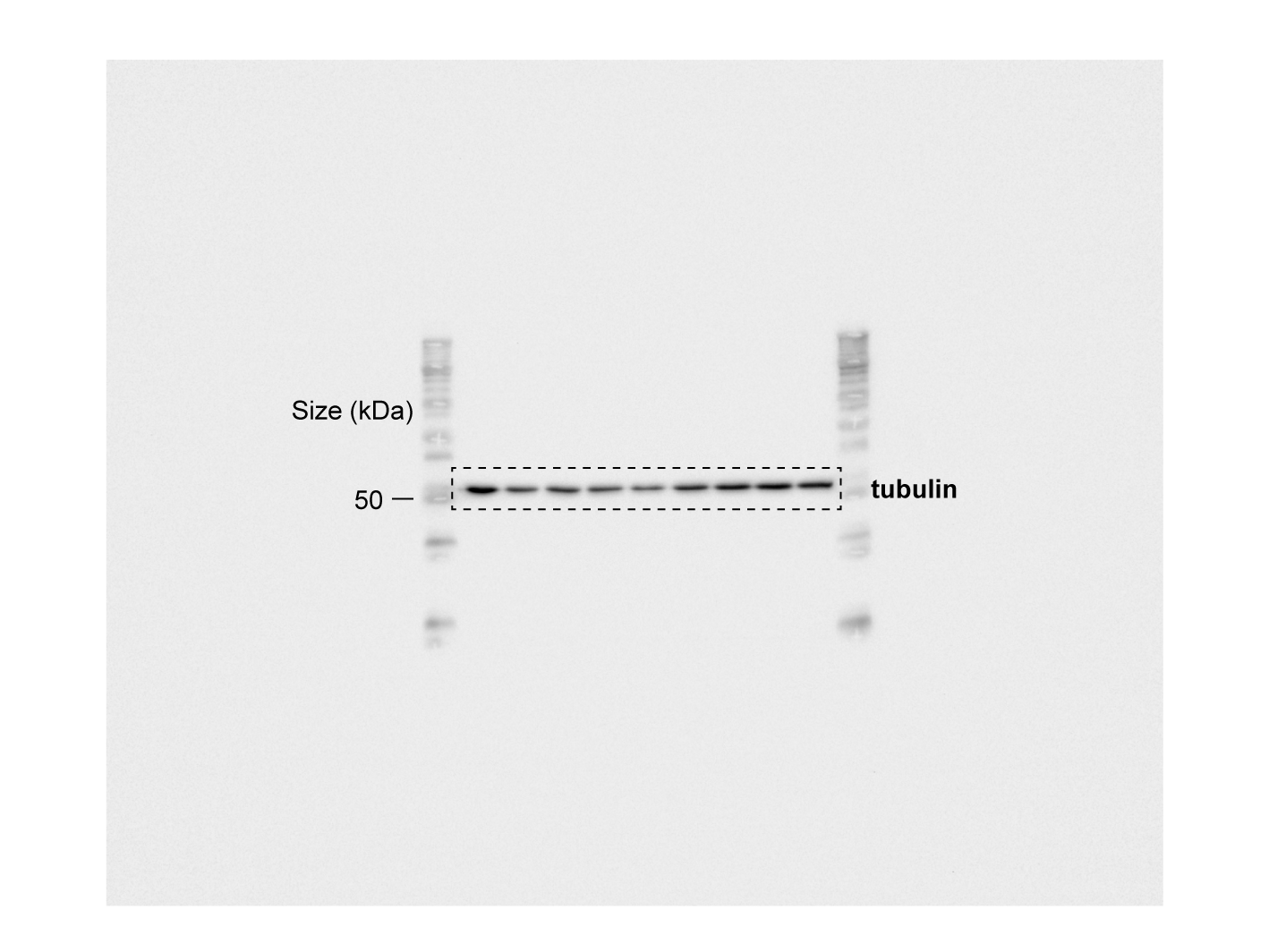

Supplement: Supplementary file 8 — Source Data for Figure 4 [file EMBR-24-e57300-s002.zip › Fig 4/4B/western tubulin.tif]

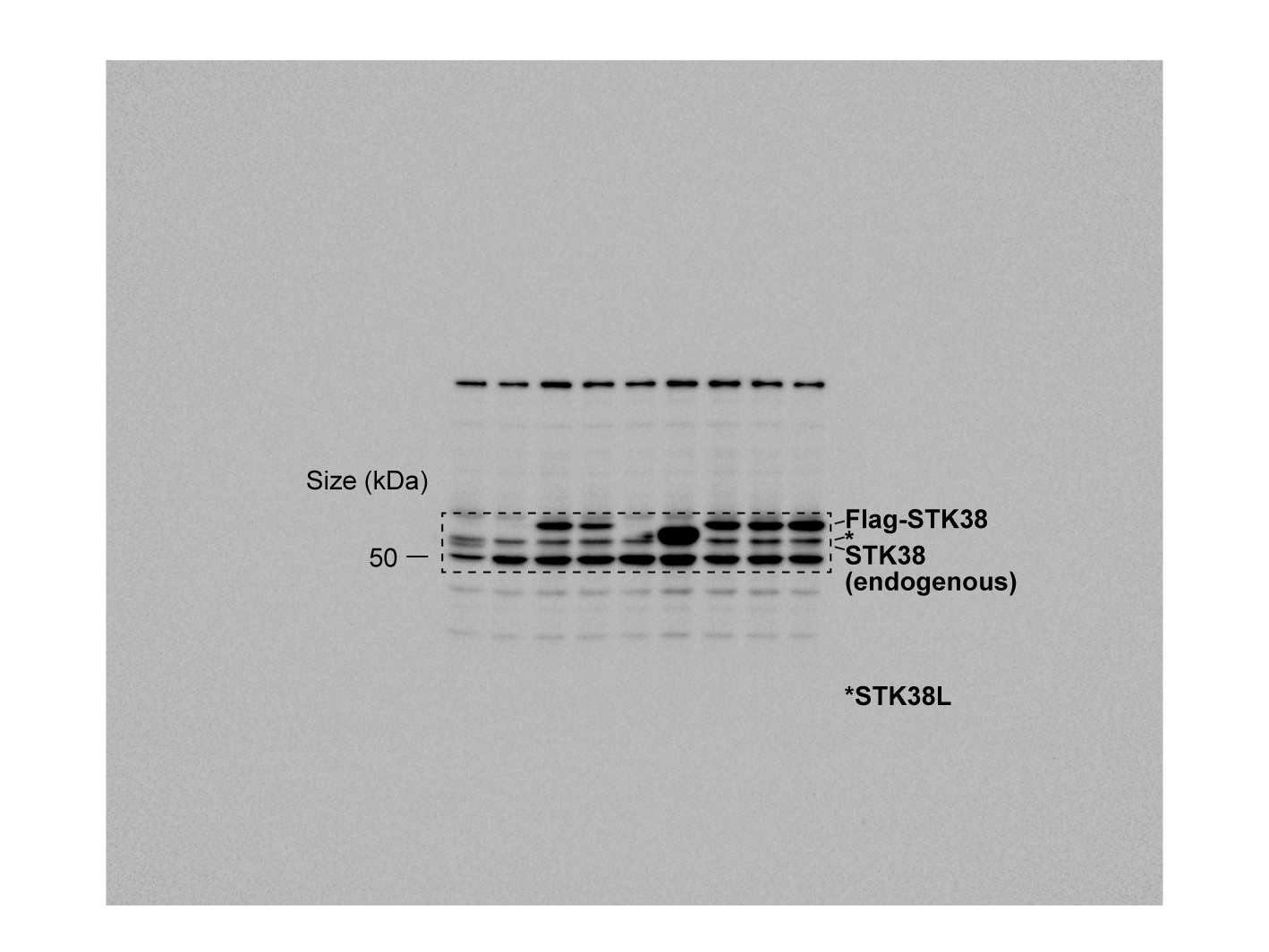

Supplement: Supplementary file 8 — Source Data for Figure 4 [file EMBR-24-e57300-s002.zip › Fig 4/4B/western STK38.tif]

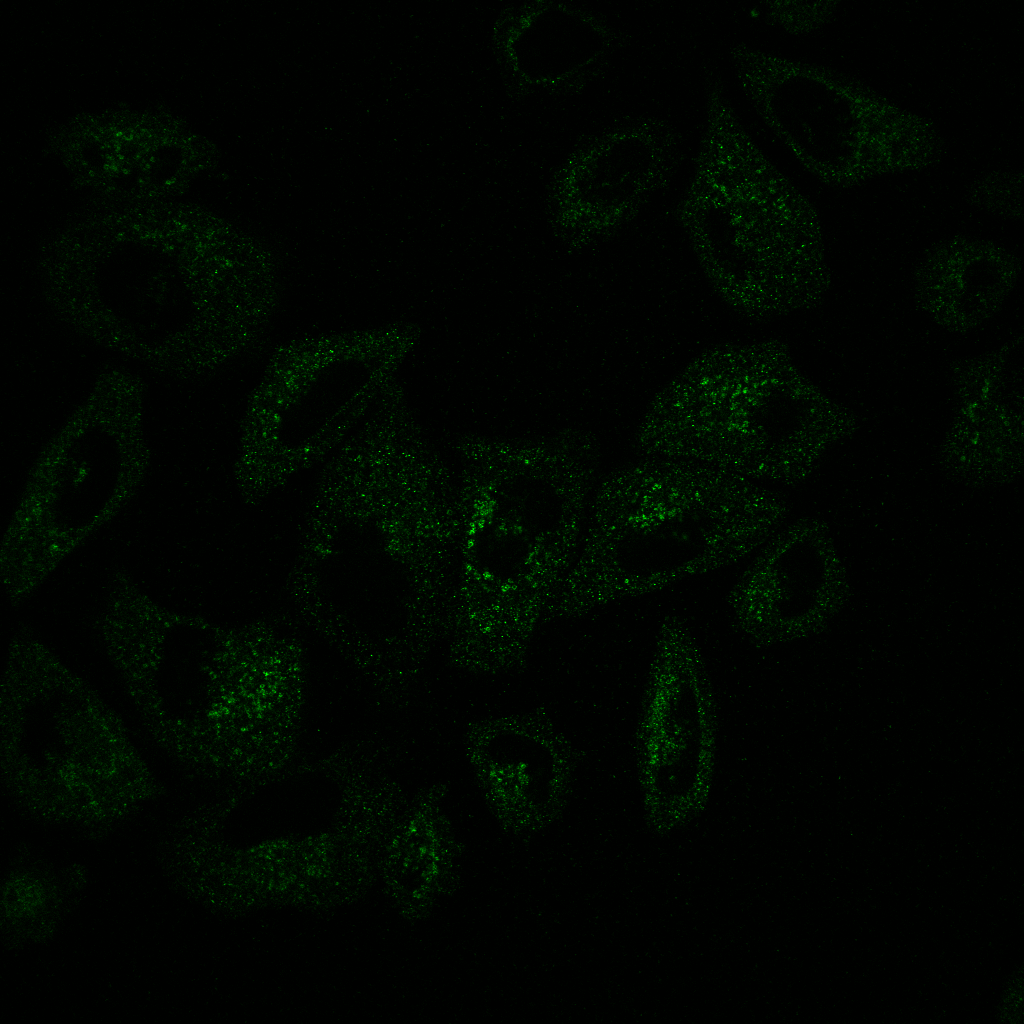

Supplement: Supplementary file 8 — Source Data for Figure 4 [file EMBR-24-e57300-s002.zip › Fig 4/4A/siSTK38_Flag-STK38 WT_LLOMe_VPS4.tif]

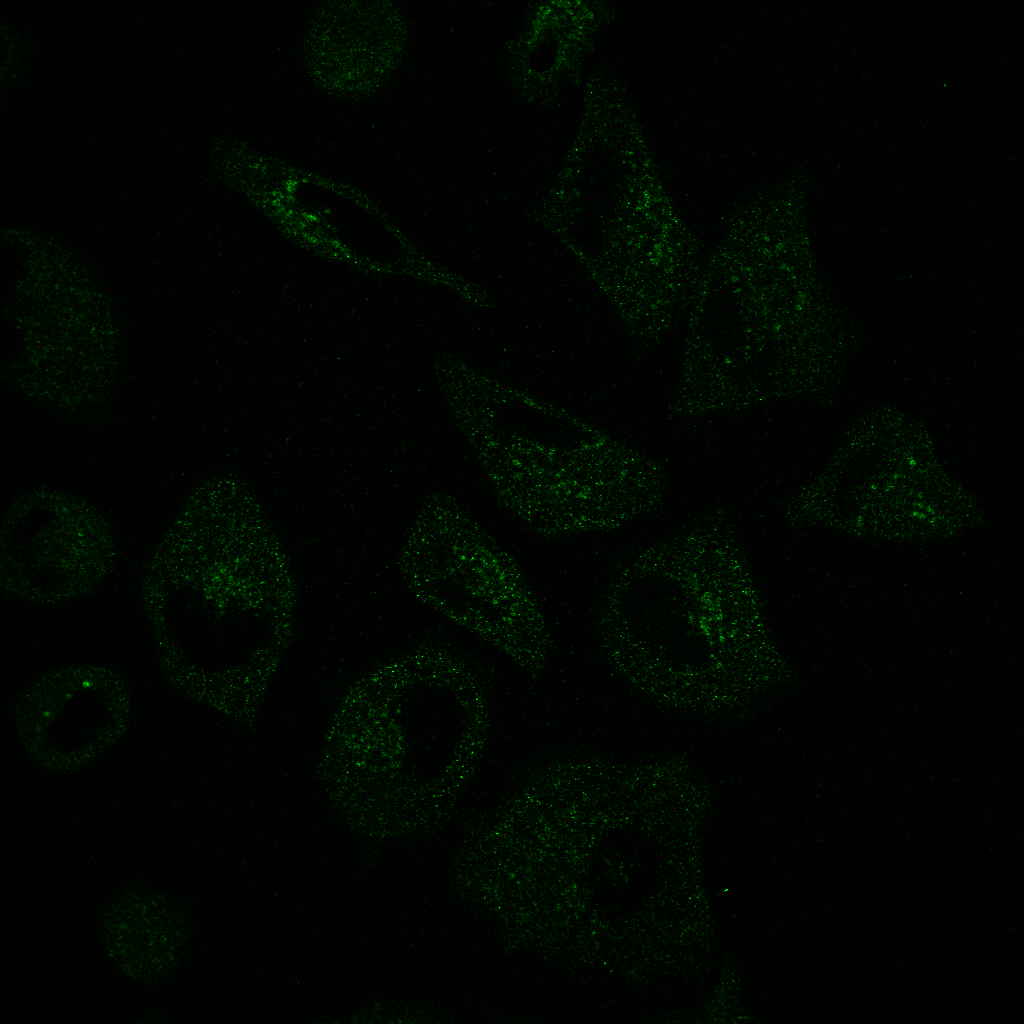

Supplement: Supplementary file 8 — Source Data for Figure 4 [file EMBR-24-e57300-s002.zip › Fig 4/4A/siSTK38_Flag-STK38 S281A_LLOMe_VPS4.tif]

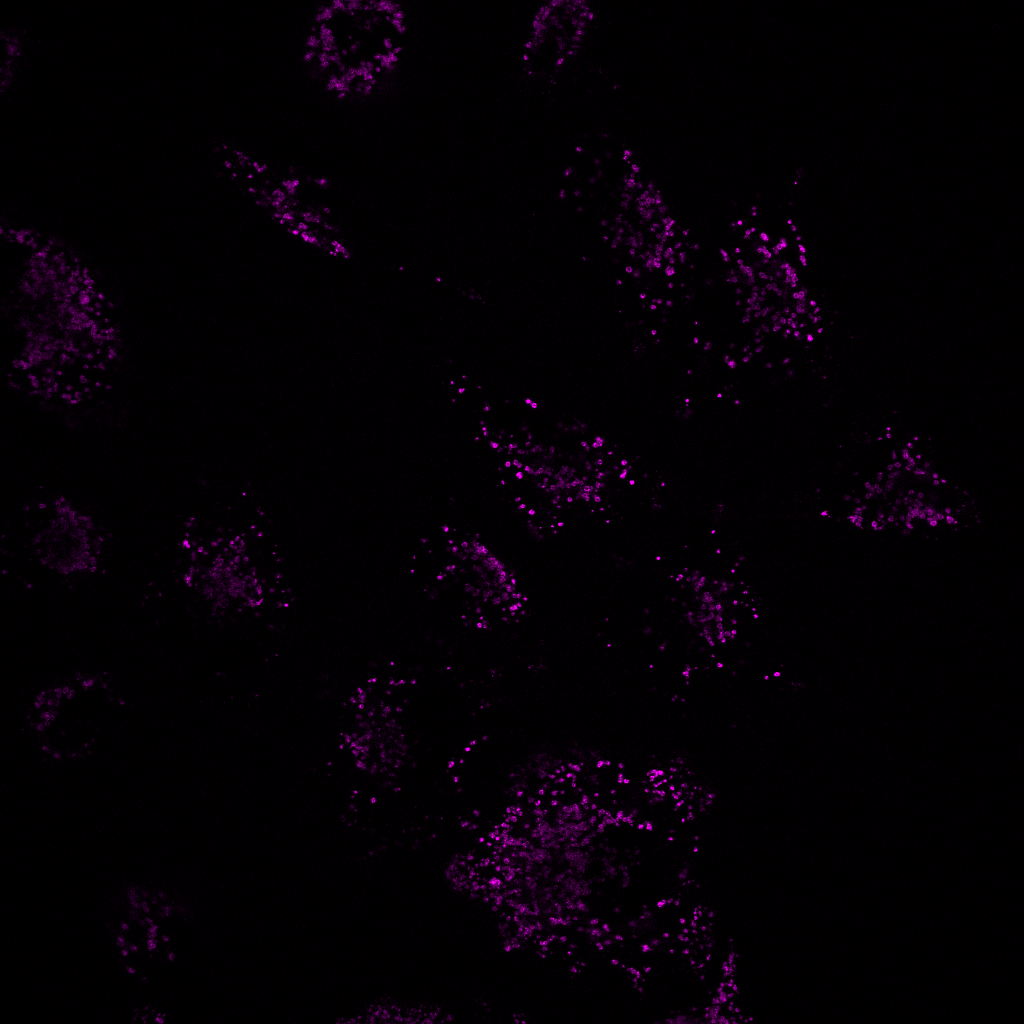

Supplement: Supplementary file 8 — Source Data for Figure 4 [file EMBR-24-e57300-s002.zip › Fig 4/4A/siSTK38_Flag-STK38 S281A_LLOMe_LAMP1.tif]

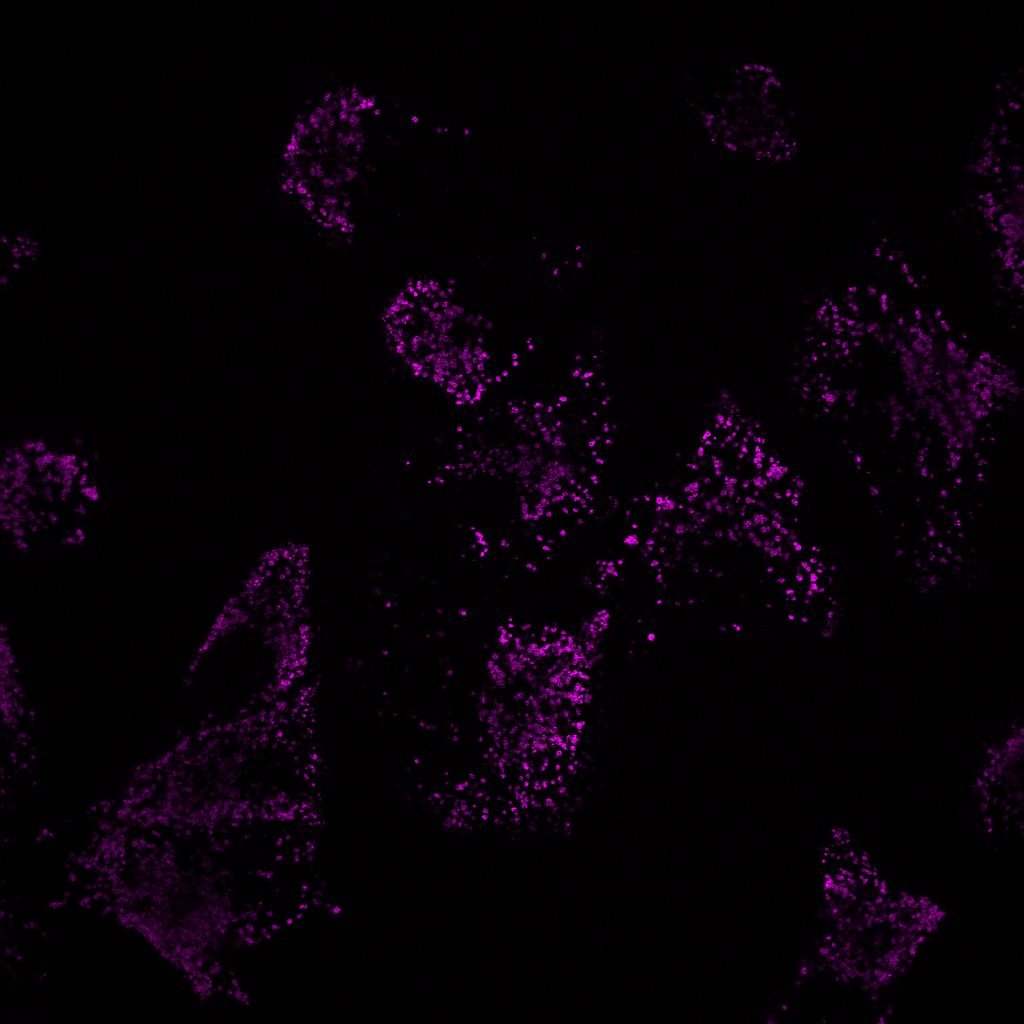

Supplement: Supplementary file 8 — Source Data for Figure 4 [file EMBR-24-e57300-s002.zip › Fig 4/4A/siSTK38_Flag-STK38 T74A_LLOMe_LAMP1.tif]

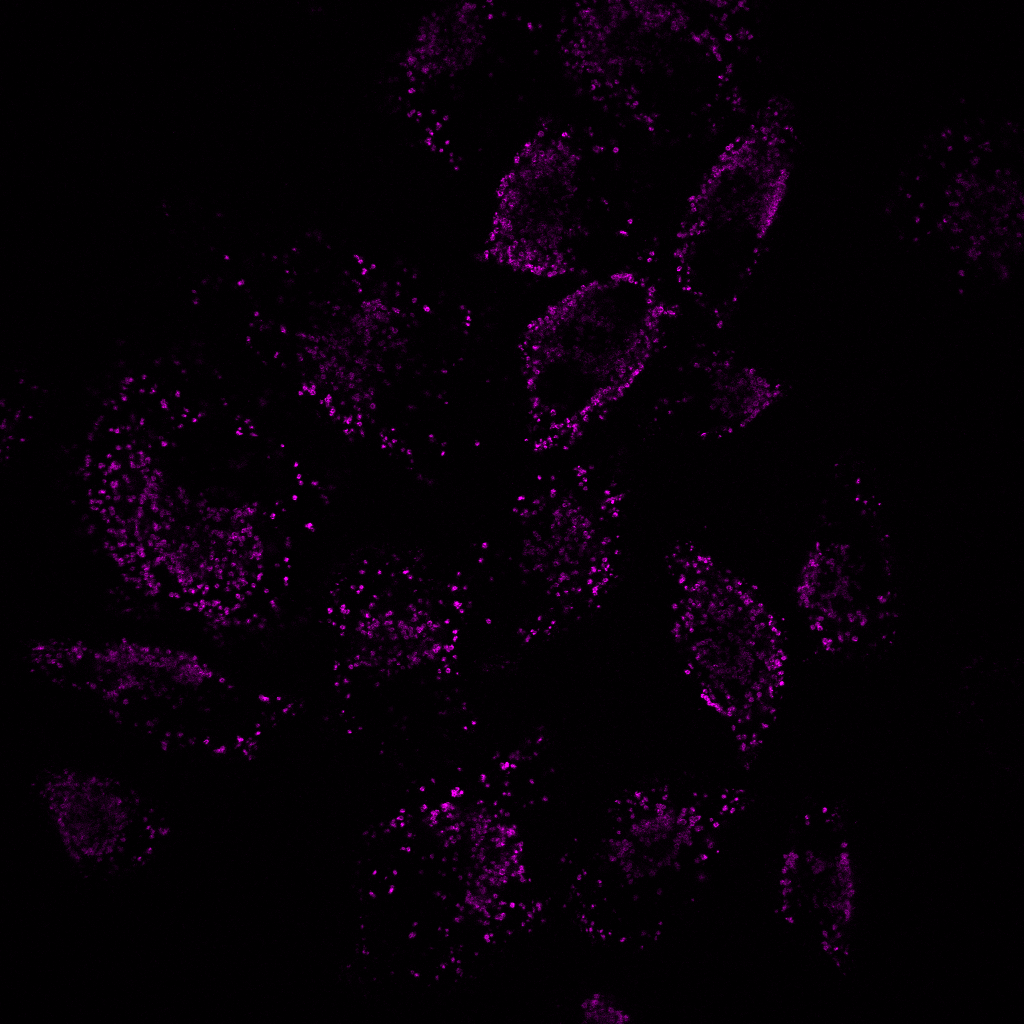

Supplement: Supplementary file 8 — Source Data for Figure 4 [file EMBR-24-e57300-s002.zip › Fig 4/4A/siSTK38_Flag-STK38 deltaC_LLOMe_LAMP1.tif]

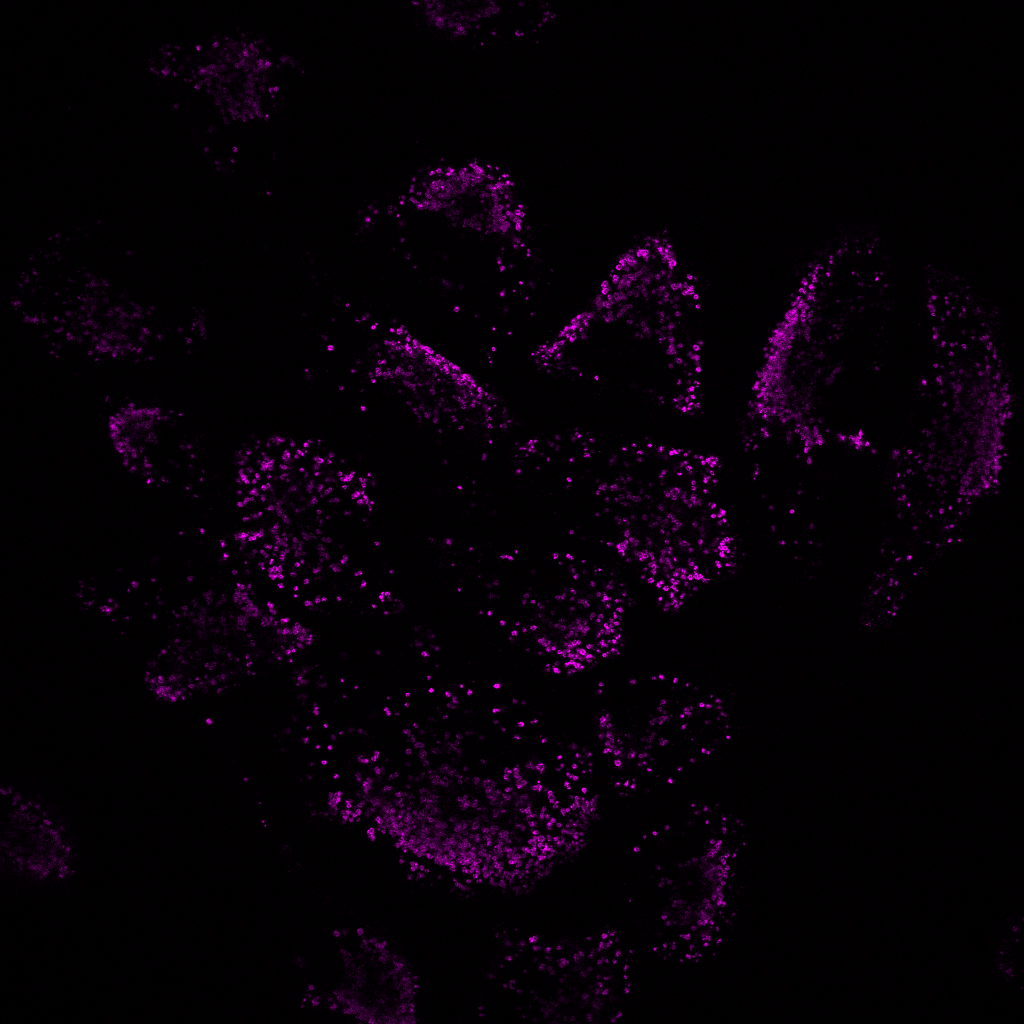

Supplement: Supplementary file 8 — Source Data for Figure 4 [file EMBR-24-e57300-s002.zip › Fig 4/4A/siSTK38_Flag-STK38 K118R_LLOMe_LAMP1.tif]

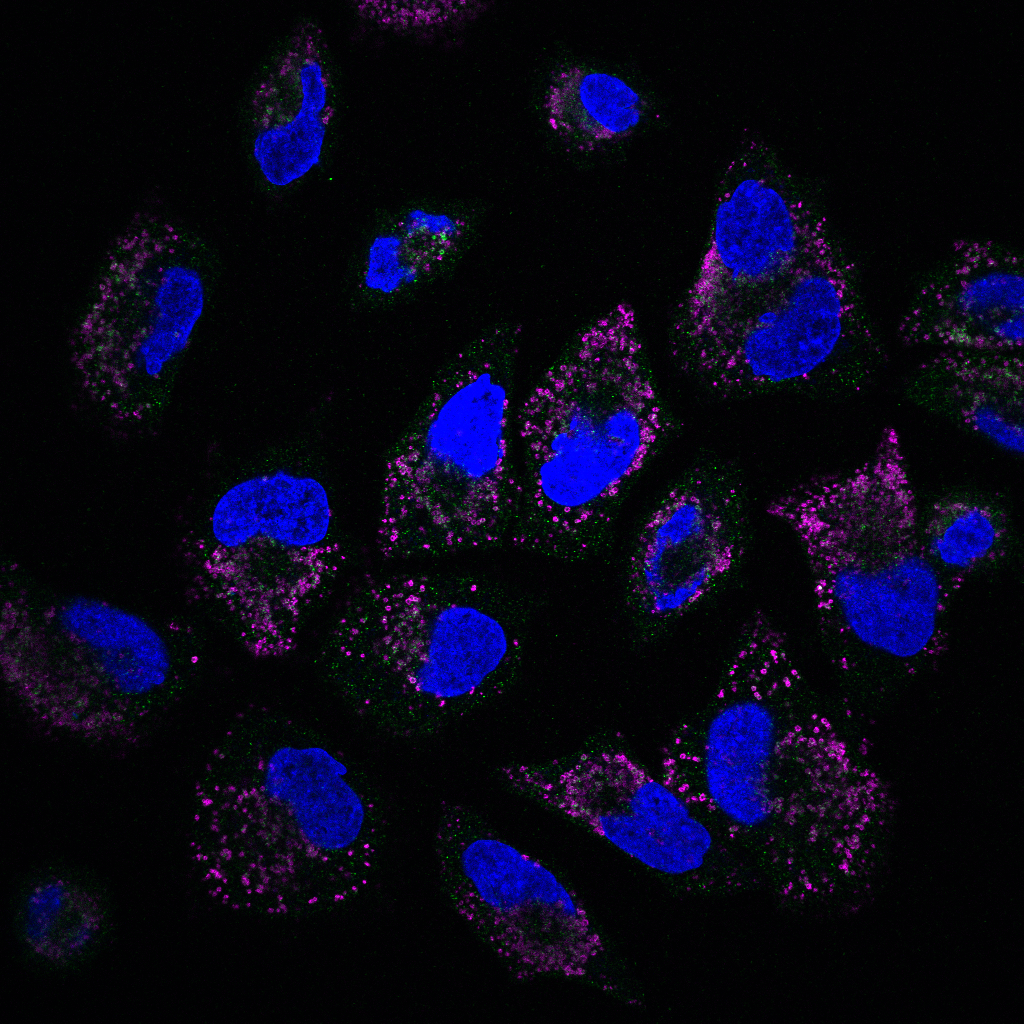

Supplement: Supplementary file 8 — Source Data for Figure 4 [file EMBR-24-e57300-s002.zip › Fig 4/4A/siSTK38_Flag-STK38 T444A_LLOMe_Merge+DAPI.tif]

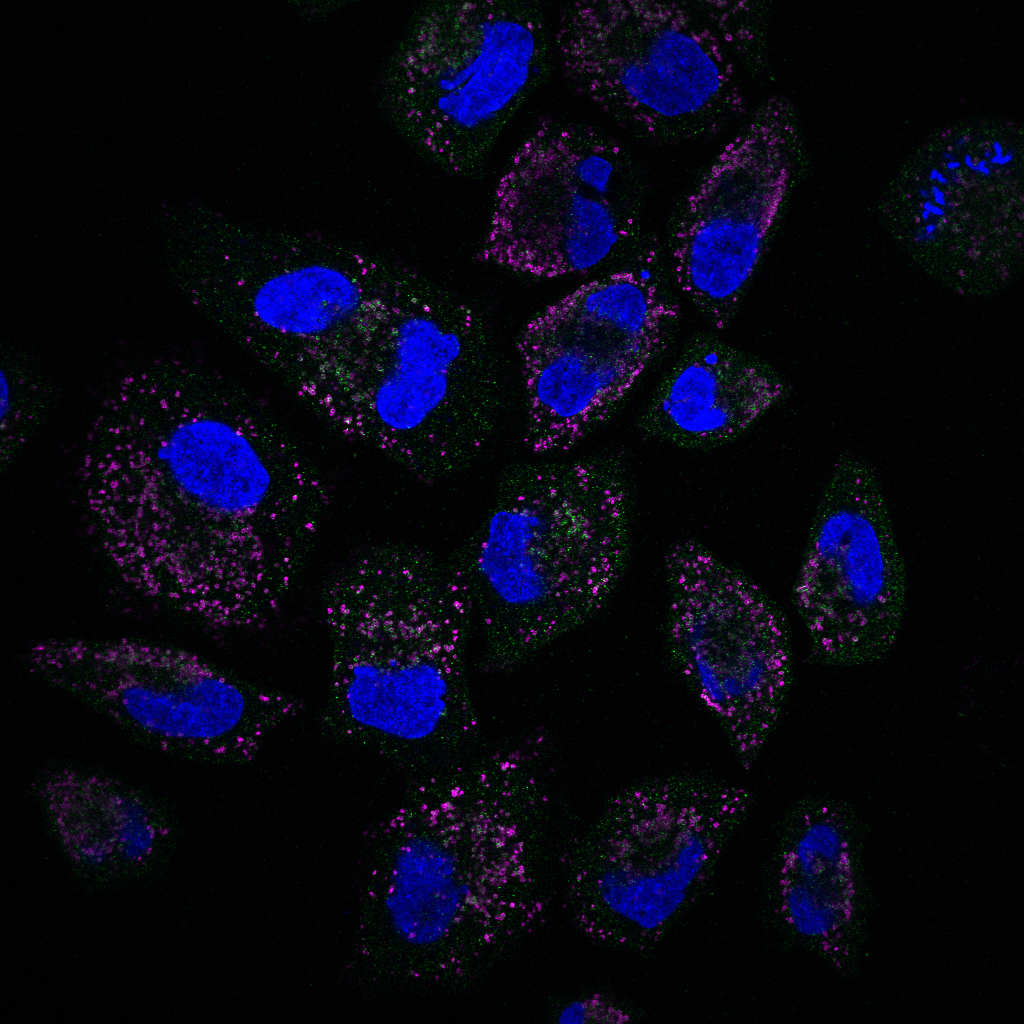

Supplement: Supplementary file 8 — Source Data for Figure 4 [file EMBR-24-e57300-s002.zip › Fig 4/4A/siSTK38_Flag-STK38 deltaC_LLOMe_Merge+DAPI.tif]

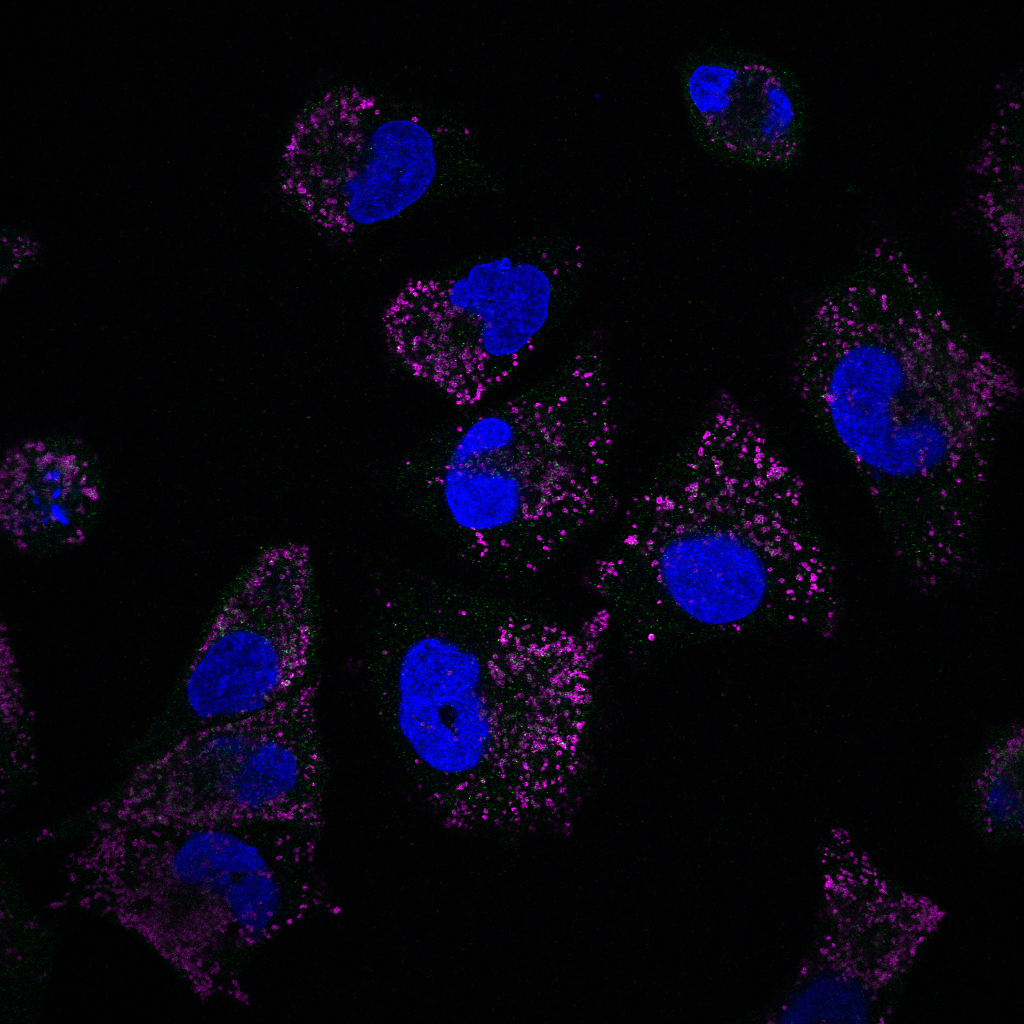

Supplement: Supplementary file 8 — Source Data for Figure 4 [file EMBR-24-e57300-s002.zip › Fig 4/4A/siSTK38_Flag-STK38 T74A_LLOMe_Merge+DAPI.tif]

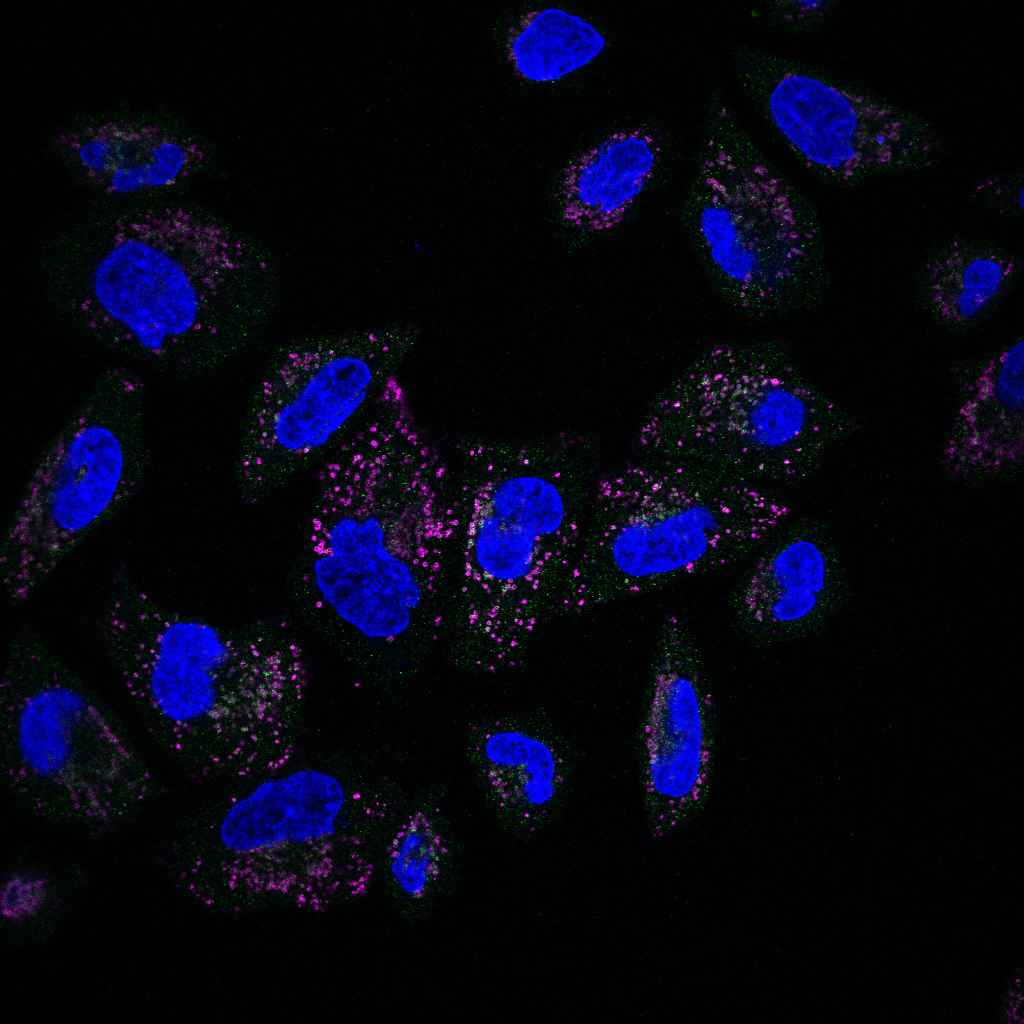

Supplement: Supplementary file 8 — Source Data for Figure 4 [file EMBR-24-e57300-s002.zip › Fig 4/4A/siSTK38_Flag-STK38 WT_LLOMe_Merge+DAPI.tif]

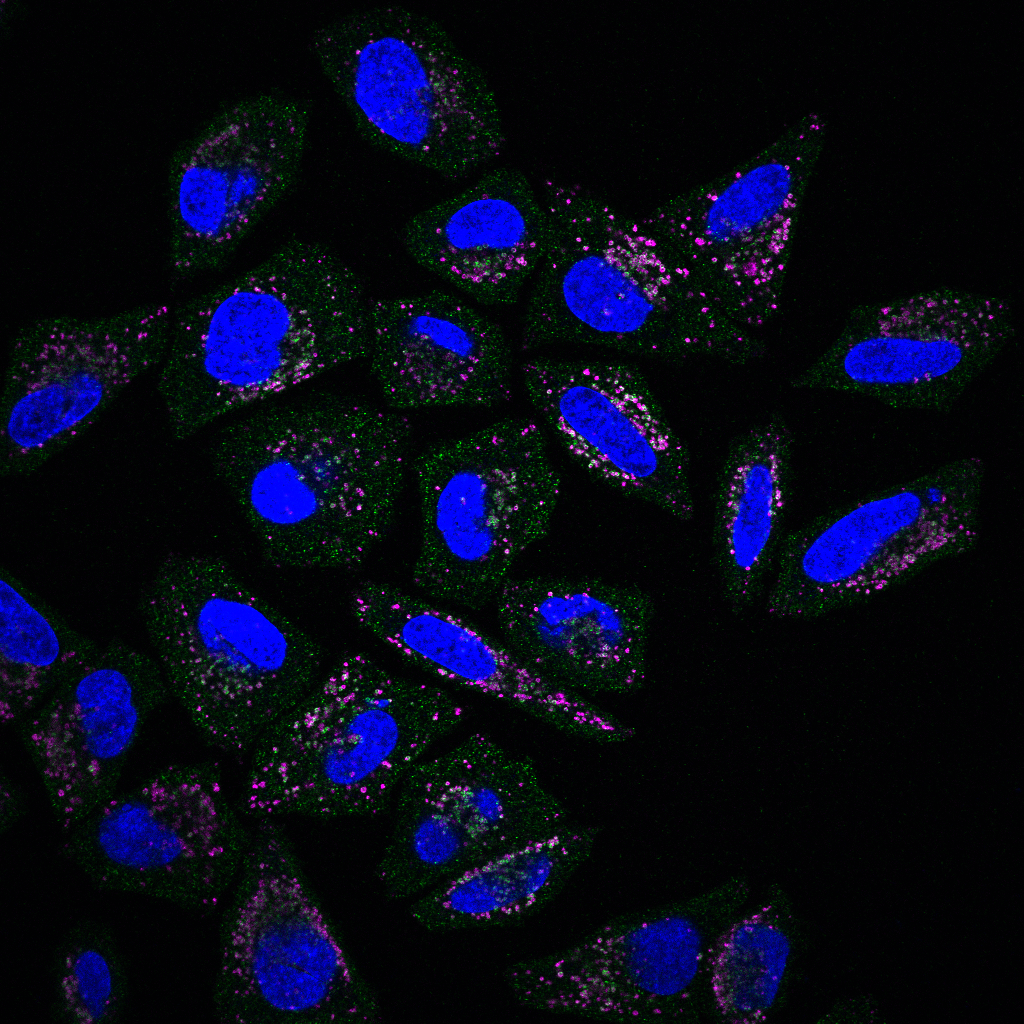

Supplement: Supplementary file 8 — Source Data for Figure 4 [file EMBR-24-e57300-s002.zip › Fig 4/4A/siLuc_Flag_LLOMe_Merge+DAPI.tif]

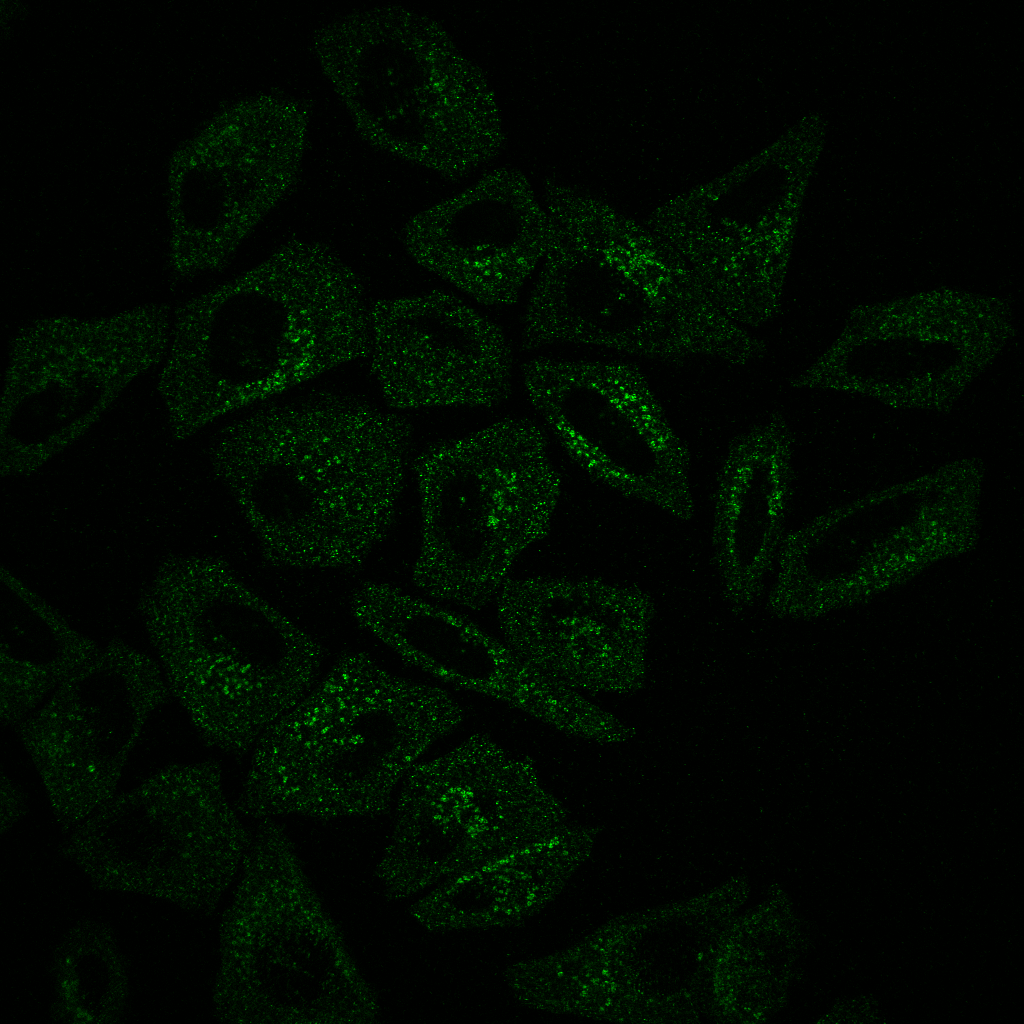

Supplement: Supplementary file 8 — Source Data for Figure 4 [file EMBR-24-e57300-s002.zip › Fig 4/4A/siLuc_Flag_LLOMe_VPS4.tif]

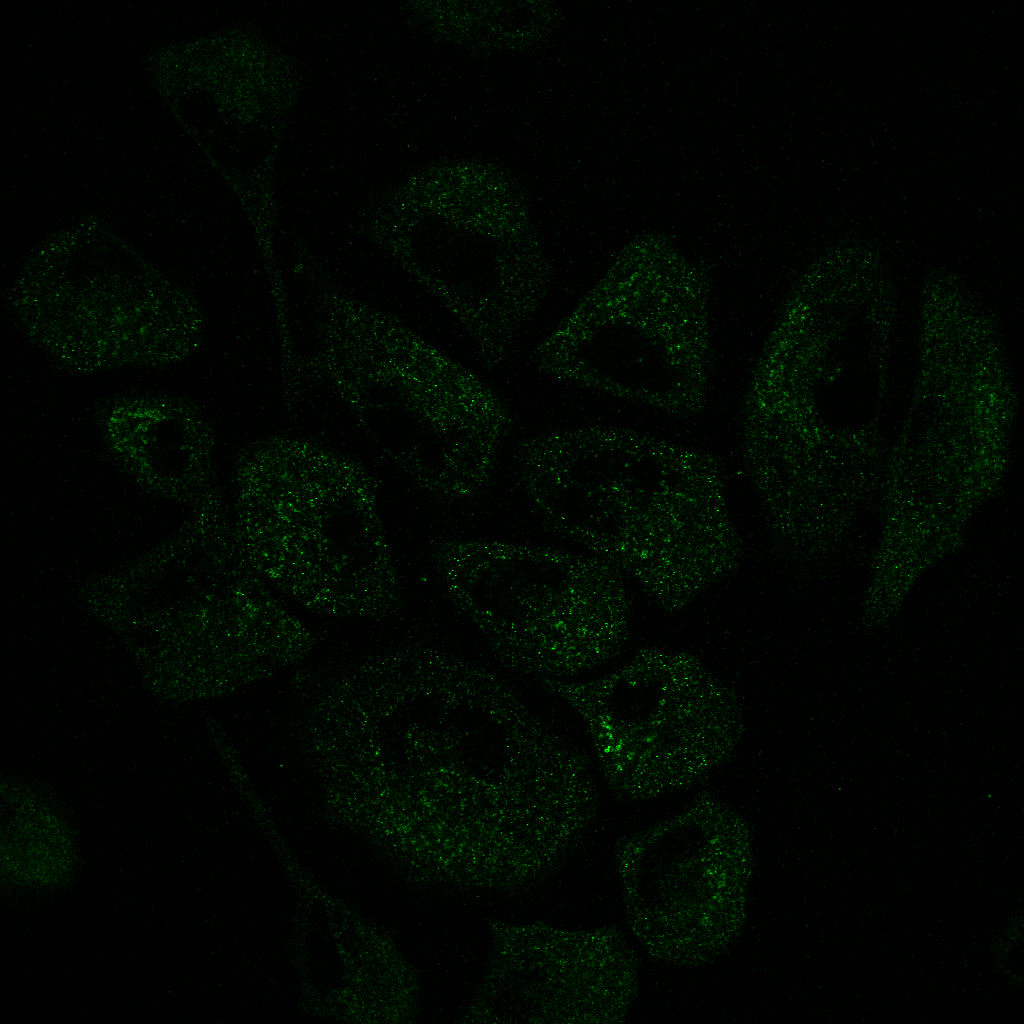

Supplement: Supplementary file 8 — Source Data for Figure 4 [file EMBR-24-e57300-s002.zip › Fig 4/4A/siSTK38_Flag-STK38 K118R_LLOMe_VPS4.tif]

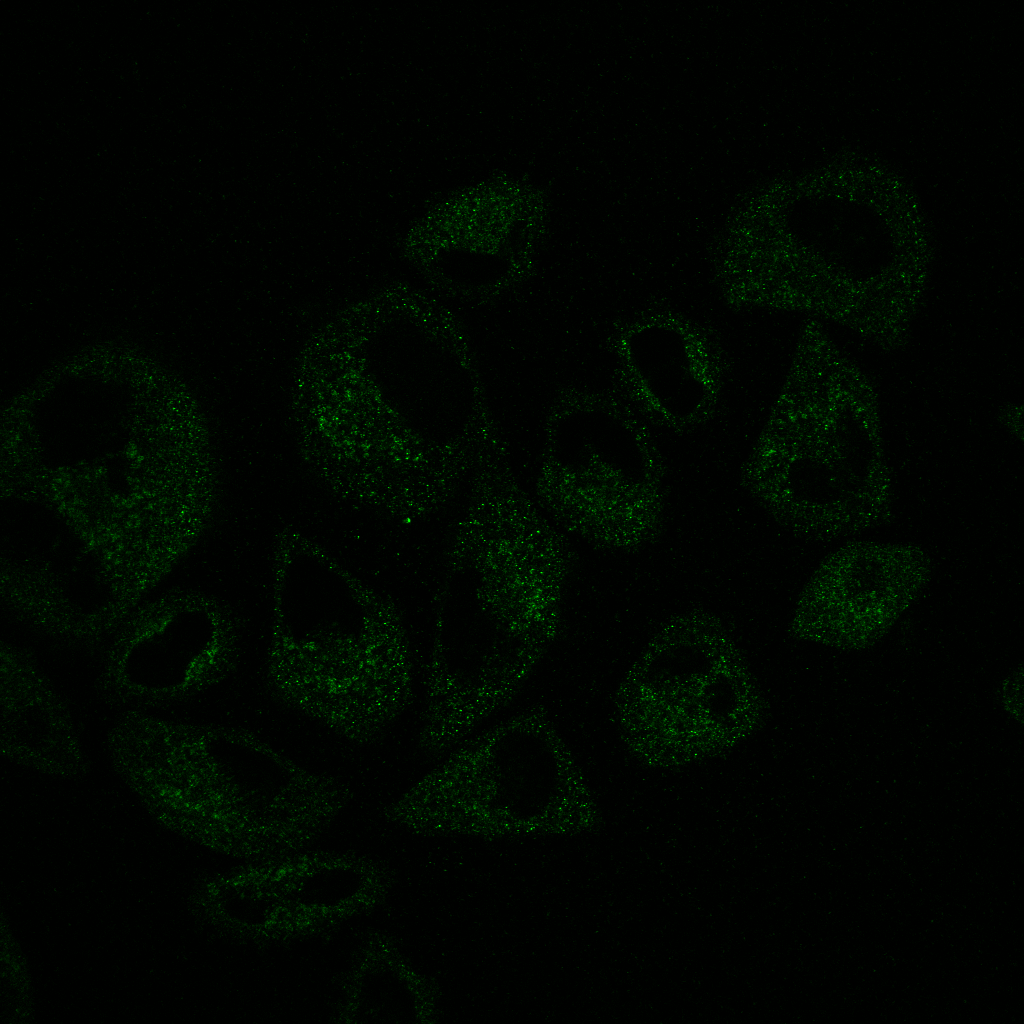

Supplement: Supplementary file 8 — Source Data for Figure 4 [file EMBR-24-e57300-s002.zip › Fig 4/4A/siSTK38_Flag_LLOMe_VPS4.tif]

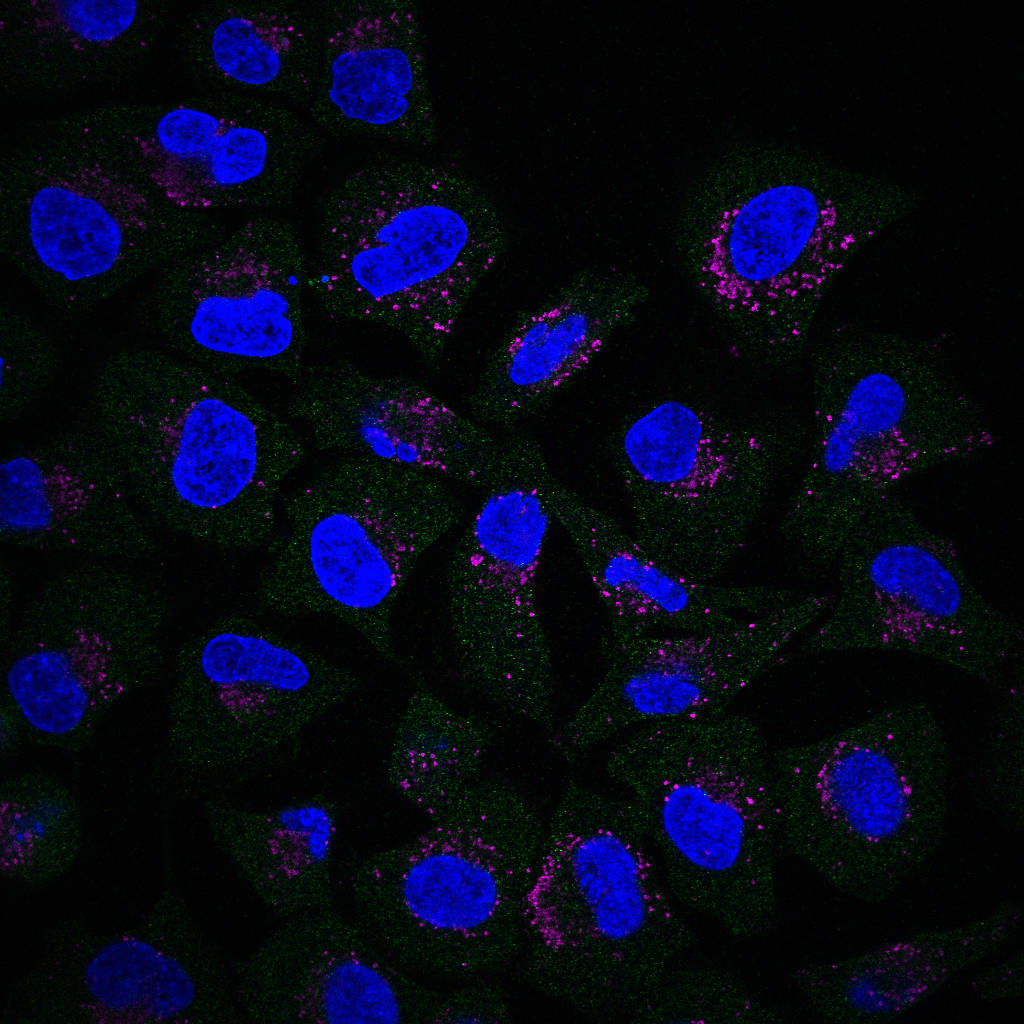

Supplement: Supplementary file 8 — Source Data for Figure 4 [file EMBR-24-e57300-s002.zip › Fig 4/4A/siLuc_Flag_non-treated_Merge+DAPI.tif]

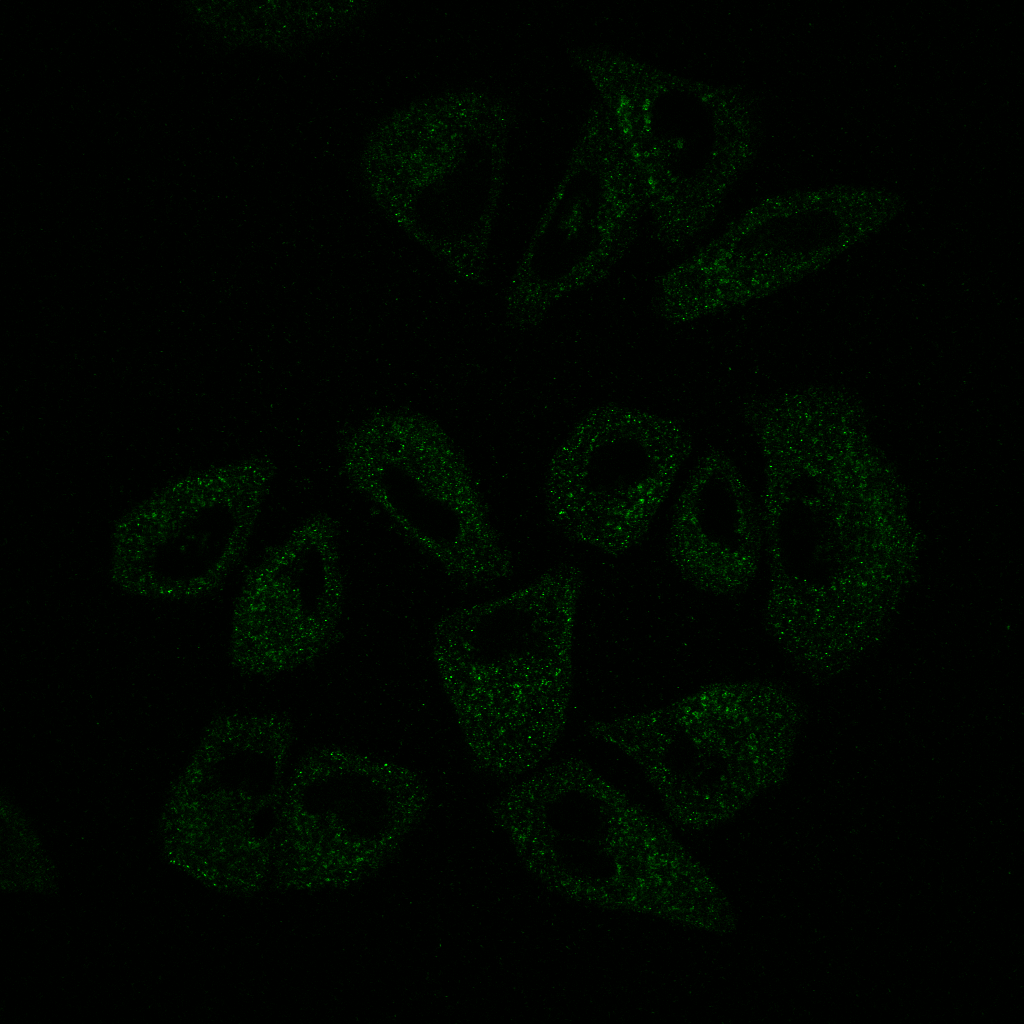

Supplement: Supplementary file 8 — Source Data for Figure 4 [file EMBR-24-e57300-s002.zip › Fig 4/4A/siSTK38_Flag-STK38 deltaN_LLOMe_VPS4.tif]

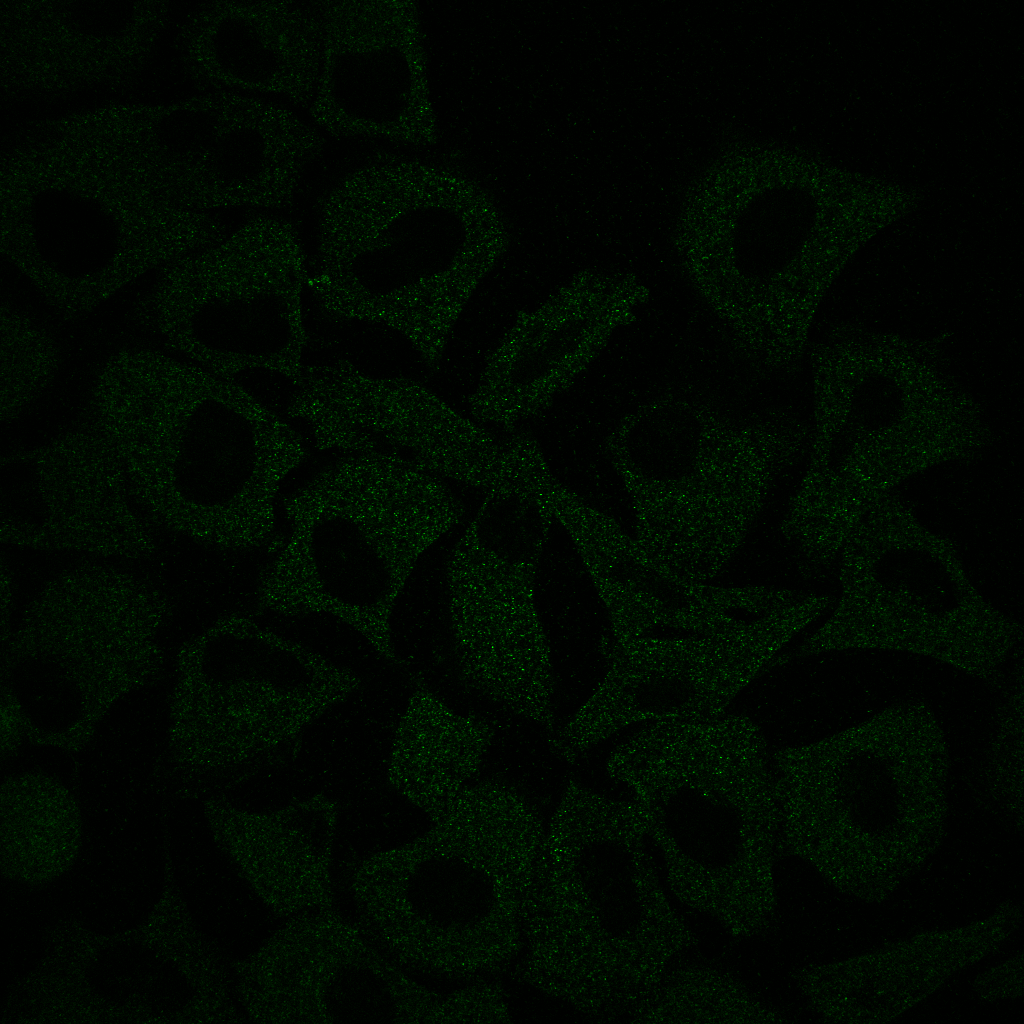

Supplement: Supplementary file 8 — Source Data for Figure 4 [file EMBR-24-e57300-s002.zip › Fig 4/4A/siLuc_Flag_non-treated_VPS4.tif]

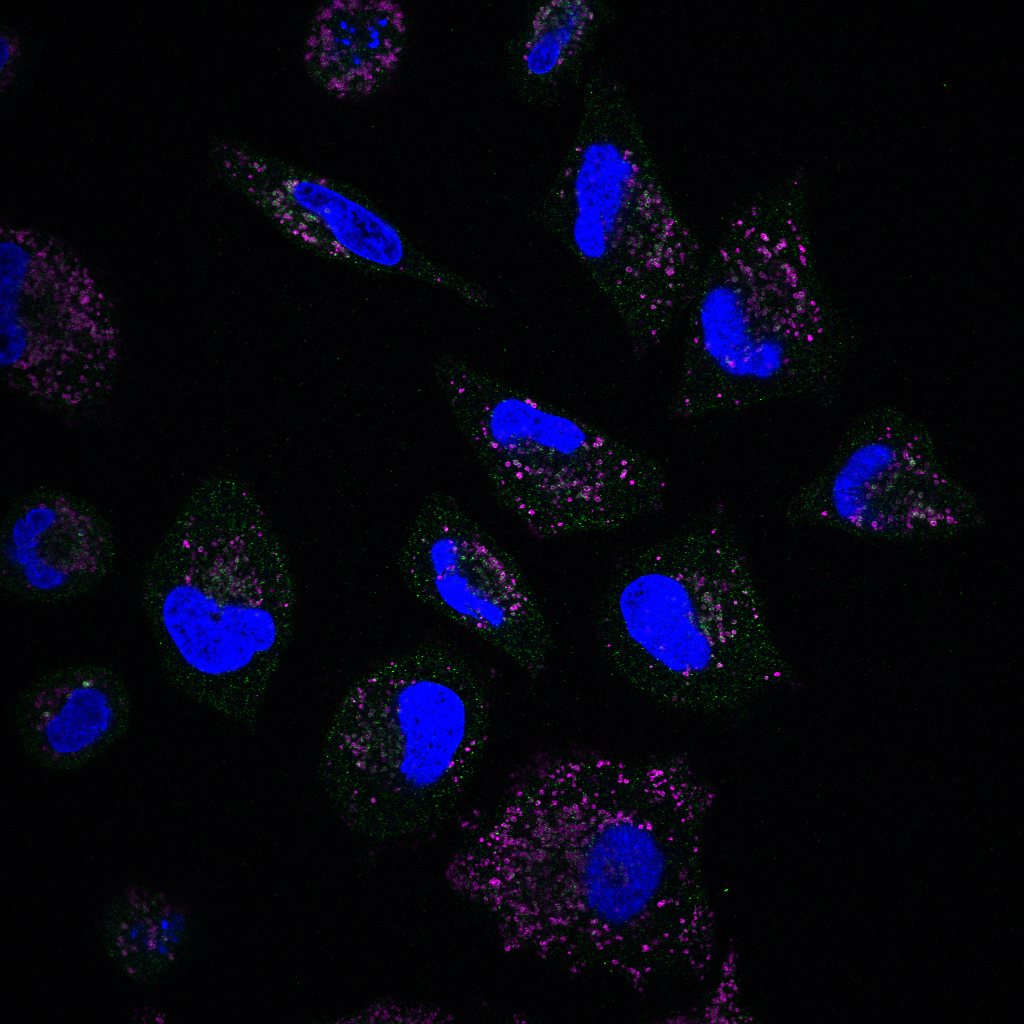

Supplement: Supplementary file 8 — Source Data for Figure 4 [file EMBR-24-e57300-s002.zip › Fig 4/4A/siSTK38_Flag-STK38 S281A_LLOMe_Merge+DAPI.tif]

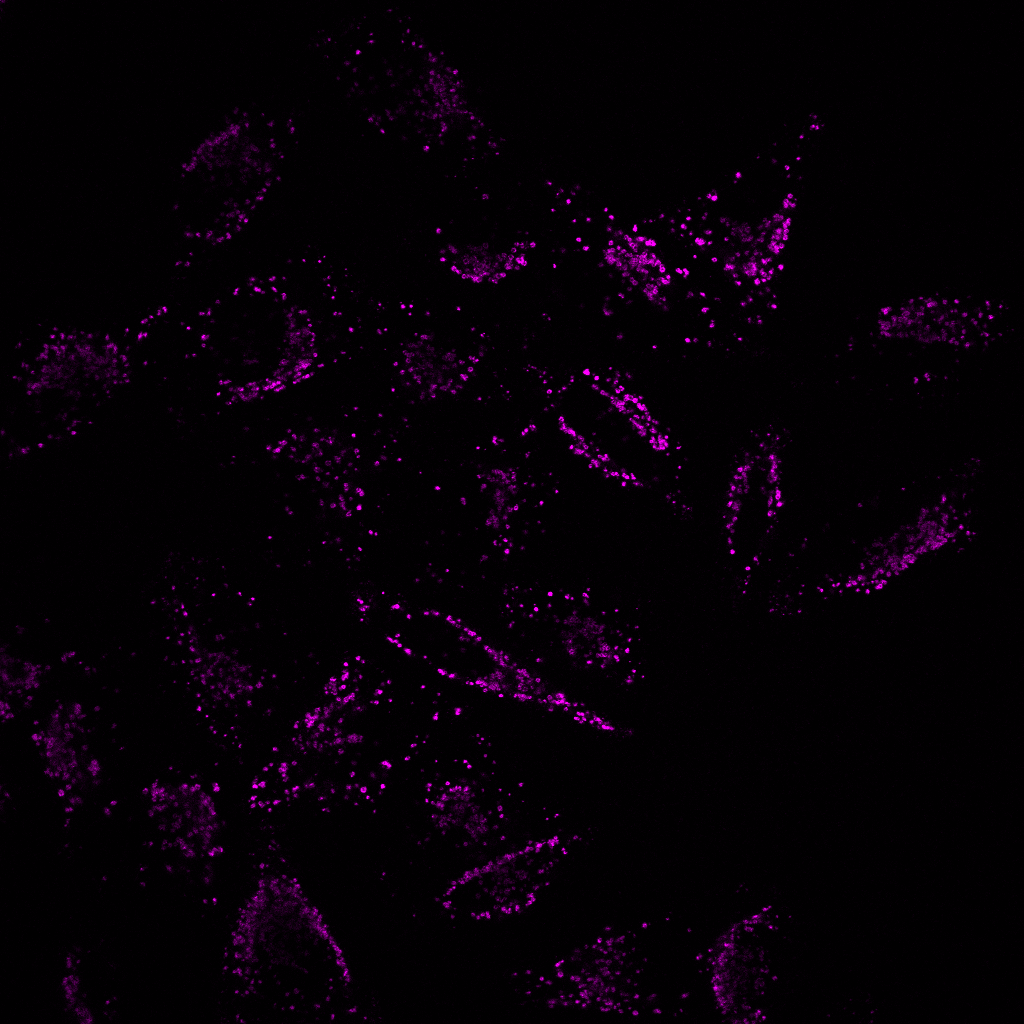

Supplement: Supplementary file 8 — Source Data for Figure 4 [file EMBR-24-e57300-s002.zip › Fig 4/4A/siLuc_Flag_LLOMe_LAMP1.tif]

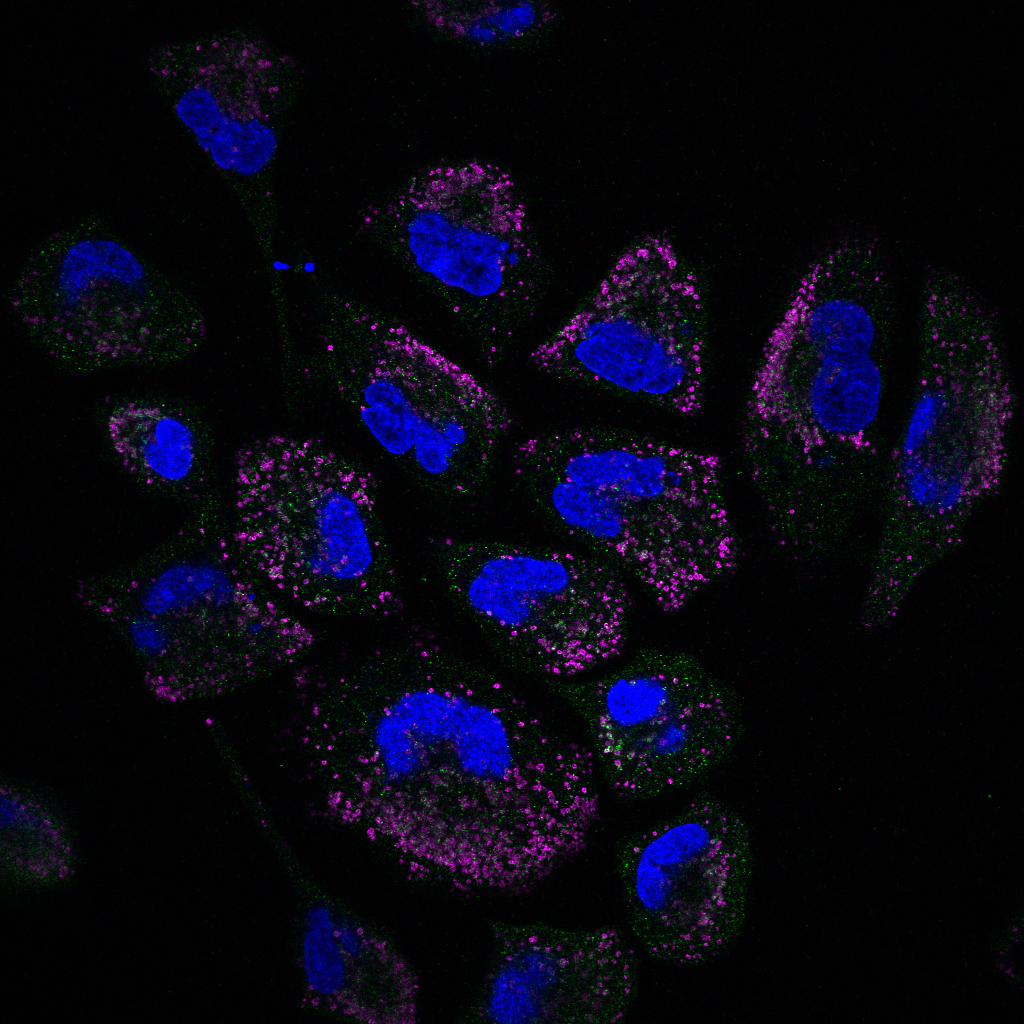

Supplement: Supplementary file 8 — Source Data for Figure 4 [file EMBR-24-e57300-s002.zip › Fig 4/4A/siSTK38_Flag-STK38 K118R_LLOMe_Merge+DAPI.tif]

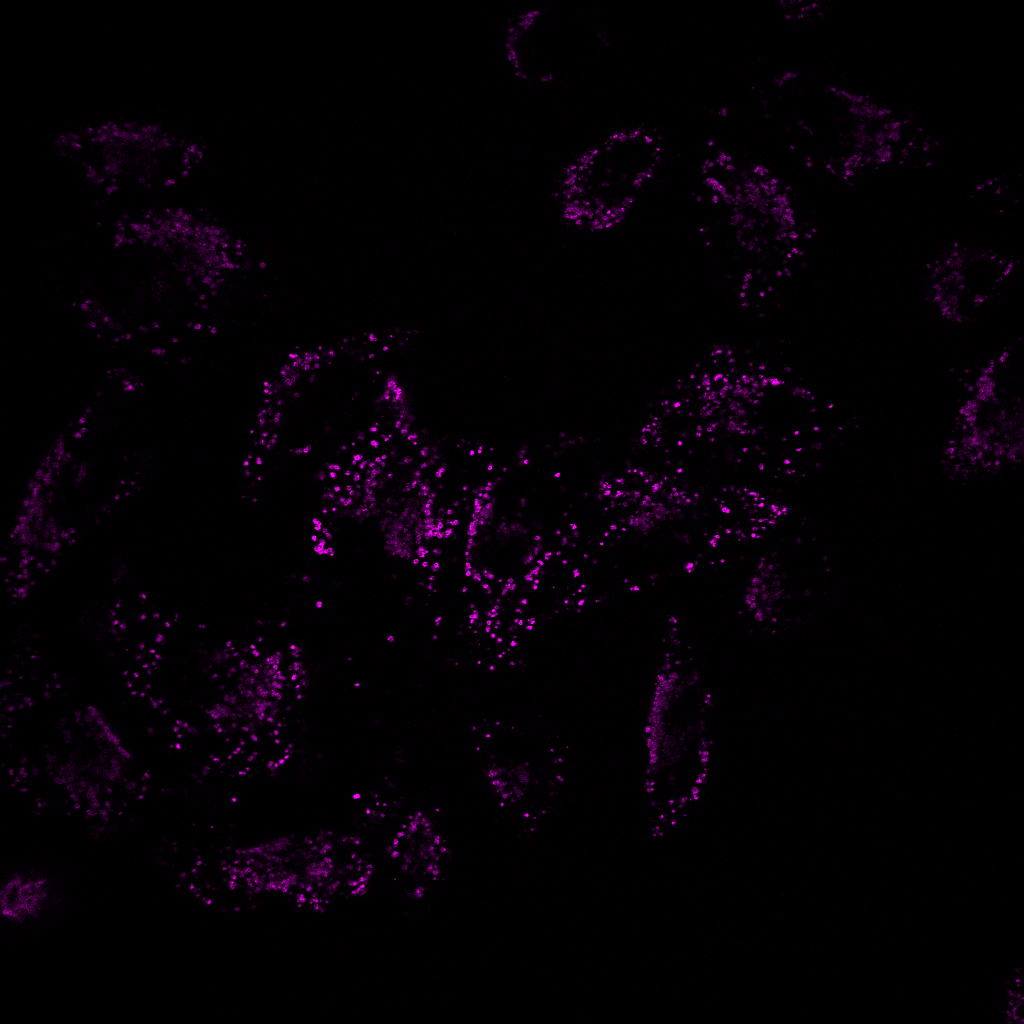

Supplement: Supplementary file 8 — Source Data for Figure 4 [file EMBR-24-e57300-s002.zip › Fig 4/4A/siSTK38_Flag-STK38 WT_LLOMe_LAMP1.tif]
