# Supplementary material for: Microautophagy regulated by STK38 and GABARAPs is essential to repair lysosomes and prevent aging
Source: EMBO Rep. 2023 Nov 21;24(12):e57300. doi: 10.15252/embr.202357300 (PMC10702834; doi:10.15252/embr.202357300)
Supplement: Supplementary file 7 — Source Data for Figure 3 [file EMBR-24-e57300-s005.zip › Fig 3/3J/3J_README.rtf]

non-treated or NH4Cl_LAMP1-GFP are unprocessed fluorescent images.To make CLEM images, these fluorescent images were flipped horizontally, rotated, and merged with EM images.
