# Supplementary figures and images for: Microautophagy regulated by STK38 and GABARAPs is essential to repair lysosomes and prevent aging (part 3 of 4)
Source: EMBO Rep. 2023 Nov 21;24(12):e57300. doi: 10.15252/embr.202357300 (PMC10702834; doi:10.15252/embr.202357300)

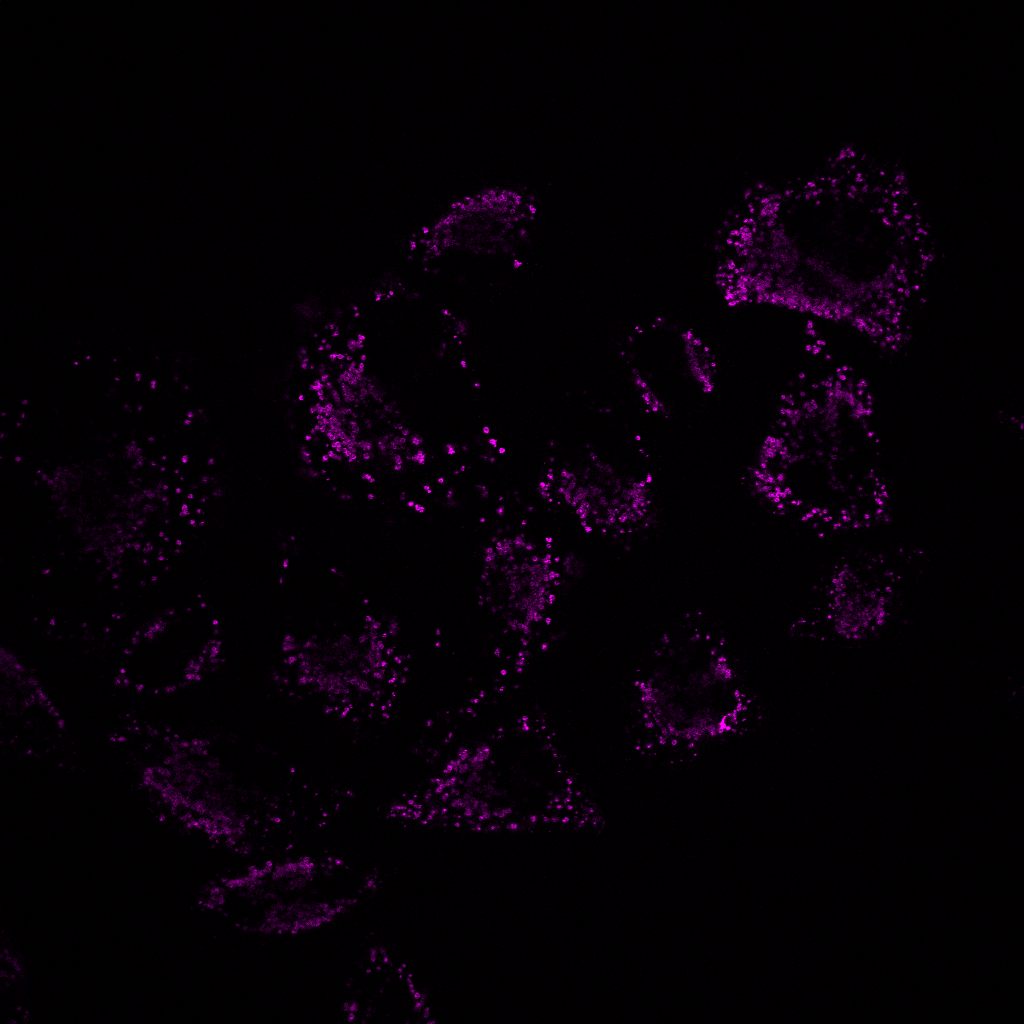

Supplement: Supplementary file 8 — Source Data for Figure 4 [file EMBR-24-e57300-s002.zip › Fig 4/4A/siSTK38_Flag_LLOMe_LAMP1.tif]

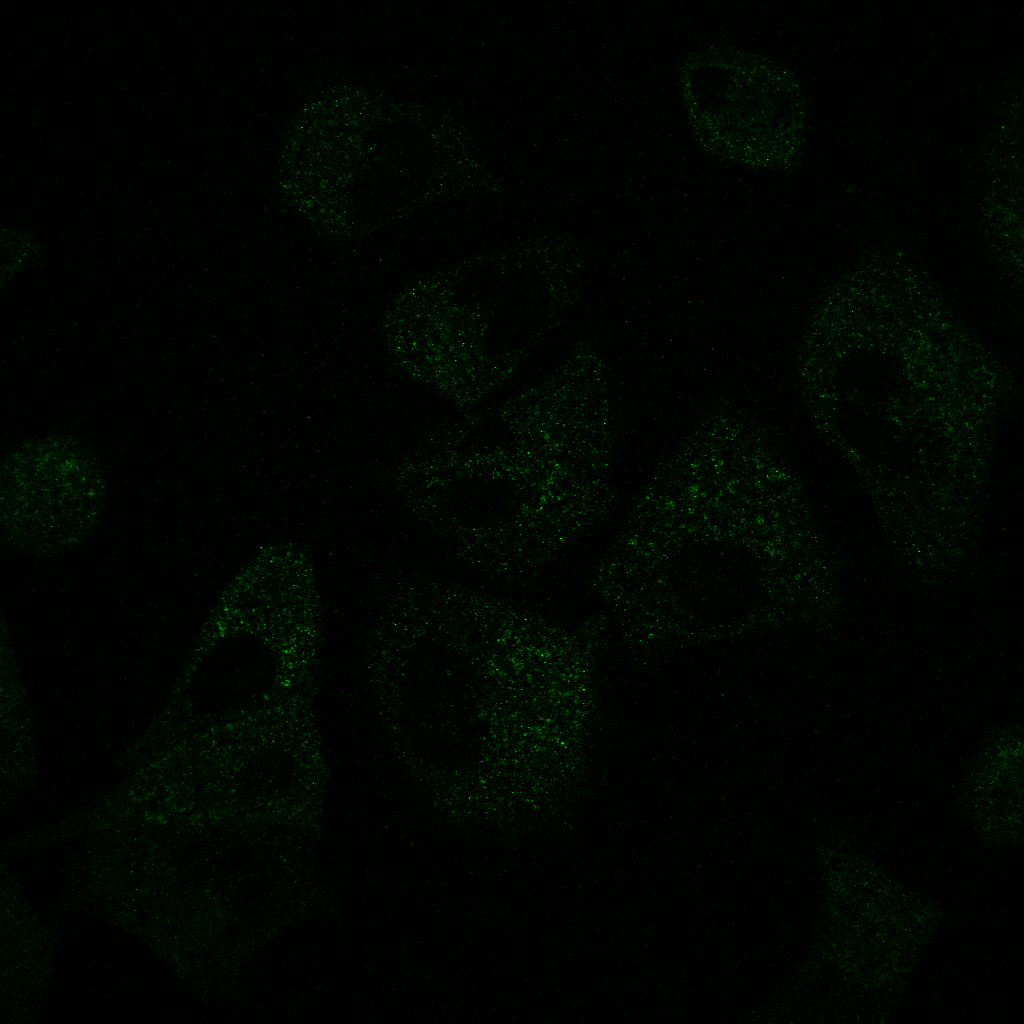

Supplement: Supplementary file 8 — Source Data for Figure 4 [file EMBR-24-e57300-s002.zip › Fig 4/4A/siSTK38_Flag-STK38 T74A_LLOMe_VPS4.tif]

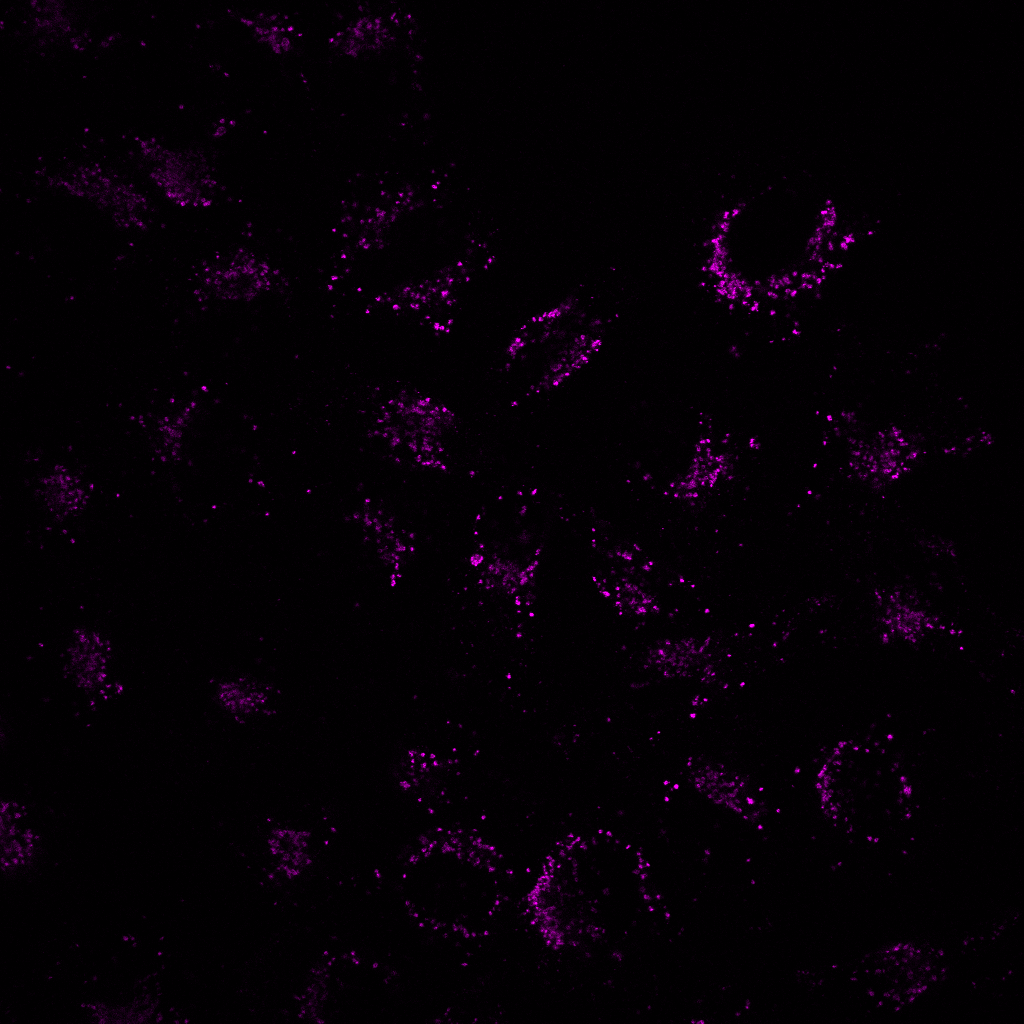

Supplement: Supplementary file 8 — Source Data for Figure 4 [file EMBR-24-e57300-s002.zip › Fig 4/4A/siLuc_Flag_non-treated_LAMP1.tif]

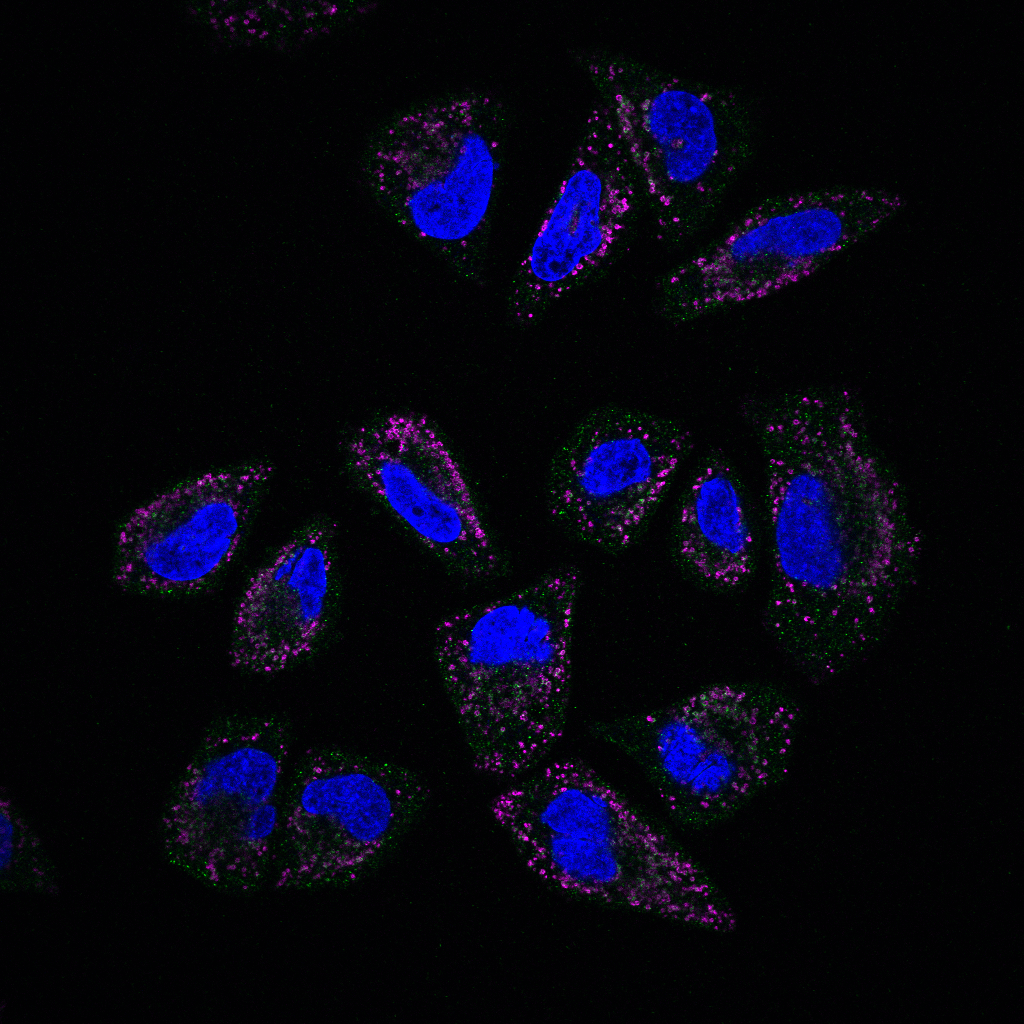

Supplement: Supplementary file 8 — Source Data for Figure 4 [file EMBR-24-e57300-s002.zip › Fig 4/4A/siSTK38_Flag-STK38 deltaN_LLOMe_Merge+DAPI.tif]

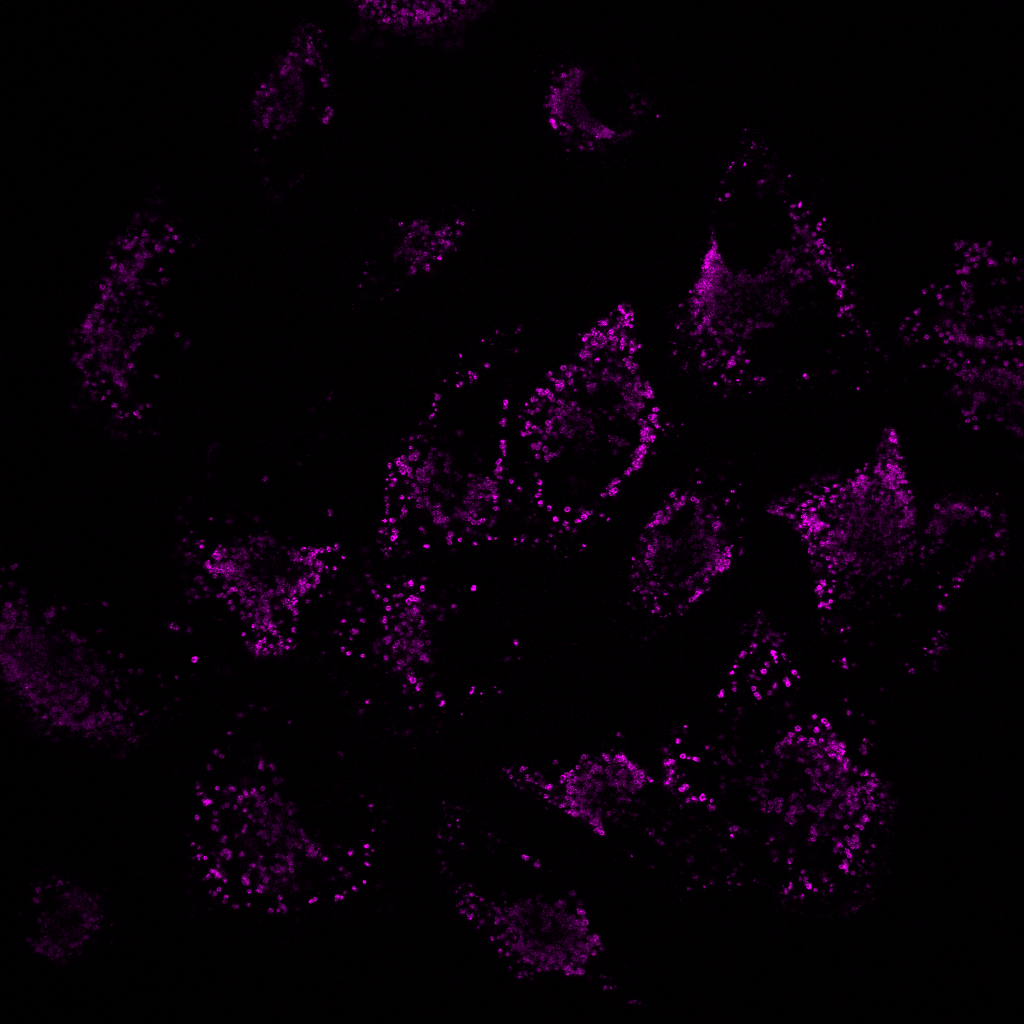

Supplement: Supplementary file 8 — Source Data for Figure 4 [file EMBR-24-e57300-s002.zip › Fig 4/4A/siSTK38_Flag-STK38 T444A_LLOMe_LAMP1.tif]

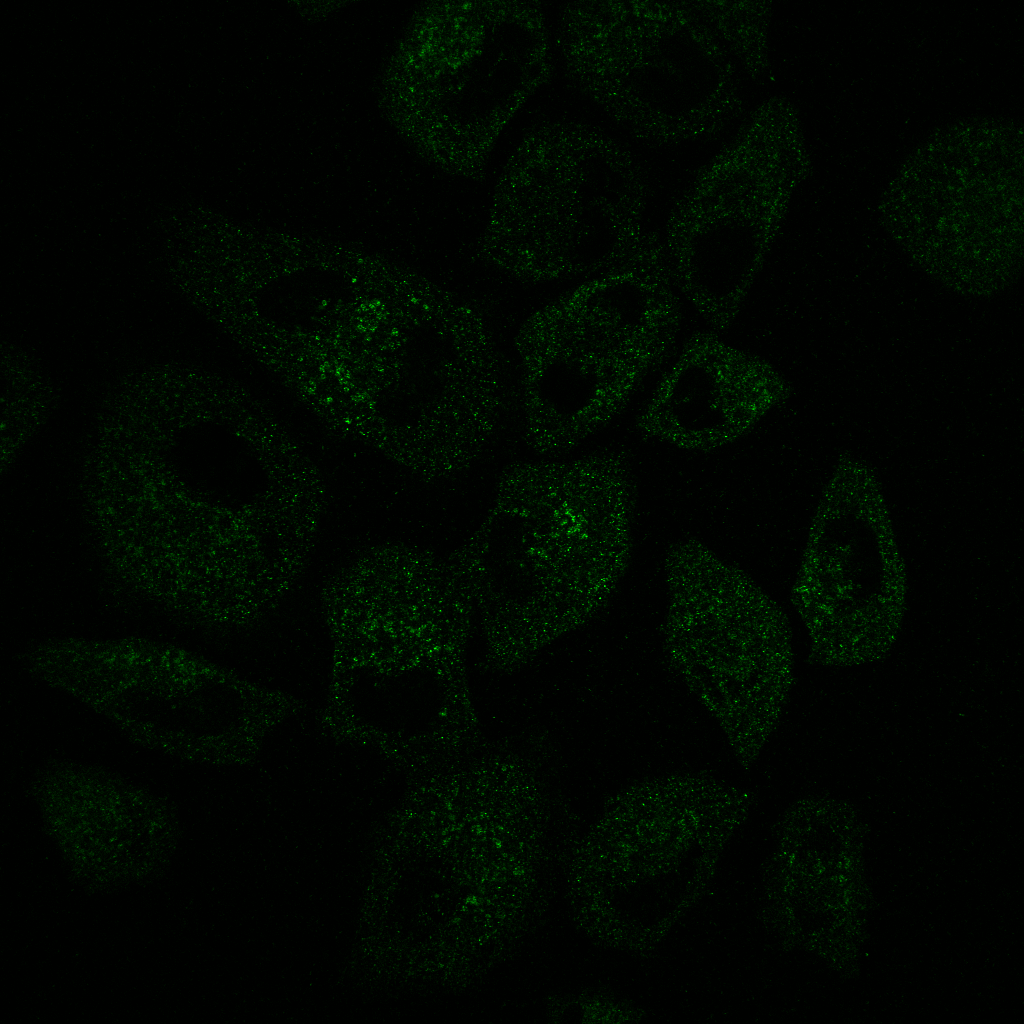

Supplement: Supplementary file 8 — Source Data for Figure 4 [file EMBR-24-e57300-s002.zip › Fig 4/4A/siSTK38_Flag-STK38 deltaC_LLOMe_VPS4.tif]

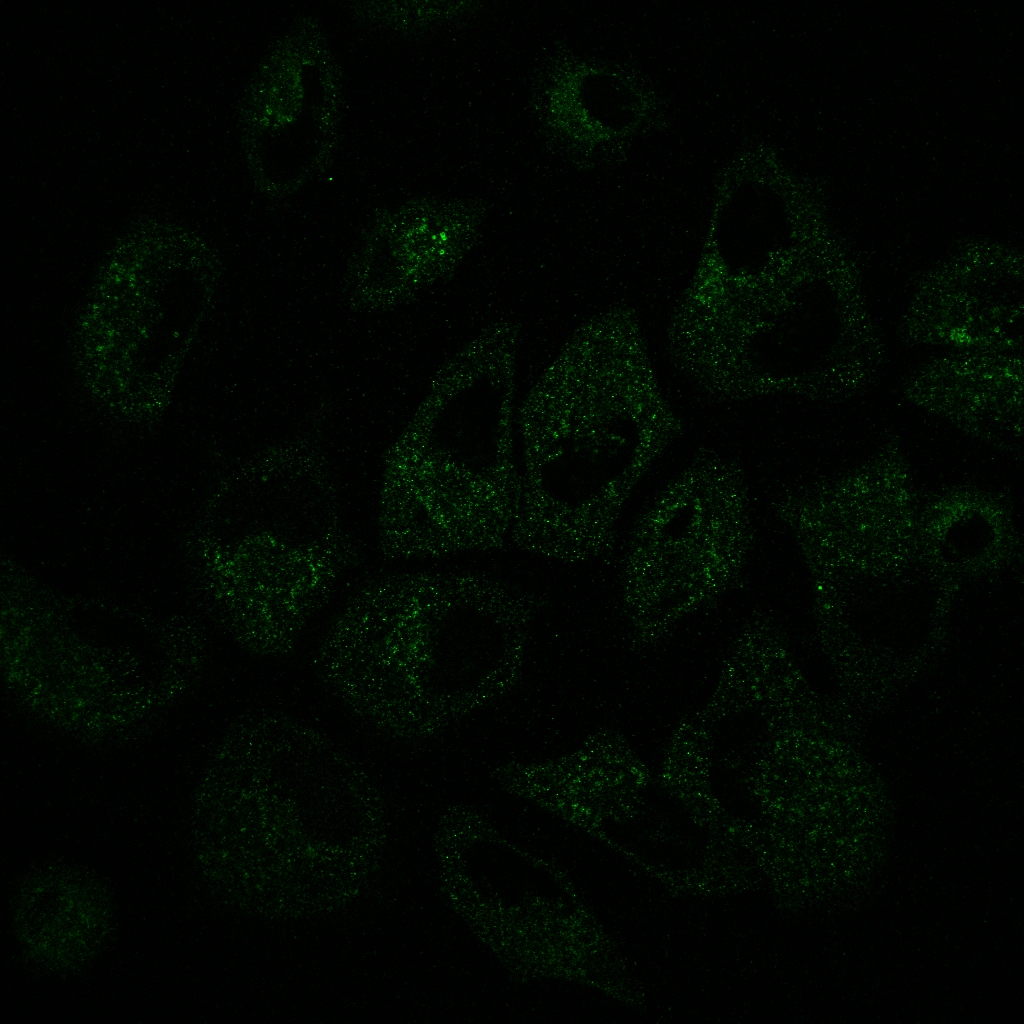

Supplement: Supplementary file 8 — Source Data for Figure 4 [file EMBR-24-e57300-s002.zip › Fig 4/4A/siSTK38_Flag-STK38 T444A_LLOMe_VPS4.tif]

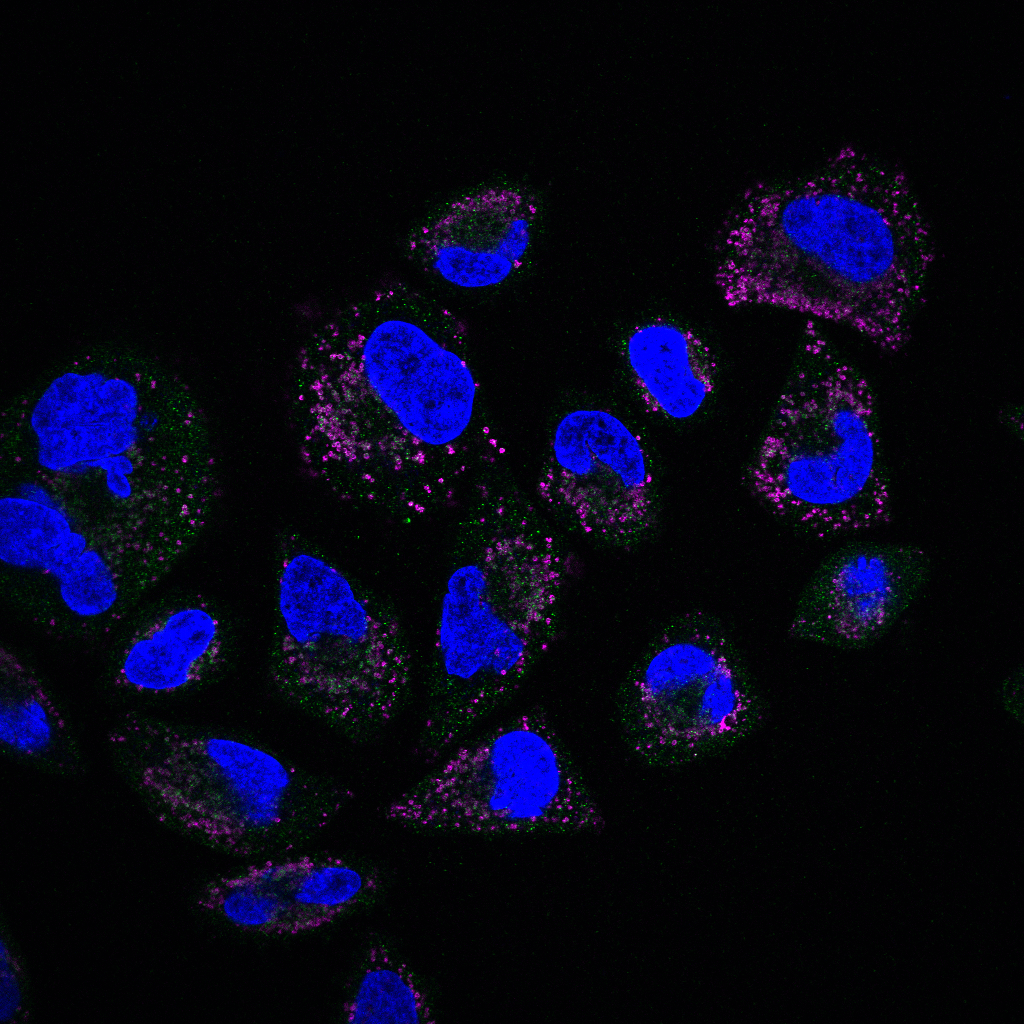

Supplement: Supplementary file 8 — Source Data for Figure 4 [file EMBR-24-e57300-s002.zip › Fig 4/4A/siSTK38_Flag_LLOMe_Merge+DAPI.tif]

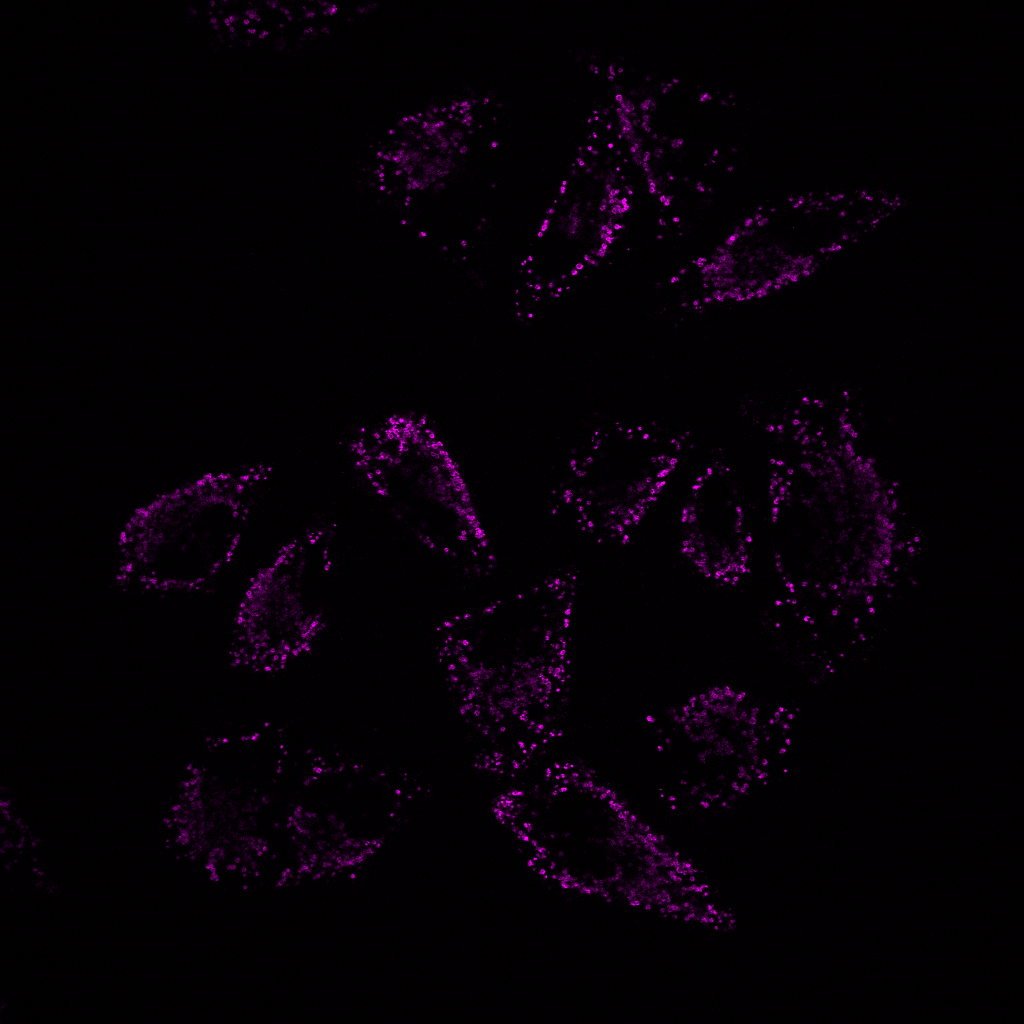

Supplement: Supplementary file 8 — Source Data for Figure 4 [file EMBR-24-e57300-s002.zip › Fig 4/4A/siSTK38_Flag-STK38 deltaN_LLOMe_LAMP1.tif]

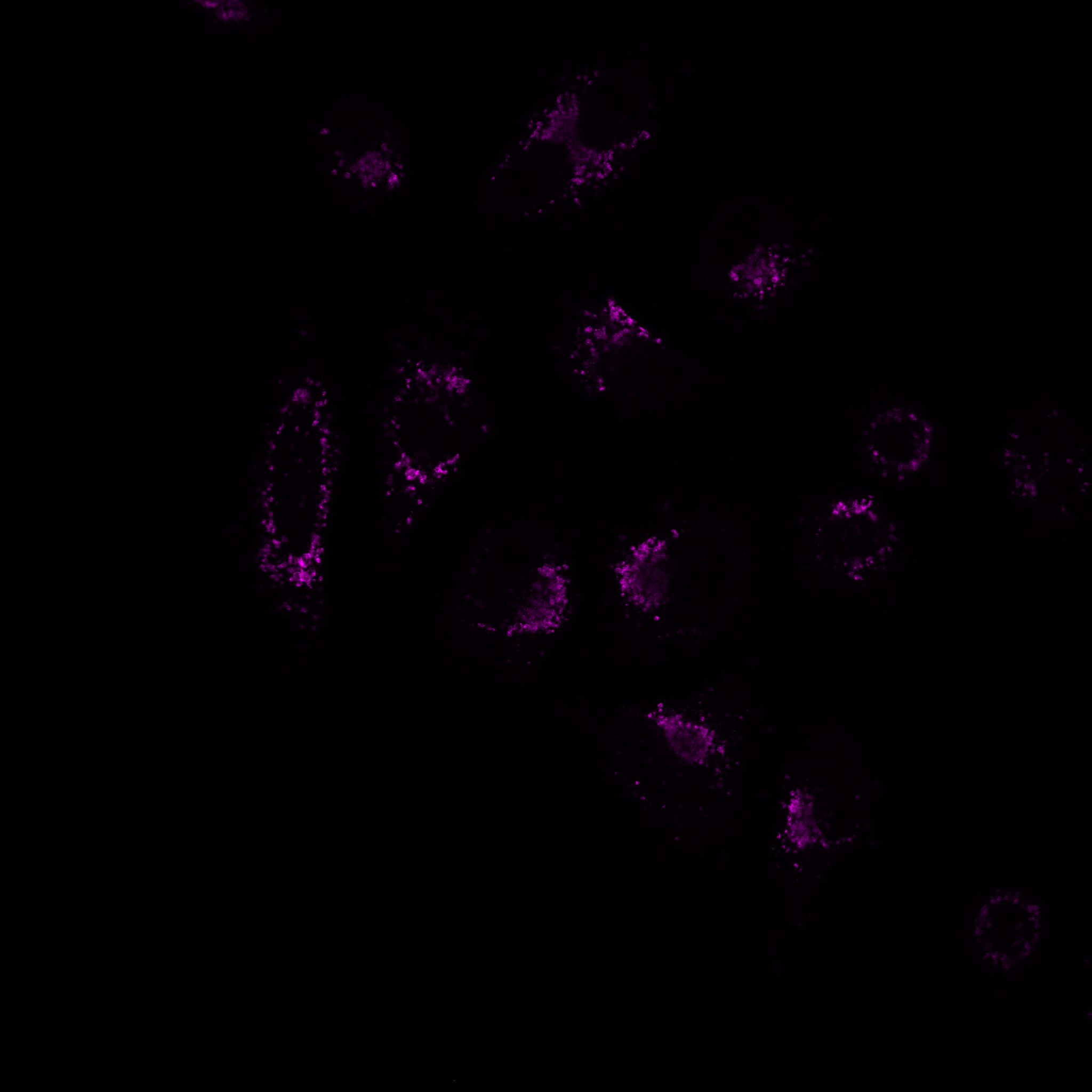

Supplement: Supplementary file 8 — Source Data for Figure 4 [file EMBR-24-e57300-s002.zip › Fig 4/4G/non-treated_LAMP1.tif]

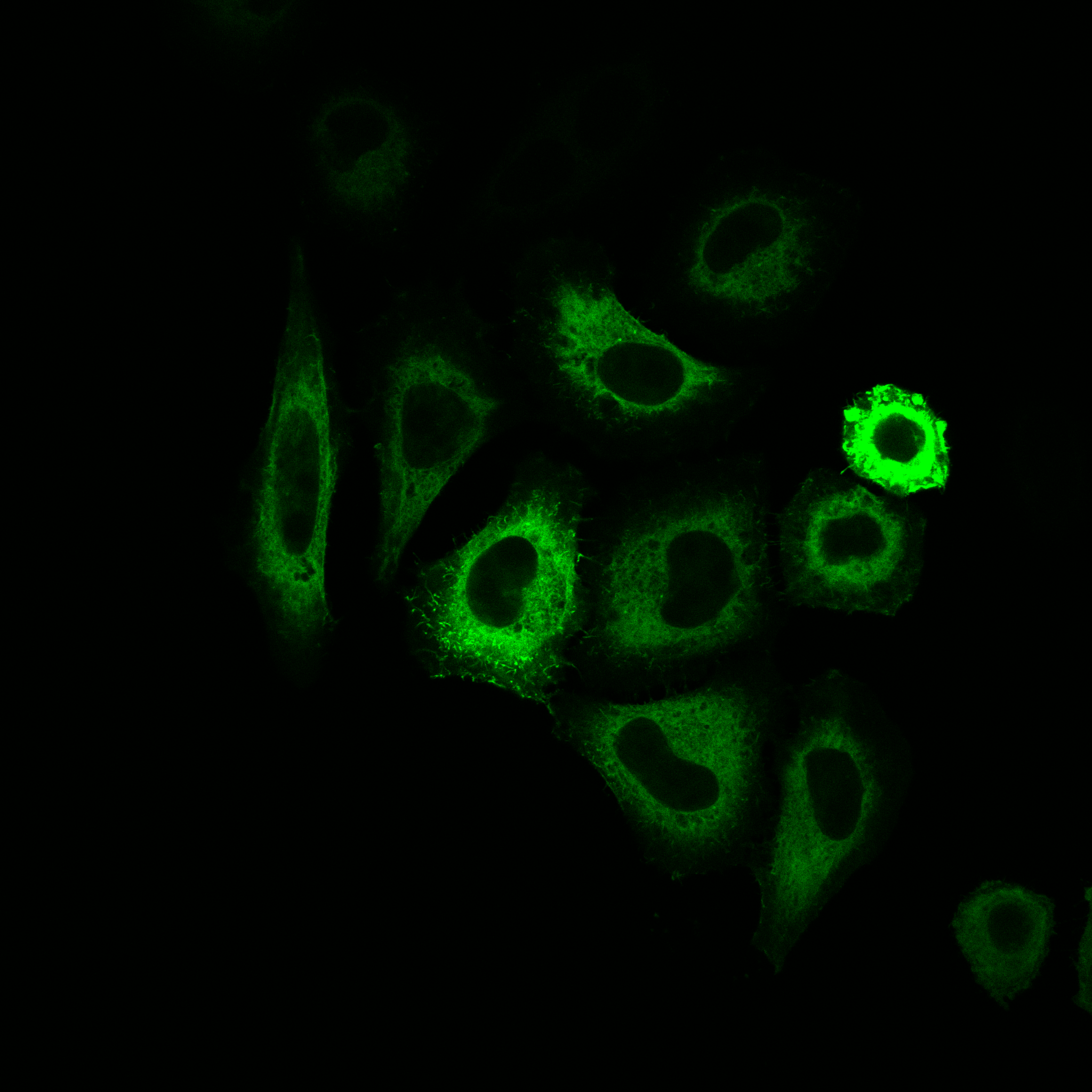

Supplement: Supplementary file 8 — Source Data for Figure 4 [file EMBR-24-e57300-s002.zip › Fig 4/4G/non-treated_mNG-DOK1.tif]

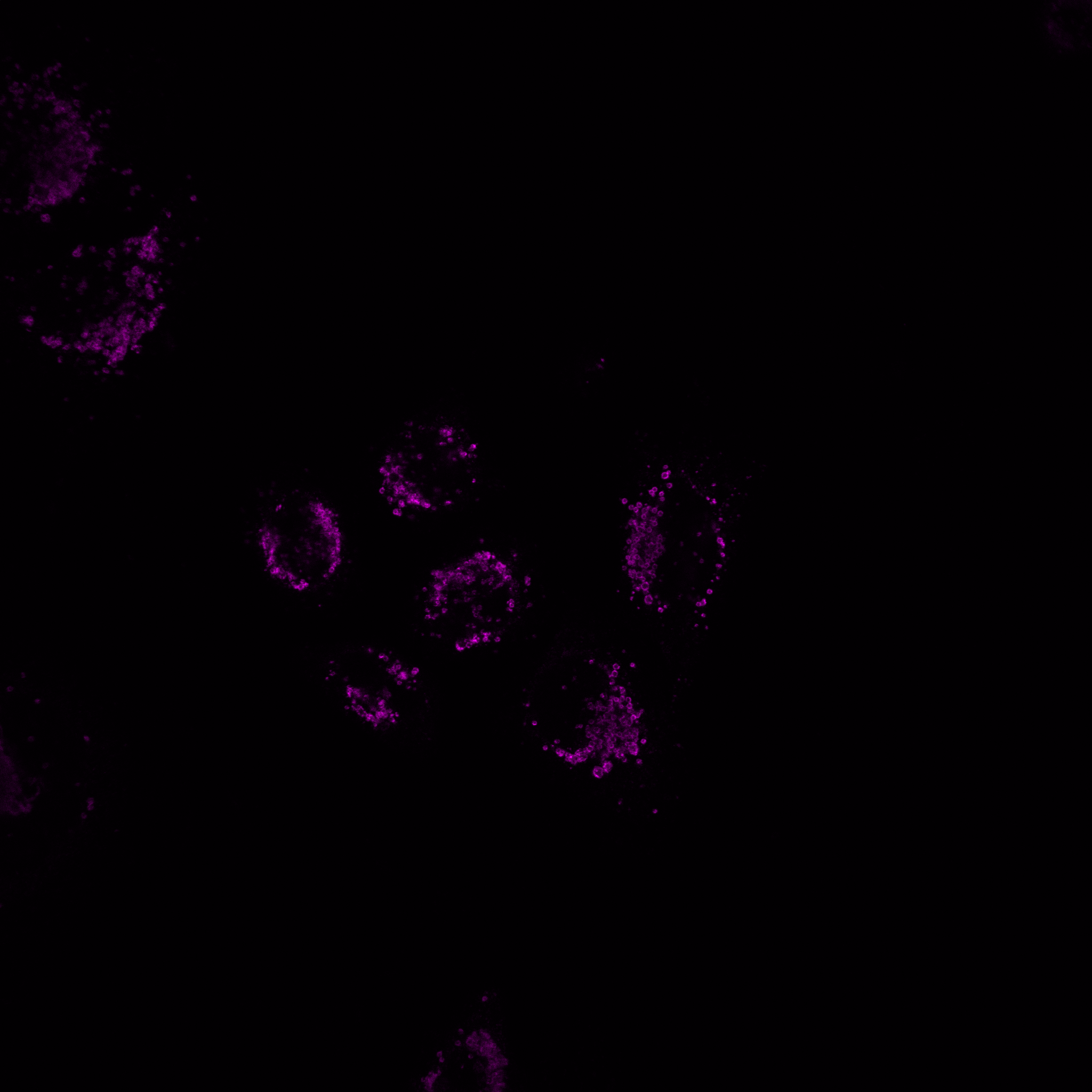

Supplement: Supplementary file 8 — Source Data for Figure 4 [file EMBR-24-e57300-s002.zip › Fig 4/4G/LLOMe_LAMP1.tif]

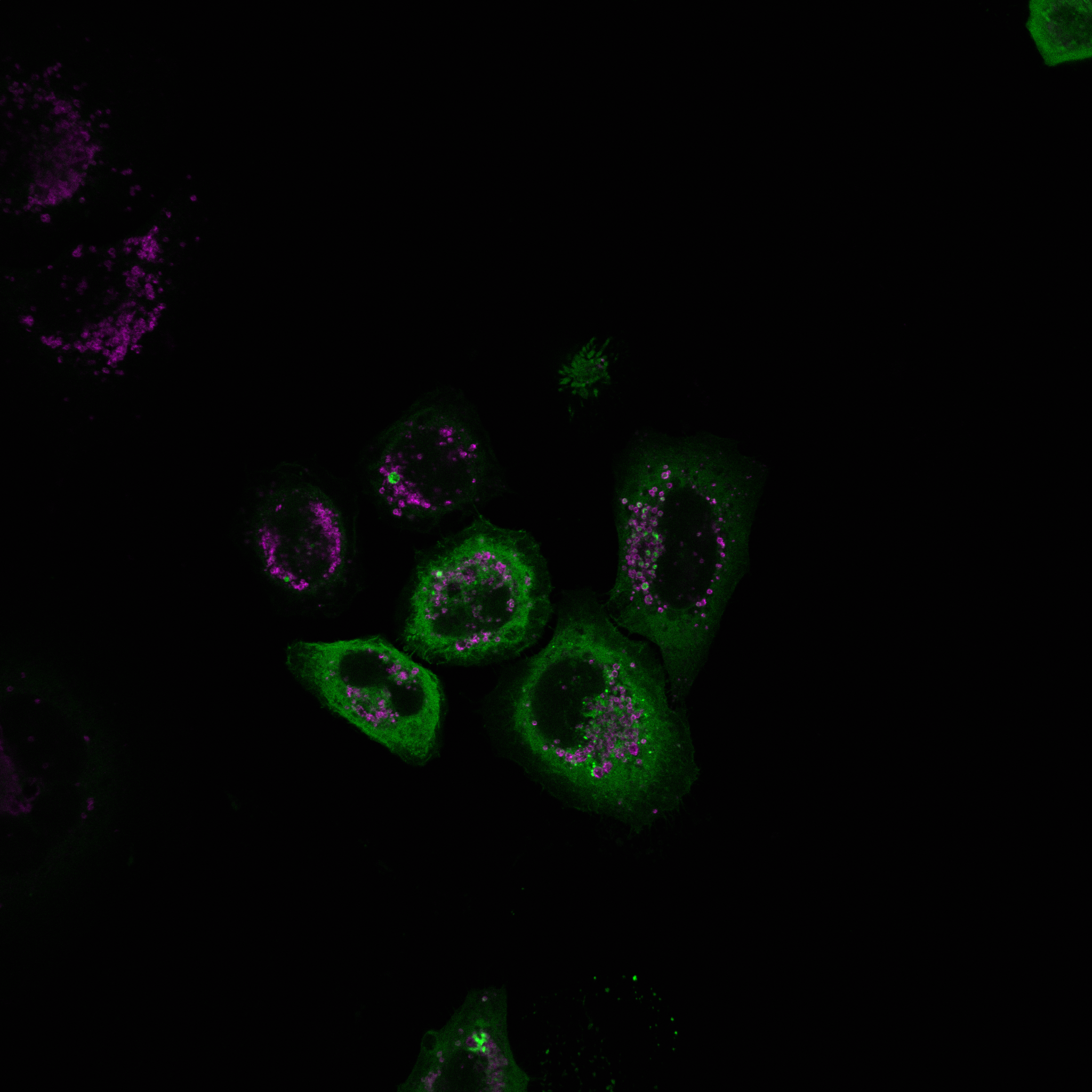

Supplement: Supplementary file 8 — Source Data for Figure 4 [file EMBR-24-e57300-s002.zip › Fig 4/4G/LLOMe_Merge.tif]

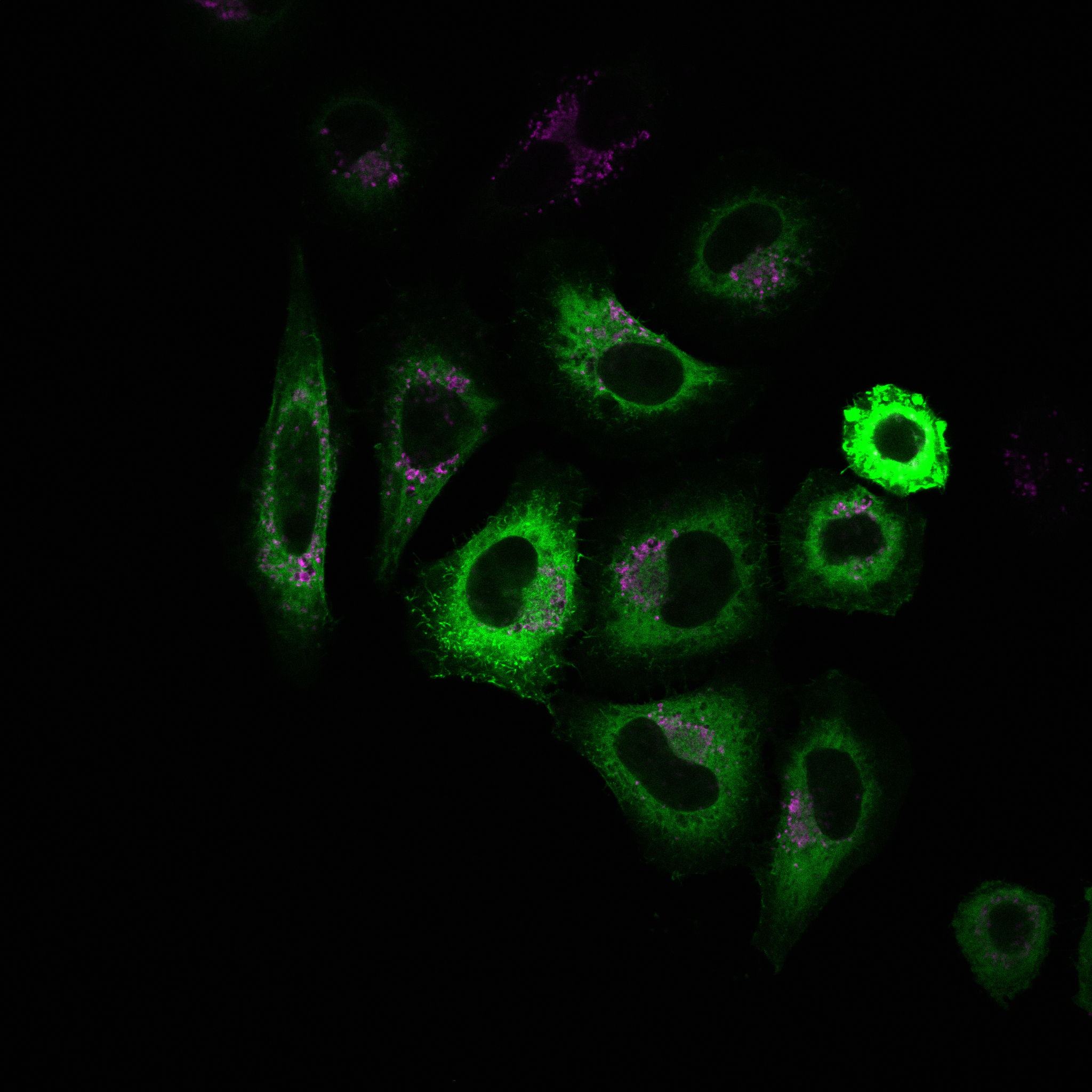

Supplement: Supplementary file 8 — Source Data for Figure 4 [file EMBR-24-e57300-s002.zip › Fig 4/4G/non-treated_Merge.tif]

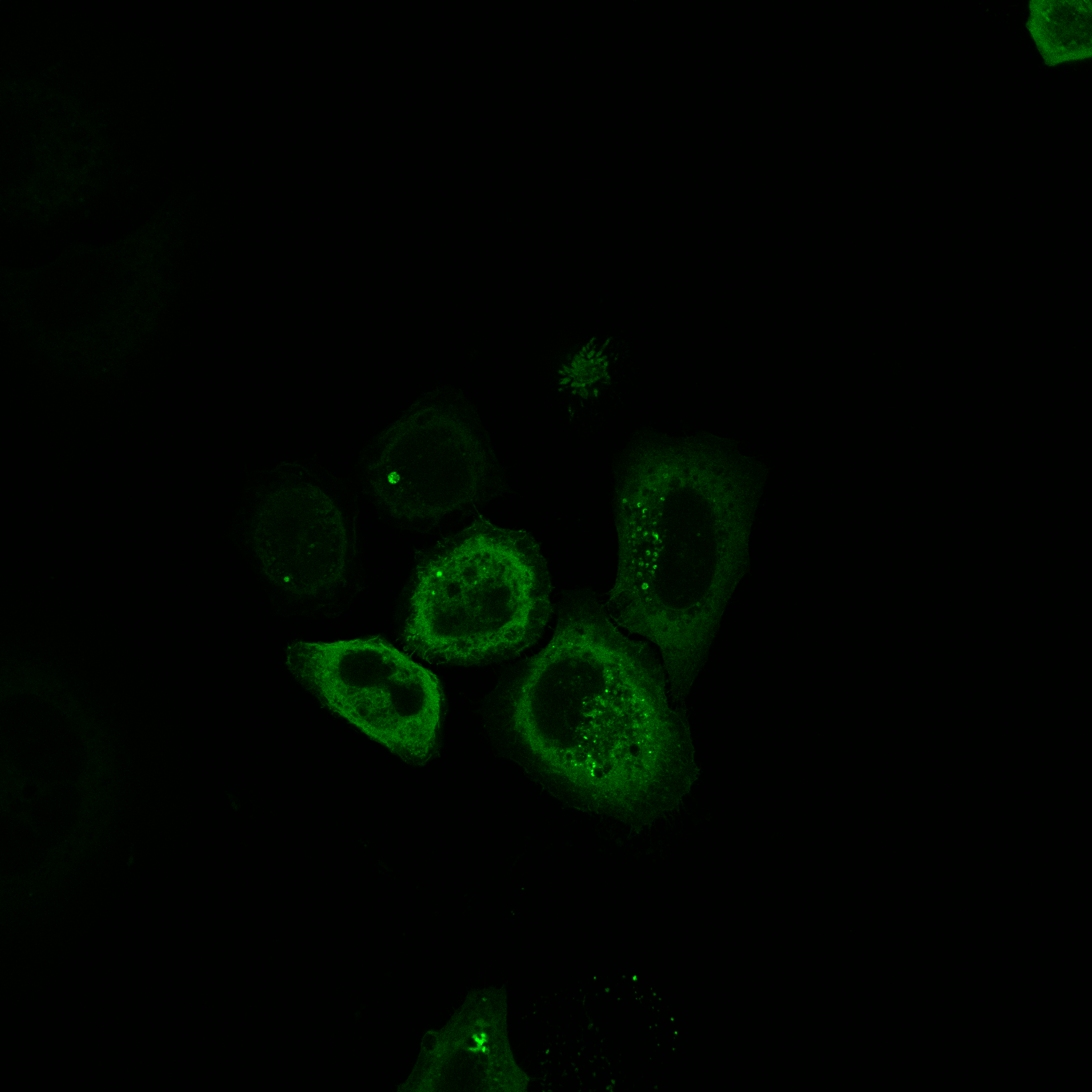

Supplement: Supplementary file 8 — Source Data for Figure 4 [file EMBR-24-e57300-s002.zip › Fig 4/4G/LLOMe_mNG-DOK1.tif]

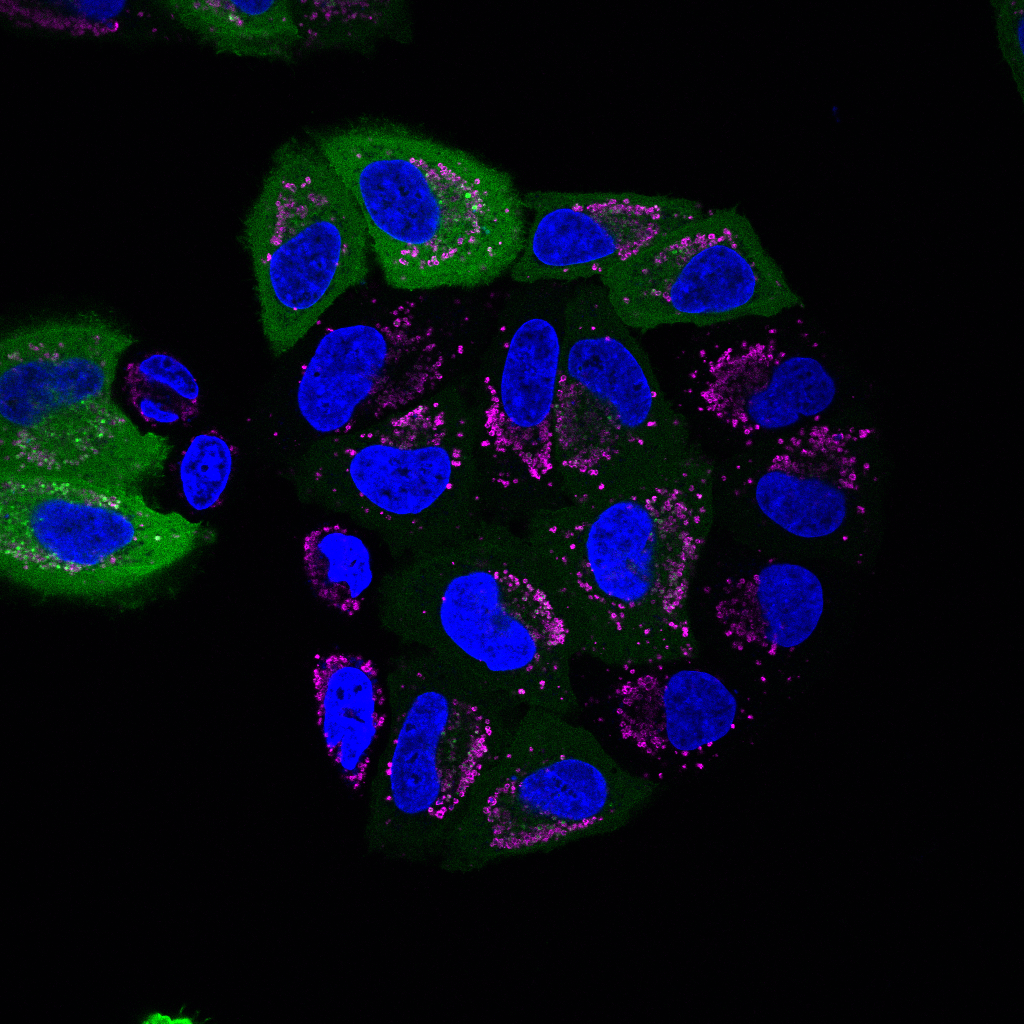

Supplement: Supplementary file 9 — Source Data for Figure 5 [file EMBR-24-e57300-s007.zip › Fig 5/5G/WT_LLOMe_Merge+DAPI.tif]

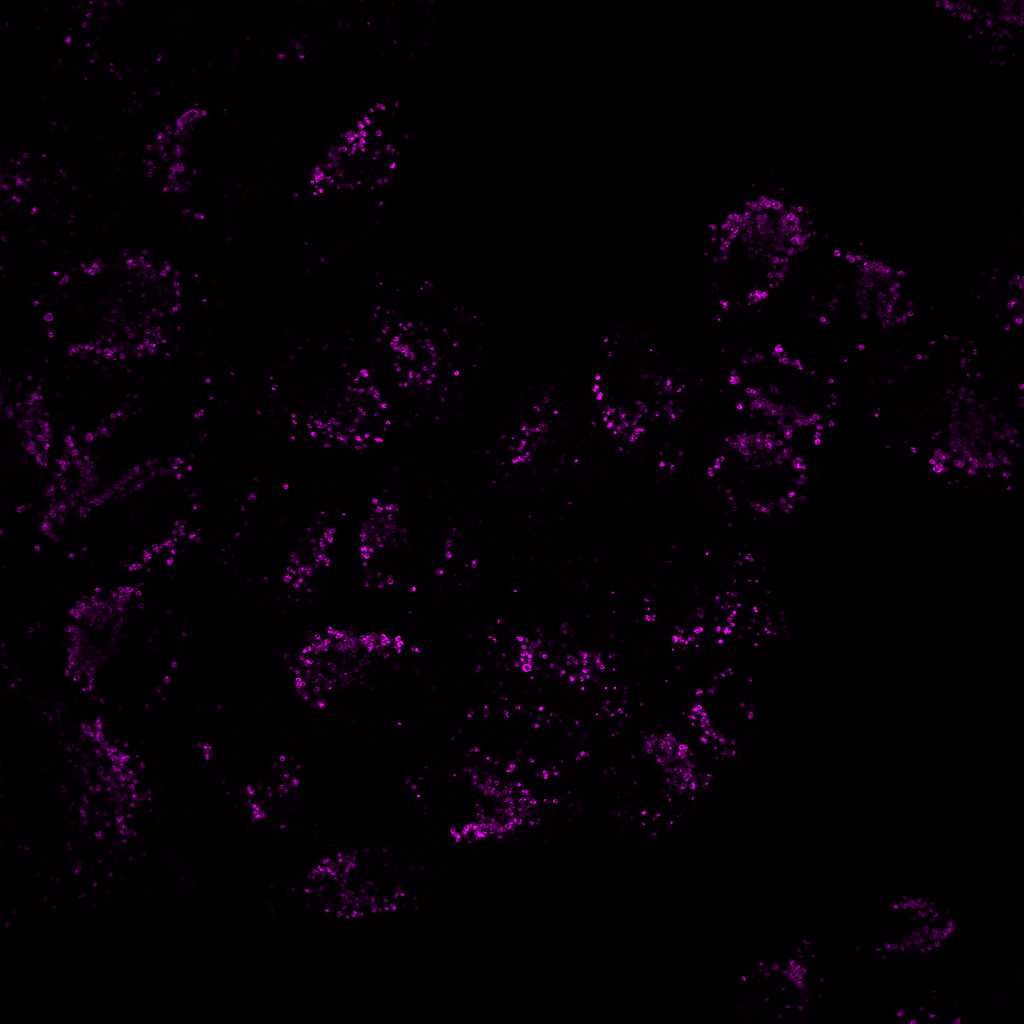

Supplement: Supplementary file 9 — Source Data for Figure 5 [file EMBR-24-e57300-s007.zip › Fig 5/5G/S269A_LLOMe_LAMP1.tif]

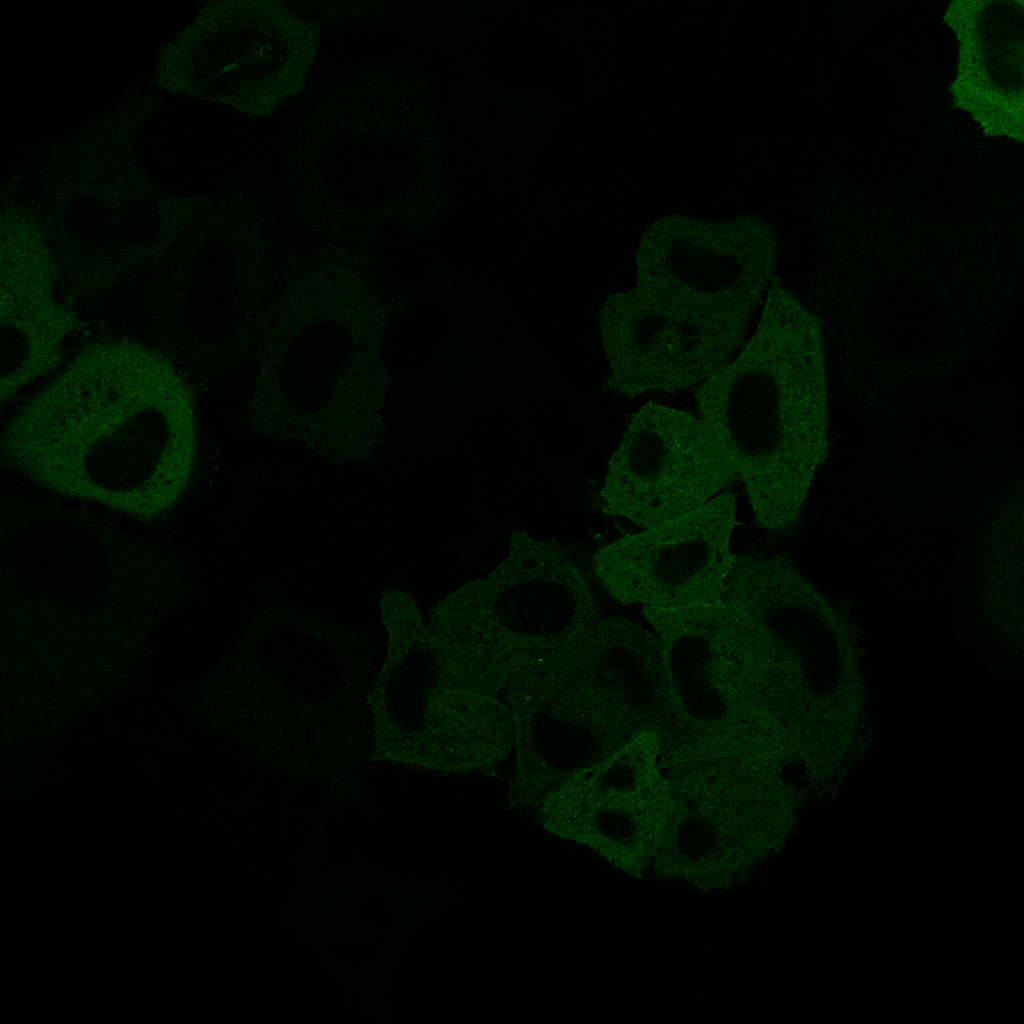

Supplement: Supplementary file 9 — Source Data for Figure 5 [file EMBR-24-e57300-s007.zip › Fig 5/5G/WT_non-treated_mNG-DOK1.tif]

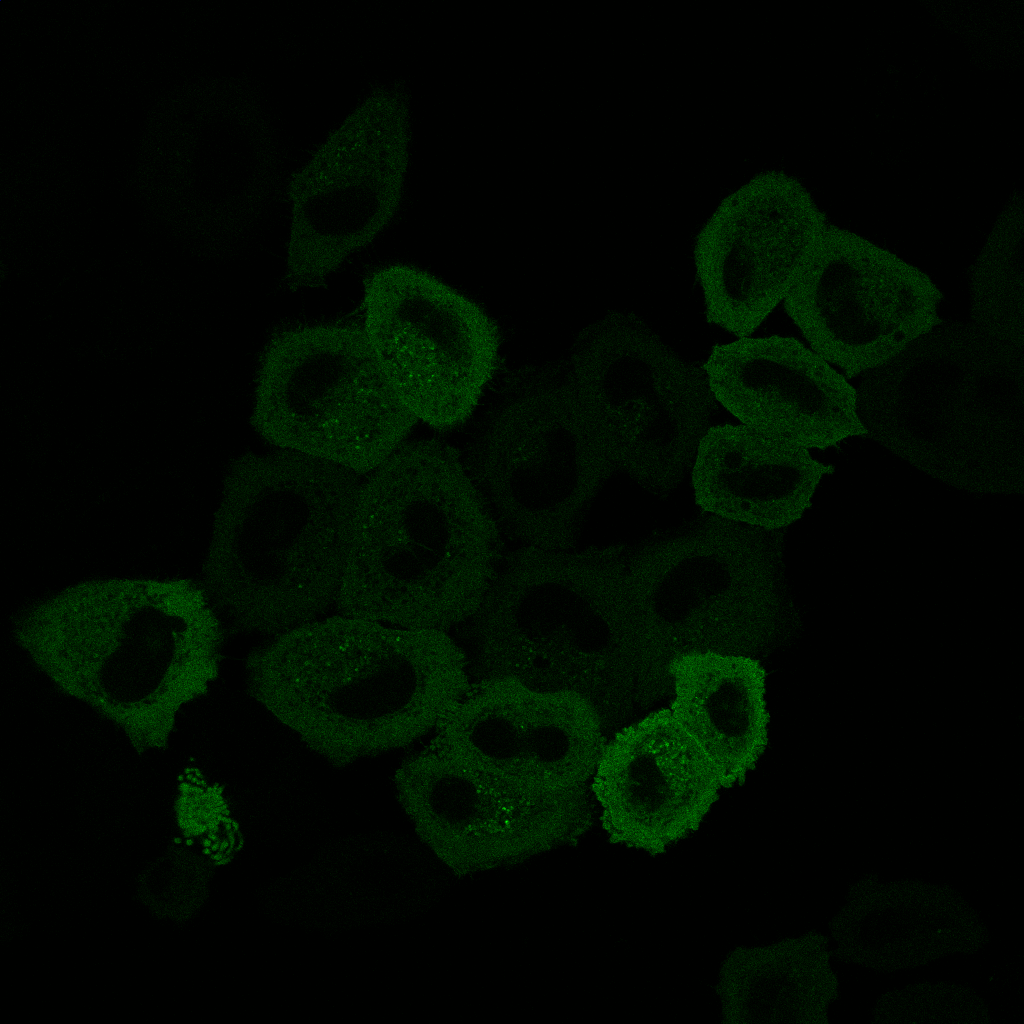

Supplement: Supplementary file 9 — Source Data for Figure 5 [file EMBR-24-e57300-s007.zip › Fig 5/5G/S269A_LLOMe_mMG-DOK1.tif]

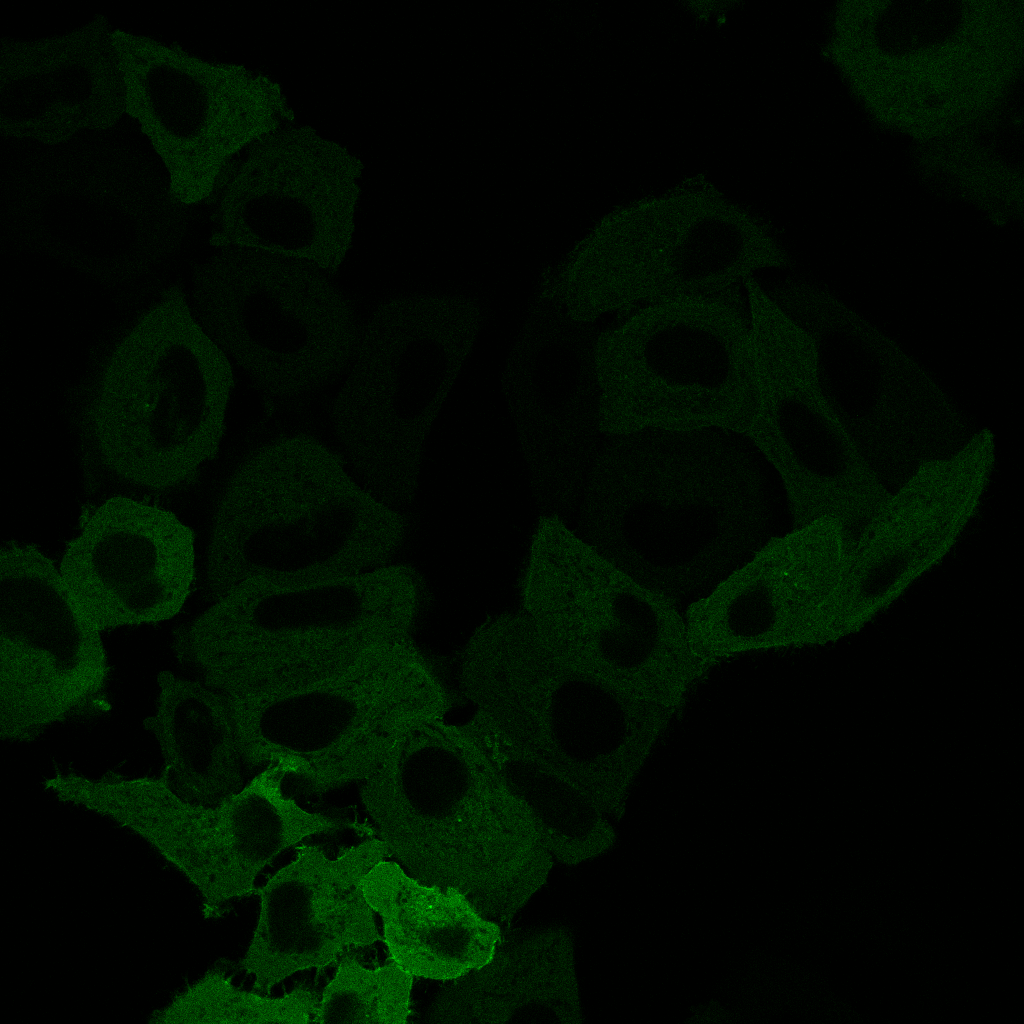

Supplement: Supplementary file 9 — Source Data for Figure 5 [file EMBR-24-e57300-s007.zip › Fig 5/5G/S269A_non-treated_mNG-DOK1.tif]

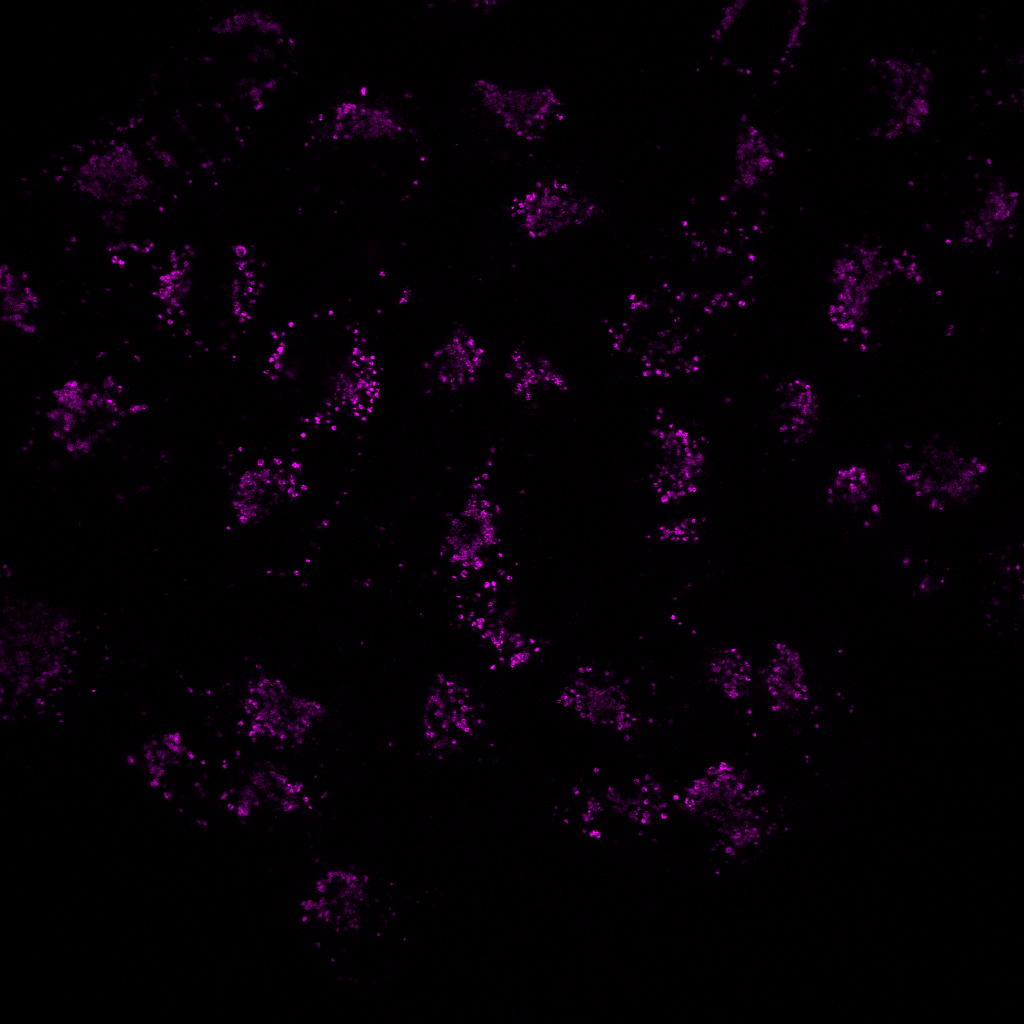

Supplement: Supplementary file 9 — Source Data for Figure 5 [file EMBR-24-e57300-s007.zip › Fig 5/5G/WT_non-treated_LAMP1.tif]

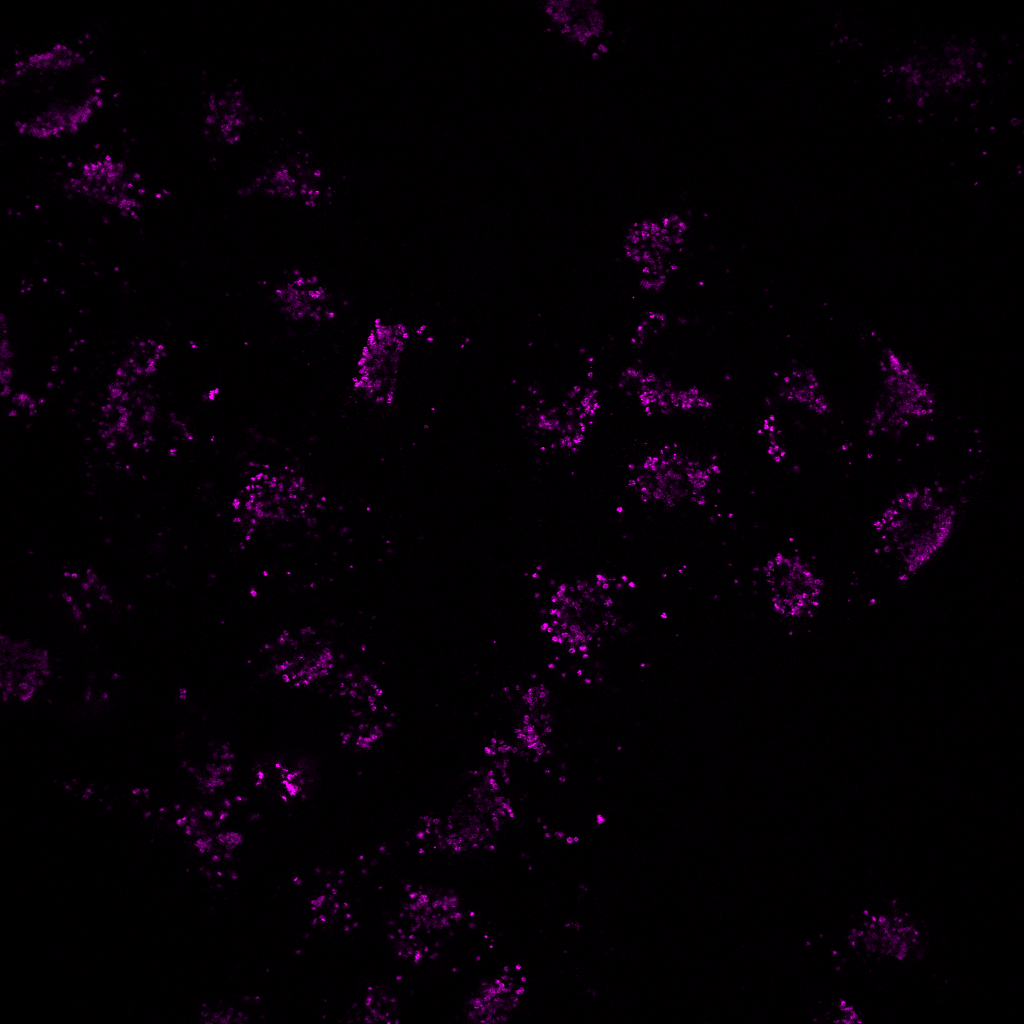

Supplement: Supplementary file 9 — Source Data for Figure 5 [file EMBR-24-e57300-s007.zip › Fig 5/5G/S269A_non-treated_LAMP1.tif]

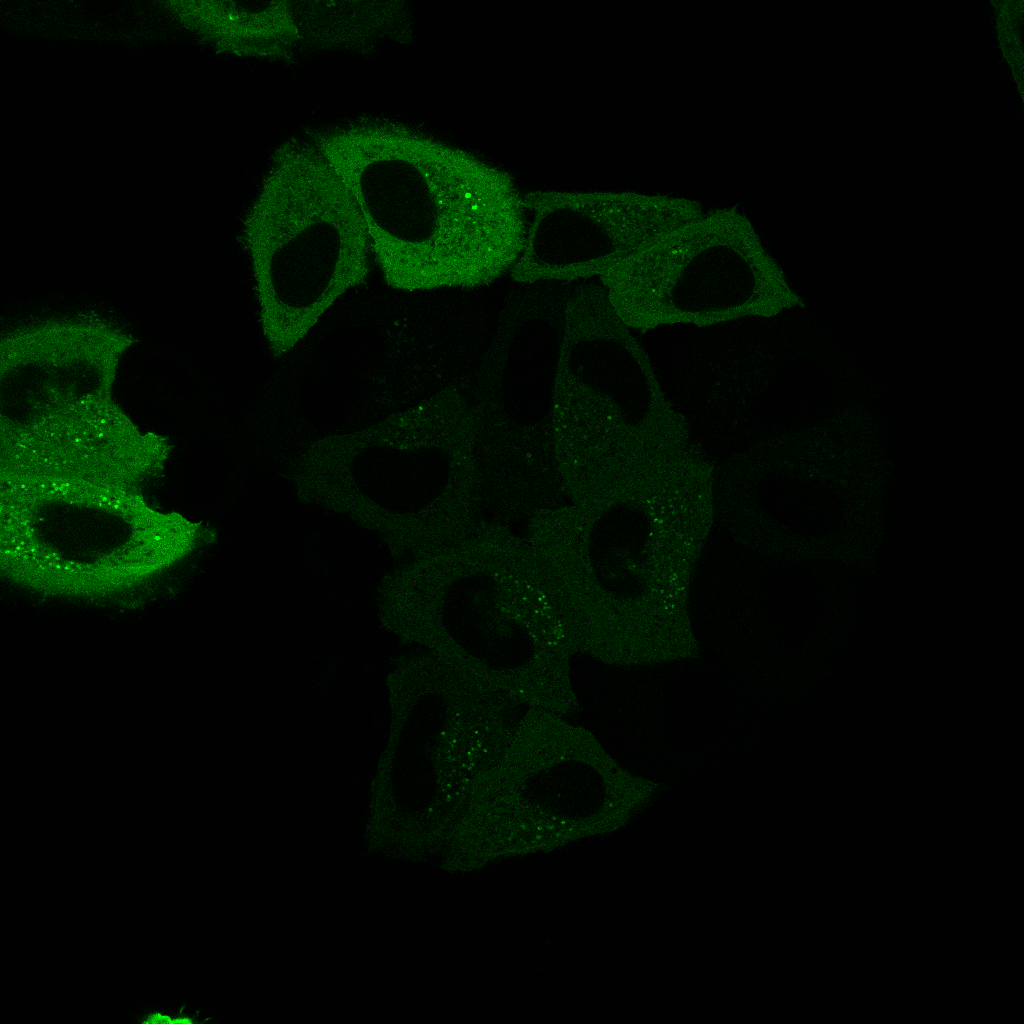

Supplement: Supplementary file 9 — Source Data for Figure 5 [file EMBR-24-e57300-s007.zip › Fig 5/5G/WT_LLOMe_mNG-DOK1.tif]

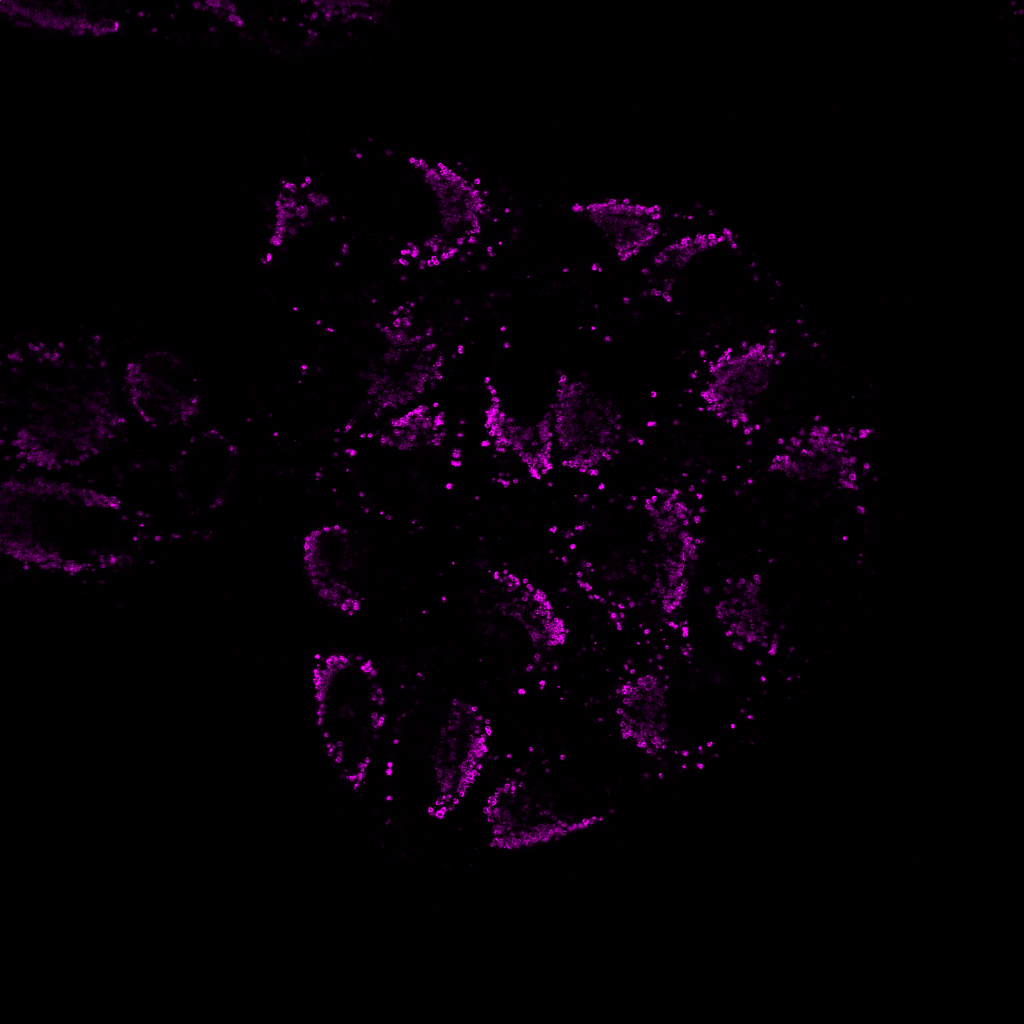

Supplement: Supplementary file 9 — Source Data for Figure 5 [file EMBR-24-e57300-s007.zip › Fig 5/5G/WT_LLOMe_LAMP1.tif]

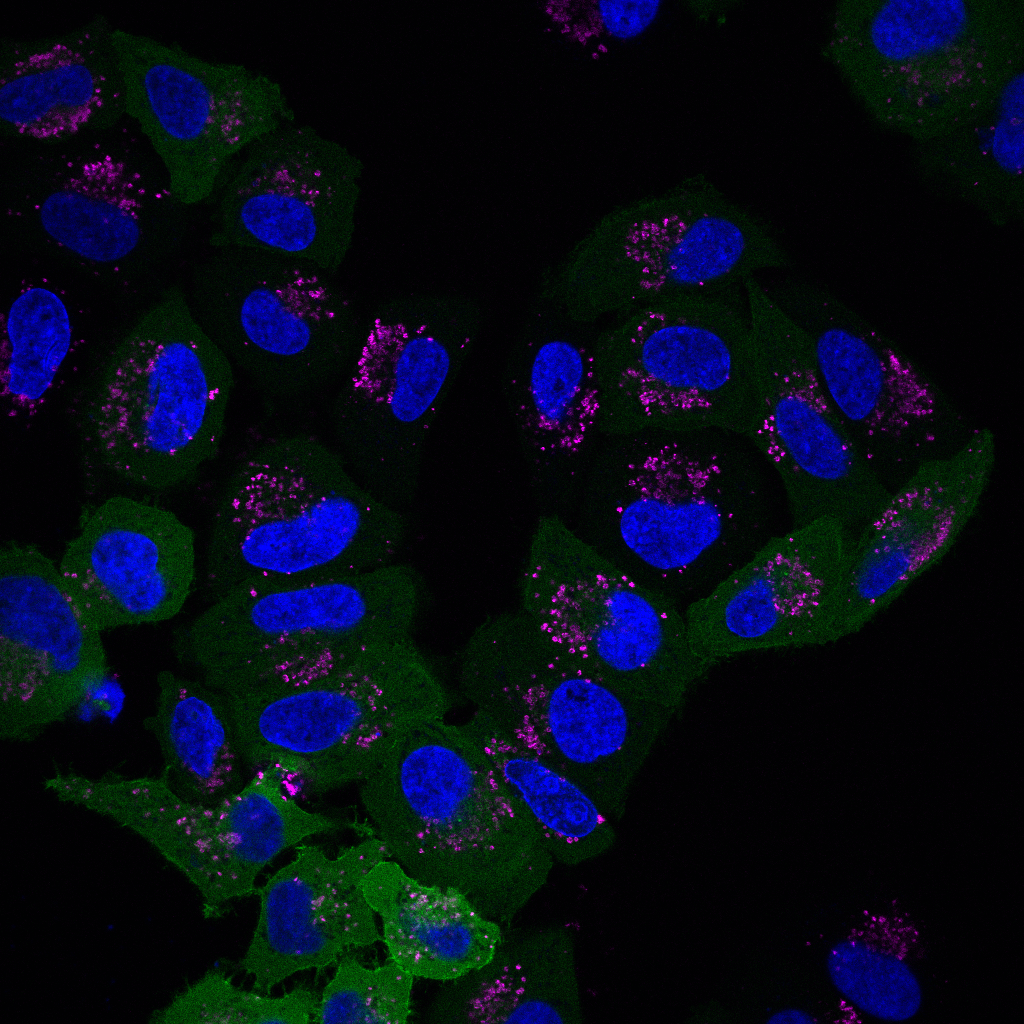

Supplement: Supplementary file 9 — Source Data for Figure 5 [file EMBR-24-e57300-s007.zip › Fig 5/5G/S269A_non-treated_Merge+DAPI.tif]

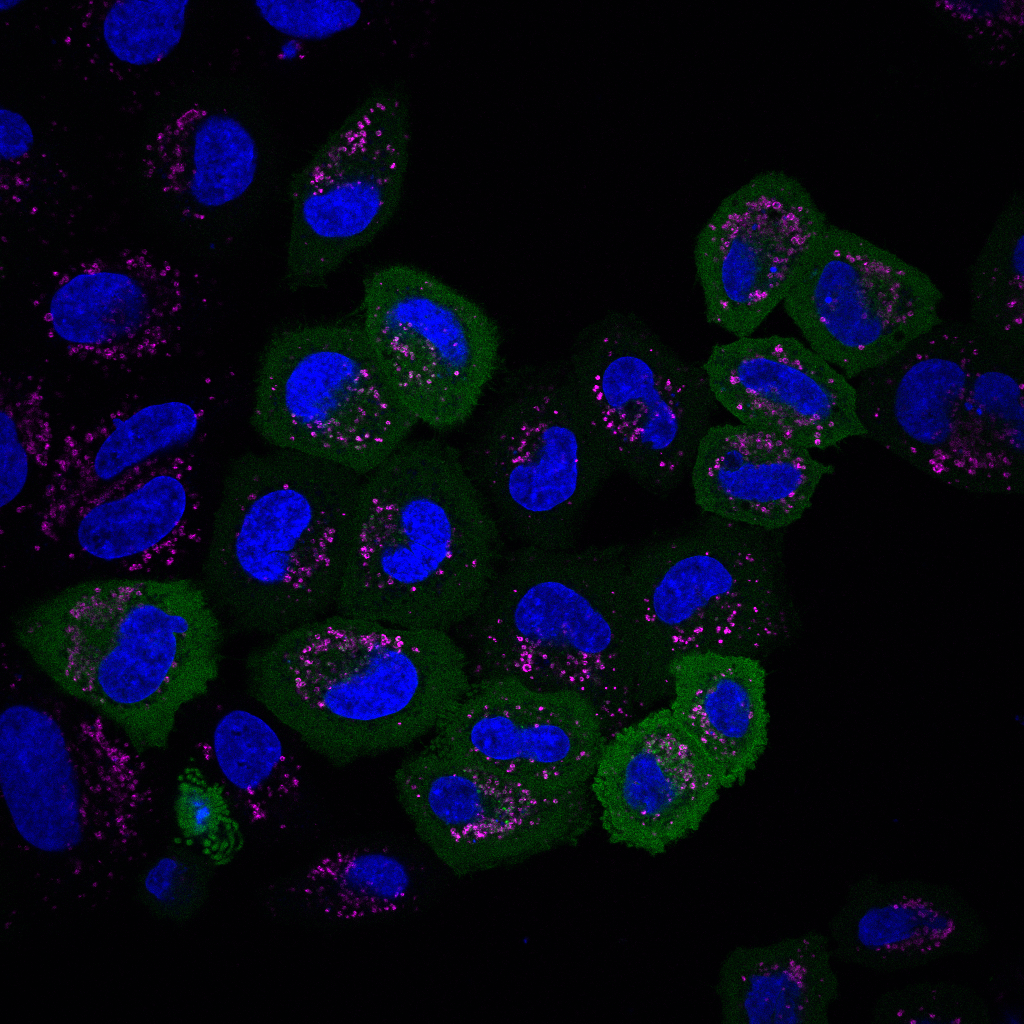

Supplement: Supplementary file 9 — Source Data for Figure 5 [file EMBR-24-e57300-s007.zip › Fig 5/5G/S269A_LLOMe_Merge+DAPI.tif]

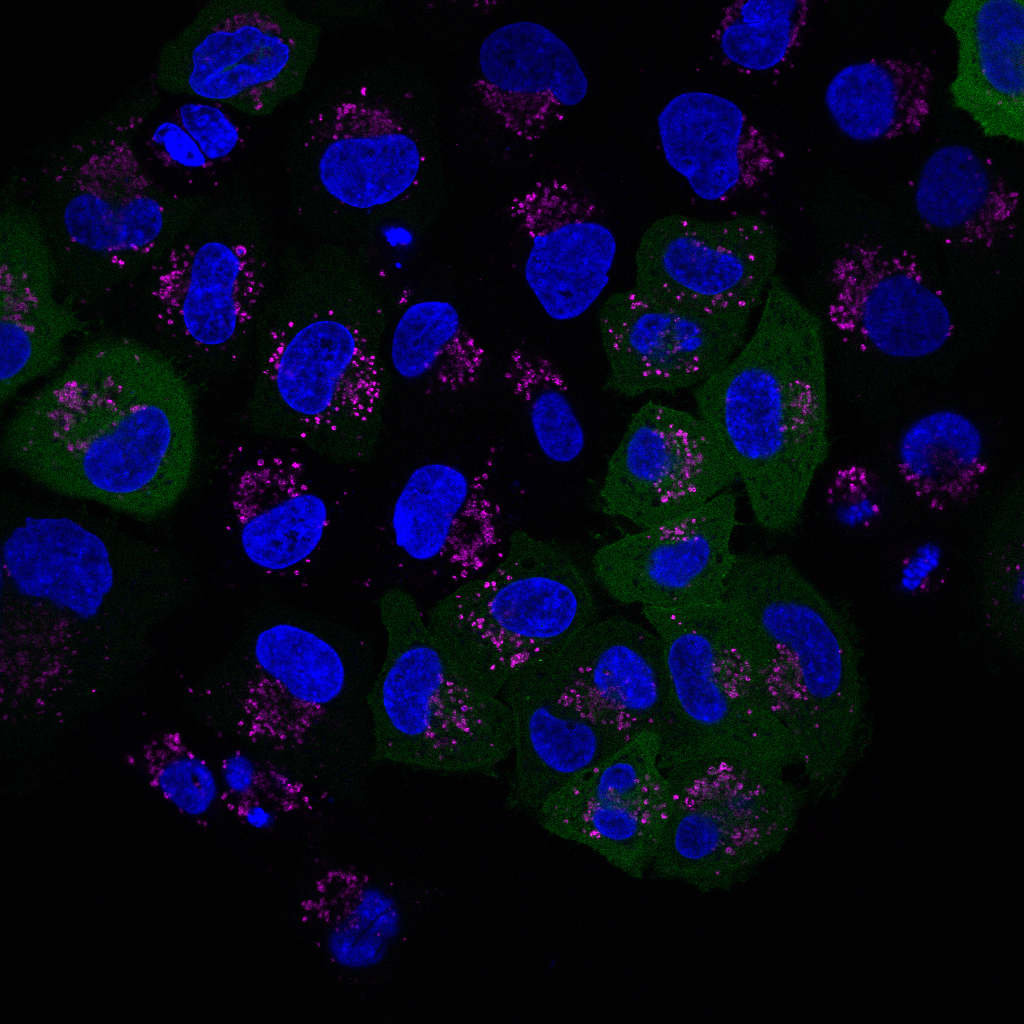

Supplement: Supplementary file 9 — Source Data for Figure 5 [file EMBR-24-e57300-s007.zip › Fig 5/5G/WT_non-treated_Merge+DAPI.tif]

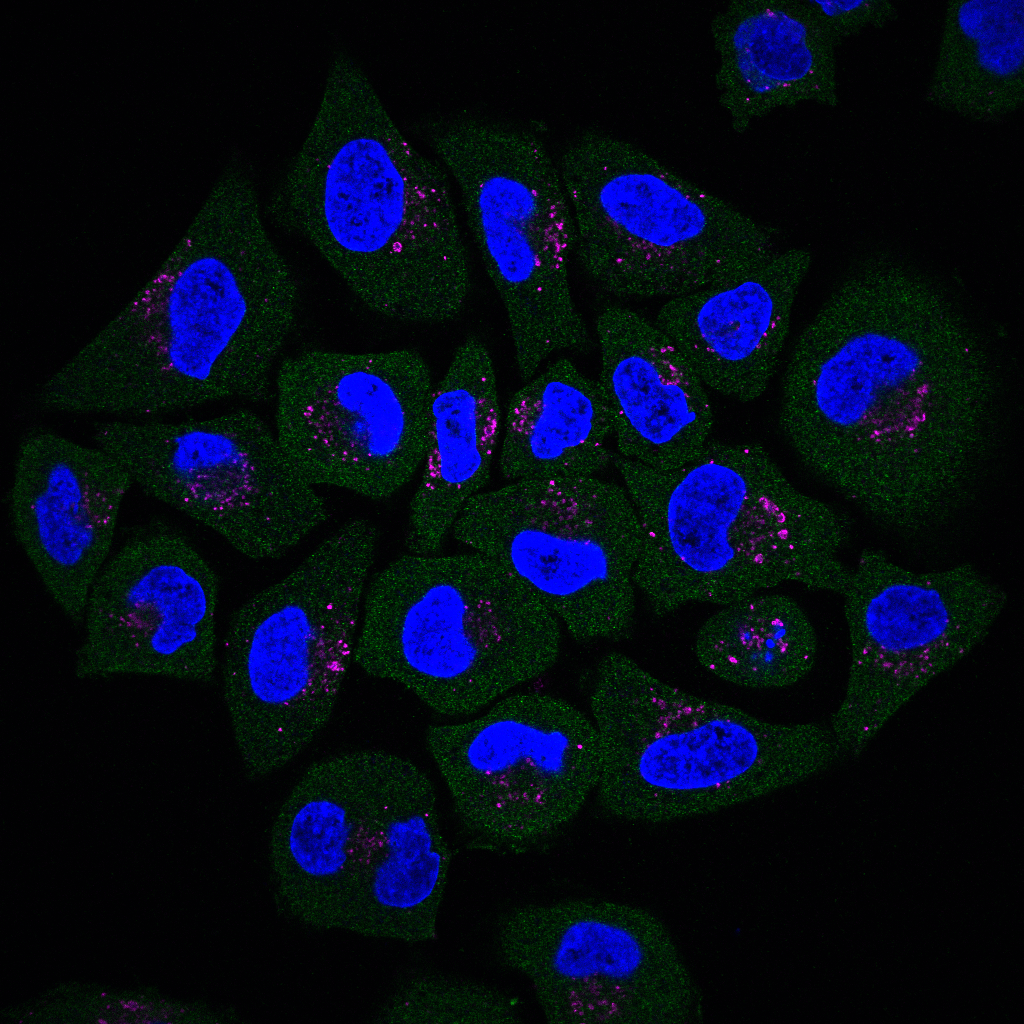

Supplement: Supplementary file 9 — Source Data for Figure 5 [file EMBR-24-e57300-s007.zip › Fig 5/5C/siControl_Flag_non-treated_Merge+DAPI.tif]

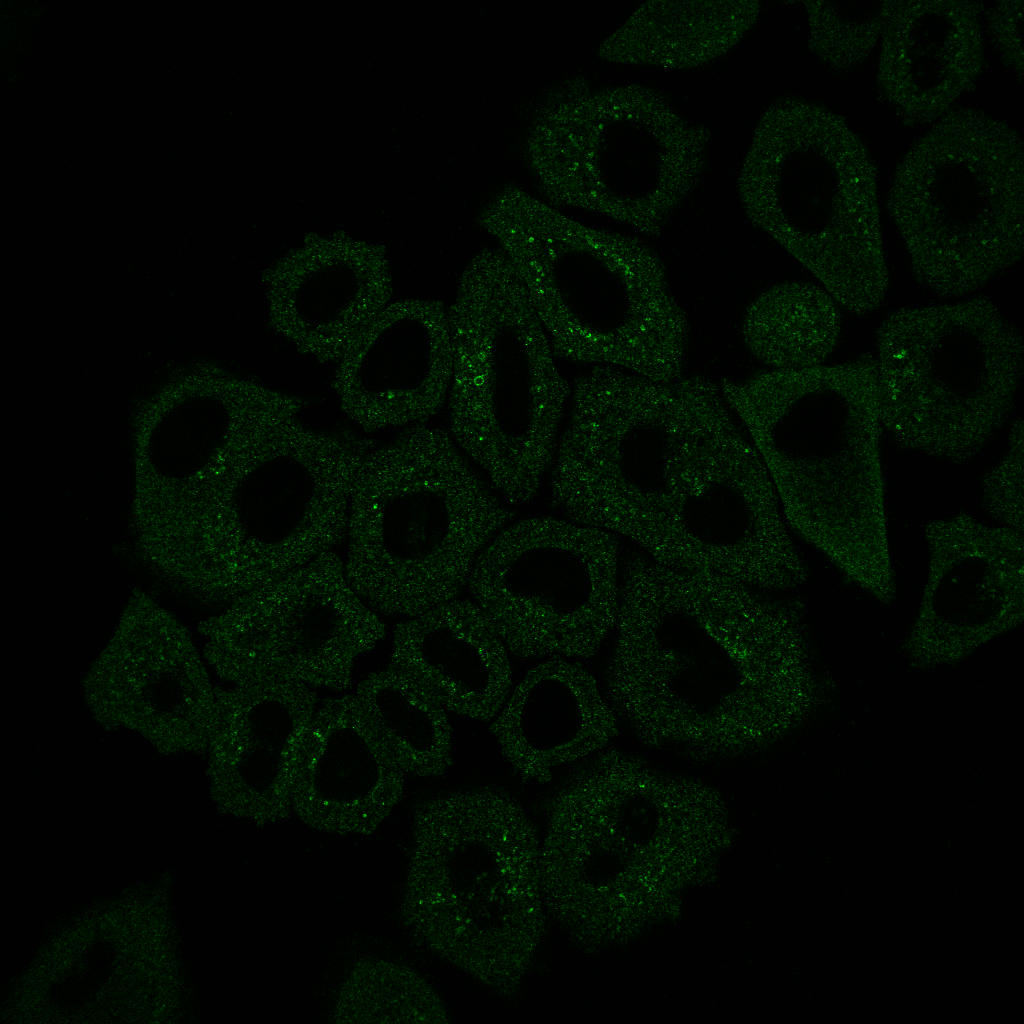

Supplement: Supplementary file 9 — Source Data for Figure 5 [file EMBR-24-e57300-s007.zip › Fig 5/5C/siDOK1_Flag-DOK1 WT_LLOMe_VPS4.tif]

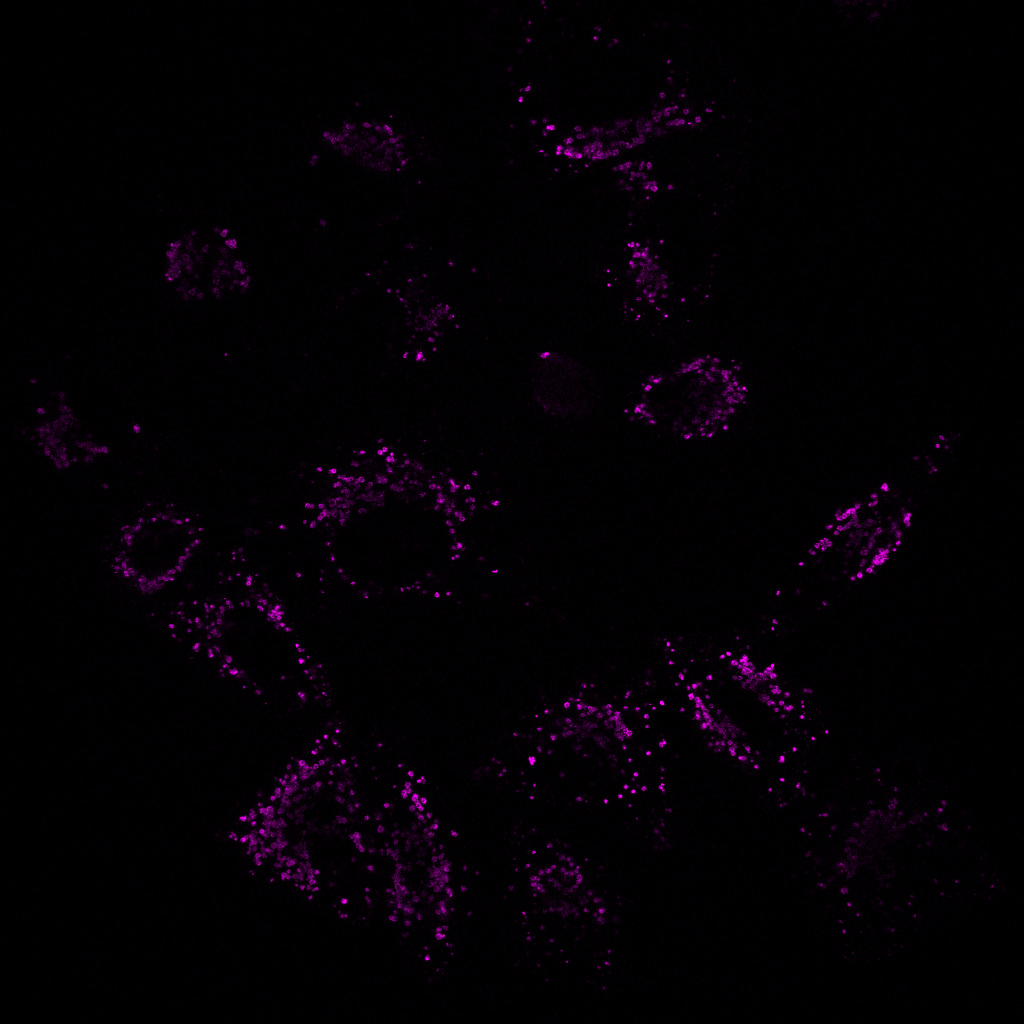

Supplement: Supplementary file 9 — Source Data for Figure 5 [file EMBR-24-e57300-s007.zip › Fig 5/5C/siDOK1_Flag_LLOMe_LAMP1.tif]

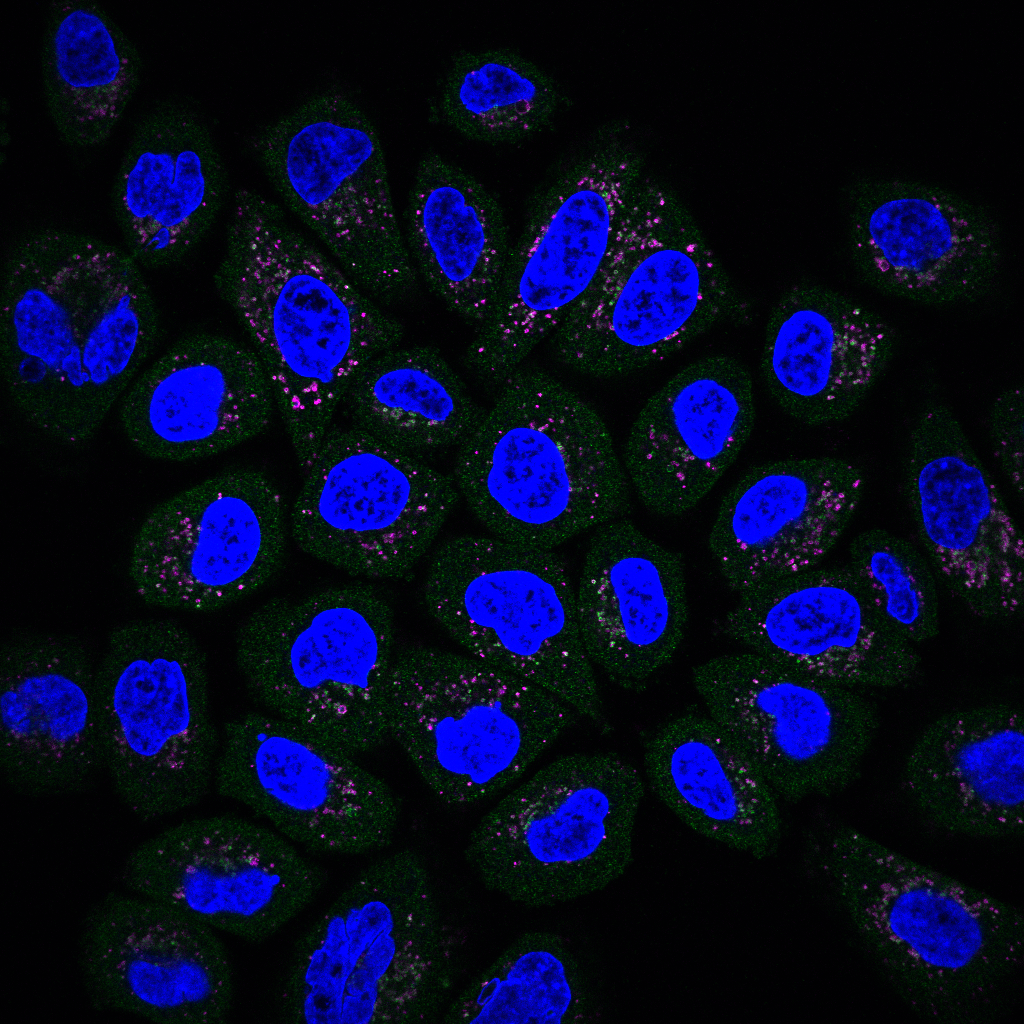

Supplement: Supplementary file 9 — Source Data for Figure 5 [file EMBR-24-e57300-s007.zip › Fig 5/5C/siControl_Fleg_LLOMe_Merge+DAPI.tif]

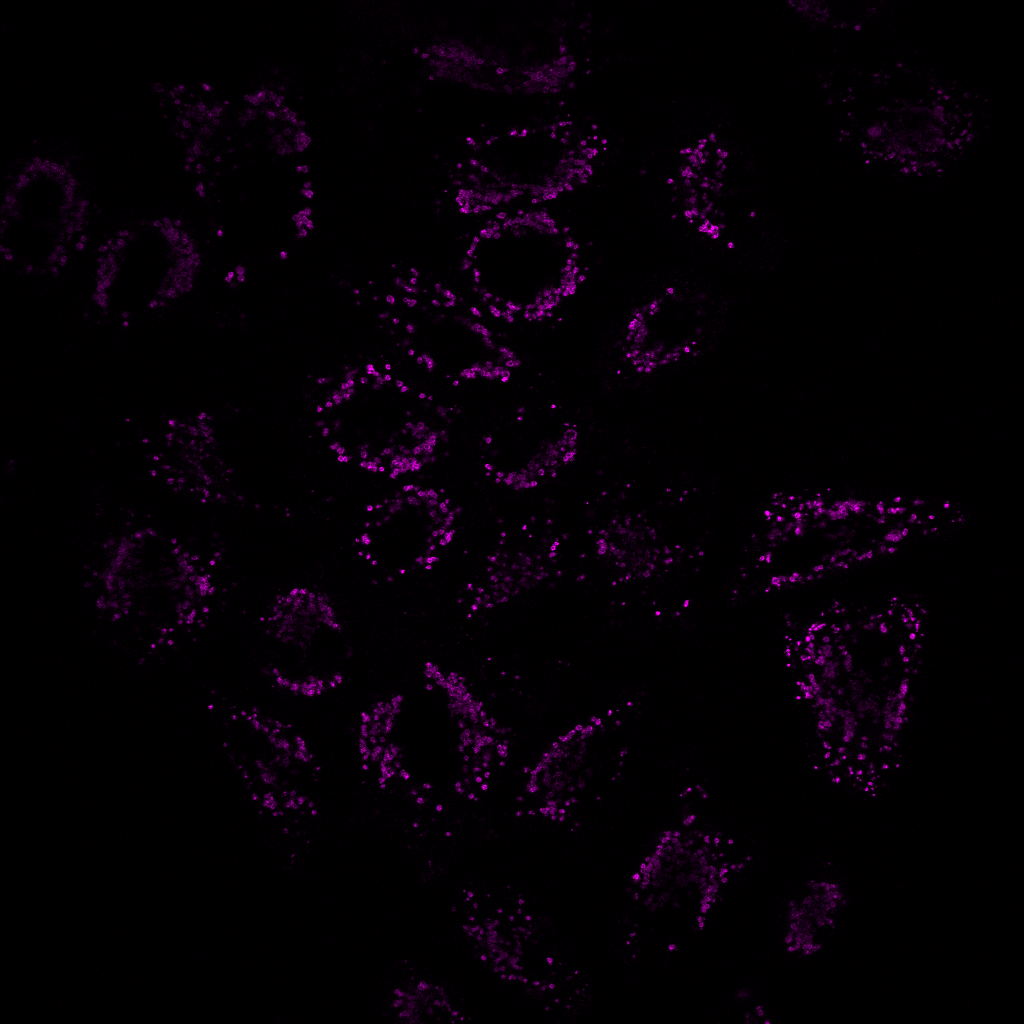

Supplement: Supplementary file 9 — Source Data for Figure 5 [file EMBR-24-e57300-s007.zip › Fig 5/5C/siDOK1_Flag-DOK1 S269A_LLOMe_LAMP1.tif]

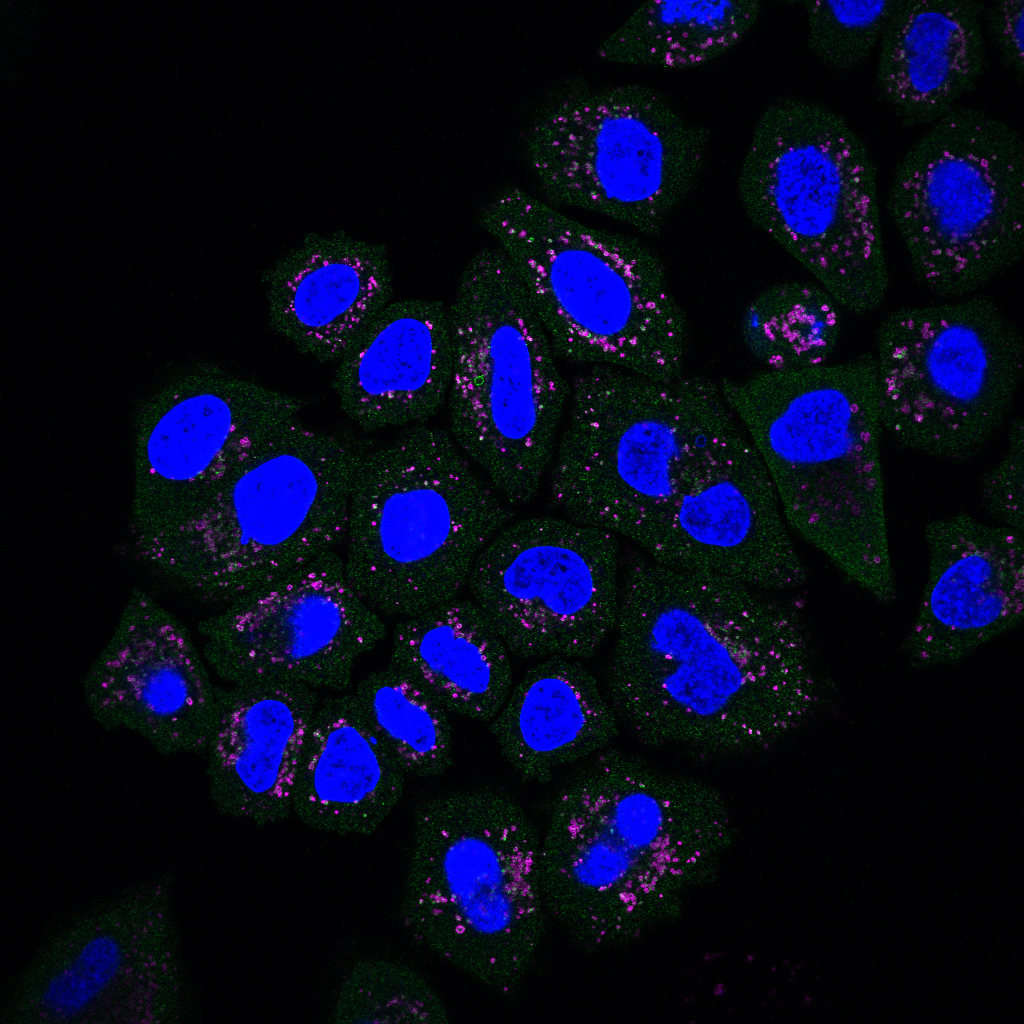

Supplement: Supplementary file 9 — Source Data for Figure 5 [file EMBR-24-e57300-s007.zip › Fig 5/5C/siDOK1_Flag-DOK1 WT_LLOMe_Merge+DAPI.tif]

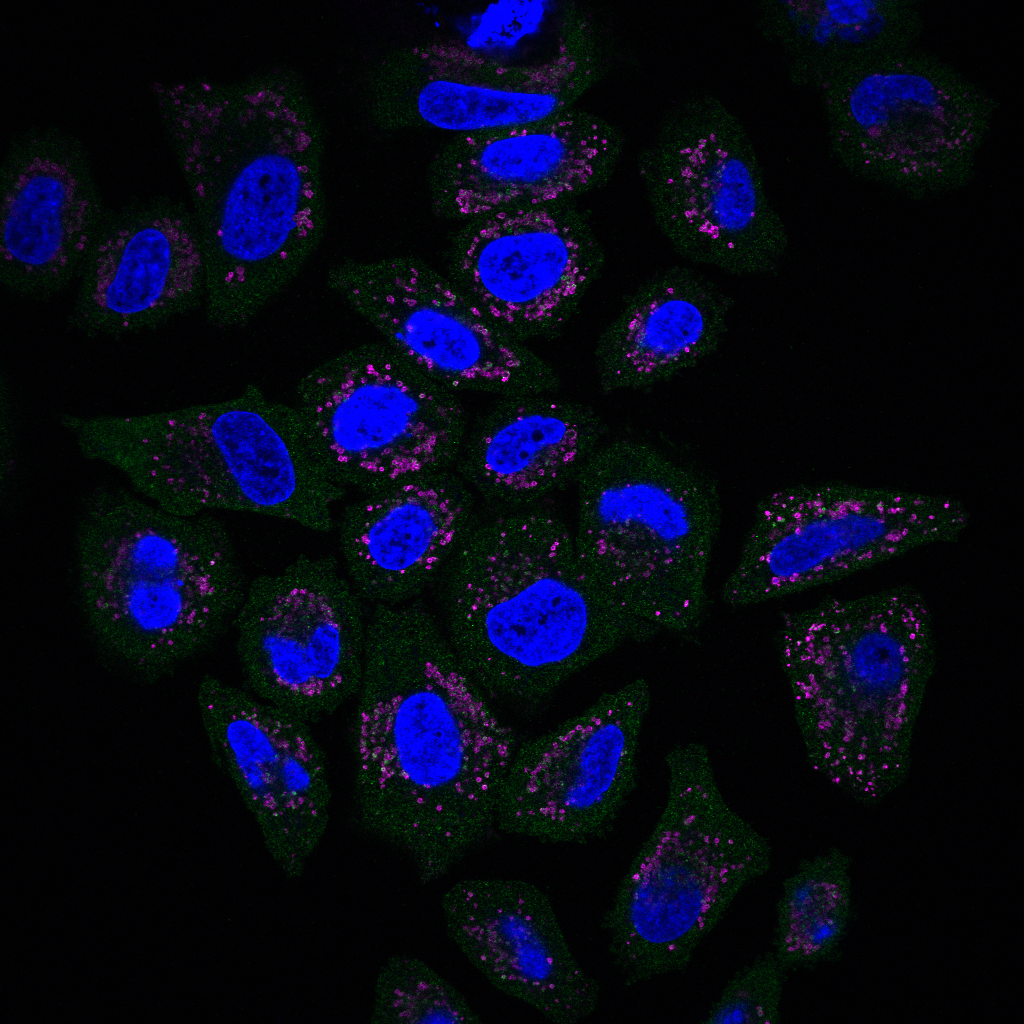

Supplement: Supplementary file 9 — Source Data for Figure 5 [file EMBR-24-e57300-s007.zip › Fig 5/5C/siDOK1_Flag-DOK1 S269A_LLOMe_Merge+DAPI.tif]

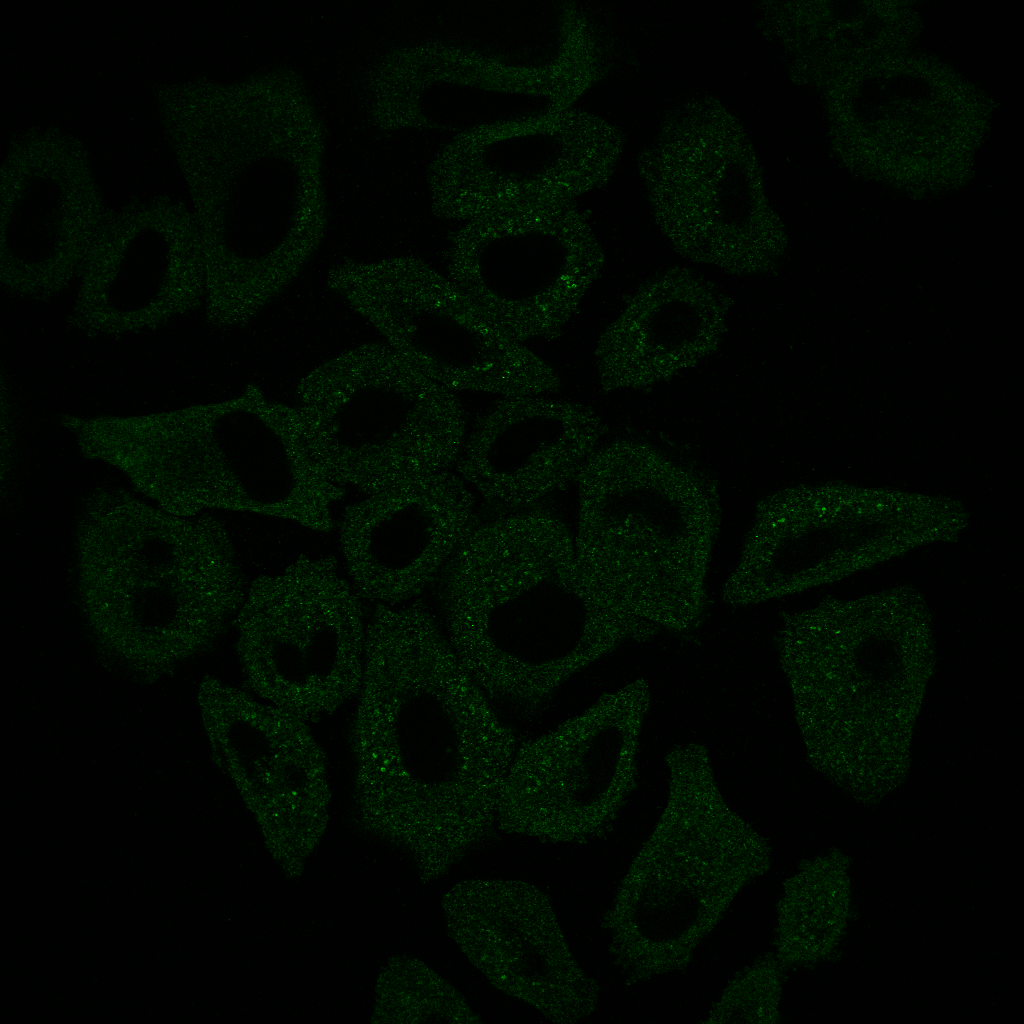

Supplement: Supplementary file 9 — Source Data for Figure 5 [file EMBR-24-e57300-s007.zip › Fig 5/5C/siDOK1_Flag-DOK1 S269A_LLOMe_VPS4.tif]

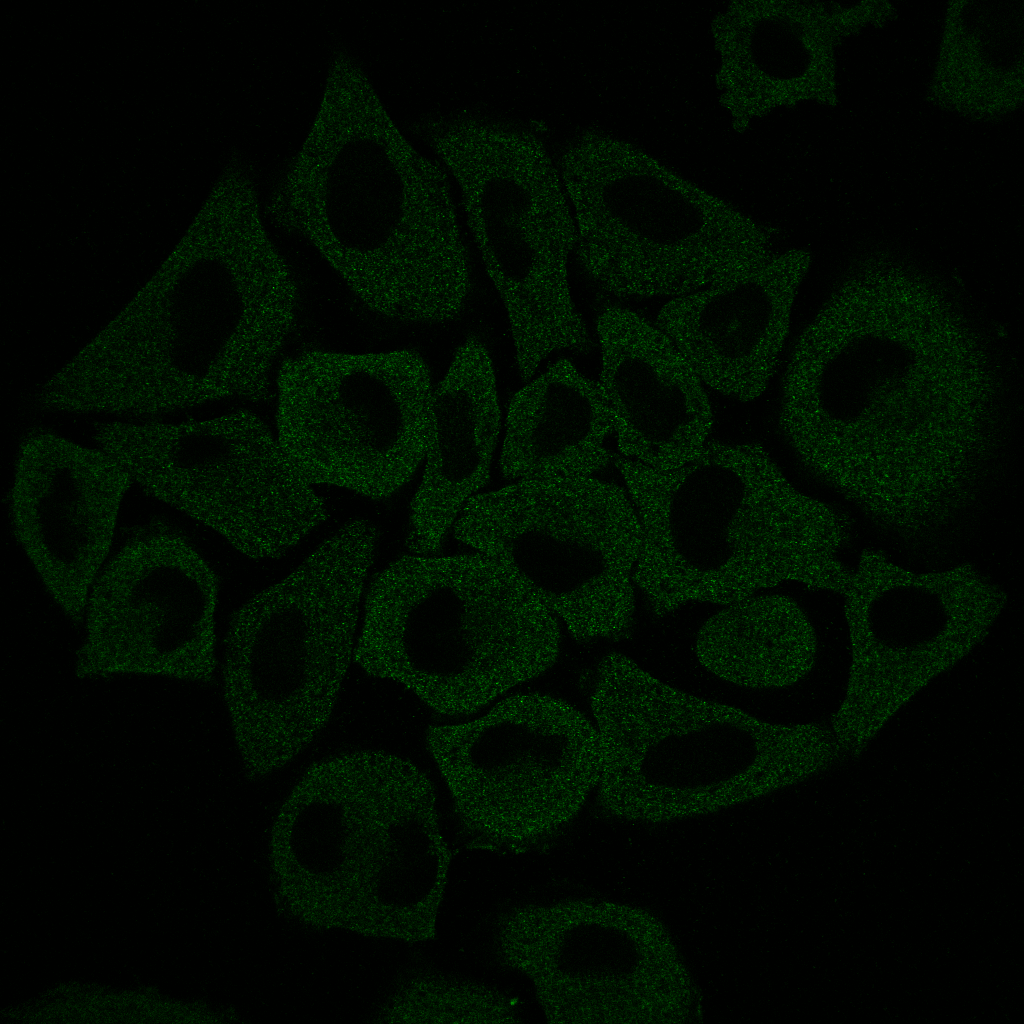

Supplement: Supplementary file 9 — Source Data for Figure 5 [file EMBR-24-e57300-s007.zip › Fig 5/5C/siControl_Flag_non-treated_VPS4.tif]

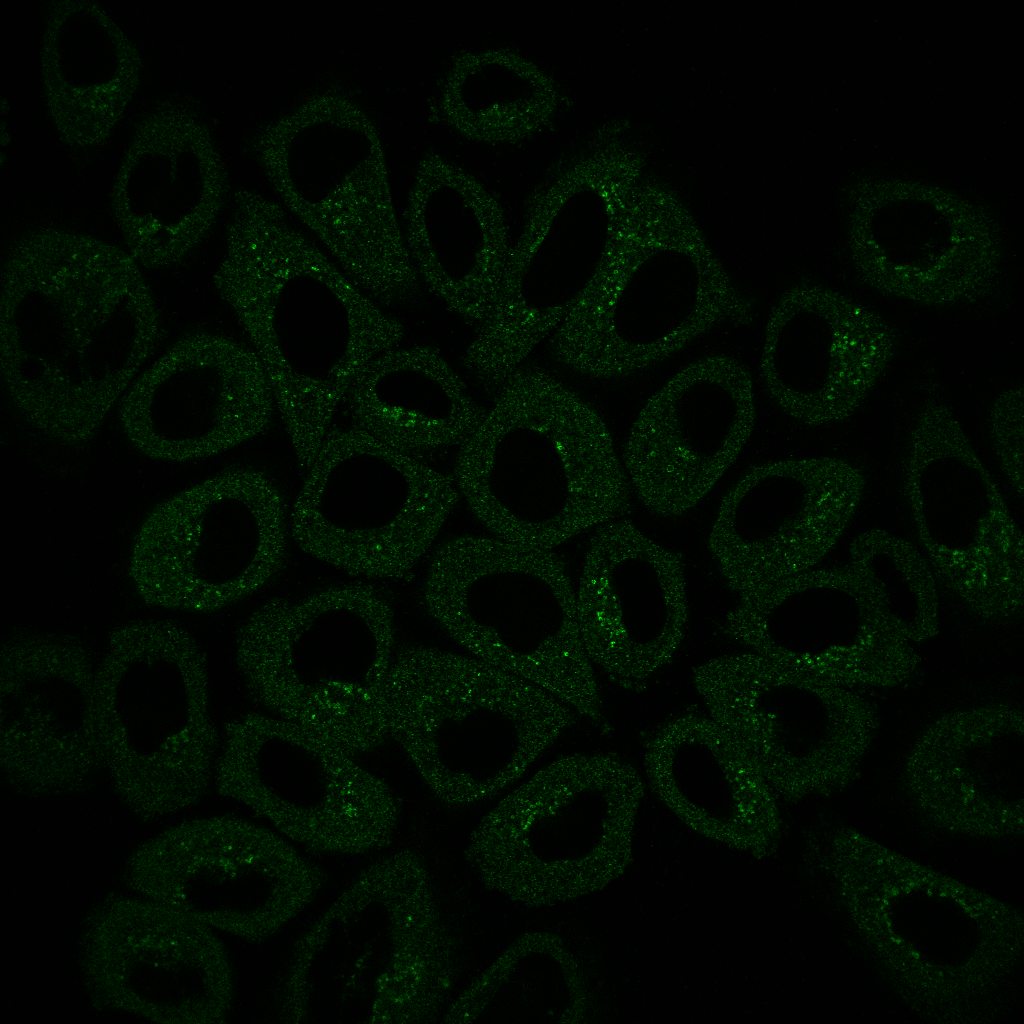

Supplement: Supplementary file 9 — Source Data for Figure 5 [file EMBR-24-e57300-s007.zip › Fig 5/5C/siControl_Flag_LLOMe_VPS4.tif]

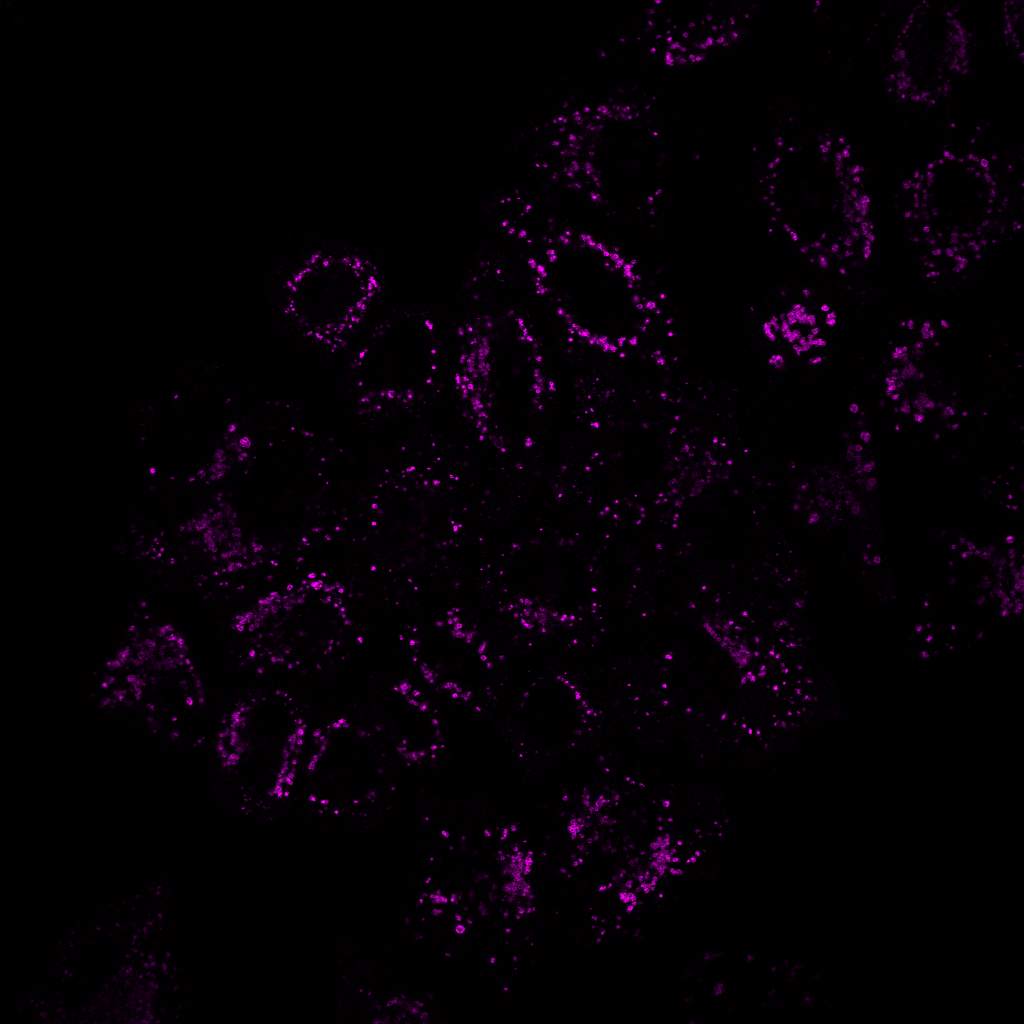

Supplement: Supplementary file 9 — Source Data for Figure 5 [file EMBR-24-e57300-s007.zip › Fig 5/5C/siDOK1_Flag-DOK1 WT_LLOMe_LAMP1.tif]

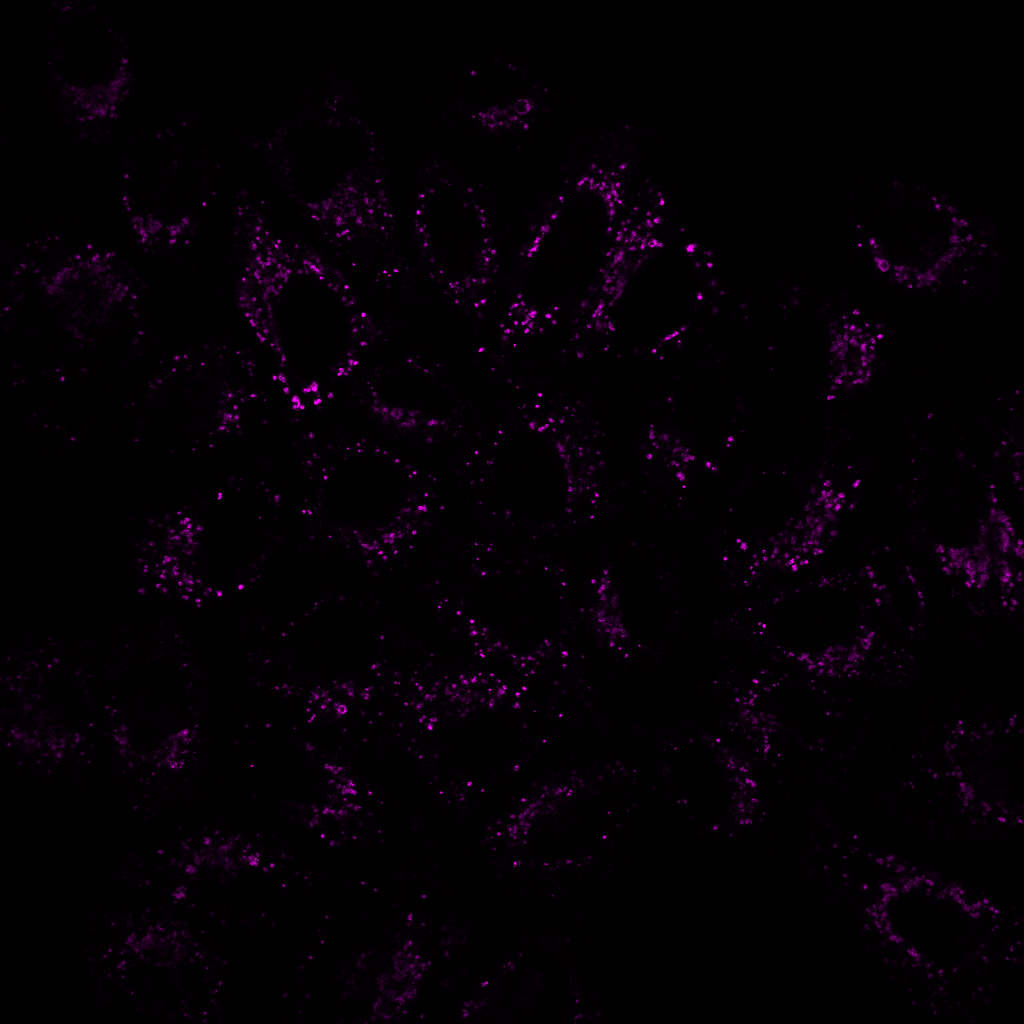

Supplement: Supplementary file 9 — Source Data for Figure 5 [file EMBR-24-e57300-s007.zip › Fig 5/5C/siControl_Flag_LLOMe_LAMP1.tif]

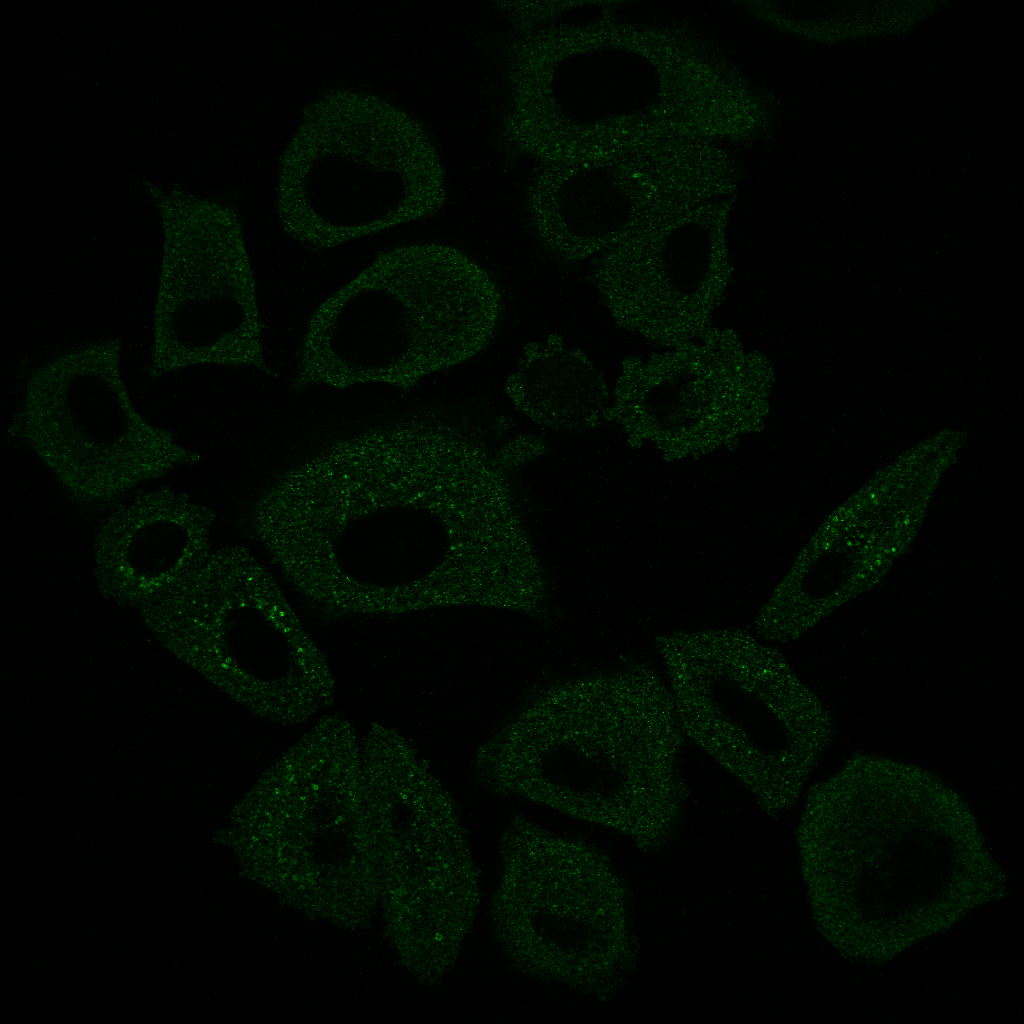

Supplement: Supplementary file 9 — Source Data for Figure 5 [file EMBR-24-e57300-s007.zip › Fig 5/5C/siDOK1_Flag_LLOMe_VPS4.tif]

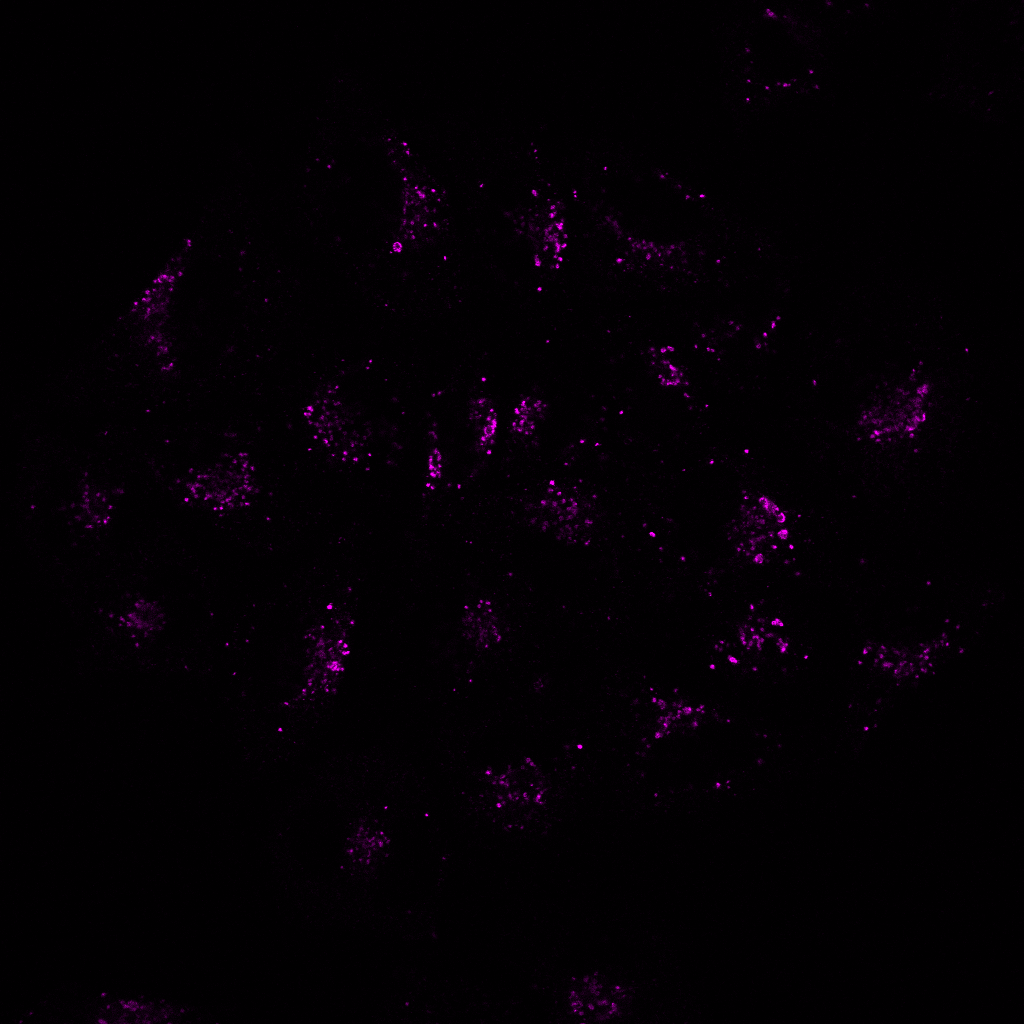

Supplement: Supplementary file 9 — Source Data for Figure 5 [file EMBR-24-e57300-s007.zip › Fig 5/5C/siControl_Flag_non-treated_LAMP1.tif]

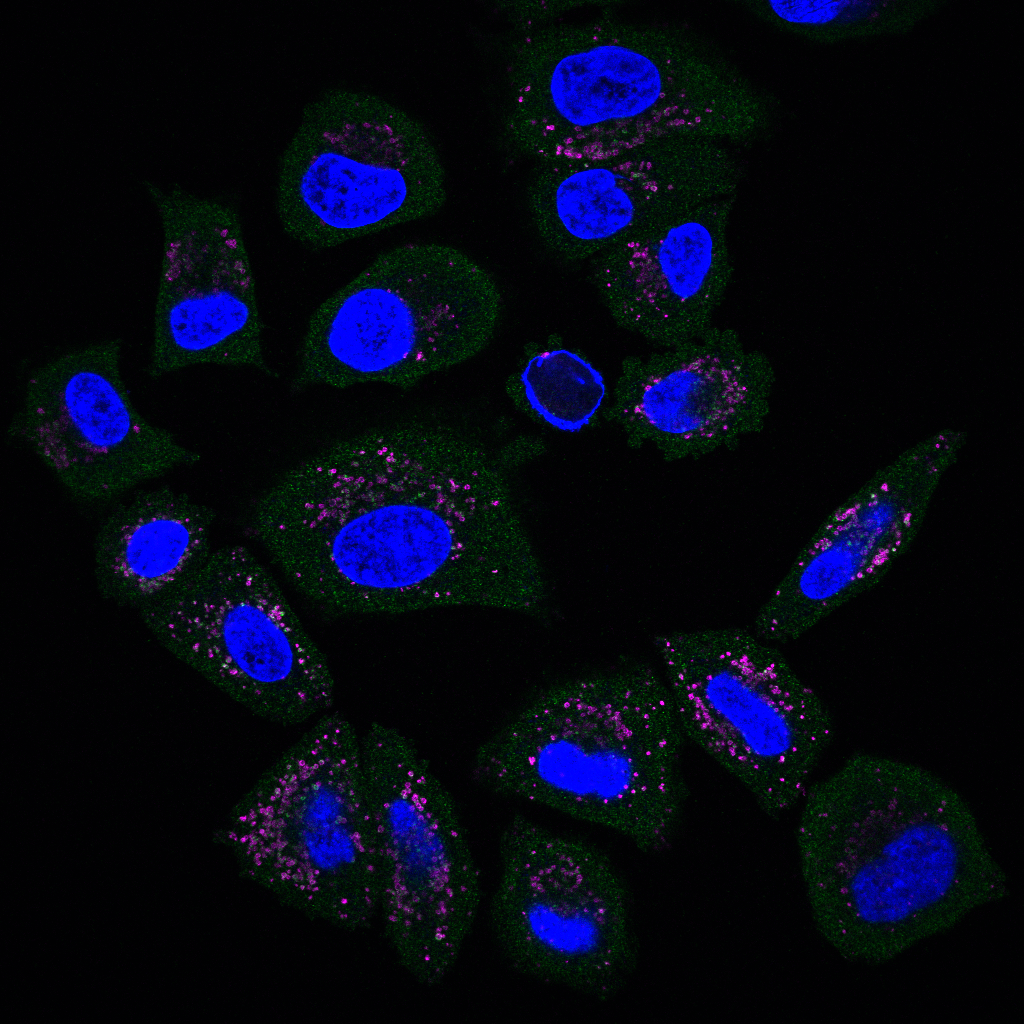

Supplement: Supplementary file 9 — Source Data for Figure 5 [file EMBR-24-e57300-s007.zip › Fig 5/5C/siDOK1_Flag_LLOMe_Merge+DAPI.tif]

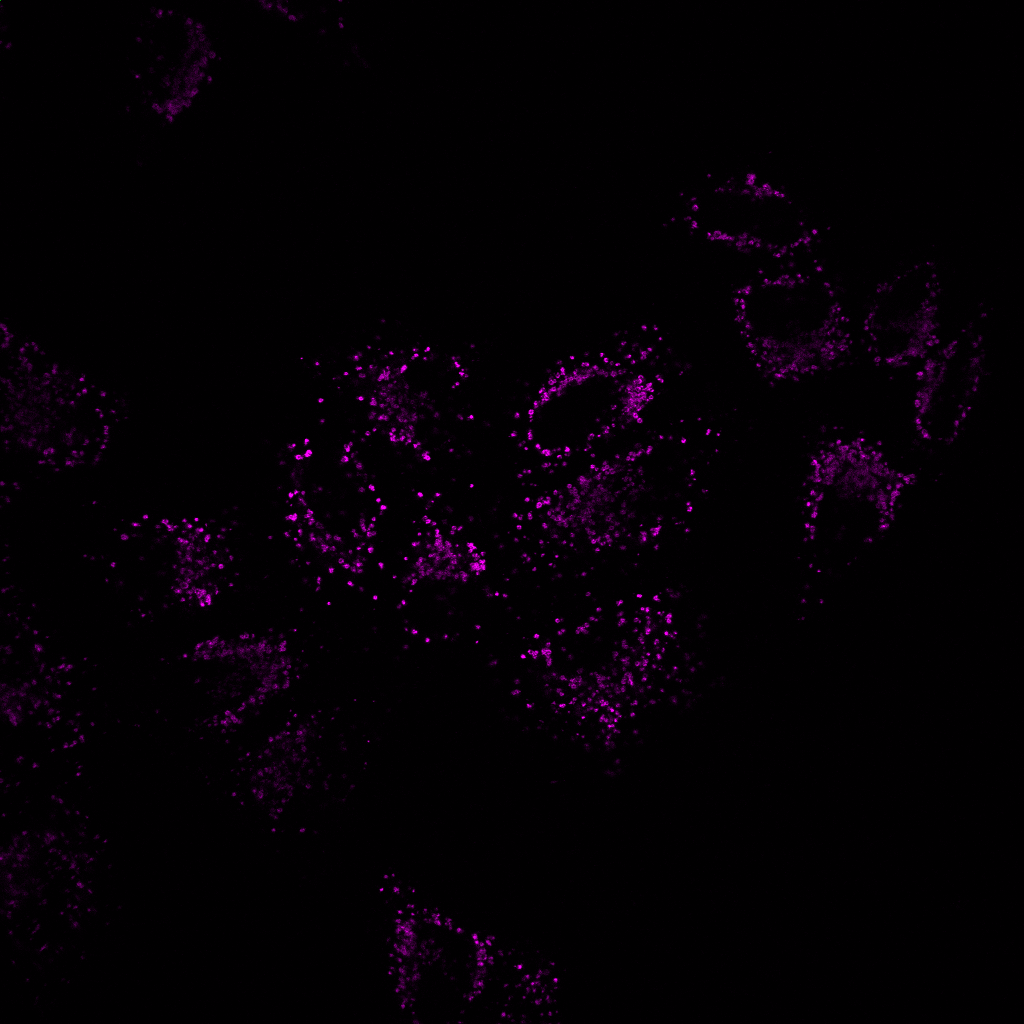

Supplement: Supplementary file 9 — Source Data for Figure 5 [file EMBR-24-e57300-s007.zip › Fig 5/5E/siLuc_LLOMe_LAMP1.tif]

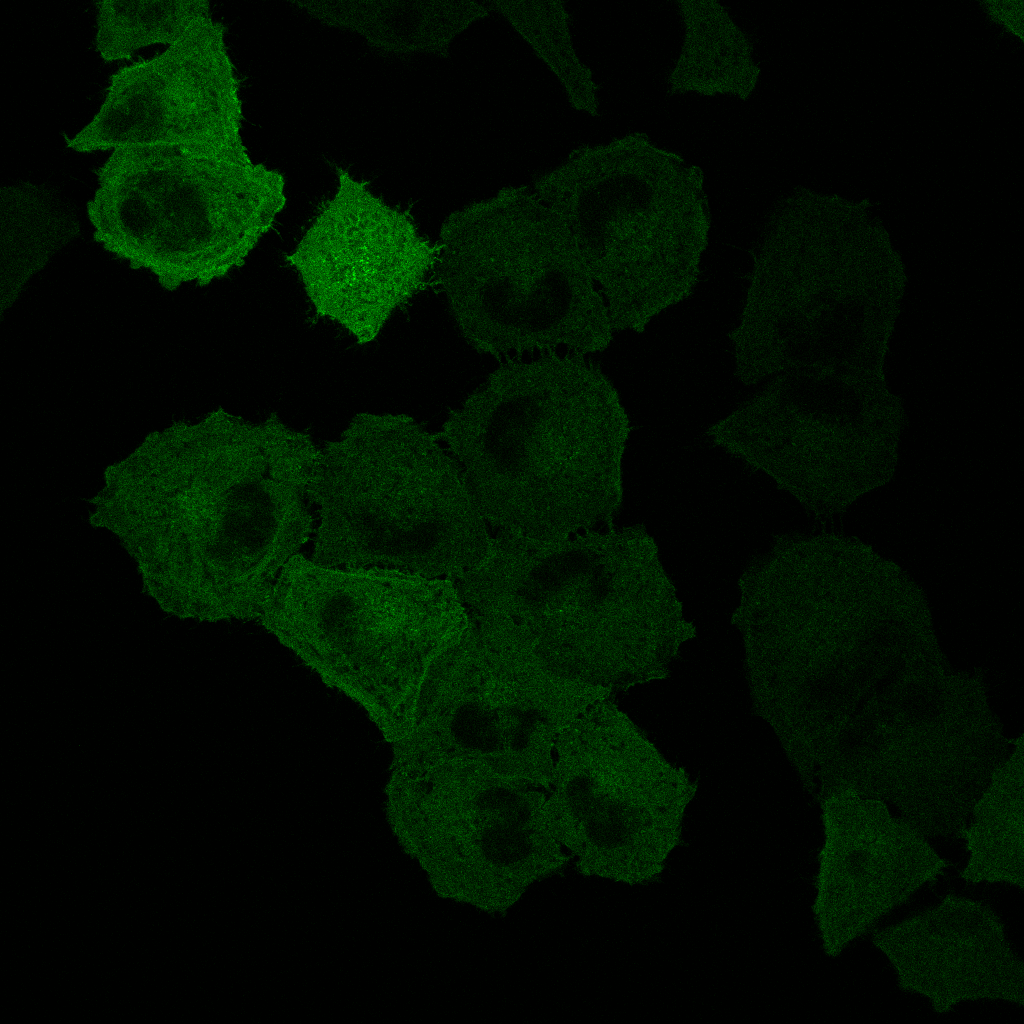

Supplement: Supplementary file 9 — Source Data for Figure 5 [file EMBR-24-e57300-s007.zip › Fig 5/5E/siSTK38 #1_non-treated_mNG-DOK1.tif]

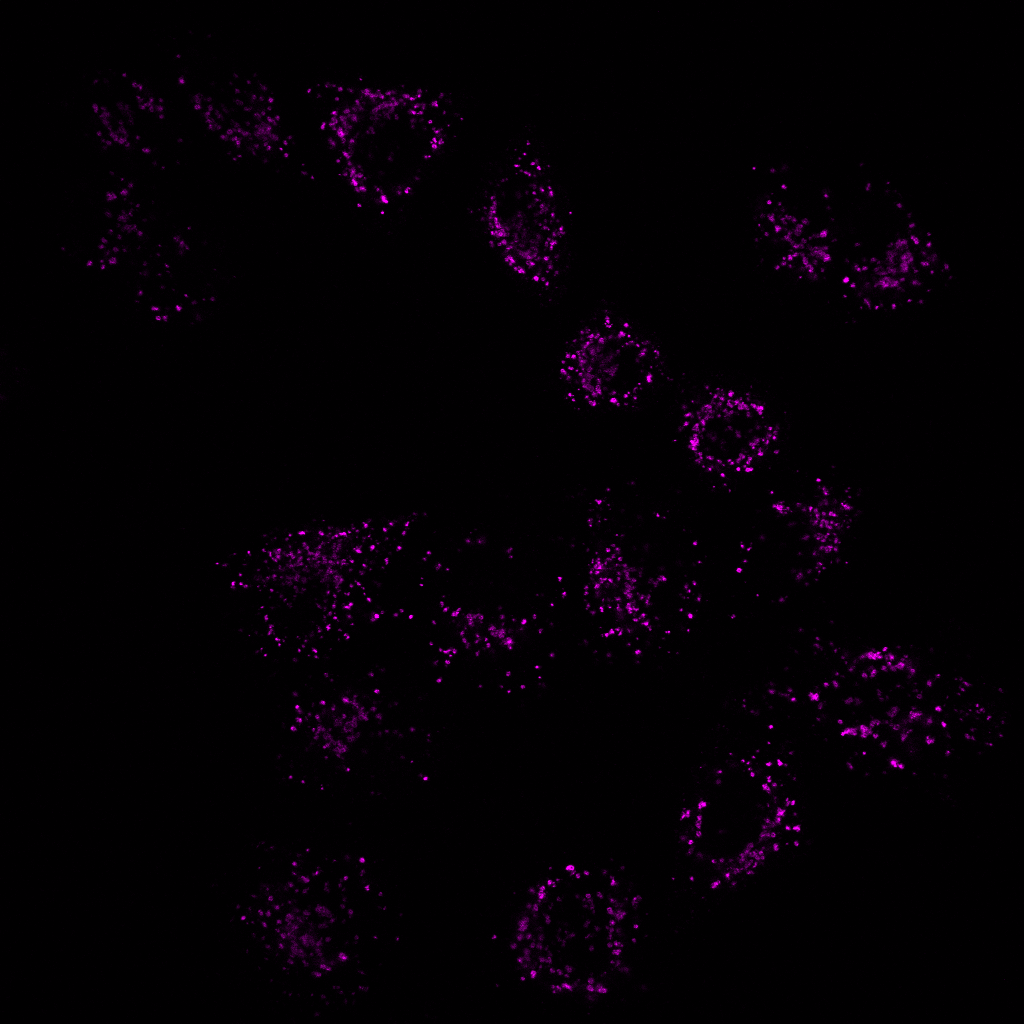

Supplement: Supplementary file 9 — Source Data for Figure 5 [file EMBR-24-e57300-s007.zip › Fig 5/5E/siSTK38 #3_LLOMe_LAMP1.tif]

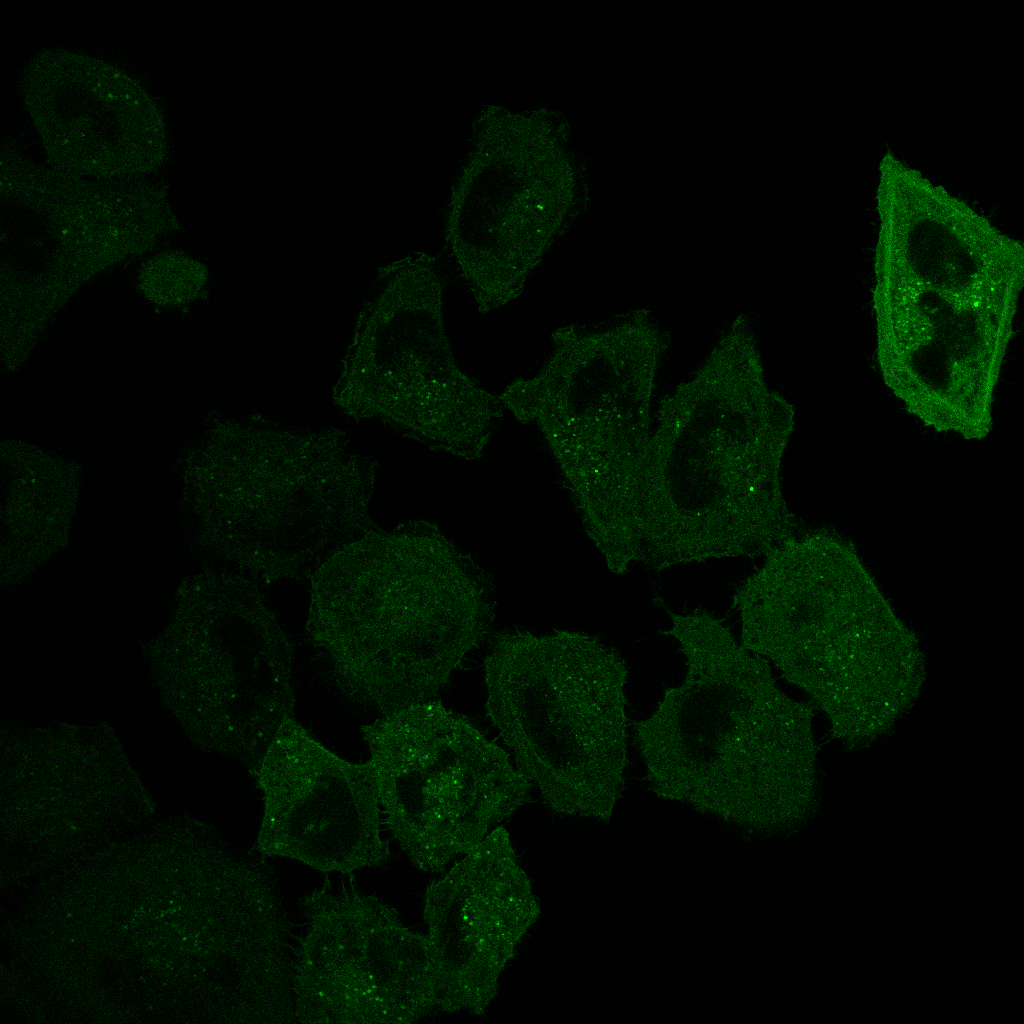

Supplement: Supplementary file 9 — Source Data for Figure 5 [file EMBR-24-e57300-s007.zip › Fig 5/5E/siSTK38 #1_LLOMe_mNG-DOK1.tif]

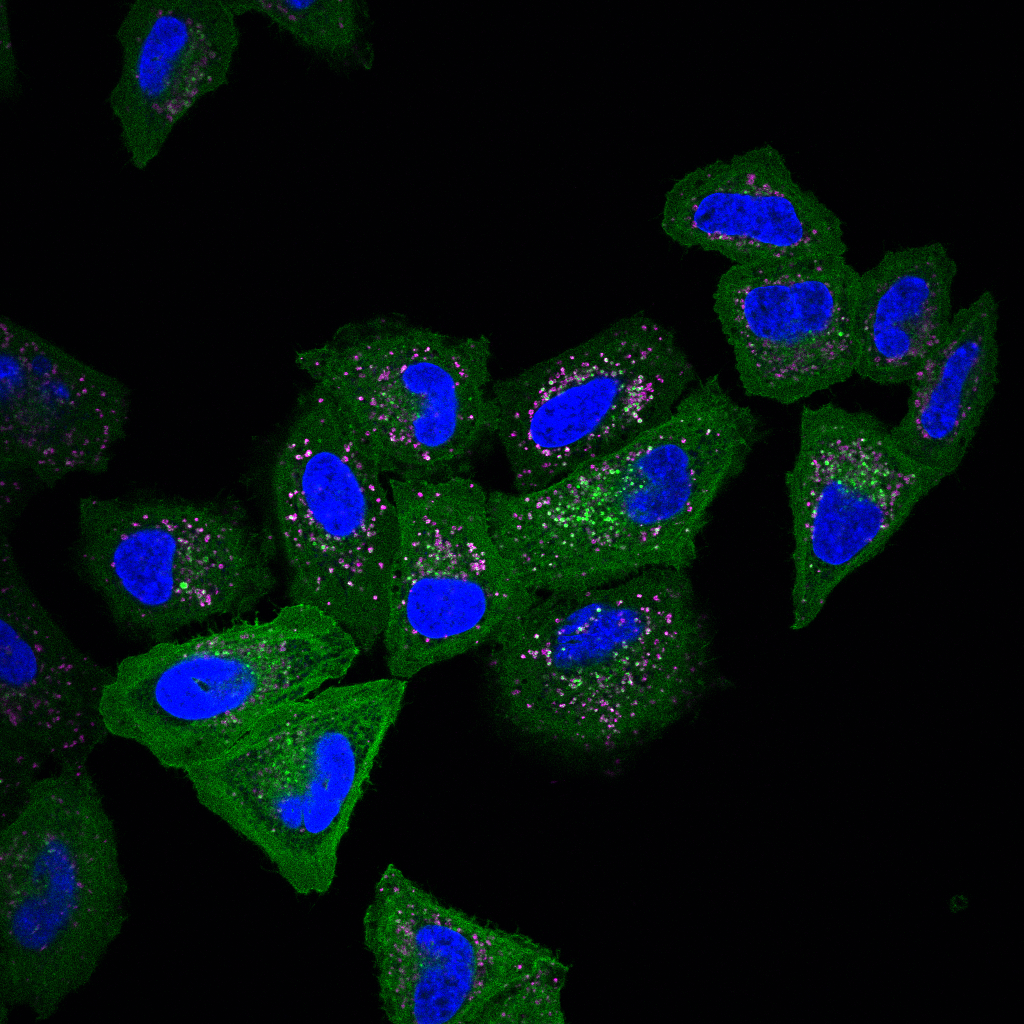

Supplement: Supplementary file 9 — Source Data for Figure 5 [file EMBR-24-e57300-s007.zip › Fig 5/5E/siLuc_LLOMe_Merge+DAPI.tif]

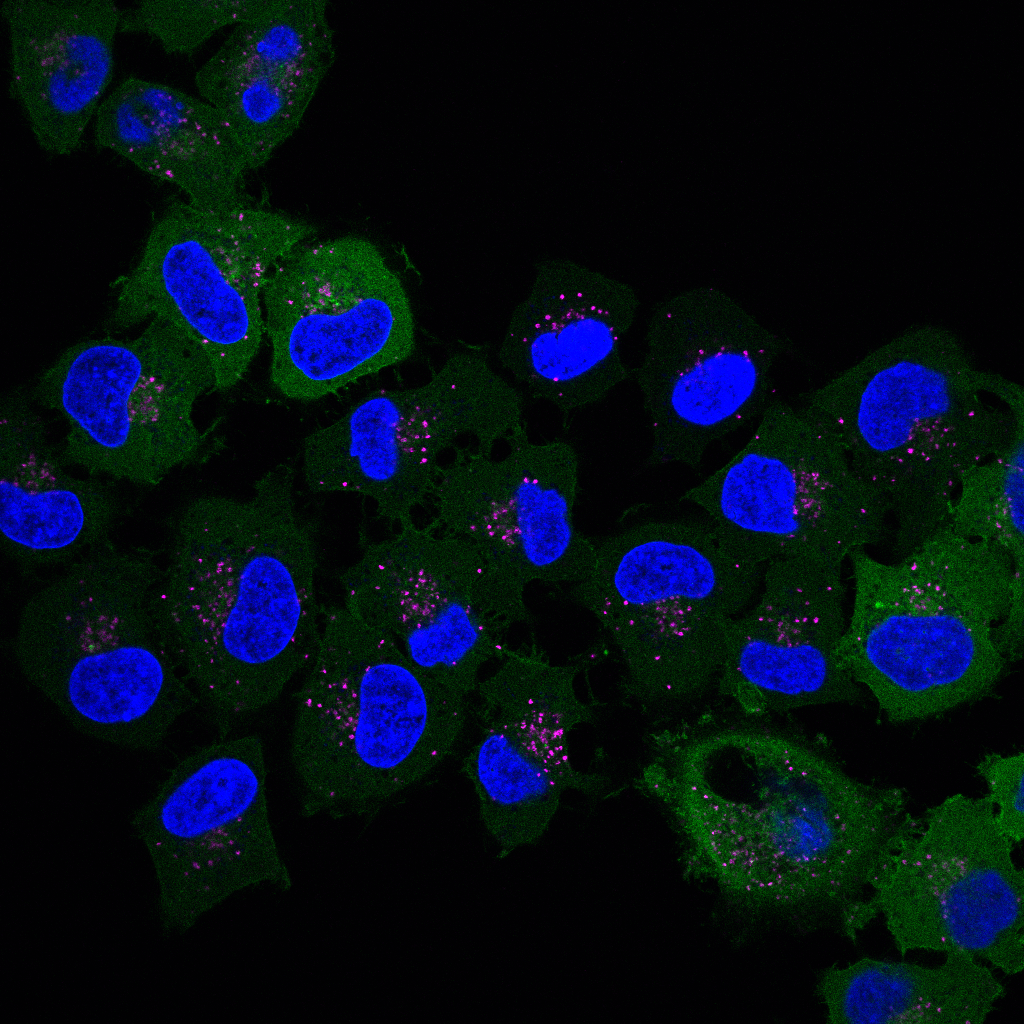

Supplement: Supplementary file 9 — Source Data for Figure 5 [file EMBR-24-e57300-s007.zip › Fig 5/5E/siSTK38 #3_non-treated_Merge+DAPI.tif]

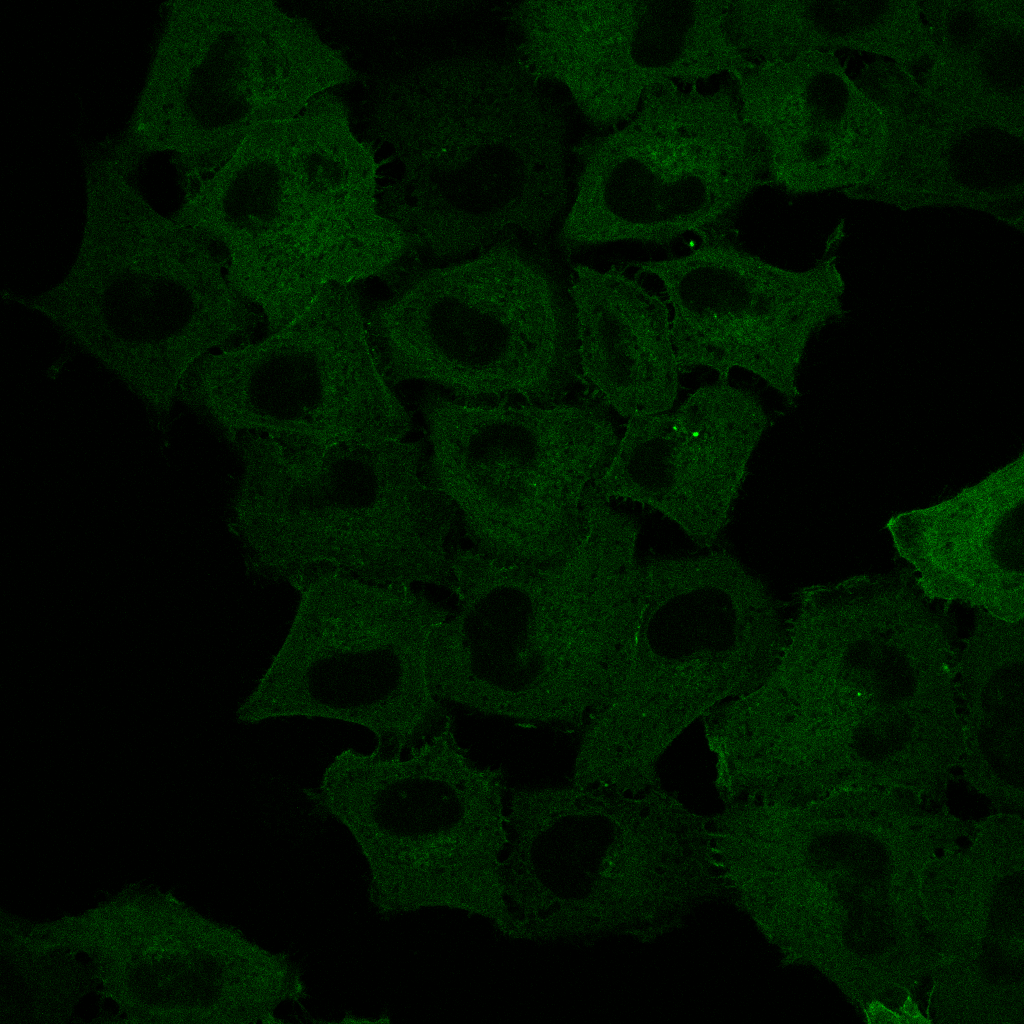

Supplement: Supplementary file 9 — Source Data for Figure 5 [file EMBR-24-e57300-s007.zip › Fig 5/5E/siLuc_non-treated_mNG-DOK1.tif]

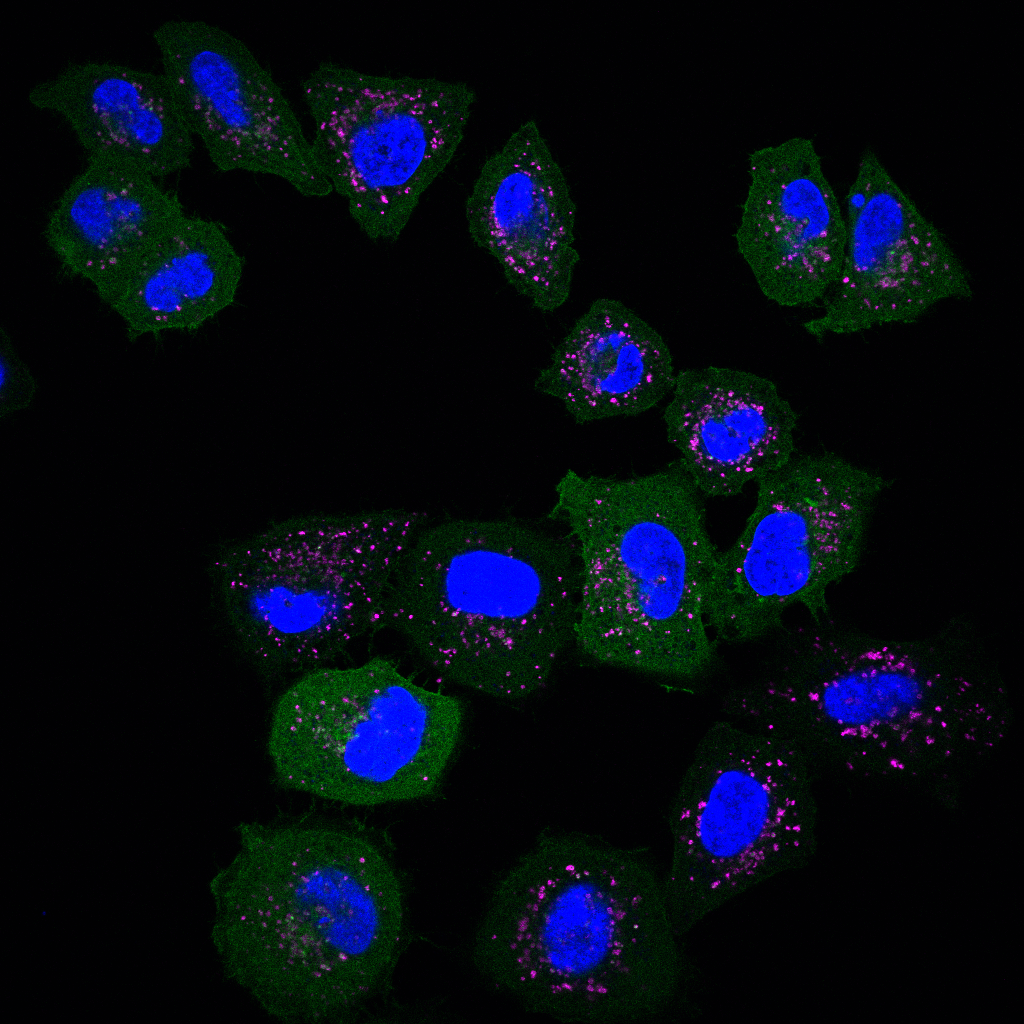

Supplement: Supplementary file 9 — Source Data for Figure 5 [file EMBR-24-e57300-s007.zip › Fig 5/5E/siSTK38 #3_LLOMe_Merge+DAPI.tif]

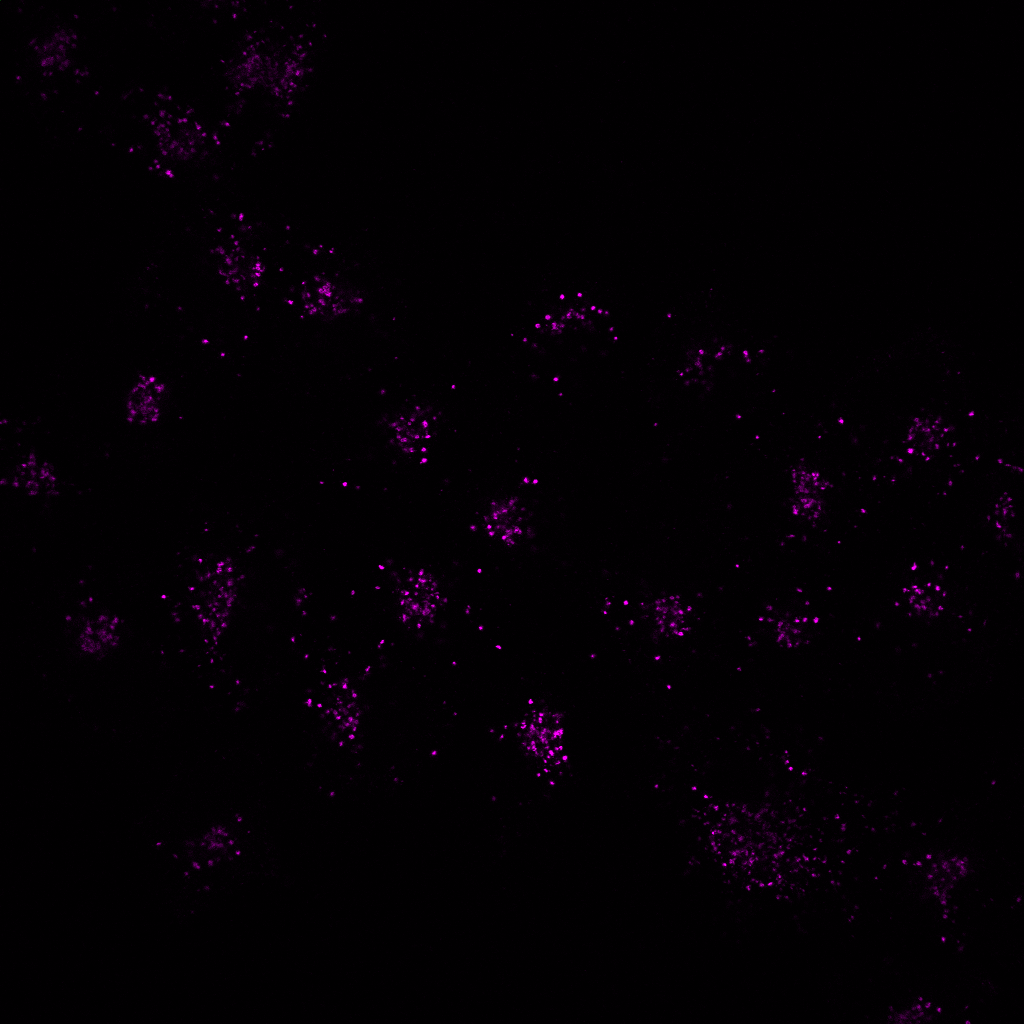

Supplement: Supplementary file 9 — Source Data for Figure 5 [file EMBR-24-e57300-s007.zip › Fig 5/5E/siSTK38 #3_non-treated_LAMP1.tif]

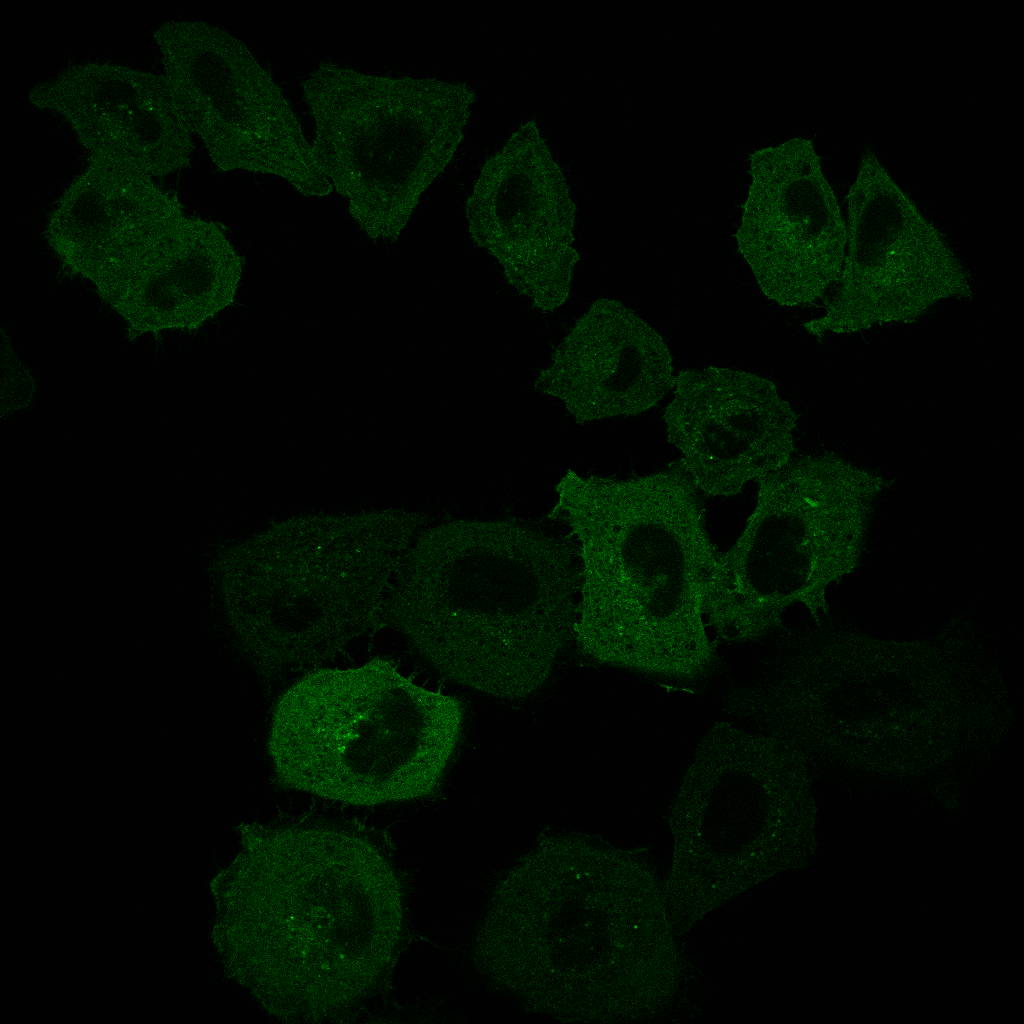

Supplement: Supplementary file 9 — Source Data for Figure 5 [file EMBR-24-e57300-s007.zip › Fig 5/5E/siSTK38 #3_LLOMe_mNG-DOK1.tif]

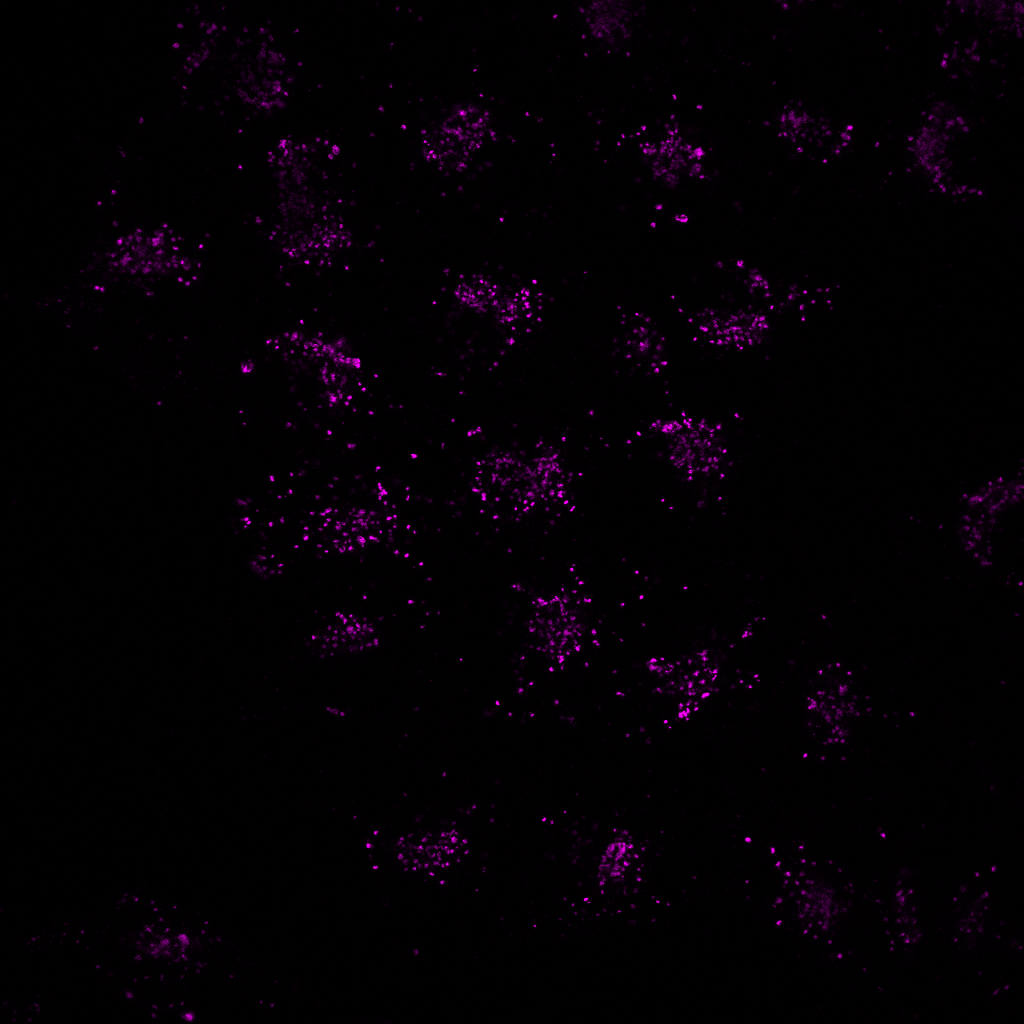

Supplement: Supplementary file 9 — Source Data for Figure 5 [file EMBR-24-e57300-s007.zip › Fig 5/5E/siLuc_non-treated_LAMP1.tif]

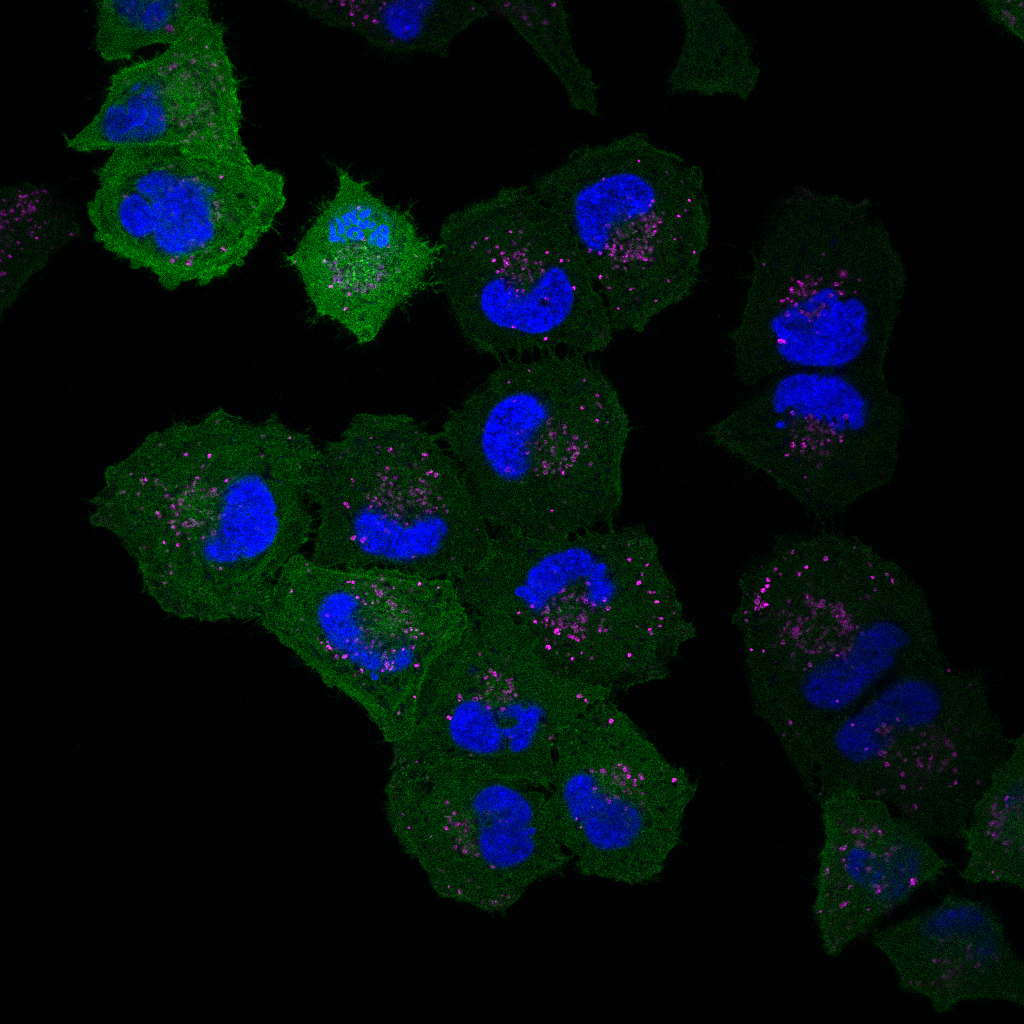

Supplement: Supplementary file 9 — Source Data for Figure 5 [file EMBR-24-e57300-s007.zip › Fig 5/5E/siSTK38 #1_non-treated_Merge+DAPI.tif]

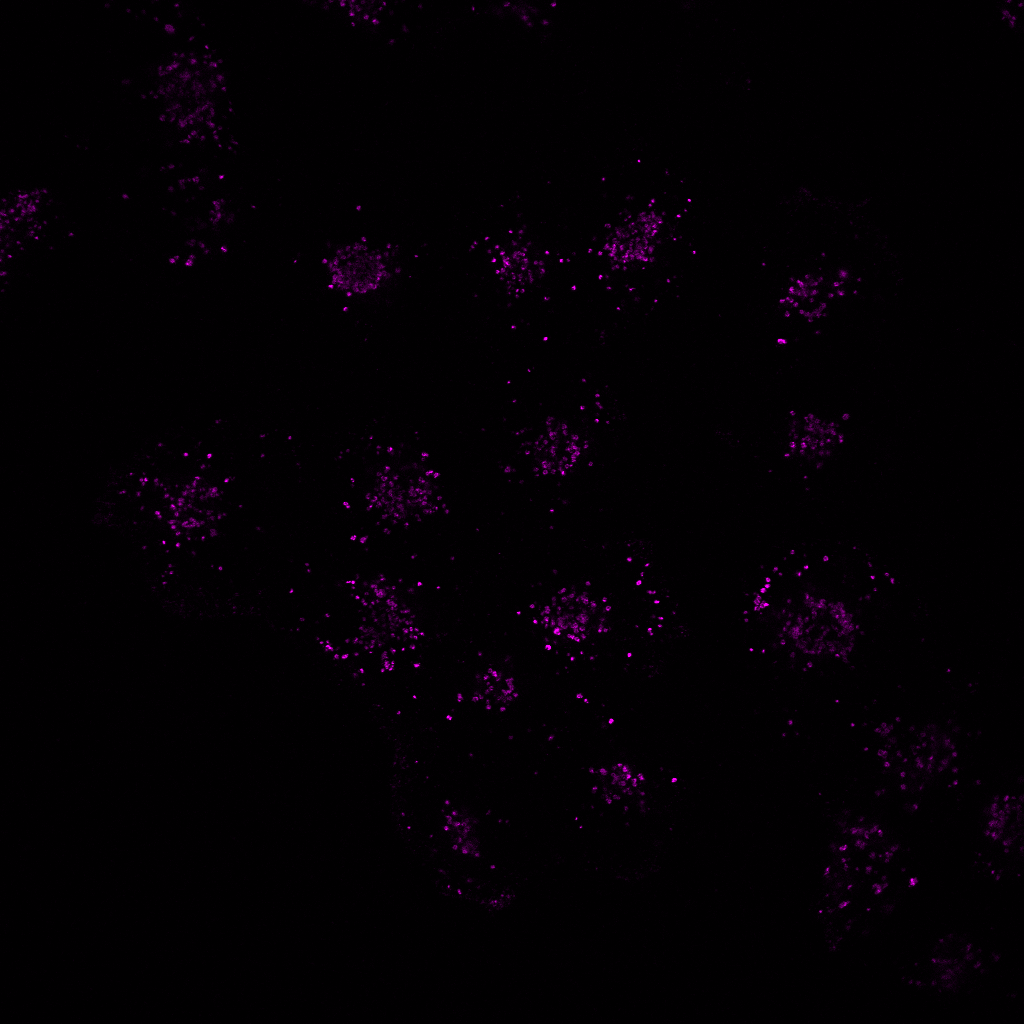

Supplement: Supplementary file 9 — Source Data for Figure 5 [file EMBR-24-e57300-s007.zip › Fig 5/5E/siSTK38 #1_non-treated_LAMP1.tif]

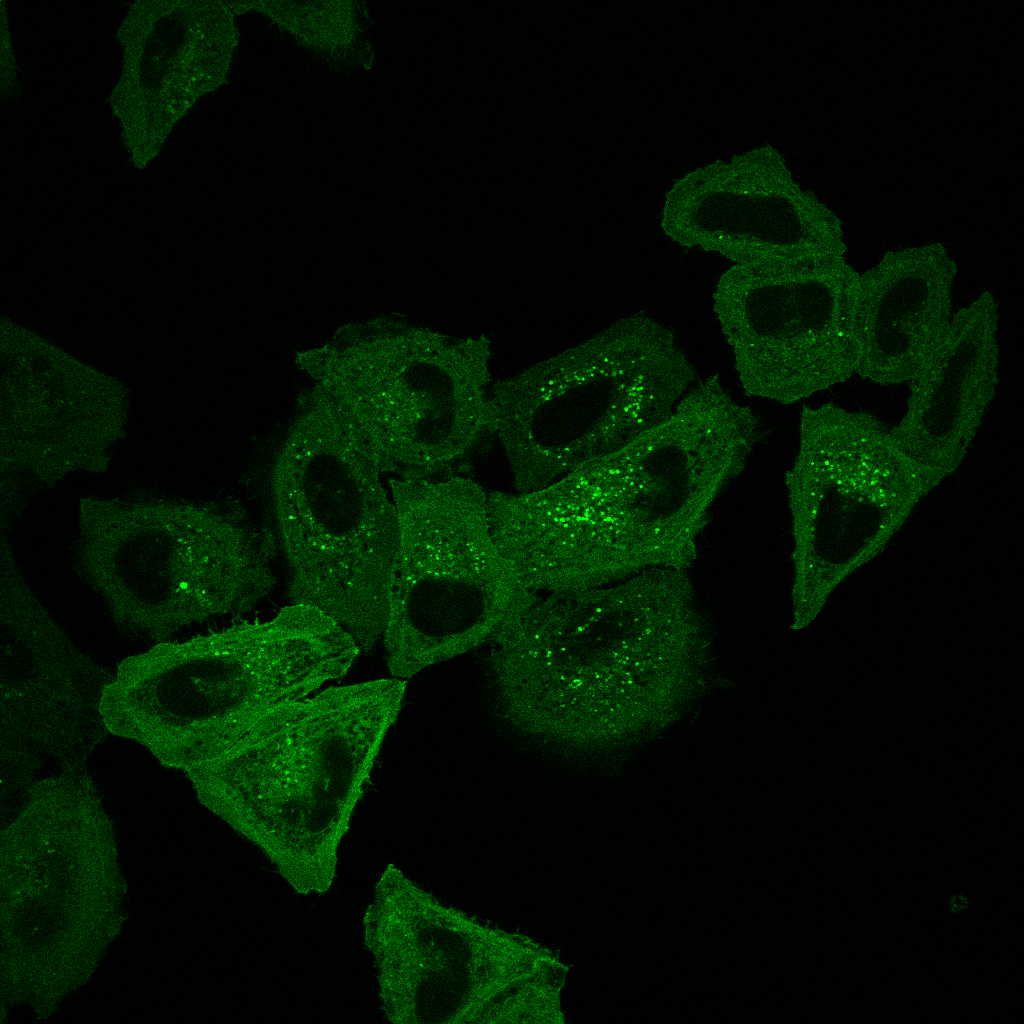

Supplement: Supplementary file 9 — Source Data for Figure 5 [file EMBR-24-e57300-s007.zip › Fig 5/5E/siLuc_LLOMe_mNG-DOK1.tif]

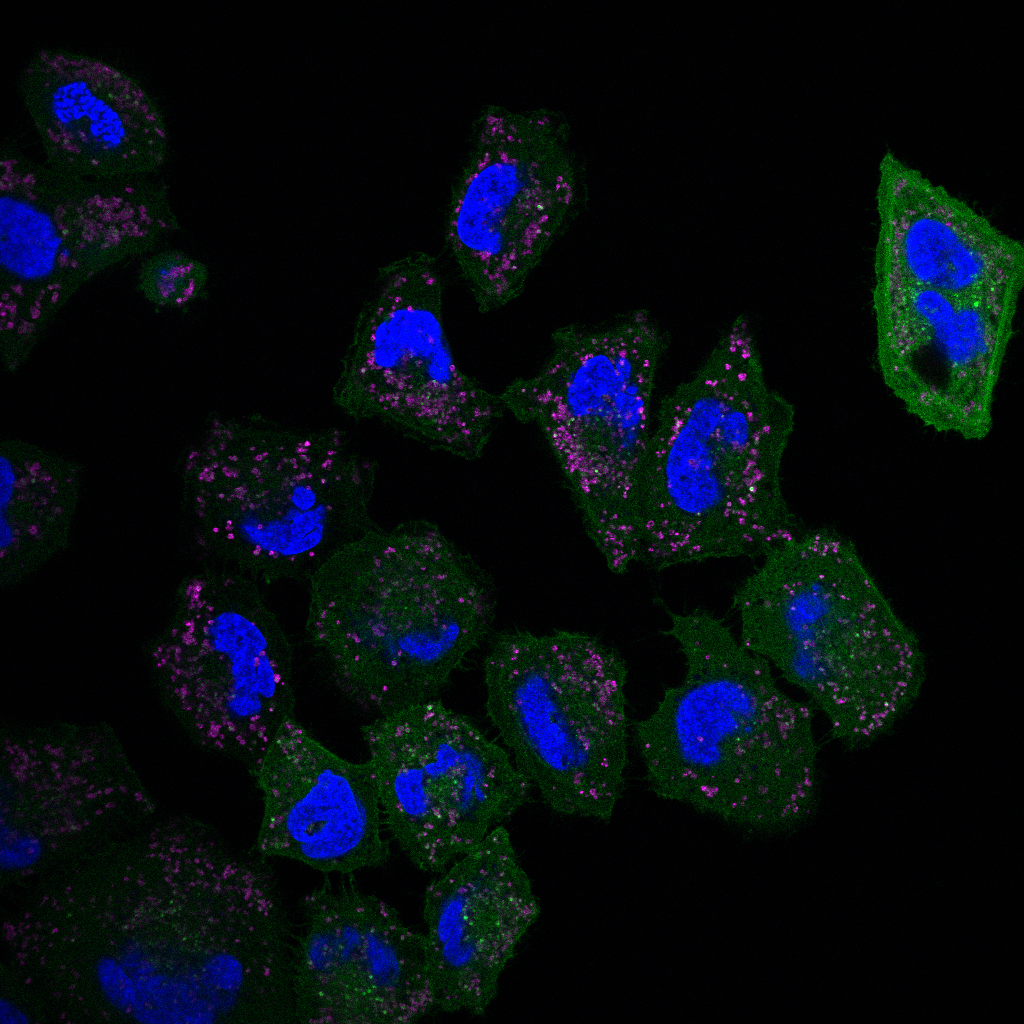

Supplement: Supplementary file 9 — Source Data for Figure 5 [file EMBR-24-e57300-s007.zip › Fig 5/5E/siSTK38 #1_LLOMe_Merge+DAPI.tif]

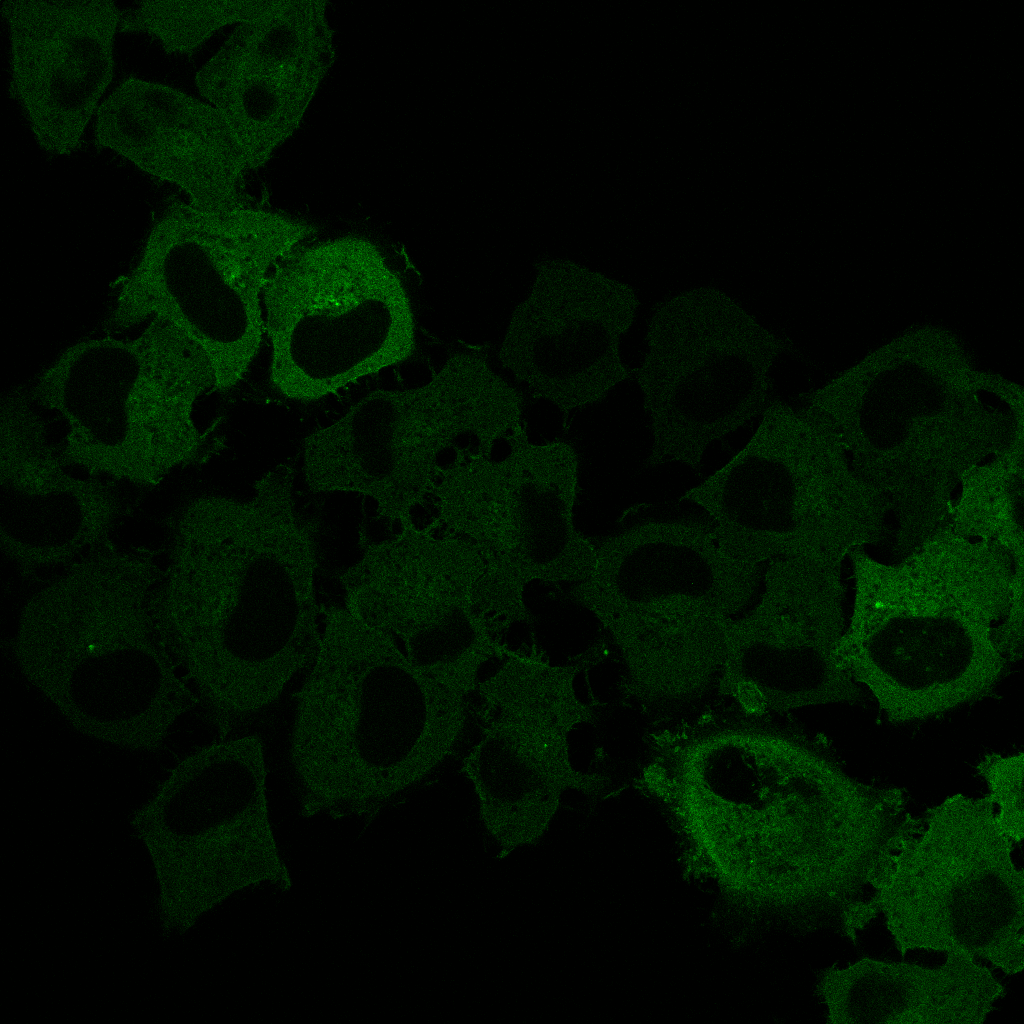

Supplement: Supplementary file 9 — Source Data for Figure 5 [file EMBR-24-e57300-s007.zip › Fig 5/5E/siSTK38 #3_non-treated_mNG-DOK1.tif]

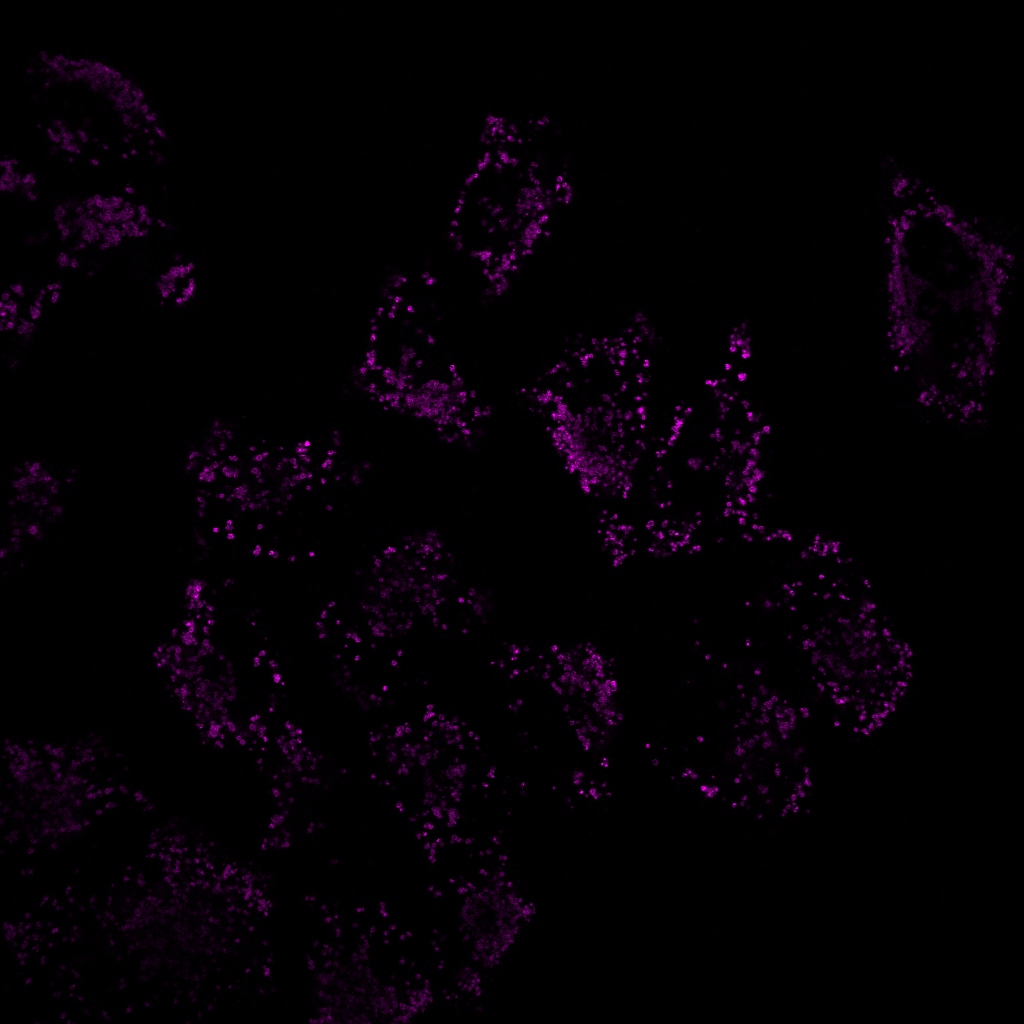

Supplement: Supplementary file 9 — Source Data for Figure 5 [file EMBR-24-e57300-s007.zip › Fig 5/5E/siSTK38 #1_LLOMe_LAMP1.tif]

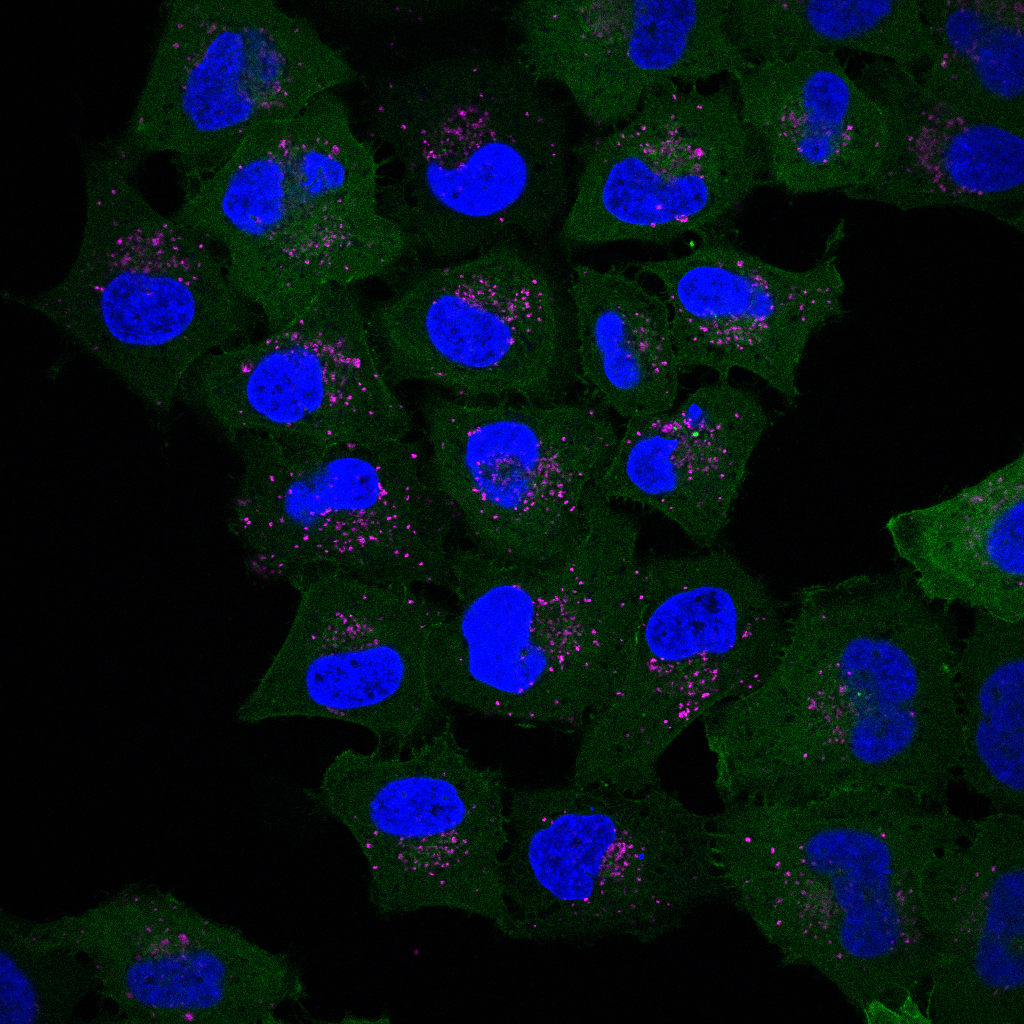

Supplement: Supplementary file 9 — Source Data for Figure 5 [file EMBR-24-e57300-s007.zip › Fig 5/5E/siLuc_non-treated_Merge+DAPI.tif]

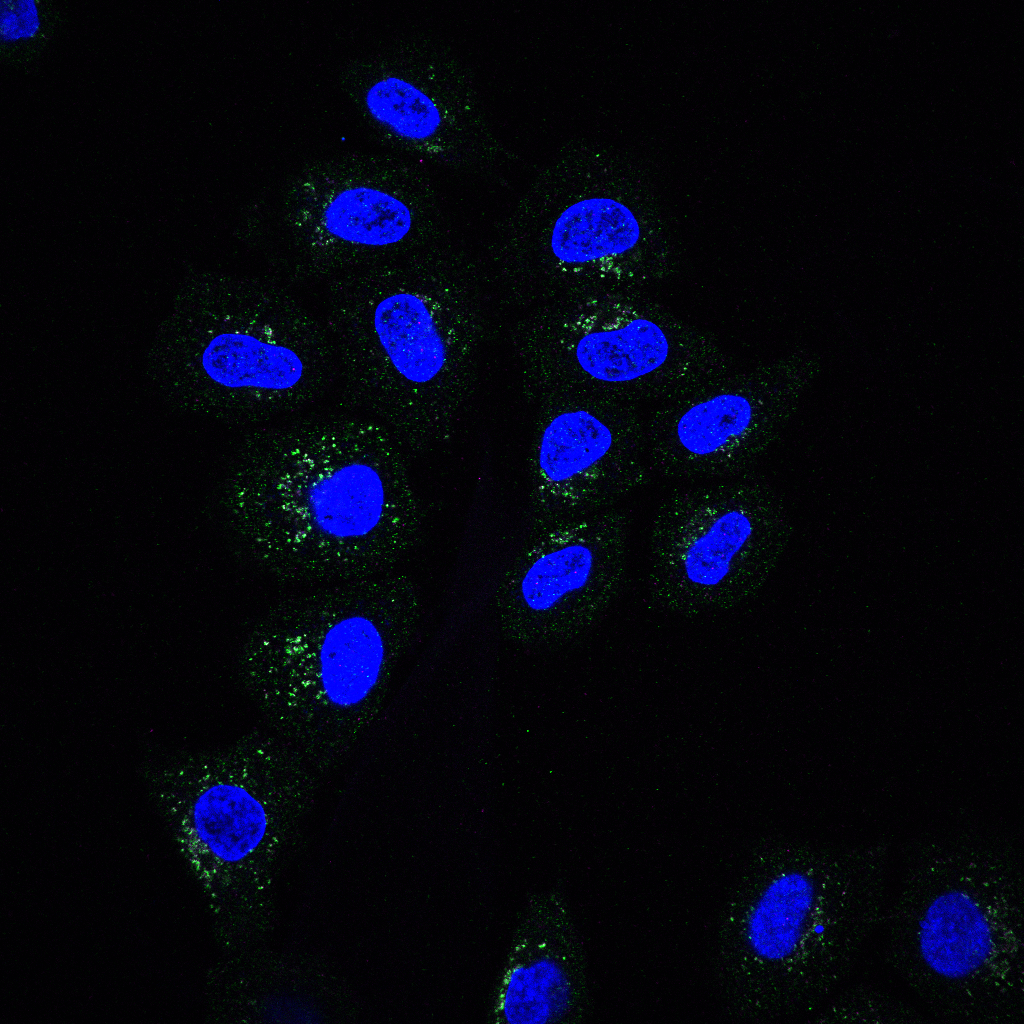

Supplement: Supplementary file 10 — Source Data for Figure 6 [file EMBR-24-e57300-s012.zip › Fig 6/6A/ATG16L1 KO_LLOMe.tif]

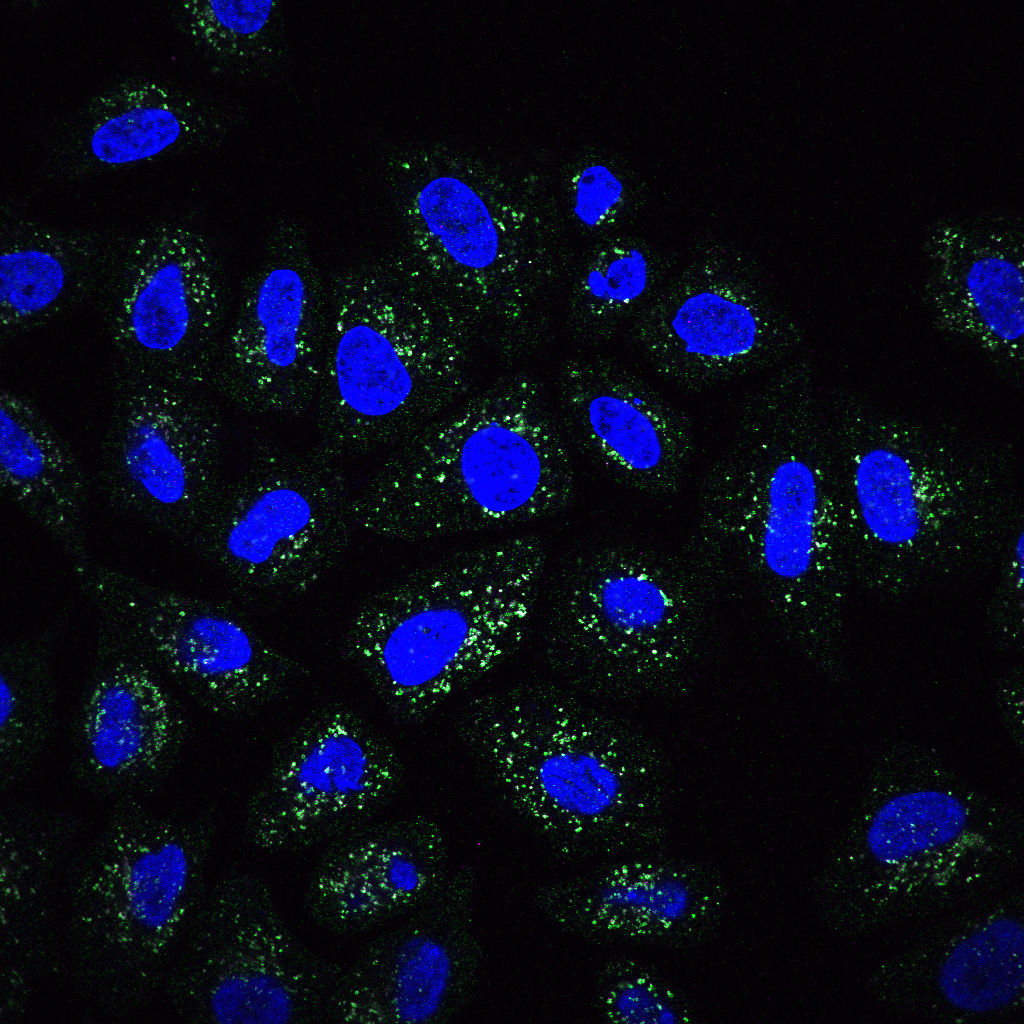

Supplement: Supplementary file 10 — Source Data for Figure 6 [file EMBR-24-e57300-s012.zip › Fig 6/6A/WT_LLOMe.tif]

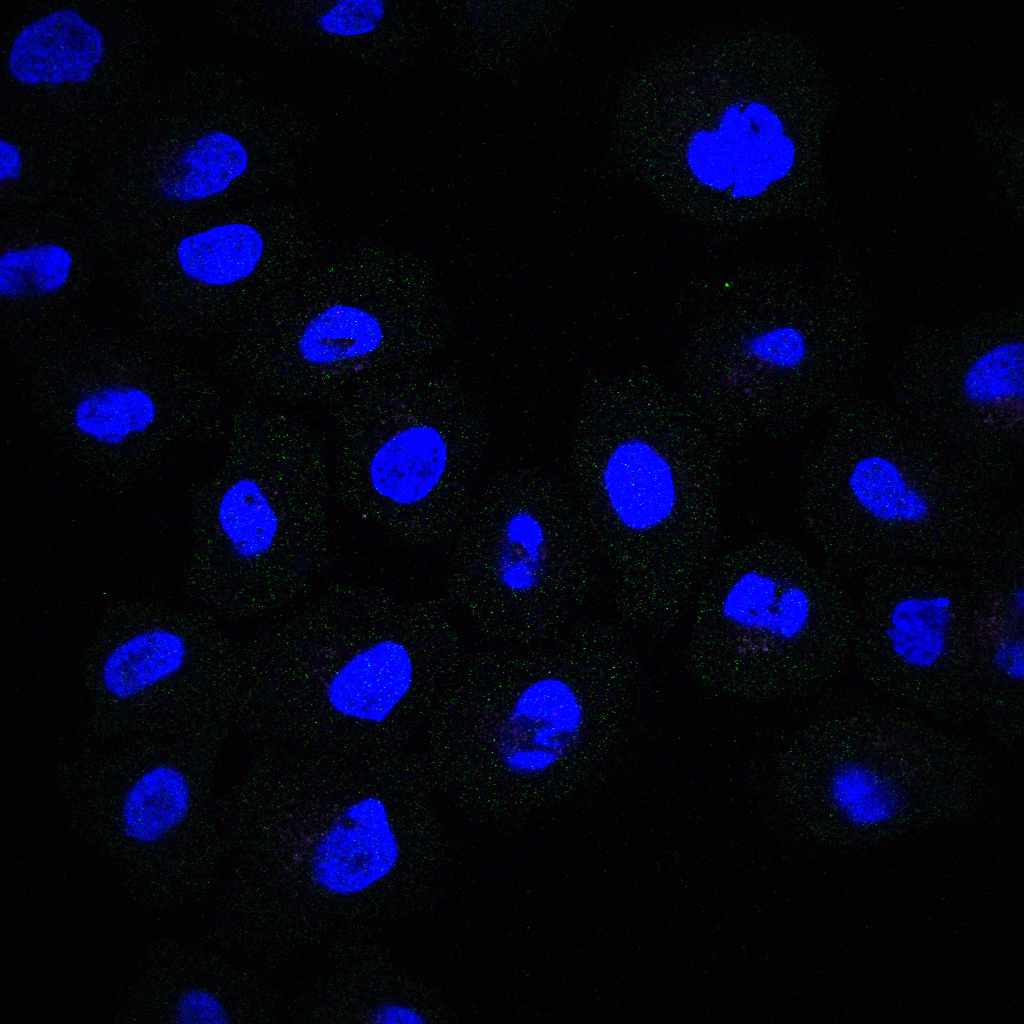

Supplement: Supplementary file 10 — Source Data for Figure 6 [file EMBR-24-e57300-s012.zip › Fig 6/6A/ATG16L1 KO_non-treated.tif]

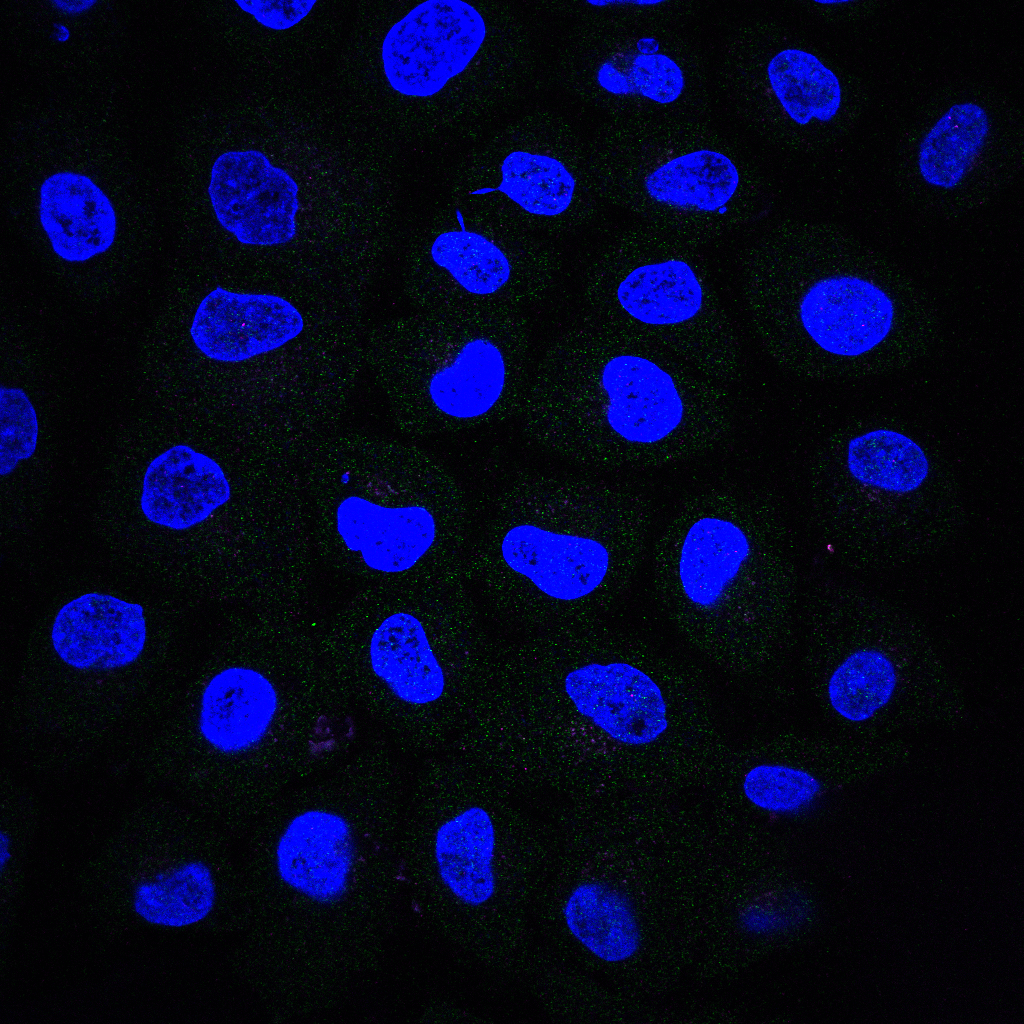

Supplement: Supplementary file 10 — Source Data for Figure 6 [file EMBR-24-e57300-s012.zip › Fig 6/6A/FIP200 KO_non-treated.tif]

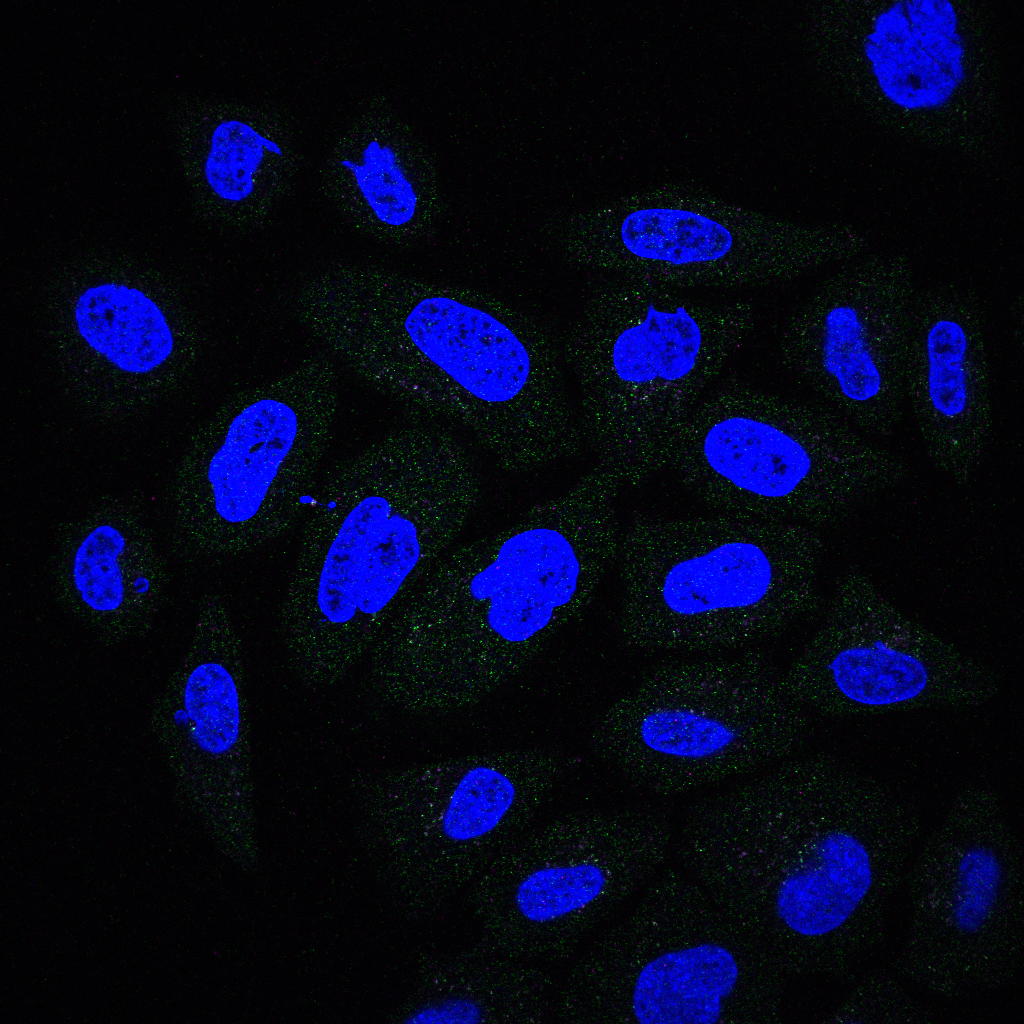

Supplement: Supplementary file 10 — Source Data for Figure 6 [file EMBR-24-e57300-s012.zip › Fig 6/6A/ATG7 KO_LLOMe.tif]

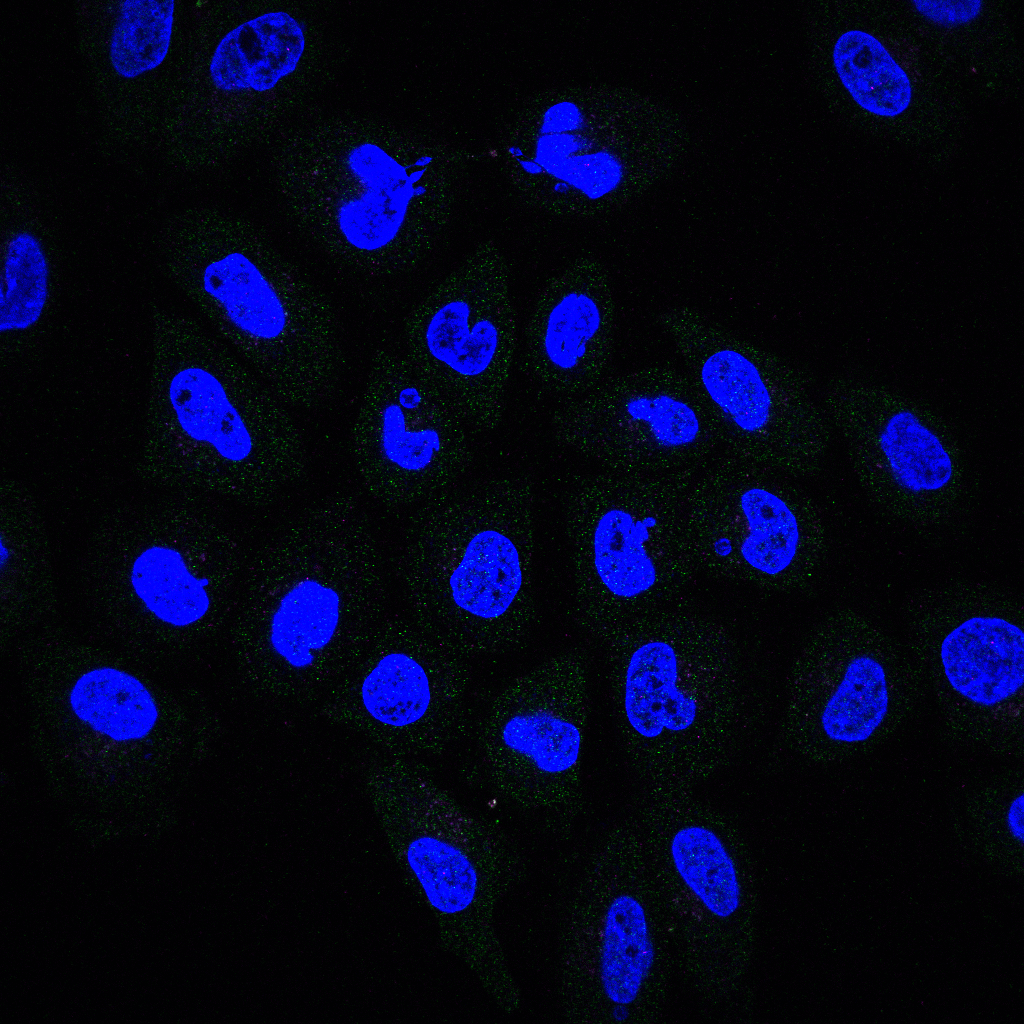

Supplement: Supplementary file 10 — Source Data for Figure 6 [file EMBR-24-e57300-s012.zip › Fig 6/6A/ATG7 KO_non-treated.tif]

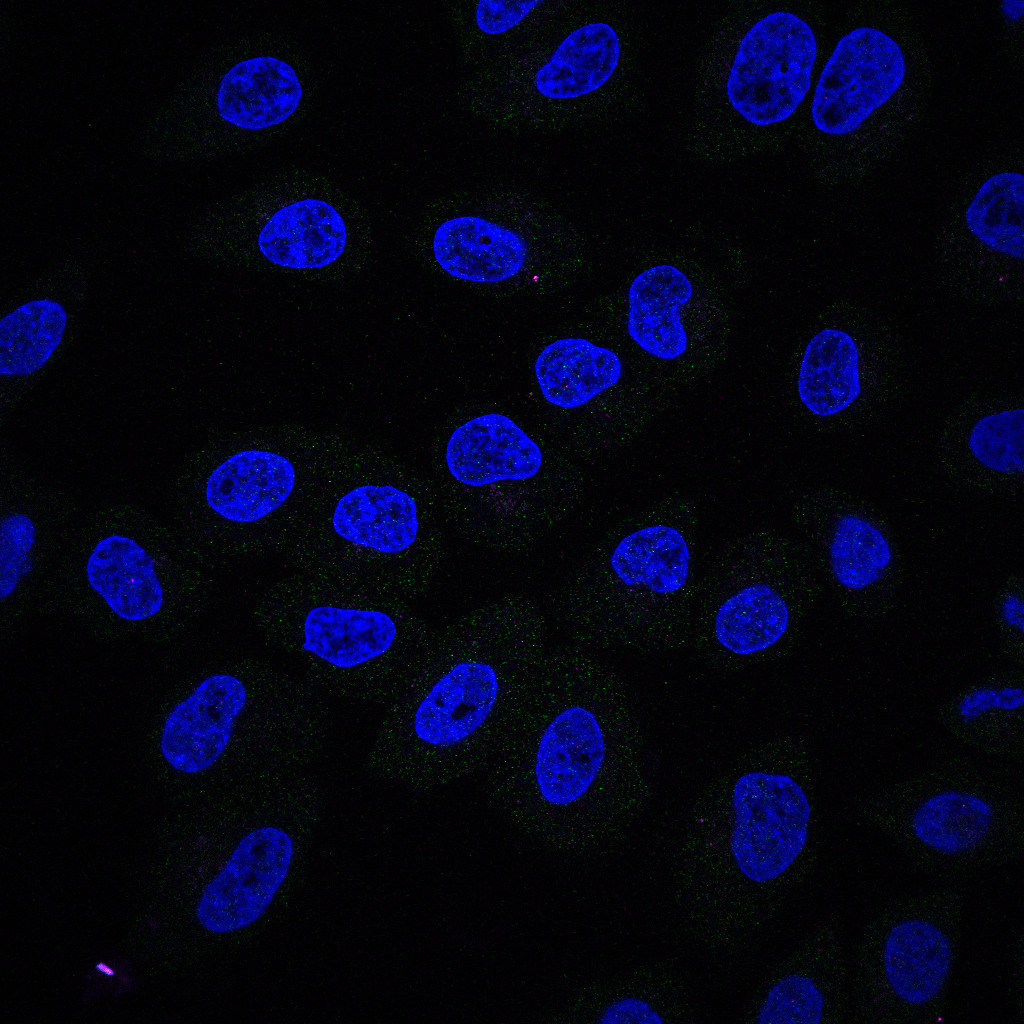

Supplement: Supplementary file 10 — Source Data for Figure 6 [file EMBR-24-e57300-s012.zip › Fig 6/6A/WT_non-treated.tif]

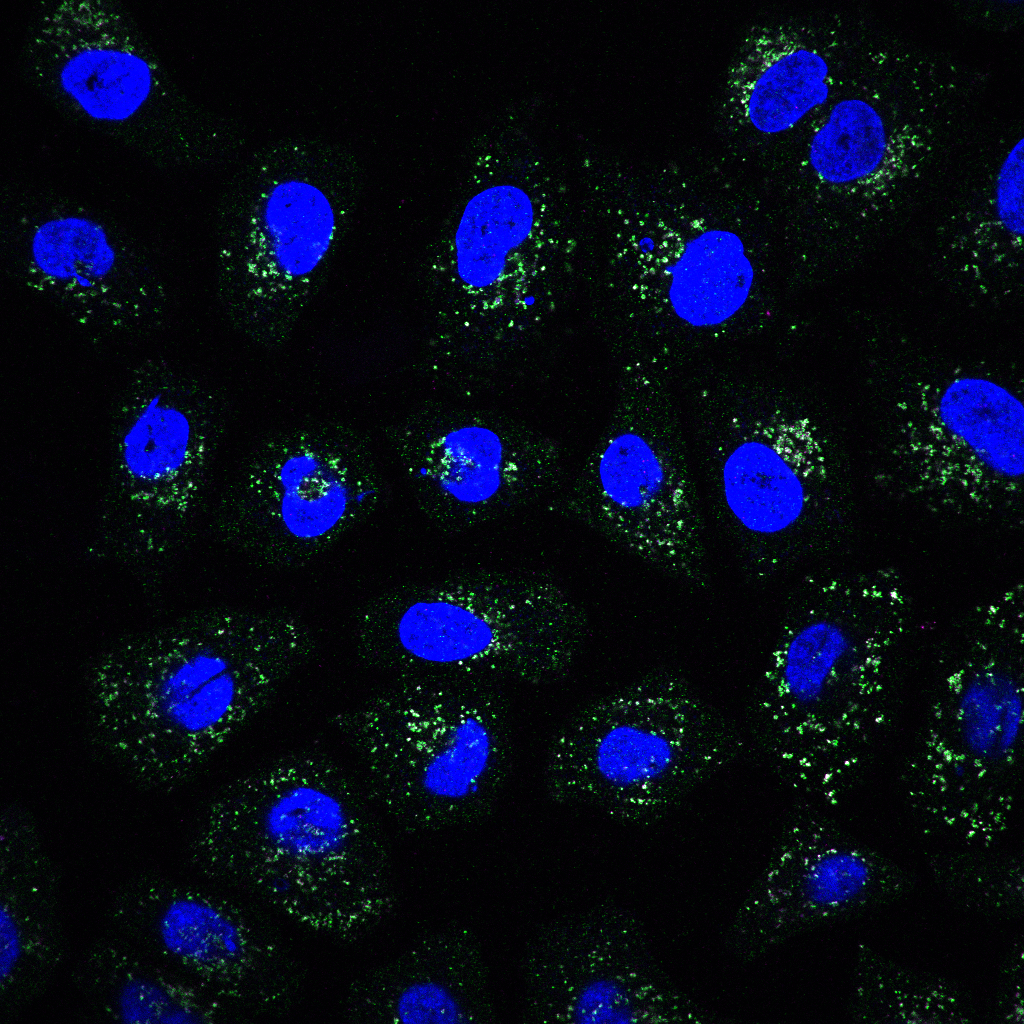

Supplement: Supplementary file 10 — Source Data for Figure 6 [file EMBR-24-e57300-s012.zip › Fig 6/6A/FIP200 KO_LLOMe.tif]

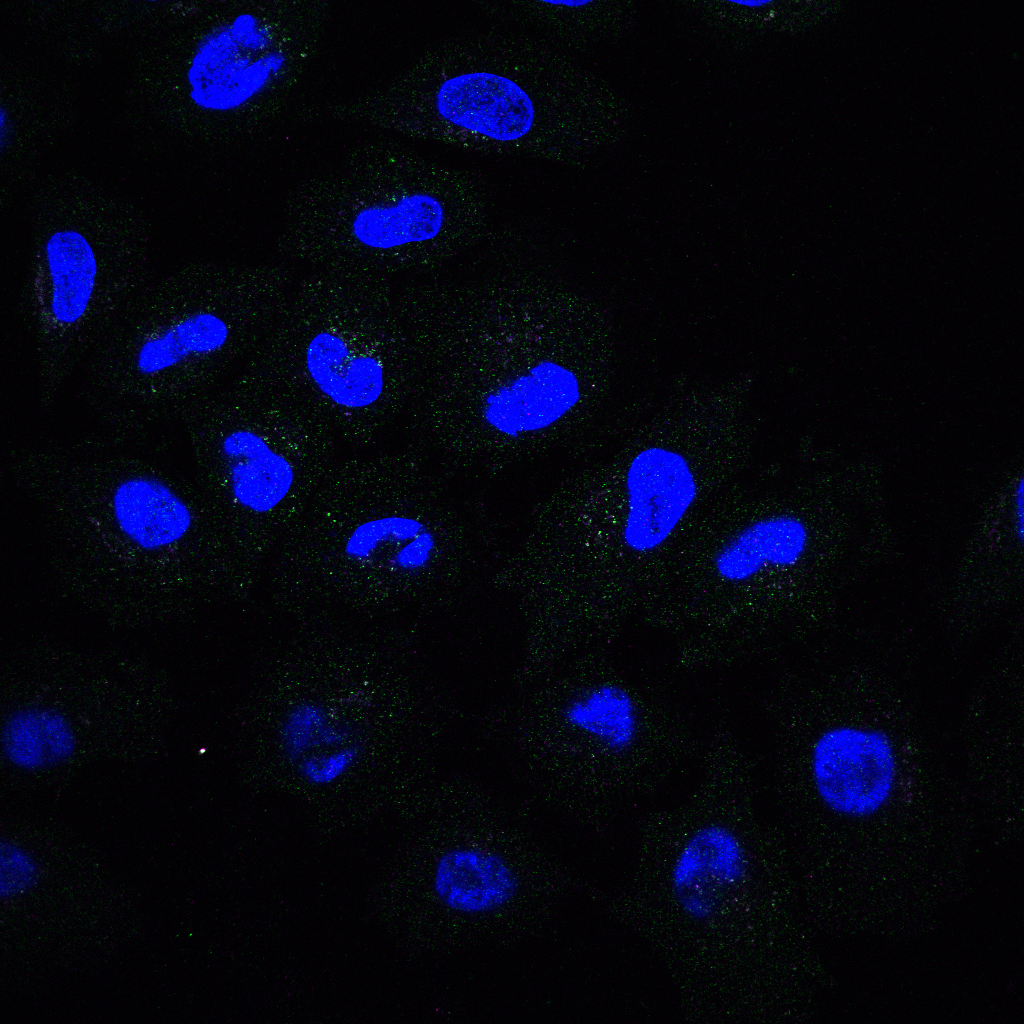

Supplement: Supplementary file 10 — Source Data for Figure 6 [file EMBR-24-e57300-s012.zip › Fig 6/6A/ATG3 KO_LLOMe.tif]

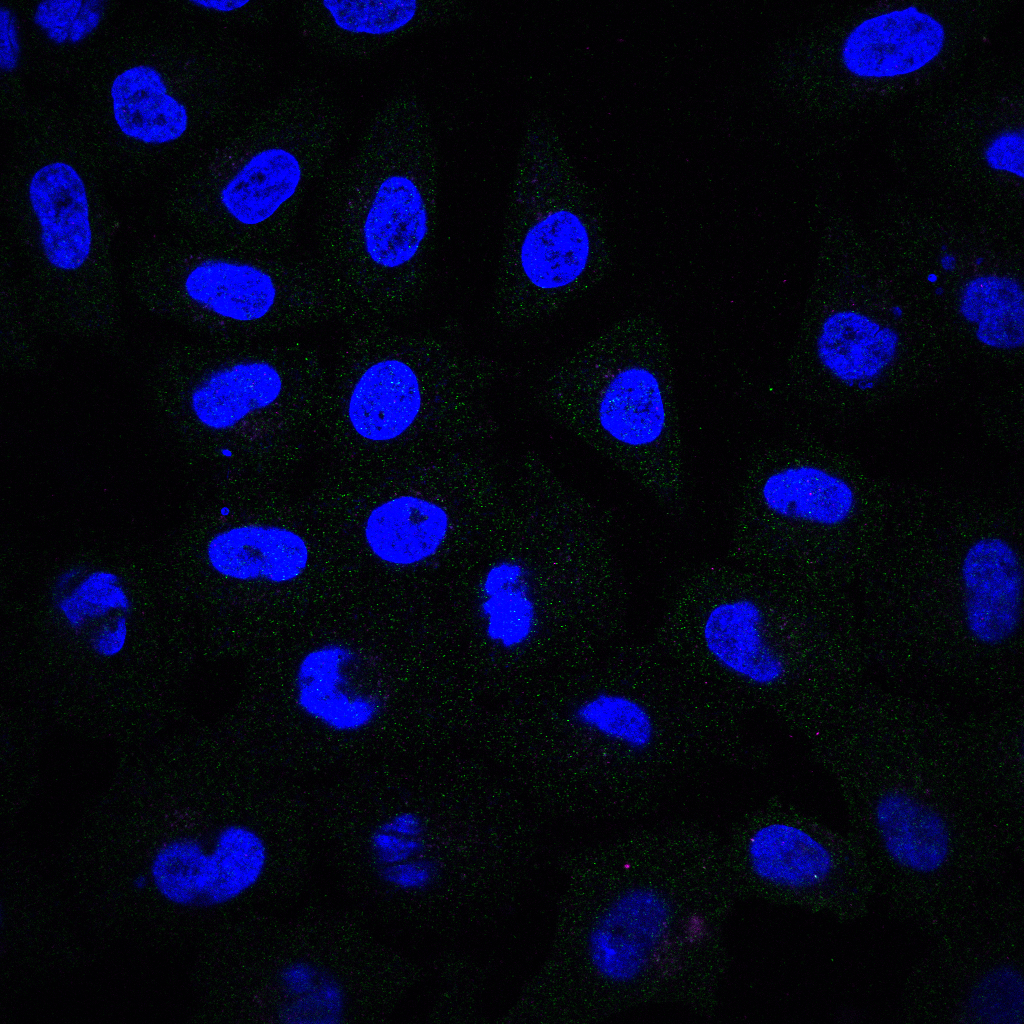

Supplement: Supplementary file 10 — Source Data for Figure 6 [file EMBR-24-e57300-s012.zip › Fig 6/6A/ATG3 KO_non-treated.tif]

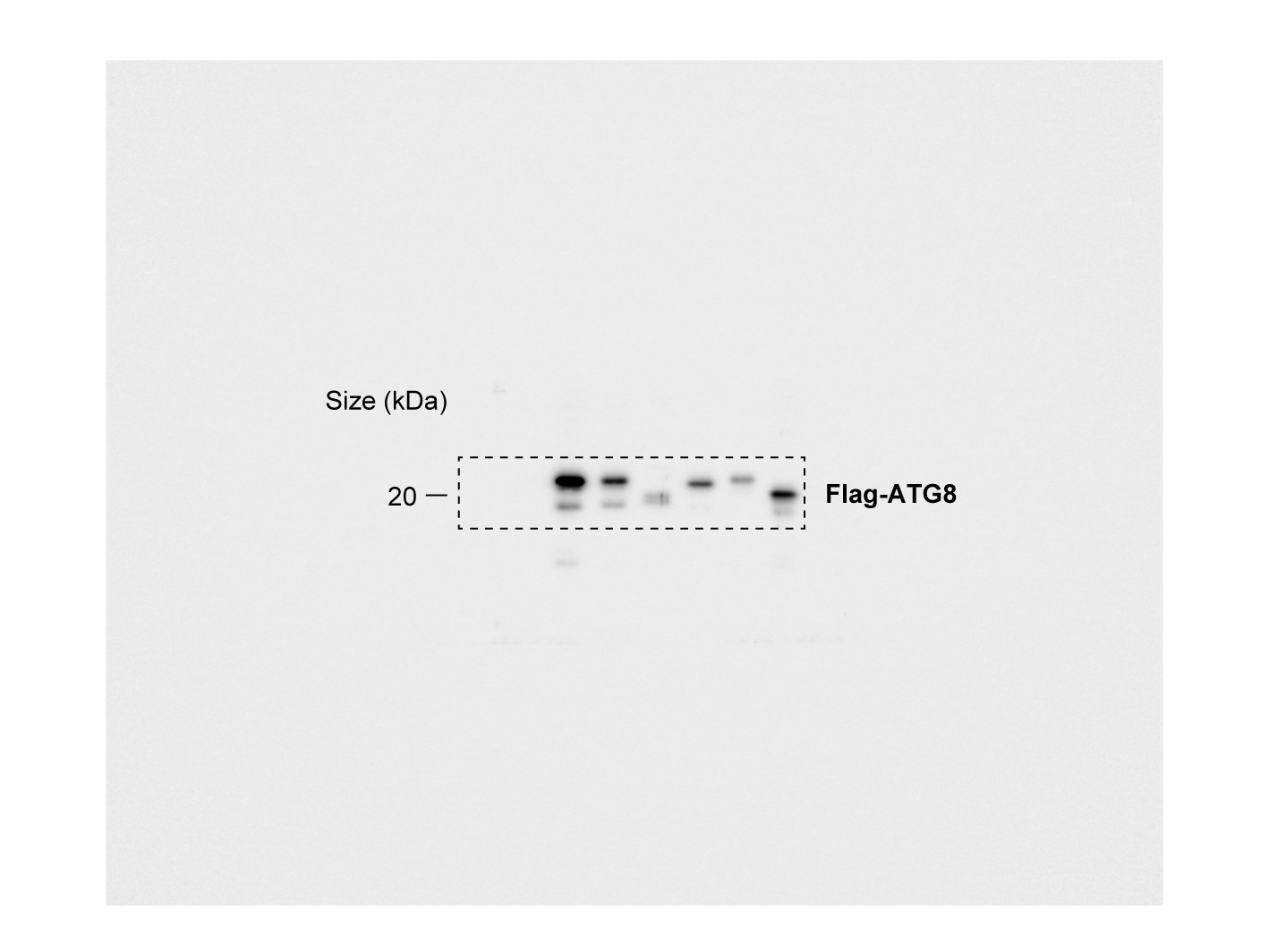

Supplement: Supplementary file 10 — Source Data for Figure 6 [file EMBR-24-e57300-s012.zip › Fig 6/6H/western Flag.tif]

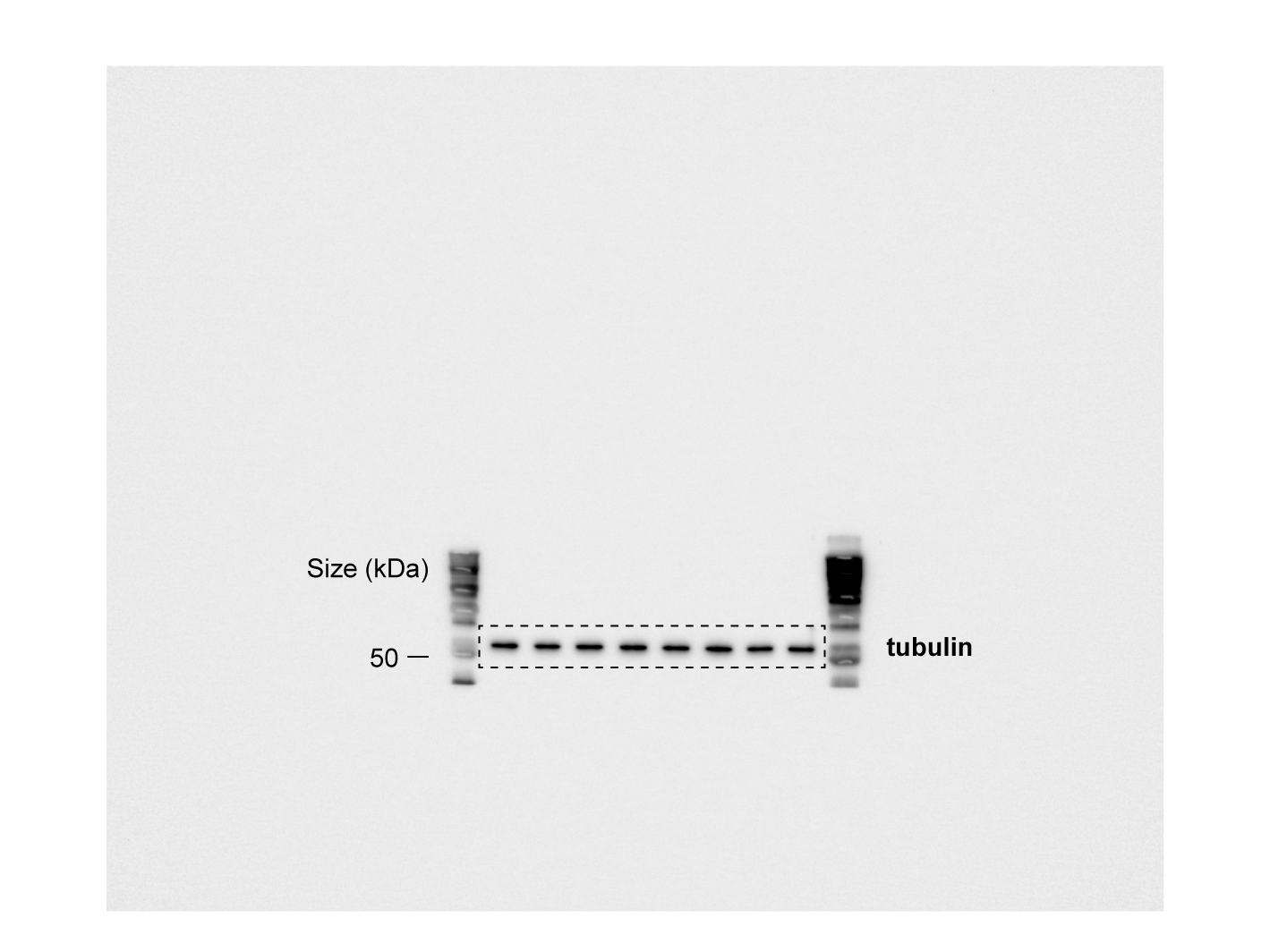

Supplement: Supplementary file 10 — Source Data for Figure 6 [file EMBR-24-e57300-s012.zip › Fig 6/6H/western tubulin.tif]

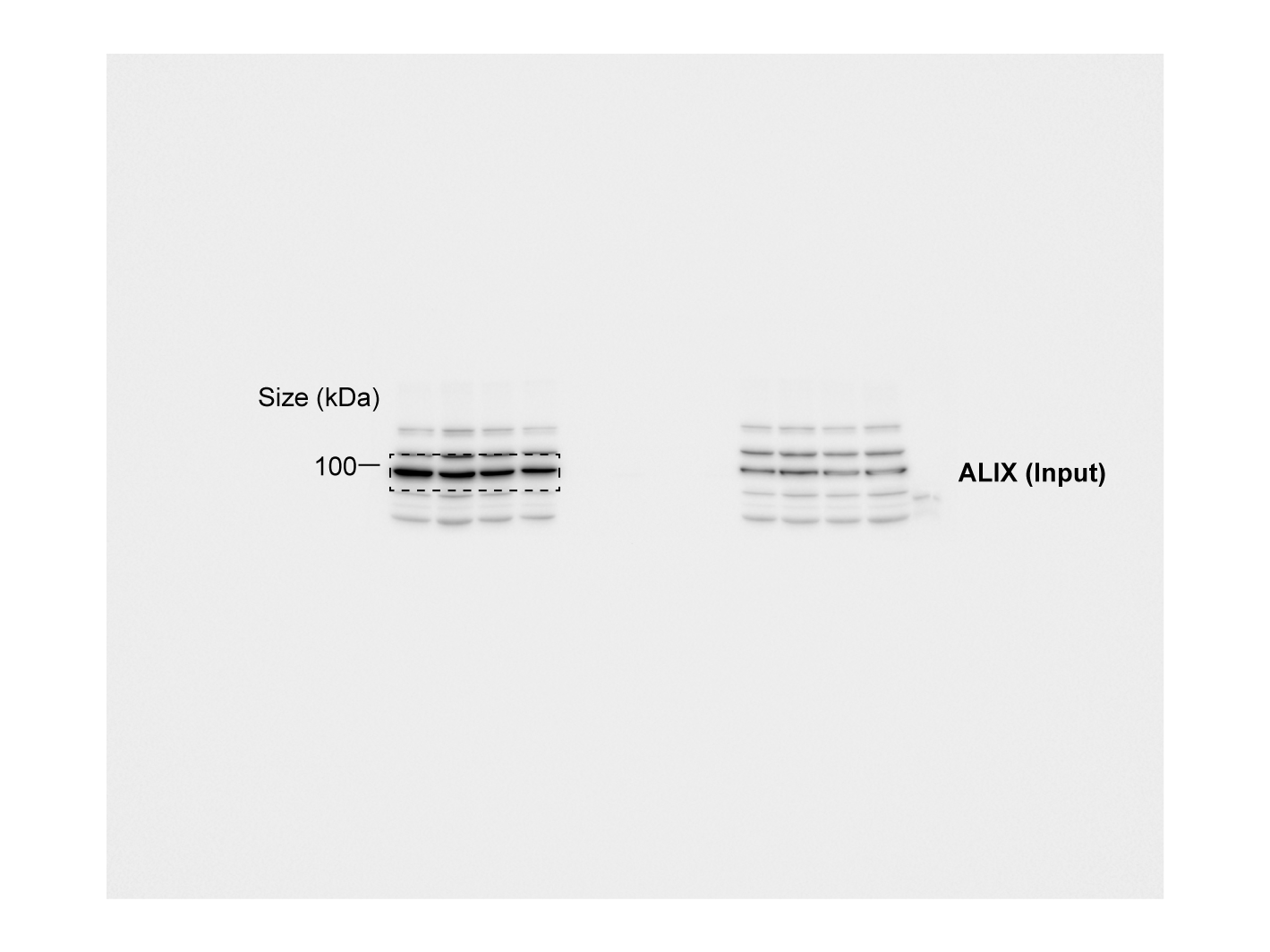

Supplement: Supplementary file 10 — Source Data for Figure 6 [file EMBR-24-e57300-s012.zip › Fig 6/6N/western ALIX (Input).tif]

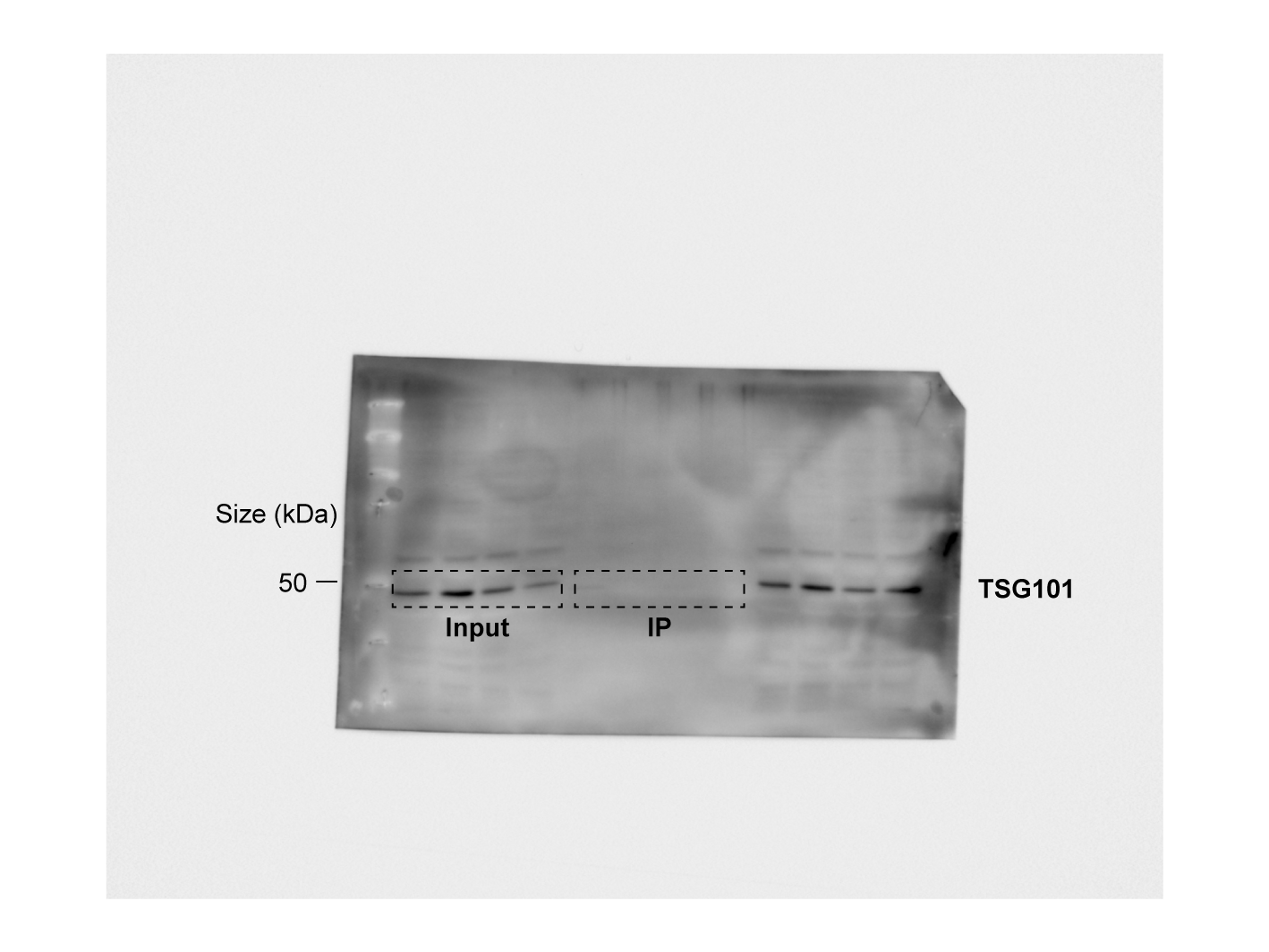

Supplement: Supplementary file 10 — Source Data for Figure 6 [file EMBR-24-e57300-s012.zip › Fig 6/6N/western TSG101.tif]

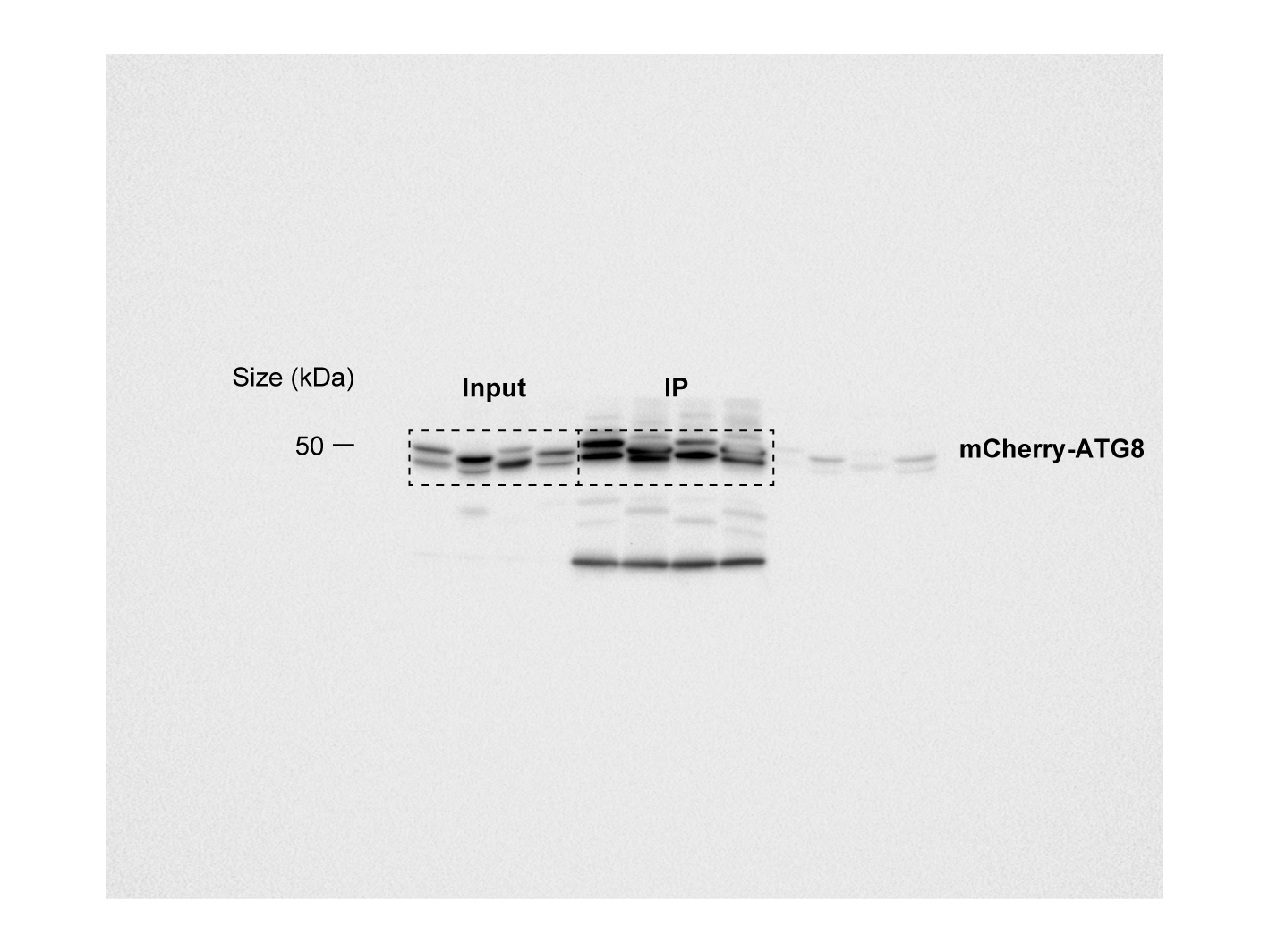

Supplement: Supplementary file 10 — Source Data for Figure 6 [file EMBR-24-e57300-s012.zip › Fig 6/6N/western RFP.tif]

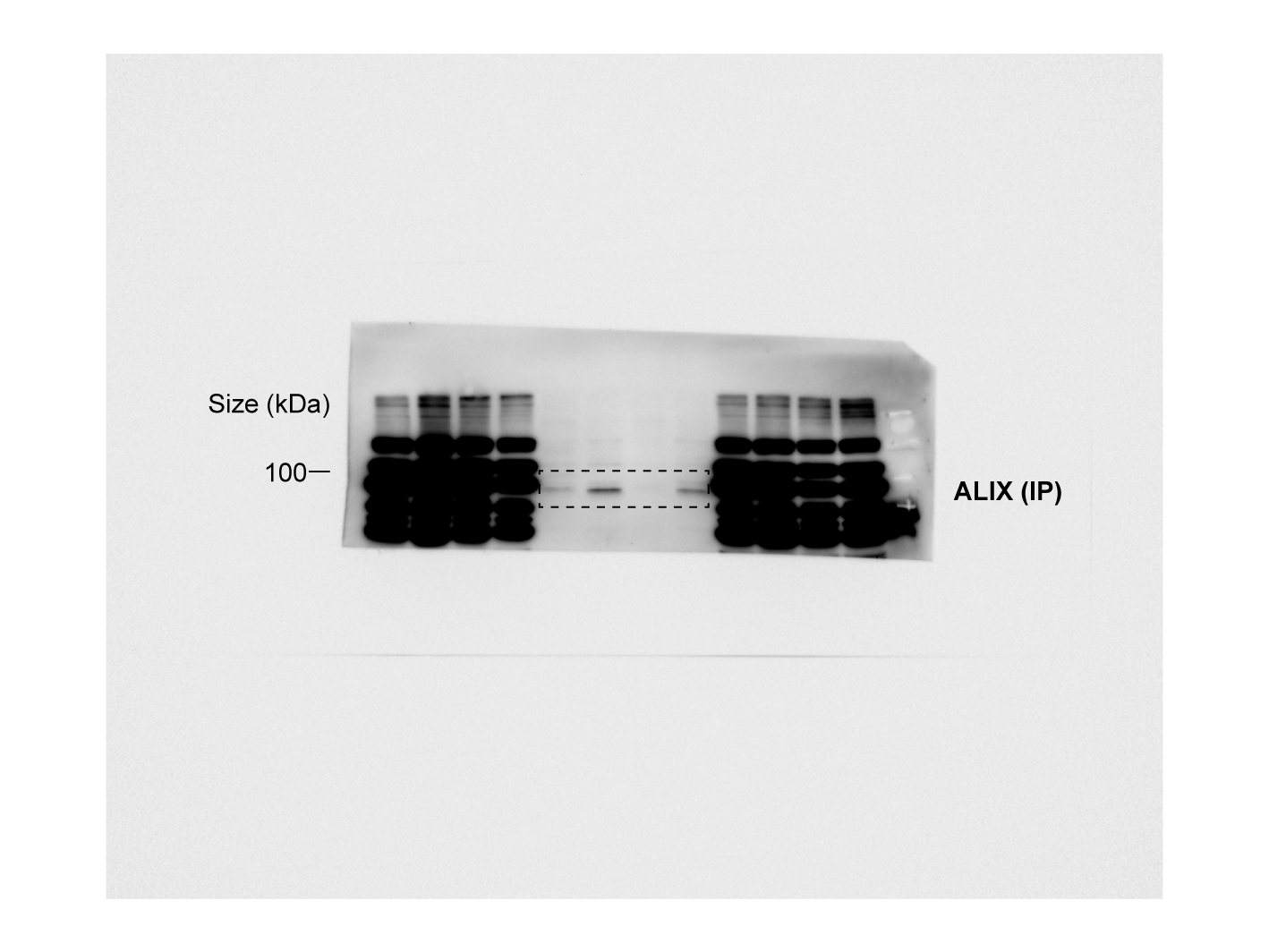

Supplement: Supplementary file 10 — Source Data for Figure 6 [file EMBR-24-e57300-s012.zip › Fig 6/6N/western ALIX (IP).tif]

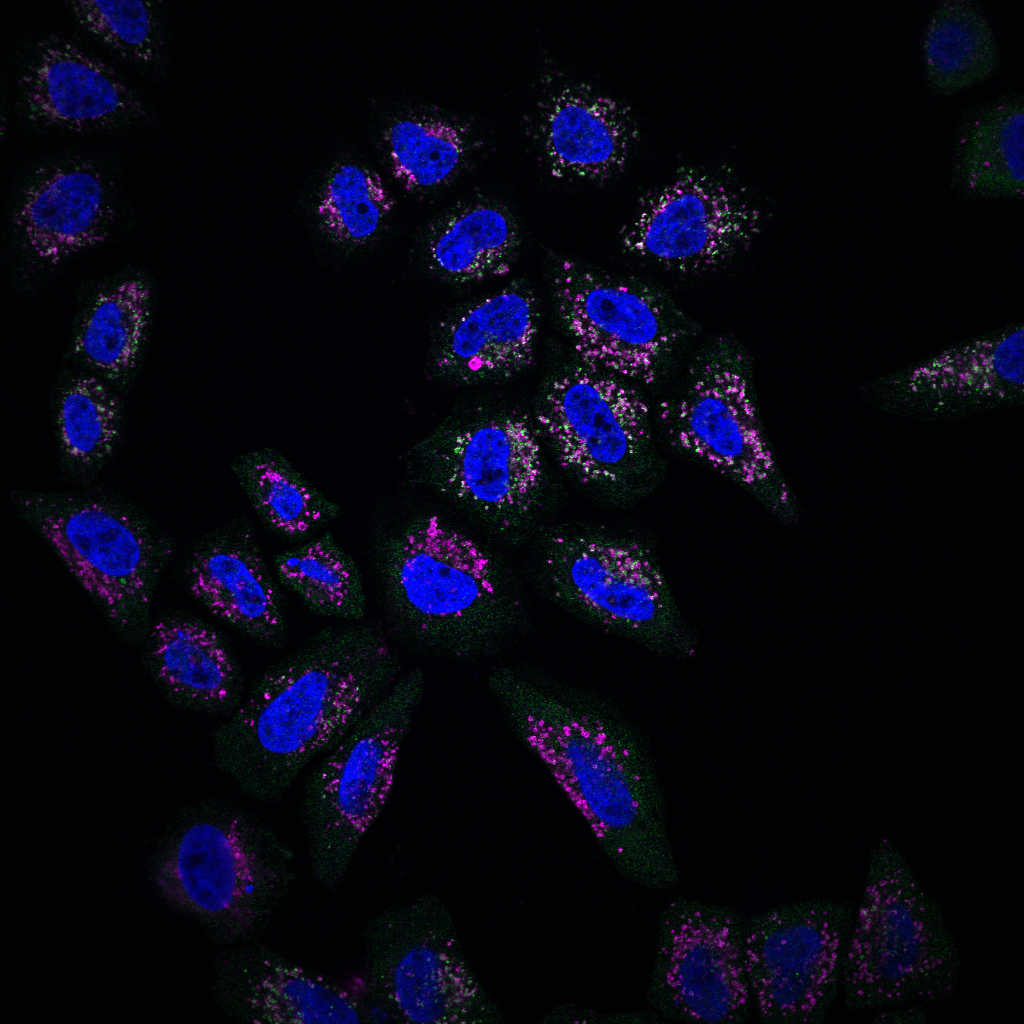

Supplement: Supplementary file 10 — Source Data for Figure 6 [file EMBR-24-e57300-s012.zip › Fig 6/6G/hexa KO_Flag-GBRPL2_LLOMe.tif]

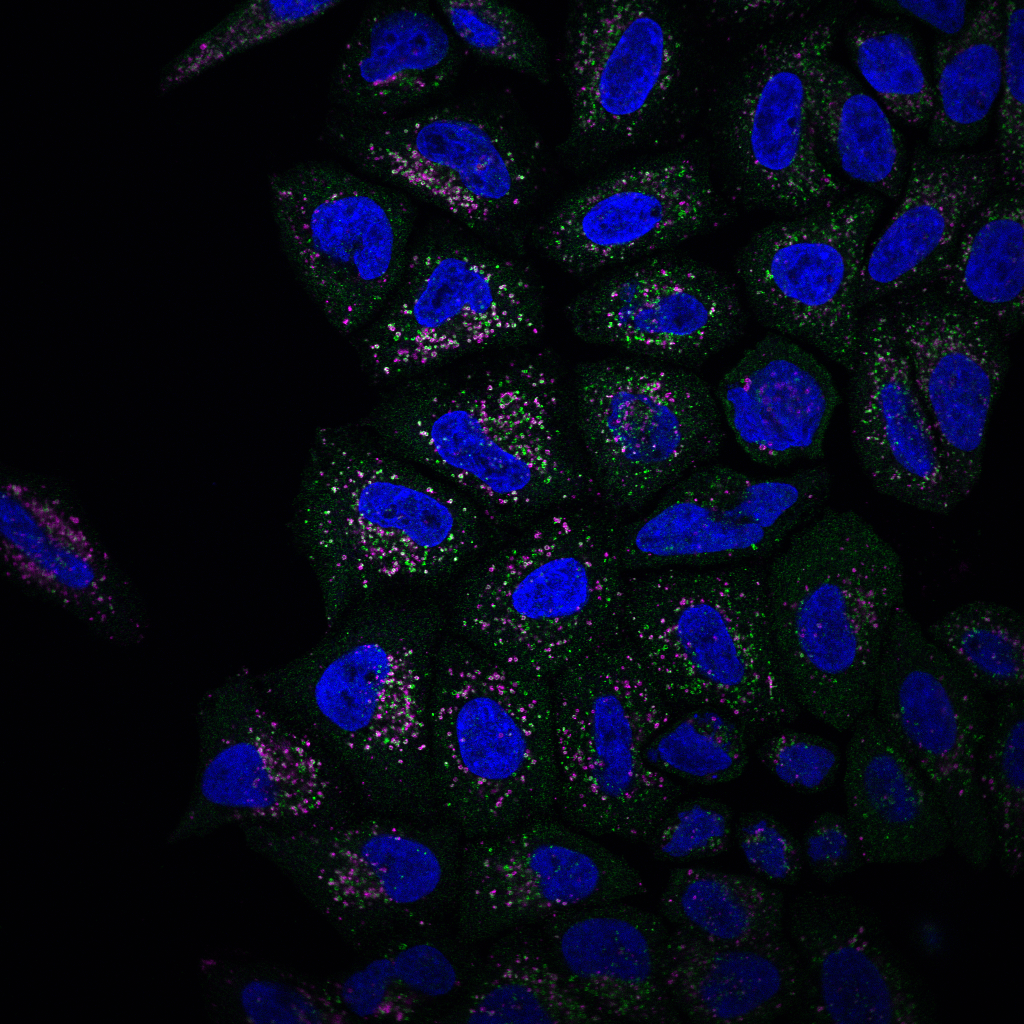

Supplement: Supplementary file 10 — Source Data for Figure 6 [file EMBR-24-e57300-s012.zip › Fig 6/6G/WT_Flag_LLOMe.tif]

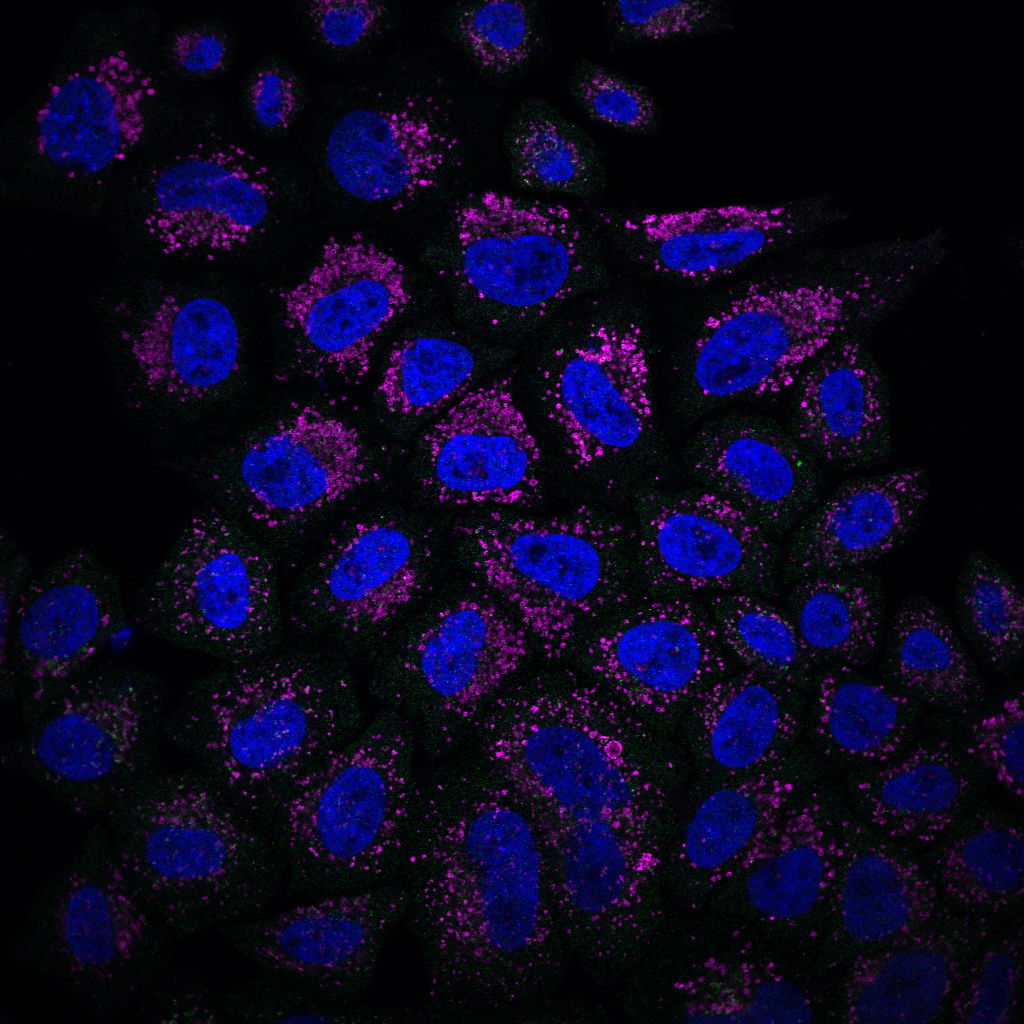

Supplement: Supplementary file 10 — Source Data for Figure 6 [file EMBR-24-e57300-s012.zip › Fig 6/6G/hexa KO_Flag-LC3C_LLOMe.tif]

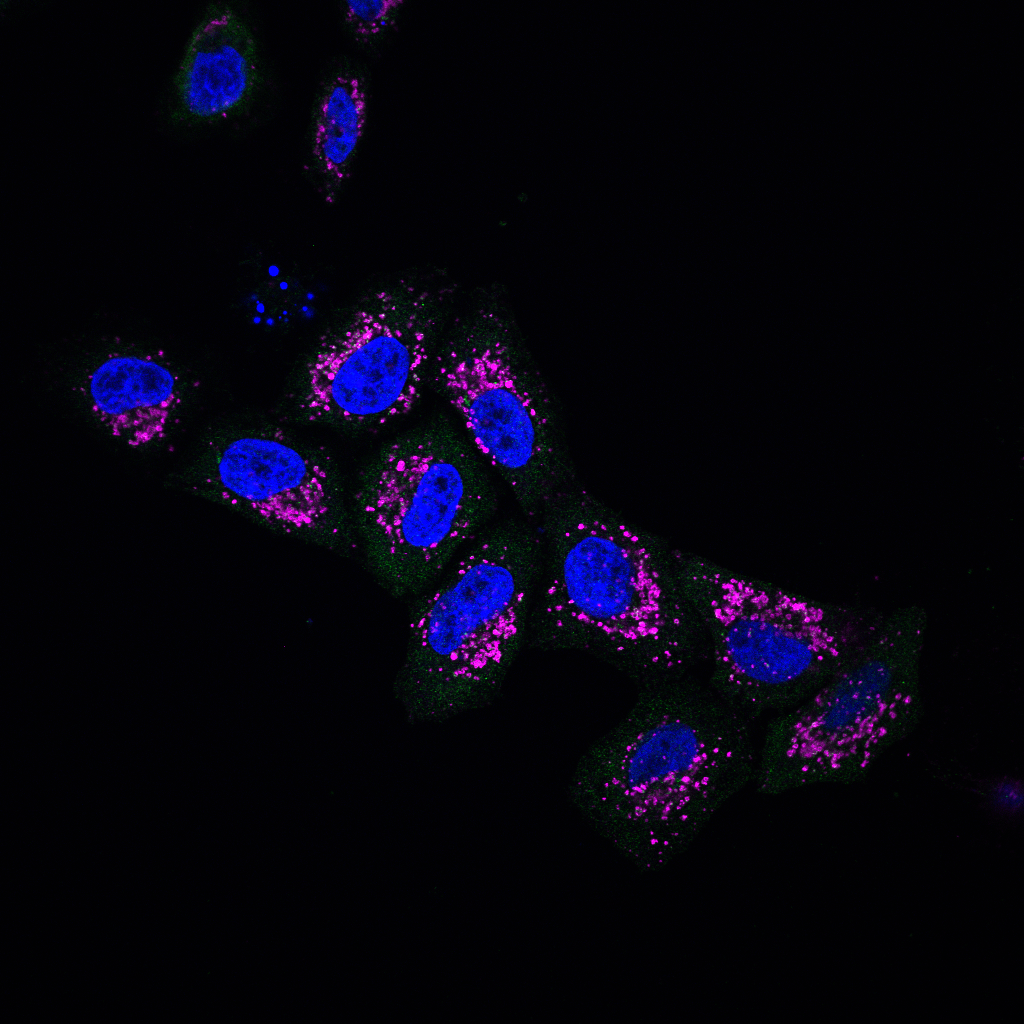

Supplement: Supplementary file 10 — Source Data for Figure 6 [file EMBR-24-e57300-s012.zip › Fig 6/6G/hexa KO_Flag_LLOMe.tif]

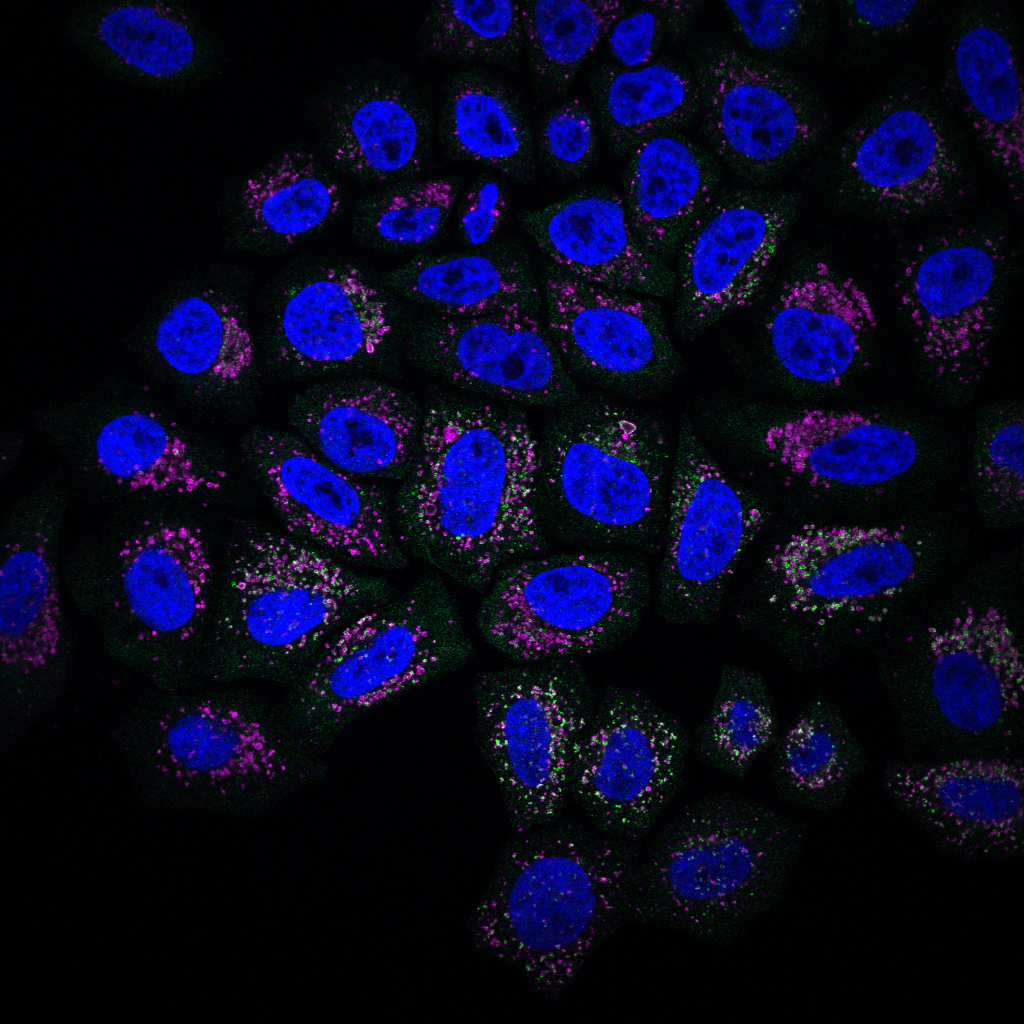

Supplement: Supplementary file 10 — Source Data for Figure 6 [file EMBR-24-e57300-s012.zip › Fig 6/6G/hexa KO_Flag-GBRP_LLOMe.tif]

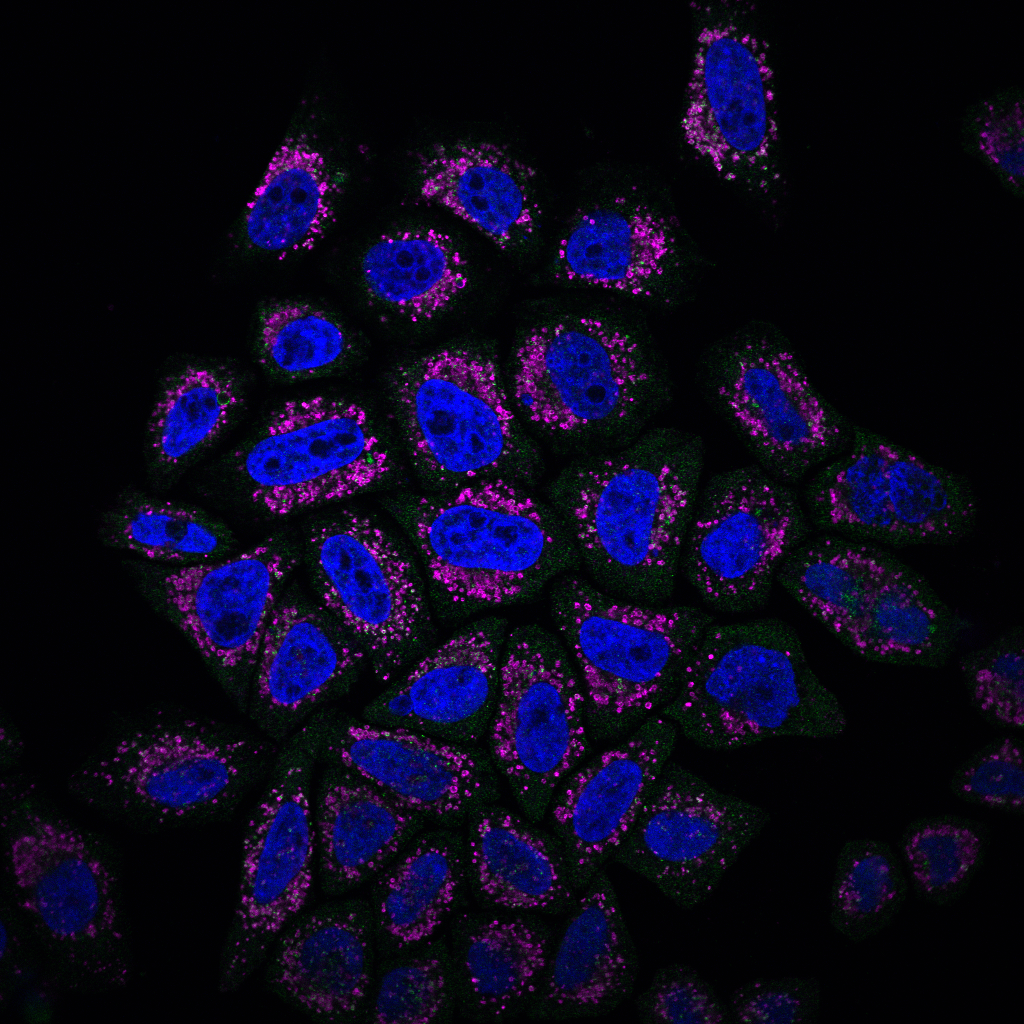

Supplement: Supplementary file 10 — Source Data for Figure 6 [file EMBR-24-e57300-s012.zip › Fig 6/6G/hexa KO_Flag-LC3B_LLOMe.tif]

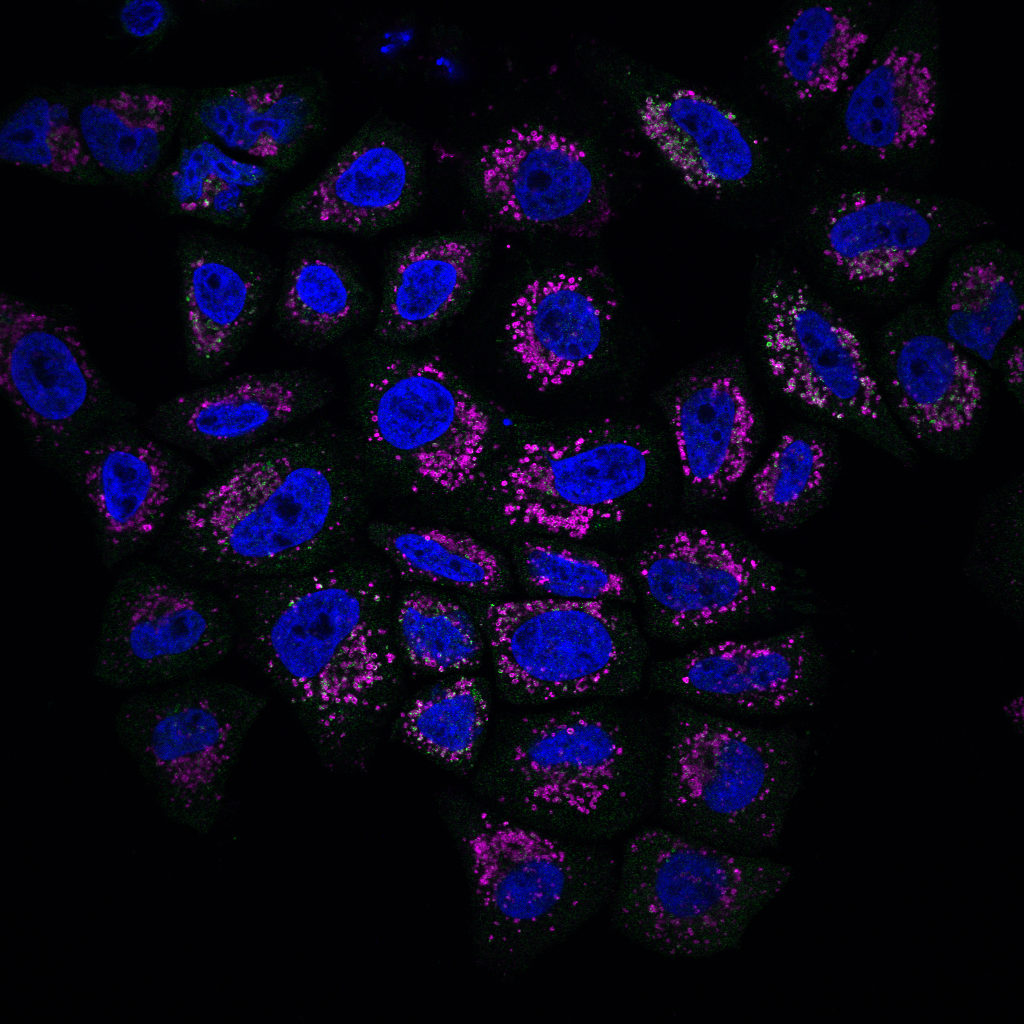

Supplement: Supplementary file 10 — Source Data for Figure 6 [file EMBR-24-e57300-s012.zip › Fig 6/6G/hexa KO_Flag-LC3A_LLOMe.tif]

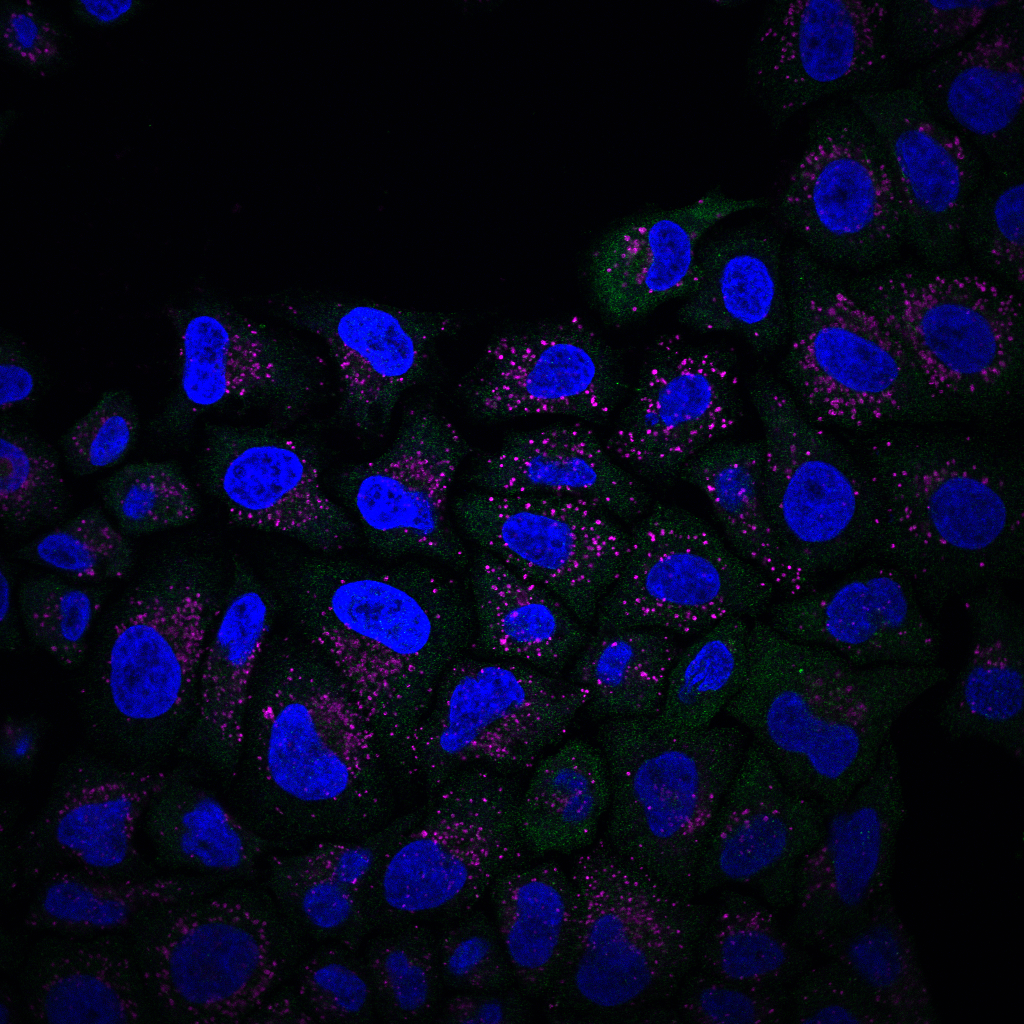

Supplement: Supplementary file 10 — Source Data for Figure 6 [file EMBR-24-e57300-s012.zip › Fig 6/6G/WT_Flag_non-treated.tif]

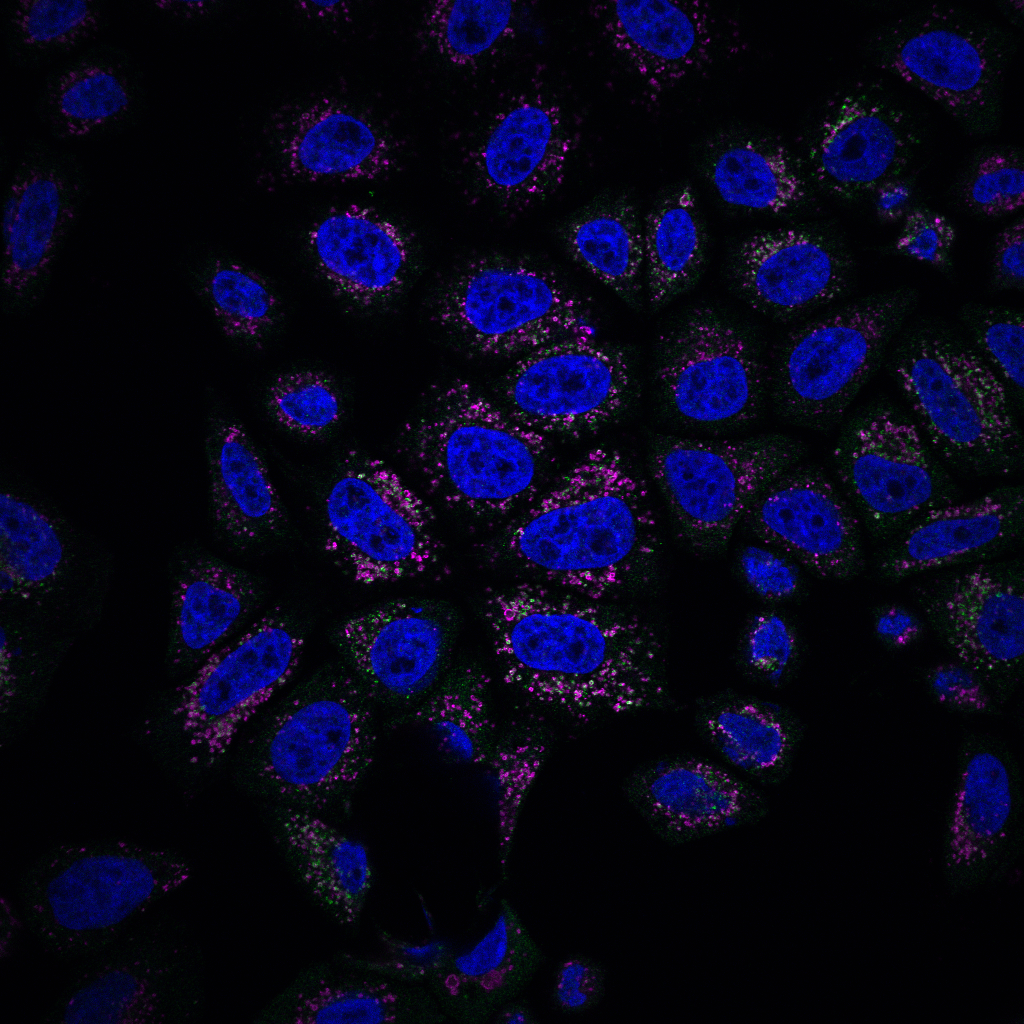

Supplement: Supplementary file 10 — Source Data for Figure 6 [file EMBR-24-e57300-s012.zip › Fig 6/6G/hexa KO_Flag-GBRPL1_LLOMe.tif]

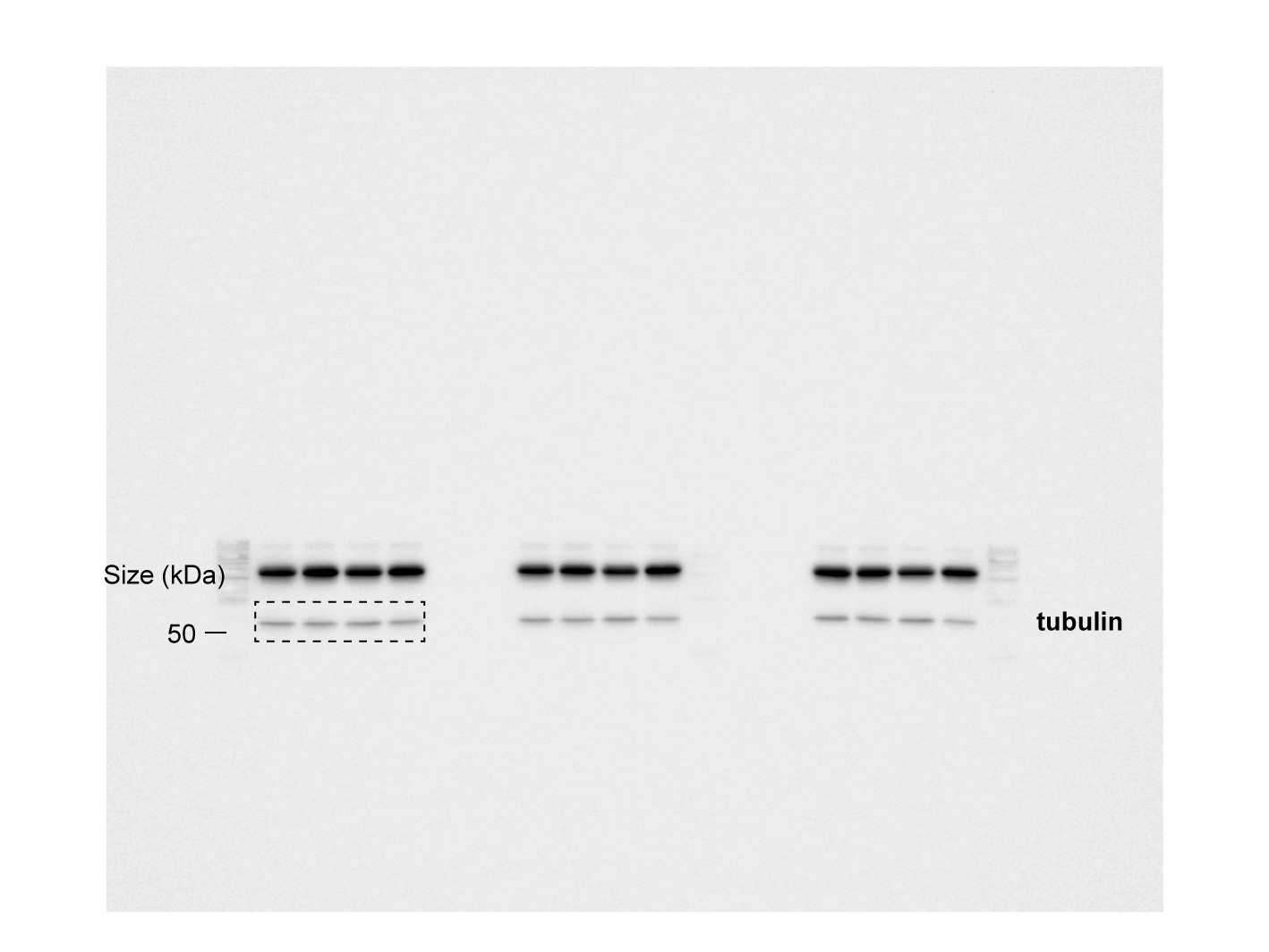

Supplement: Supplementary file 10 — Source Data for Figure 6 [file EMBR-24-e57300-s012.zip › Fig 6/6L/western tubulin.tif]

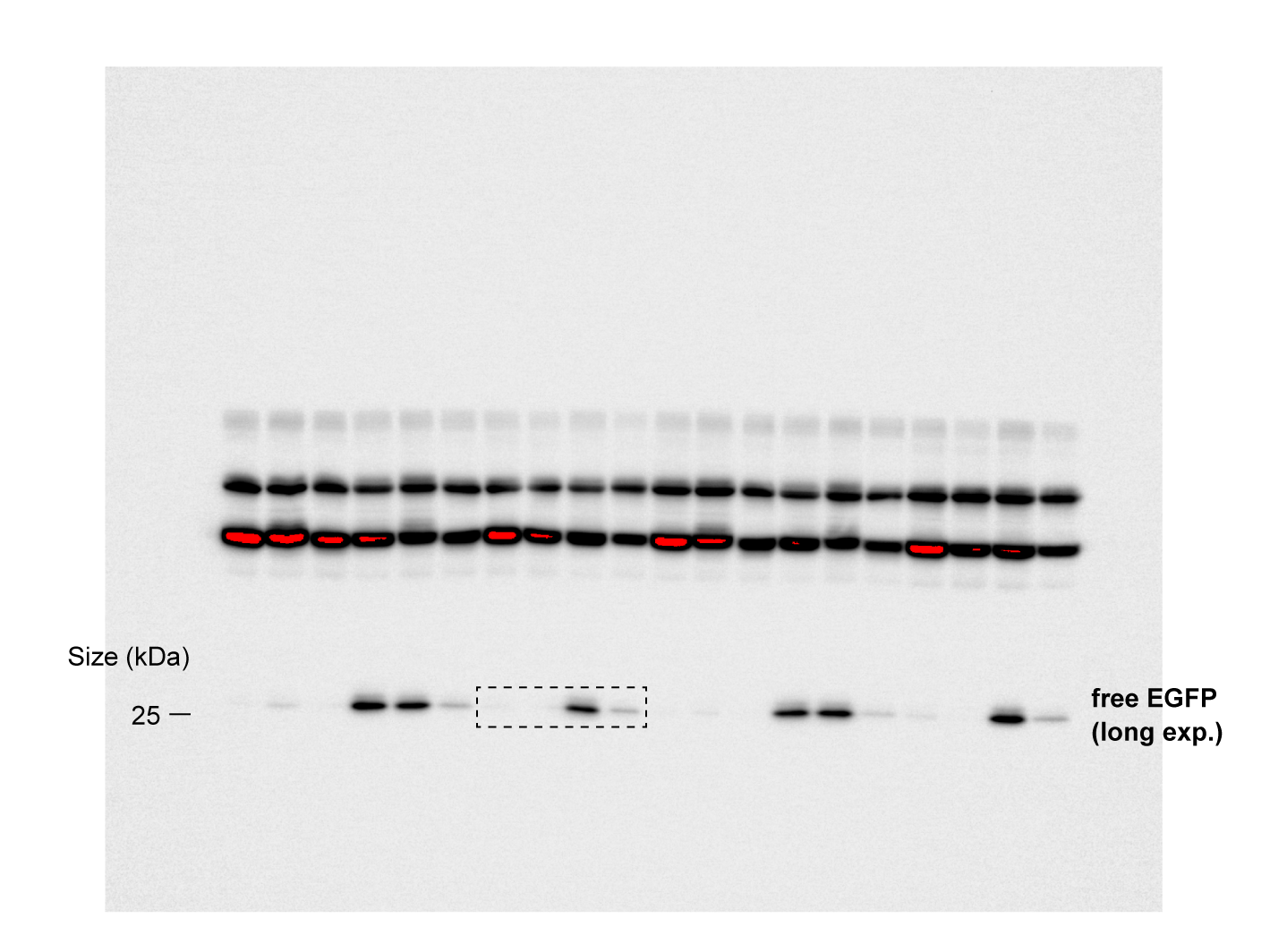

Supplement: Supplementary file 10 — Source Data for Figure 6 [file EMBR-24-e57300-s012.zip › Fig 6/6L/western EGFP (long exp.).tif]

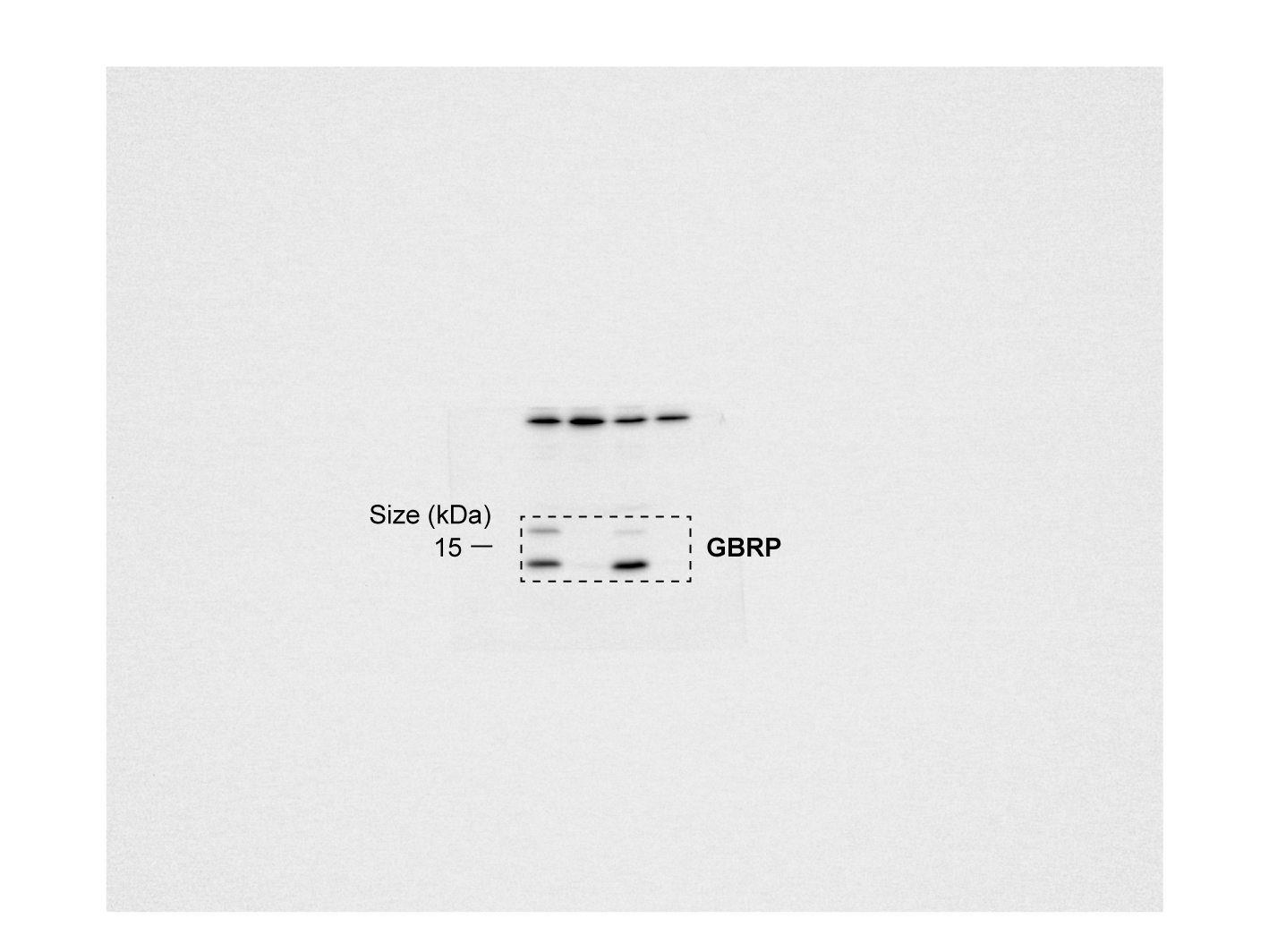

Supplement: Supplementary file 10 — Source Data for Figure 6 [file EMBR-24-e57300-s012.zip › Fig 6/6L/western GBRP.tif]

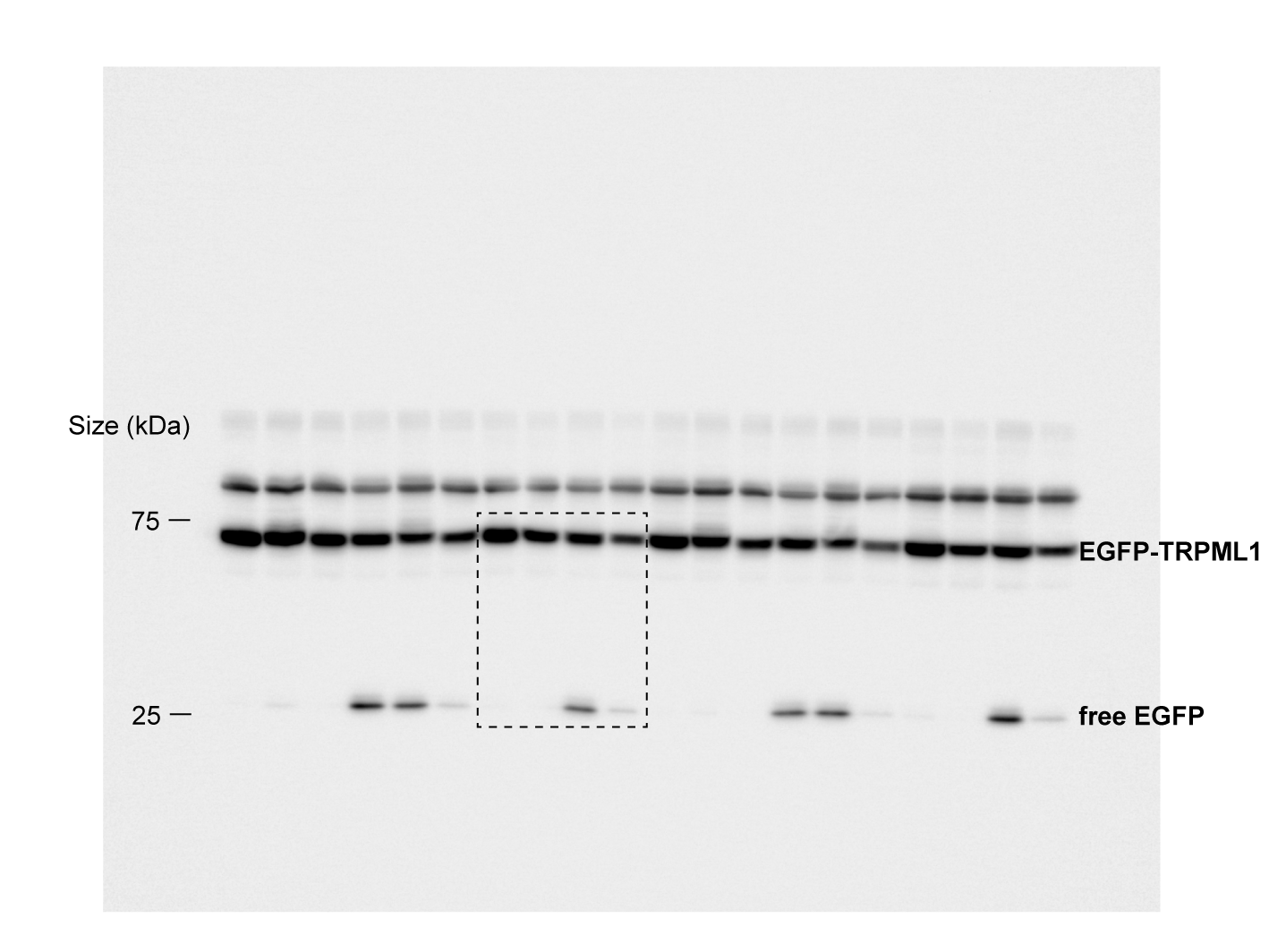

Supplement: Supplementary file 10 — Source Data for Figure 6 [file EMBR-24-e57300-s012.zip › Fig 6/6L/western EGFP.tif]

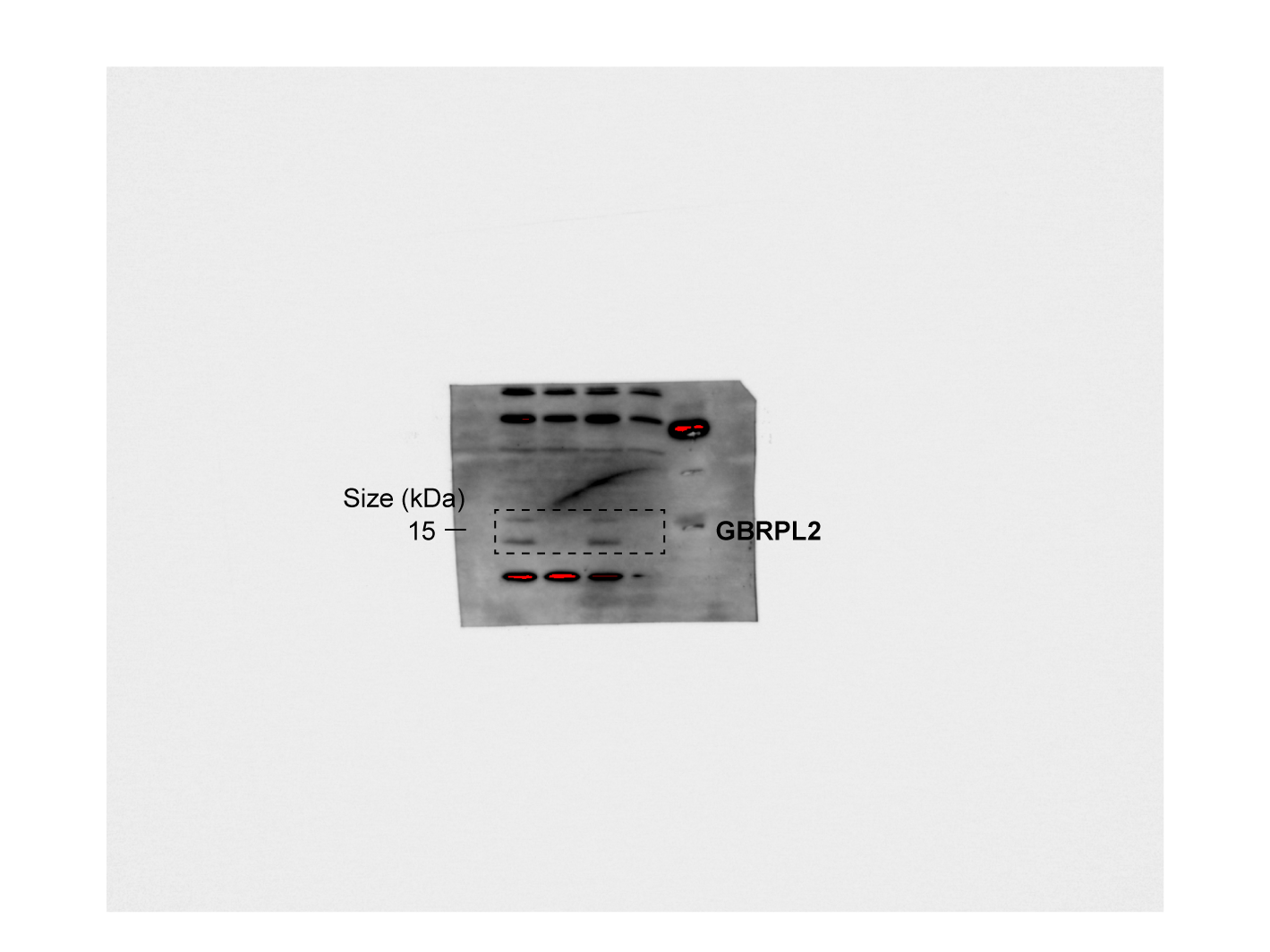

Supplement: Supplementary file 10 — Source Data for Figure 6 [file EMBR-24-e57300-s012.zip › Fig 6/6L/western GBRPL2.tif]

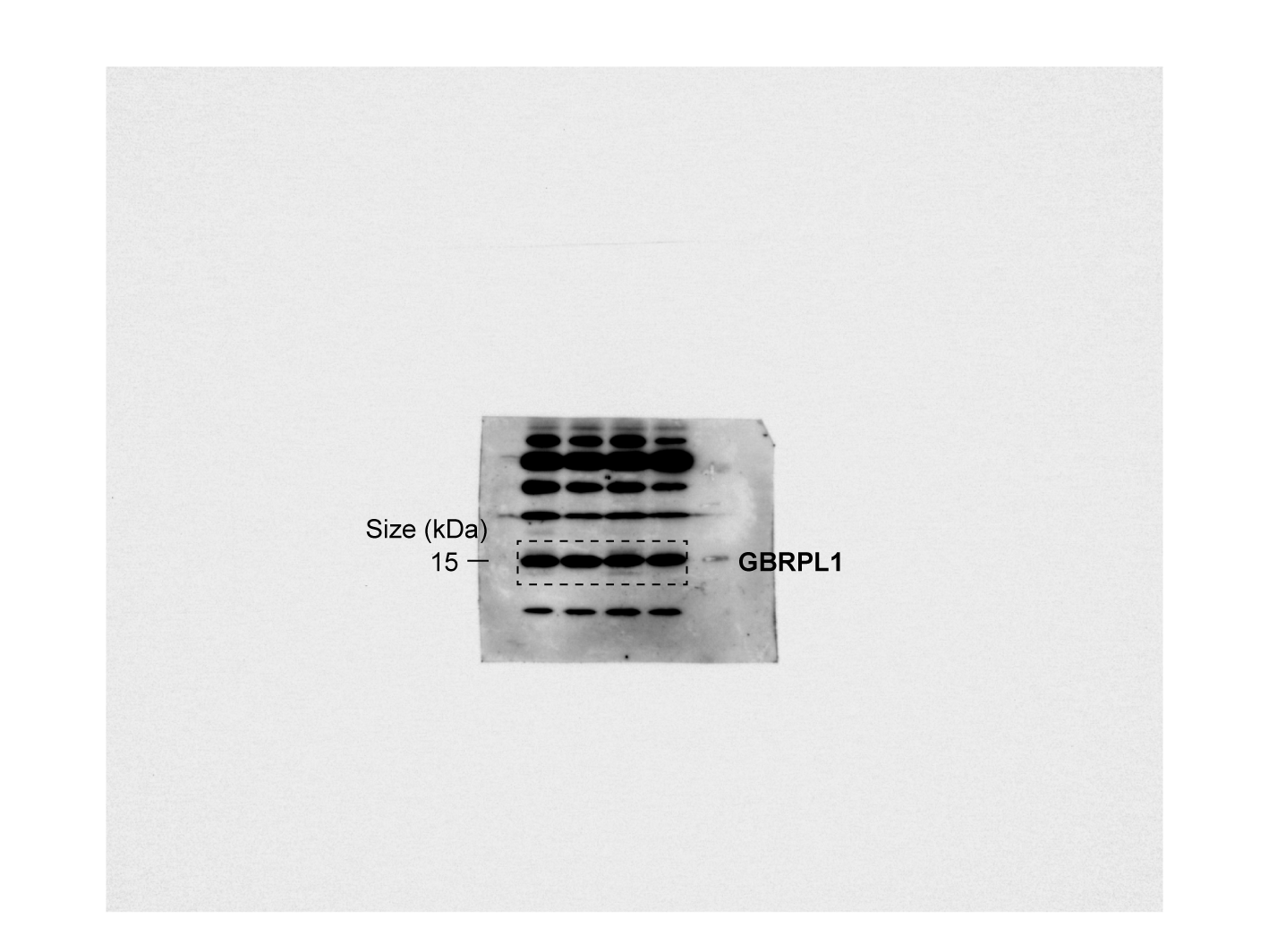

Supplement: Supplementary file 10 — Source Data for Figure 6 [file EMBR-24-e57300-s012.zip › Fig 6/6L/western GBRPL1.tif]

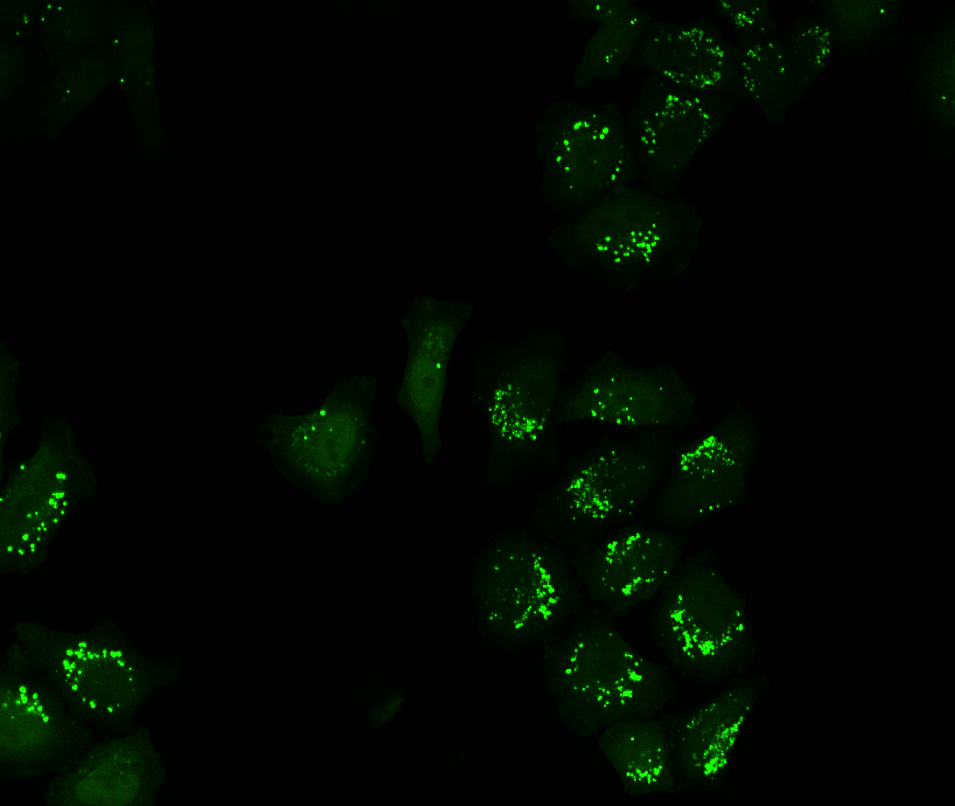

Supplement: Supplementary file 10 — Source Data for Figure 6 [file EMBR-24-e57300-s012.zip › Fig 6/6J/GBRP TKO_0h.tif]

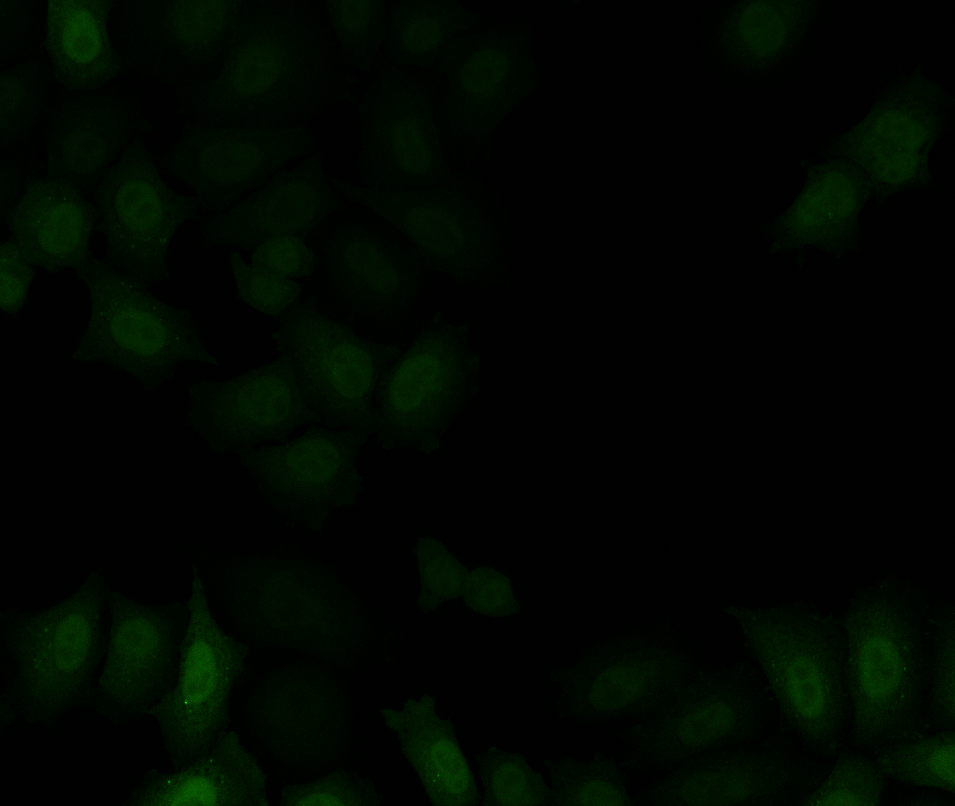

Supplement: Supplementary file 10 — Source Data for Figure 6 [file EMBR-24-e57300-s012.zip › Fig 6/6J/WT_-1h.tif]

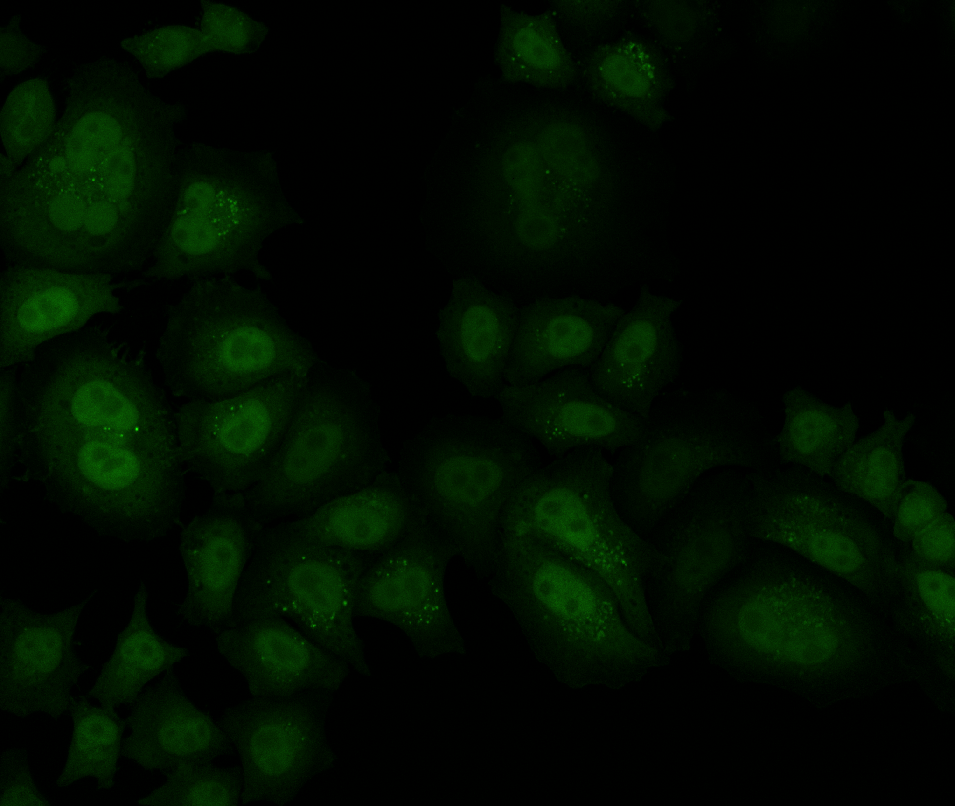

Supplement: Supplementary file 10 — Source Data for Figure 6 [file EMBR-24-e57300-s012.zip › Fig 6/6J/GBRP TKO_-1h.tif]

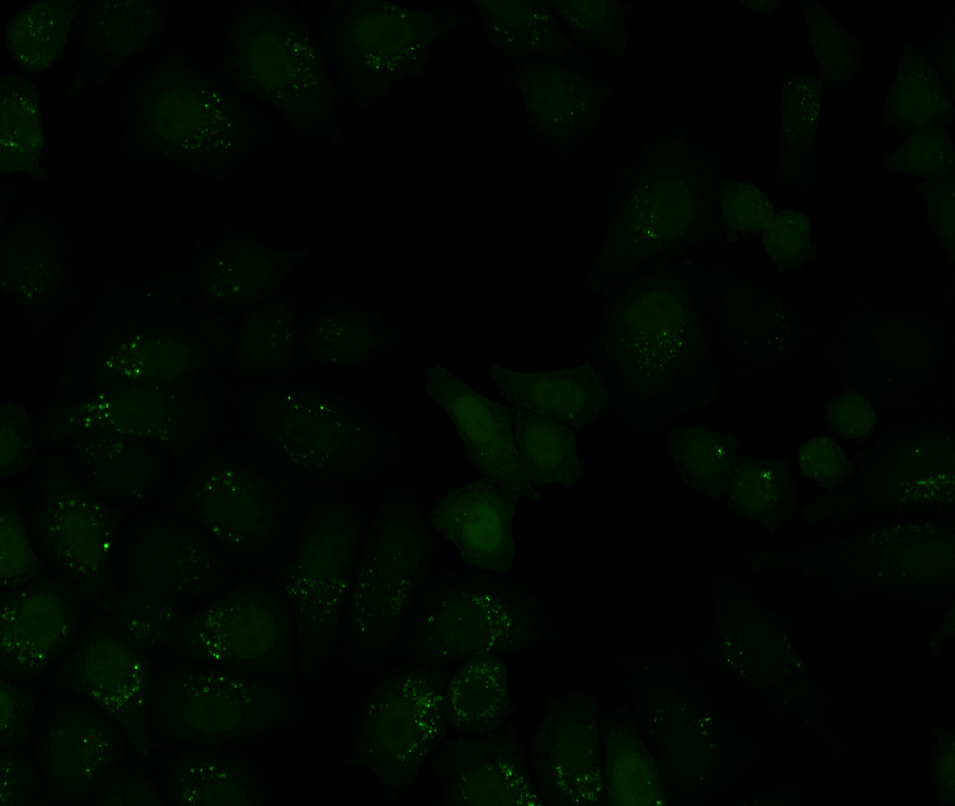

Supplement: Supplementary file 10 — Source Data for Figure 6 [file EMBR-24-e57300-s012.zip › Fig 6/6J/WT_10h.tif]

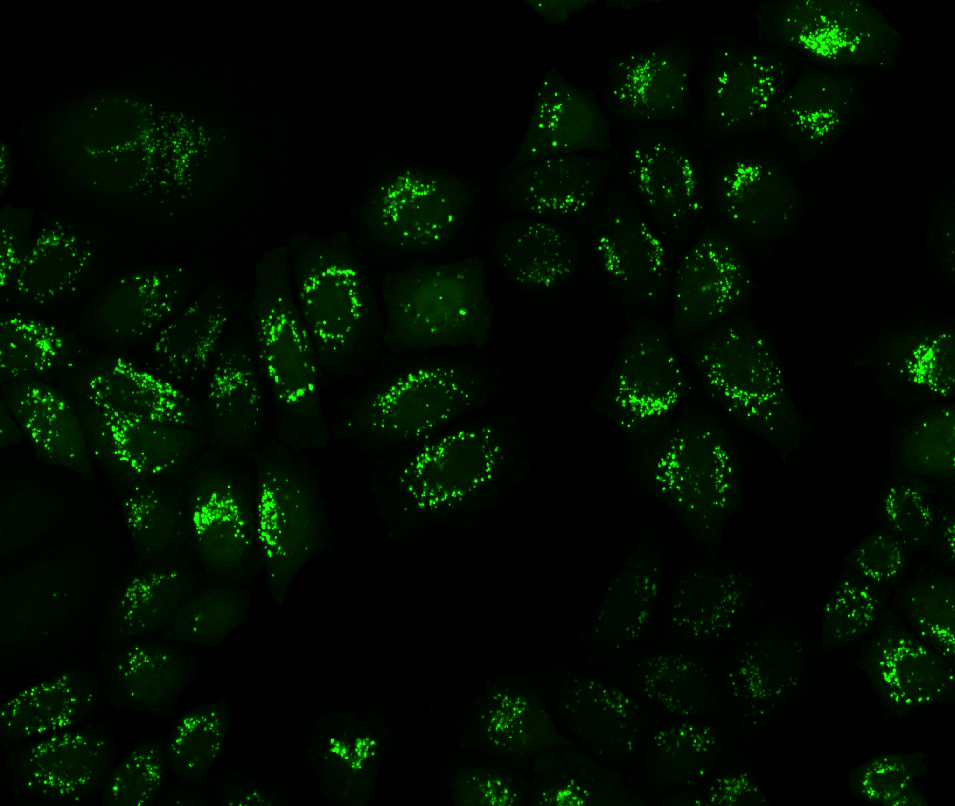

Supplement: Supplementary file 10 — Source Data for Figure 6 [file EMBR-24-e57300-s012.zip › Fig 6/6J/WT_0h.tif]

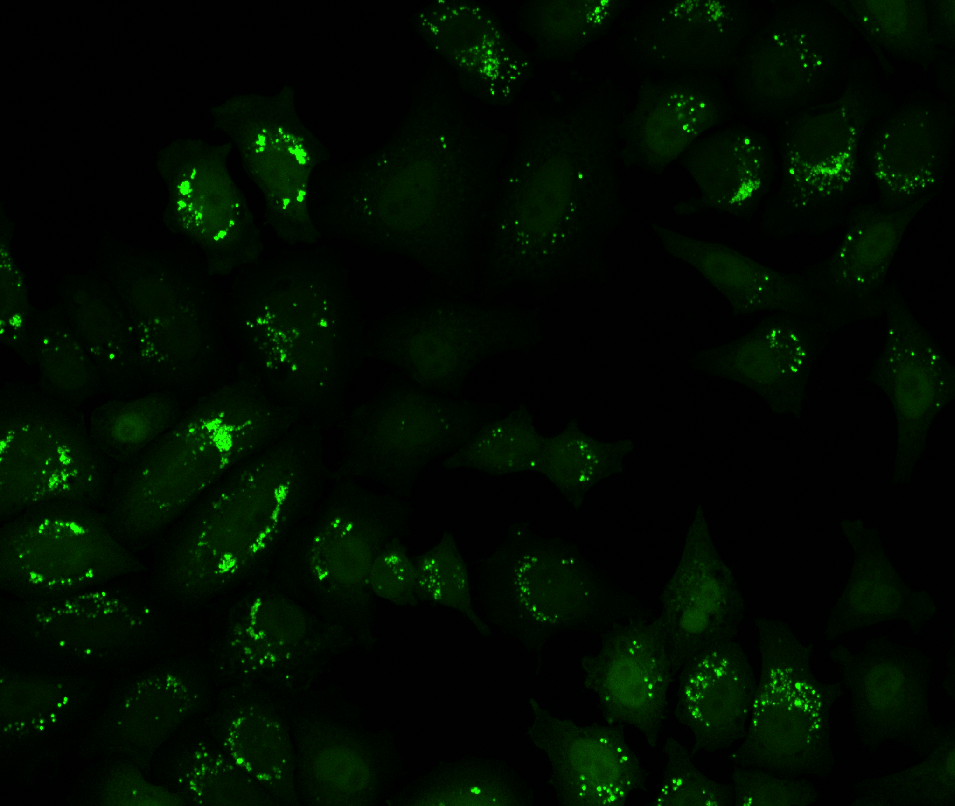

Supplement: Supplementary file 10 — Source Data for Figure 6 [file EMBR-24-e57300-s012.zip › Fig 6/6J/GBRP TKO_10h.tif]

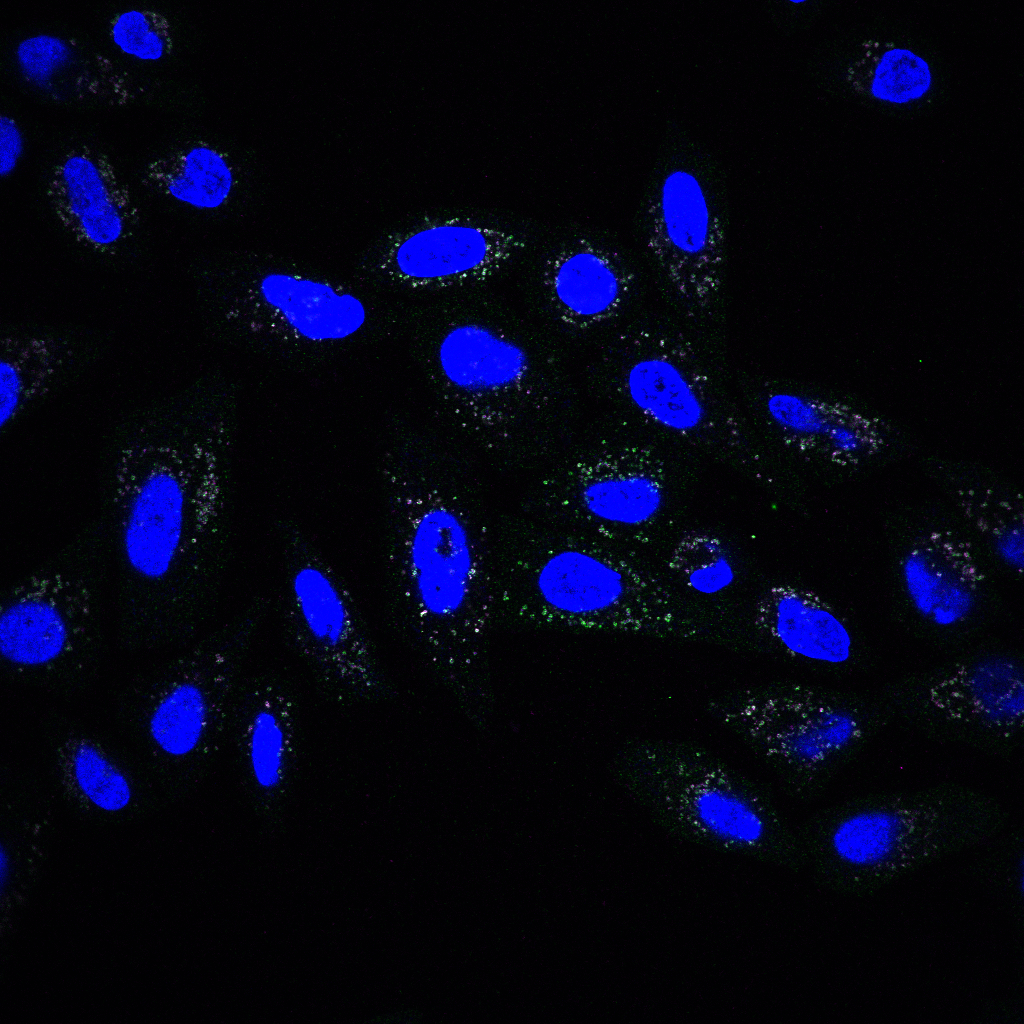

Supplement: Supplementary file 10 — Source Data for Figure 6 [file EMBR-24-e57300-s012.zip › Fig 6/6D/WT_LLOMe.tif]

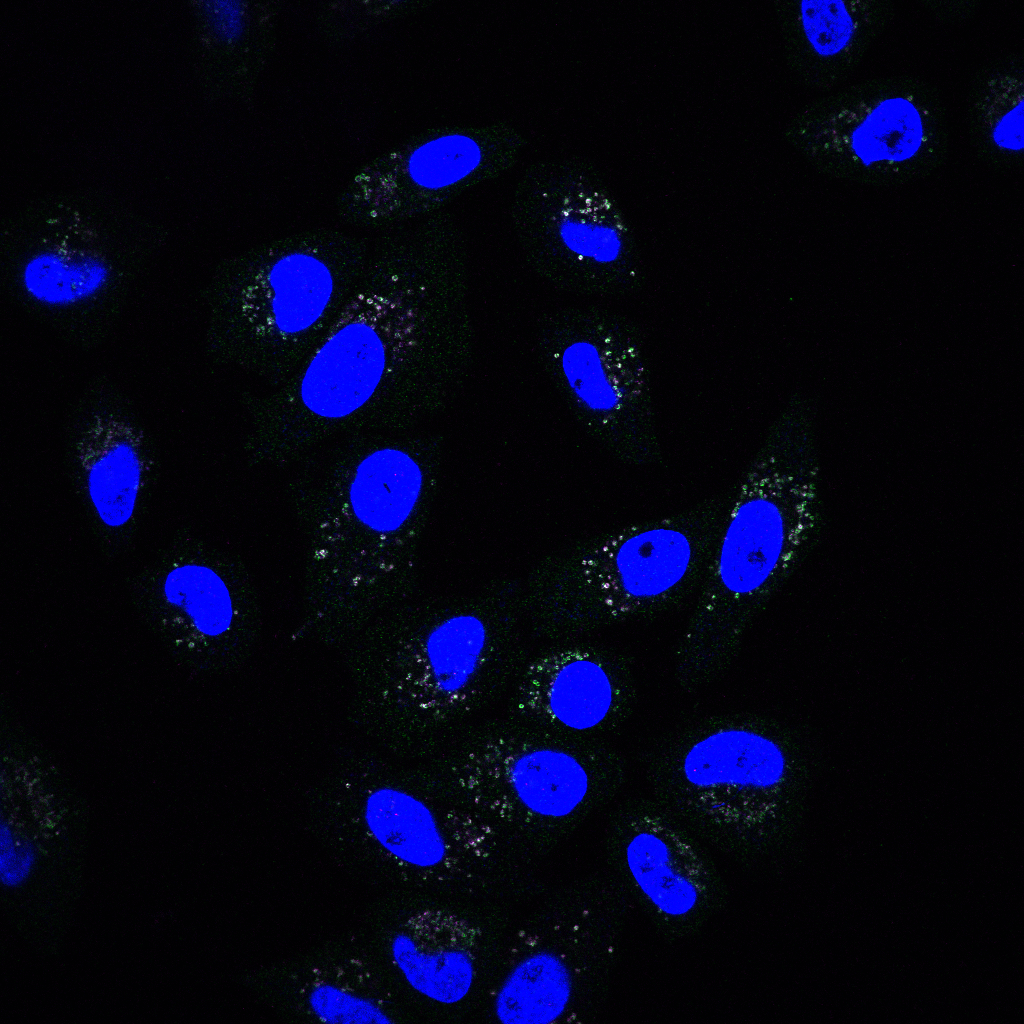

Supplement: Supplementary file 10 — Source Data for Figure 6 [file EMBR-24-e57300-s012.zip › Fig 6/6D/LC3 TKO_LLOMe.tif]

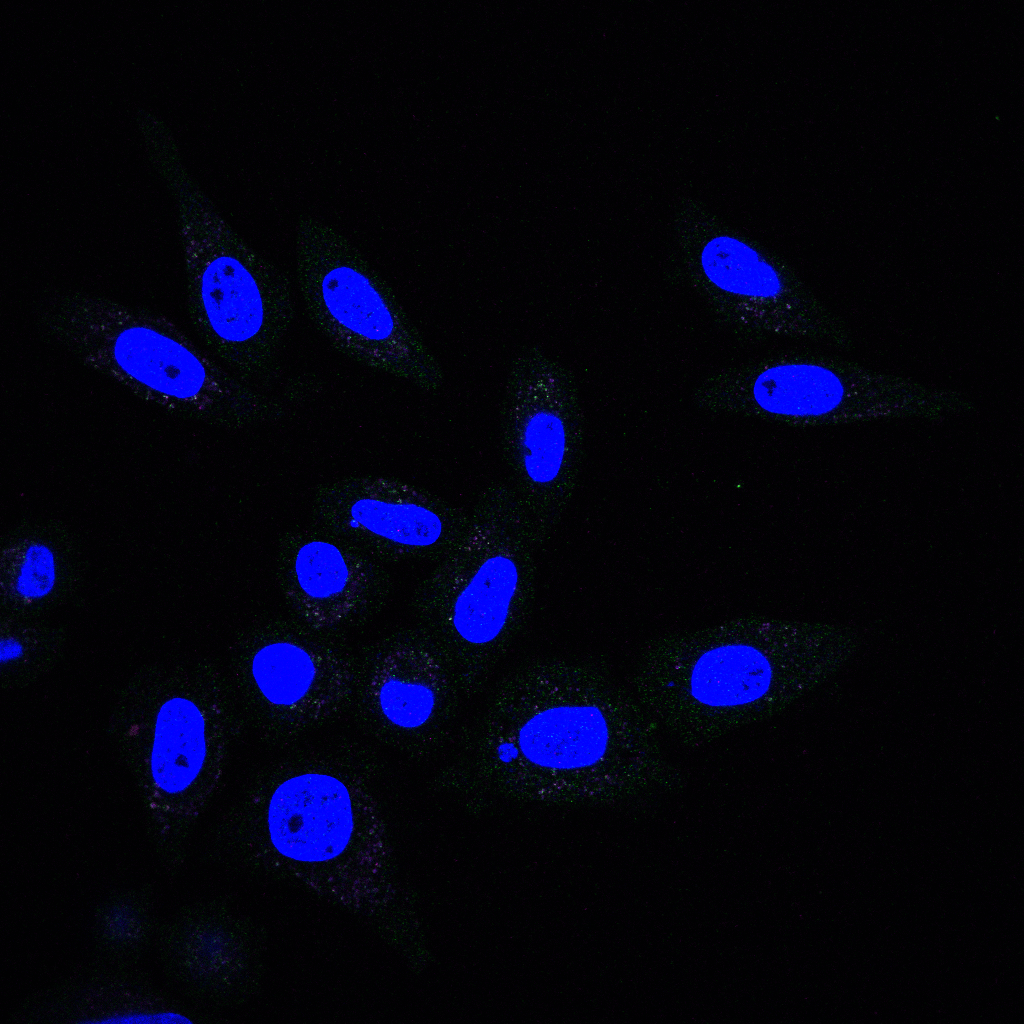

Supplement: Supplementary file 10 — Source Data for Figure 6 [file EMBR-24-e57300-s012.zip › Fig 6/6D/GABARAP TKO_LLOMe.tif]
