# Supplementary figures and images for: Microautophagy regulated by STK38 and GABARAPs is essential to repair lysosomes and prevent aging (part 4 of 4)
Source: EMBO Rep. 2023 Nov 21;24(12):e57300. doi: 10.15252/embr.202357300 (PMC10702834; doi:10.15252/embr.202357300)

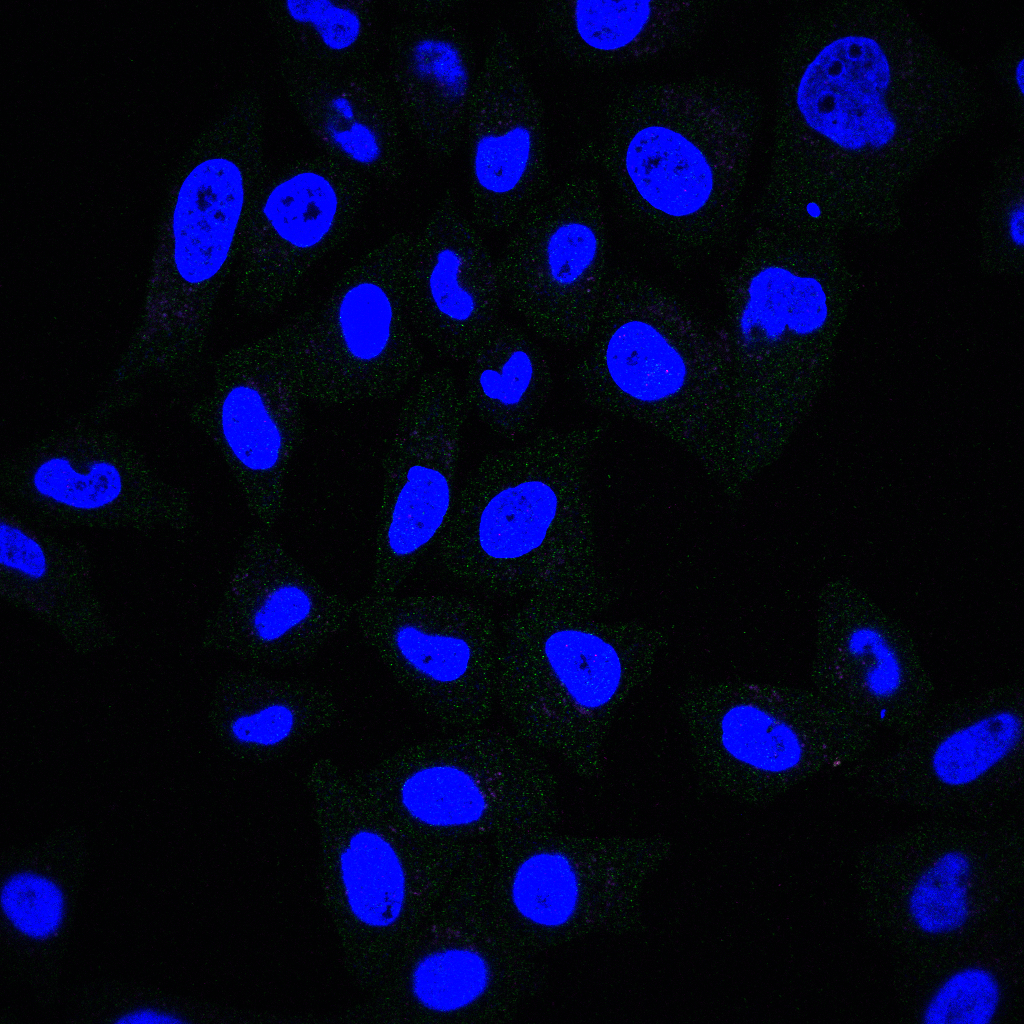

Supplement: Supplementary file 10 — Source Data for Figure 6 [file EMBR-24-e57300-s012.zip › Fig 6/6D/GABARAP TKO_non-treated.tif]

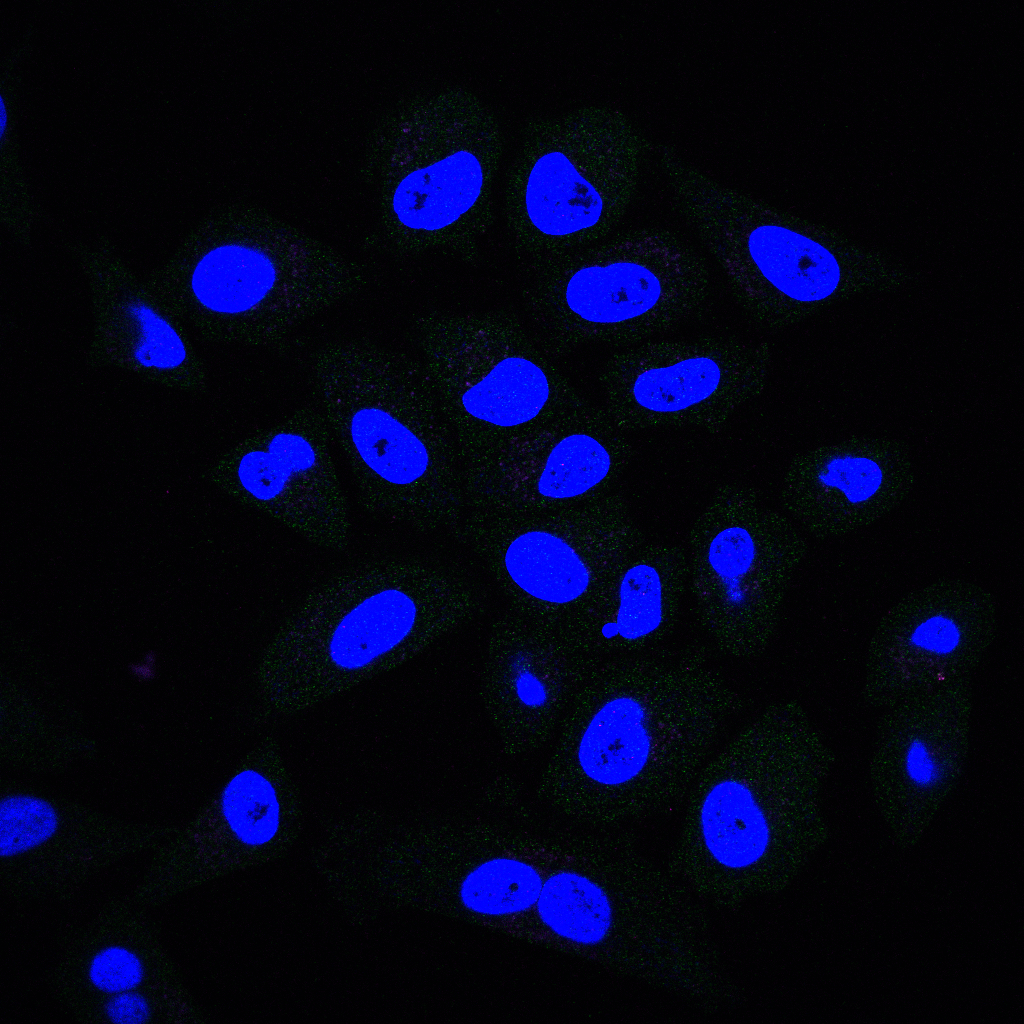

Supplement: Supplementary file 10 — Source Data for Figure 6 [file EMBR-24-e57300-s012.zip › Fig 6/6D/LC3 TKO_non-treated.tif]

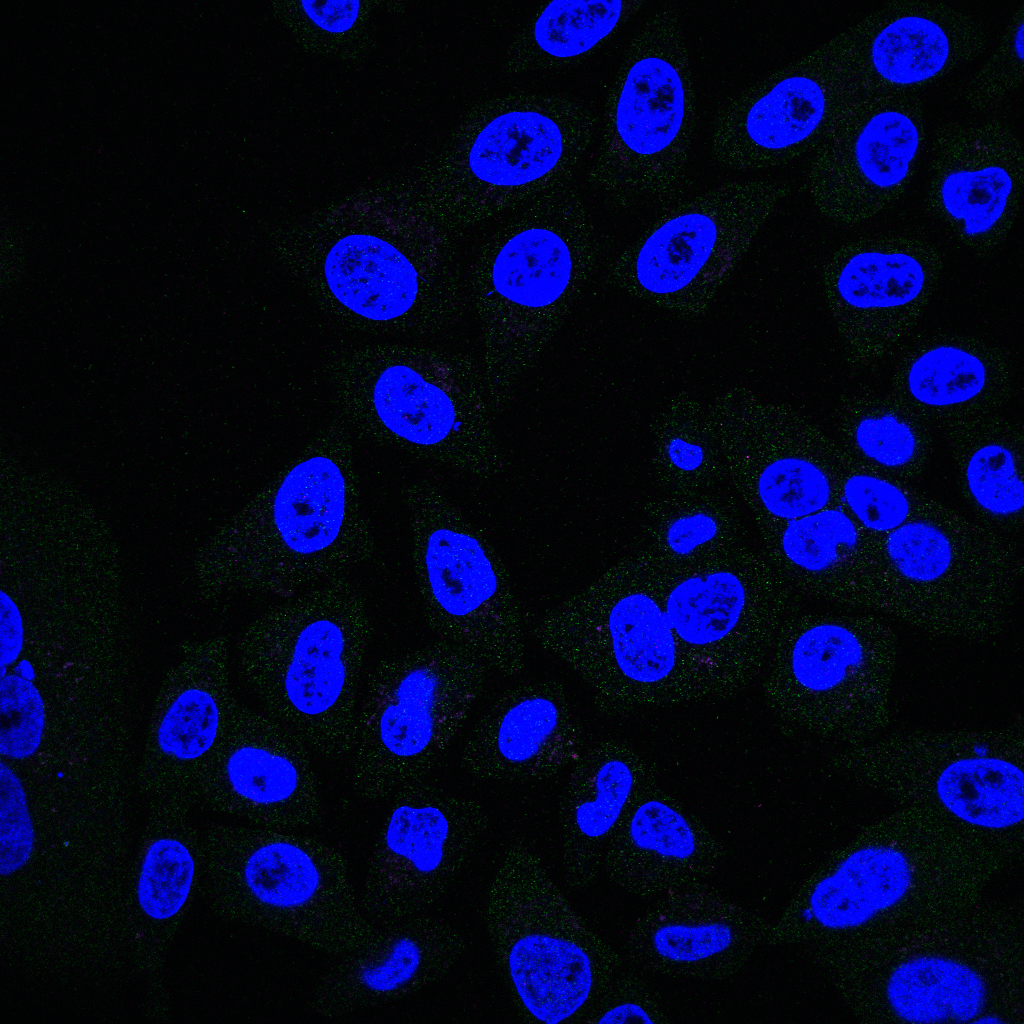

Supplement: Supplementary file 10 — Source Data for Figure 6 [file EMBR-24-e57300-s012.zip › Fig 6/6D/hexa KO_non-treated.tif]

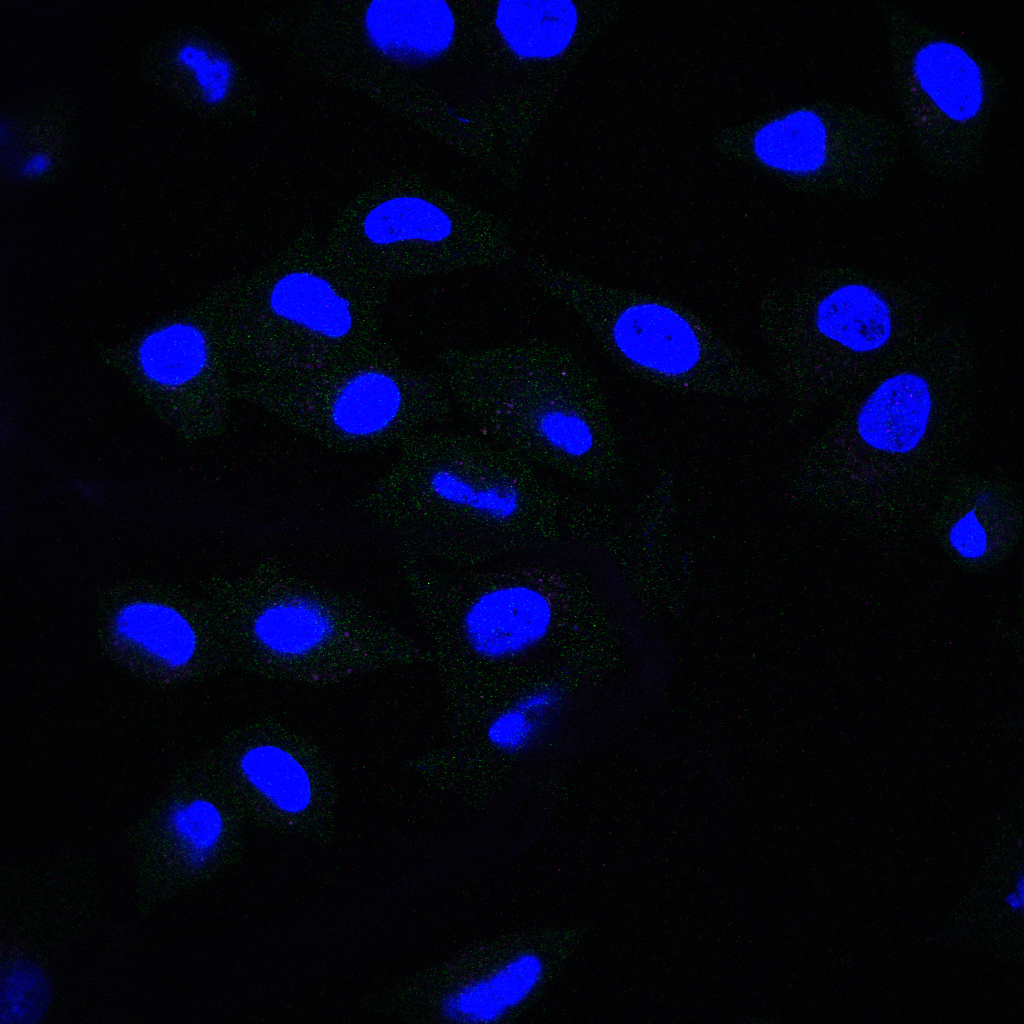

Supplement: Supplementary file 10 — Source Data for Figure 6 [file EMBR-24-e57300-s012.zip › Fig 6/6D/WT_non-treated.tif]

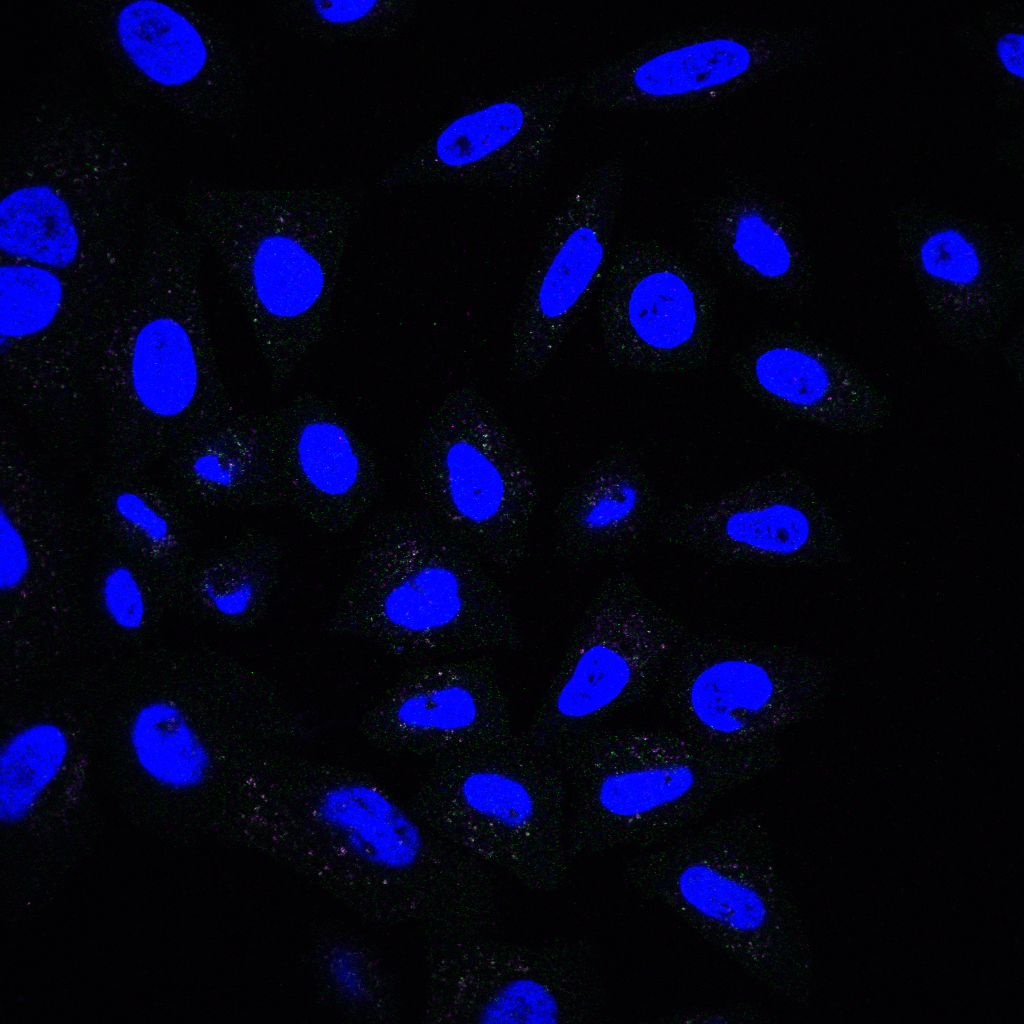

Supplement: Supplementary file 10 — Source Data for Figure 6 [file EMBR-24-e57300-s012.zip › Fig 6/6D/hexa KO_LLOMe.tif]

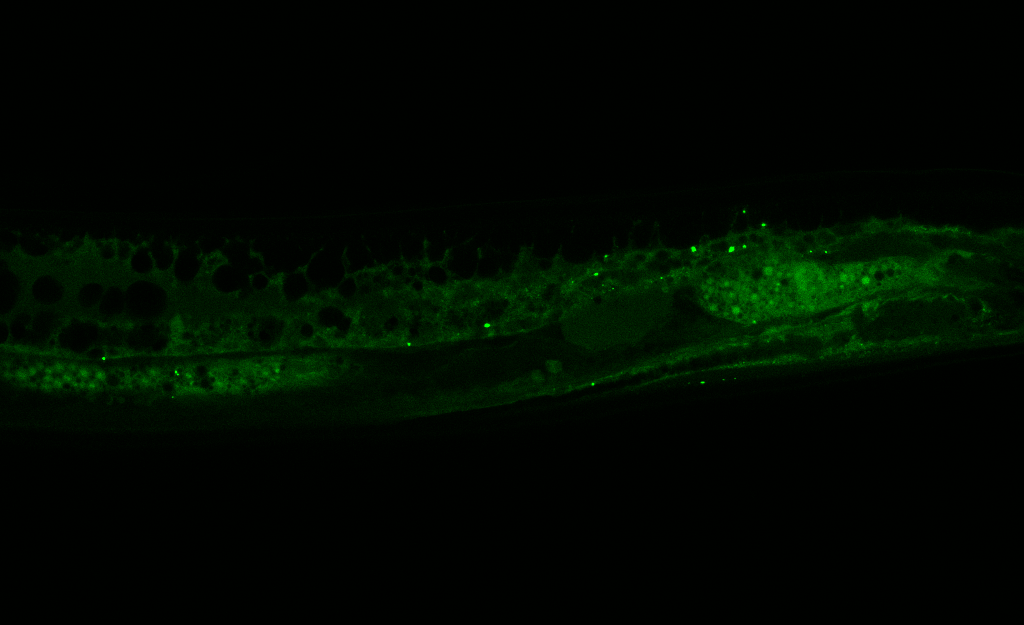

Supplement: Supplementary file 11 — Source Data for Figure 7 [file EMBR-24-e57300-s010.zip › Fig 7/7M/lgg-2 KD.tif]

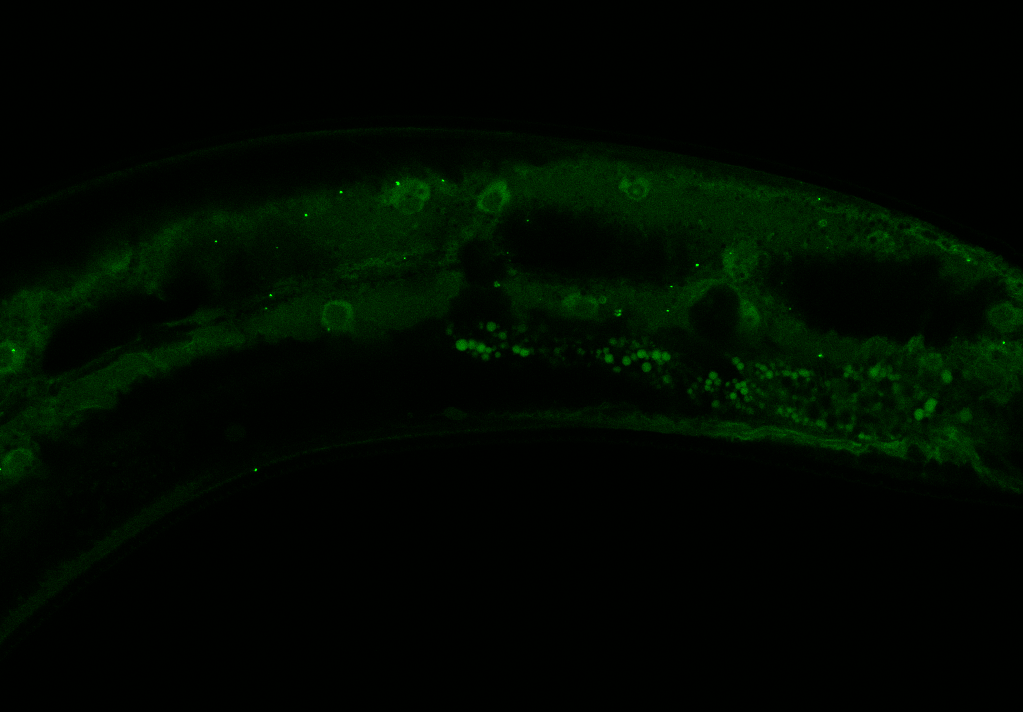

Supplement: Supplementary file 11 — Source Data for Figure 7 [file EMBR-24-e57300-s010.zip › Fig 7/7M/control.tif]

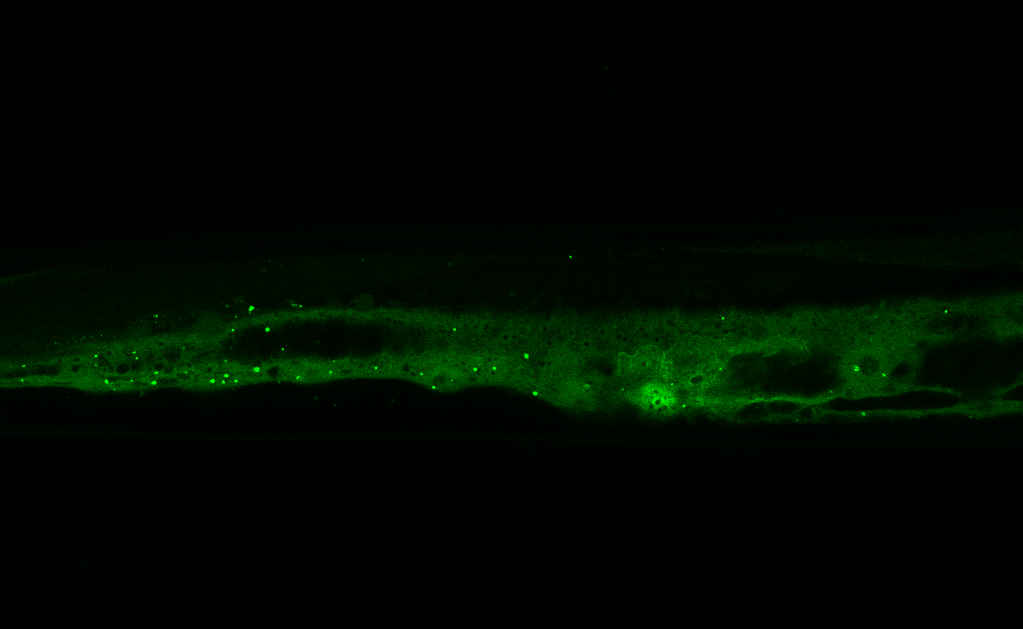

Supplement: Supplementary file 11 — Source Data for Figure 7 [file EMBR-24-e57300-s010.zip › Fig 7/7M/lgg-1 KD.tif]

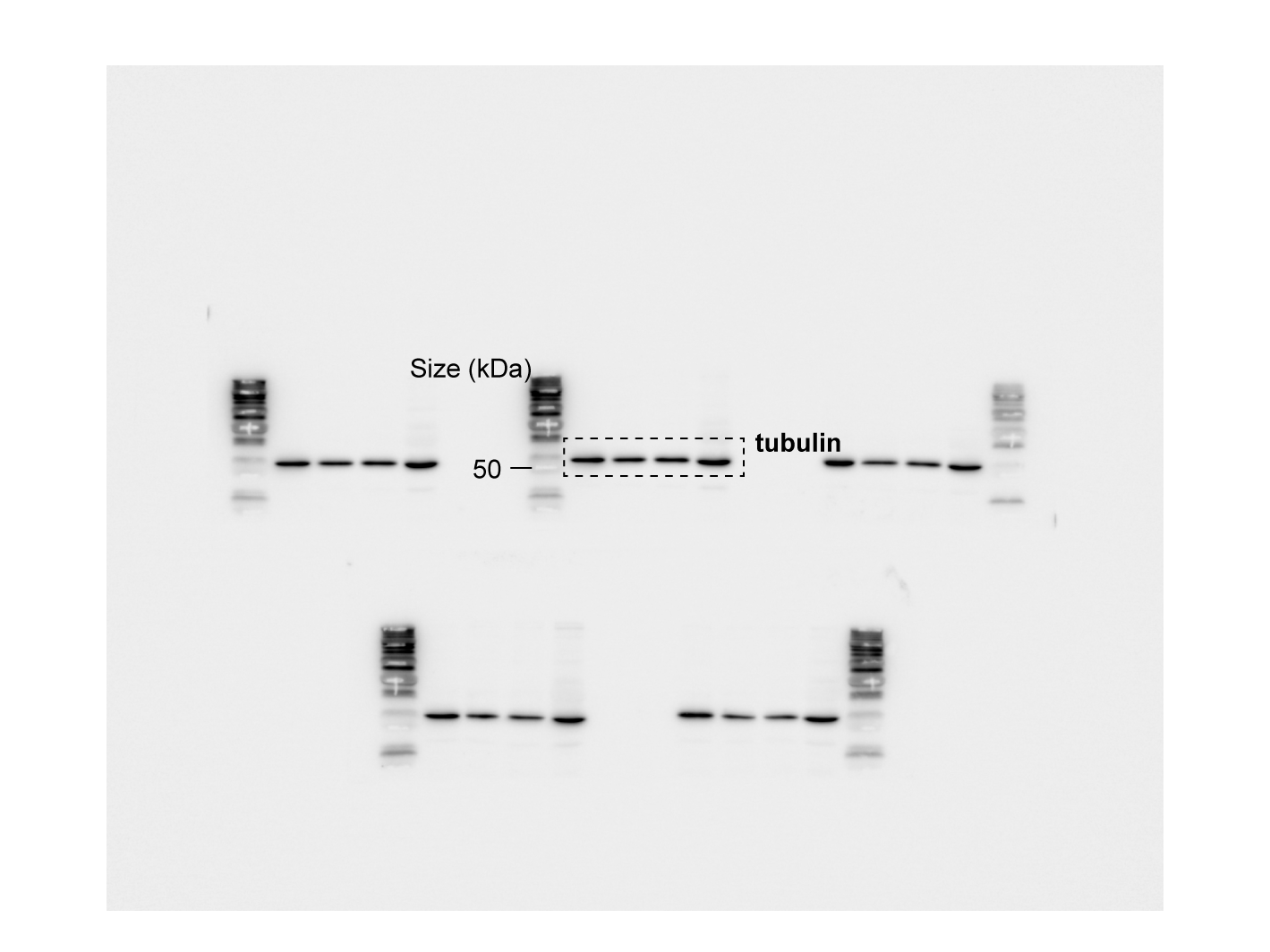

Supplement: Supplementary file 11 — Source Data for Figure 7 [file EMBR-24-e57300-s010.zip › Fig 7/7C/western tubulin.tif]

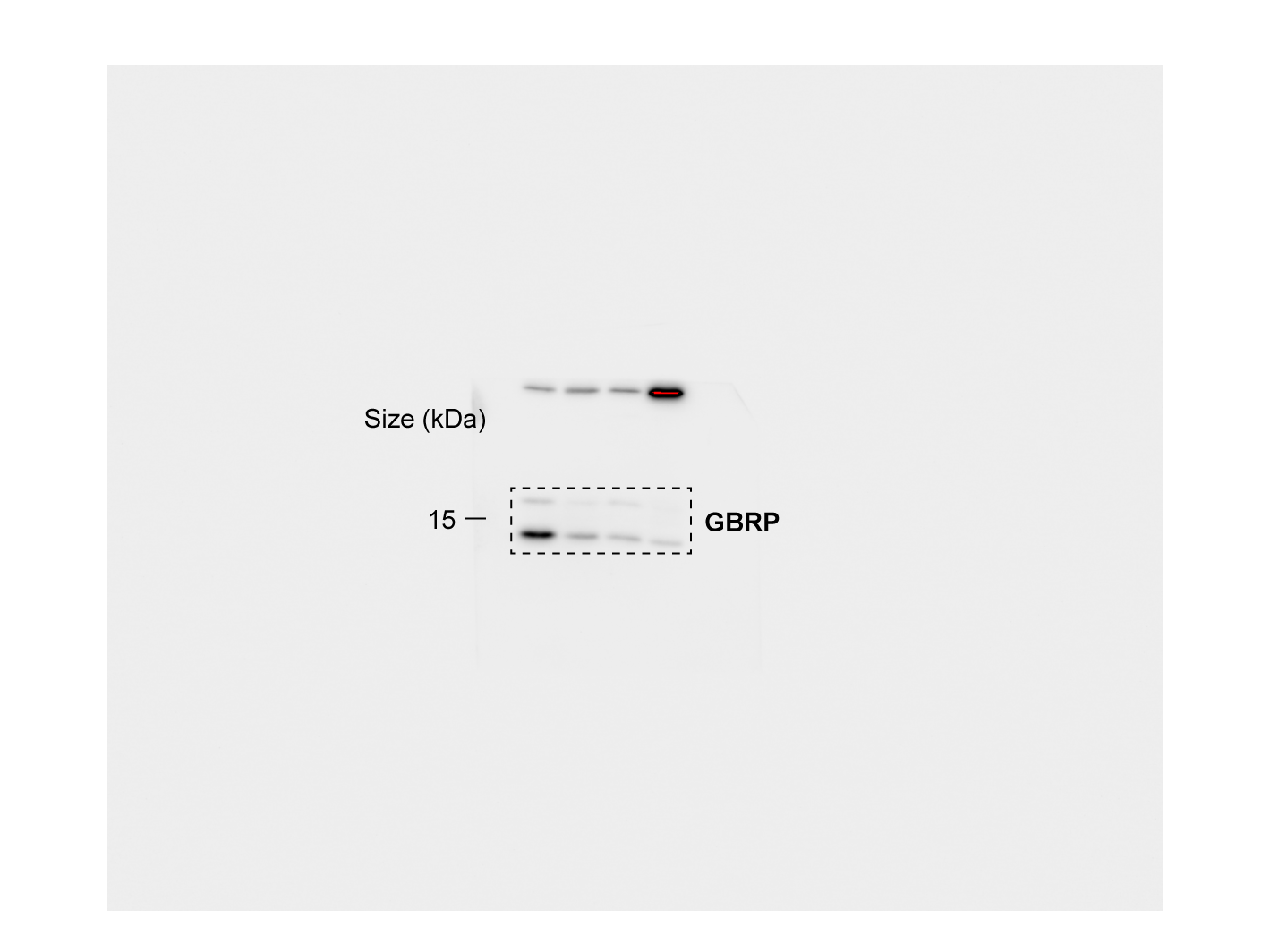

Supplement: Supplementary file 11 — Source Data for Figure 7 [file EMBR-24-e57300-s010.zip › Fig 7/7C/western GBRP.tif]

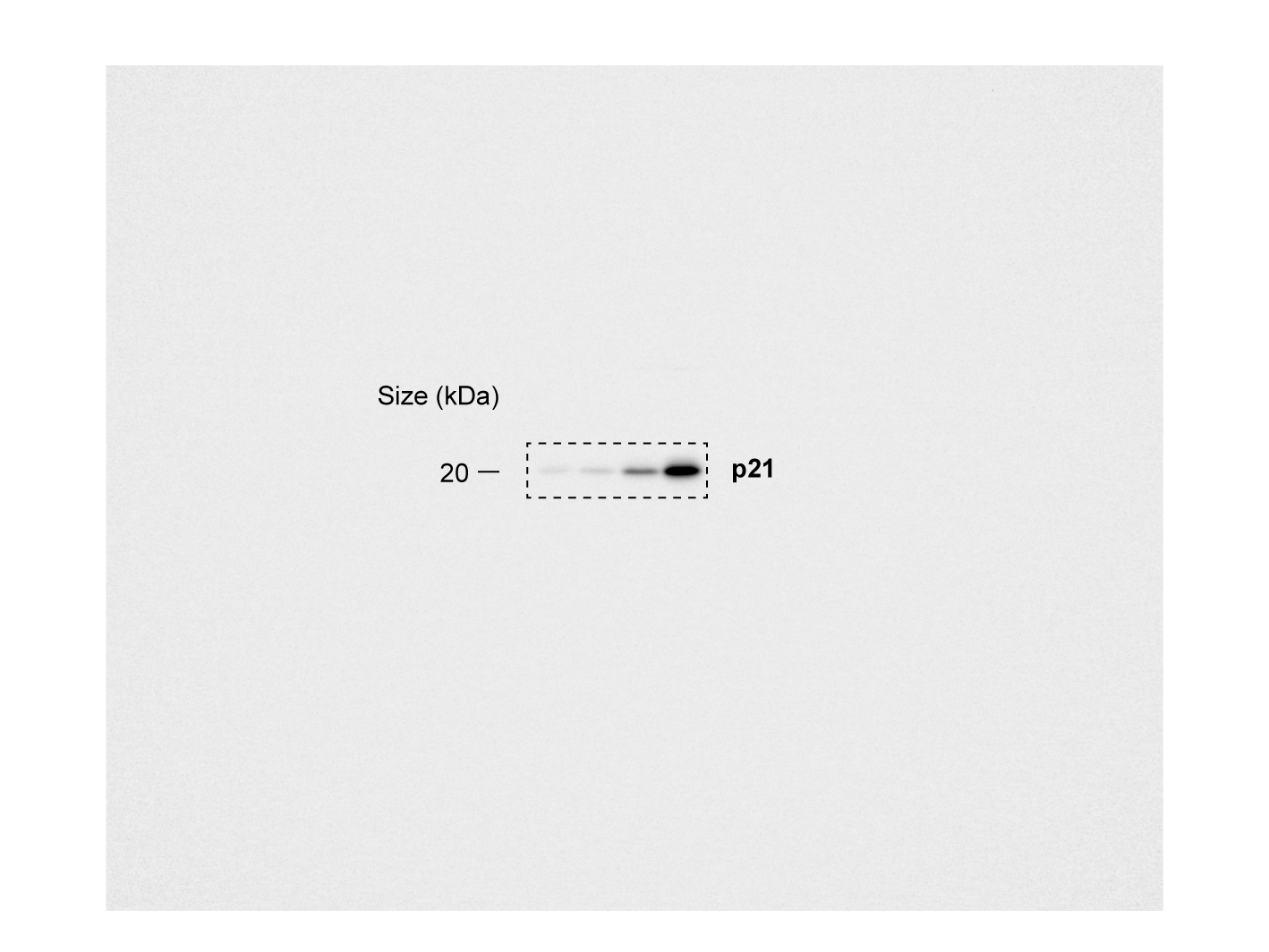

Supplement: Supplementary file 11 — Source Data for Figure 7 [file EMBR-24-e57300-s010.zip › Fig 7/7C/western p21.tif]

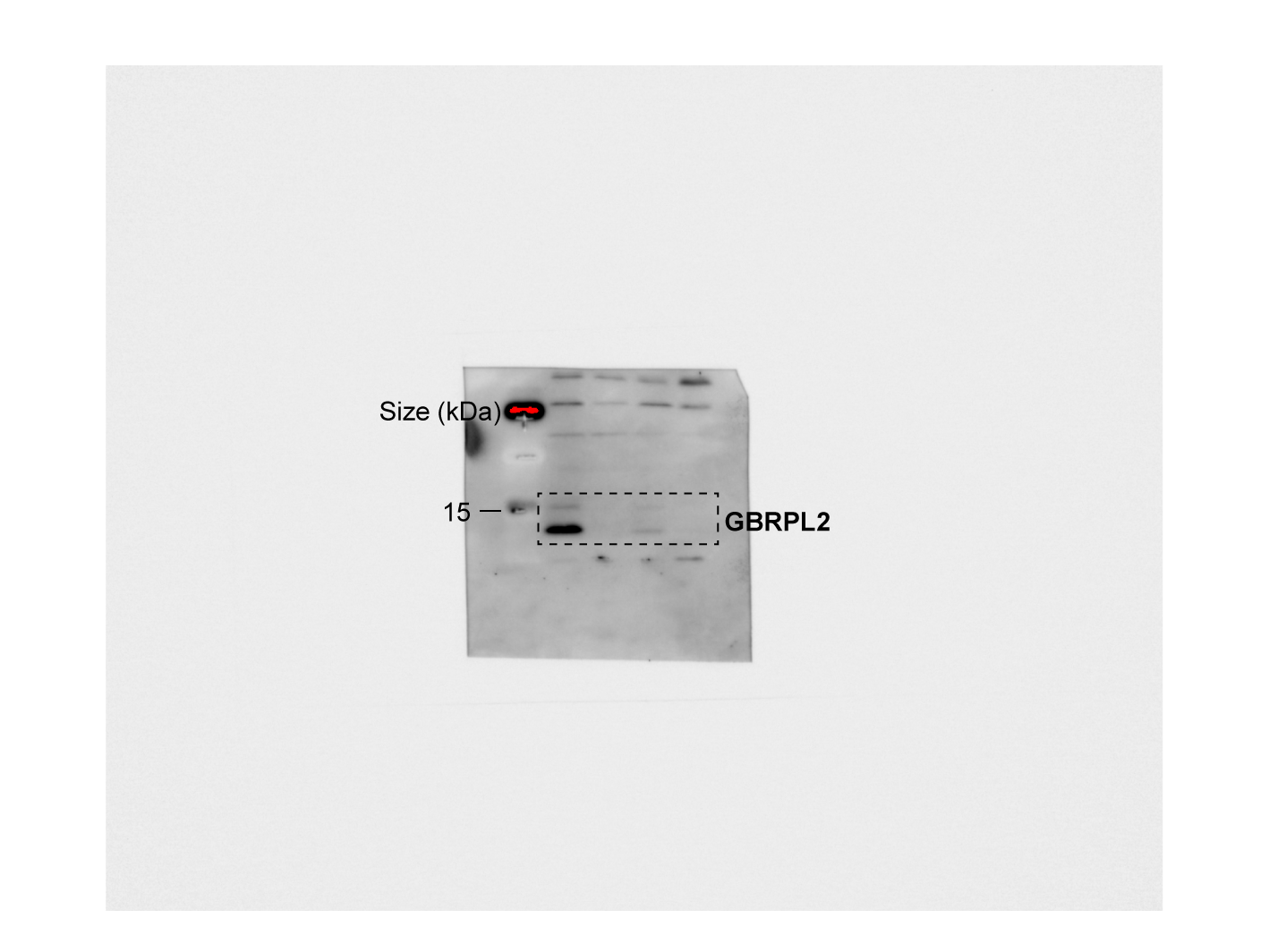

Supplement: Supplementary file 11 — Source Data for Figure 7 [file EMBR-24-e57300-s010.zip › Fig 7/7C/western GBRPL2.tif]

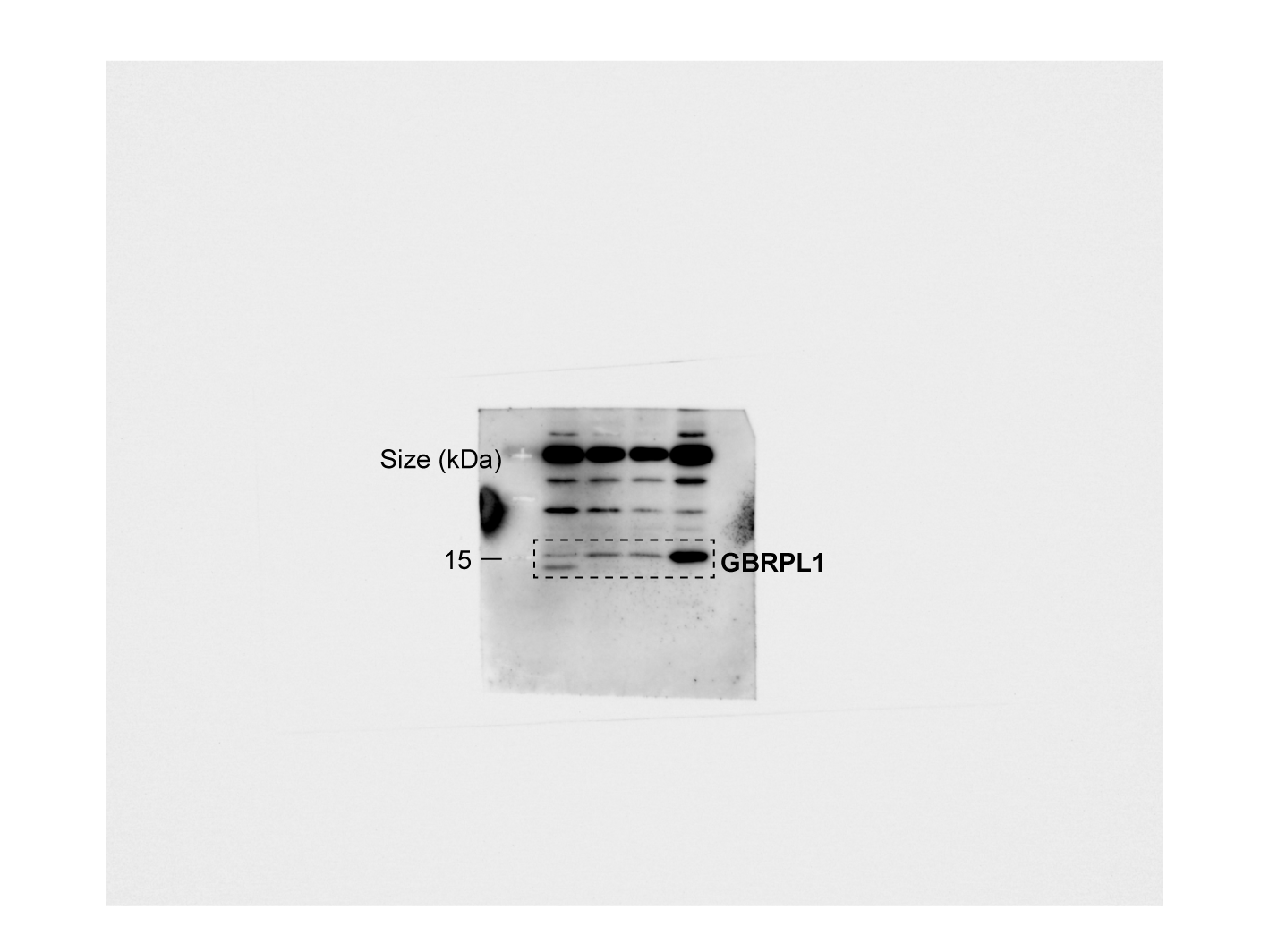

Supplement: Supplementary file 11 — Source Data for Figure 7 [file EMBR-24-e57300-s010.zip › Fig 7/7C/western GBRPL1.tif]

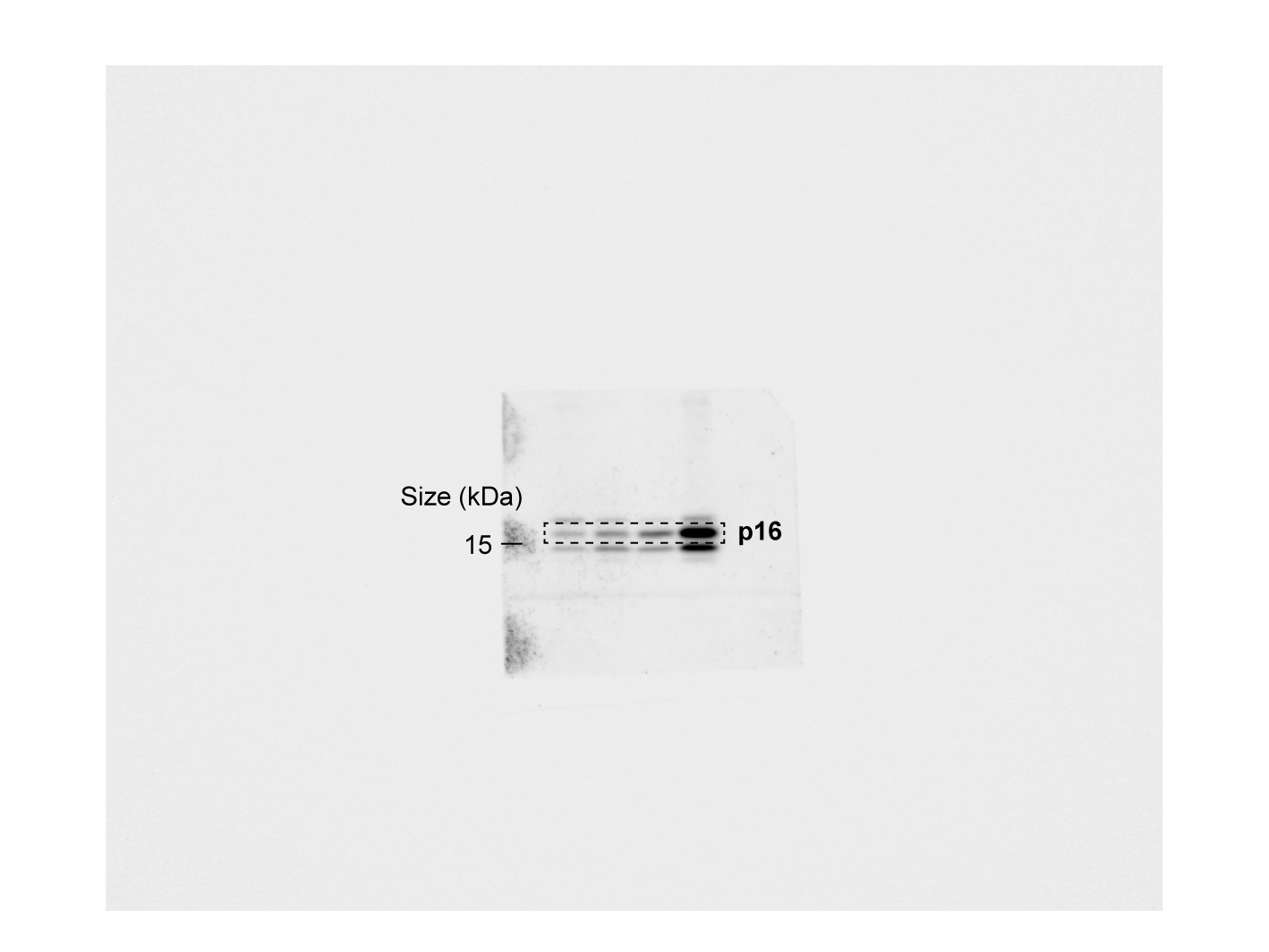

Supplement: Supplementary file 11 — Source Data for Figure 7 [file EMBR-24-e57300-s010.zip › Fig 7/7C/western p16.tif]

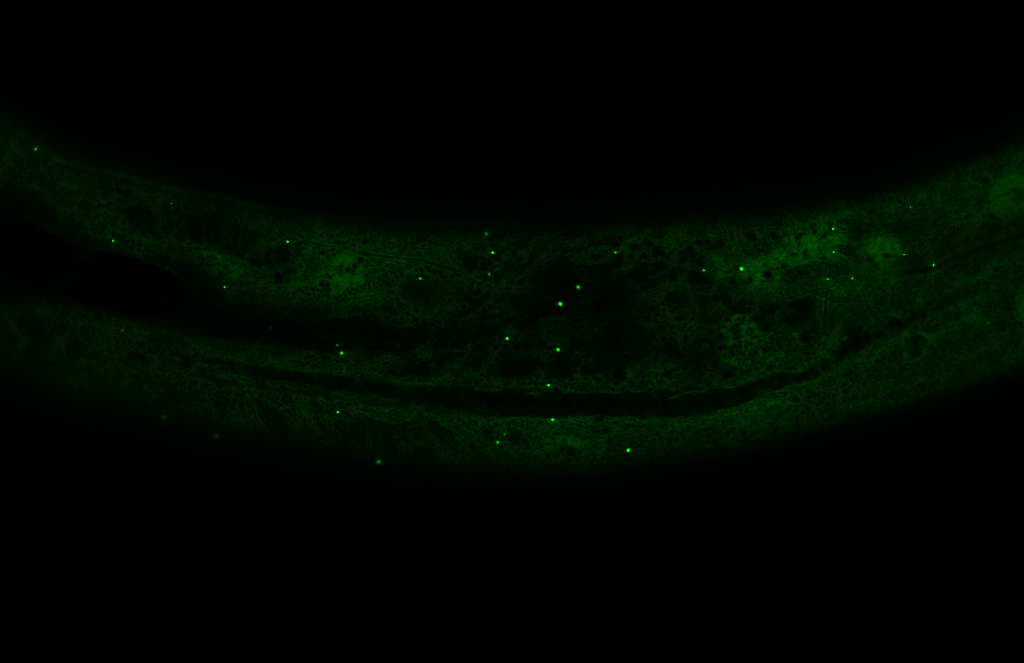

Supplement: Supplementary file 11 — Source Data for Figure 7 [file EMBR-24-e57300-s010.zip › Fig 7/7K/N2.tif]

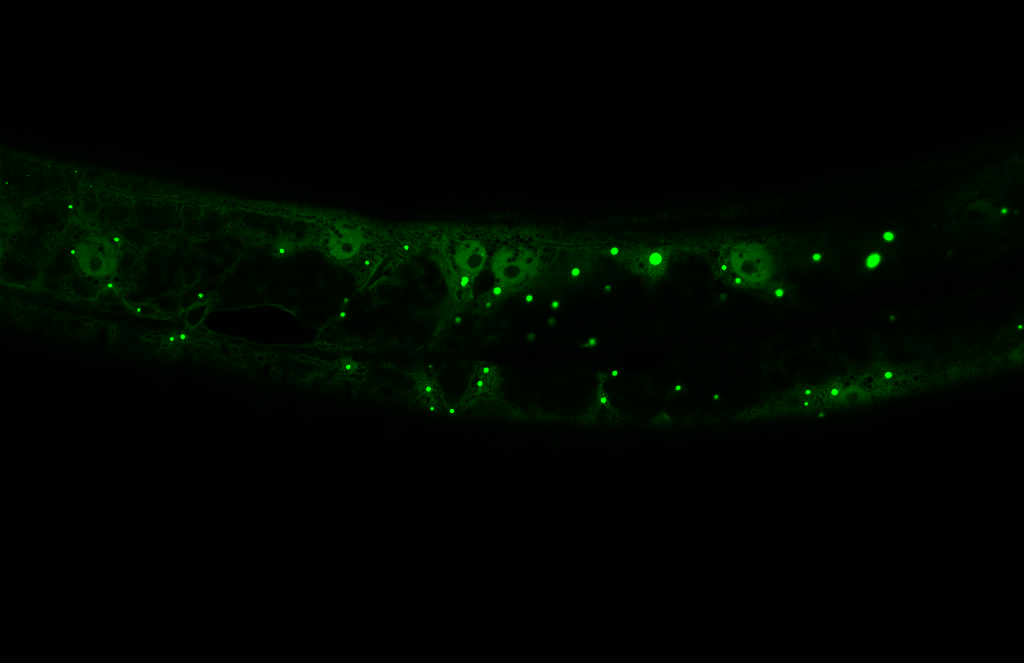

Supplement: Supplementary file 11 — Source Data for Figure 7 [file EMBR-24-e57300-s010.zip › Fig 7/7K/sax-1.tif]

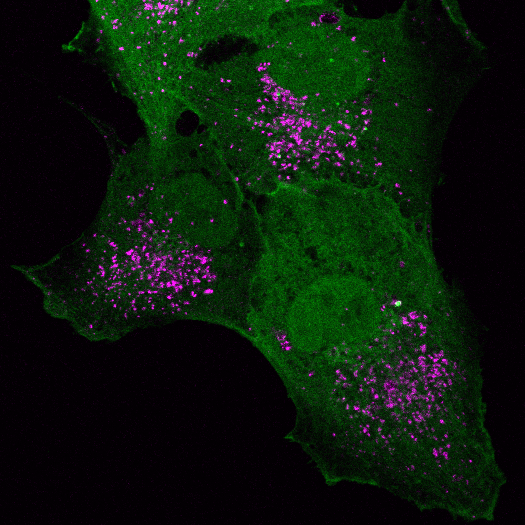

Supplement: Supplementary file 11 — Source Data for Figure 7 [file EMBR-24-e57300-s010.zip › Fig 7/7I/siSTK38 #1_- DXR.tif]

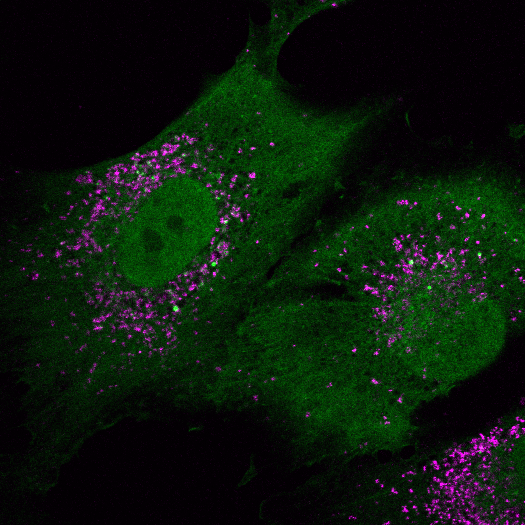

Supplement: Supplementary file 11 — Source Data for Figure 7 [file EMBR-24-e57300-s010.zip › Fig 7/7I/siGBRP_- DXR.tif]

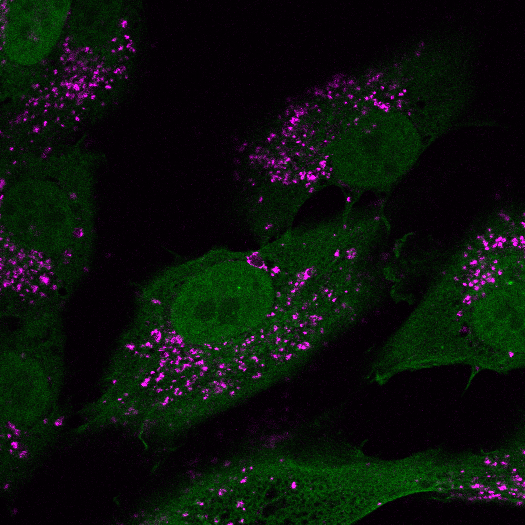

Supplement: Supplementary file 11 — Source Data for Figure 7 [file EMBR-24-e57300-s010.zip › Fig 7/7I/siLuc_- DXR.tif]

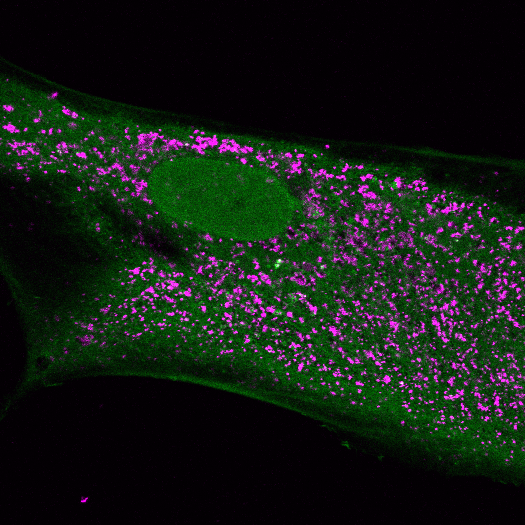

Supplement: Supplementary file 11 — Source Data for Figure 7 [file EMBR-24-e57300-s010.zip › Fig 7/7I/siLuc_+ DXR.tif]

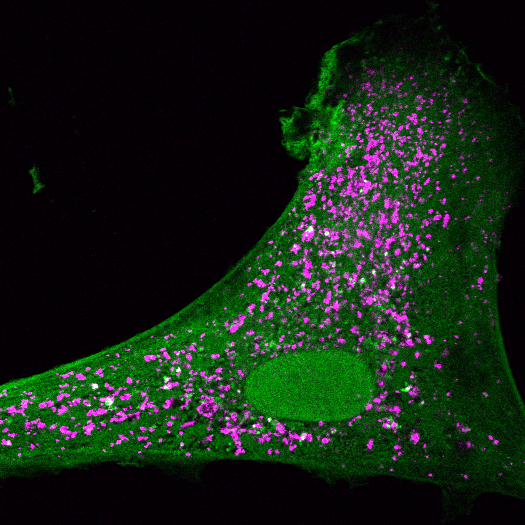

Supplement: Supplementary file 11 — Source Data for Figure 7 [file EMBR-24-e57300-s010.zip › Fig 7/7I/siGBRP_+ DXR.tif]

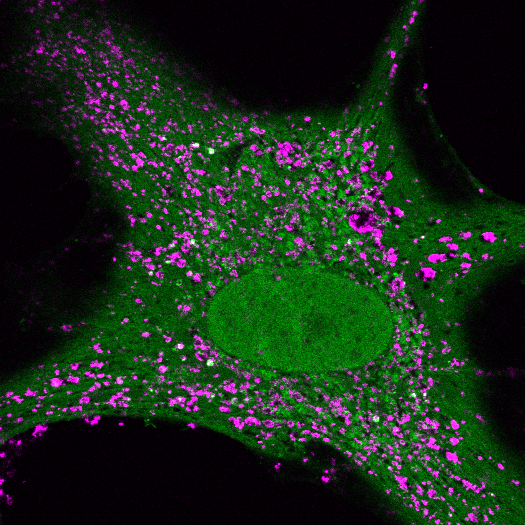

Supplement: Supplementary file 11 — Source Data for Figure 7 [file EMBR-24-e57300-s010.zip › Fig 7/7I/siSTK38 #1_+ DXR.tif]

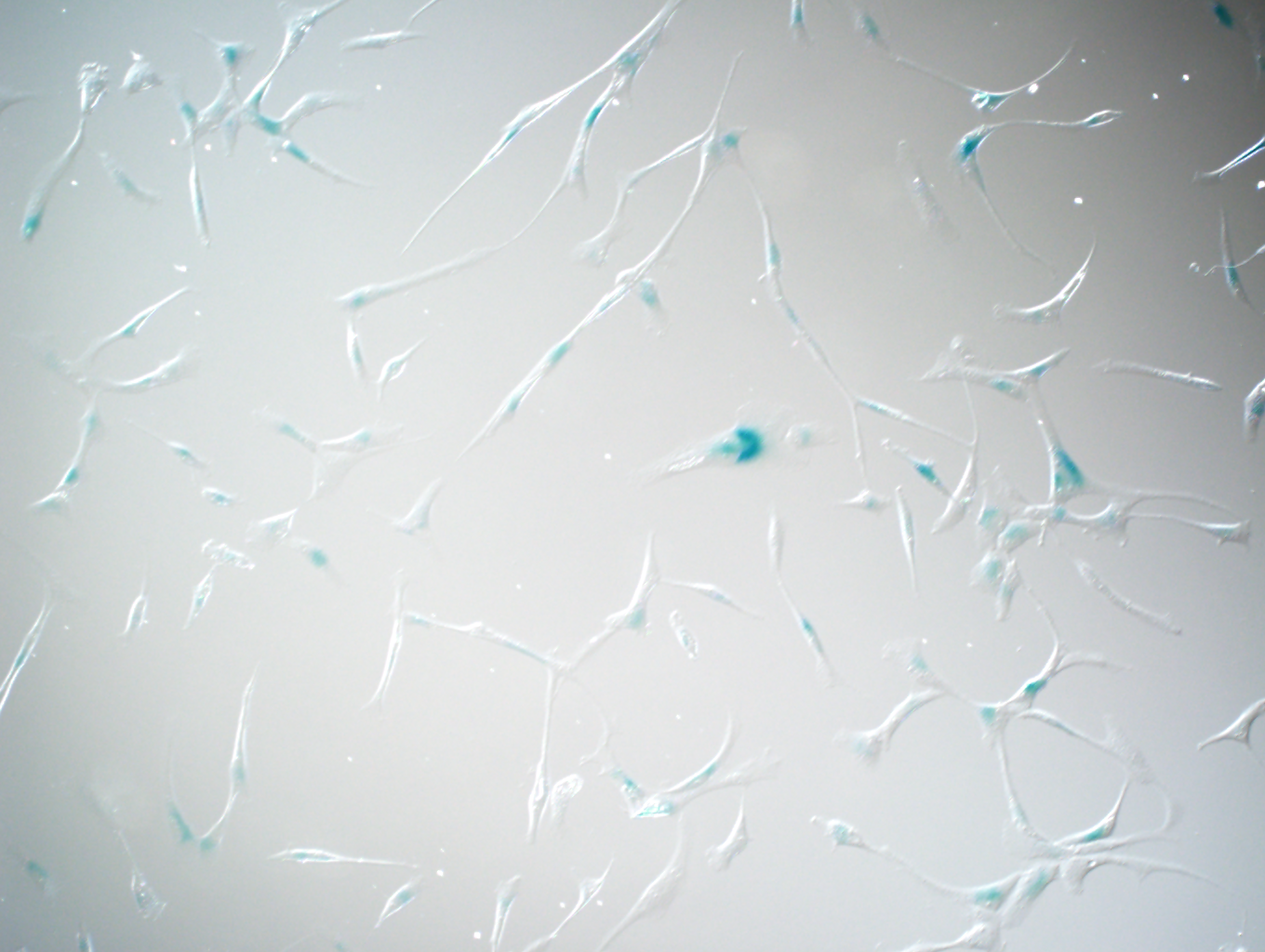

Supplement: Supplementary file 11 — Source Data for Figure 7 [file EMBR-24-e57300-s010.zip › Fig 7/7G/siGBRP.tif]

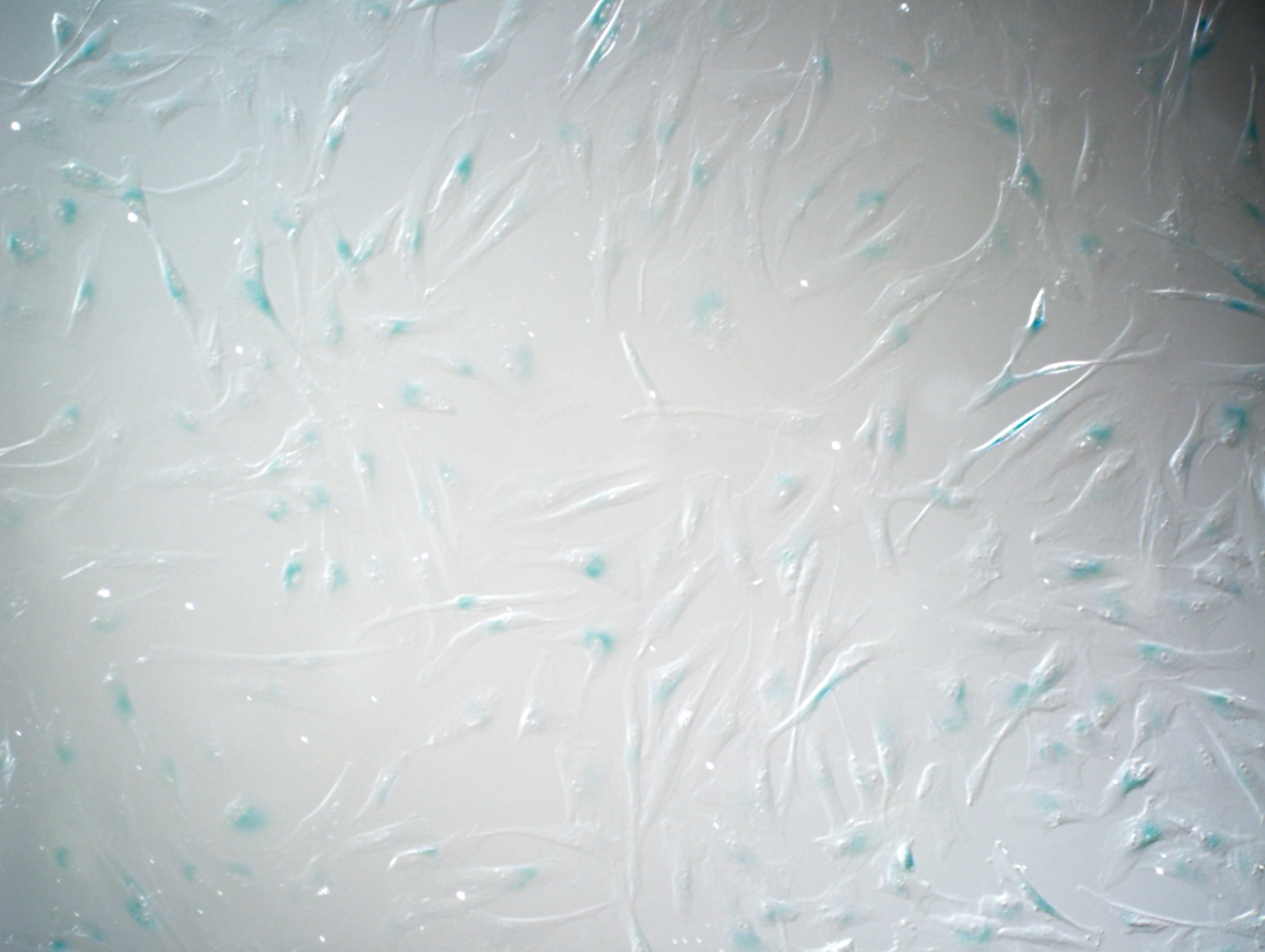

Supplement: Supplementary file 11 — Source Data for Figure 7 [file EMBR-24-e57300-s010.zip › Fig 7/7G/siLuc.tif]

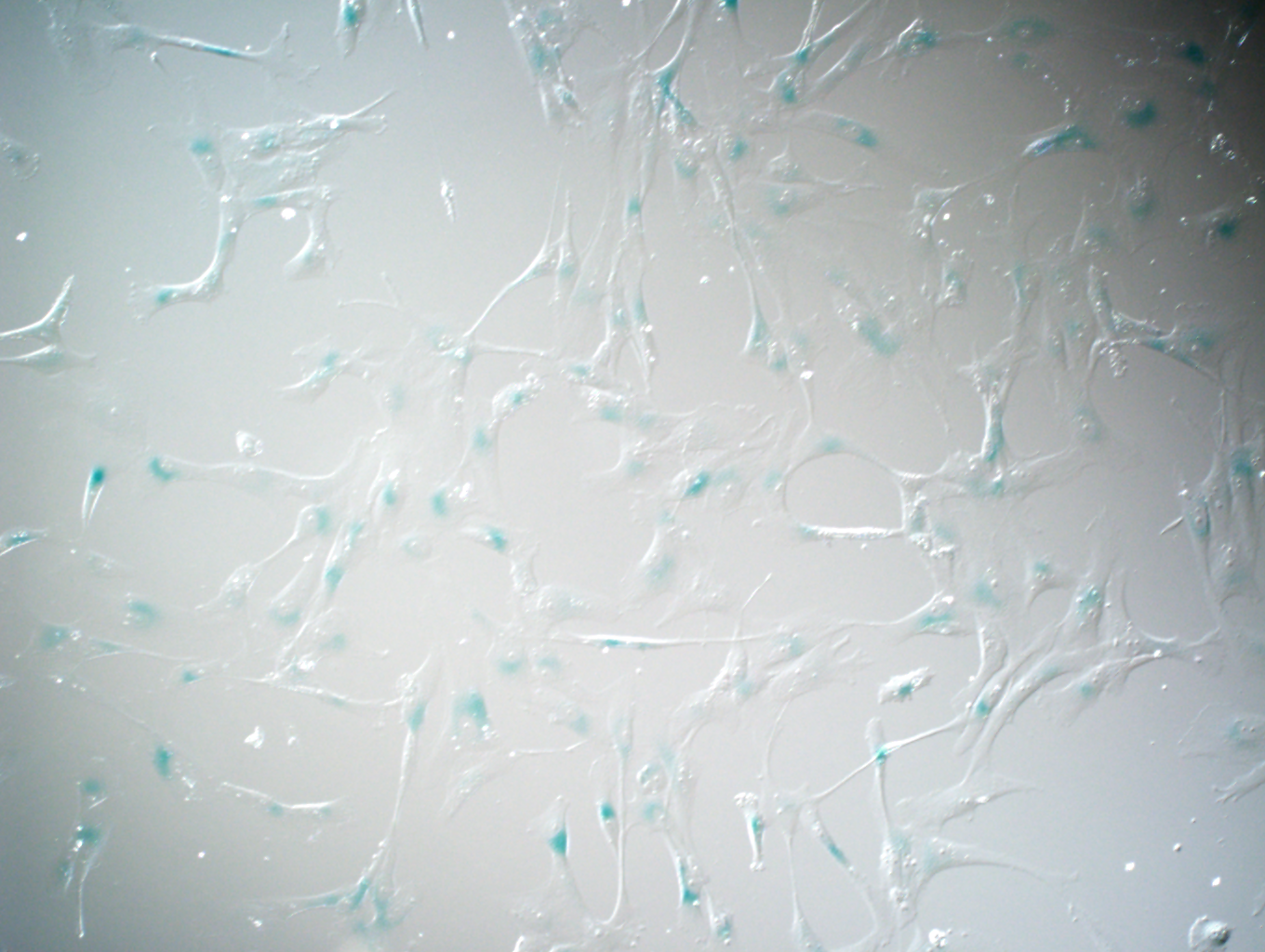

Supplement: Supplementary file 11 — Source Data for Figure 7 [file EMBR-24-e57300-s010.zip › Fig 7/7G/siSTK38 #1.tif]

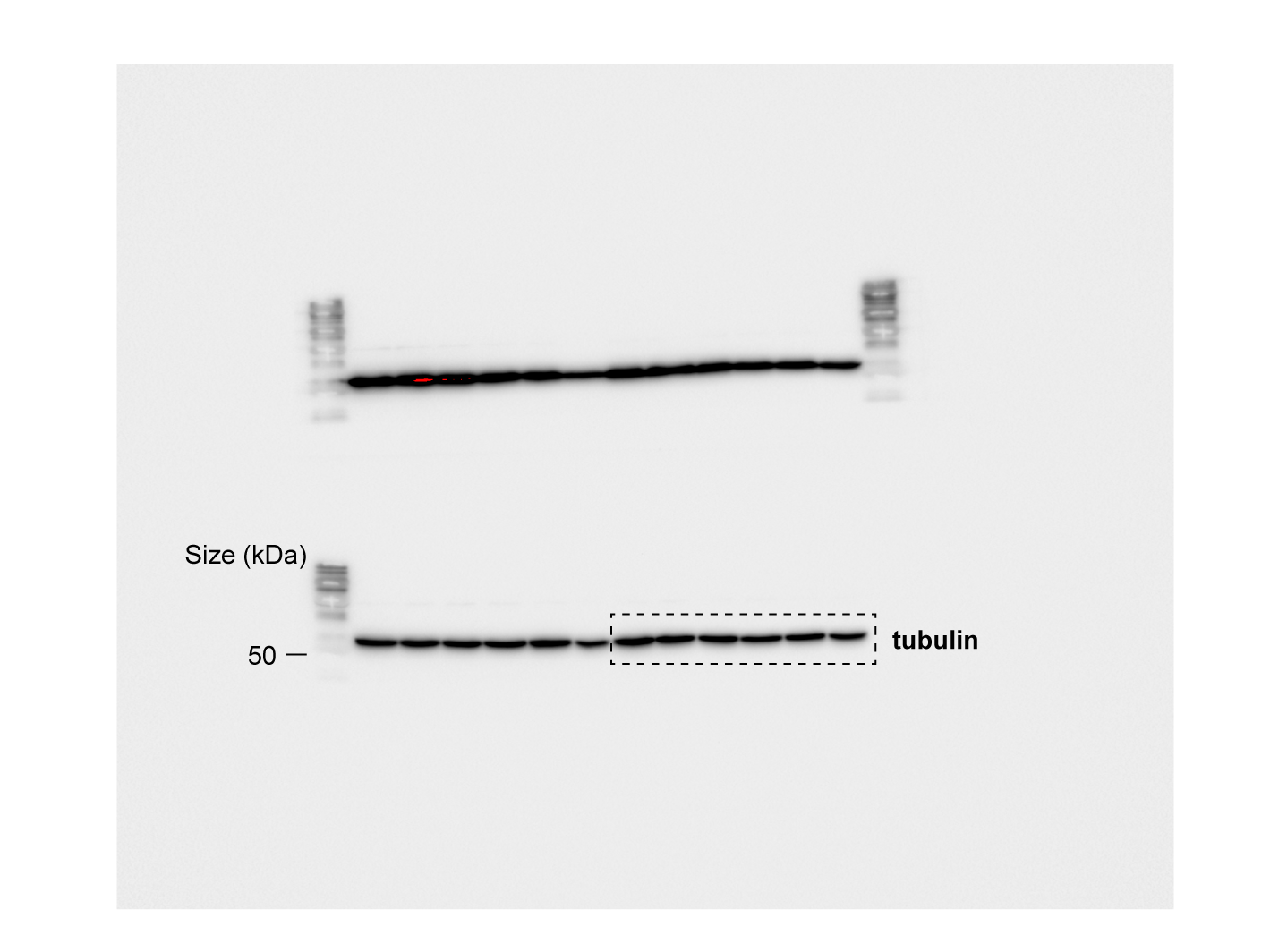

Supplement: Supplementary file 11 — Source Data for Figure 7 [file EMBR-24-e57300-s010.zip › Fig 7/7A/western tubulin.tif]

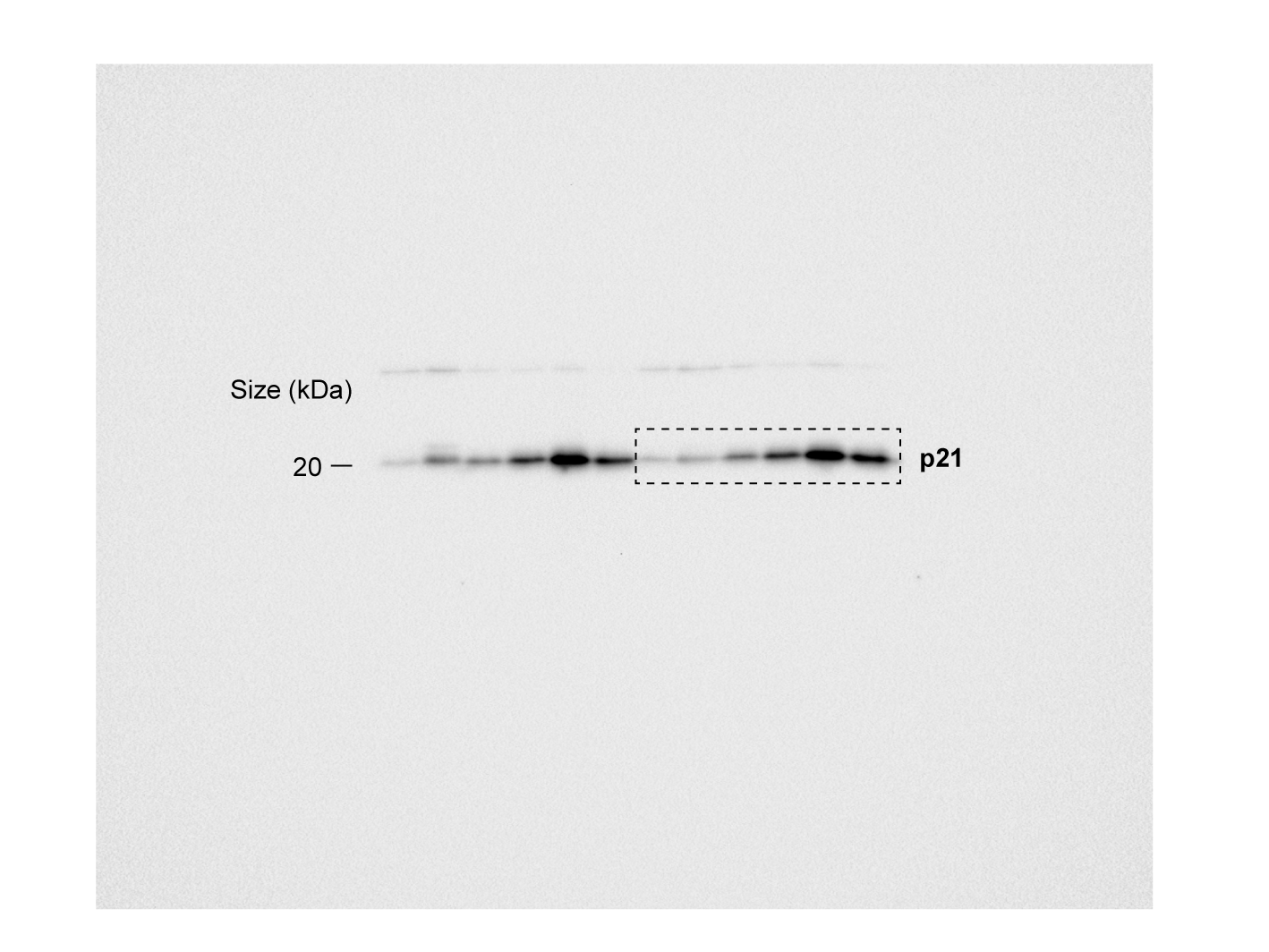

Supplement: Supplementary file 11 — Source Data for Figure 7 [file EMBR-24-e57300-s010.zip › Fig 7/7A/western p21.tif]

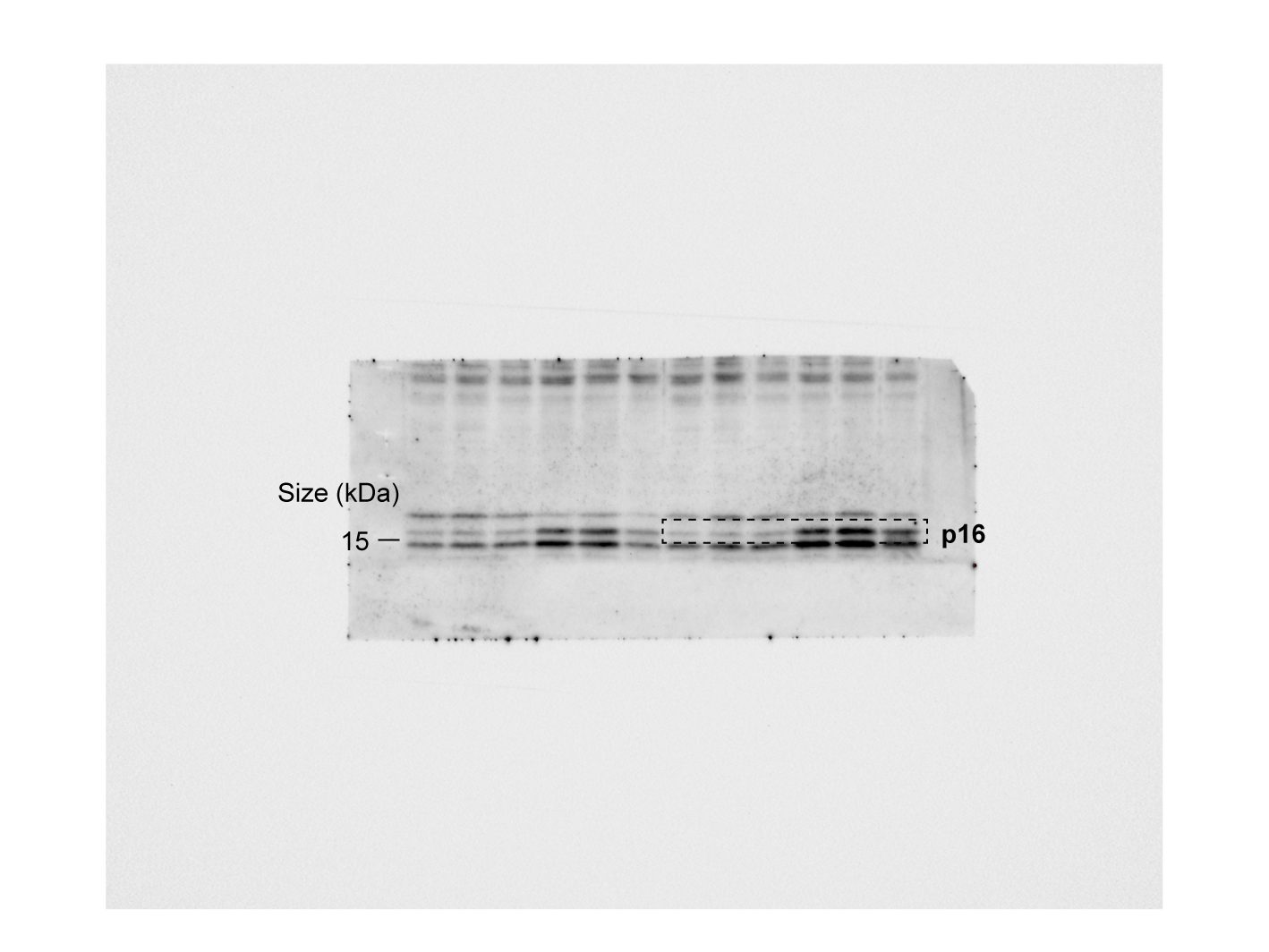

Supplement: Supplementary file 11 — Source Data for Figure 7 [file EMBR-24-e57300-s010.zip › Fig 7/7A/western p16.tif]

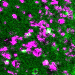

Supplement: Supplementary file 11 — Source Data for Figure 7 [file EMBR-24-e57300-s010.zip › Fig 7/7I/magnified images/siSTK38 #1_+ DXR_Merge.tif]

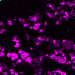

Supplement: Supplementary file 11 — Source Data for Figure 7 [file EMBR-24-e57300-s010.zip › Fig 7/7I/magnified images/siLuc_+ DXR_LAMP1.tif]

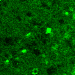

Supplement: Supplementary file 11 — Source Data for Figure 7 [file EMBR-24-e57300-s010.zip › Fig 7/7I/magnified images/siSTK38 #1_+ DXR_GFP-Gal3.tif]

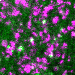

Supplement: Supplementary file 11 — Source Data for Figure 7 [file EMBR-24-e57300-s010.zip › Fig 7/7I/magnified images/siGBRP_+ DXR_Merge.tif]

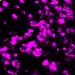

Supplement: Supplementary file 11 — Source Data for Figure 7 [file EMBR-24-e57300-s010.zip › Fig 7/7I/magnified images/siSTK38 #1_+ DXR_LAMP1.tif]

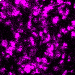

Supplement: Supplementary file 11 — Source Data for Figure 7 [file EMBR-24-e57300-s010.zip › Fig 7/7I/magnified images/siGBRP_+ DXR_LAMP1.tif]

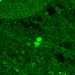

Supplement: Supplementary file 11 — Source Data for Figure 7 [file EMBR-24-e57300-s010.zip › Fig 7/7I/magnified images/siLuc_+ DXR_GFP-Gal3.tif]

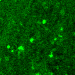

Supplement: Supplementary file 11 — Source Data for Figure 7 [file EMBR-24-e57300-s010.zip › Fig 7/7I/magnified images/siGBRP_+ DXR_GFP-Gal3.tif]

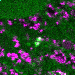

Supplement: Supplementary file 11 — Source Data for Figure 7 [file EMBR-24-e57300-s010.zip › Fig 7/7I/magnified images/siLuc_+ DXR_Merge.tif]
