# Supplementary material for: Microautophagy regulated by STK38 and GABARAPs is essential to repair lysosomes and prevent aging
Source: EMBO Rep. 2023 Nov 21;24(12):e57300. doi: 10.15252/embr.202357300 (PMC10702834; doi:10.15252/embr.202357300)
Supplement: Supplementary file 12 [file EMBR-24-e57300-s008.pdf]

EMBO reports

Microautophagy regulated by STK38 and GABARAPs is essential to repair lysosomes and prevent aging

Monami Ogura, Tatsuya Kaminishi, Takayuki Shima, Miku Torigata, Nao Bekku, Keisuke Tabata, Satoshi Minami, Kohei Nishino, Akiko Nezu, Maho Hamasaki, Hidetaka Kosako, Tamotsu Yoshimori, and Shuhei Nakamura
DOI: 10.15252/embr.202357300

Corresponding author(s): Shuhei Nakamura (K175569@naramed-u.ac.jp) , Tamotsu Yoshimori (yoshimori.tamotsu.med@osaka-u.ac.jp)

Review Timeline:

Transfer Date:	5th Apr 23
Editorial Decision:	6th Apr 23
Revision Received:	27th Sep 23
Editorial Decision:	18th Oct 23
Revision Received:	24th Oct 23
Accepted:	25th Oct 23

Editor: Martina Rembold

Transaction Report: This manuscript was transferred to EMBO *reports* following peer review at the EMBO Journal.

Referee #1 Review
Received: 2nd Mar 23

Report for Author:
The authors of this study investigated the role of STK38 in regulating lysosomal repair by microautophagy after damage. They found that STK38 is recruited to lysosomes after lysosomal damage induced by LLOMe, that the C-terminal domain of STK38 is important for recruitment to lysosomes while the N-terminus is dispensable and that KD of STK38 leads to a sustained and enhanced formation of ESCRT puncta and a concomitant reduction of VPS4 puncta in an LLOMe dependent manner. They found that the recruitment of STK38 to LAMP1 positive structures was independent of canonical autophagy proteins including FIP200, ATG14, ATG9, ATG7 and STX17 arguing that STK38 regulates lysosome repair by microautophagy. They also identified substrates of STK38 that are phosphorylated upon lysosomal damage, but the relationship of the identified proteins to lysosomal repair was not further studied. They also correlated STK38 function in lysosomal repair with senescence and longevity, but both aspects remain mechanistically unconnected and premature. The major claim of the authors that STK38 is essential for lysosomal repair by recruiting VPS4 to induce recycling of ESCRT-III components is not supported by the experimental data for reasons that are mentioned in the specific points listed below.
Given the strong limitations of the study and the significant concerns, the manuscript is not suited for publication in the EMBO journal.

1) There is only a small fraction of LAMP1 positive structures colocalizing with STK38 upon induction of lysosome damage. Moreover, knockdown of STK38 has only a mild impact on lysosomal clearance compared to MOB2 or LATS2. The authors should quantify the knockdown efficiency for the different members of the HIPO pathway shown in EV1 A. This is particularly important since KD of LATS2 has an even stronger effect than KD of STK38. Moreover, both, STK38 and LATS2 are regulated by upstream kinases, which might act in a redundant manner so that KD of only one can be compensated by other. The authors should perform co-KD of MST1/2/3 or combinations of two.
2) The LLOMe induced phosphorylation of T444 (Fig. EV1E) is not very convincing. First, there is no obvious change in the phosphorylation status of STK38 based on western blots. The authors need to verify i) the identity of the bands (in the reference blots from the vendor there is only a single band visible for pSTK38, the double band is not visible), and quantify the phosphorylated STK38 fraction (normalized to total STK38 expressed and corrected by loading control). Second, the T444A mutant still shows robust colocalization with LAMP1 while the effect of deleting the C-terminal part of STK38 has a much stronger effect.

3) The detection of damaged lysosomes by colocalization with LAMP1 after LLOMe treatment is too unspecific. The authors should include co-localization studies of STK38 in wt and siRNA treated cells with Gal3 (Fig 1E, Fig EV 1C, Fig EV 2A).
4) Knockdown of FIP200, ATG14, ATG7 and STX17 appears to induce an even more pronounced colocalization of STK38 with LAMP1, indicating that disruption of macroautophagy leads to an accumulation of damaged lysosomes to which STK38 is recruited. To determine, which of the two pathways is predominant, the authors should investigate colocalization of FIP200 with lysosomes in STK38 KO/KD (for STK38 KD, the efficiency of siRNA treatment need to be verified, see point 1).
5) The authors argue that KD of STK38 has no impact on starvation induced autophagy, but in nonstarved cells, much less LC3B-II is detected in BafA1 treated cells. What is the reason for this strong decline (Fig EV 2C).
6) The authors demonstrated KD of STK38 by westernblot (Fig. EV2C) referring to the upper band of the two bands detected and cutting the lower band, which was even in the KD very strong. In Fig. EV1E, they referred to the lower band for STK38, indicating that the band that has been cut in Fig. EV2C corresponds to STK38. Moreover, the reference blots of the vendor did not show a double band. The authors need to verify the identity of the bands to reveal why they detect two bands and which one corresponds to STK38. This is very important because most data of the paper are based on comparing STK38 expressing and KD cells.
7) The reduction of VPS4 puncta in the STK38 KO is much less significant than in the KD. The same is true for ALIX puncta. If STK38 is required to recruit VPS4, one would expect to see a much stronger effect. The authors should better characterize what type of puncta they count by costaining of VPS4 and Gal3 in WT and STK KD and KO cells.
8) The authors used the GFP fused lysosomal channel TRPML1 as a marker for micolysophagy. However, TRPML1 will also be degraded by macrolysophagy. It is not clear why the GFP-TRPML1 degradation assay was chosen to distinguish between both options.
9) Why did the authors used LLOMe in some experiments (Fig. 1, 2 and EV 1-5) but nigericin in this experiment? Different ways to induce lysosomal damage or stress could result in the activation of different cellular response pathways. This is even more evident as using LLOMe in EGFP-TRPML1 degradation assays has almost no impact on the generation of free EGFP. Only a minor fraction of EGFP-TRPML1 is degraded (free EGFP band is extremely weak in control experiments) and a further reduction of the intensity in STK38 KD (Fig. 3D-G) is not very convincing.
10) Based on EM images shown in Fig 3H, the authors argue that treatment of cells with NH4Cl induces microautophagy resulting in the formation of intraluminal vesicles in lysosomes. However, the structures in the siLuc NH4Cl sample look like MVBs and not like lysosomes, while those in non-treated conditions contain not only ILVs, but also other material, what would be expected. To clarify this, the authors need to perform CLEM experiments using lyosomal markers and compare this with CLEM using MVB markers.
11) The authors analyzed proteins the are phosphorylated in an STK38 dependent manner using MassSpec analysis and identified 25 candidates of which 10 decreased the number of VPS4 dots per cell. They further showed that one of these proteins, DOK1, is recruited to lysosomes upon LLOMe treatment. This study is extremely preliminary and does not add much value to the manuscript. It is not clear why DOK1 is recruited and whether this depends on STK38 kinase activity. Furthermore, DOK1 appears to be insight LAMP1 positive structure, i.e. in the lumen of lysosomes suggesting that it is degraded upon LLOMe treatment in an lysosome dependent manner. The meaning and significance of this observation was not further evaluated.
12) The authors analyzed the contribution of ATG8 proteins to the recruitment of ESCRT proteins to lysosomes after inducing lysosomal damage. They found that cells that recruitment of CHMP4B to lysosomes in hexa-KO cells expressing LC3C, GR-L1 or GR-L2 was restored. Although this recruitment depends on LLOMe treatment, it is not clear whether puncta represent lysosomes and whether this is related to microautophagy. The authors should i) co-stain using lysosomal markers and ii) distinguish macroautophagy from microautophgy. The latter is in general not very well-established by the experiments in this manuscript.
13) The observation of the authors that KD of STK38 and GR induces senescence is interesting, but it remains unclear how this is related to microlysophagy, given that both proteins play also important roles in the regulation of macroautophagy. Overall, the connection between senescence, longevity and microlysophagy appears premature.

Not all references are correctly given, please check the formatting of the bibliography.

Referee #2 Review
Received: 23rd Mar 23

Report for Author:
Recent years led to the discovery of several intracellular mechanisms used to recover lysosomal damage. These include macrolysophagy (a process extensively studied by Dr. Yoshimori and colleagues); ESCRT-dependent repair and the recently reported ESCRT-independent repair governed by ER-lysosome membrane contacts (Tan and Finkel Nature 2022 and Radulovic et al. EMBO J. 2022). The latter was neglected by the authors of the present study.
In the present study, Ogura et al. identified STK38, a serine/threonine kinase implicated in multiple cellular pathways, including cell cycle regulation and apoptosis, as a critical factor regulating ESCRT-dependent repair of lysosomal damage. The authors

found that STK38 is incorporated into damaged lysosomes in a T444 phosphorylation and Ca+2-dependent manner, where it recruits VPS4, a key regulator of ESCRT function. The authors also provide evidence for the involvement of GABARAP proteins in assembling the ESCRT machinery on the damaged lysosomal membranes by interaction with the early-acting ESCRT component, ALIX. Additionally, the knockdown of STK38 and GABARAPs increases cellular sensitivity to senescence. The C. elegans orthologues of these factors were also tested for their involvement in aging.

The authors provide solid data indicating the recruitment of STK38 to the damaged lysosome. Lysosomal membrane damage, determined by the incorporation of Galectin 3 was shown to be affected by a lack of STK38. However, the same assay for Galectin 3 is missing in the GABARAP knockout background. Additionally, Galectin 3 localization should be determined in the background of the different STK38 mutants, including the phosphorylation mutants and the C' and N' truncated STK38 constructs.

In general, many aspects of this manuscript should be better characterized. For example, while the authors claim that VPS4 is recruited to lysosomal membranes by STK38, it remains unclear whether STK38 directly associates with VPS4 or indirectly recruits it through DOK1 upon phosphorylation by STK38. The exact role of DOK1 in this process was also not established. The authors should explain why they focused on STK38, while the original data obtained for MOB2 were more convincing. Finally, no physiological conclusions may be drawn according to LC3s role in senescence assay results since the knockdown of LC3s didn't work (Fig. EV7).

Additional comments

1. Figure 1 A-B - the comparison between WT siSTK38 and FIP200 KO siSTK38 is missing. The accumulation of GFP-Gal3 in FIP200 KO siSTK38 is minor.
2. Figure 2 C-D - that data present a negative result that does not contribute to the figure - should be moved to supplementary information.
3. Figure 3 D and F - the cleavage assay of GFP-TRPM1 is not convincing. The amount of free GFP is similar and hardly detected even in the positive control.
4. Figure 3 H - the TEM images should be supported by CLEM or by using appropriate markers in IF.
5. Figure 4A-B - the data obtained by these experiments are inconclusive, possibly because the phospho-mimicking mutant was expressed on the background of endogenous STK38.
6. Figure 5K -quantification and statistical information are needed.
7. Figure 5L - the IP results are unclear. It appears as LLOMe treatment reduces ALIX association with mCherry-GABARAPL2. In addition, the blot lacks the IP with mCherry alone as a control. The reciprocal IP may support the authors' claims.
8. Figure 6 A and C - the western blots should include siLC3s and siGABARAPs. The decrease in SA-β-Gal positive cells or reduction of senescence markers upon recovery of STK38 and GABARAPs may support the suggested model.
9. Figure 6K-L - the probability of survival upon depletion of the different orthologs should be tested under lysosomal stress and not only under basal conditions.
10. Supplementary figure EV3 D-E - the effect of STK38 is not convincing. The cell in the lower left corner looks like the control cells.

1st Editorial Decision 6th Apr 2023

Dear Shuhei,

Thank you for the transfer of your research manuscript to our journal.

As discussed, I would like to invite you to revise your manuscript along the lines outlined in your revision plan. From our side, the most important point to address is that you strengthen the proposed role of STK38 in lysosomal repair and that you test the relative contribution of micro- versus macroautophagy - also by further validating the EGFP-TRPML1 cleavage assay, as outlined. If the data on senescence remain correlative, this is fine from our side, given that all conclusions are carefully phrased.

Please address all referee concerns in a complete point-by-point response. Acceptance of the manuscript will depend on a positive outcome of a second round of review. It is EMBO reports policy to allow a single round of revision only and acceptance or rejection of the manuscript will therefore depend on the completeness of your responses included in the next, final version of the manuscript.

We realize that it is difficult to revise to a specific deadline. In the interest of protecting the conceptual advance provided by the work, we recommend a revision within 3 months (July 6th). Please discuss the revision progress ahead of this time with me if you require more time to complete the revisions.

I am also happy to discuss the revision further via a video call at any point, if you wish.

***** IMPORTANT GENERAL NOTE:
We perform an initial quality control of all revised manuscripts before re-review. Your manuscript will FAIL this control and the handling will be DELAYED if the following APPLIES:

1) A data availability section providing access to data deposited in public databases is missing. If you have not deposited any data, please add a sentence to the data availability section that explains that.

2) Your manuscript contains statistics and error bars based on n=2. Please use scatter blots in these cases. No statistics should be calculated if n=2.

When submitting your revised manuscript, please carefully review the instructions that follow below. Failure to include requested items will delay the evaluation of your revision. *****

When submitting your revised manuscript, we will require:

1) a .docx formatted version of the manuscript text (including legends for main figures, EV figures and tables). Please make sure that the changes are highlighted to be clearly visible.

2) individual production quality figure files as .eps, .tif, .jpg (one file per figure).
Please download our Figure Preparation Guidelines (figure preparation pdf) from our Author Guidelines pages https://www.embopress.org/page/journal/14693178/authorguide for more info on how to prepare your figures.

3) a .docx formatted letter INCLUDING the reviewers' reports and your detailed point-by-point responses to their comments. As part of the EMBO Press transparent editorial process, the point-by-point response is part of the Review Process File (RPF), which will be published alongside your paper.

4) a complete author checklist, which you can download from our author guidelines (<https://www.embopress.org/page/journal/14693178/authorguide>). Please insert information in the checklist that is also reflected in the manuscript. The completed author checklist will also be part of the RPF.

5) Please note that all corresponding authors are required to supply an ORCID ID for their name upon submission of a revised manuscript (<https://orcid.org/>). Please find instructions on how to link your ORCID ID to your account in our manuscript tracking system in our Author guidelines
(<https://www.embopress.org/page/journal/14693178/authorguide#authorshipguidelines>)

6) We replaced Supplementary Information with Expanded View (EV) Figures and Tables that are collapsible/expandable online. A maximum of 5 EV Figures can be typeset. EV Figures should be cited as 'Figure EV1, Figure EV2" etc... in the text and their respective legends should be included in the main text after the legends of regular figures.

- For the figures that you do NOT wish to display as Expanded View figures, they should be bundled together with their legends in a single PDF file called *Appendix*, which should start with a short Table of Content. Appendix figures should be referred to in the main text as: "Appendix Figure S1, Appendix Figure S2" etc. See detailed instructions regarding expanded view here: <https://www.embopress.org/page/journal/14693178/authorguide#expandedview>

- Additional Tables/Datasets should be labeled and referred to as Table EV1, Dataset EV1, etc. Legends have to be provided in a separate tab in case of .xls files. Alternatively, the legend can be supplied as a separate text file (README) and zipped together with the Table/Dataset file.

7) Please note that a Data Availability section at the end of Materials and Methods is now mandatory. In case you have no data that requires deposition in a public database, please state so instead of refereeing to the database.
See also < https://www.embopress.org/page/journal/14693178/authorguide#dataavailability>). Please note that the Data Availability Section is restricted to new primary data that are part of this study.

8) At EMBO Press we ask authors to provide source data for the main figures. Our source data coordinator will contact you to discuss which figure panels we would need source data for and will also provide you with helpful tips on how to upload and organize the files.

Additional information on source data and instruction on how to label the files are available <https://www.embopress.org/page/journal/14693178/authorguide#sourcedata>.

9) The journal requires a statement specifying whether or not authors have competing interests (defined as all potential or actual interests that could be perceived to influence the presentation or interpretation of an article). In case of competing interests, this must be specified in your disclosure statement. Further information: https://www.embopress.org/competing-interests

10) Figure legends and data quantification:
The following points must be specified in each figure legend:

- the name of the statistical test used to generate error bars and P values,
- the number (n) of independent experiments (please specify technical or biological replicates) underlying each data point,
- the nature of the bars and error bars (s.d., s.e.m.)

- If the data are obtained from n {less than or equal to} 5, show the individual data points in addition to the SD or SEM.
- If the data are obtained from n {less than or equal to} 2, use scatter blots showing the individual data points.

Discussion of statistical methodology can be reported in the materials and methods section, but figure legends should contain a basic description of n, P and the test applied.

See also the guidelines for figure legend preparation:
https://www.embopress.org/page/journal/14693178/authorguide#figureformat

- Please also include scale bars in all microscopy images.

11) Our journal encourages inclusion of *data citations in the reference list* to directly cite datasets that were re-used and obtained from public databases. Data citations in the article text are distinct from normal bibliographical citations and should directly link to the database records from which the data can be accessed. In the main text, data citations are formatted as follows: "Data ref: Smith et al, 2001" or "Data ref: NCBI Sequence Read Archive PRJNA342805, 2017". In the Reference list, data citations must be labeled with "[DATASET]". A data reference must provide the database name, accession number/identifiers and a resolvable link to the landing page from which the data can be accessed at the end of the reference. Further instructions are available at <https://www.embopress.org/page/journal/14693178/authorguide#referencesformat>.

12) As part of the EMBO publication's Transparent Editorial Process, EMBO reports publishes online a Review Process File to accompany accepted manuscripts. This File will be published in conjunction with your paper and will include the referee reports, your point-by-point response and all pertinent correspondence relating to the manuscript.

You are able to opt out of this by letting the editorial office know (emboreports@embo.org). If you do opt out, the Review Process File link will point to the following statement: "No Review Process File is available with this article, as the authors have chosen not to make the review process public in this case."

We would also welcome the submission of cover suggestions, or motifs to be used by our Graphics Illustrator in designing a cover.

I look forward to seeing a revised form of your manuscript when it is ready and please let me know if you have questions or

comments regarding the revision.

Kind regards,

Martina

Martina Rembold, PhD
Senior Editor
EMBO reports

Referee #1:

(Our responses are shown in blue)

The authors of this study investigated the role of STK38 in regulating lysosomal repair by microautophagy after damage. They found that STK38 is recruited to lysosomes after lysosomal damage induced by LLOMe, that the C-terminal domain of STK38 is important for recruitment to lysosomes while the N-terminus is dispensable and that KD of STK38 leads to a sustained and enhanced formation of ESCRT puncta and a concomitant reduction of VPS4 puncta in an LLOMe dependent manner. They found that the recruitment of STK38 to LAMP1 positive structures was independent of canonical autophagy proteins including FIP200, ATG14, ATG9, ATG7 and STX17 arguing that STK38 regulates lysosome repair by microautophagy. They also identified substrates of STK38 that are phosphorylated upon lysosomal damage, but the relationship of the identified proteins to lysosomal repair was not further studied. They also correlated STK38 function in lysosomal repair with senescence and longevity, but both aspects remain mechanistically unconnected and premature. The major claim of the authors that STK38 is essential for lysosomal repair by recruiting VPS4 to induce recycling of ESCRT-III components is not supported by the experimental data for reasons that are mentioned in the specific points listed below.

Given the strong limitations of the study and the significant concerns, the manuscript is not suited for publication in the EMBO journal.

First of all, we thank the reviewer for investing tremendous efforts and time to review our paper. We sincerely appreciate the reviewer's constructive and valuable comments. All of the comments raised are quite helpful to improve our manuscripts. The specific point-by-point responses are detailed below.

1) There is only a small fraction of LAMP1 positive structures colocalizing with STK38 upon induction of lysosome damage. Moreover, knockdown of STK38 has only a mild impact on lysosomal clearance compared to MOB2 or LATS2. The authors should quantify the knockdown efficiency for the different members of the HIPO pathway shown in EV1 A. This is particularly important since KD of LATS2 has an even stronger effect than KD of STK38. Moreover, both, STK38 and LATS2 are regulated by upstream kinases, which might act in a redundant manner so that KD of only one can be compensated by other. The authors should perform co-KD of MST1/2/3 or combinations of two.

Colocalization between STK38 and LAMP1

We have two reasons for the low co-localization rate between STK38 and LAMP1 after 1 h of LLOMe treatment: i) analytical issue and ii) experimental limitation.

i) In the previous analysis, we set the strict threshold to remove the signal of mNG-STK38 at nucleus. However, it was too strict to detect all mNG-STK38 puncta colocalized with LAMP1. Therefore, we have revised the analytical setting and re-analyzed the colocalization between STK38 and LAMP1. As a result, the colocalization rate between LAMP1 and mNG-STK38 after 1h LLOMe treatment was around 25%, which was not so much different from the rate between LAMP1 and VPS4 (around 30%, shown in later) (**Fig 1B and C**).

ii) Although, STK38 was much more recruited on LAMP1 positive lysosomes when we examined at the later time point as indicated in Fig 1A, we quantified the colocalization rate soon after the treatment of LLOMe for 1h in Fig 1B and C. This is because this time window was the best condition so far we tested to combine with BAPTA-AM treatment.

In the revised manuscript, we have replaced Fig 1B and C with the re-analyzed result explained above.

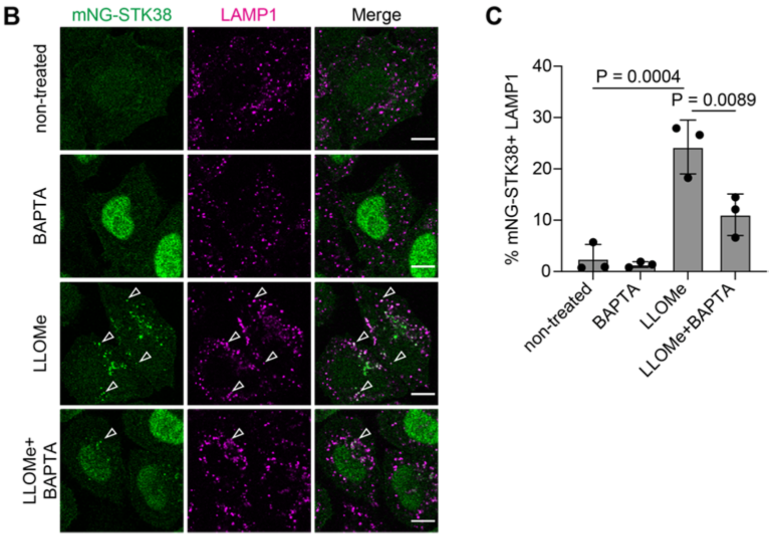

Fig 1B and C

Knockdown efficiency of Hippo pathway siRNAs

We have checked the knockdown efficiency of siRNAs for Hippo pathway members by qPCR or WB (for siSTK38). These results are added in the revised manuscript (**Appendix Fig S1**).

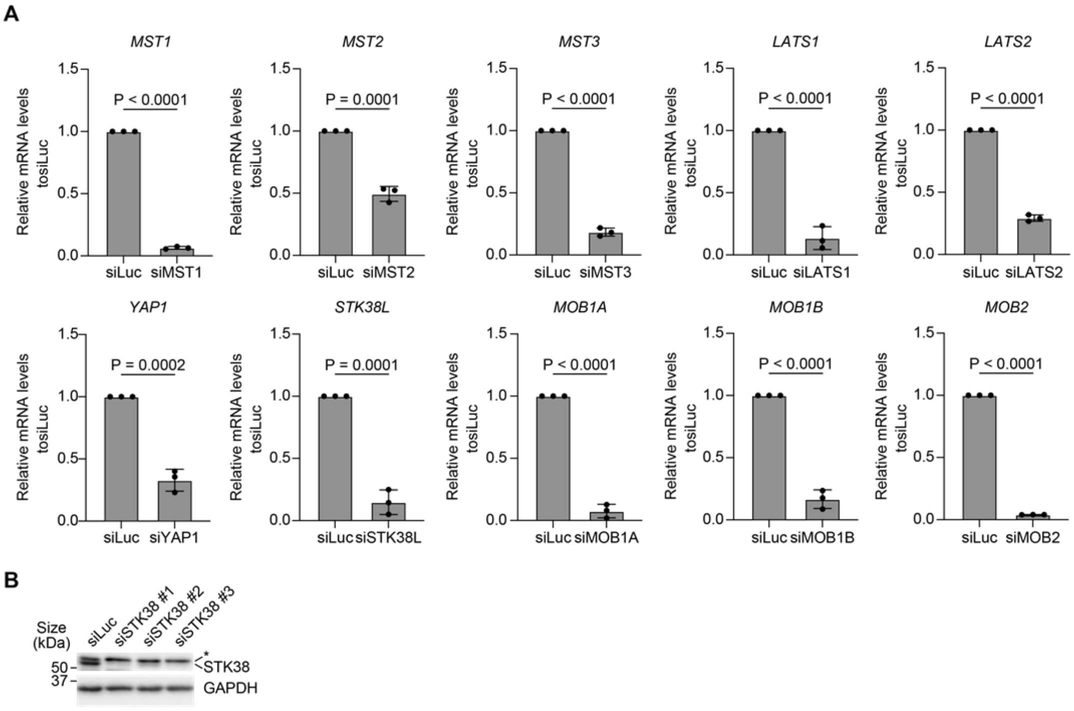

Appendix Fig S1

Effects of Hippo pathway factors on lysosomal damage clearance

We deeply appreciate the reviewer's suggestions. We have performed GFP-Gal3 dot count screening including LATS2 and MOB2 KD sample using siRNA with confirmed high knockdown efficiency (see **Appendix Fig S1** for the knockdown efficiency of these siRNAs) and MST1/2/3 triple-KD samples. For the multiple comparison, we did this screening again for three times.

Similar to our previous screening, STK38 and MOB2 KD consistently and significantly increased the remaining Gal3 dots, suggesting that both components are required for clearance of damaged lysosomes (**Fig EV1A and B**). However, in contrary to our previously screening, LATS2 KD did not show significant increase of Gal3 dots, while MST2 KD did. Although the reason of slightly different results from our previous screening is currently unclear, our data consistently indicates that STK38 and MOB2 have essential roles for the clearance of damaged lysosomes. In line with this, the previous work suggests that MOB2 binds to STK38 and act as a cofactor of STK38 (Bothos et al, 2005; Devroe et al, 2004). And as the reviewer pointed out, MST2 might work upstream of these complex, although why triple KD of MST1/2/3 did not show significant increase of Gal3.

Based on these results, in the current study, we mainly pursued roles of STK38, since it is implicated to play a role in autophagy and locates in the center of MST2-STK38-MOB2 cascade. We mentioned these results, possibility and explanation in the result and discussion part (page 4, line 122 and page 12, line 433). Due to weak phenotype of LATS2, we have removed the localization data in the revised manuscript. We hope that our explanation and revision are reasonable for the reviewer.

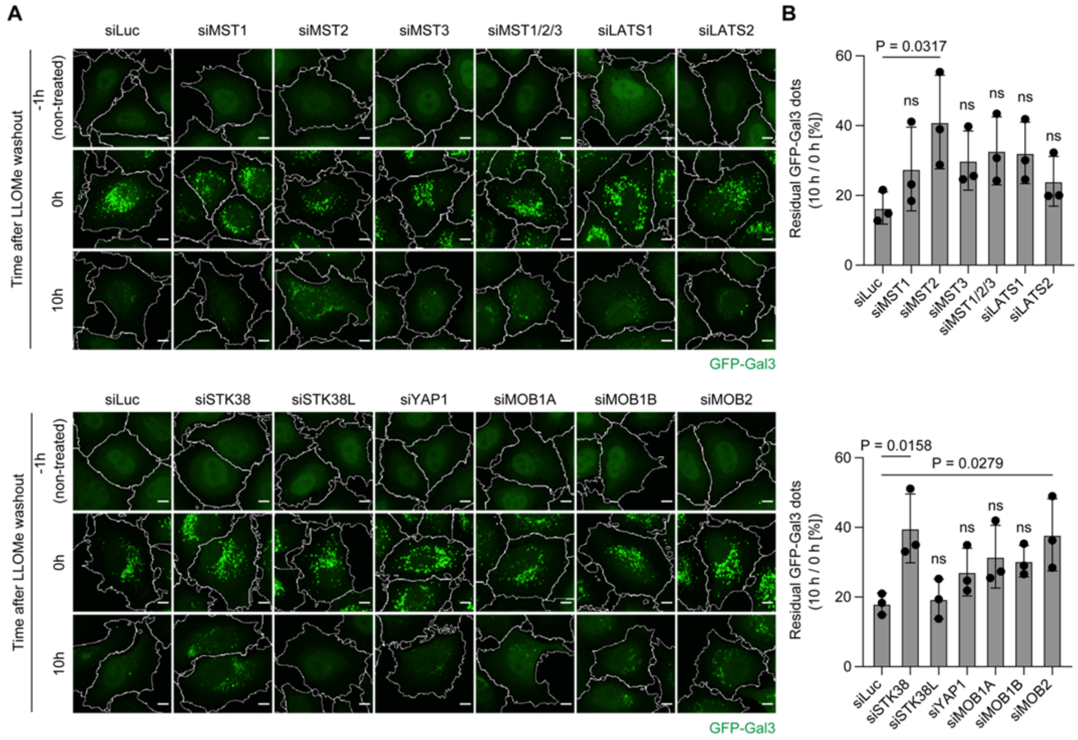

Fig EV1A and B

2) The LLOMe induced phosphorylation of T444 (Fig. EV1E) is not very convincing. First, there is no obvious change in the phosphorylation status of STK38 based on western blots. The authors need to verify i) the identity of the bands (in the reference blots from the vendor there is only a single band visible for pSTK38, the double band is not visible), and quantify the phosphorylated STK38 fraction (normalized to total STK38 expressed and corrected by loading control). Second, the T444A mutant still shows robust colocalization with LAMP1 while the effect of deleting the C-terminal part of STK38 has a much stronger effect.

Phosphorylation of STK38 T444 after LLOMe treatment

i) Identity of the bands

The pSTK38 antibody (Signalway Antibody, #12521) we used in this experiment detects both STK38 (pT444) and STK38L (pT442). By two independent siRNA against STK38 (#2 and #3) and STK38L (#1 and #2), we verified the upper band and the lower band corresponding to STK38L and STK38, respectively (Additional Fig 1). Since STK38 and STK38L are very close in size, we speculate the band pattern may seem to be a single or double band depending on the condition of SDS-PAGE and/or cell type. We have indicated STK38 and STK38L in blots shown in **Fig EV1E** in the revised manuscript (STK38L is indicated as an asterisk). Please also see our answer to question 6), explaining how to determine the actual STK38 band in detail.

Figure for referees not shown.

Additional Fig 1

ii) Quantification of phosphorylated STK38

As the reviewer #1 suggested, we have quantified the phosphorylation of STK38 T444 after LLOMe treatment. As shown below, phosphorylation of STK38 T444 was significantly increased after LLOMe treatment. We have added the blots and graph as **Fig EV1E and F** in the revised manuscript.

E

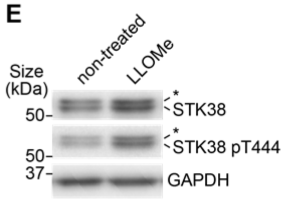

Fig EV1E and F

Localization of STK38 T444A mutant

To clarify the effect of T444 phosphorylation on lysosomal localization of STK38, we have quantified co-localization between STK38 mutants and LAMP1. As the reviewer #1 pointed out, STK38 T444A mutant showed less lysosomal localization than WT, but it was not significant, while deletion of C-terminal of STK38 (deltaC mutant) completely abolished its lysosomal localization (**Fig 1E and F**). Therefore, we have omitted the following sentence “a phosphor-dead mutation (T444A) reduced the lysosomal localization of STK38”, and replaced with a weaker expression in the revised manuscript.

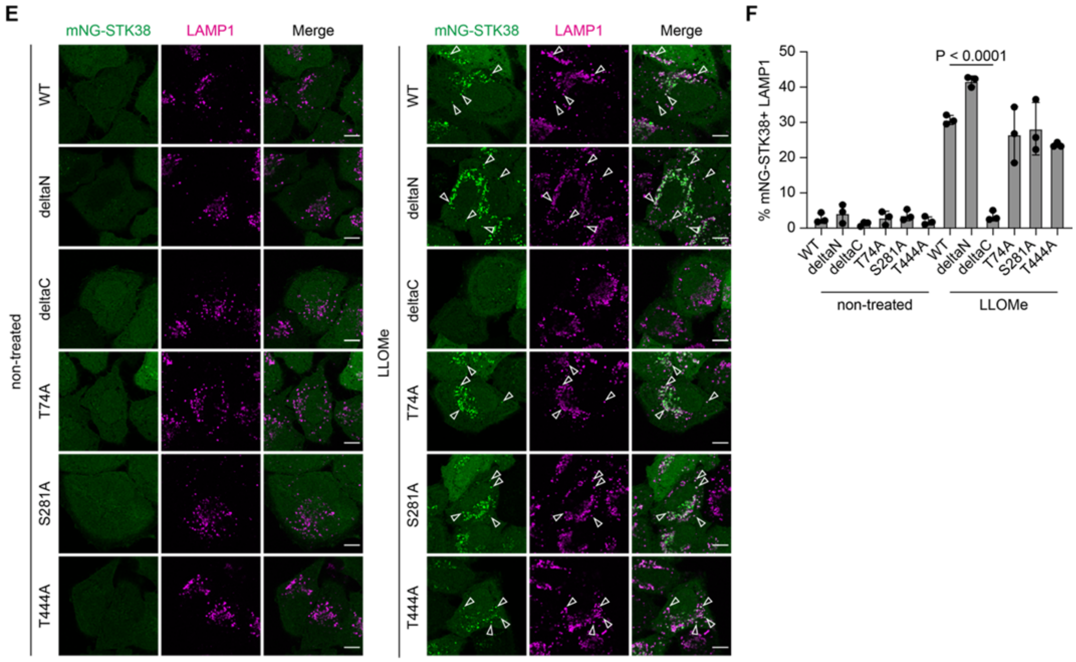

Fig 1E and F

3) The detection of damaged lysosomes by colocalization with LAMP1 after LLOMe treatment is too unspecific. The authors should include co-localization studies of STK38 in wt and siRNA treated cells with Gal3 (Fig 1E, Fig EV 1C, Fig EV 2A).

We have included images of mNG-STK38 with Gal3 in **Fig EV1D**. However, it is important to note that Gal3 positive lysosomes do not reflect all damaged lysosomes. Gal3 only labels the ruptured lysosomes with severe damage but not the ones with small damage. A previous report (Lee et al., Dev Cell, 2020) together with our current work suggests that ESCRTs dependent microlysophagy could target the Gal3 negative lysosomes with small damage in addition to Gal3 positive lysosomes with severe damage. Indeed, we confirmed that the treatment of lysosomal stress inducers such as nigericin, monensin, and NH_4Cl did not cause Gal3 positive severe lysosomal damage (shown later in the answer to question 9, **Fig EV3C**), but induced microlysophagy. Given that there are no appropriate markers to label lysosomes with small damage, we still think co-localization analysis using general lysosomal marker LAMP1 is valuable. Therefore, we have also included and maintained the co-localization studies between mNG-STK38 (WT and mutants) and LAMP1 in WT cells in **Fig 1E and F** (shown above). For the same reason, we also maintained the colocalization data for MOB2 (Fig. EV1C) and WT STK38 in several macroautophagy deficient cells (now in Appendix Fig S2), respectively in the revised manuscript.

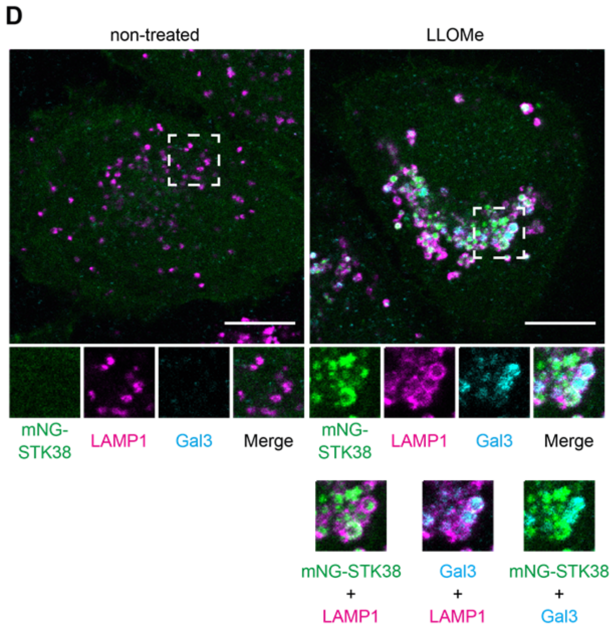

Fig EV1D

4) Knockdown of FIP200, ATG14, ATG7 and STX17 appears to induce an even more pronounced colocalization of STK38 with LAMP1, indicating that disruption of macroautophagy leads to an accumulation of damaged lysosomes to which STK38 is recruited. To determine, which of the two pathways is predominant, the authors should investigate colocalization of FIP200 with lysosomes in STK38 KO/KD (for STK38 KD, the efficiency of siRNA treatment need to be verified, see point 1).

Thank you so much for this suggestion. Unfortunately, we have no good antibodies to immunostain FIP200. Therefore, we have checked the lysosomal localization of ULK1 which forms functional complex with FIP200 and is required for formation of autophagosomes. Our results showed that mNG tagged ULK1 was co-localized with LAMP1 after LLOMe treatment and the co-localization rate in STK38 KD cells were comparable to control cells (**Fig EV 2C and D**). Although it is not enough to make conclusion which of the two pathways is predominant, this result at least suggests that macroautophagy is not affected by the suppression of microautophagy. We have added these results as **Fig EV2C and D** in the revised manuscript.

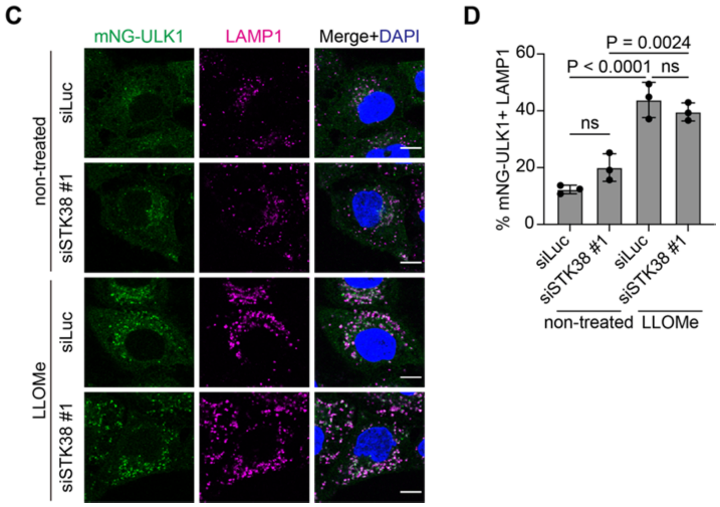

Fig EV2C and D

5) The authors argue that KD of STK38 has no impact on starvation induced autophagy, but in nonstarved cells, much less LC3B-II is detected in BafA1 treated cells. What is the reason for this strong decline (Fig EV 2C).

We guess the reviewer pointed out LC3B-II in Fig EV2D in the initial manuscript. Although LC3B-II intensity seems slightly decreased, the quantification and statistical analysis from 3 independent biological replicates did not show significant difference between siLuc plus BafA1 and siSTK38 plus BafA1 (Additional Fig 2). We have added statistical analysis (ns) to the quantification data (**Fig EV2B** in the revised manuscript).

Figure for referees not shown.

Additional Fig 2

6) The authors demonstrated KD of STK38 by westernblot (Fig. EV2C) referring to the upper band of the two bands detected and cutting the lower band, which was even in the KD very strong. In Fig. EV1E, they referred to the lower band for STK38, indicating that the band that has been cut in Fig. EV2C corresponds to STK38. Moreover, the reference blots of the vendor did not show a double band. The authors need to verify the identity of the bands to reveal why they detect two bands and which one corresponds to STK38. This is very important because most data of the paper are based on comparing STK38 expressing and KD cells.

This is related to the question in 2). First, we deeply apologize to the reviewer for raising many questions and confusion due to our insufficient information. Actually, we used different cell types in Fig EV1E (HeLa cells) and EV2D (U2OS). This is one of the reasons why the blots of STK38 look different.

We realized that the reason why we detect several bands is due to the combinatory effects of different cell types (HeLa, MCF10A, U2OS or RPE1 cells) and different percentages of SDS PAGE gel (8% or 10%) used in our experiment (please see also below). Nevertheless, we have confidently identified actual STK38 band by using several siRNA against STK38 and STK38L in different cell types. In the following section, we show the detailed evidence how to determine the STK38 band.

Identification of bands detected by immunoblotting for STK38

In WB with anti-STK38 antibody (Santacruz cat# sc-100404) in HeLa cells, three bands were detected around 50-54 kDa (Additional Fig 3A, uppermost band indicated in green: band1, middle band indicated in magenta: band2, and bottom band indicated in cyan: band3). Of these, only band 2 (siSTK38 #1) or band 2 and 3 (siSTK38 #2, #3) were disappeared in siSTK38 transfected cells, suggesting that these bottom two bands (band 2 and 3) are corresponding to STK38 in HeLa cells. In contrast, band 1 was suggested to be STK38L which have an amino acid sequence 87% homologous to STK38, since it disappeared in STK38L knockdown cells, (Additional Fig 3, green).

Figure for referees not shown.

Additional Fig 3

To further identify band 2 and 3 which correspond to STK38, we checked the sequence of each siRNA against STK38 and found that siRNA #1 targets the 3' UTR, while siRNAs #2 and #3 target the CDS of STK38 (Additional Fig 4). Based on NCBI database, there is an isoform mRNA of which does not contain the 3'UTR targeted by siRNA #1 (NCBI Reference Sequence: XP_016865715.1). These data suggested that this isoform corresponds to band 3, and band 2 corresponds to the canonical STK38.

Figure for referees not shown.

Additional Fig 4

Identification of STK38 bands in various cell lines used in this study

Results of immunoblotting of STK38 in siSTK38 transfected cell lines used in the paper are shown in Additional Fig 5. Bands of STK38 and STK38L are located very close to each other, which makes it very difficult to separate them depending on conditions of WB such as the concentration of acrylamide in the gel and the time of electrophoresis. In HeLa, MCF10A, and RPE1 cells, three bands were detected with anti-STK38 antibody, and the middle band (magenta, band 2) was disappeared in every siSTK38 transfected cells (Additional Fig 5A-C). Besides this, in U2OS cells,

only two bands were detected and the uppermost band (corresponding to band1, STK38L in HeLa cells) is almost invisible, probably due to the low expression level of STK38L in this cell line (Additional Fig 5D). Importantly, in U2OS, band 2 was clearly decreased by knockdown of STK38, suggesting that this band corresponds to STK38.

Figure for referees not shown.

Additional Fig 5

Identification and validation of the phosphorylated STK38 band

As we explained in the above answer to question 2), for WB of phosphorylated STK38, we used antibodies capable of detecting both pSTK38 and pSTK38L. Among the two detected bands, the upper one is STK38L and the lower one is STK38, as confirmed by knockdown experiment (Additional Fig 6).

Figure for referees not shown.

Additional Fig 6

In conclusion, among three or two bands detected by STK38 antibody, **the middle one (in HeLa,**

MCF10A and RPE1 cell) or **the upper one (in U2OS cell)** were referred as **STK38** in our manuscript. In the revised manuscript, we have provided the blots of STK38 cropped the lowest band for the better visualization. In addition, we have indicated which siRNA for STK38 was used in every experiment.

7) The reduction of VPS4 puncta in the STK38 KO is much less significant than in the KD. The same is true for ALIX puncta. If STK38 is required to recruit VPS4, one would expect to see a much stronger effect. The authors should better characterize what type of puncta they count by costaining of VPS4 and Gal3 in WT and STK KD and KO cells.

Why the phenotype of KO is weaker than KD is currently unclear. One possibility is that STK38L might compensate for function in STK38 KO cells. Indeed, this type of compensation is reported in previous studies (Rossi et al, 2015; El-Brolosy, 2017).

We have also characterized the type of puncta by co-staining of VPS4 and LAMP1 in STK38 KD cells (we used LAMP1 instead of Gal3 since Gal3 is not suited to detect lysosomes repaired by ESCRTs as explained in point 3). As shown below, co-localization between VPS4 and LAMP1 after LLOMe treatment was significantly suppressed in STK38 KD cells (**Fig 2F and G** in the revised manuscript). Furthermore, we have confirmed that reconstitution of STK38 in STK38 KD cells rescued VPS4 recruitment to lysosomes, suggesting that suppression of VPS4 recruitment is indeed caused by STK38 depletion (**Fig 4A-C** in the revised manuscript). In STK38 KO cells, we could not see the significant suppression of this co-localization due to its weak phenotype on ESCRT recruitment by possible compensation, same reason explained above (now in **Appendix Fig S3E and F**). We have mentioned this weak phenotype of KO cells and its possible reason in the revised manuscript (page 6, line 195).

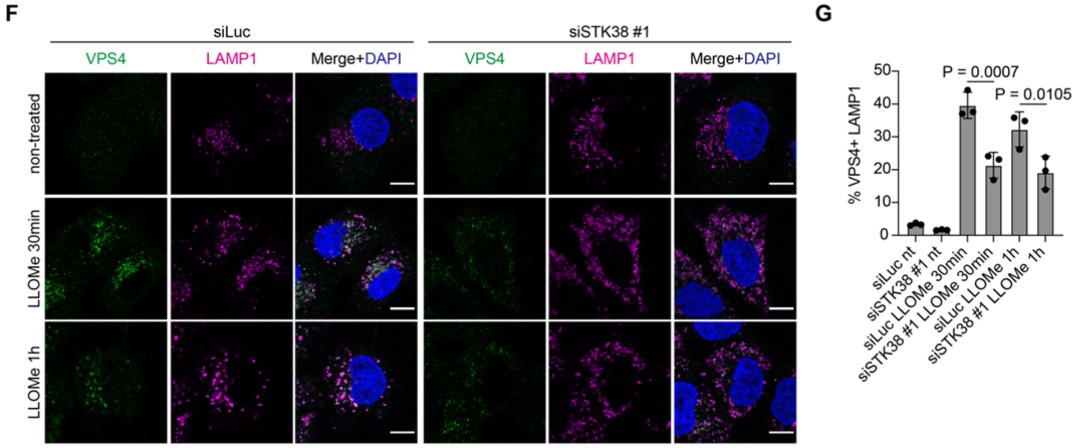

Fig 2F and G

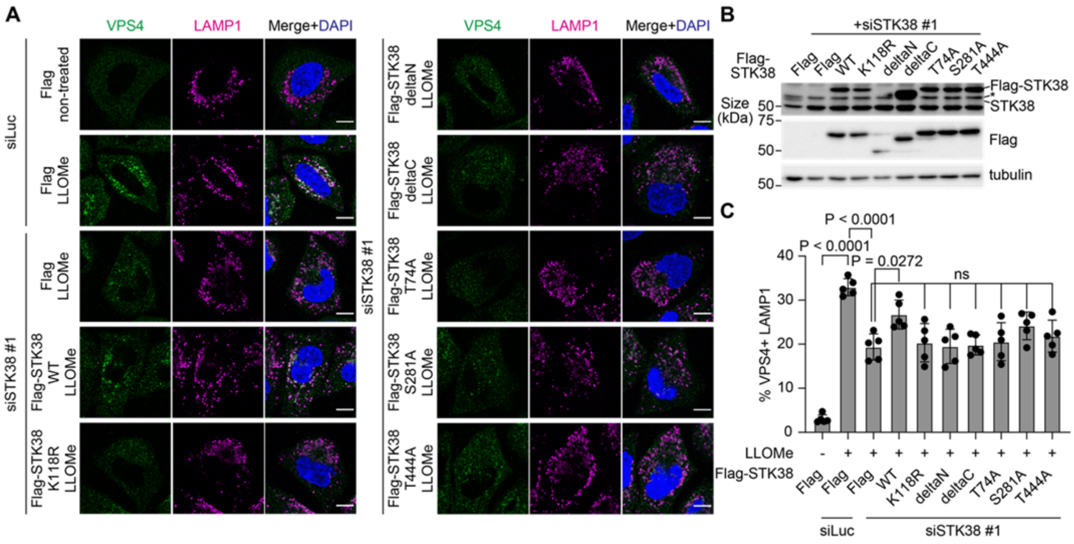

Fig 4A-C

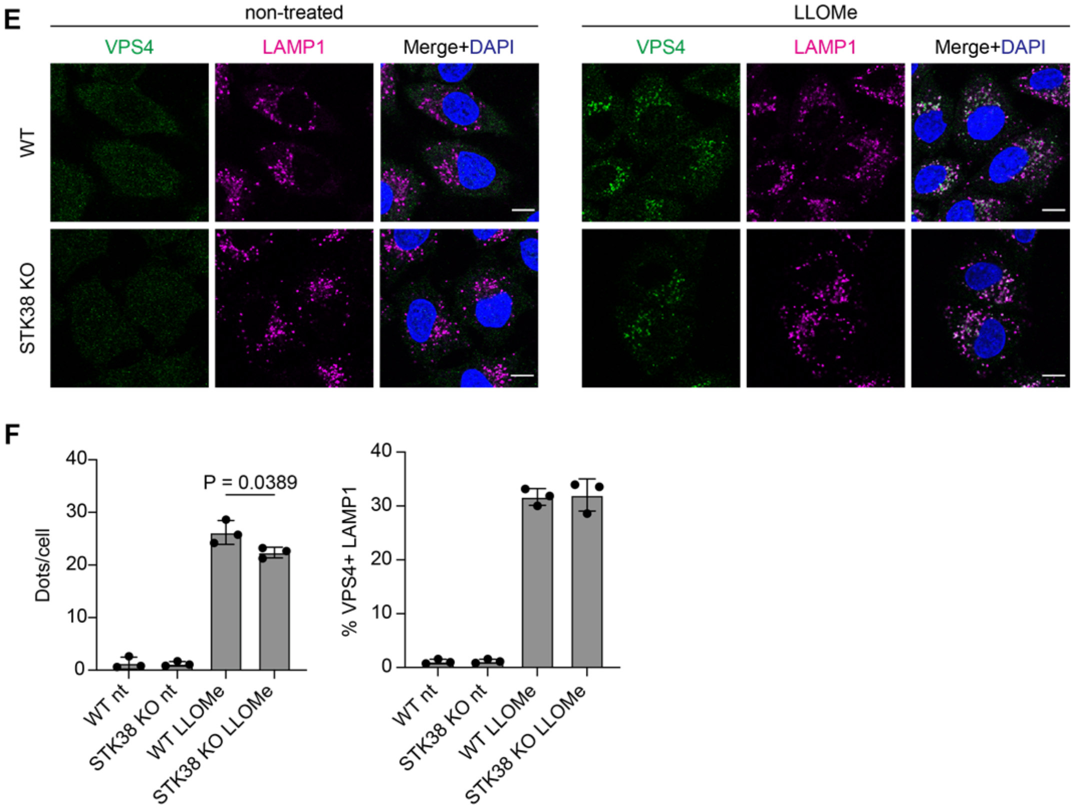

Appendix Fig S3E and F

8) The authors used the GFP fused lysosomal channel TRPML1 as a marker for micolysophagy. However, TRPML1 will also be degraded by macrolysophagy. It is not clear why the GFP-TRPML1 degradation assay was chosen to distinguish between both options.

In general, microautophagy in mammals is poorly understood and there is no established way to detect its activity. Under these circumstances, EGFP-TRPML1 has been reported as the novel probe to monitor microautophagy, especially under lysosomal stresses/damage conditions (Lee et al, 2020). In this previous study, authors showed that EGFP-TRPML1 was cleaved by microautophagy-dependent and macroautophagy-independent manner under lysosomal stress (nigericin treatment). We confirmed this specificity by ourselves. Importantly, the cleavage of EGFP-TRPML1 was observed in macroautophagy deficient ATG13 KO cells similar to WT under LLOMe in addition to nigericin treatment, but was impaired in both micro-and macroautophagy deficient ATG5 KO cells as shown in **Fig EV3A and B** in the revised manuscript, suggesting that the cleavage of EGFP-TRPML1 is indeed mediated by microautophagy and thus suitable assay to monitor microautophagy. Moreover, depletion of STK38 suppressed the cleavage of EGFP-TRPML1 both in WT and ATG13 KO cells, suggesting that STK38 regulates microlysophagy in macrolysophagy independent manner (**Fig 3D and E** in the revised manuscript). Why the cleavage of lysosomal EGFP-TRPML1 is not altered in macroautophagy deficient cells is currently unclear, but it could be due to the sensitivity of this probe.

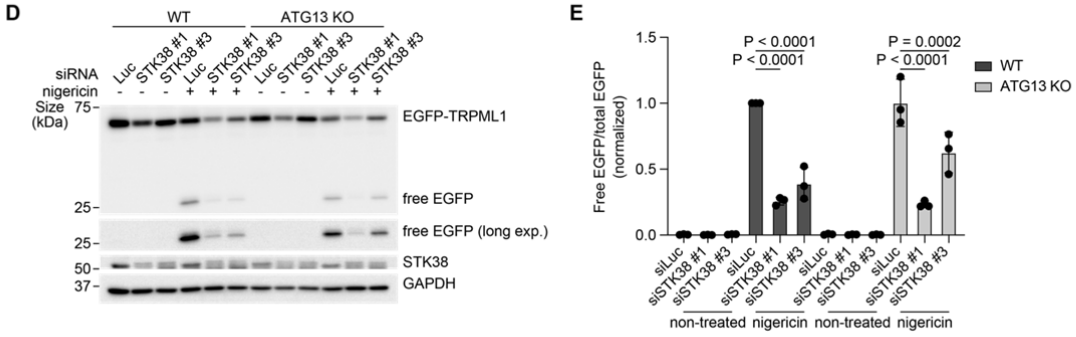

Fig 3D and E

9) Why did the authors used LLOMe in some experiments (Fig. 1, 2 and EV 1-5) but nigericin in this experiment? Different ways to induce lysosomal damage or stress could result in the activation of different cellular response pathways. This is even more evident as using LLOMe in EGFP-TRPML1 degradation assays has almost no impact on the generation of free EGFP. Only a minor fraction of EGFP-TRPML1 is degraded (free EGFP band is extremely weak in control experiments) and a further reduction of the intensity in STK38 KD (Fig. 3D-G) is not very convincing.

We apologize for the lack of a detailed explanation. Based on the previous finding (Lee et al, 2020), it is suggested that the lysosomal ‘stress’ inducers (e.g. Nigericin/Monensin/NH_4Cl) only induces microautophagy, while the lysosomal ‘damage’ inducer (LLOMe) presumably induces both micro-

and macroautophagy. Indeed, we have confirmed that these lysosomal stress inducers did not induce the recruitment of Gal3, p62, and ubiquitin to lysosomes which are known to be accompanied with macrolysophagy (**Fig EV3C** in the revised manuscript). As the reviewer mentioned, we also feel that the level of EGFP-TRPML1 cleavage was slightly low upon LLOMe treatment compared to lysosomal stress inducers for unknown reasons. Based on these situations, we utilized lysosomal stress inducers in some experiments especially when we want to evaluate microautophagy-specific activity. In the revised manuscript, we have included these explanations. In addition, regarding the free GFP band, we have added the blots of long-exposure time together to show the difference in a convincing manner.

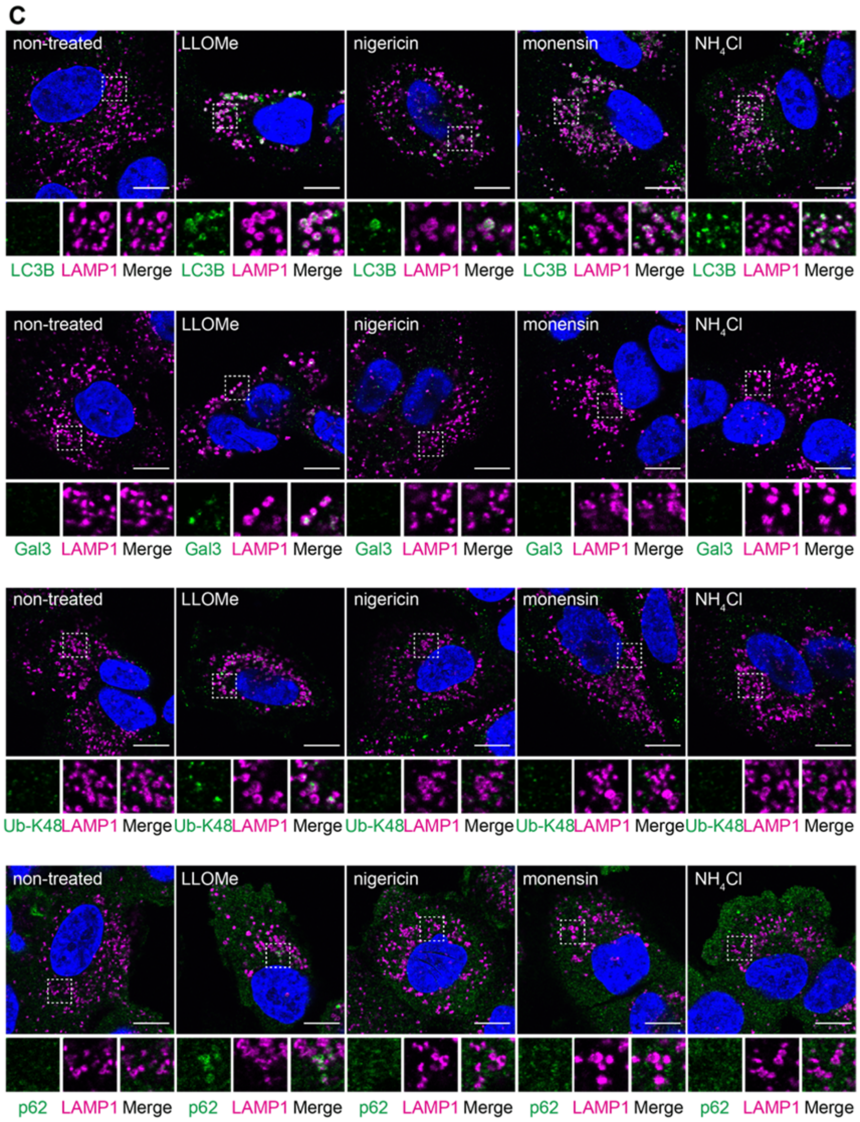

Fig EV3C

10) Based on EM images shown in Fig 3H, the authors argue that treatment of cells with NH4Cl

induces microautophagy resulting in the formation of intraluminal vesicles in lysosomes. However, the structures in the siLuc NH4Cl sample look like MVBs and not like lysosomes, while those in non-treated conditions contain not only ILVs, but also other material, what would be expected. To clarify this, the authors need to perform CLEM experiments using lyosomal markers and compare this with CLEM using MVB markers.

We have tried to identify ILVs containing compartment using lysosomal and MVB markers by confocal microscopy and CLEM. To this aim, we used CD63 and LAMP1 as markers of MVBs or lysosomes, respectively. First, we have performed IF-based observation of EGFP-TRPML1 positive ILVs in pharmacologically swollen LAMP1 or CD63 positive compartments. As shown in **Fig EV4D and E** in the revised manuscript, EGFP-TRPML1 positive ILVs were observed in either LAMP1 or CD63 positive endolysosomal compartment after monensin treatment. We also have performed CLEM using LAMP1-GFP expressing cells and observed ILVs in LAMP1-GFP positive endolysosomes (**Fig 3J** in the revised manuscript). These results suggest that microlysophagy could occur LAMP1 and/or CD63 positive endolysosomes. Of note, even these two membrane proteins, LAMP1 and CD63, are enriched on MVBs or lysosomes, respectively and often used as markers, it is difficult to dissect these two compartments clearly by them, since their localization is not specific on single compartment and overlapped between lysosomes and endosomes. In conclusion, our results suggest that EGFP-TRPML1 positive ILVs are formed at least LAMP1 positive lysosomal compartment. Importantly, we also showed that STK38 function is dispensable for EGFR degradation which are known to be mediated by MVB pathway, suggesting that STK38 mediated microlysophagy is distinct from canonical MVB pathway (**Fig EV2G and H**).

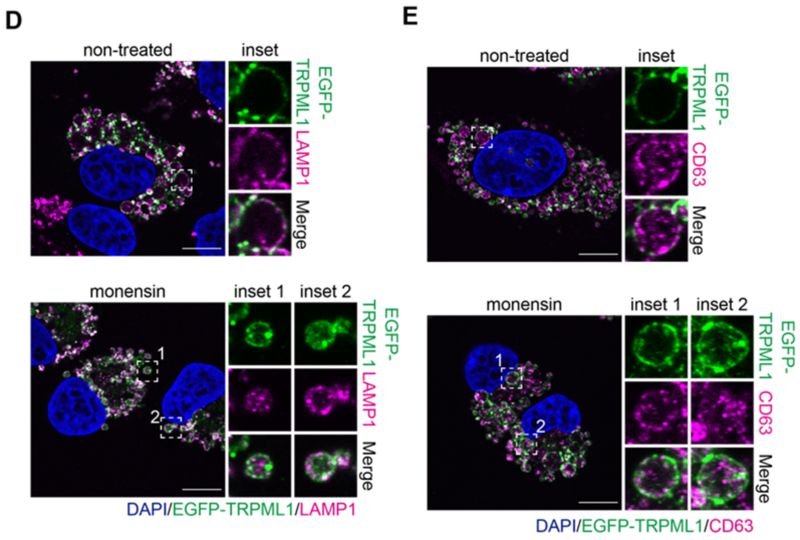

Fig EV4D and E

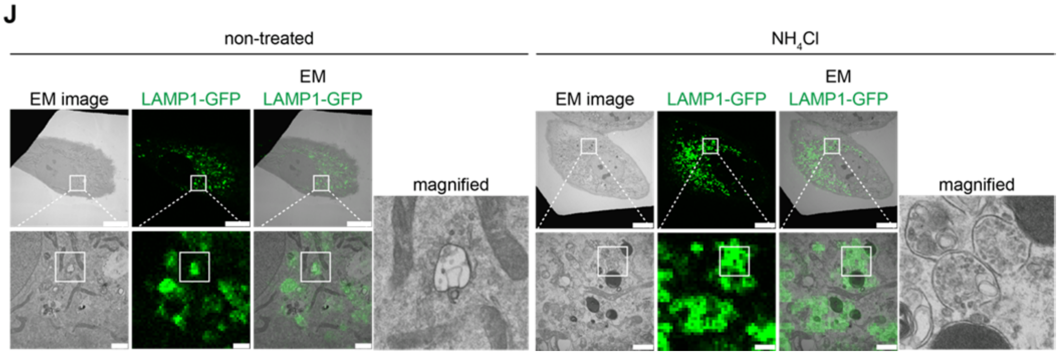

Fig 3J

11) The authors analyzed proteins the are phosphorylated in an STK38 dependent manner using MassSpec analysis and identified 25 candidates of which 10 decreased the number of VPS4 dots per cell. They further showed that one of these proteins, DOK1, is recruited to lysosomes upon LLOMe treatment. This study is extremely preliminary and does not add much value to the manuscript. It is not clear why DOK1 is recruited and whether this depends on STK38 kinase activity. Furthermore, DOK1 appears to be insight LAMP1 positive structure, i.e. in the lumen of lysosomes suggesting that it is degraded upon LLOMe treatment in an lysosome dependent manner. The meaning and significance of this observation was not further evaluated.

We totally agree with the reviewer's comments, and addressed several experiments to reveal how DOK1 contributes to STK38-VPS4 axis as follows.

Confirmation of STK38-mediated phosphorylation of DOK1

We first checked if the specific site of DOK1 is indeed phosphorylated by STK38 upon LLOMe treatment. From TMT-based phosphoproteomics, S269 of DOK1 was identified as a putative phosphorylation site by STK38. To confirm this, we quantified phosphorylation of this site by Parallel Reaction Monitoring-Mass Spectrometry (PRM-MS). This analysis revealed that S269 of DOK1 was phosphorylated after LLOMe treatment, and it was suppressed in STK38 depleted cells (**Fig 5A and B** in the revised manuscript).

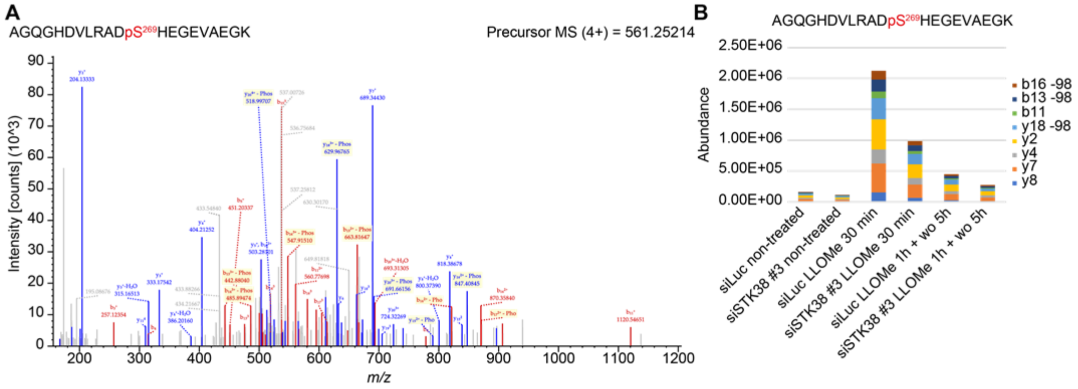

Fig 5A and B

Contribution of STK38-mediated DOK1 phosphorylation to VPS4 recruitment

We next tested whether the phosphorylation of DOK1 S269 is required for VPS4 recruitment. Expression of DOK1 WT but not phosphor-dead S269A mutant rescued VPS4 recruitment to lysosomes in DOK1 depleted cells, suggesting this phosphorylation is important for VPS4 recruitment to lysosomes (**Figs 5C, D, and EV4D**).

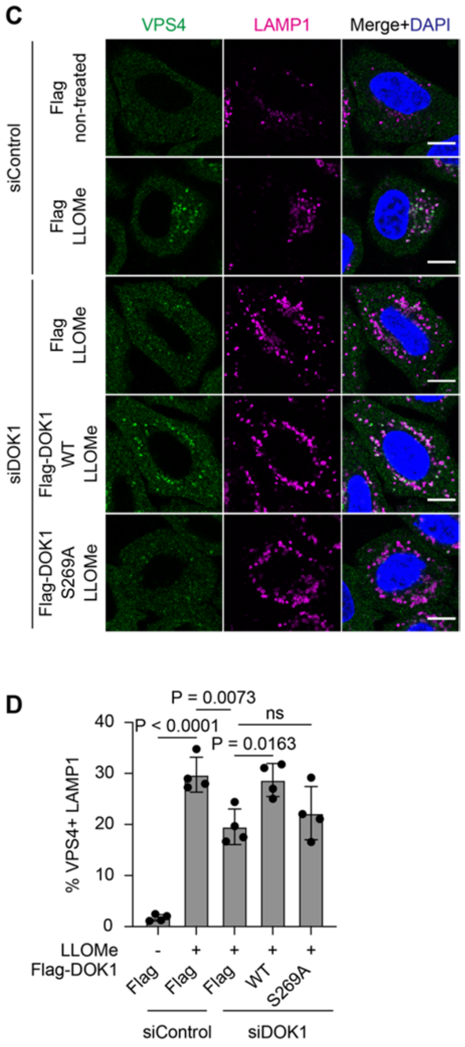

Figs 5C, D (left), and EV4D (right)

Lysosomal localization of DOK1 after LLOMe treatment

Next, we tested whether DOK1 recruitment on damaged lysosomes depends on STK38. As shown in **Fig 5E and F**, co-localization rate between mNG-DOK1 and LAMP1 after LLOMe treatment was significantly suppressed in STK38 KD cells, suggesting that STK38 recruits DOK1 to lysosomes. Then we checked whether phosphorylation of DOK1 S269 is required for its lysosomal localization. Phospho-dead mutation on S269 suppressed co-localization between DOK1 and LAMP1 after LLOMe treatment, suggesting importance of phosphorylation of this site for lysosomal localization (**Fig 5G and H**). As the reviewer pointed out, we also noticed that DOK1 is inside of LAMP1 positive structure. Actually, this localization is very similar to that of STK38, further implying they work together (**Figs 4G and EV1D**). This point is also mentioned in the result section of the revised manuscript (page 8, line 274).

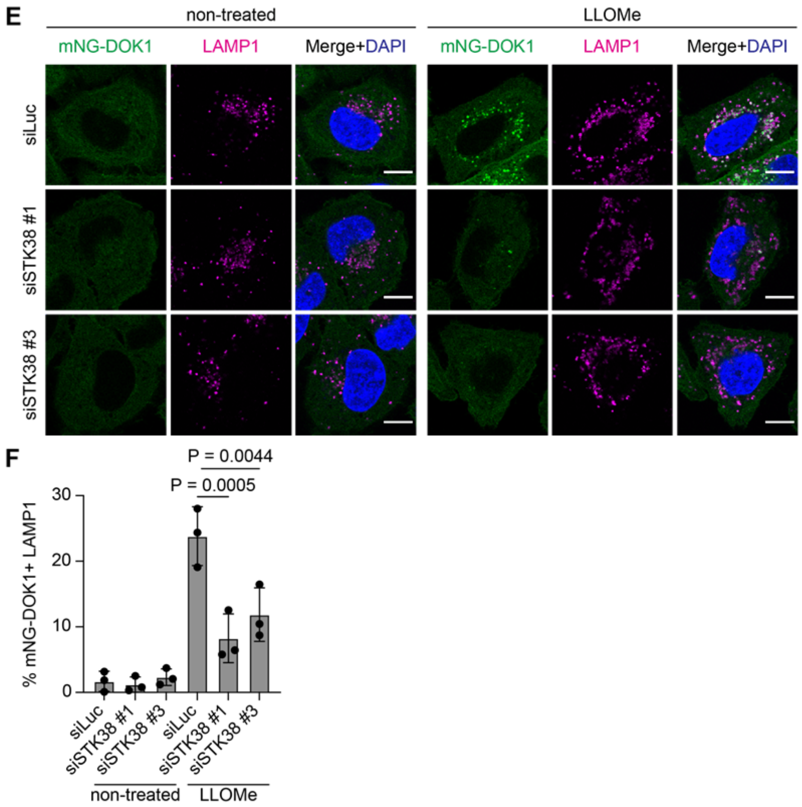

Fig 5E and F

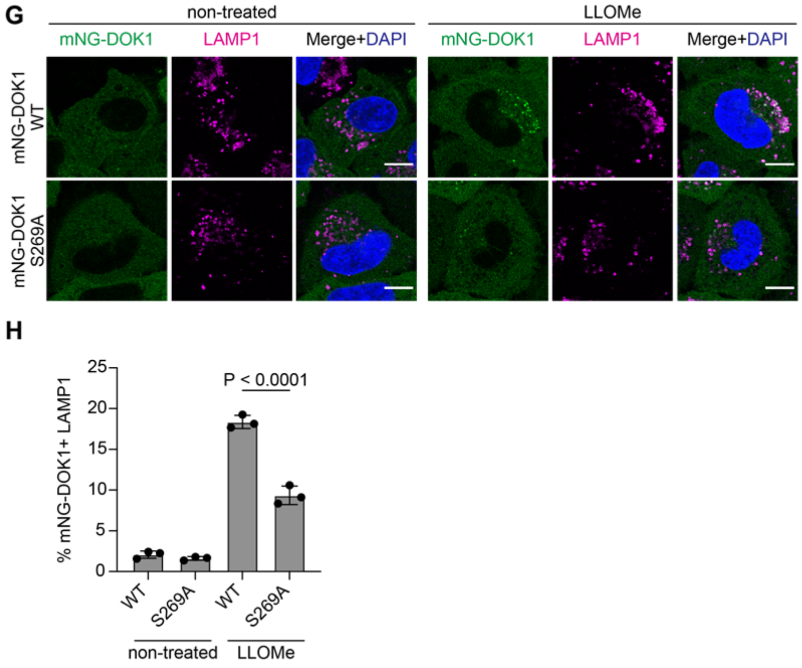

Fig 5G and H

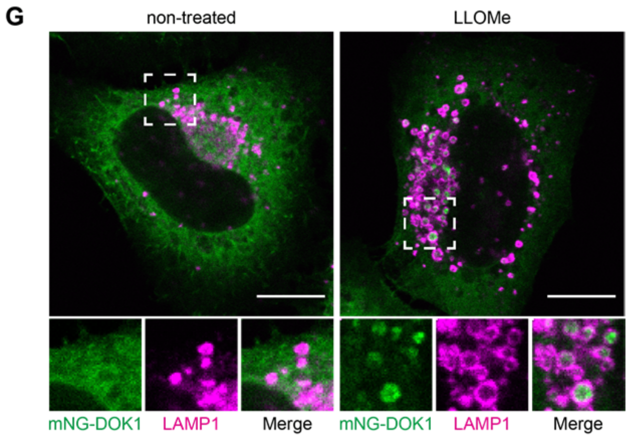

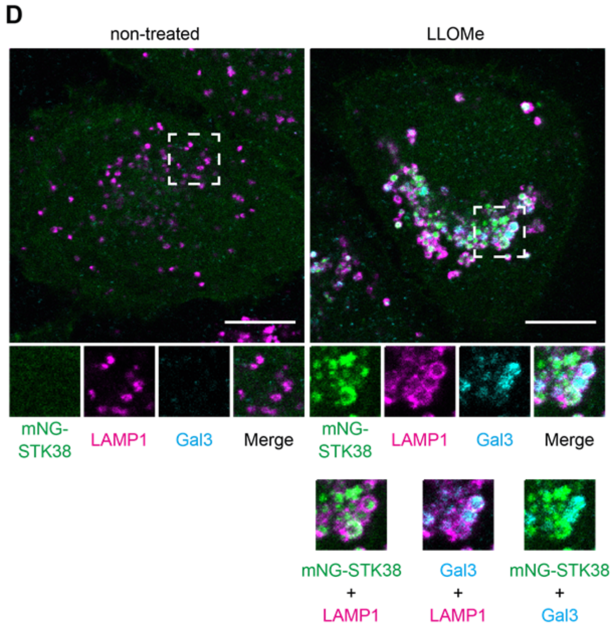

Figs 4G (upper) and EV1D (lower)

Interaction between STK38, DOK1, and VPS4

Furthermore, we tested if STK38, DOK1, and VPS4 work as a complex. Co-immunoprecipitation experiment showed interaction between STK38, DOK1 and VPS4 (**Fig EV4E**). Of note, this interaction was detected without LLOMe treatment, probably due to overexpression of Flag-DOK1.

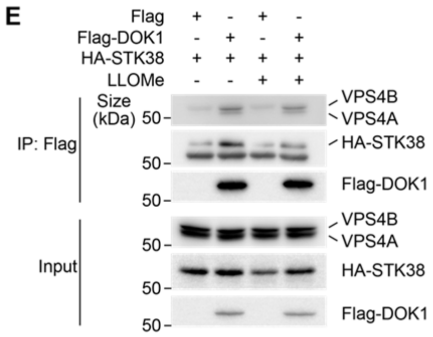

Fig EV4E

In conclusion, it is suggested that STK38 phosphorylates DOK1 on S269, which is required for lysosomal localization of DOK1 and subsequent recruitment of VPS4. We speculate that by forming ternary complex, DOK1 could be act as the docking site of VPS4 on lysosomes to terminate microlysophagy.

12) The authors analyzed the contribution of ATG8 proteins to the recruitment of ESCRT proteins to lysosomes after inducing lysosomal damage. They found that cells that recruitment of CHMP4B to lysosomes in hexa-KO cells expressing LC3C, GR-L1 or GR-L2 was restored. Although this recruitment depends on LLOMe treatment, it is not clear whether puncta represent lysosomes and whether this is related to microautophagy. The authors should i) co-stain using lysosomal markers and ii) distinguish macroautophagy from microautophgy. The latter is in general not very well-established by the experiments in this manuscript.

Regarding to point i), we have analyzed the co-localization rate between CHMP4B and LAMP1 in hexa KO cells expressing six ATG8 paralogs. Among them, only GABARAP family proteins could rescue CHMP4B recruitment to LAMP1 (**Fig 6G-I** in the revised manuscript).

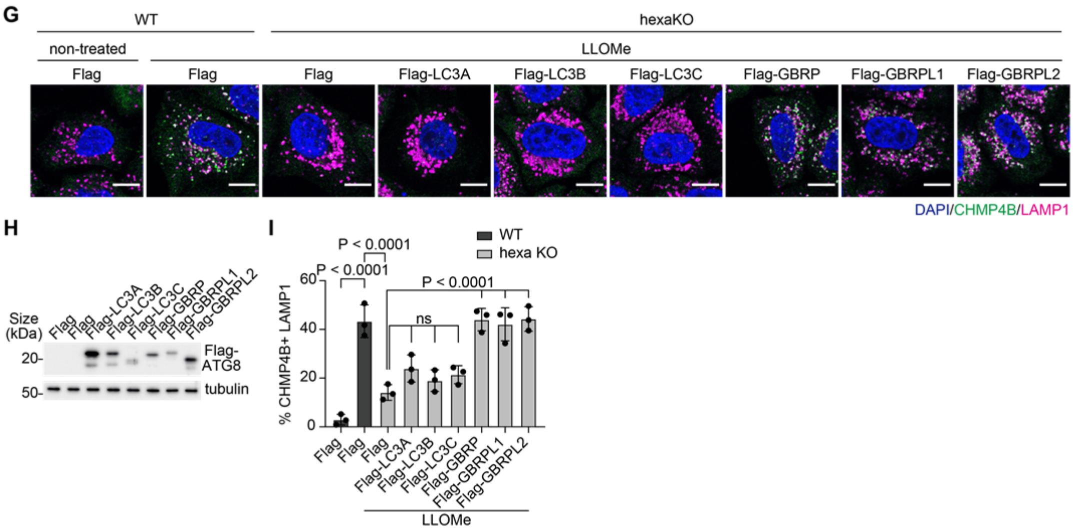

Fig 6G-I

Regarding to point ii), we have performed the GFP-Gal3 dots count assay, and the EGFP-TRPML1 cleavage assay in GABARAPs KO/KD cells to check whether GABARAPs are required for microlysophagy. As shown below, deletion of GABARAPs suppressed the clearance of GFP-Gal3 dots after LLOMe treatment (**Fig 6J and K**) as well as the cleavage of EGFP-TRPML1 after nigericin treatment (**Fig 6L and M**). These results suggest that GABARAPs are required for lysosomal damage response, especially microlysophagy. Specificity of EGFP-TRPML1 assay and lysosomal stress inducers (nigericin) to detect microlysophagy but not macrolysophagy is shown and explained in the response to question 8 and 9.

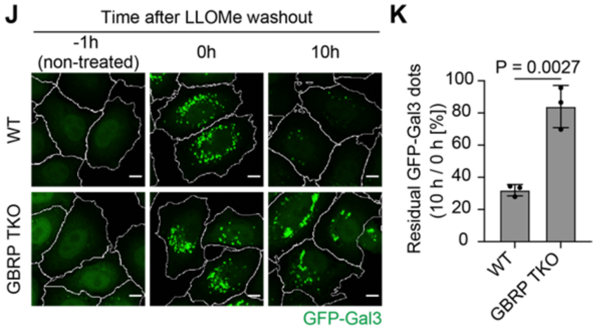

Fig 6J and K

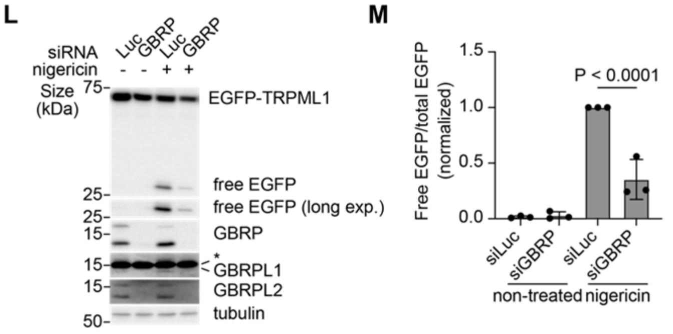

Fig 6L and M

13) The observation of the authors that KD of STK38 and GR induces senescence is interesting, but it remains unclear how this is related to microlysophagy, given that both proteins play also important roles in the regulation of macroautophagy. Overall, the connection between senescence, longevity and microlysophagy appears premature.

Thank you so much for pointing this out. We have evaluated senescence-induced lysosomal damage and found that knockdown of STK38 or GABARAPs further increased damaged lysosomes labeled by Gal3 compared to control cells (**Fig 7I and J** in the revised manuscript), suggesting that both are important to maintain lysosomal homeostasis during cellular senescence. Similar tendency was obtained in *sax-1* or *lgg-1* (homolog of GABARAPs) but not in *lgg-2* (homolog of LC3s) depleted worms (**Fig 7K-N** in the revised manuscript). Collectively, we believe that these data manifest our conclusion that lysosomal homeostasis regulated by STK38 and GABARAPs and presumably through microlysophagy is essential to prevent aging at cellular and organismal level.

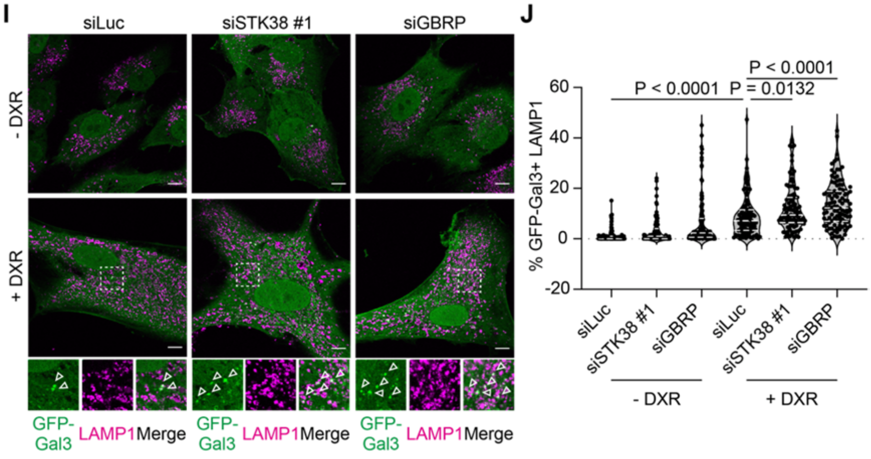

Fig 7I and J

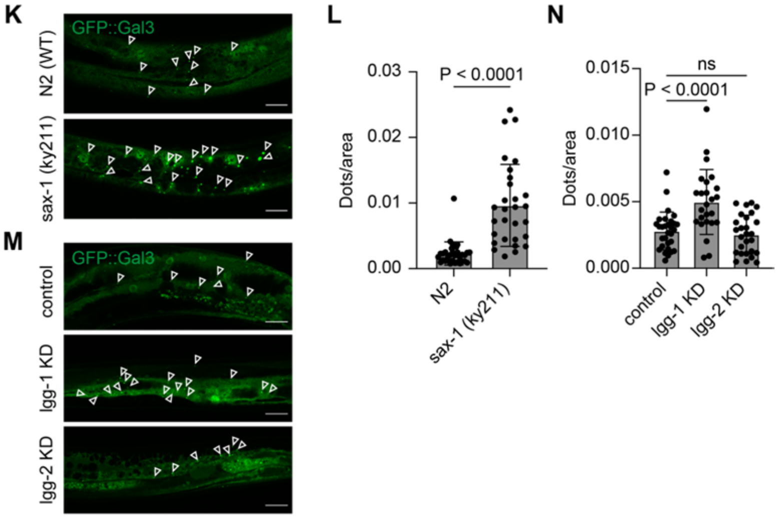

Fig 7K-N

Not all references are correctly given, please check the formatting of the bibliography.

Yes, we checked once again all formatting. Thank you!

Referee #2:

(Our responses are shown in blue)

First of all, we thank the reviewer for investing tremendous efforts and time to review our paper. We sincerely appreciate the reviewer's constructive and valuable comments. All of the comments raised are quite helpful to improve our manuscripts. The specific point-by-point responses are detailed below.

Recent years led to the discovery of several intracellular mechanisms used to recover lysosomal damage. These include macrolysophagy (a process extensively studied by Dr. Yoshimori and colleagues); ESCRT-dependent repair and the recently reported ESCRT-independent repair governed by ER-lysosome membrane contacts (Tan and Finkel Nature 2022 and Radulovic et al. EMBO J. 2022). The latter was neglected by the authors of the present study.

We deeply apologize for this insufficient citation. We have added all these references in the introduction part in the revised manuscript.

In the present study, Ogura et al. identified STK38, a serine/threonine kinase implicated in multiple cellular pathways, including cell cycle regulation and apoptosis, as a critical factor regulating ESCRT-dependent repair of lysosomal damage. The authors found that STK38 is incorporated into damaged lysosomes in a T444 phosphorylation and Ca+2-dependent manner, where it recruits VPS4, a key regulator of ESCRT function. The authors also provide evidence for the involvement of GABARAP proteins in assembling the ESCRT machinery on the damaged lysosomal membranes by interaction with the early-acting ESCRT component, ALIX. Additionally, the knockdown of STK38 and GABARAPs increases cellular sensitivity to senescence. The C. elegans orthologues of these factors were also tested for their involvement in aging.

The authors provide solid data indicating the recruitment of STK38 to the damaged lysosome. Lysosomal membrane damage, determined by the incorporation of Galectin 3 was shown to be affected by a lack of STK38. However, the same assay for Galectin 3 is missing in the GABARAP knockout background.

We have performed Galectin3 dot count assay in GABARAPs TKO cells. The clearance of Galectin3 dots was significantly suppressed in GABARAPs TKO cells, indicating GABARAPs are required for lysosomal damage response (**Fig 6J and K** in the revised manuscript).

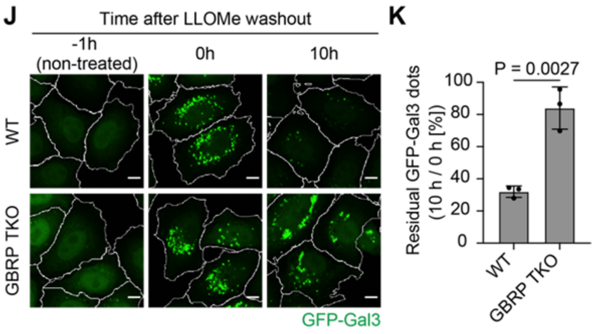

Fig 6J and K

Additionally, Galectin 3 localization should be determined in the background of the different STK38 mutants, including the phosphorylation mutants and the C' and N' truncated STK38 constructs.

Actually, the dynamic range and sensitivity of the Gal3 assay were not high enough to evaluate the effect of reconstitution of STK38. Therefore, instead of this assay, we have performed the rescue experiment by colocalization study of VPS4 and LAMP1, and confirmed that expression of STK38 WT but not all mutants could rescue VPS4 recruitment to lysosomes (**Fig 4A-C** in the revised manuscript). These mutants we used in this study lack one of phosphorylation sites of STK38 (T74, S281, or T444), whose mutation abolished the kinase activity of STK38 (Tamaskovic et al, 2003). Given that, this result suggested that the kinase activity of STK38 is important for VPS4 recruitment.

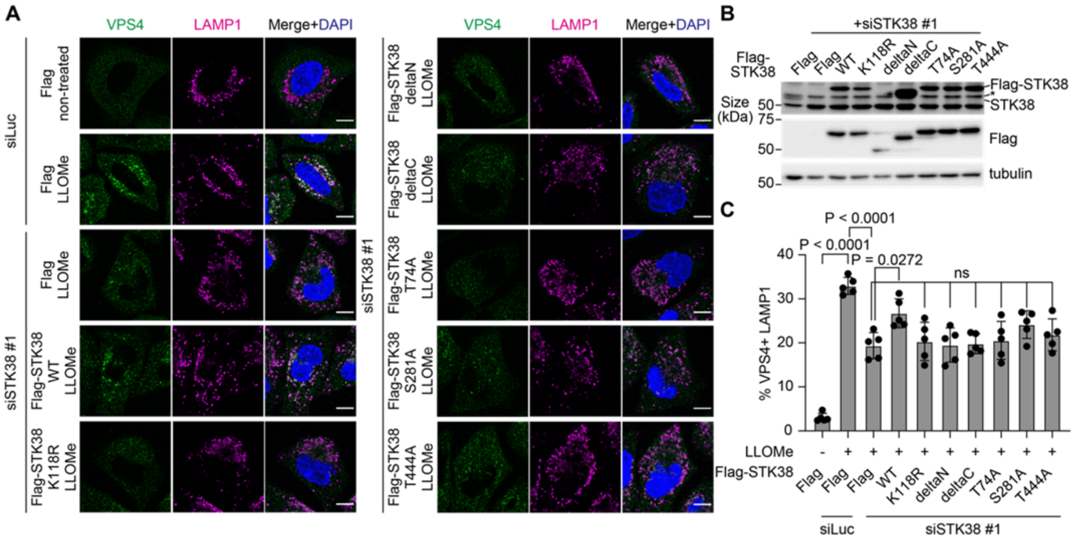

Fig 4A-C

In general, many aspects of this manuscript should be better characterized. For example, while the authors claim that VPS4 is recruited to lysosomal membranes by STK38, it remains unclear whether

STK38 directly associates with VPS4 or indirectly recruits it through DOK1 upon phosphorylation by STK38. The exact role of DOK1 in this process was also not established.

We totally agree with the reviewer's comments, and conducted several experiments to reveal how DOK1 contributes to STK38-VPS4 axis as follows.

Confirmation of STK38-mediated phosphorylation of DOK1

We first checked if the specific site of DOK1 is indeed phosphorylated by STK38 upon LLOMe treatment. From TMT-based phosphoproteomics, S269 of DOK1 was identified as a putative phosphorylation site by STK38. To confirm this, we quantified phosphorylation of this site by Parallel Reaction Monitoring-Mass Spectrometry (PRM-MS). This analysis revealed that S269 of DOK1 was phosphorylated after LLOMe treatment, and it was suppressed in STK38 depleted cells (**Fig 5A and B** in the revised manuscript).

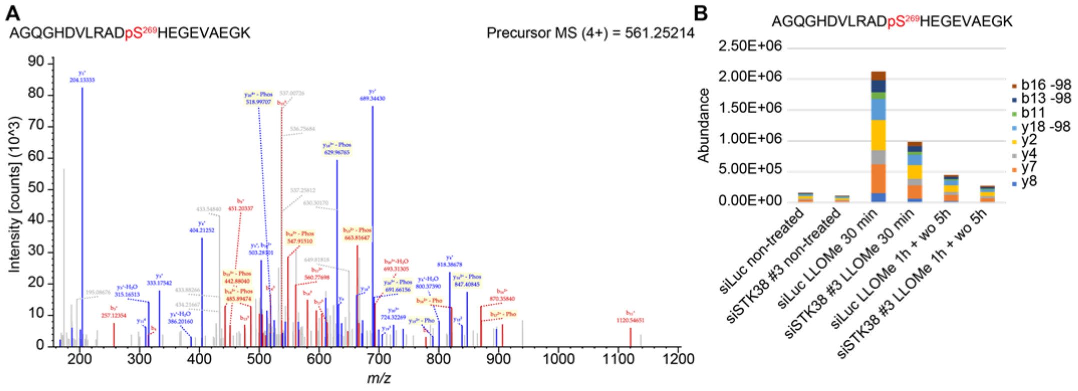

Fig 5A and B

Contribution of STK38-mediated DOK1 phosphorylation to VPS4 recruitment

We next tested whether the phosphorylation of DOK1 S269 is required for VPS4 recruitment. Expression of DOK1 WT but not phosphor-dead S269A mutant rescued VPS4 recruitment to lysosomes in DOK1 depleted cells, suggesting this phosphorylation is important for VPS4 recruitment to lysosomes (**Figs 5C, D, and EV4D**).

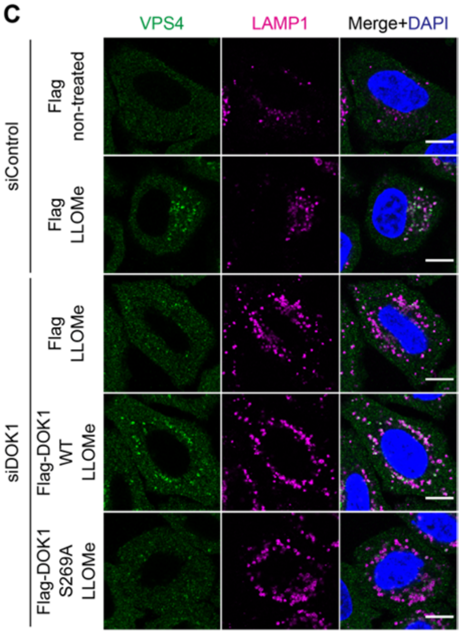

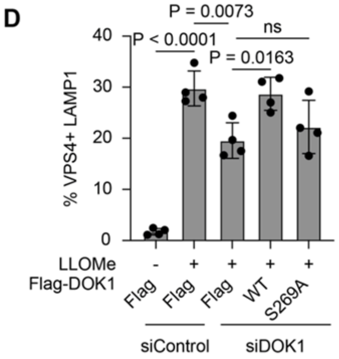

Figs 5C, D (left), and EV4D (right)

Lysosomal localization of DOK1 after LLOMe treatment

Furthermore, we tested whether DOK1 recruitment on damaged lysosomes depends on STK38. As shown in **Fig 5E and F**, co-localization rate between mNG-DOK1 and LAMP1 after LLOMe treatment was significantly suppressed in STK38 KD cells, suggesting that STK38 recruits DOK1 to lysosomes. Then we checked whether phosphorylation of DOK1 S269 is required for its lysosomal localization. Phospho-dead mutation on S269 suppressed co-localization between DOK1 and LAMP1 after LLOMe treatment, suggesting importance of phosphorylation of this site for lysosomal localization (**Fig 5G and H**).

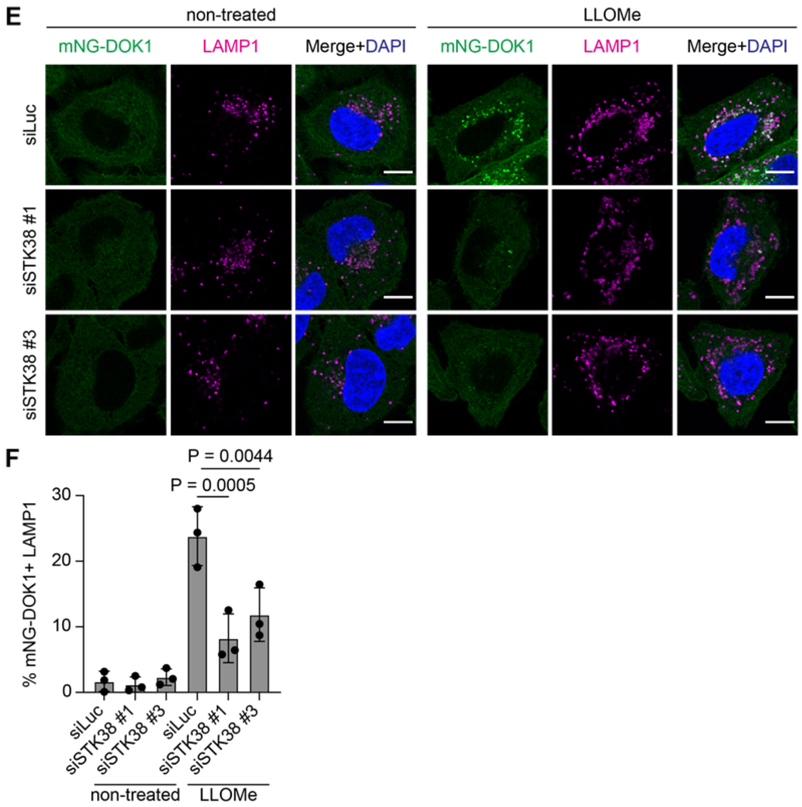

Fig 5E and F

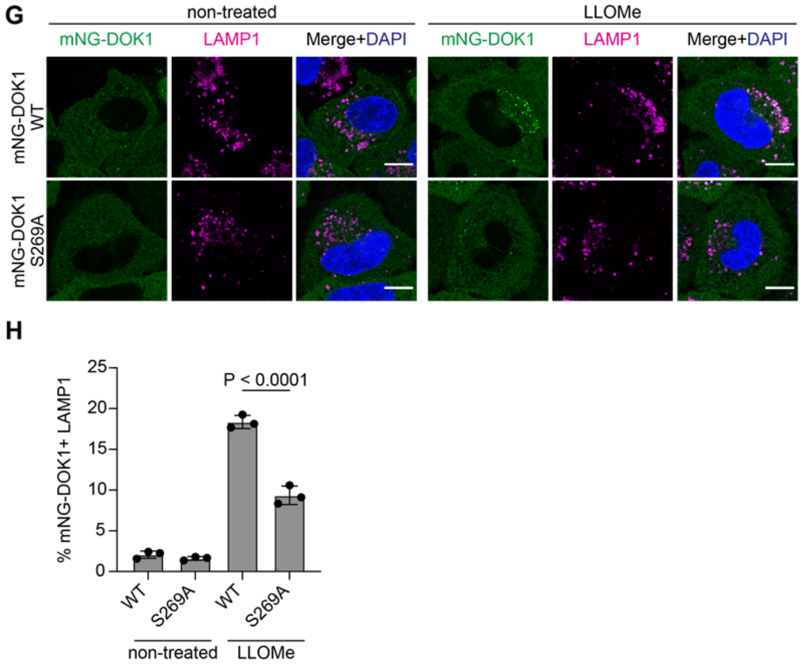

Fig 5G and H

Interaction between STK38, DOK1, and VPS4

Furthermore, we tested if STK38, DOK1, and VPS4 work as a complex. Co-immunoprecipitation experiment showed interaction between STK38, DOK1 and VPS4 (**Fig EV4E**). Of note, this interaction was detected without LLOMe treatment, probably due to overexpression of Flag-DOK1.

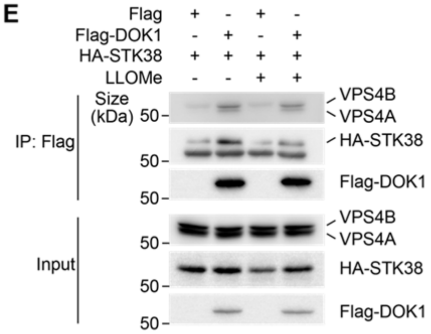

Fig EV4E

In conclusion, it is suggested that STK38 phosphorylates DOK1 on S269, which is required for lysosomal localization of DOK1 and subsequent recruitment of VPS4. We speculate that by forming ternary complex, DOK1 could be act as the docking site of VPS4 on lysosomes to terminate microlysophagy.

The authors should explain why they focused on STK38, while the original data obtained for MOB2 were more convincing.

Thank you for your good suggestions. First of all, we have chosen STK38 as our main-focusing target since it was thought to play a role in macroautophagy (Joffre et al, 2015; Klimek et al, 2019; Martin et al, 2019), and we first hypothesized its contribution to lysosomal damage response via macrolysophagy (as a result we have concluded that STK38 was required for microautophagy). In addition, previous study has demonstrated that MOB2 could bind to STK38 and act as co-factor of STK38 (Bothos et al, 2005; Devroe et al, 2004), which also prompted us to investigate STK38 as a novel regulator of lysosomal damage response.

Given reviewers' suggestions, we have performed GFP-Gal3 dot count screening again including MOB2 KD sample using siRNA with confirmed high knockdown efficiency (see **Appendix Fig S1** for the knockdown efficiency of these siRNAs). For the multiple comparison, we did this screening for three times. Similar to our previous screening, STK38 and MOB2 KD consistently increased the remaining Gal3 dots, suggesting that both components are required for clearance of damaged lysosomes (**Fig EV1A and B**). In addition, depletion of MST2, an upstream kinase of STK38 in Hippo pathway, also showed strong impact on it.

Based on previous findings and our results, in the current study, we mainly pursued roles of STK38, since it is implicated to play a role in autophagy and locates in the center of MST2-STK38-MOB2 cascade. We mentioned these results, possibility and explanation in the result and discussion part (page 4, line 122 and page 12, line 433). We hope that our explanation and revision are reasonable for the reviewer.

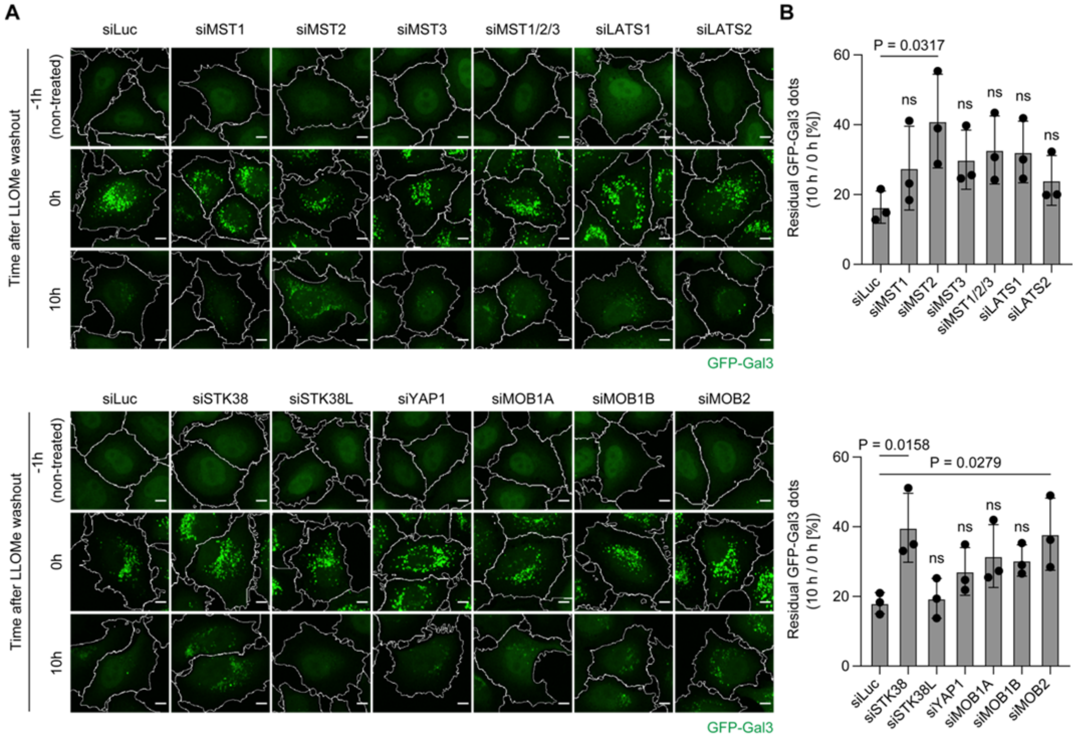

Fig EV1A and B

Finally, no physiological conclusions may be drawn according to LC3s role in senescence assay results since the knockdown of LC3s didn't work (Fig. EV7).

Although we have tested other siRNAs against LC3s, we failed to find efficient siRNAs. Therefore, we omitted results of LC3 KD from **Fig 7C, D and F** and **Appendix Fig S7B** in the revised manuscript.

Additional comments

1. Figure 1 A-B - the comparison between WT siSTK38 and FIP200 KO siSTK38 is missing. The accumulation of GFP-Gal3 in FIP200 KO siSTK38 is minor.

We guess the reviewer pointed out Fig 2A and B. We apologize for lack of indication of difference between WT siSTK38 and FIP200KO siSTK38 in the figure. As shown in **Fig 2B**, The accumulation

of GFP-Gal3 in FIP200 KO siSTK38 was statistically significantly increased compared to WT siSTK38. We have added that statistical result to the **Fig 2B** in the revised manuscript.

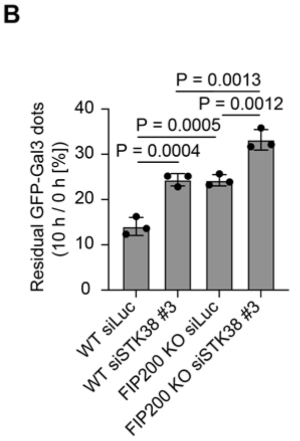

Fig 2B

2. Figure 2 C-D - that data present a negative result that does not contribute to the figure - should be moved to supplementary information.

As the reviewer suggested, we have transferred that data to **Fig EV2E and F**. Thank you.

3. Figure 3 D and F - the cleavage assay of GFP-TRPM1 is not convincing. The amount of free GFP is similar and hardly detected even in the positive control.

Regarding the free GFP band, we will also include the blots of long-exposure time together like following blots (**Fig 3F** in the revised manuscript) to show the difference in a convincing manner in the revised manuscript.

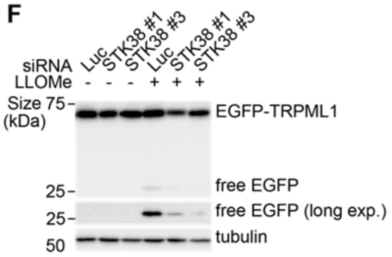

Fig 3F

4. Figure 3 H - the TEM images should be supported by CLEM or by using appropriate markers in IF.

We have observed ILVs containing compartment with lysosomal and MVB markers (LAMP1 and

CD63, respectively) by confocal microscopy or CLEM. First, we have performed IF-based observation of EGFP-TRPML1 positive ILVs in pharmacologically swollen LAMP1 or CD63 positive compartments. As shown in **Fig EV4D and E** in the revised manuscript, EGFP-TRPML1 positive ILVs were observed in either LAMP1 or CD63 positive endolysosomal compartment after monensin treatment. We also have performed CLEM using LAMP1-GFP expressing cells and observed ILVs in LAMP1-GFP positive lysosomal compartment (**Fig 3J**). These results suggest that microlysophagy could occur LAMP1 and/or CD63 positive endolysosomes. Of note, even these two membrane proteins LAMP1 and CD63 are enriched on lysosomes or MVBs, respectively and thus often used as each marker, it is difficult to dissect these two compartments clearly by them, since their localization is not specific on single compartment and overlapped between lysosomes and endosomes. In conclusion, our results suggest that EGFP-TRPML1 positive ILVs are formed at least LAMP1 positive lysosomal compartment.

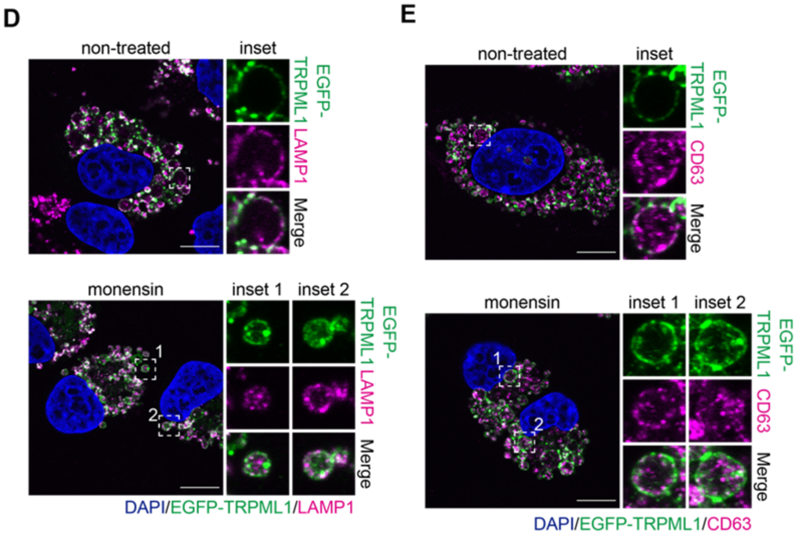

Fig EV4D and E

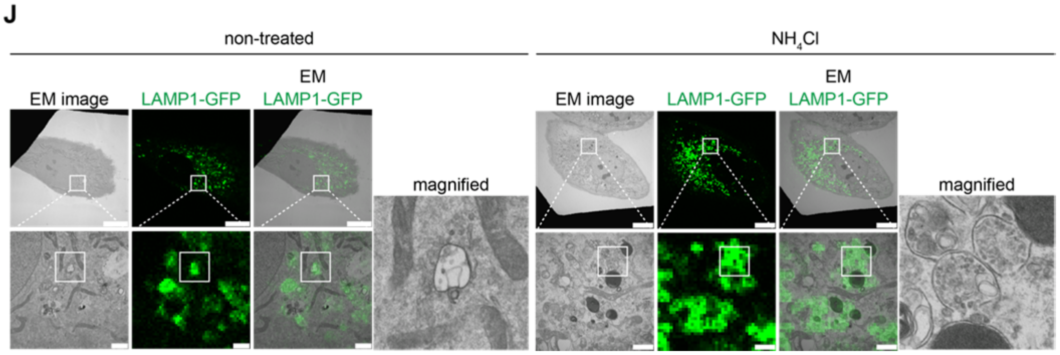

Fig 3J

5. Figure 4A-B - the data obtained by these experiments are inconclusive, possibly because the phospho-mimicking mutant was expressed on the background of endogenous STK38.

In Fig.4A-B of our initial manuscript, we overexpressed STK38 WT and kinase dead K118R mutant to see whether the kinase activity of STK38 is sufficient for enhancement of microlysophagy. The EGFP-TRPML1 cleavage assay was the only assay to evaluate microlysophagy so far. However, it needs to be improved in terms of the sensitivity, and we have confirmed that this assay was not enough to evaluate the effect of reconstitution of STK38. Given this limitation, we have performed the rescue experiment by colocalization study of VPS4 and LAMP1. We have confirmed that expression of STK38 WT but not K118R mutant could rescue VPS4 recruitment to lysosomes highlighting the requirement of kinase activity of STK38 to microlysophagy (**Fig 4A-C**). Of note, we have transferred the data of EGFP-TRPML1 cleavage assay in STK38 WT and K118R expressed cells to **Fig EV4A and B** in the revised manuscript.

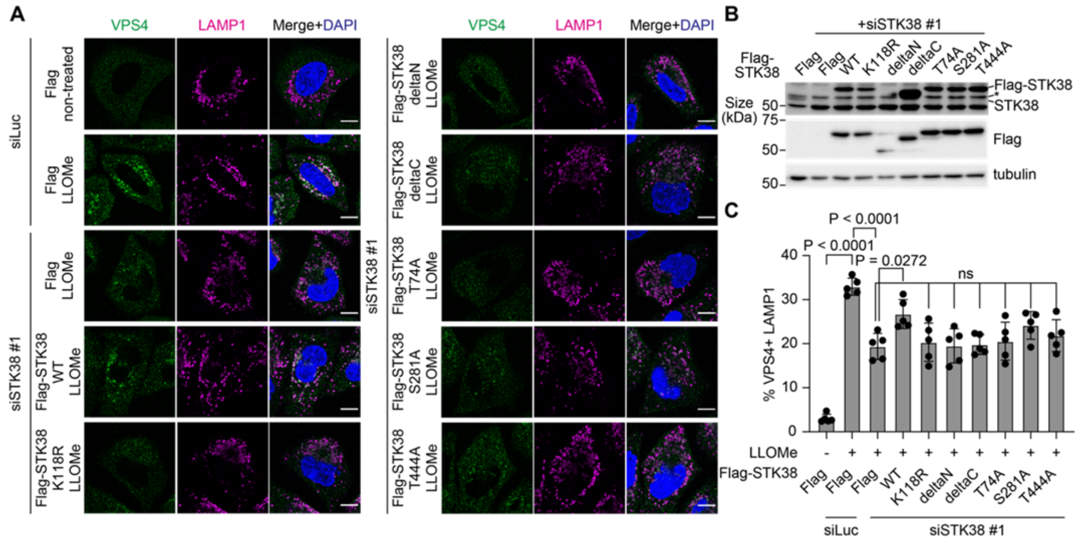

Fig 4A-C

6. Figure 5K -quantification and statistical information are needed.

We have added the quantification data and statistical information to this result (**Fig 6G-I** in the revised manuscript).

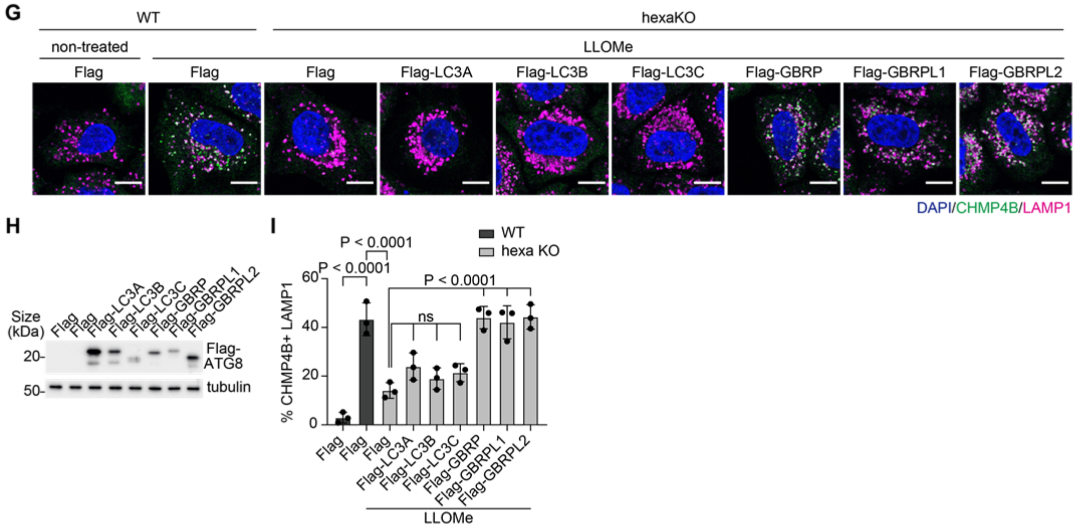

Fig 6G-I

7. Figure 5L - the IP results are unclear. It appears as LLOMe treatment reduces ALIX association with mCherry-GABARAPL2. In addition, the blot lacks the IP with mCherry alone as a control. The reciprocal IP may support the authors' claims.

We speculate that the interaction between mCherry-GABARAPL2 and ALIX in non-treated cells is due to the overexpression of mCherry-GABARAPL2, since we could see partial lysosomal localization of mCherry-GL2 even in non-treated cells. Apart from this, we performed this co-IP experiment using Flag-tagged LC3B and GABARAPL2 including Flag-tag alone samples, and quantified co-immunoprecipitated ALIX normalized to Flag-ATG8 (we used Flag tag instead of mCherry tag since we could not prepare mCherry alone construct immediately). As shown below, ALIX was co-immunoprecipitated much more with Flag-GABARAPL2 than LC3B while no interaction was appeared in tag alone samples (asterisk indicates non-specific band) (**Fig EV5E**). Again, the interaction was observed in non-treated cell probably due to the overexpression. In addition to the mCherry-tag IP, we have added the result of Flag-tag IP in **Fig EV5E** in the revised manuscript. As the reviewer pointed out, the reciprocal IP would support our claims. However, we prioritized other critical experiments first and had no time to try this experiment in the limited time. Thank you.

E

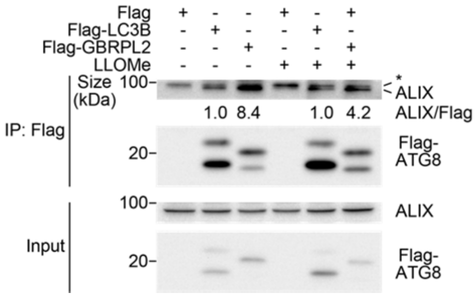

Fig EV5E

8. Figure 6 A and C - the western blots should include siLC3s and siGABARAPs. The decrease in SA-β-Gal positive cells or reduction of senescence markers upon recovery of STK38 and GABARAPs may support the suggested model.

Thank you so much for pointing this out. As mentioned in above, we failed to find efficient siRNAs for LC3s. Therefore, we have omitted results of siLC3s. Besides this, blots of GABARAPs are included in the revised manuscript (asterisk in GBRPL1 blots indicates non specific band) (**Fig 7C**). As the reviewer pointed out, the rescue experiment would support our working model. However, we are so sorry that we did not have enough time to investigate this point furthermore in during the revision period. We would like to access the connection between microlysophagy and aging further in our future study.

C

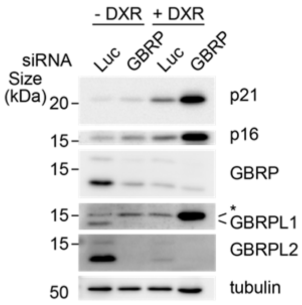

Fig 7C

9. Figure 6K-L - the probability of survival upon depletion of the different orthologs should be tested under lysosomal stress and not only under basal conditions.

Thank you for pointing this out. Although we have tried to conduct lifespan on NGM plate containing LLOMe, we could not find appropriate condition to give worms appropriate lysosomal

stress for the lifespan experiments. We would like to definitely optimize this condition in our future study with updated mechanistic insight.

10. Supplementary figure EV3 D-E - the effect of STK38 is not convincing. The cell in the lower left corner looks like the control cells.

We have changed the representative image of VPS4 in LLOMe-treated STK38 KO cells. In addition, we have added LAMP1 image to visualize co-localization between VPS4 and LAMP1. These results are now in **Appendix Fig S3E**. Although we did this experiment 3 times and confirmed the statistically significant difference between WT and STK38, we also feel that the phenotype of STK38 KO is slightly weaker than STK38 KD. We speculate this could be due to the compensation by STK38L, a paralog of STK38. We have mentioned this weak phenotype of KO cells and its possible reason in the revised manuscript (page 6, line 195).

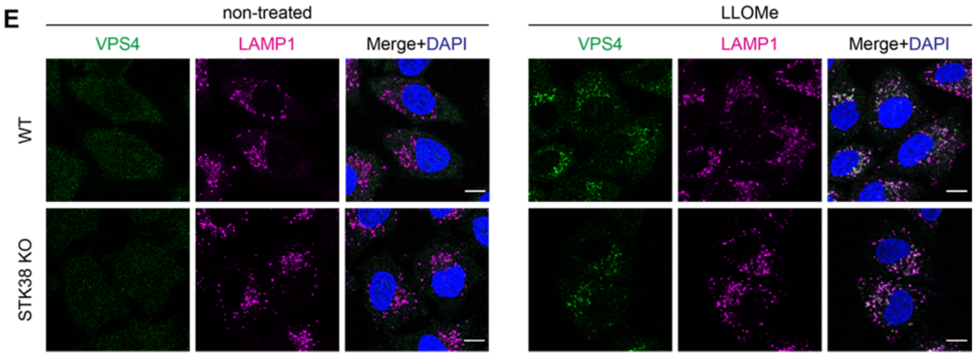

Appendix Fig S3E

1st Revision - Editorial Decision 18th Oct 2023

Dear Shuhei,

Thank you for the submission of your revised manuscript to EMBO reports. Former referee #1 has evaluated the revised manuscript, also taking into account your responses to the concerns from referee #2, who was not available.

As you will see, the referee is very positive about the study and requests only minor changes to clarify text and conclusions.

From the editorial side, there are also a few things that we need before we can proceed with the official acceptance of your study.

- Please change the name "Data and code availability" to "Data availability" and don't forget to remove the reviewer password.

- Please remove the Author Contributions from the manuscript file and make sure that the author contributions in our online submission system are correct and up-to-date. The information you specified in the system will be automatically retrieved and typeset into the article. You can enter additional information in the free text box provided, if you wish.

- Please provide an ORCID ID for Dr. Yoshimori. You can find instructions on how to link your ORCID ID to your account in our manuscript tracking system in our Author guidelines
(<https://www.embopress.org/page/journal/14693178/authorguide#authorshipguidelines

- Please provide the synopsis image at a final size of 550 pixels width. At this size the text is not so easy to read, so please design the image and text for this small size. Note that the height can be up to 600 pixels, so you might want to rearrange. But most important: look at the image at its final size for a last quality control step.

- Radulovic M et al is a preprint. Please cite it as (preprint: Radulovic et al, 2022) in the text and add [PREPRINT] at the end of the citation in the reference list. We also need the bioRxiv doi (Author NAME1, Author NAME2 (YEAR) article title. bioRxiv doi [PREPRINT].

- In Appendix Fig. S2 and Fig. S4E&G the scale bars are barely visible. Can you make the lines thicker?

- Our production/data editors have asked you to clarify one point in the figure legends:
i) Please note that information related to n is missing in the legend of figure 4d.

- Figure 3D STK38 is too closely cropped. Please show a larger fraction of the blot. In case the lower MW band is unspecific, please indicate this in the figure and legend.

- In Fig. 3E I find it difficult to correlate the categories 'non-treated', 'nigericin' etc with the three bars it is supposed to refer to. Can you shift the treatments in the x-axis a bit more to the left, so that the line is underneath the three bars it refers to? The same is true for Fig. 3C, the line for non-treated extends into the nigericin conditions. (I have not checked other figures for that, similar points may apply)

- The abstract needs to be written in present tense. Please find my suggestion with minor changes below my signature.

- On a different note, I would like to alert you that EMBO Press offers a new format for a video-synopsis of work published with us, which essentially is a short, author-generated film explaining the core findings in hand drawings, and, as we believe, can be very useful to increase visibility of the work. This has proven to offer a nice opportunity for exposure i.p. for the first author(s) of the study. Please see the following link for representative examples and their integration into the article web page:
https://www.embopress.org/video_synopses
https://www.embopress.org/doi/full/10.15252/embj.2019103932

Please let me know, should you be interested to engage in commissioning a similar video synopsis for your work. According operation instructions are available and intuitive.

We look forward to seeing a final version of your manuscript as soon as possible.

With kind regards,

Martina

Martina Rembold, PhD
Senior Editor

PS: If you were able to submit the revised version before October 30, we could still publish it in our December issue and under the charge conditions that were valid when you submitted.

****

Referee #1:

The authors present here the revised version of their initial manuscript "Microautophagy regulated by STK38 and GABARAPs is essential to repair lysosomes and prevent aging". They addressed all points raised by reviewers 1 and 2 in their detailed point-to-point responses by providing many new experiments which clarified /resolved all concerns of the reviewers. Some minor criticism that remains concernce the importance of lysosomal repair by microautophagy for cellular senescence and aging. Although the authors included new data, the conclusion might not be fully supported and the authors are encouraged to reformulate their conclusions to avoid overly strong statements. Apart from this minor criticism, this manuscript is very well suited for publication in EMBO reports.

****

Abstract:
Lysosomes are degradative organelles and signaling hubs that maintain cell and tissue homeostasis, and lysosomal dysfunction is implicated in aging and reduced longevity. Lysosomes are frequently damaged, but their repair mechanisms remain unclear. Here, we demonstrate that damaged lysosomal membranes are repaired by microautophagy (a process termed "microlysophagy"), and identifiy key regulators of the first and last steps. We reveal the AGC kinaseSTK38 as a novel microlysophagy regulator. Through phosphorylation of the scaffold protein DOK1, STK38 is specifically required for the lysosomal recruitment of the AAA+ ATPase VPS4, which terminates microlysophagy by promoting the disassembly of ESCRT components. By contrast, microlysophagy initiation involves non-canonical lipidation of ATG8s, especially the GABARAP subfamily, which is required for ESCRT assembly through interaction with ALIX. Depletion of STK38 and GABARAPs accelerates DNA damage-induced cellular senescence in human cells and curtails lifespan in C. elegans, respectively. Thus, microlysophagy is regulated by STK38 and GABARAPs and is essential for maintaining lysosomal integrity and preventing aging.

Point-by-point responses to requests from the editors and reviewers

First of all, we are most grateful to the editors and the reviewers for the positive decision on our revised manuscript. We also appreciated the helpful and constructive comments on it. We have corrected our manuscript according to all the requests from the editors and reviewers. Please check the following point-by-point responses.

General notes:
Our responses to the requests are shown in blue
Changes and corrections are highlighted in yellow in the manuscript.

Editorial requests:
- Please change the name "Data and code availability" to "Data availability" and don't forget to remove the reviewer password.

We have changed the name of this section and deleted the password.

- Please remove the Author Contributions from the manuscript file and make sure that the author contributions in our online submission system are correct and up-to-date. The information you specified in the system will be automatically retrieved and typeset into the article. You can enter additional information in the free text box provided, if you wish.

We have deleted this section from the manuscript and confirmed that our registered information about the author contribution in the online submission system are correct.

- Please provide an ORCID ID for Dr. Yoshimori. You can find instructions on how to link your ORCID ID to your account in our manuscript tracking system in our Author guidelines
(<https://www.embopress.org/page/journal/14693178/authorguide#authorshipguidelines

Yes. We have provided it through the online system.

- Please provide the synopsis image at a final size of 550 pixels width. At this size the text is not so easy to read, so please design the image and text for this small size. Note that the height can be up to 600 pixels, so you might want to rearrange. But most important: look at the image at its final size for a last quality control step.

We have corrected the size of the synopsis image and rearranged it to improve its visibility.

- Radulovic M et al is a preprint. Please cite it as (preprint: Radulovic et al, 2022) in the text and add [PREPRINT] at the end of the citation in the reference list. We also need the bioRxiv doi (Author NAME1, Author NAME2 (YEAR) article title. bioRxiv doi [PREPRINT].

Agree. Since the article has already been published, we have updated the citation in the main text and the reference list.

- In Appendix Fig. S2 and Fig. S4E&G the scale bars are barely visible. Can you make the lines thicker?

We have put thicker scale bars to these panels.

- Our production/data editors have asked you to clarify one point in the figure legends:
i) Please note that information related to n is missing in the legend of figure 4d.

We have added the information about the biological replicate of the experiment in the legend of Fig 4D (page 28, line 1024).

- Figure 3D STK38 is too closely cropped. Please show a larger fraction of the blot. In case the lower MW band is unspecific, please indicate this in the figure and legend.

We have replaced the blot with a larger one, and the lower unspecific band was indicated both in the figure and legend (page 27, line 993-994).

- In Fig. 3E I find it difficult to correlate the categories 'non-treated', 'nigericin' etc with the three bars it is supposed to refer to. Can you shift the treatments in the x-axis a bit more to the left, so that the line is underneath the three bars it refers to? The same is true for Fig. 3C, the line for non-treated extends into the nigericin conditions. (I have not checked other figures for that, similar points may apply)

We rearranged the position and the length of lines in the Fig 3E to make readers recognize easily what categories of treatment conditions refer to the bars. In addition, we have checked all the other figures and rearranged some figures in the same way.

- The abstract needs to be written in present tense. Please find my suggestion with minor changes below my signature.

Agree. We have edited the abstract in the present tense according to your suggestion. Thank you for your great suggestion!

- On a different note, I would like to alert you that EMBO Press offers a new format for a video-synopsis of work published with us, which essentially is a short, author-generated film explaining the core findings in hand drawings, and, as we believe, can be very useful to increase visibility of the work. This has proven to offer a nice opportunity for exposure i.p. for the first author(s) of the study. Please see the following link for representative examples and their integration into the article web page:
https://www.embopress.org/video_synopses
https://www.embopress.org/doi/full/10.15252/embj.2019103932

Thank you for offering the video-synopsis. I am afraid we wiIl be declining this time, but we would like to try it in the next time.

Minor errors:
Besides from editorial requests, we noticed some minor errors which do not affect our conclusion after submission of our revised manuscript. Here is the list of corrections in the final version of our manuscript. Of note, all of the corrections are highlighted in yellow in the manuscript.

Manuscript
-page 28, line 1027: (incorrect) comparisons shown in (C) → (correct) comparisons shown in (D)
-page 29, line1065: addition of “See also” information same as in other figures.

Figures
-Fig EV2F: Replacement of the graph (P-values were incorrect in the previous version).
-Appendix Fig S3A: Addition of some elements indicating molecular weights to blots.
-Appendix Fig S6B (fluorescent images of downstream candidates of STK38): The conditions taking these images were different between candidates. So, these images will be divided into individual panels.

Comments from the referee #1:
The authors present here the revised version of their initial manuscript "Microautophagy regulated by STK38 and GABARAPs is essential to repair lysosomes and prevent aging". They addressed all points raised by reviewers 1 and 2 in their detailed point-to-point responses by providing many new experiments which clarified /resolved all concerns of the reviewers. Some minor criticism that

remains concernce the importance of lysosomal repair by microautophagy for cellular senescence and aging. Although the authors included new data, the conclusion might not be fully supported and the authors are encouraged to reformulate their conclusions to avoid overly strong statements. Apart from this minor criticism, this manuscript is very well suited for publication in EMBO reports.

We thank the reviewer for investing tremendous efforts and time to evaluate our revised manuscript. We sincerely appreciate the reviewer's constructive and valuable comments. As the reviewer pointed out, we have no direct evidence of the preventive role of microlysophagy against aging and our conclusion needs to be reformulated with a weaker expression. Therefore, we have changed related sentences in the manuscript as follows: abstract (page 2, line 41-44), introduction (page 3-4, line 105-107), discussion (page 11, line 375-376 and page 12, line 438-440), and Figure 8 legend (page 31, line 1150-1151).

2nd Revision - Editorial Decision 25th Oct 2023

Prof. Shuhei Nakamura
Nara Medical University
Department of Biochemistry
840 Shijo-cho
Kashihara, Nara 634-8521
Japan

Dear Prof. Nakamura,

I am very pleased to accept your manuscript for publication in the next available issue of EMBO reports. Thank you for your contribution to our journal.

At the end of this email I include important information about how to proceed. Please ensure that you take the time to read the information and complete and return the necessary forms to allow us to publish your manuscript as quickly as possible.

As part of the EMBO publication's Transparent Editorial Process, EMBO reports publishes online a Review Process File to accompany accepted manuscripts. As you are aware, this File will be published in conjunction with your paper and will include the referee reports, your point-by-point response and all pertinent correspondence relating to the manuscript.

If you do NOT want this File to be published, please inform the editorial office within 2 days, if you have not done so already, otherwise the File will be published by default [contact: emboreports@embo.org]. If you do opt out, the Review Process File link will point to the following statement: "No Review Process File is available with this article, as the authors have chosen not to make the review process public in this case."

Thank you again for your contribution to EMBO reports and congratulations on a successful publication. Please consider us again in the future for your most exciting work.

Kind regards,

Martina Rembold, PhD
Senior Editor
EMBO reports

****

THINGS TO DO NOW:

Please note that you will be contacted by Wiley Author Services to complete licensing and payment information. The required 'Page Charges Authorization Form' is available here: https://www.embopress.org/pb-assets/embo-site/er_apc.pdf - please download and complete the form and return to embopressproduction@wiley.com

EMBO Press participates in many Publish and Read agreements that allow authors to publish Open Access with reduced/no publication charges. Check your eligibility: https://authorservices.wiley.com/author-resources/Journal-Authors/open-access/affiliation-policies-payments/index.html

You will receive proofs by e-mail approximately 2-3 weeks after all relevant files have been sent to our Production Office; you should return your corrections within 2 days of receiving the proofs.

Please inform us if there is likely to be any difficulty in reaching you at the above address at that time. Failure to meet our deadlines may result in a delay of publication, or publication without your corrections.

All further communications concerning your paper should quote reference number EMBOR-2023-57300V3 and be addressed to emboreports@wiley.com.

Should you be planning a Press Release on your article, please get in contact with emboreports@wiley.com as early as possible, in order to coordinate publication and release dates.

EMBO Press Author Checklist

Corresponding Author Name: Tamotsu Yoshimori and Shuhei Nakamura
Journal Submitted to: EMBO Reports
Manuscript Number: EMBOR-2023-57300

USEFUL LINKS FOR COMPLETING THIS FORM
The EMBO Journal - Author Guidelines
EMBO Reports - Author Guidelines
Molecular Systems Biology - Author Guidelines
EMBO Molecular Medicine - Author Guidelines

Reporting Checklist for Life Science Articles (updated January
This checklist is adapted from Materials Design Analysis Reporting (MDAR) Checklist for Authors. MDAR establishes a minimum set of requirements in transparent reporting in the life sciences (see Statement of Task: 10.31222/osf.io/9sm4x). Please follow the journal's guidelines in preparing your
Please note that a copy of this checklist will be published alongside your article.

Abridged guidelines for figures
1. Data
The data shown in figures should satisfy the following conditions:
- the data were obtained and processed according to the field's best practice and are presented to reflect the results of the experiments in an accurate and unbiased manner.
- ideally, figure panels should include only measurements that are directly comparable to each other and obtained with the same assay.
- plots include clearly labeled error bars for independent experiments and sample sizes. Unless justified, error bars should not be shown for technical
- if n<5, the individual data points from each experiment should be plotted. Any statistical test employed should be justified.
- Source Data should be included to report the data underlying figures according to the guidelines set out in the authorship guidelines on Data

2. Captions
Each figure caption should contain the following information, for each panel where they are relevant:
- a specification of the experimental system investigated (eg cell line, species name).
- the assay(s) and method(s) used to carry out the reported observations and measurements.
- an explicit mention of the biological and chemical entity(ies) that are being measured.
- an explicit mention of the biological and chemical entity(ies) that are altered/varied/perturbed in a controlled manner.
- the exact sample size (n) for each experimental group/condition, given as a number, not a range;
- a description of the sample collection allowing the reader to understand whether the samples represent technical or biological replicates (including how many animals, litters, cultures, etc.).
- a statement of how many times the experiment shown was independently replicated in the laboratory.
- definitions of statistical methods and measures:
  - common tests, such as t-test (please specify whether paired vs. unpaired), simple χ2 tests, Wilcoxon and Mann-Whitney tests, can be unambiguously identified by name only, but more complex techniques should be described in the methods section;
  - are tests one-sided or two-sided?
  - are there adjustments for multiple comparisons?
  - exact statistical test results, e.g., P values = x but not P values < x;
  - definition of 'center values' as median or average;
  - definition of error bars as s.d. or s.e.m.

Please complete ALL of the questions below.
Select "Not Applicable" only when the requested information is not relevant for your study.

Materials

Newly Created Materials	Information included in the manuscript?	In which section is the information available? (Reagents and Tools Table, Materials and Methods, Figures, Data Availability Section)
New materials and reagents need to be available; do any restrictions apply?	Not Applicable	

Antibodies	Information included in the manuscript?	In which section is the information available? (Reagents and Tools Table, Materials and Methods, Figures, Data Availability Section)
For **antibodies** provide the following information:<br>- Commercial antibodies: RRID (if possible) or supplier name, catalogue number and or/clone number<br>- Non-commercial: RRID or citation	Yes	Materials and Methods

DNA and RNA sequences	Information included in the manuscript?	In which section is the information available? (Reagents and Tools Table, Materials and Methods, Figures, Data Availability Section)
**Short novel DNA or RNA including primers, probes**: provide the sequences.	Yes	Materials and Methods, Appendix

Cell materials	Information included in the manuscript?	In which section is the information available? (Reagents and Tools Table, Materials and Methods, Figures, Data Availability Section)
**Cell lines:** Provide species information, strain. Provide accession number in repository **OR** supplier name, catalog number, clone number, and/**OR** RRID.	Yes	Materials and Methods
**Primary cultures:** Provide species, strain, sex of origin, genetic modification status.	Not Applicable	
Report if the cell lines were recently **authenticated** (e.g., by STR profiling) and tested for mycoplasma contamination.	Not Applicable	

Experimental animals	Information included in the manuscript?	In which section is the information available? (Reagents and Tools Table, Materials and Methods, Figures, Data Availability Section)
**Laboratory animals or Model organisms:** Provide species, strain, sex, age, genetic modification status. Provide accession number in repository **OR** supplier name, catalog number, clone number, **OR** RRID.	Yes	Materials and Methods
**Animal observed in or captured from the field:** Provide species, sex, and age where possible.	Not Applicable	
Please detail **housing and husbandry conditions**.	Yes	Materials and Methods

Plants and microbes	Information included in the manuscript?	In which section is the information available? (Reagents and Tools Table, Materials and Methods, Figures, Data Availability Section)
**Plants:** provide species and strain, ecotype and cultivar where relevant, unique accession number if available, and source (including location for collected wild specimens).	Not Applicable	
**Microbes:** provide species and strain, unique accession number if available, and source.	Not Applicable	

Human research participants	Information included in the manuscript?	In which section is the information available? (Reagents and Tools Table, Materials and Methods, Figures, Data Availability Section)
If collected and within the bounds of privacy constraints report on age, sex and gender or ethnicity for all study participants.	Not Applicable	

Core facilities	Information included in the manuscript?	In which section is the information available? (Reagents and Tools Table, Materials and Methods, Figures, Data Availability Section)
If your work benefited from core facilities, was their service mentioned in the acknowledgments section?	Yes	Acknowledgements

Design

Study protocol	Information included in the manuscript?	In which section is the information available? (Reagents and Tools Table, Materials and Methods, Figures, Data Availability Section)

If study protocol has been **pre-registered, provide DOI in the manuscript**. For clinical trials, provide the trial registration number **OR** cite DOI.	Not Applicable	
Report the **clinical trial registration number** (at ClinicalTrials.gov or equivalent), where applicable.	Not Applicable	

Laboratory protocol	Information included in the manuscript?	In which section is the information available? (Reagents and Tools Table, Materials and Methods, Figures, Data Availability Section)
Provide DOI OR other citation details if **external detailed step-by-step protocols** are available.	Not Applicable	

Experimental study design and statistics	Information included in the manuscript?	In which section is the information available? (Reagents and Tools Table, Materials and Methods, Figures, Data Availability Section)
Include a statement about **sample size** estimate even if no statistical methods were used.	Yes	Figure Legends
Were any steps taken to minimize the effects of subjective bias when allocating animals/samples to treatment (e.g. **randomization procedure**)? If yes, have they been described?	Not Applicable	
Include a statement about **blinding** even if no blinding was done.	Yes	Materials and Methods
Describe **inclusion/exclusion criteria** if samples or animals were excluded from the analysis. Were the criteria pre-established?<br><br>If sample or data points were omitted from analysis, report if this was due to attrition or intentional exclusion and provide justification.	Not Applicable	
For every figure, are **statistical tests** justified as appropriate? Do the data meet the assumptions of the tests (e.g., normal distribution)? Describe any methods used to assess it. Is there an estimate of variation within each group of data? Is the variance similar between the groups that are being statistically compared?	Yes	Figure Legends

Sample definition and in-laboratory replication	Information included in the manuscript?	In which section is the information available? (Reagents and Tools Table, Materials and Methods, Figures, Data Availability Section)
In the figure legends: state number of times the experiment was **replicated** in laboratory.	Yes	Figure Legends
In the figure legends: define whether data describe **technical or biological replicates.**	Yes	Figure Legends

Ethics

Ethics	Information included in the manuscript?	In which section is the information available? (Reagents and Tools Table, Materials and Methods, Figures, Data Availability Section)
Studies involving **human participants**: State details of **authority granting ethics approval** (IRB or equivalent committee(s), provide reference number for approval.	Not Applicable	
Studies involving **human participants**: Include a statement confirming that **informed consent** was obtained from all subjects and that the experiments conformed to the principles set out in the WMA Declaration of Helsinki and the Department of Health and Human Services Belmont Report.	Not Applicable	
Studies involving **human participants:** For publication of **patient photos**, include a statement confirming that consent to publish was obtained.	Not Applicable	
Studies involving experimental **animals**: State details of **authority granting ethics approval** (IRB or equivalent committee(s), provide reference number for approval. Include a statement of compliance with ethical regulations.	Not Applicable	
Studies involving **specimen and field samples:** State if relevant **permits** obtained, provide details of authority approving study; if none were required, explain why.	Not Applicable	

Dual Use Research of Concern (DURC)	Information included in the manuscript?	In which section is the information available? (Reagents and Tools Table, Materials and Methods, Figures, Data Availability Section)
Could your study fall under dual use research restrictions? Please check biosecurity documents and list of **select agents and toxins** (CDC): https://www.selectagents.gov/sat/list.htm	Not Applicable	
If you used a select agent, is the security level of the lab appropriate and reported in the manuscript?	Not Applicable	
If a study is subject to dual use research of concern regulations, is the name of the **authority granting approval and reference number** for the regulatory approval provided in the manuscript?	Not Applicable	

Reporting

The MDAR framework recommends adoption of discipline-specific guidelines, established and endorsed through community initiatives. Journals have their own policy about requiring specific guidelines and recommendations to complement MDAR.

Adherence to community standards	Information included in the manuscript?	In which section is the information available? (Reagents and Tools Table, Materials and Methods, Figures, Data Availability Section)
State if relevant guidelines or checklists (e.g., **ICMJE, MIBBI, ARRIVE, PRISMA**) have been followed or provided.	Not Applicable	
For **tumor marker prognostic studies,** we recommend that you follow the **REMARK** reporting guidelines (see link list at top right). See author guidelines, under 'Reporting Guidelines'. Please confirm you have followed these guidelines.	Not Applicable	
For **phase II and III randomized controlled trials**, please refer to the **CONSORT** flow diagram (see link list at top right) and submit the CONSORT checklist (see link list at top right) with your submission. See author guidelines, under 'Reporting Guidelines'. Please confirm you have submitted this list.	Not Applicable	

Data Availability

Data availability	Information included in the manuscript?	In which section is the information available? (Reagents and Tools Table, Materials and Methods, Figures, Data Availability Section)
Have **primary datasets** been deposited according to the journal's guidelines (see 'Data Deposition' section) and the respective accession numbers provided in the Data Availability Section?	Yes	Data Availability Section
Were **human clinical and genomic datasets** deposited in a public access-controlled repository in accordance to ethical obligations to the patients and to the applicable consent agreement?	Not Applicable	
Are **computational models** that are central and integral to a study available without restrictions in a machine-readable form? Were the relevant accession numbers or links provided?	Not Applicable	
If publicly available data were reused, provide the respective **data citations in the reference list**.	Not Applicable	
